# Transcriptome Analysis of mRNA and miRNA in Somatic Embryos of *Larix leptolepis* Subjected to Hydrogen Treatment

**DOI:** 10.3390/ijms17111951

**Published:** 2016-11-22

**Authors:** Yali Liu, Suying Han, Xiangming Ding, Xinmin Li, Lifeng Zhang, Wanfeng Li, Haiyan Xu, Zhexin Li, Liwang Qi

**Affiliations:** 1 State Key Laboratory of Tree Genetics and Breeding, Research Institute of Forestry, Chinese Academy of Forestry, Beijing 100091, China; liuyali2008goto@163.com (Y.L.); XinminLi@mednet.ucla.edu (X.L.); zhanglifeng1029@163.com (L.Z.); liwf@caf.ac.cn (W.L.); xuhy_lily@163.com (H.X.); lizhexin_8903@163.com (Z.L.); 2 Key Laboratory of Research Institute of Forest Ecology and Protection, Chinese Academy of Forestry, Beijing 100091, China; syhan@caf.ac.cn; 3 Department of Pathology and Laboratory Medicine, University of California at Los Angeles, 1000 Veteran Ave., Los Angeles, CA 90095, USA; XDing@mednet.ucla.edu

**Keywords:** hydrogen, somatic embryogenesis, pro-embryogenic mass, miRNA, reactive oxygen species (ROS) homeostasis, cell cycle

## Abstract

Hydrogen is a therapeutic antioxidant that has been used extensively in clinical trials. It also acts as a bioactive molecule that can alleviate abiotic stress in plants. However, the biological effects of hydrogen in somatic embryos and the underlying molecular basis remain largely unknown. In this study, the morphological and physiological influence of exogenous H_2_ treatment during somatic embryogenesis was characterized in *Larix leptolepis* Gordon. The results showed that exposure to hydrogen increased the proportions of active pro-embryogenic cells and normal somatic embryos. We sequenced mRNA and microRNA (miRNA) libraries to identify global transcriptome changes at different time points during H_2_ treatment of larch pro-embryogenic masses (PEMs). A total of 45,393 mRNAs and 315 miRNAs were obtained. Among them, 4253 genes and 96 miRNAs were differentially expressed in the hydrogen-treated libraries compared with the control. Further, a large number of the differentially expressed mRNAs and miRNAs were related to reactive oxygen species (ROS) homeostasis and cell cycle regulation. We also identified 4399 potential target genes for 285 of the miRNAs. The differential expression data and the mRNA-miRNA interaction network described here provide new insights into the molecular mechanisms that determine the performance of PEMs exposed to H_2_ during somatic embryogenesis.

## 1. Introduction

Molecular hydrogen (H_2_) was first identified as an antioxidant that could selectively reduce cytotoxicity in animals [1]. Since then, the role of hydrogen in repairing many clinical disorders related to oxidative stress has been reported [2,3,4,5,6,7,8]. Hydrogen also acts as an antioxidant or signaling molecule in plant developmental processes and responses to environmental stresses. Hydrogen promotes adventitious root formation and stomatal closure [9,10], and can enhance tolerance to various abiotic stresses, including high salinity [11,12,13], low temperature [13], drought [10,13], and high light [14] by modulating reactive oxygen species (ROS) homeostasis.

ROS, such as superoxide anions (O^2−^), hydroxyl radicals (OH^−^), and hydrogen peroxide (H_2_O_2_), are generated and detoxified to maintain a fine-tuned balance in plants. To sustain normal cellular ROS metabolism, the expression of many genes is up or down-regulated at the transcriptional and translational levels. These genes encode proteins that participate in ROS-producing and ROS-scavenging processes, and play critical roles in plant growth, development, and adaptation to stress [15,16,17].

Studies using clinical and experimental animal models in which hydrogen has been applied by inhalation of hydrogen gas, consumption of hydrogen-rich water, injection with hydrogen-saturated saline, and culture in vitro on H_2_-rich media have revealed that hydrogen is an important bioactive factor with antioxidant, anti-inflammatory, and anti-apoptotic effects on cells, tissues, organs, and individuals against oxidative injury [1,2,3,4,5,6,7,8]. In plants, the protective roles have also been proven when seeds, seedlings, explants, and fruits were subjected to hydrogen treatment with hydrogen-rich water or media [9,10,11,12,13,14,18,19,20,21]. However, the biological effects of exogenous hydrogen on plant cells in vitro cultures have not been documented to date. Furthermore, although the functions of H_2_ have been reported to be closely correlated with the ROS system, the molecular mechanism has not been investigated by integrated mRNA and microRNA (miRNA) transcriptome analysis.

Japanese larch (*Larix leptolepis* Gordon) is an important species for several of its particularities, such as fast growth, adaptability to severe environments, and its ecological and economic value. Somatic embryos (SEs) are useful tools to obtain saplings with desirable reproductive and cloning characteristics, and are also useful for studying the molecular mechanisms underlying embryo pattern formation [22]. The somatic embryogenic process involves two main phases: pro-embryogenic masses (PEMs) and SEs. In the PEM phase, PEMs pass through three characteristic stages distinguished by cellular organization and cell number, from a primitive single cell aggregate to aggregates of several cells, and then to cellular clusters, which can never develop directly into real embryos. In the SE phase, PEMs are transdifferentiated to SEs [23]. Japanese larch somatic embryos, as a suitable and valuable system, have been previously used to investigate embryogenesis patterns at the morphological, physiological, and molecular levels [24,25,26,27,28,29,30].

In the present study, we aimed to investigate the effects of exogenous H_2_ treatment during the somatic embryogenesis process using a larch SE in vitro culture system. To further examine the gene network underlying how H_2_ affects somatic embryogenesis, we performed RNA-sequencing analyses of mRNA and miRNA expression profiles in PEMs on H_2_-enriched and control media. The interactions between miRNAs and their target genes were also characterized. These analyses will contribute to a more comprehensive understanding of the regulatory mechanisms involved in the effects of hydrogen on somatic embryogenesis.

## 2. Results

### 2.1. Hydrogen Improved the Survival Rates of Active PEMs and Normal SEs

**Figure 1:**
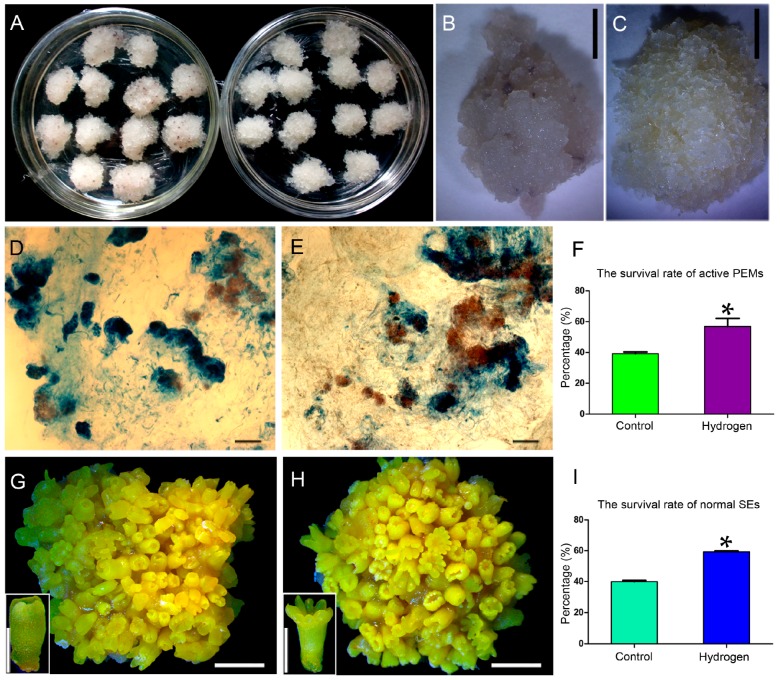
Effect of exogenous hydrogen treatment on pro-embryogenic masses (PEMs) and somatic embryos (SEs) during somatic embryogenesis. (**A**) PEMs sub-cultured on control medium (**left**) and hydrogen-rich medium (**right**). Magnified PEMs on control medium (**B**) and hydrogen-rich medium (**C**). Double-stained PEMs sub-cultured on control medium (**D**) and hydrogen-rich medium (**E**). Active pro-embryogenic cells stained red (acetic acid-carmine); inactive pro-embryogenic cells were stained blue (Evans blue). (**F**) Frequency of active PEMs on the different media. SEs sub-cultured on control medium (**G**), with insert showing an abnormal SE, and hydrogen-rich medium (**H**), with insert showing a normal SE. (**I**) Incidence of normal SEs sub-cultured on the different media. Data are presented as mean ± standard error from three independent experiments. Asterisks indicate significant difference at * *p* < 0.05 by Student’s *t* test. Scale bars, 5 mm in (**B**,**C**,**G**,**H**); 500 µm for insets (**G**,**H**); and 200 µm in (**D**,**E**).

To identify physiological changes, the morphology of PEMs sub-cultured for 36 days on control and hydrogen-rich media was observed (Figure 1). The calli were light yellow on the hydrogen-rich medium (Figure 1A,C), but pale and disintegrated on the control medium (Figure 1A,B). Cell death and survival rates of the PEMs were detected by acetic acid-carmine and Evans blue double staining (Figure 1D,E). The percentage of active PEMs on the hydrogen-rich medium (52.6%) was higher than on the control medium (39.7%) (Figure 1F).

To further explore the effects of hydrogen on SEs during the SE phase, PEMs were transferred to hydrogen-rich or control maturation media. The morphology of the SEs was observed and photographed after culturing for 42 days (Figure 1G,H). Normal SEs had whole cotyledons and radicles, and polar pattern formations could also be seen, while malformed SEs had abnormal shoot and/or root apexes, and/or abnormal cotyledons (inserts in Figure 1G,H). The percentage of normal SEs on the hydrogen-rich medium (59.3%) was higher than on the control medium (39.9%) (Figure 1I).

### 2.2. H_2_ Reduced Reactive Oxygen Species (ROS) Levels and Enhanced Antioxidant Enzyme Activity

To examine whether the influence of H_2_ on PEMs was tightly related to redox status, we measured ROS levels and the activities of several antioxidant enzymes. We found that ROS levels were lower in PEMs on hydrogen-rich medium compared with control medium (Figure S1A,B). The activities of SOD (superoxide dismutase), CAT (catalase), and POD (peroxidase) were significantly increased in PEMs after hydrogen treatment compared with untreated controls (Figure S1C–E). These results suggested that exposure to exogenous H_2_ decreased ROS levels and enhanced detoxification.

### 2.3. Changes in mRNA Expression in PEMs under Hydrogen Treatment

Twenty-two cDNA libraries from larch PEMs sub-cultured on hydrogen-rich and control media at six time points (0 h, 12 h, 48 h, 7 days, 21 days, 36 days), were sequenced. A total of 584,557,848 raw reads were obtained. After removing adaptor sequences, ambiguous nucleotides, and low-quality sequences, 572,103,351 clean reads remained. After de novo assembly of the clean reads, we obtained 62,729 transcripts and 45,393 unigenes with contig N50 lengths of 1304 and 1310 bp, respectively. Automatic annotation using Trinotate (Broad Institute of Massachusetts Institute of Technology and Harvard, Cambridge, MA, USA; Massachusetts Institute of Technology, Cambridge, MA, USA) and KASS (KEGG (Kyoto Encyclopedia of Genes and Genomes) Automatic Annotation Server, Kyoto University, Kyoto, Japan) produced 62,729 annotations for the assembled transcripts (Table S1).

**Figure 2:**
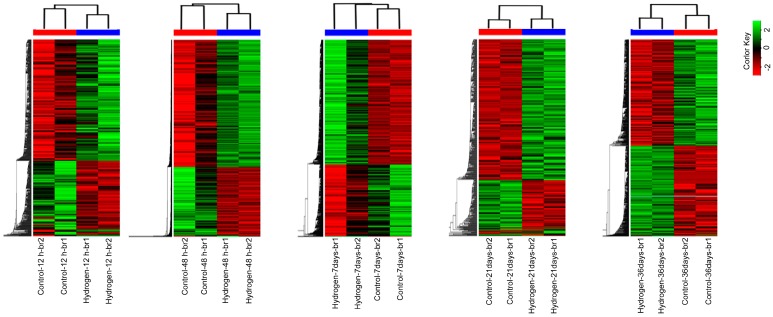
Hierarchical clustering maps of differentially expressed genes (DEGs) after 12 h, 48 h, 7 days, 21 days, and 36 days of hydrogen treatment. The color scale shows log_2_ fold changes (FC). FC = normalized TPM (transcripts per million) values in H_2_-treated PEMs/normalized TPM (transcripts per million) values in control PEMs. br1/br2: two biological replicates, respectively. Red color line indicates br1/br2 clustering of control PEMs and blue color line indicates br1/br2 clustering of H_2_-treated PEMs at each time point.

**Figure 3:**
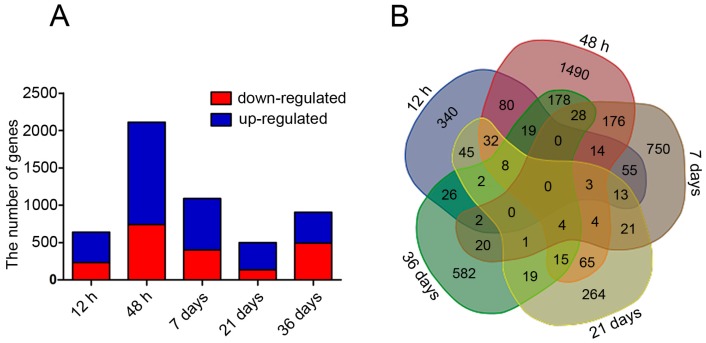
Changes in mRNA expression levels in H_2_-exposed PEMs. The histogram (**A**); and Venn diagram (**B**) show the number of DEGs at different time points.

Heat maps were constructed to show the transcript abundance in the PEMs sub-cultured on hydrogen-rich medium for 12 h, 48 h, 7 days, 21 days, and 36 days (Figure 2). We identified 4253 differentially expressed genes (DEGs) by comparing the hydrogen-treatment and control samples (Table S2); 1874 were down-regulated and 2379 were up-regulated (Figure 3A). Of these, 145 genes were expressed at more than three time points, as shown in the Venn diagram (Figure 3B). The largest number of DEGs was detected at 48 h after H_2_-treatment (Figure 3A). Moreover, 82 DEGs encoding proteins related to the ROS system, including antioxidant enzymes like SOD, CAT, and POD, and low molecular mass non-enzymatic antioxidants were identified (Table S3).

Sixty-four DEGs encoding proteins involved in cell cycle regulation, including DNA replication factors, chromosome organization and remodeling regulators, cyclins, cyclin-dependent kinases, cyclin-dependent kinase inhibitors, cell division control proteins, and retinoblastoma-related proteins, were identified (Table S4). Clearly, the global gene expression patterns were greatly altered in H_2_-exposed larch PEMs.

### 2.4. Validation of Differentially Expressed Genes (DEGs) by qPCR

**Figure 4:**
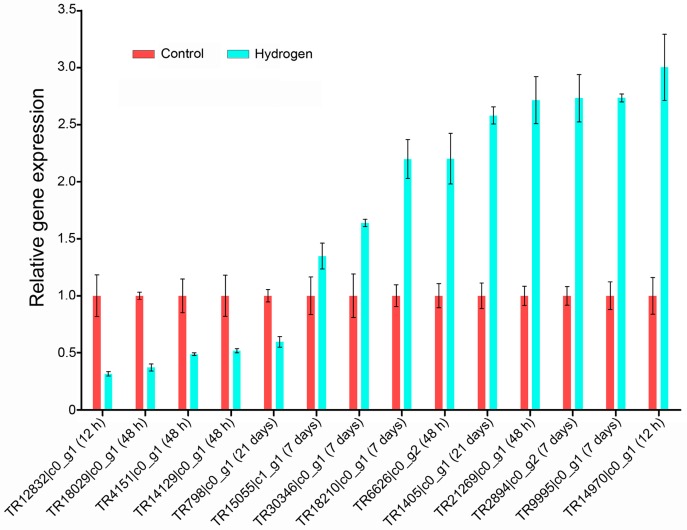
Verification of DEGs using qPCR (real-time quantitative PCR). Three biological repeats were reverse transcribed and amplified independently in the qPCR. The samples were quantified using *LaEF-1α* as a reference gene and the data are presented as mean ± standard error. The time point of differential expression is shown in parentheses for each gene.

To verify the differential expression identified by RNA-sequencing, 14 randomly selected DEGs were selected for qPCR (real-time quantitative PCR) analysis (Figure 4, Table S5). These genes were involved in electron transport, ROS homeostasis, transcription factor regulation, cellulose biosynthesis, cell proliferation, and protein transport. Most of the selected genes showed significant differences between the H_2_-treated and control PEMs, consistent with the RNA-seq data, confirming the reliability of the RNA-seq analysis of the *L. leptolepis* libraries.

### 2.5. Gene Ontology (GO) and Kyoto Encyclopedia of Genes and Genomes (KEGG) Enrichment Analysis

All the DEGs were mapped to the PANTHER, Gene Ontology, BioCyc, and KEGG PATHWAY databases using KOBAS. At 12 h, sulfate adenylyl transferase activity, auxin influx transmembrane transporter activity, plant seed peroxidase activity, and heme oxygenase (decyclizing) activity were the most enriched gene ontology (GO) terms, and selenate reduction, sulfate assimilation, sulfate reduction II, and 4-hydroxybenzoate biosynthesis I were the most enriched pathways. At 48 h, arginine catabolic process, negative regulation of cell proliferation, cinnamic acid metabolic process, and maintenance of organ identity were the most enriched GO terms, and anthocyanin biosynthesis, spermine and spermidine degradation III, and kaempferol glycoside biosynthesis were the most enriched pathways. At 7 days, 6-phosphofructokinase complex, cinnamic acid metabolic process, regulation of oxidoreductase activity, and ammonia-lyase activity were the most enriched GO terms, and homogalacturonan biosynthesis, purine deoxyribonucleosides salvage, cytidine monophosphate (CMP) phosphorylation, and adenosine deoxyribonucleotides de novo biosynthesis were the most enriched pathways. At 21 days, sesquiterpenoid catabolic process, apocarotenoid catabolic process, abscisic acid catabolic process, and lactoylglutathione lyase activity were the most enriched GO terms, and uridine triphosphate (UTP) and cytidine triphosphate (CTP) de novo biosynthesis, phase late biosynthesis, and de novo pyrimidine ribonucleotides biosynthesis were the most enriched pathways. At 36 days, RNA polymerase II core binding, voltage-gated potassium channel complex, cation channel complex, and potassium channel complex were the most enriched GO terms, while adenine and hypoxanthine salvage pathway, adenosine nucleotides degradation I, cutin biosynthesis, and purine nucleotides degradation I were the most enriched pathways (Table S6). Moreover, reactive oxygen species biosynthetic process, hydrogen peroxide biosynthetic process, and regulation of cell cycle were significantly enriched GO terms at 48 h, 7 days, 21 days, and 36 days after H_2_ treatment (Table S6).

GOseq was further employed to perform the GO enrichment analyses at the five time points for the up-regulated and down-regulated genes. In total, 20,976 genes from all the samples were classified under 803 functional terms; 183 for down-regulated genes and 620 for up-regulated genes. At all time points, oxidoreductase activity, external encapsulating structures, and cell walls were significantly enriched, while cellular macromolecule metabolic processes were greatly depressed (Table S7).

Additionally, many of these DEGs were annotated with GO terms and KEGG pathways related to redox metabolic processes, such as response to oxidative stress, reactive oxygen species biosynthetic process, reactive oxygen species metabolic process, antioxidant activity, l-ascorbate biosynthesis, and carotenoid biosynthesis (Tables S6 and S7). Taken together, these results provide insights into the cellular and molecular mechanisms of the response of larch PEMs to hydrogen treatment.

### 2.6. Effect of Hydrogen on miRNAs in Larch PEMs

Twenty-two small RNA libraries from larch PEMs, sub-cultured on hydrogen-rich and control media at five different time points, were sequenced. A total of 315 miRNAs were identified using MiRDeep2 (Table S8). The 315 miRNAs were compared to known miRNAs in miRBase; 49 of them matched 40 known miRNA families (Table S8). The most highly conserved miRNAs were found in more than 20 species; for instance, miR167 was found in 38 plant species. Nine miRNA families were found only in gymnosperms; for example, miR1311 and miR1314 were present in *Picea abies* and *Pinus taeda*. (Table S8). A further 266 miRNAs were identified as novel miRNAs, having a corresponding miRNA* and the characteristic hairpin structure of the precursor sequences (File S1).

**Figure 5:**
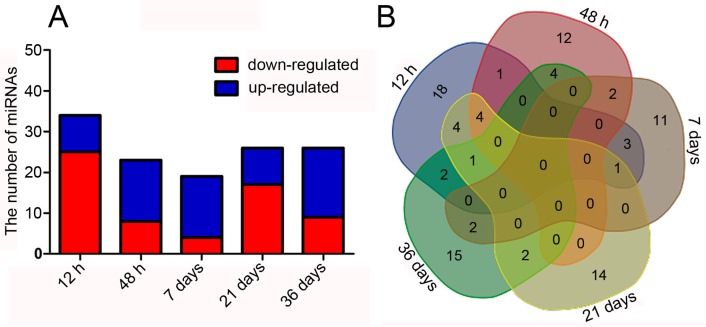
Changes in miRNA expression levels in H_2_-exposed PEMs. The histogram (**A**); and Venn diagram (**B**) show the number of differentially expressed miRNAs at different time points.

The expression levels of the miRNAs were estimated using the quantifier module in MiRDeep2. Consequently, 96 differentially expressed miRNAs were obtained; 47 were down-regulated and 49 were up-regulated (Figure 5A,B, Table S9). The maximum number of differentially expressed miRNAs was detected at 12 h after H_2_ treatment (Figure 5A, Table S9).

### 2.7. mRNA-miRNA Interaction Network

**Figure 6:**
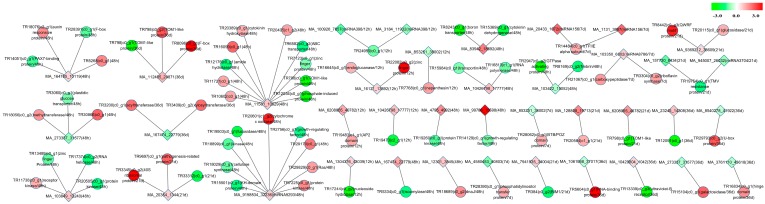
miRNA-mRNA interaction network. The network shows the predicted interactions between differently expressed miRNAs and their target DEGs in PEMs at five time points. Red indicates up-regulated mRNAs or miRNAs; green indicates down-regulated mRNAs or miRNAs. The color depth is based on the log_2_FC (Fold change H_2_-treatment/control). Circles indicate mRNAs; squares indicate miRNAs. An interaction is represented by one edge.

To understand the miRNA regulatory mechanisms underlying the improved physiological status of larch PEMs upon hydrogen exposure, the interactions between the miRNAs and their target mRNAs were investigated using PsRobot. We identified 4399 potential target genes for 285 of the miRNAs (Table S10). After 12 h, 48 h, 7 days, 21 days, and 36 days of hydrogen treatment, we found 8, 13, 5, 7, and 8 differently expressed miRNAs that putatively regulated 8, 40, 5, 9, and 10 DEGs, respectively (Figure 6, Table S10). Among them, 42 miRNA-mRNA pairs displayed negative correlation (Figure 6, Table S10).

## 3. Discussion

Molecular hydrogen plays a cytoprotective role by exerting protective effects against plant oxidative damage through the regulation of ROS homeostasis [9,10,11,12,13,14,18,19,20,21]. In this study, we showed that hydrogen treatment raised the frequency of active pro-embryogenic cells and normal somatic embryos at the PEM and SE phases, respectively (Figure 1). Furthermore, exogenous hydrogen application significantly reduced ROS levels and enhanced antioxidant enzyme activity in H_2_-treated PEMs (Figure S1). Considering that redox status is a crucial factor in determining plant development [31] and ROS homeostasis regulates somatic embryogenesis [32], we presume that the increased rate of active pro-embryogenic cells and normal somatic embryos may be, at least partially, a result of reduced ROS levels after H_2_ treatment.

To gain insight into the molecular mechanism underlying how H_2_ affects somatic embryogenesis, RNA-seq was subsequently performed. To our knowledge, this is the first global and high-throughput sequencing analysis of mRNA and miRNA expression in H_2_-exposed plant tissues. The mRNA-seq analysis detected 4253 genes that were differentially expressed at multiple time points after H_2_ treatment (Table S2). Eighty-two of these DEGs encoded proteins that are involved in ROS homeostasis, including 31 ROS-producing enzymes, 23 ROS-scavenging enzymes, and 28 antioxidant-related proteins (Table S3), and many other DEGs were found to be involved in other redox metabolic processes (Tables S6 and S7). Furthermore, we found DEGs of the ROS system, response to oxidative stress, reactive oxygen species biosynthetic process, and carotenoid biosynthesis consist of both up-regulated and down-regulated genes (Figure 4, Tables S2 and S5–S7). In the ROS scavenging system, two genes encoding SOD and one gene encoding CAT were up-regulated, and among 16 genes encoding POD, 13 were up-regulated, and 3 were down-regulated (Table S3). However, in protein activity level, SOD, CAT, and POD enzymes were all enhanced (Figure S1). The results demonstrate that redox homeostasis is a complicated and coordinated process, in which many genes are up- or down-regulated and the activities of their encoded proteins are also influenced. Taken together, our sequencing results confirmed that the enhanced performance of the H_2_-exposed PEMs could be attributed to the activation of antioxidant defense signaling, and thus the regulation of redox homeostasis. Additionally, six genes encoding AP2-like ethylene-responsive transcription factors BABY BOOM (BBM) and WUSCHEL(WUS)-related homeobox proteins, which are critical regulators for somatic embryogenesis [33,34,35], and 30 genes encoding endonucleases, exonuclease, cysteine proteases, aspartic proteases and metacaspases, which have been described to be involved in programmed cell death (PCD) [36,37,38,39], exhibited remarkably differential expression (Tables S11 and S12). The large number of DEGs encoding cell cycle regulators, PCD-related proteins, and BBM and WUS-related transcription factors that were identified suggested that they may also play a crucial role during this process.

Maximova et al. [40] found relatively stronger expression of genes related to stress responses during somatic embryogenesis than in zygotic embryogenesis of *Theobroma cacao* L., which may be caused by the in vitro culture environment. Consistent with the results, in our study many stress related genes were highly expressed in larch PEMs and showed differential expression after H_2_-treatment (Tables S1, S2, S6 and S7). The results suggested that these genes also acted a significant role in H_2_-exposed PEMs.

Hydrogen gas has been shown to act as a bioactive molecule in ameliorating oxidative injury in animals and in enhancing plant tolerance to oxidative stress by modulating the heme oxygenase-1 system [8,9,13]. The transcripts TR3453|c0_g1 and TR19727|c0_g2, which were annotated as peroxygenases and enriched in heme oxygenase (decyclizing) activity (GO: 0004392), were highly up-regulated at 12 h after hydrogen treatment (Tables S2 and S6). TR15756|c0_g1, annotated as heme oxygenase 1, was significantly up-regulated at 48 h after hydrogen treatment (Table S2). These results suggest that heme oxygenase 1 signaling systems may play a critical role in the response of larch PEMs to H_2_ exposure.

Owing to the fact that external molecular hydrogen is inherently an antioxidant counteracting oxidative stress, we thus speculate that reducing stress by applying ethylene, vitamin C, or a redox modulator such as, cysteine, or glutathione during somatic embryogenesis could result in similar biological effects and molecular responses as H_2_. It is interesting, but the hypothesis needs to be further tested by future research. Plant miRNAs are important regulators in developmental processes and abiotic stress responses [41]. In particular, miR398 and miR408 have been reported to be involved in plant response to oxidative stress and regulating ROS balance [42,43]. Nevertheless, little is known about miRNA profiles in response to H_2_ exposure, and their roles during this process remain unclear. The miRNA-seq analysis detected 96 miRNAs that were differentially expressed at five time points after H_2_ treatment, and 4399 target genes were predicted for 285 of the miRNAs (Tables S9 and S10). Among them, larch miR398 and miR408 and their target genes were differentially expressed at 12 h after H_2_ exposure, and miR5293 and its target gene TR20601|c1_g2, annotated as cytochrome c oxidase, which is involved in an ROS-producing process [44], were differentially expressed at 48 h after H_2_ exposure (Tables S2, S9 and S10). Two novel miRNAs, MA_3761110_43618 and MA_10429804_1042, and their negatively regulated targets genes TR16834|c0_g1 and TR13339|c0_g3, respectively, which may participate in chromosome structural maintenance and condensation during the cell cycle process, were differentially expressed at 48 h after H_2_ exposure (Tables S2, S9 and S10). These results suggest that miRNA-target gene interactions play significant roles in the larch PEMs response to hydrogen treatment.

Collectively, the changes in mRNA and miRNA expression levels between the H_2_-treatment and control libraries together with the complex mRNA-miRNA interaction network reported in this study will help to elucidate the regulatory effect of exogenous H_2_ on the somatic embryogenesis process in *L. leptolepis*. However, whether hydrogen has similar effects on somatic embryogenesis in other plant species and the corresponding molecular mechanism need to be further explored. Moreover, our study provides comprehensive mRNA and miRNA expression profiles during somatic embryogenesis and a resource for further comparative transcriptomics, and functional genomics research, particularly for network dissection in the regulation of oxidative stress responses in plants.

## 4. Experimental Section

### 4.1. Plant Materials and Growth Conditions

Callus tissues were generated on induction medium from immature zygotic embryos of *Larix leptolepis* Gordon, which were collected from the Da Gu Jia National Forest *Larix* Eugenic Species Base (Liaoning, China) [24]. The embryogenic calli were subcultured on culture medium to proliferate and produce PEMs [24]. To obtain SEs, the resultant PEMs was transferred directly to maturation medium [24].

### 4.2. Hydrogen Treatment

Purified 99.99% (*v*/*v*) H_2_ gas generated from a hydrogen gas generator (SHC-300, Saikesaisi Hydrogen Energy Co., Ltd., Jinan, China) was bubbled into a conical flask containing 1 L sterile culture or maturation media at a rate of 150 mL·min^−1^ for 1 h immediately after autoclave sterilization. The H_2_ concentration in the freshly prepared hydrogen-treated media was 0.4 mM, as determined by the method of Seo et al. [45]. Subsequently, every 40 mL culture or maturation media were added on a Petri dish at about 55 °C. The PEMs were then transferred to hydrogen-treated (hydrogen-rich) or non-hydrogen-treated (control) solid culture or maturation media under static cultivation in a dark environment at 25 ± 2 °C.

### 4.3. Measurement of ROS Levels and Antioxidant Enzyme Activity

PEMs were incubated in 10 µM H_2_DCFDA (a fluorescent probe), vacuum-infiltrated for 20 min, washed with double distilled water and observed [46] under a fluorescence microscope (BX51, Olympus, Tokyo, Japan) equipped with a camera (DP73, Olympus). The SOD activity was determined by nitro-blue tetrazolium colorimetry [47]. The catalase assay was performed following Aebi [48]. The peroxidase activity was measured as described previously [49].

### 4.4. Microscopic Observation of PEMs and SEs

PEMs, cultured on control and hydrogen-rich culture media for 36 days, were stained using acetic acid-carmine and Evans blue, and observed and photographed using a stereo microscope (AXIOIMAGER A1, Carl Zeiss, Oberkochen, Germany) equipped with a camera (AxioCamMRc5, Carl Zeiss, Oberkochen, Germany) [50].

After the PEMs were transferred to control and hydrogen-rich maturation media and cultured for 42 days, the resultant SEs were recorded, observed, and photographed under a microscope (M205FA, Leica Microsystems, Wetzlar, Germany) equipped with a camera (DFC425C, Leica Microsystems, Wetzlar, Germany).

### 4.5. Library Preparation and Sequencing

PEMs, sub-cultured on hydrogen-rich and control media, were collected after different incubation times (0 days, 12 h, 48 h, 7 days, 21 days, 36 days) and stored in liquid nitrogen. Total RNA was extracted with RNAiso Plus and RNAiso-mate for Plant Tissue kits (Takara, Dalian, China). Sample quality was measured using a 2100 Bioanalyzer (Agilent Technologies, Palo Alto, CA, USA), and RNAs with a RNA integrity number of 9 or above were used (Table S1). Two biological replicates were included for each sample. A total of 22 mRNA and 22 small RNA libraries were prepared according to the Illumina RNA and miRNA sequencing protocols, and sequenced on the NextSeq500 and HiSeq2500 platforms (Illumina, San Diego, CA, USA), respectively, at the University of California, Los Angeles (UCLA) Center for Pathology Research Services (Los Angeles, CA, USA).

### 4.6. De Novo Assembly and Statistical Analyses of mRNA-Seq Data

The reads from all the samples were combined and BBNorm (https://sourceforge.net/projects/bbmap/) was used for digital normalization and error correction. Trinity [51] was used for the whole transcriptome de novo assembly. Trinotate (http://trinotate.github.io) and the KEGG Automatic Annotation Server (KASS) (http://www.genome.jp/kaas-bin/kaas_main?mode=est_b) were used for comprehensive annotation of the assembled transcripts, including protein domain identification (HMMER/PFAM), protein signal prediction (SignalP/tmHMM), and comparison to currently curated annotation databases (EMBL/Uniprot/eggNOG/GO/KEGG Pathways databases). The reads in each sample were mapped to the assembled transcripts using Bowtie2 [52] and the gene expression levels were estimated usingRNA-Seq by Expectation Maximization (RSEM) [53]. TPM (transcripts per million) values were used to normalize the gene expression in each library. Differentially expressed genes (DEGs) were identified using the DeSeq program [54]. Genes showing more than two-fold changes with *p* < 0.05 were considered differentially expressed. Hierarchical clustering was performed and heat maps were created using the R platform (http://www.rproject.org/). A Venn Diagram was constructed using an online tool (http://bioinformatics.psb.ugent.be/webtools/Venn/). GOseq [55] and KOBAS [56] were used to perform the gene ontology (GO) and Kyoto Encyclopedia of Genes and Genomes (KEGG) enrichment analysis for the DEGs.

### 4.7. Real-Time Quantitative PCR (qPCR) Validation of DEGs

The total RNA used for RNA-seq was reverse transcribed to cDNAs using SuperScript III RT (Invitrogen, Carlsbad, CA, USA). qPCRs were performed on a ViiATM 7 Real-Time PCR System (Applied Biosystems, Foster City, CA, USA) with SYBR green (Invitrogen) following the manufacturer’s instructions. Melting curves were analyzed at the dissociation step to determine the amplification specificity. *LaEF-1α* (*Larix leptolepis* translation elongation factor-1 alpha 1, GenBank accession No. JX157845) [24,25,26,27,28], a housekeeping gene, was selected as the internal control for expression normalization, and the relative expression levels were determined using the 2^−ΔΔ*C*t^ method [57]. The primer sequences are listed in Table S5.

### 4.8. Statistical Analysis of miRNA-Seq Data

Cutadapt (http://code.google.com/p/cutadapt/) was used to remove the adaptors from the raw reads, which were then mapped to the Norway spruce genome [58]. MiRDeep2 [59] was used to identify the miRNAs based on their secondary structures from the mapped reads in the 22 libraries. The Quantifier module in MiRDeep2 was used to calculate the expression levels of the identified miRNAs in each sample. Differentially expressed miRNAs were identified using edgeR [60]. miRNAs showing more than 1.5-fold changes with *p* < 0.05 were considered differentially expressed. To identify conserved and novel miRNAs, the miRNA sequences were compared with known miRNAs in miRBase (release 21; http://www.mirbase.org) and larch miRNAs [26,27,28], allowing for 0–2 nucleotide mismatches.

### 4.9. mRNA-miRNA Integration Analysis

PsRobot was used to identify the mRNA targets of each miRNA [61]. To better understand the mRNA-miRNA interactions, a custom R script was used to perform an integrative analysis of the miRNA and miRNA data at each time point [62]. Cytoscape (v3.0.1; www.cytoscape.org/) was used to create a potential network of differentially expressed miRNAs and their corresponding target genes.

### 4.10. Availability of Supporting Data

The mRNA and miRNA transcriptomes data in this study have been deposited at the NCBI BioProject website (http://www.ncbi.nlm.nih.gov/bioproject) under accession number PRJNA328201.

## Supplementary Materials

Supplementary materials can be found at www.mdpi.com/1422-0067/17/11/1951/s1.

## Abbreviations

BBM	BABY BOOM
CAT	Catalase
DEG	Differentially Expressed Gene
FC	Fold Change
GO	Gene Ontology
KEGG	Kyoto Encyclopedia of Genes and Genomes
miRNA	MicroRNA
PCD	Programmed Cell Death
PEM	Pro-embryogenic Mass
POD	Peroxidase
qPCR	Real-time Quantitative PCR
ROS	Reactive Oxygen species
SE	Somatic Embryo
SOD	Superoxide Dismutase
TPM	Transcripts per Million
WUS	WUSCHEL
