# Supplementary material for: Transcriptome Analysis of mRNA and miRNA in Somatic Embryos of Larix leptolepis Subjected to Hydrogen Treatment
Source: Int J Mol Sci. 2016 Nov 22;17(11):1951. doi: 10.3390/ijms17111951 (PMC5133945; doi:10.3390/ijms17111951)
Supplement: Supplementary file 1 [file ESM_ijms-155406-supple-file S1.pdf › ijms-155406-Supplementary Materials/ESM_ijms-155406-supple-file S1.pdf]

Provisional ID	:	MA_1007189_31962
Score total	:	1.7
Score for star read(s)	:	-1.3
Score for read counts	:	0
Score for mfe	:	1.4
Score for randfold	:	1.6
Score for cons. seed	:	
Total read count	:	32
Mature read count	:	32
Loop read count	:	0
Star read count	:	0

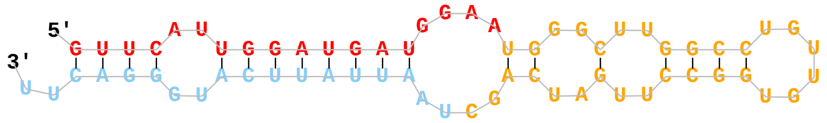

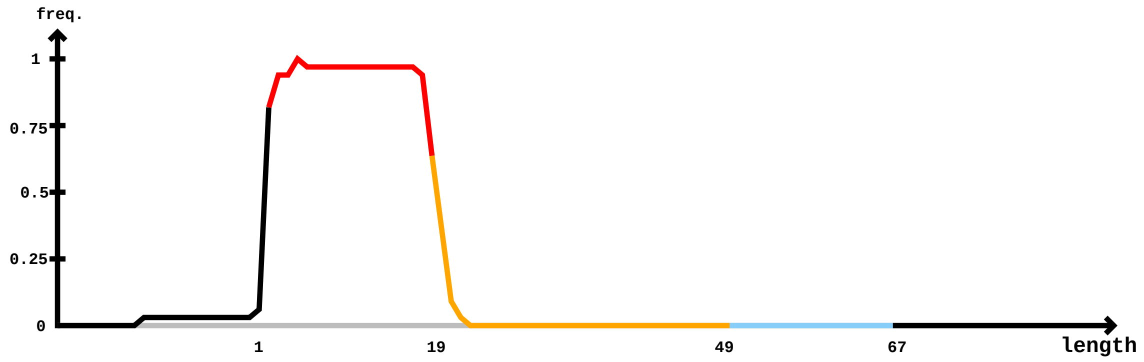

Mature Star

```
5'- aauggucuuggauggucgugguucauuggaugauggaaugggcuuggccuguuguggccuugaucagcuaauuauucaugggacuugacuuucuuugauggacacaaa   -3'    exp
    ..((((((.(((.(((((.(((((..((((((((....((..(..((((......))))..)..))....))))))))..)))))))))).))).....)))).))..    reads   mm   sample
    .......Cuggauggucgugguuc....................................................................................    1       1    seq
    ..................gguucauuggaugaugg.........................................................................    1       0    seq
    ....................guucauuggaugaugga.......................................................................    10      0    seq
    ....................guucauuggaugauggaG......................................................................    1       1    seq
    ....................guucauCggaugauggaa......................................................................    1       1    seq
    ....................guucauuggaugauggaa......................................................................    12      0    seq
    ....................guucauuggaugauggaaC.....................................................................    1       1    seq
    .....................uucauuggaugauggaa......................................................................    3       0    seq
    .....................uucauuggaugauggaaGg....................................................................    1       1    seq
    .......................cauuggaugauggaaGg....................................................................    1       1    seq
    .......................cauuggaugauggaaugg...................................................................    1       0    seq
```

Provisional ID	:	MA_10074432_34554
Score total	:	1.8
Score for star read(s)	:	-1.3
Score for read counts	:	0
Score for mfe	:	1.5
Score for randfold	:	1.6
Score for cons. seed	:	
Total read count	:	459
Mature read count	:	459
Loop read count	:	0
Star read count	:	0

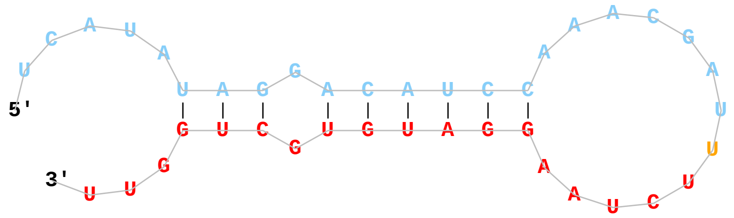

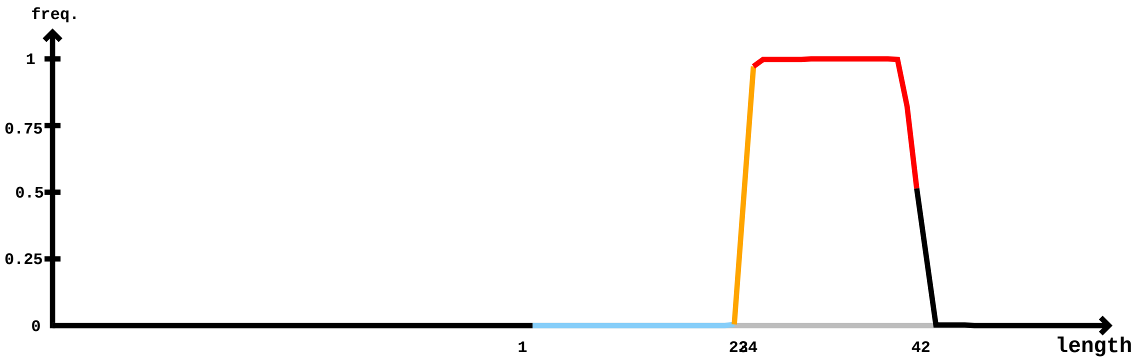

Star Mature

```
5'- uggaaacaaguugggcaucaacuuuuaucaggcucgcuugauagggaaucauauaggacauccaaacgauuucuaaggaugugcugguucuguuggugcucugucaaa -3'  exp
    ((....))....((((((((((..((((((((....)))))))).((((((....(.((((((.............)))))).))))))).)))))))))).......  reads  mm  sample
    .....................................................................uuucuaaggaugugcug......................  1  0  seq
    .....................................................................uuucuaaggaugugcugg.....................  1  0  seq
    ......................................................................uucuaaggaugugUugg.....................  1  1  seq
    ......................................................................uucuaaggaugugcugg.....................  73  0  seq
    ......................................................................uucuaaggGugugcugg.....................  1  1  seq
    ......................................................................uucuaaggaugugcugU.....................  1  1  seq
    ......................................................................uucuaaggaugugcugA.....................  4  1  seq
    ......................................................................uucuaaggaugugcuggu....................  72  0  seq
    ......................................................................uucuaaggaugugcuggC....................  7  1  seq
    ......................................................................uuUuaaggaugugcuggu....................  1  1  seq
    ......................................................................Nucuaaggaugugcuggu....................  1  1  seq
    ......................................................................uucuaaggaugugcuggA....................  1  1  seq
    ......................................................................uucuaaggaugugcuAgu....................  1  1  seq
    ......................................................................Aucuaaggaugugcuggu....................  1  1  seq
    ......................................................................uucuaaggaugugcugguA...................  101  1  seq
    ......................................................................uucuaaggaugugcugguG...................  2  1  seq
    .......................................................................ucuaaggaugugcuggA....................  4  1  seq
    .......................................................................ucuaaggaugugcuggG....................  2  1  seq
    .......................................................................ucuaaggaugugcuggC....................  10  1  seq
    .......................................................................ucuaaggaugugcuggu....................  41  0  seq
    .......................................................................ucuaaggaugugcugguG...................  1  1  seq
    .......................................................................ucuaaggaugugcugguA...................  119  1  seq
    ........................................................................cuaaggaugugcugguA...................  12  1  seq
    .............................................................................gaugugcuUguucuguu..............  1  1  seq
```

Provisional ID	:	MA_100832_7971
Score total	:	0.1
Score for star read(s)	:	-1.3
Score for read counts	:	0
Score for mfe	:	-0.1
Score for randfold	:	1.6
Score for cons. seed	:	
Total read count	:	104
Mature read count	:	104
Loop read count	:	0
Star read count	:	0

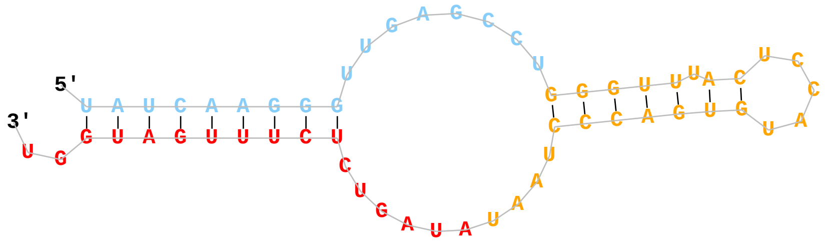

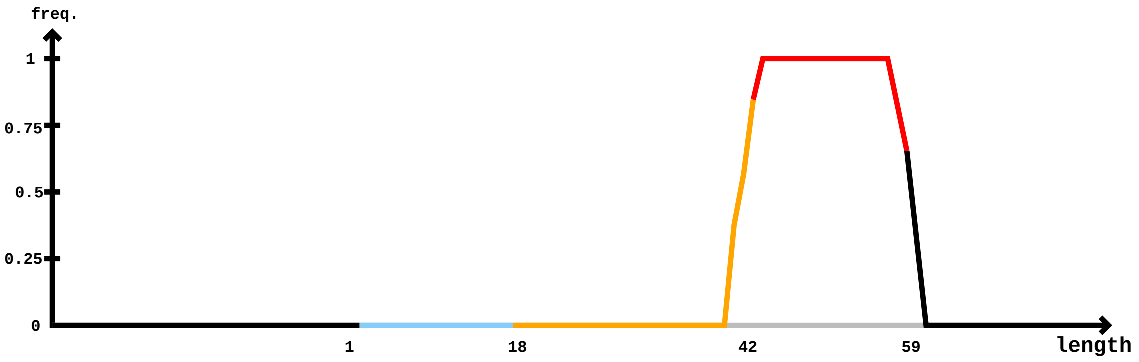

Star Mature

5'- uggaaaaucucaccuugaauacuucgucacuaucaaggguugagccuggguuuacuccaugugacccuaauauagucucuuugaugguuacacaguauucucugaacc -3'	exp		
.........(((....(((((((..((.(((((((((((........(((((.((.....)))))))..........))))))))))).))..)))))))..)))...	reads	mm	sample
....Uuauagucucuuugaug......................	17	1	seq
....Cuauagucucuuugaug......................	1	1	seq
....Uuauagucucuuugaugg.....................	13	1	seq
....Uuauagucucuuugauggu....................	7	1	seq
....Guauagucucuuugauggu....................	1	1	seq
....uauagucucuuugaugg.....................	4	0	seq
....uauagucucuuugaugA.....................	1	1	seq
....uauagucucuuugauggC....................	3	1	seq
....uauaguAucuuugauggu....................	1	1	seq
....uauagucucuuugauggu....................	8	0	seq
....uauagucucuuugauggG....................	1	1	seq
....uauagucucuuugaugguA...................	2	1	seq
....auagucucuuugauggC....................	3	1	seq
....auagucucuuugauggu....................	18	0	seq
....auaguGucuuugauggu....................	2	1	seq
....auagucucuuugauggG....................	1	1	seq
....auagucucuuugaugguA...................	4	1	seq
....auagucucuuugaugguG...................	1	1	seq
....uagucucuuugaugguA...................	15	1	seq
....uagucucuuugaugguG...................	1	1	seq

Provisional ID	:	MA_100926_7857
Score total	:	1355.9
Score for star read(s)	:	3.9
Score for read counts	:	1348.1
Score for mfe	:	2.3
Score for randfold	:	1.6
Score for cons. seed	:	
Total read count	:	2656
Mature read count	:	2650
Loop read count	:	0
Star read count	:	6

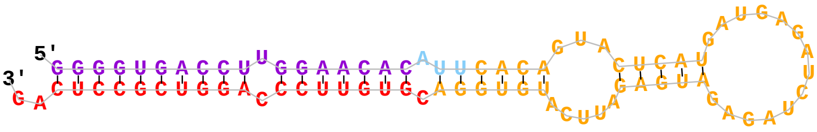

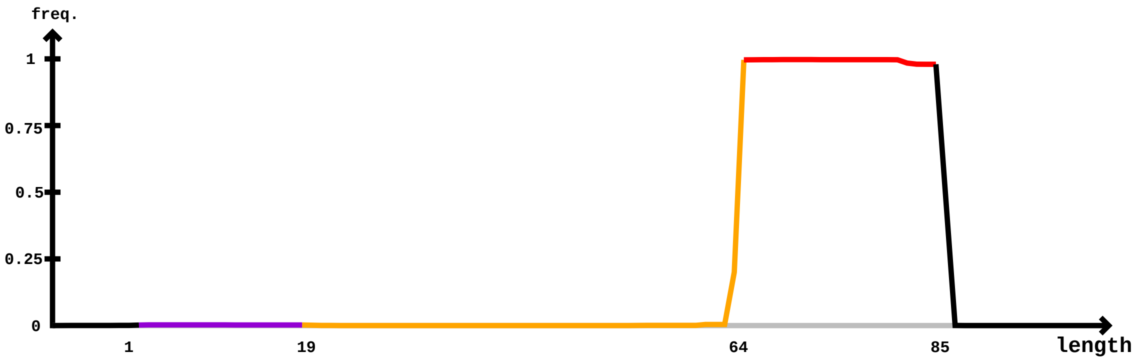

Star **Mature**

```
5'- uucgacuggggugaccuuggaacacauucacaguacucaugaugagaucuagagaugagauucauguggacguguucccaggucgccucagucgaaaccuccucugcu   -3'   obs
    uucgacuggggugaccuuggaacacauucacaguacucaugaugagaucuagagaugagauucauguggacguguucccaggucgccucagucgaaaccuccucugcu         exp
    (((((((((((((((((.(((((((.((((((...(((((.............))))).....)))))).))))))).))))))))))))))))).............   reads   mm   sample
    uucgacuggggugaccA...........................................................................................   1   1   seq
    .....cuggggugaGcuuggaacaca..................................................................................   1   1   seq
    .......ggggugaGcuuggaacac...................................................................................   2   1   seq
    .......ggggugaGcuuggaacacauu................................................................................   1   1   seq
    ........gggugaGcuuggaacaca..................................................................................   2   1   seq
    ...........................................................auuUauguggacguguucc..............................   1   1   seq
    ............................................................uuUauguggacguguucccaggucgc......................   1   1   seq
    ..................................................................uggacguguucccaggucgcc.....................   6   0   seq
    ..................................................................uggacguguucccaggucgcU.....................   1   1   seq
    ..................................................................uggacguguucccaggucgccu....................   2   0   seq
    .....................................................................acguguucccaggucgcc.....................   5   0   seq
    .....................................................................acguguucccaggucgccA....................   1   1   seq
    .....................................................................acguguucccaggucgccAcag.................   1   1   seq
    .....................................................................acguguucccaggucgccucag.................   1   0   seq
    .....................................................................acguguucccaggucgccCcag.................   509   1   seq
    .....................................................................acguguucccaggucgccCcagu................   6   1   seq
    ......................................................................cguguucccaggucgcc.....................   17   0   seq
    ......................................................................cguguucccaggucgcA.....................   2   1   seq
    ......................................................................cguguucccaggucgcU.....................   2   1   seq
    ......................................................................cguguucccaggucgcAu....................   1   1   seq
    ......................................................................cguguucccaggucgccu....................   4   0   seq
    ......................................................................cguguucccaggucgccA....................   3   1   seq
    ......................................................................cguguucccaggucgccAc...................   1   1   seq
    ......................................................................cguguucccaggucgccCcag.................   2066   1   seq
    ......................................................................cguguucccaggucgccucag.................   7   0   seq
    ......................................................................cguguucccaggucgccAcag.................   2   1   seq
    ......................................................................cgugCucccaggucgccucag.................   1   1   seq
    ......................................................................cguguucccaggucgccGcag.................   1   1   seq
    ......................................................................cguguucccaggucgccCcagu................   16   1   seq
    ......................................................................cguguucccaggucgccCcaguc...............   1   1   seq
    .......................................................................guguucccaggucgccCcag.................   1   1   seq
    ........................................................................uguucccaggucgccCcagu................   1   1   seq
    ..........................................................................uucccaggucgccCcag.................   1   1   seq
```

Star Mature

uucgacuggggugaccuuggaacacauucacaguacucaugaugagaucuagagaugagauucaugugacguguucccaggucgccucagucgaaaccuccucugcu

Provisional ID	:	MA_10099790_39948
Score total	:	1.5
Score for star read(s)	:	-1.3
Score for read counts	:	0
Score for mfe	:	1.2
Score for randfold	:	1.6
Score for cons. seed	:	
Total read count	:	21
Mature read count	:	21
Loop read count	:	0
Star read count	:	0

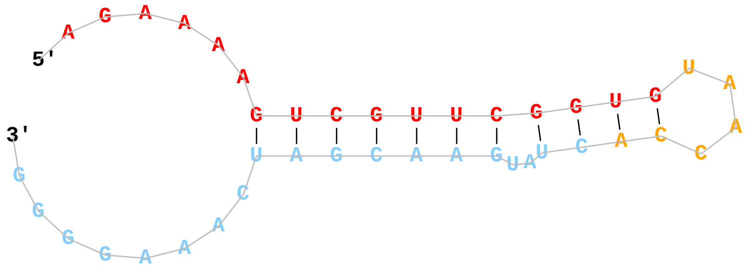

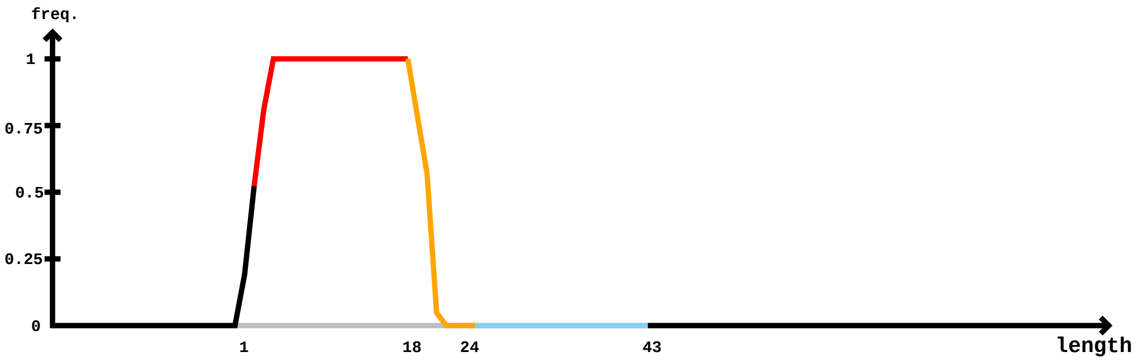

Mature **Star**

```
5'- acaaacacuagcacacaggagaaaagucguucgguguaaccacuaugaacgaucaaaggggaagcauucaauaugcgcgugcgaaggugguggggauuauucaccacca   -3'   exp
    ..........((((...........((((((((((....))))..)))))))..........((((.....))))..))))...(((((((((....))))))))).   reads   mm   sample
    .................Aagaaaagucguucggug........................................................................   1       1    seq
    .................Aagaaaagucguucggugu.......................................................................   1       1    seq
    .................Aagaaaagucguucggugua......................................................................   2       1    seq
    ..................agaaaagucguucggug........................................................................   6       0    seq
    ..................agaaaagucguucggugua......................................................................   1       0    seq
    ...................gaaaagucguucggugC.......................................................................   1       1    seq
    ...................gaaaagucguucggugua......................................................................   5       0    seq
    ....................aaaagucguucggugua......................................................................   3       0    seq
    ....................aaaagucguucgguguaU.....................................................................   1       1    seq
```

Provisional ID	:	MA_10114_10681
Score total	:	1.1
Score for star read(s)	:	-1.3
Score for read counts	:	0
Score for mfe	:	0.8
Score for randfold	:	1.6
Score for cons. seed	:	
Total read count	:	97
Mature read count	:	97
Loop read count	:	0
Star read count	:	0

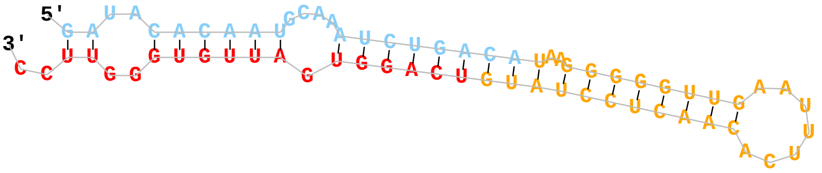

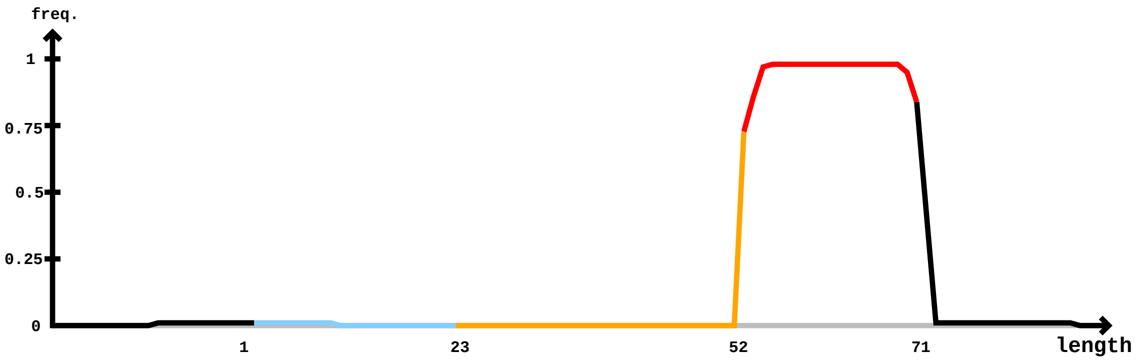


```
                         Star                                          Mature
5'- aacuacgguuuagcucaaugauacacaaugcaaaucugacauaaggggguugaauuucacaacuccuaugucaggugauuguggguuccauggucauuugaaauauag   -3'    exp
    (((....)))....((((((((.((((((....(((((((((..((((((((.......)))))))))))))))))).)))))).........)))).)))).......   reads   mm   sample
    .........uGagcucaaugauacacaa................................................................................   1       1    seq
    ......................................................................ucaggugauuguggguG.....................   1       1    seq
    ......................................................................ucaggugauuguggguu.....................   1       0    seq
    ......................................................................ucaggugauuguggguC.....................   1       1    seq
    ......................................................................ucaggGgauuguggguuc....................   1       1    seq
    ......................................................................ucaggAgauuguggguuc....................   1       1    seq
    ......................................................................ucaggugauuguggguuc....................   8       0    seq
    ......................................................................ucaggugauuguggguCc....................   1       1    seq
    ......................................................................ucaggCgauuguggguucc...................   58      1    seq
    .......................................................................caggugauuguggguuc....................   1       0    seq
    .......................................................................caggCgauuguggguucc...................   12      1    seq
    ........................................................................aggCgauuguggguucc...................   11      1    seq
    .........................................................................ggCgauuguggguucca..................   1       1    seq
    ........................................................................................cauggucauuGgaaaua...   1       1    seq
```

Provisional ID	:	MA_10148035_40528
Score total	:	4339.7
Score for star read(s)	:	3.9
Score for read counts	:	4361.2
Score for mfe	:	-26.9
Score for randfold	:	1.6
Score for cons. seed	:	
Total read count	:	8566
Mature read count	:	7723
Loop read count	:	0
Star read count	:	843

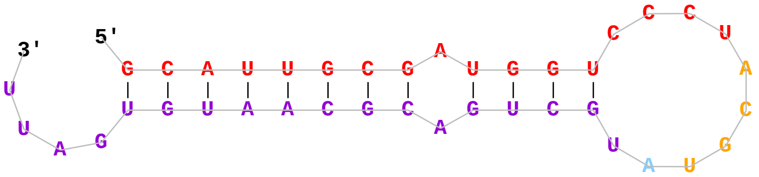

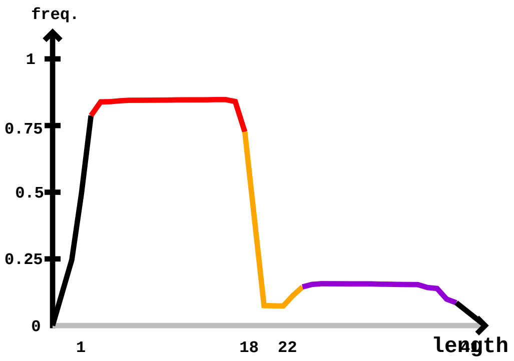

Mature **Star**

```
5'- aagcauugcgauggucccuacguaugcugacgcaaugugauuu  -3'   obs
    aagcauugcgauggucccuacguaugcugacgcaaugugauuu         exp
    ..((((((((.((((..........)))).)))))))).....      reads     mm        sample
    aagcauugcgaugguUc..........................      1         1         seq
    aagcauugcgauggucA..........................      2         1         seq
    aagcauCgcgauggucc..........................      1         1         seq
    aagcauugcgauggucc..........................      61        0         seq
    aagcauugGgaugguccc.........................      1         1         seq
    aagcauugcgaugguccUc........................      2         1         seq
    aagcauCgcgaugguccc.........................      3         1         seq
    aagcauugcgaugguUcc.........................      1         1         seq
    aagcauugcgaugguccc.........................      449       0         seq
    aagcauugcgauAguccc.........................      1         1         seq
    aagcauugcgauggGccc.........................      1         1         seq
    Cagcauugcgaugguccc.........................      1         1         seq
    aagcauugcgaugguccGc........................      1         1         seq
    Gagcauugcgaugguccc.........................      2         1         seq
    aagcauugcgauggCccc.........................      3         1         seq
    aagcauugcgauggucccG........................      5         1         seq
    aagcauugcgaugguAcc.........................      1         1         seq
    aagcauugcgGugguccc.........................      2         1         seq
    aagcaCugcgaugguccc.........................      1         1         seq
    Uagcauugcgaugguccc.........................      1         1         seq
    aagcGuugcgaugguccc.........................      1         1         seq
    aagcauugcgGuggucccu........................      8         1         seq
    aGgcauugcgauggucccu........................      3         1         seq
    aUgcauugcgauggucccu........................      2         1         seq
    aagcGuugcgauggucccu........................      2         1         seq
    Nagcauugcgauggucccu........................      1         1         seq
    Uagcauugcgauggucccu........................      1         1         seq
    aagcaCugcgauggucccu........................      2         1         seq
    aagcauugcgaugguccccG.......................      53        1         seq
    Cagcauugcgauggucccu........................      2         1         seq
    aagcauugcgaugguccccC.......................      221       1         seq
    aagcauugcgaugguccccA.......................      64        1         seq
    aagcauugcgauggucAcu........................      2         1         seq
```

Mature **Star**

aagcauugcgauggucccuacguaugcugacgcaaugugauuu

aagcauugcgaugAucccu........................	1	1	seq
aagcauugcgauggucccu........................	1141	0	seq
aagcauugcgaCggucccu........................	3	1	seq
aagcauugcgauggGcccu........................	1	1	seq
aagcauugcgauggucUcu........................	3	1	seq
aagcauugcgauggCcccu........................	3	1	seq
aagcauugcgaugguUccu........................	1	1	seq
aagcauugcgauAgucccu........................	1	1	seq
Gagcauugcgauggucccu........................	3	1	seq
Nagcauugcgauggucccua.......................	1	1	seq
aagUauugcgauggucccua.......................	1	1	seq
aagcauCgcgauggucccua.......................	1	1	seq
aagcauugcgauggucccAa.......................	1	1	seq
aagcauugcgauggucccCa.......................	7	1	seq
aagcauugcgauggucccua.......................	183	0	seq
aagcaCugcgauggucccua.......................	2	1	seq
Cagcauugcgauggucccua.......................	1	1	seq
aagcauugcgGuggucccua.......................	1	1	seq
aagcauugcgaugguccGua.......................	1	1	seq
aagcauugcgaugAucccua.......................	1	1	seq
aagcauugcgauggucccuCc......................	1	1	seq
aagcauugcgauggucccuGcgua...................	1	1	seq
.Ugcauugcgaugguccc.........................	1	1	seq
.agcauugcgauggCccc.........................	2	1	seq
.agcauugcgauggucUc.........................	1	1	seq
.Ggcauugcgaugguccc.........................	1	1	seq
.agcauugcgauggGccc.........................	2	1	seq
.agcauugcgaugguccc.........................	545	0	seq
.agcauugcgaugguccG.........................	8	1	seq
.agcauugcgGugguccc.........................	3	1	seq
.agcauugcgaCgguccc.........................	1	1	seq
.agcaCugcgaugguccc.........................	3	1	seq
.agcauCgcgaugguccc.........................	1	1	seq
.agcauugcgUuggucccu........................	1	1	seq
.agcauugcgCuggucccu........................	1	1	seq
.agGauugcgauggucccu........................	1	1	seq
.agcauugcgauggCcccu........................	1	1	seq
.agcauugcgaCggucccu........................	3	1	seq
.agcaCugcgauggucccu........................	2	1	seq
.agcauugcgaugAucccu........................	2	1	seq
.agcauugcgauggucccA........................	64	1	seq
.agcauugcgauggucccG........................	55	1	seq
.agcGuugcgauggucccu........................	3	1	seq
.agcauugcgGuggucccu........................	5	1	seq
.agcauugcgauggGcccu........................	2	1	seq
.agcauugcgauggucccu........................	1124	0	seq
.agcauugcgauggucccC........................	217	1	seq
.agcauCgcgauggucccu........................	1	1	seq
.agcauugGgauggucccu........................	2	1	seq
.agcauugcgaGggucccu........................	1	1	seq
.agcauugcgaugguGccu........................	1	1	seq
.agcauGgcgauggucccu........................	1	1	seq
.agcauugcgauggucGcu........................	1	1	seq
.agcauugcgauggucccCa.......................	10	1	seq
.agcauugcgauggucccua.......................	189	0	seq
.agcauugcgauggCcccua.......................	1	1	seq
.agcauugcgaugguccUua.......................	1	1	seq
.agcauugcgauAgucccua.......................	1	1	seq
.Ggcauugcgauggucccua.......................	1	1	seq
.agcGuugcgauggucccua.......................	1	1	seq
.agcauCgcgauggucccua.......................	1	1	seq
.agcauugcgauggucccuacg.....................	1	0	seq
.agcauugcgauggucccuGcgu....................	3	1	seq
.agcauugcgauggucccuGcgua...................	1	1	seq
.agcauugcgauggucccuGcguaugcugacg...........	1	1	seq
..gcUuugcgauggucccu........................	1	1	seq
..Ccauugcgauggucccu........................	1	1	seq
..gcauugcgauggucAcu........................	3	1	seq
..Ncauugcgauggucccu........................	4	1	seq
..gcauugcgauggucccu........................	1771	0	seq

Mature **Star**

```
aagcauugcgauggucccuacguaugcugacgcaaugugauuu
..gcauAgcgauggucccu........................   1     1     seq
..gGauugcgauggucccu........................   2     1     seq
..gcGuugcgauggucccu........................   6     1     seq
..gcauugcgaAggucccu........................   2     1     seq
..gcauugcgUuggucccu........................   3     1     seq
..gcauugcgaugguAccu........................   3     1     seq
..gcaCugcgauggucccu........................   1     1     seq
..gcauugcgGuggucccu........................   8     1     seq
..gcauugcgaugguUccu........................   1     1     seq
..gcauCgcgauggucccu........................   3     1     seq
..gcauugcgaugguccC.........................   331   1     seq
..gcauugcgaugAucccu........................   3     1     seq
..gcauugcgauggucUcu........................   3     1     seq
..gcauugcgauAgucccu........................   1     1     seq
..gcauugcgaCggucccu........................   3     1     seq
..gcauGgcgauggucccu........................   1     1     seq
..gcauugcgauUgucccu........................   1     1     seq
..gcauugcgaugguccA.........................   75    1     seq
..gcauugcgauggGcccu........................   2     1     seq
..gcauugAgauggucccu........................   1     1     seq
..gcauugcgaugguccG.........................   82    1     seq
..gcauugcgauggCcccu........................   2     1     seq
..gcauugcgGuggucccua.......................   3     1     seq
..gcaCugcgauggucccua.......................   1     1     seq
..gcauugcgaugguccCa........................   17    1     seq
..gUauugcgauggucccua.......................   1     1     seq
..gcauugcgaugguccGa........................   4     1     seq
..gcauugcgauggucccua.......................   321   0     seq
..gcauugcgauggGcccua.......................   2     1     seq
..gcauCgcgauggucccua.......................   1     1     seq
..gcauugcgaugguccAa........................   1     1     seq
..gcauugcgaCggucccua.......................   1     1     seq
..Acauugcgauggucccua.......................   1     1     seq
..gcauugcgauggucccuCc......................   2     1     seq
..gcauugcgauggucccuacg.....................   2     0     seq
..gcauugcgauggucccuGcgu....................   13    1     seq
..gcauugcgauggucccuacgCa...................   1     1     seq
..gcauugcgauggucccuacguU...................   1     1     seq
..gcauugcgauggucccuGcguau..................   1     1     seq
..gcauugcgauggucccuGcguaug.................   1     1     seq
..gcauugcgauggucccuGcguaugcug..............   1     1     seq
...cauugcgaugguccAa........................   1     1     seq
...Nauugcgauggucccua.......................   1     1     seq
...cauugcgauggCcccua.......................   1     1     seq
...cauugcgaCggucccua.......................   1     1     seq
...cauugcgaugguccCa........................   21    1     seq
...cGuugcgauggucccua.......................   1     1     seq
...cauugcgaugguGccua.......................   1     1     seq
...cauugcgauggucUcua.......................   2     1     seq
...cauugcgauggucccua.......................   417   0     seq
...Uauugcgauggucccua.......................   1     1     seq
...cauugcgaugAucccua.......................   1     1     seq
...cauugcgaugguccGa........................   1     1     seq
...cauugcgauggucccuac......................   1     0     seq
...cauugcgauggucccuCc......................   5     1     seq
...cauugcgauggucccuUc......................   1     1     seq
...cauugcgauggucccuacg.....................   1     0     seq
...cauugcgauggucccuacA.....................   1     1     seq
...cauugcgauggucccuacU.....................   1     1     seq
...cauugcgauggucccuGcgu....................   10    1     seq
...cauugcgauggucccuGcguaugcugacgca.........   1     1     seq
....auugcgauggucccuCc......................   2     1     seq
....auugcgauggucccuac......................   1     0     seq
....auugcgauggucccuGcgu....................   2     1     seq
....auugcgauggucccuacguC...................   1     1     seq
....auugcgauggucccuGcguaugcugacgca.........   1     1     seq
.....uugcgauggucccuUcg.....................   1     1     seq
.....uugcgauggucccuacA.....................   1     1     seq
.....uugcgauggucccuGcgu....................   16    1     seq
.....uugcgauggucccuGcgua...................   2     1     seq
```

Mature **Star**

```
aagcauugcgaugguccc uacguaugcugacgcaaugugauuu
.....uugcgauggucccuGcguaugc................    2     1     seq
.....uugcgauggucccuGcguaugcu...............    1     1     seq
.....uugcgauggucccuGcguaugcug..............    1     1     seq
.....uugcgauggucccuGcguaugcugacgca.........    1     1     seq
.....uugcgauggucccuGcguaugcugacgcaa........    1     1     seq
.....uugcgauggucccuGcguaugcugacgcaaug......    3     1     seq
.....uugcgauggucccuGcguaugcugacgcaaugug....    1     1     seq
......ugcgauggucccuGcgu....................    13    1     seq
......ugcgauggucccuGcgua...................    1     1     seq
......ugcgauggucccuGcguaugcugacg...........    2     1     seq
......ugcgauggucccuGcguaugcugacgcaau.......    2     1     seq
.......gcgauggucccuGcguaugcugacgcaa........    1     1     seq
........cgauggucccuGcguau..................    1     1     seq
........cgauggucccuGcguaug.................    1     1     seq
.........gauggucccuGcguaugcugacg...........    2     1     seq
.........gauggucccuGcguaugcugacgcaau.......    1     1     seq
..........auggucccuGcguaugcugacgca.........    1     1     seq
..........auggucccuGcguaugcugacgcaaug......    1     1     seq
...........uggucccuGcguaugcugacg...........    1     1     seq
...........uggucccuGcguaugcugacgc..........    1     1     seq
...........uggucccuGcguaugcugacgca.........    1     1     seq
...........uggucccuGcguaugcugacgcaaug......    1     1     seq
...........uggucccuGcguaugcugacgcaaugug....    2     1     seq
............ggucccuGcguaugcugacgca.........    1     1     seq
.............gucccuGcguaugcugacg...........    1     1     seq
...............cccuGcguaugcugacg...........    3     1     seq
...............cccuGcguaugcugacgc..........    2     1     seq
...............cccuGcguaugcugacgca.........    1     1     seq
...............cccuGcguaugcugacgcaaugu.....    1     1     seq
.................cuGcguaugcugacgca.........    1     1     seq
..................uGcguaugcugacgcaaugug....    1     1     seq
...................Gcguaugcugacgcaaug......    2     1     seq
...................Gcguaugcugacgcaaugug....    9     1     seq
...................Gcguaugcugacgcaaugugauu.    1     1     seq
....................Aguaugcugacgcaaug......    1     1     seq
....................cguaugcugacgcaaug......    25    0     seq
....................cguaugcugacgcaauA......    1     1     seq
....................cgCaugcugacgcaaug......    5     1     seq
....................cgAaugcugacgcaaug......    59    1     seq
....................cNuaugcugacgcaaug......    1     1     seq
....................Nguaugcugacgcaaug......    1     1     seq
....................cgAaugcugacgcaaugu.....    20    1     seq
....................cguaugcugacgcaaugG.....    1     1     seq
....................cgCaugcugacgcaaugu.....    2     1     seq
....................cUuaugcugacgcaaugu.....    1     1     seq
....................cNuaugcugacgcaaugu.....    1     1     seq
....................cguaugcugacgcaaugu.....    3     0     seq
....................Nguaugcugacgcaaugu.....    2     1     seq
....................cguaugcugacgcaGugug....    2     1     seq
....................cguaugcugacgcaauguA....    5     1     seq
....................Nguaugcugacgcaaugug....    12    1     seq
....................cUuaugcugacgcaaugug....    2     1     seq
....................cgCaugcugacgcaaugug....    26    1     seq
....................cguaugcugacgcaaugug....    88    0     seq
....................cguaugcugaUgcaaugug....    1     1     seq
....................Uguaugcugacgcaaugug....    2     1     seq
....................cgAaugcugacgcaaugug....    201   1     seq
....................cguaugcugacgcGaugug....    1     1     seq
....................cguaugcugGcgcaaugug....    1     1     seq
....................cguaugcCgacgcaaugug....    1     1     seq
....................Gguaugcugacgcaaugug....    1     1     seq
....................cNuaugcugacgcaaugug....    5     1     seq
....................Aguaugcugacgcaaugug....    1     1     seq
....................cguaugcugacgcaaCgug....    1     1     seq
....................cgAaugcugacgcaauguga...    11    1     seq
....................Nguaugcugacgcaauguga...    2     1     seq
....................cgCaugcugacgcaauguga...    2     1     seq
....................cguaugcugacgcaauguga...    4     0     seq
....................cgAaugcugacgcaaugugau..    7     1     seq
....................cgCaugcugacgcaaugugau..    1     1     seq
```

Mature Star

```
aagcauugcgauggucccuacguaugcugacgcaaugugauuu
....................cguaugcugacgcaaugugau..    6      0      seq
....................cguaugcugacgcaaugugaG..    1      1      seq
....................Nguaugcugacgcaaugugauu.    1      1      seq
....................cgAaugcugacgcaaugugauu.    21     1      seq
....................cguaugcugacgcaaugugauu.    6      0      seq
....................cgCaugcugacgcaaugugauu.    2      1      seq
....................cguaugcugacgcaaugugauG.    1      1      seq
....................cguaugcugacgcaaugugauuu    1      0      seq
....................cgAaugcugacgcaaugugauuu    4      1      seq
.....................guaugcugacgcaaugC.....    1      1      seq
.....................guaugcugacgcaaugu.....    1      0      seq
.....................gAaugcugacgcaaugug....    2      1      seq
.....................Nuaugcugacgcaaugug....    1      1      seq
.....................guaugcugacgcaaugugauu.    1      0      seq
......................uCugcugacgcaaugug....    1      1      seq
......................Aaugcugacgcaaugug....    1      1      seq
......................Naugcugacgcaaugugauu.    1      1      seq
.......................augcugacgcaauguga...    98     0      seq
.......................augcugacgcaaugugU...    3      1      seq
.......................augcugacgcaaugGga...    1      1      seq
.......................augcCgacgcaauguga...    1      1      seq
.......................Gugcugacgcaaugugau..    2      1      seq
.......................augcugacgcaaugugaC..    14     1      seq
.......................augcugacgcaauAugau..    1      1      seq
.......................augcugacgcaaugugaG..    1      1      seq
.......................augcugacgcaaugugGu..    1      1      seq
.......................augcugacgcaaugugau..    83     0      seq
.......................auAcugacgcaaugugauu.    1      1      seq
.......................augcugacgcaaugugauG.    2      1      seq
.......................augcugacgcaaugugaGu.    1      1      seq
.......................augcugacgcaaugugaAu.    1      1      seq
.......................augcugacgcaaugugauC.    26     1      seq
.......................Cugcugacgcaaugugauu.    1      1      seq
.......................augcugacgcaauAugauu.    1      1      seq
.......................augcugacgcGaugugauu.    1      1      seq
.......................augcugacgcaaugugauu.    164    0      seq
.......................augcugacgcaaCgugauu.    1      1      seq
.......................augcuCacgcaaugugauu.    1      1      seq
.......................augcugacgcaaugugauuu    5      0      seq
.......................augcugacgcaaugugauuC    2      1      seq
........................Cgcugacgcaaugugau..    1      1      seq
........................ugcugacgcaaugugaC..    13     1      seq
........................ugcugacgcaaugugau..    71     0      seq
........................ugcugacgcaaugugaA..    1      1      seq
........................ugcugacgcaaugugUu..    1      1      seq
........................ugcugacgcaaugugauu.    171    0      seq
........................ugcugacgcaaugugGuu.    2      1      seq
........................ugcugacgcaUugugauu.    1      1      seq
........................ugcugacgcaaCgugauu.    1      1      seq
........................uAcugacgcaaugugauu.    2      1      seq
........................ugcugacgcGaugugauu.    1      1      seq
........................ugcugacgcaaugugaCu.    2      1      seq
........................ugcugacgcaGugugauu.    2      1      seq
........................ugGugacgcaaugugauu.    1      1      seq
........................ugcugacgcaaugugauC.    20     1      seq
........................ugcugacgcaaugugauuC    3      1      seq
........................ugcugacgcaaugugauuu    14     0      seq
.........................gcugacgcaaugugGuu.    1      1      seq
.........................gcugacgcaaugugauA.    1      1      seq
.........................gcugacgcaaugugCuu.    1      1      seq
.........................gcugacgcaaugugauu.    74     0      seq
.........................gcugacgcaaugugauC.    8      1      seq
.........................gcugacgcaaugugauG.    1      1      seq
.........................gcugacgcaaugCgauu.    1      1      seq
.........................gcugacgcaaugugauuu    2      0      seq
..........................cugacgcaaugugauuC    4      1      seq
..........................cugacgcaGugugauuu    1      1      seq
..........................cugacgcaaugugauuu    21     0      seq
```

Provisional ID	:	MA_10148535_42135
Score total	:	4866.1
Score for star read(s)	:	3.9
Score for read counts	:	4866.9
Score for mfe	:	-2.4
Score for randfold	:	-2.2
Score for cons. seed	:	
Total read count	:	9558
Mature read count	:	9548
Loop read count	:	0
Star read count	:	10

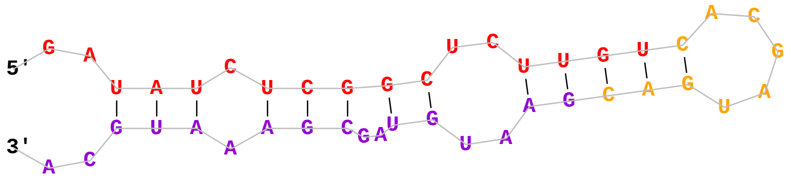

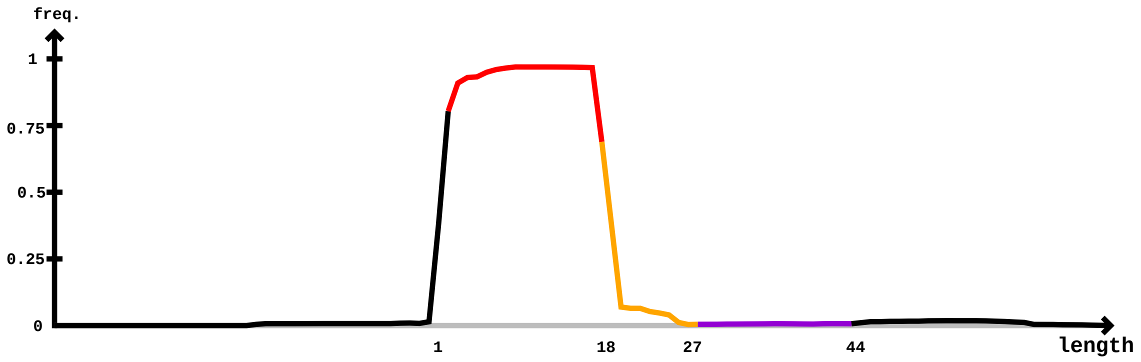


```
                                         Mature          Star
5'- ggguucgucaggaaugugaggaaaugacucucgacaauggauaucucggcucuugucacgaugacgaauguagcgaaaugcaauacuuagugugaauugaagaauccc   -3'   obs
    ggguucgucaggaaugugaggaaaugacucucgacaauggauaucucggcucuugucacgaugacgaauguagcgaaaugcaauacuuagugugaauugaagaauccc          exp
    ((((((.((((...(((((((.......)))).))).((.((.((.(.((..(((((.....)))))..)).).)).)).))..............)))).)))))).   reads   mm   sample
    ...................Agaaaugacucucgaca........................................................................   44      1    seq
    ...................Agaaaugacucucgacaa.......................................................................   2       1    seq
    ...................Agaaaugacucucgacaau......................................................................   1       1    seq
    ....................gaaaugacucucgacaa.......................................................................   11      0    seq
    ....................gaaaugacucucgCcaa.......................................................................   1       1    seq
    ....................gaaaugacucucCacaa.......................................................................   1       1    seq
    ....................gaaaugacucucgacCa.......................................................................   2       1    seq
    ....................gaaaugacucucgUcaau......................................................................   1       1    seq
    ....................gaaaugacucucgacaau......................................................................   4       0    seq
    ....................gaaaugacucucgCcaau......................................................................   1       1    seq
    ....................gaaaugacucucgacaUu......................................................................   1       1    seq
    ....................gaaaugacucucgacaaA......................................................................   1       1    seq
    ....................gaaaugacucucgacaaugga...................................................................   1       0    seq
    ....................gaaaugacucucgacaauggaua.................................................................   1       0    seq
    ....................gaaaugacucucgacaauggauauc...............................................................   1       0    seq
    ....................gaaaugacucucgUcaauggauaucuc.............................................................   1       1    seq
    .....................aaaugacucucgacaaA......................................................................   1       1    seq
    ........................ugacucucgacaaAggauaucucggc..........................................................   1       1    seq
    .........................gacucucgacaauggauaucu..............................................................   1       0    seq
    ..........................acucucgacaauggaCa.................................................................   1       1    seq
    ..................................caauggauaucucggcu.........................................................   1       0    seq
    ..................................caauggauaucucgCcu.........................................................   1       1    seq
    ..................................caauggauaucucggcCc........................................................   1       1    seq
    ..................................caauggauaucucggcuc........................................................   1       0    seq
    ..................................caauggauaucucggcucuC......................................................   1       1    seq
    ..................................caauggauaucucggcucuCg.....................................................   8       1    seq
    ..................................caauggauaucucggcucuCgu....................................................   2       1    seq
    ...................................aaGggauaucucggcuc........................................................   1       1    seq
    ...................................aaAggauaucucggcucu.......................................................   1       1    seq
    ...................................aauggauaucucggcucuCg.....................................................   3       1    seq
    ...................................aauggauaucucggcucuCguc...................................................   1       1    seq
    ....................................auggauaucucggcucu.......................................................   4       0    seq
    ....................................auggauaucucggcucC.......................................................   2       1    seq
```

Mature **Star**

```
ggguucgucaggaaugugaggaaaugacucucgacaauggauaucucggcucuugucacgaugacgaauguagcgaaaugcaauacuuagugugaauugaagaauccc
....................................auggauaucucggcucG.......................................................	1	1	seq
....................................auggauaucucggcucuC......................................................	1	1	seq
....................................auggauaucucggcucuCg.....................................................	12	1	seq
....................................auggauaucucggcucuCgu....................................................	9	1	seq
.....................................Gggauaucucggcucuu......................................................	1	1	seq
.....................................uggauaucucggcucuu......................................................	1	0	seq
.....................................uggauaucucggcucuC......................................................	6	1	seq
.....................................uggauaucucggcucuCg.....................................................	59	1	seq
.....................................uggauaucucggcucuCgu....................................................	9	1	seq
.....................................uggauaucucggcucuCguc...................................................	4	1	seq
.....................................uggauaucucggcucuCgucacga...............................................	1	1	seq
......................................ggauaucucggcucuug.....................................................	13	0	seq
......................................ggauaucucggcCcuug.....................................................	1	1	seq
......................................ggauaucucggcucuAg.....................................................	3	1	seq
......................................ggauaucucggcucAug.....................................................	1	1	seq
......................................ggauaucucggcucuCg.....................................................	2788	1	seq
......................................ggauaucucggcucuugu....................................................	1	0	seq
......................................ggauaucucggcucuCgu....................................................	793	1	seq
......................................ggauaucucggcucuGgu....................................................	1	1	seq
......................................ggauaucucggcucuuguU...................................................	1	1	seq
......................................ggauaucucggcucuCguc...................................................	205	1	seq
......................................ggauaucucggcucuuguUa..................................................	1	1	seq
......................................ggauaucucggcucuCguca..................................................	3	1	seq
......................................ggauaucucggcucuCgucacga...............................................	1	1	seq
......................................ggauaucucggcucuuguUacgau..............................................	1	1	seq
......................................ggauaucucggcucuCgucacgauga............................................	1	1	seq
.......................................gauaucucggcucuugC....................................................	2	1	seq
.......................................gauaucucggcucuCgu....................................................	3182	1	seq
.......................................gauaucucggcucuAgu....................................................	2	1	seq
.......................................gauaucucggcucuugu....................................................	4	0	seq
.......................................gauaucucggcucuuguG...................................................	1	1	seq
.......................................gauaucucggcucuCguc...................................................	1148	1	seq
.......................................gauaucucggcucuuguc...................................................	2	0	seq
.......................................gauaucucggcucuuguU...................................................	7	1	seq
.......................................gauaucucggcucuCguca..................................................	7	1	seq
.......................................gauaucucggcucuuguUa..................................................	1	1	seq
.......................................gauaucucggcucuCgucacg................................................	5	1	seq
.......................................gauaucucggcucuuguUacg................................................	6	1	seq
.......................................gauaucucggcucuuguUacgau..............................................	1	1	seq
.......................................gauaucucggcucuCgucacgau..............................................	4	1	seq
.......................................gauaucucggcucuuguUacgaug.............................................	4	1	seq
.......................................gauaucucggcucuCgucacgaug.............................................	3	1	seq
.......................................gauaucucggcucuCgucacgauga............................................	1	1	seq
........................................auaucucggcucuCguc...................................................	1045	1	seq
........................................auaucucggcucuuguU...................................................	7	1	seq
........................................auaucucggcucuuguc...................................................	1	0	seq
........................................auaucucggcucuCguca..................................................	6	1	seq
........................................auaucucggcucuuguUac.................................................	1	1	seq
........................................auaucucggcucuCgucacg................................................	4	1	seq
........................................auaucucggcucuuguUacg................................................	7	1	seq
........................................auaucucggcucuuguUacga...............................................	3	1	seq
........................................auaucucggcucuCgucacga...............................................	4	1	seq
........................................auaucucggcucuCgucacgau..............................................	2	1	seq
........................................auaucucggcucuCgucacgaug.............................................	9	1	seq
........................................auaucucggcucuuguUacgaug.............................................	3	1	seq
.........................................uaucucggcucuuguUa..................................................	6	1	seq
.........................................uaucucggcucuCguca..................................................	50	1	seq
.........................................uaucucggcucuCgucacg................................................	22	1	seq
.........................................uaucucggcucuuguUacg................................................	23	1	seq
.........................................uaucucggcucuCgucacga...............................................	13	1	seq
.........................................uaucucggcucuugucacgU...............................................	1	1	seq
.........................................uaucucggcucuuguUacga...............................................	8	1	seq
.........................................uaucucggcucuuguUacgau..............................................	5	1	seq
.........................................uaucucggcucuCgucacgau..............................................	9	1	seq
.........................................uaucucggcucuCgucacgaug.............................................	33	1	seq
.........................................uaucucggcucuuguUacgaug.............................................	29	1	seq
.........................................uaucucggcucuuguUacgauga............................................	9	1	seq
.........................................uaucucggcucuCgucacgauga............................................	9	1	seq
..........................................aucucggcucuuguUac.................................................	1	1	seq
..........................................aucucggcucuCgucacg................................................	2	1	seq
```

Mature **Star**

```
ggguucgucaggaaugugaggaaaugacucucgacaauggauaucucggcucuugucacgaugacgaauguagcgaaaugcaauacuuagugugaauugaagaauccc
..........................................aucucggcucuuguUacg................................................  1   1  seq
..........................................aucucggcucuCgucacga...............................................  5   1  seq
..........................................aucucggcucuuguUacga...............................................  1   1  seq
..........................................aucucggcucuuguUacgau..............................................  3   1  seq
..........................................aucucggcucuCgucacgau..............................................  2   1  seq
..........................................aucucggcucuCgucacgaug.............................................  6   1  seq
..........................................aucucggcucuuguUacgaug.............................................  2   1  seq
..........................................aucucggcucuuguUacgauga............................................  1   1  seq
..........................................aucucggcucuCgucacgauga............................................  1   1  seq
...........................................ucucggcucuCgucacg................................................  23  1  seq
...........................................ucucggcucuuguUacg................................................  32  1  seq
...........................................ucucggcucuuguUacga...............................................  5   1  seq
...........................................ucucggcucuCgucacga...............................................  8   1  seq
...........................................ucucggcucuCgucacgau..............................................  14  1  seq
...........................................ucucggcucuuguUacgau..............................................  9   1  seq
...........................................ucucggcucuCgucacgaug.............................................  45  1  seq
...........................................ucucggcucuuguUacgaug.............................................  29  1  seq
...........................................ucucggcucuuguUacgauga............................................  5   1  seq
...........................................ucucggcucuCgucacgauga............................................  12  1  seq
............................................cucggcucuuguUacga...............................................  4   1  seq
............................................cucggcucuCgucacga...............................................  5   1  seq
............................................cucggcucuCgucacgau..............................................  13  1  seq
............................................cucggcucuuguUacgau..............................................  9   1  seq
............................................cucggcucuCgucacgaug.............................................  29  1  seq
............................................cucggcucuuguUacgaug.............................................  32  1  seq
............................................cucggcucuCgucacgauga............................................  5   1  seq
............................................cucggcucuuguUacgauga............................................  8   1  seq
.............................................ucggcucuuguUacgau..............................................  2   1  seq
.............................................ucggcucuCgucacgau..............................................  2   1  seq
.............................................ucggcucuCgucacgaug.............................................  20  1  seq
.............................................ucggcucuuguUacgaug.............................................  16  1  seq
.............................................ucggcucuCgucacgauga............................................  9   1  seq
.............................................ucggcucuuguUacgauga............................................  10  1  seq
..............................................cggcucuCgucacgaug.............................................  16  1  seq
..............................................cggcucuuguUacgaug.............................................  18  1  seq
..............................................cggcucuCgucacgauga............................................  7   1  seq
..............................................cggcucuuguUacgauga............................................  3   1  seq
.........................................................acgaugacgaaCguagc..................................  1   1  seq
.........................................................acgaugaAgaauguagc..................................  2   1  seq
.........................................................acgaugaAgaauguagcga................................  3   1  seq
.........................................................acgaugaAgaauguagcgaa...............................  1   1  seq
.........................................................acgaugaAgaauguagcgaaa..............................  1   1  seq
.........................................................acgaugaAgaauguagcgaaaug............................  1   1  seq
..........................................................cgaugacgaaCguagcg.................................  1   1  seq
..........................................................cgaugaAgaauguagcg.................................  4   1  seq
..........................................................cgaugaAgaauguagcga................................  2   1  seq
..........................................................cgaugaAgaauguagcgaa...............................  6   1  seq
..........................................................cgaugaAgaauguagcgaaa..............................  4   1  seq
..........................................................cgaugacgaaCguagcgaaa..............................  1   1  seq
..........................................................cgaugacgaaCguagcgaaau.............................  1   1  seq
..........................................................cgaugaAgaauguagcgaaau.............................  3   1  seq
..........................................................cgaugaAgaauguagcgaaaug............................  1   1  seq
..........................................................cgaugaAgaauguagcgaaaugc...........................  2   1  seq
...........................................................gaugacgaaCguagcga................................  1   1  seq
............................................................augacgaaCguagcgaaaug............................  1   1  seq
.............................................................ugacgaaCguagcgaaa..............................  1   1  seq
..............................................................gacgaaCguagcgaaau.............................  1   1  seq
..............................................................gacgaaCguagcgaaaug............................  1   1  seq
................................................................cgaaCguagcgaaaugc...........................  1   1  seq
.................................................................gaaCguagcgaaaugca..........................  8   1  seq
.................................................................gaaCguagcgaaaugcaauacuuaguguga.............  1   1  seq
..................................................................aaCguagcgaaaugcaauacuuaguguga.............  1   1  seq
...................................................................auguagcgaaaugcGauacuuagugugaau...........  1   1  seq
....................................................................Cguagcgaaaugcaauac......................  1   1  seq
....................................................................Cguagcgaaaugcaauacuuaguguga.............  1   1  seq
....................................................................Cguagcgaaaugcaauacuuagugugaa............  2   1  seq
....................................................................uguagcgaaaugcGauacuuagugugaau...........  1   1  seq
....................................................................Cguagcgaaaugcaauacuuagugugaau...........  1   1  seq
....................................................................uguagcgaaaugcGauacuuagugugaauu..........  1   1  seq
.....................................................................guagcgaaaugcaauacuuagugugaa............  1   0  seq
```

```
                                   Mature      Star
ggguucgucaggaaugugaggaaaugacucucgacaauggauaucucggcucuugucacgaugacgaauguagcgaaaugcaauacuuagugugaauugaagaauccc
......................................................................uagcgaaaugcGauacuuagugugaauuga........   3    1    seq
.......................................................................agcgaaaugcaauacuuagug................   1    0    seq
.......................................................................agcgaaaugcGauacuuagugugaauuga........   1    1    seq
........................................................................gcgaaaugcaauacuuagugugaau...........   1    0    seq
........................................................................gcgaaaugcGauacuuagugugaauuga........   1    1    seq
........................................................................gcgaaaugcaauacuuagugugaauugCaga.....   1    1    seq
.........................................................................cgaaaugcGauacuuagugugaauuga........   6    1    seq
...........................................................................aaaugcaauacuuagugugaa............   1    0    seq
...........................................................................aaaugcaauacuuagugugaau...........   1    0    seq
............................................................................aaugcaauacuuaguguga.............   1    0    seq
.............................................................................augcaauacuuagugugaau...........   1    0    seq
.............................................................................augcaauacuuagugugaauu..........   2    0    seq
.............................................................................augcaGuacuuagugugaauu..........   1    1    seq
..............................................................................ugcaauacuuagugCga.............   1    1    seq
..............................................................................ugcaauacuuaguguga.............   1    0    seq
..............................................................................ugcaauacuuagugugaa............   7    0    seq
..............................................................................ugcaauacuuagugugaau...........   3    0    seq
..............................................................................ugcCauacuuagugugaau...........   1    1    seq
..............................................................................ugcaauacuuagugugaauu..........   5    0    seq
..............................................................................ugcCauacuuagugugaauu..........   1    1    seq
...............................................................................gcaauacuuagugugaG............   1    1    seq
...............................................................................gAaauacuuagugugaa............   1    1    seq
...............................................................................gcaauacuuagugugaau...........   1    0    seq
...............................................................................gcaauacuuagugugaauA..........   1    1    seq
...............................................................................gcaauacuuagugugaauu..........   4    0    seq
...............................................................................gcCauacuuagugugaauu..........   1    1    seq
................................................................................caauacuuagugugaauu..........   3    0    seq
.................................................................................aauacuuagugugaauG..........   1    1    seq
..................................................................................auacuuagugugaauug.........   17   0    seq
..................................................................................auacuuagugugaauuga........   15   0    seq
..................................................................................auacuuagugugaauugG........   2    1    seq
..................................................................................auacuuagugugaauuCa........   1    1    seq
..................................................................................auacuuagugugaauugaaga.....   1    0    seq
..................................................................................auacuuagugugaauugGagaauc..   1    1    seq
...................................................................................uacuuagugugaauuga........   40   0    seq
...................................................................................uacuuagCgugaauuga........   1    1    seq
...................................................................................uacuuagugugaauuCa........   1    1    seq
...................................................................................uacuuagugugGauuga........   1    1    seq
...................................................................................uacuuagugCgaauuga........   1    1    seq
...................................................................................uacuuagugugaauugG........   3    1    seq
...................................................................................uacuuagugugaauugGa.......   1    1    seq
...................................................................................uacuuagugugaauugGaga.....   1    1    seq
...................................................................................uacuuagugugaauugaagaau...   1    0    seq
....................................................................................acuuagugugaauugGaga.....   1    1    seq
.....................................................................................cuuagugugaauugaag......   1    0    seq
.....................................................................................cuuagugugaauugGag......   1    1    seq
.....................................................................................cuuagugugaauugGaga.....   1    1    seq
.....................................................................................cuuagugugaauugaaga.....   3    0    seq
.....................................................................................cuuagugugaauugaagaa....   1    0    seq
.....................................................................................cuuagugugaauugGagaau...   1    1    seq
.....................................................................................cuuagugugaauugaagaauc..   1    0    seq
.....................................................................................cuuagugugaauugaagaaucc.   1    0    seq
.....................................................................................cuuagugugaauugaagaauccU   1    1    seq
.....................................................................................cuuagugugaauugGagaauccc   1    1    seq
......................................................................................uuagugugaauugaaga.....   2    0    seq
.......................................................................................uagugugaauugGagaa....   1    1    seq
.......................................................................................uagugugaauugaagaau...   1    0    seq
.......................................................................................uagugugaauugaagaauc..   1    0    seq
.......................................................................................uagugugaauugGagaauc..   2    1    seq
.......................................................................................uagugugaauugaagaaucc.   1    0    seq
.........................................................................................gugugaauugaagaauc..   2    0    seq
.........................................................................................gugugaauugGagaauc..   3    1    seq
.........................................................................................gugugaauugGagaaucc.   2    1    seq
.........................................................................................gugugaauugaagaaucc.   3    0    seq
.........................................................................................gugugaauugGagaauccc   3    1    seq
..........................................................................................ugugaauugaagaaucc.   1    0    seq
..........................................................................................ugugaauugGagaaucc.   1    1    seq
..........................................................................................ugugaauugaagaauccc   1    0    seq
...........................................................................................gugaauugGagaauccc   2    1    seq
```

Provisional ID	:	MA_101556_10714
Score total	:	653.9
Score for star read(s)	:	3.9
Score for read counts	:	646.1
Score for mfe	:	2.4
Score for randfold	:	1.6
Score for cons. seed	:	
Total read count	:	1279
Mature read count	:	983
Loop read count	:	15
Star read count	:	281

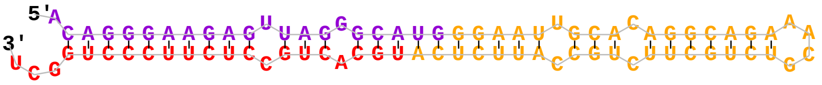

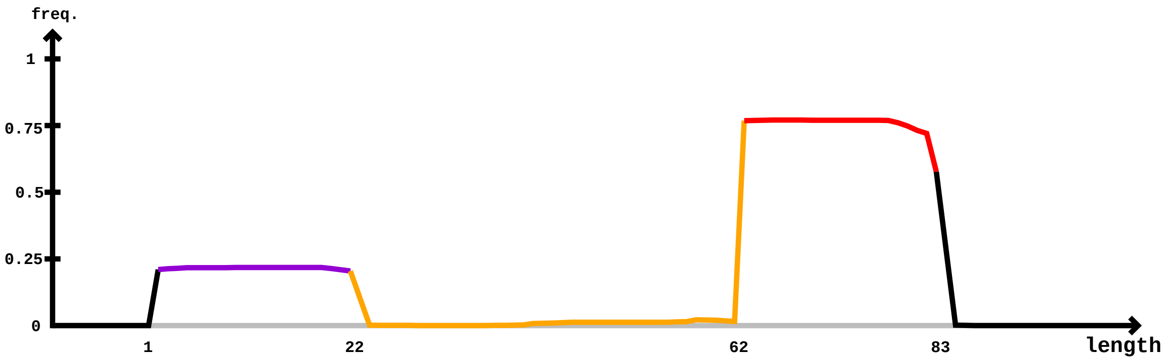

Star   Mature

```
5'- cacagagagacagggaagaguuagggcaugggaauugcacaggcagaaacgucugcuucugccauucucaugcacugccucuucccuggcucacuucuucuccuguuauau  -3'  obs
    cacagagagacagggaagaguuagggcaugggaauugcacaggcagaaacgucugcuucugccauucucaugcacugccucuucccuggcucacuucuucuccuguuauau       exp
    .((((.((((((((((((((.(((.((((((((((.(((.(((((((....))))))).))).))))))))))).))).))))))))))..........)))))))).....  reads  mm  sample
    .........acagggaagaguuagggc....................................................................................  2  0  seq
    .........acagggaUgaguuagggc....................................................................................  2  1  seq
    .........acagggaUgaguuagggca...................................................................................  4  1  seq
    .........acagggaagaguuagggcau..................................................................................  2  0  seq
    .........acagggaUgaguuagggcau..................................................................................  2  1  seq
    .........acagggaagGguuagggcaug.................................................................................  1  1  seq
    .........acagggaagaguuaAggcaug.................................................................................  1  1  seq
    .........acagggaagaguuagggcauA.................................................................................  5  1  seq
    .........acagggaagagCuagggcaug.................................................................................  1  1  seq
    .........Ncagggaagaguuagggcaug.................................................................................  1  1  seq
    .........acagggaagaguuagggcaAg.................................................................................  1  1  seq
    .........acagggaUgaguuagggcaug.................................................................................  132  1  seq
    .........acagggGagaguuagggcaug.................................................................................  1  1  seq
    .........acGgggaagaguuagggcaug.................................................................................  3  1  seq
    .........acagggaagaguuagggcaug.................................................................................  111  0  seq
    .........acagggaagaguuaggAcaug.................................................................................  1  1  seq
    .........acagggaagaguuUgggcaug.................................................................................  1  1  seq
    .........acagggaagaguuagggcaugA................................................................................  1  1  seq
    ..........cagggaUgaguuagggc....................................................................................  1  1  seq
    ..........cagggaUgaguuagggca...................................................................................  2  1  seq
    ..........cagggaUgaguuagggcau..................................................................................  1  1  seq
    ...........agggaagaguuagggcau..................................................................................  1  0  seq
    ...........agggaUgaguuagggcauggg...............................................................................  1  1  seq
    ............gggaUgaguuagggcaug.................................................................................  1  1  seq
    ............gggaagaguuagggcaug.................................................................................  2  0  seq
    .................gaguuagggcaugggaGuu...........................................................................  1  1  seq
    ............................................agaaacgucugcuucugccauucucaugcGcug..................................  1  1  seq
    ..............................................aaacgucugcuucugcAauu.............................................  1  1  seq
    ...............................................aacgucugcuucugcAauucuca.........................................  1  1  seq
    ................................................acgucugcuucugcAauu.............................................  2  1  seq
    ................................................acgucugcuucugcAauuc............................................  1  1  seq
    ................................................acgucugcuucugcAauucu...........................................  2  1  seq
    ................................................acgucugcuucugcGauucu...........................................  1  1  seq
```

Star / Mature			
cacagagagacagggaagaguuagggcaugggaauugcacaggcagaaacgucugcuucugccauucucaugcacugccucuucccuggcucacuucuucuccuguuauau			
....acgucugcuucugcAauucuc..	1	1	seq
....cgucugcuucugcAauucuc..	1	1	seq
....gucugcuucugcAauucuc..	1	1	seq
....ucugcuucugcAauucuca...	1	1	seq
....ucugcuucugccauucucaug.......................................	1	0	seq
....cugcuucugcAauucuc..	1	1	seq
....cugcuucugcAauucuca...	1	1	seq
....auucucUugcacugccucuuccc.........................	1	1	seq
....auucucUugcacugccucuucccu........................	1	1	seq
....uucucaugcacugccucuucc..........................	1	0	seq
....ucucUugcacugccucuuccc.........................	2	1	seq
....ucucaugcacCgccucuuccc.........................	1	1	seq
....ucucaugcacugccucuuccc.........................	5	0	seq
....ucucaugcacugccucuucccU........................	1	1	seq
....cucaugcacugccucuuccc.........................	1	0	seq
....cucaugcacugccucuucccu........................	1	0	seq
....Cugcacugccucuucccuggcu....................	1	1	seq
....ugcacugccucuucccC........................	2	1	seq
....ugcacugccucuucccu........................	12	0	seq
....ugcacugccucuucccuU.......................	1	1	seq
....ugcacugccucuucccug.......................	16	0	seq
....ugcacugccucuucccuC.......................	1	1	seq
....ugcacugccucuucccuA.......................	1	1	seq
....ugcacugccucuucccAg.......................	1	1	seq
....ugcacugccucuucccugg......................	8	0	seq
....ugcacugccucuucccugA......................	1	1	seq
....ugcacugccucuucccugU......................	5	1	seq
....Ggcacugccucuucccugg......................	1	1	seq
....ugcacugccucuucccuggU.....................	21	1	seq
....ugcacugccucuucccugCc.....................	1	1	seq
....ugcacugccCcuucccuggc.....................	1	1	seq
....Agcacugccucuucccuggc.....................	1	1	seq
....ugcacugccucuucccuggA.....................	3	1	seq
....ugUacugccucuucccuggc.....................	1	1	seq
....ugcacugccucuucccuggc.....................	155	0	seq
....ugcacugccucuucccuAgcu....................	2	1	seq
....ugcacugccucuucccugUcu....................	1	1	seq
....ugcacuCccucuucccuggcu....................	1	1	seq
....ugcaGugccucuucccuggcu....................	1	1	seq
....ugcacugccuGuucccuggcu....................	1	1	seq
....ugUacugccucuucccuggcu....................	1	1	seq
....ugcacugccucuucccuggGu....................	1	1	seq
....ugcacugccucuucccuggcu....................	538	0	seq
....ugcacugccCcuucccuggcu....................	5	1	seq
....ugcacugccucuucccugAcu....................	4	1	seq
....ugcacugcGucuucccuggcu....................	2	1	seq
....ugcacugccucuucccAggcu....................	1	1	seq
....ugcacugccucuucccuggcA....................	29	1	seq
....ugcacugccucuucccuggcG....................	26	1	seq
....ugcacugccucuucccuggcC....................	104	1	seq
....ugcacugccucuucccCggcu....................	3	1	seq
....uUcacugccucuucccuggcu....................	1	1	seq
....ugcGcugccucuucccuggcu....................	1	1	seq
....Cgcacugccucuucccuggcu....................	4	1	seq
....ugcacCgccucuucccuggcu....................	1	1	seq
....ugcacugccucCucccuggcu....................	4	1	seq
....ugcacugccucuucccuggcuc...................	3	0	seq
....ugcacugccucuucccuggcuA...................	3	1	seq
....ugcacugccucuucccuggcuU...................	7	1	seq
....ugcacugccucuucccuggcuca..................	1	0	seq
....ugcacugccucuucccuggcuUac.................	1	1	seq
....gcacugccucuucccug.......................	1	0	seq
....cacugccucuucccuggc.....................	2	0	seq
....acugccucuucccuggc.....................	1	0	seq

Provisional ID	:	MA_101956_1593
Score total	:	0.7
Score for star read(s)	:	-1.3
Score for read counts	:	0
Score for mfe	:	0.4
Score for randfold	:	1.6
Score for cons. seed	:	
Total read count	:	9929
Mature read count	:	9929
Loop read count	:	0
Star read count	:	0

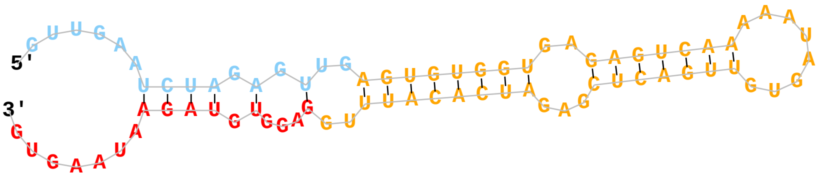

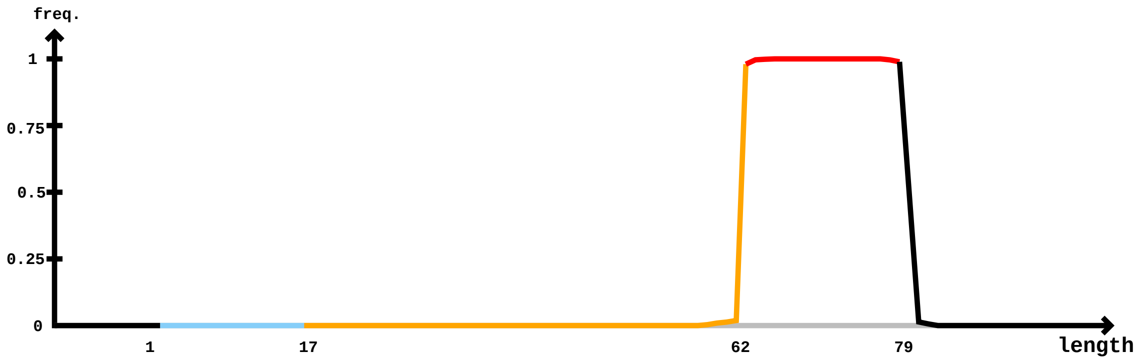

Star Mature

```
5'- uuuggugcaguugaaucuagaguugaguguggugagagucaaaaauaguguugacucgagaucacauuuggagguguagaauaagugacacacaacugggagggucuc   -3'    exp
    .((((((((.(((..((((.(.(..((((((((..(((((((........)))))))...))))))))..)...).)))).))).)).))).)))..(((.....)))     reads      mm        sample
    ..................................................................uuGggagguguagaauaag.......................     10         1         seq
    ..................................................................Cuuggagguguagaauaagu......................     1          1         seq
    ..................................................................uuGggagguguagaauaagu......................     10         1         seq
    ..................................................................uuGggagguguagaauaagug.....................     13         1         seq
    ...................................................................uGggagguguagaauaag.......................     18         1         seq
    ...................................................................uGggagguguagaauaagu......................     22         1         seq
    ...................................................................uGggagguguagaauaagug.....................     19         1         seq
    ...................................................................uGggagguguagaauaaguga....................     1          1         seq
    ....................................................................Nggagguguagaauaag.......................     2          1         seq
    ....................................................................Gggagguguagaauaag.......................     7          1         seq
    ....................................................................uUgagguguagaauaag.......................     1          1         seq
    ....................................................................uggagguguagaauaagu......................     1          0         seq
    ....................................................................uUgagguguagaauaagu......................     2          1         seq
    ....................................................................Gggagguguagaauaagu......................     9          1         seq
    ....................................................................Gggagguguagaauaagug.....................     11         1         seq
    ....................................................................uUgagguguagaauaagug.....................     2          1         seq
    ....................................................................Gggagguguagaauaaguga....................     2          1         seq
    .....................................................................Ugagguguagaauaagu......................     4          1         seq
    .....................................................................ggagguguagaauaagC......................     2          1         seq
    .....................................................................ggGgguguagaauaagu......................     1          1         seq
    .....................................................................Cgagguguagaauaagu......................     1          1         seq
    .....................................................................ggagguguagaauaagu......................     17         0         seq
    .....................................................................Ngagguguagaauaagu......................     3          1         seq
    .....................................................................Ugagguguagaauaagug.....................     6          1         seq
    .....................................................................ggagguguagaauaagug.....................     12         0         seq
    .....................................................................Ngagguguagaauaagug.....................     7          1         seq
    .....................................................................Cgagguguagaauaagug.....................     2          1         seq
    .....................................................................ggagguguagaauaagugG....................     3          1         seq
    .....................................................................Agagguguagaauaagugaca..................     1          1         seq
    ......................................................................gaggCguagaauaagug.....................     32         1         seq
    ......................................................................gagguguGgaauaagug.....................     20         1         seq
    ......................................................................gagguguagaaGaagug.....................     2          1         seq
    ......................................................................gagguguagGauaagug.....................     27         1         seq
    ......................................................................gagguguagaauaCgug.....................     2          1         seq
```

Star **Mature**

uuuggugcaguugaaucuagaguugaguguggugagagucaaaaauaguguugacucgagaucacauuuggagguguagaauaagugacacacaacugggagggucuc			
....gaggAguagaauaagug.....................	6	1	seq
....Cagguguagaauaagug.....................	5	1	seq
....gagguguagaauaaUug.....................	4	1	seq
....gaggugAagaauaagug.....................	2	1	seq
....gaggugCagaauaagug.....................	18	1	seq
....gagguguagaauaagGg.....................	31	1	seq
....gagguguUgaauaagug.....................	7	1	seq
....gagguguagaauaaguC.....................	28	1	seq
....gaCguguagaauaagug.....................	1	1	seq
....gagguguCgaauaagug.....................	1	1	seq
....gagguguagCauaagug.....................	1	1	seq
....gagguguagaauaagug.....................	9116	0	seq
....Nagguguagaauaagug.....................	13	1	seq
....gagguCuagaauaagug.....................	1	1	seq
....gUgguguagaauaagug.....................	6	1	seq
....gagguAuagaauaagug.....................	5	1	seq
....gaggugGagaauaagug.....................	4	1	seq
....gGgguguagaauaagug.....................	26	1	seq
....gagguguagaauaaAug.....................	4	1	seq
....gagguguagGauaaguga....................	1	1	seq
....gagguguagaauaGguga....................	2	1	seq
....gagguguagaauaaguga....................	182	0	seq
....gagguguagaauaagugC....................	24	1	seq
....gGgguguagaauaaguga....................	1	1	seq
....gaAguguagaauaaguga....................	2	1	seq
....Uagguguagaauaaguga....................	1	1	seq
....gagguguagaaAaaguga....................	1	1	seq
....gagguguagaauaagugGc...................	41	1	seq
....gagguguagaauaagugGca..................	16	1	seq
....gagguguagaauaagugaca..................	1	0	seq
....agguguagaaCaaguga....................	1	1	seq
....agguguagaauaaguga....................	100	0	seq
....agguguagaauaagugC....................	15	1	seq
....aUguguagaauaaguga....................	1	1	seq
....agguguagaauaGguga....................	2	1	seq
....Ggguguagaauaaguga....................	1	1	seq
....agguguagaauaaguAa....................	1	1	seq
....agguguagaauaaguUa....................	1	1	seq
....agguguagaauaagugGc...................	22	1	seq
....agguguagaauaagugGca..................	19	1	seq
....agguguagaauaagugaAa..................	1	1	seq
....gguguagaauaagugGc...................	10	1	seq
....gguguagaauaagugCc...................	1	1	seq
....gguguagaauaagugGca..................	8	1	seq
....gguguagaauaagugaca..................	2	0	seq
....guguagaauaagugGca..................	14	1	seq

Provisional ID	: MA_102331_14629
Score total	: 0
Score for star read(s)	: -1.3
Score for read counts	: 0
Score for mfe	: -0.3
Score for randfold	: 1.6
Score for cons. seed	:
Total read count	: 500
Mature read count	: 500
Loop read count	: 0
Star read count	: 0

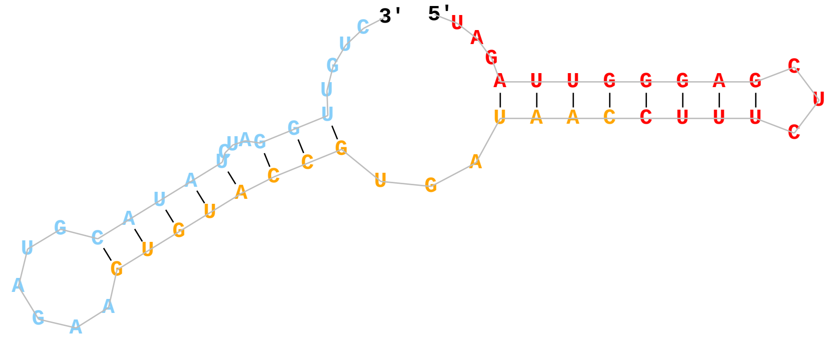

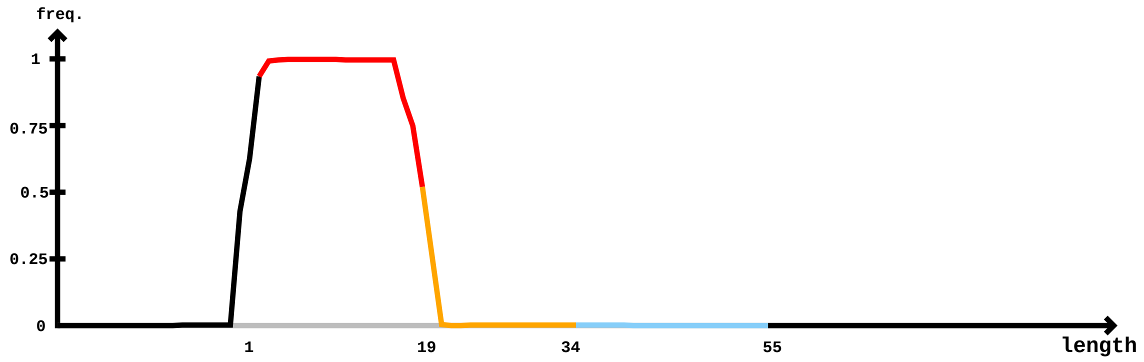

Mature Star

5'- auauugaaugugauuuggauagauugggagcucuuuccaauagugccaugugaagaugcauaucuagguuguccaugagucacuauuguaaagugauucaacacuaaa -3'	exp		
.........(((...(((((((.(((((.((((((..((.........))..)))).))...))))).))))))).((((((((.......))))))))..)))....	reads	mm	sample
...........gauuuggauagauuUgg..	1	1	seq
.................gaCagauugggagcucu..	72	1	seq
.................gaCagauugggagcucuu...	27	1	seq
.................gaCagauugggagcucuuu..	38	1	seq
.................gaCagauugggagcucuuuc...	63	1	seq
.................gaCagauugggagcucuuucc..	12	1	seq
.................gauagauugAgagcucuuucc..	2	1	seq
..................aCagauugggagcucuu...	25	1	seq
..................aCagauugggagcucuuu..	28	1	seq
..................aCagauugggagcucuuuc...	44	1	seq
..................aCagauugggagcucuuucc..	1	1	seq
..................auagauugAgagcucuuucc..	1	1	seq
...................Cagauugggagcucuuu..	49	1	seq
...................Cagauugggagcucuuuc...	100	1	seq
...................Cagauugggagcucuuucc..	4	1	seq
...................uagauugAgagcucuuucc..	2	1	seq
....................agauugUgagcucuuuc...	1	1	seq
....................agauugggagcucuuuc...	21	0	seq
....................agauugggagcucuuucc..	4	0	seq
....................agauugCgagcucuuucc..	1	1	seq
....................agauugggagcucuuucA..	1	1	seq
....................agauugAgagcucuuucca...	1	1	seq
.....................gauugggagcucuuucc..	2	0	seq
......................auugAgagcucuuucca...	1	1	seq
....agugAcaugugaagaug..	1	1	seq

Provisional ID	:	MA_10248616_31320
Score total	:	1.5
Score for star read(s)	:	-1.3
Score for read counts	:	0
Score for mfe	:	1.2
Score for randfold	:	1.6
Score for cons. seed	:	
Total read count	:	21939
Mature read count	:	21939
Loop read count	:	0
Star read count	:	0

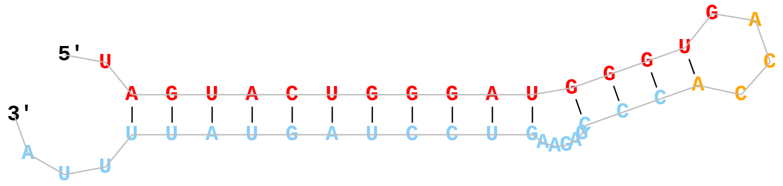

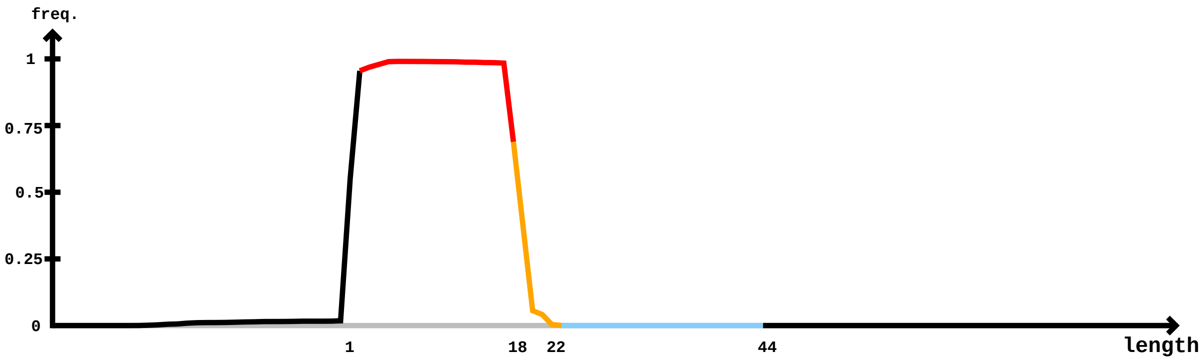

Mature **Star**

```
5'- acuucauaguuaagcgugcuugggcuaguguaguacugggaugggugaccacccgagaaguccuaguauuuuacaugccuauggcauuuuauaaacuuaguccaauuuaucccac   -3'    exp
    (((....((((....((((((((((..(((.((((((((((((((((....)))).....)))))))))))).)))..))))).)))))......)))).)))............   reads      mm         sample
    .......aguuaagcgugcuugggcu.........................................................................................   1          0          seq
    .......aguuaagcgCgcuugggcuagu......................................................................................   4          1          seq
    ........guuaagcgugcuugggcu.........................................................................................   4          0          seq
    ........guuaagcgugcuugggcG.........................................................................................   1          1          seq
    ........guuaagcgugcuugggcua........................................................................................   1          0          seq
    ........guuaagcgCgcuugggcuagu......................................................................................   13         1          seq
    ........guuaagcgCgcuugggcuagugu....................................................................................   1          1          seq
    ........guuaagcgCgcuugggcuagugua...................................................................................   2          1          seq
    ........guuaagcgCgcuugggcuaguguagu.................................................................................   1          1          seq
    ........guuaagcgCgcuugggcuaguguaguacugggaug........................................................................   1          1          seq
    .........uuaagcgugcuugggcu.........................................................................................   4          0          seq
    .........uuaagcgugcuugggcua........................................................................................   2          0          seq
    .........uuaagcgCgcuugggcuagu......................................................................................   15         1          seq
    .........uuaagcgCgcuugggcuagug.....................................................................................   2          1          seq
    .........uuaagcgugcuugggcuagGg.....................................................................................   1          1          seq
    .........uuaagcgCgcuugggcuagugu....................................................................................   5          1          seq
    .........uuaagcgCgcuugggcuagugua...................................................................................   1          1          seq
    .........uuaagcgCgcuugggcuaguguag..................................................................................   1          1          seq
    .........uuaagcgCgcuugggcuaguguagua................................................................................   1          1          seq
    ..........uaagcgugcuugggcuag.......................................................................................   3          0          seq
    ..........uaagcgCgcuugggcuagu......................................................................................   33         1          seq
    ..........uaagcgCgcuugggcuagug.....................................................................................   2          1          seq
    ..........uaagcgCgcuugggcuagugu....................................................................................   5          1          seq
    ..........uaagcgCgcuugggcuagugua...................................................................................   4          1          seq
    ..........uaagcgCgcuugggcuaguguag..................................................................................   1          1          seq
    ..........uaagcgCgcuugggcuaguguagua................................................................................   1          1          seq
    ..........uaagcgCgcuugggcuaguguaguacugggaugggu.....................................................................   1          1          seq
    ...........aagcgugcuugggcuag.......................................................................................   1          0          seq
    ...........aagcgCgcuugggcuagu......................................................................................   16         1          seq
    ...........aagcgCgcuugggcuagugu....................................................................................   2          1          seq
    ...........aagcgCgcuugggcuagugua...................................................................................   1          1          seq
    ...........aagcgCgcuugggcuaguguagua................................................................................   2          1          seq
    ...........aagcgCgcuugggcuaguguaguacugggauggg......................................................................   2          1          seq
    ............agcgCgcuugggcuagu......................................................................................   37         1          seq
```

Mature Star

acuucauaguuaagcgugcuugggcuagug**uaguacugggaugggug**acca**cccgagaaguccuaguauuuua**caugccuauggcauuuuauaaacuuaguccaauuuaucccac

............agcgCgcuugggcuagug...	7	1	seq
............agcgCgcuugggcuagugu..	6	1	seq
............agcgCgcuugggcuagugua...	5	1	seq
............agcgCgcuugggcuaguguag..	3	1	seq
............agcgCgcuugggcuaguguagu...	1	1	seq
............agcgCgcuugggcuaguguaguacug...	1	1	seq
............agcgGgcuugggcuaguguaguacuggga..	1	1	seq
............agcgCgcuugggcuaguguaguacugggaug..	1	1	seq
............agcgCgcuugggcuaguguaguacugggaugggu...	2	1	seq
.............gcgCgcuugggcuagug...	13	1	seq
.............gcgCgcuugggcuagugu..	4	1	seq
.............gcgCgcuugggcuagugua...	2	1	seq
.............gcgCgcuugggcuaguguag..	1	1	seq
.............gcgugcuugggcuagGguag..	1	1	seq
.............gcgCgcuugggcuaguguagu...	1	1	seq
.............gcgCgcuugggcuaguguagua..	1	1	seq
.............gcgCgcuugggcuaguguaguacu..	2	1	seq
.............gcgCgcuugggcuaguguaguacug...	1	1	seq
.............gcgCgcuugggcuaguguaguacuggg...	1	1	seq
.............gcgCgcuugggcuaguguaguacuggga..	1	1	seq
.............gcgCgcuugggcuaguguaguacugggau...	1	1	seq
.............gcgCgcuugggcuaguguaguacugggaug..	2	1	seq
.............gcgCgcuugggcuaguguaguacugggaugg...	1	1	seq
.............gcgCgcuugggcuaguguaguacugggauggg..	1	1	seq
.............gcgCgcuugggcuaguguaguacugggaugggug..	4	1	seq
..............cgugcuugggcuagGguaguacug...	1	1	seq
..............cgCgcuugggcuaguguaguacuggga..	3	1	seq
..............cgCgcuugggcuaguguaguacugggaug..	3	1	seq
..............cgCgcuugggcuaguguaguacugggaugg...	1	1	seq
..............cgCgcuugggcuaguguaguacugggauggg..	3	1	seq
..............cgCgcuugggcuaguguaguacugggaugggug..	4	1	seq
...............gCgcuugggcuaguguaguacugggaugggu...	1	1	seq
................Cgcuugggcuaguguaguacuggga..	3	1	seq
................ugcuugggcuagGguaguacugggau...	1	1	seq
................Cgcuugggcuaguguaguacugggaug..	1	1	seq
................Cgcuugggcuaguguaguacugggauggg..	1	1	seq
................Cgcuugggcuaguguaguacugggaugggu...	2	1	seq
................Cgcuugggcuaguguaguacugggaugggugac..	1	1	seq
.................gcuugggUuaguguagua..	1	1	seq
.................gcuugggcuaguguaguacuA...	1	1	seq
.................gcuugggcuaguguaguacuggg...	1	0	seq
.................gcuugggcuagCguaguacuggg...	1	1	seq
.................gcuugggcuaguguaguacuggga..	2	0	seq
.................gcuugggCaguguaguacuggga..	2	1	seq
.................gcuugggcuaguguaguacugggaC...	1	1	seq
.................gcuugggcuaguguaguacugggaug..	7	0	seq
.................gcuugggcuagCguaguacugggaug..	1	1	seq
.................gcuugggcuaguguaguacugggauggg..	1	0	seq
.................gcuugggcuagAguaguacugggaugggugacca..	1	1	seq
..................cuugggcuaguguagua..	1	0	seq
..................cuugggcuaguguaguacug...	2	0	seq
..................cuugggcuaguguaguacugg..	1	0	seq
..................cuugggcuaguguaguacuggg...	2	0	seq
..................cuugggcuaguguaguacuggga..	1	0	seq
..................cuugggcuagCguaguacuggga..	2	1	seq
..................cuugggcuagCguaguacugggaug..	1	1	seq
..................cuugggcuaguguaguacugggaug..	3	0	seq
..................cuugggcuaguguaguacugggaugg...	1	0	seq
..................cuugggcuagCguaguacugggaugg...	1	1	seq
..................cuugggcuaguguaguacugggauggg..	2	0	seq
..................cuugggcuaguguaguacugggaugggu...	2	0	seq
..................cuugggcuaguguaguacugggaugggC...	1	1	seq
..................cuugggcuagCguaguacugggaugggug..	1	1	seq
...................uugggcuaguguaguac...	1	0	seq
...................uugggcuagCguaguacu..	1	1	seq
...................uugggcuaguguaguacu..	1	0	seq
...................uugggcuagCguaguacug...	1	1	seq
...................uugggcuaguguaguacuggg...	3	0	seq
...................uugggcuaguguaguacuggga..	1	0	seq
...................uugggcuaguguaguacugggau...	1	0	seq

Mature Star

```
acuucauaguuaagcgugcuugggcuaguguaguacugggaugggugaccacccgagaaguccuaguauuuuacaugccuauggcauuuuauaaacuuaguccaauuuaucccac
...................uugggcuaguguaguacugggaugggu..................................................................... 1 0 seq
...................uugggcuaguguaguacugggaugggug.................................................................... 2 0 seq
....................ugggcuaguguaguacu.............................................................................. 1 0 seq
....................ugggcuaguguaguacug............................................................................. 3 0 seq
....................ugggcuaguguaguacugg............................................................................ 1 0 seq
....................ugggcuaguguaguacuggga.......................................................................... 2 0 seq
....................ugggcuaguguaguacugggauA........................................................................ 1 1 seq
....................ugggcuaguguaguacugggaug........................................................................ 2 0 seq
....................ugggcuaguguaguacugggaugg....................................................................... 1 0 seq
....................ugggcuaguguaguacugggauggU...................................................................... 1 1 seq
....................ugggcuaguguaguacugggauggg...................................................................... 4 0 seq
....................ugggcuaguguaguacugggaugggu..................................................................... 3 0 seq
....................ugggcuaguguaguacugggaugggG..................................................................... 1 1 seq
....................ugggcuagCguaguacugggaugggu..................................................................... 1 1 seq
....................ugggcuaguguaguacugggaugggug.................................................................... 1 0 seq
.....................gggcuaguguaguacugggaugggC..................................................................... 1 1 seq
......................ggcuaguguaguacugg............................................................................ 1 0 seq
......................ggcuaguguaguacuggga.......................................................................... 1 0 seq
......................ggcuaguguaguacugggaugggu..................................................................... 1 0 seq
.......................gcuaguguaguacuggg........................................................................... 1 0 seq
.......................gcuaguguaguacugCga.......................................................................... 1 1 seq
.......................gcuaguguaguacCggga.......................................................................... 1 1 seq
.......................gcuaguguaguacuggga.......................................................................... 5 0 seq
.......................gcuaguguaguacugggaug........................................................................ 2 0 seq
........................cuaguguaguacuggga.......................................................................... 5 0 seq
........................cuaguguaguacugggaC......................................................................... 2 1 seq
........................cGaguguaguacugggaug........................................................................ 1 1 seq
........................cuaguguaguacugggaug........................................................................ 2 0 seq
........................cuaguguaguacugggaugA....................................................................... 1 1 seq
........................cuaguguaguacugggauggg...................................................................... 1 0 seq
........................cuaguguaguacugggaugggu..................................................................... 4 0 seq
........................cuaguguaguacugggaugggug.................................................................... 1 0 seq
........................cuaguguaguacugggaugggugaccUccc............................................................. 1 1 seq
.........................uaguguaguacugggauggg...................................................................... 2 0 seq
..........................aguguaguacugggaug........................................................................ 1 0 seq
..........................aguguaguacugggauggg...................................................................... 1 0 seq
..........................aUuguaguacugggaugggu..................................................................... 2 1 seq
..........................aguguaguacugggaugggu..................................................................... 1 0 seq
..........................Gguguaguacugggaugggu..................................................................... 1 1 seq
...........................guguaguacugggaugA....................................................................... 1 1 seq
...........................Cuguaguacugggaugg....................................................................... 1 1 seq
...........................guguaguGcugggauggg...................................................................... 1 1 seq
...........................guguaguacugggauggg...................................................................... 1 0 seq
...........................guguaguacugggaugggu..................................................................... 1 0 seq
...........................guguaguacugggaugggC..................................................................... 1 1 seq
...........................guguaguacugggaugggug.................................................................... 2 0 seq
............................uguaguacugggauggA...................................................................... 1 1 seq
............................uCuaguacugggauggg...................................................................... 1 1 seq
............................uguaguacugggauggg...................................................................... 7 0 seq
............................Nguaguacugggauggg...................................................................... 6 1 seq
............................uguaguacugggGuggg...................................................................... 1 1 seq
............................uguaguacugggaugggu..................................................................... 4 0 seq
............................Nguaguacugggaugggu..................................................................... 6 1 seq
............................uCuaguacugggaugggu..................................................................... 1 1 seq
............................Nguaguacugggaugggug.................................................................... 4 1 seq
............................uguaguacugggaugggug.................................................................... 1 0 seq
............................uCuaguacugggaugggug.................................................................... 1 1 seq
.............................Nuaguacugggaugggu..................................................................... 13 1 seq
.............................guaguacugggaugggu..................................................................... 6541 0 seq
.............................guaguacugggaugggGg.................................................................... 41 1 seq
.............................guaguacugggaugggug.................................................................... 4915 0 seq
.............................Nuaguacugggaugggug.................................................................... 4 1 seq
.............................guaguacugggaugggGga................................................................... 1 1 seq
.............................guaguacugggaugggugC................................................................... 1 1 seq
.............................guaguacugggauggguga................................................................... 230 0 seq
.............................guaguacugggaugggugac.................................................................. 110 0 seq
.............................guaguacugggaugggugCc.................................................................. 1 1 seq
.............................guaguacugggaugggugacc................................................................. 169 0 seq
.............................guaguacugggaugggugacca................................................................ 5 0 seq
..............................uaguacugggaugggGg.................................................................... 82 1 seq
```

```
                              Mature           Star
acuucauaguuaagcgugcuugggcuaguguaguacugggaugggugaccacccgagaaguccuaguauuuuacaugccuauggcauuuuauaaacuuaguccaauuuaucccac
..............................uaguacugggaugggug....................................................................	8377	0	seq
..............................Naguacugggaugggug....................................................................	14	1	seq
..............................uaguacugggaugggugC...................................................................	1	1	seq
..............................uaguacugggauggguga...................................................................	281	0	seq
..............................Naguacugggaugggugac..................................................................	1	1	seq
..............................uaguacugggaugggugac..................................................................	84	0	seq
..............................Naguacugggaugggugacc.................................................................	1	1	seq
..............................uaguacugggaugggugacc.................................................................	179	0	seq
..............................Gaguacugggaugggugacc.................................................................	1	1	seq
..............................uaguacugggaugggugacca................................................................	18	0	seq
...............................aguacugggaugggGga...................................................................	3	1	seq
...............................aguacugggauggguga...................................................................	157	0	seq
...............................aguacugggaugggugC...................................................................	1	1	seq
...............................aguacugggaugggugac..................................................................	51	0	seq
...............................aguacugggaugggugacc.................................................................	111	0	seq
...............................aguacugggaugggGgacc.................................................................	2	1	seq
...............................aguacugggaugggugacca................................................................	2	0	seq
................................guacugggaugggugac..................................................................	76	0	seq
................................guacugggaugggugacc.................................................................	154	0	seq
................................guacugggaugggugacca................................................................	18	0	seq
................................guacugggaugggugaccac...............................................................	1	0	seq
.................................Nacugggaugggugacc.................................................................	1	1	seq
.................................uacugggaugggugacc.................................................................	217	0	seq
.................................uacugggaugggugacca................................................................	19	0	seq
..................................acugggaugggugacca................................................................	19	0	seq
..................................Ncugggaugggugacca................................................................	1	1	seq
..................................acugggGugggugacca................................................................	1	1	seq
...................................cugggaugggugaccac...............................................................	2	0	seq
...................................cugggaugggugaccacc..............................................................	1	0	seq
```

Provisional ID	:	MA_102488_11501
Score total	:	1863.4
Score for star read(s)	:	3.9
Score for read counts	:	1855.4
Score for mfe	:	2.5
Score for randfold	:	1.6
Score for cons. seed	:	
Total read count	:	3651
Mature read count	:	3274
Loop read count	:	52
Star read count	:	325

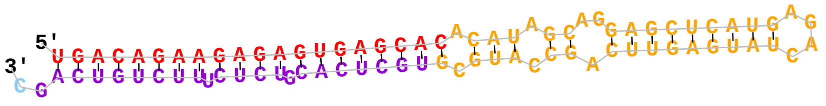

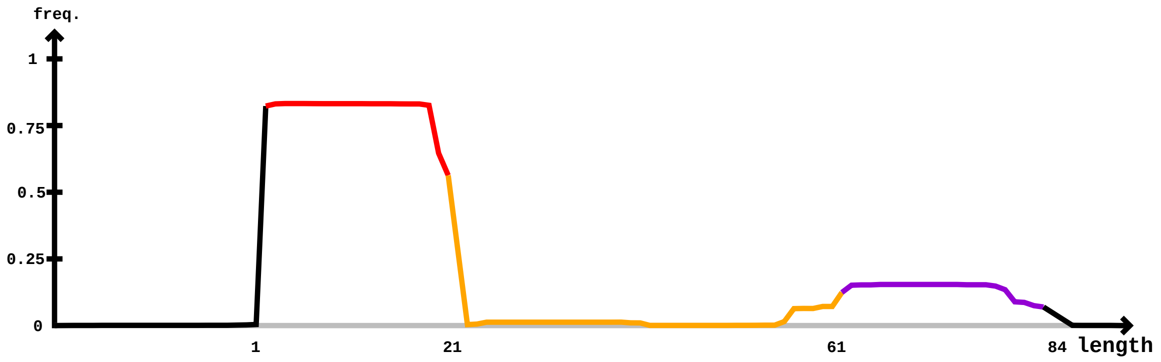

Mature　　　　　　　　　　　　　　　　　　Star

```
5'- cgccucgaucuguugagugcugacagaagagagugagcacacauagcaggagcucaugagacuaugaguucagccaugcgugcucacgucucuuucugucagcggcucau  -3'   obs
    cgccucgaucuguugagugcugacagaagagagugagcacacauagcaggagcucaugagacuaugaguucagccaugcgugcucacgucucuuucugucagcggcucau        exp
    .............(((((((((((((((((((((((((((.(((.((..(((((((((....))))))))).)).))).)))))))).)))).)))))))))).))))).   reads   mm   sample
    cgccucgaucuguugagugcugaca.....................................................................................   1       0    seq
    cgccucgaucuguugagugcugacag....................................................................................   1       0    seq
    ...cucgaucuguugagugc..........................................................................................   1       0    seq
    ..............gagugcugacagaagag...............................................................................   1       0    seq
    .................ugcugacagaagagagu............................................................................   2       0    seq
    .................ugcugacagaagagagug...........................................................................   1       0    seq
    ..................gcugacagaagagagugagca.......................................................................   3       0    seq
    ...................cugacagaagGgagugagca.......................................................................   1       1    seq
    ...................cugacagaagagagugagca.......................................................................   3       0    seq
    ...................cugacagaagagagugagcGc......................................................................   1       1    seq
    ...................cugacagaagagagugagcac......................................................................   1       0    seq
    ....................Ggacagaagagagugag.........................................................................   1       1    seq
    ....................ugacagaagagagugaA.........................................................................   1       1    seq
    ....................ugacagaagagagugag.........................................................................   18      0    seq
    ....................Agacagaagagagugagc........................................................................   1       1    seq
    ....................ugacagaGgagagugagc........................................................................   2       1    seq
    ....................ugGcagaagagagugagc........................................................................   3       1    seq
    ....................ugacagaagagagugaCc........................................................................   2       1    seq
    ....................ugaUagaagagagugagc........................................................................   1       1    seq
    ....................ugacagaagagagugagc........................................................................   607     0    seq
    ....................ugacGgaagagagugagc........................................................................   2       1    seq
    ....................ugacagGagagagugagc........................................................................   1       1    seq
    ....................ugacagaagagaAugagc........................................................................   1       1    seq
    ....................ugacagaagagagugagU........................................................................   63      1    seq
    ....................uCacagaagagagugagc........................................................................   1       1    seq
    ....................ugacagaagaUagugagc........................................................................   1       1    seq
    ....................ugacaAaagagagugagc........................................................................   1       1    seq
    ....................ugacagaagagagugagG........................................................................   1       1    seq
    ....................Ngacagaagagagugagc........................................................................   1       1    seq
    ....................ugacagUagagagugagc........................................................................   1       1    seq
    ....................ugacagaagagagugaUc........................................................................   1       1    seq
    ....................ugaUagaagagagugagca.......................................................................   1       1    seq
    ....................ugacagaagagaguAagca.......................................................................   1       1    seq
```

Mature   Star

```
cgccucgaucuguugagugcugacagaagagagugagcacacauagcaggagcucaugagacuaugaguucagccaugcgugcucacgucucuuucugucagcggcucau
....................ugacagaagagagugagcC.......................................................................  3  1  seq
....................ugacagaagagGgugagca.......................................................................  1  1  seq
....................ugacagaGgagagugagca.......................................................................  2  1  seq
....................ugacagaagagagugagcG.......................................................................  15  1  seq
....................ugacagaagagagugagcU.......................................................................  4  1  seq
....................ugacagaagagagugagca.......................................................................  274  0  seq
....................ugacGgaagagagugagca.......................................................................  1  1  seq
....................ugGcagaagagagugagca.......................................................................  1  1  seq
....................ugacagaagaCagugagca.......................................................................  1  1  seq
....................ugacagaagagagugaAca.......................................................................  1  1  seq
....................ugacagaagagagCgagca.......................................................................  1  1  seq
....................ugacagaagagagugaAcac......................................................................  1  1  seq
....................ugacagaGgagagugagcac......................................................................  6  1  seq
....................ugacUgaagagagugagcac......................................................................  1  1  seq
....................ugacagaagagagugagcac......................................................................  2015  0  seq
....................ugacagUagagagugagcac......................................................................  1  1  seq
....................ugacagaagagagugagcaA......................................................................  15  1  seq
....................ugacaAaagagagugagcac......................................................................  1  1  seq
....................ugaUagaagagagugagcac......................................................................  3  1  seq
....................ugacagaagagagugagcGc......................................................................  10  1  seq
....................ugacagaagagaAugagcac......................................................................  1  1  seq
....................ugacagaagagagCgagcac......................................................................  7  1  seq
....................ugacagaagaAagugagcac......................................................................  1  1  seq
....................ugacagaagagagugagcUc......................................................................  1  1  seq
....................ugacagGagagagugagcac......................................................................  13  1  seq
....................ugacagaagagagugagUac......................................................................  2  1  seq
....................ugacagaagagagugUgcac......................................................................  2  1  seq
....................ugacGgaagagagugagcac......................................................................  9  1  seq
....................ugacagaagGgagugagcac......................................................................  10  1  seq
....................ugacaUaagagagugagcac......................................................................  2  1  seq
....................ugacagaagagaguAagcac......................................................................  2  1  seq
....................ugacagaagagagGgagcac......................................................................  1  1  seq
....................ugacagaagagagugCgcac......................................................................  1  1  seq
....................Ggacagaagagagugagcac......................................................................  1  1  seq
....................Agacagaagagagugagcac......................................................................  2  1  seq
....................ugUcagaagagagugagcac......................................................................  2  1  seq
....................Ngacagaagagagugagcac......................................................................  5  1  seq
....................ugacagaagagGgugagcac......................................................................  3  1  seq
....................Cgacagaagagagugagcac......................................................................  5  1  seq
....................ugacagaaUagagugagcac......................................................................  1  1  seq
....................ugacagaagUgagugagcac......................................................................  1  1  seq
....................ugacagaagagagugagcaG......................................................................  2  1  seq
....................ugacagaagagagugagcCc......................................................................  1  1  seq
....................ugGcagaagagagugagcac......................................................................  10  1  seq
....................ugacagaagagagugagcacC.....................................................................  12  1  seq
....................ugacagaagagagugagGaca.....................................................................  1  1  seq
....................ugacagGagagagugagcaca.....................................................................  1  1  seq
....................ugacagaagagagugagcaca.....................................................................  32  0  seq
....................ugacagaagagagugagcacU.....................................................................  23  1  seq
....................ugacagaagagagugagcCca.....................................................................  1  1  seq
....................ugacagaagagagugagcacG.....................................................................  3  1  seq
....................ugacagaagagagugagcacaA....................................................................  1  1  seq
.....................gacagaagagagugagU........................................................................  2  1  seq
.....................gacagaagagagugagc........................................................................  15  0  seq
.....................gacagaagagagugagca.......................................................................  10  0  seq
.....................gGcagaagagagugagca.......................................................................  1  1  seq
.....................gacagaagagagGgagca.......................................................................  1  1  seq
.....................gacagaagagagugagcac......................................................................  3  0  seq
......................acagaagagagugagca.......................................................................  3  0  seq
......................acagaagagagugagcac......................................................................  1  0  seq
......................acagaagagagugagcaca.....................................................................  1  0  seq
........................................acauagcaggagcucaugag..................................................  1  0  seq
........................................acauagcaggagcucaugagacu...............................................  1  0  seq
.........................................cauagcUggagcucaug....................................................  5  1  seq
.........................................Gauagcaggagcucaug....................................................  6  1  seq
..........................................auagcaggagcucaugU...................................................  1  1  seq
..........................................auagcaggagcucaugUg..................................................  8  1  seq
...........................................uagcaggagcucaugag..................................................  1  0  seq
...........................................uagcaggagcucaugUg..................................................  27  1  seq
.........................................................gagacuaugaguucagccau.................................  1  0  seq
```

Mature   Star

```
cgccucgaucuguugagugcugacagaagagagugagcacacauagcaggagcucaugagacuaugaguucagccaugcgugcucacgucucuuucugucagcggcucau
...............................................................augaguucagccaugcg..............................	1	0	seq
.....................................................................ucagccaugcgugcucacgucucuuucugucagcgg.....	1	0	seq
.......................................................................agccaugcgugcucacgucucC.................	1	1	seq
........................................................................gccaugcgugcucacgucucu.................	1	0	seq
........................................................................gccaugcgugcucacgucucuuucugucagcggcuca.	1	0	seq
..........................................................................caugcgugcucacgucucG.................	1	1	seq
..........................................................................caugcgugcCcacgucucuuuc..............	1	1	seq
..........................................................................caugcgugcucacgucucuuuc..............	13	0	seq
..........................................................................caugcAugcucacgucucuuucu.............	1	1	seq
..........................................................................caugcgugcucCcgucucuuucu.............	1	1	seq
..........................................................................caugcgCgcucacgucucuuucu.............	1	1	seq
..........................................................................caugcgugcucaUgucucuuucu.............	1	1	seq
..........................................................................caugcgugcucacgucucuuucC.............	8	1	seq
..........................................................................caugcgugcucacgucucuuucu.............	24	0	seq
..........................................................................caugUgugcucacgucucuuucu.............	1	1	seq
..........................................................................caugcgugcucacgucucuuucug............	1	0	seq
...........................................................................augcgugcucacgucucu.................	1	0	seq
...........................................................................augcgugcucacgucucuuuc..............	5	0	seq
...........................................................................augcgugcAcacgucucuuucu.............	1	1	seq
...........................................................................augcgugcucacgucucuuucu.............	8	0	seq
...........................................................................augcgugcucacgucucuuucG.............	1	1	seq
...........................................................................augcgugcucacgucucuuucuU............	1	1	seq
...........................................................................augUgugcucacgucucuuucug............	1	1	seq
...........................................................................augcgugcucacgucucuuucAg............	1	1	seq
...........................................................................augcgugUucacgucucuuucug............	2	1	seq
...........................................................................augcUugcucacgucucuuucug............	1	1	seq
...........................................................................augcgugcucacgucucuuucug............	154	0	seq
...........................................................................augcgugcucacgucucuuucCg............	1	1	seq
...........................................................................augcgugcucacgucucuuucuA............	7	1	seq
...........................................................................augcgugcucacgucucuuCcug............	1	1	seq
...........................................................................augcgugcucacgCcucuuucug............	1	1	seq
...........................................................................augGgugcucacgucucuuucug............	1	1	seq
...........................................................................augcgugcucacgAcucuuucug............	2	1	seq
...........................................................................augcgugcucacgucucuuucGg............	1	1	seq
...........................................................................augcgugcucacgucucuuucugA...........	1	1	seq
............................................................................ugcgugcucacgucucuuuc..............	1	0	seq
............................................................................ugcgugcucacgucucuuucug............	2	0	seq
..............................................................................cgugcucacgucucuuucC.............	2	1	seq
..............................................................................cgugcucacgucucuuucu.............	1	0	seq
..............................................................................cgugcucacgucucuuucug............	1	0	seq
..............................................................................cgugcucacgucucuuucuguc..........	23	0	seq
..............................................................................cgugcucacgucucuuucuguU..........	2	1	seq
..............................................................................cgugcucacgucucuuucugucU.........	1	1	seq
................................................................................ugcucacgucucuuucu.............	3	0	seq
................................................................................ugcucacgucucuuucug............	1	0	seq
................................................................................ugcucacgucucuuucugC...........	2	1	seq
................................................................................ugcucacgucucuuucugu...........	1	0	seq
................................................................................ugcucacgucucuuucuguU..........	2	1	seq
................................................................................ugcucacgucucuuucugCc..........	1	1	seq
................................................................................ugcucacgucucuuucuguc..........	11	0	seq
................................................................................ugcucacgucucuuucuguca.........	8	0	seq
................................................................................ugcucacgucCcuuucugucag........	1	1	seq
................................................................................ugcucacgucucuuucugCcag........	2	1	seq
................................................................................ugcucacgucucuuucugucGg........	1	1	seq
................................................................................ugcucacgucucuCucugucag........	1	1	seq
................................................................................ugcucacgucucuuucugucaU........	1	1	seq
................................................................................ugcucacgucucuuucugucaA........	2	1	seq
................................................................................uCcucacgucucuuucugucag........	1	1	seq
................................................................................ugcucacgucucuuucugucag........	141	0	seq
................................................................................ugGucacgucucuuucugucag........	1	1	seq
................................................................................ugcucacgucucuuucugucagU.......	1	1	seq
................................................................................ugcucacgucCcuuucugucagc.......	1	1	seq
................................................................................ugcucacgucucuuucuAucagc.......	1	1	seq
................................................................................ugcucacgucucuuCcugucagc.......	1	1	seq
................................................................................ugcucacgucucuuucugucagA.......	1	1	seq
................................................................................ugcucacgucucuuucugucagc.......	21	0	seq
................................................................................ugcucacgucucuuucugucagAg......	1	1	seq
................................................................................ugcucacgucucuuucugucagcA......	1	1	seq
.................................................................................gcucacgucucuuucugC...........	2	1	seq
.................................................................................gcucacgucucCuucugu...........	1	1	seq
```

```
                    Mature                                                      Star
cgccucgaucuguugagugcugacagaagagagugagcacacauagcaggagcucaugagacuaugaguucagccaugcgugcucacgucucuuucugucagcggcucau
.................................................................................gcucacgucucuuucugu...........    1    0    seq
.................................................................................gcucacgucucuuucugA...........    1    1    seq
.................................................................................gcucacgucucuuucugCc..........    1    1    seq
.................................................................................gcucacgucucuuucuguc..........    7    0    seq
.................................................................................gcucGcgucucuuucuguca.........    1    1    seq
.................................................................................gcucacgucucuuucuguca.........    8    0    seq
.................................................................................gcucacgucucuuucugucag........    38   0    seq
.................................................................................gcucacgucucuuCcugucag........    1    1    seq
.................................................................................gcucacgCcucuuucugucag........    1    1    seq
.................................................................................gcucacgucucuuucugucaA........    2    1    seq
.................................................................................gcucacgucucuuucugucagU.......    2    1    seq
.................................................................................gcucGcgucucuuucugucagc.......    1    1    seq
.................................................................................gcucacgucucuuucugucagc.......    38   0    seq
.................................................................................gcucacgucucuuCcugucagc.......    1    1    seq
.................................................................................gcucacgucucuuucugucagcggcuca.    1    0    seq
..................................................................................cucGcgucucuuucuguc..........    1    1    seq
..................................................................................cucacgucucuuucugucag........    2    0    seq
..................................................................................cucacgucucuuucugucagU.......    1    1    seq
..................................................................................cucacgucucuuucugucagc.......    1    0    seq
....................................................................................cacgucucuuucuguca.........    2    0    seq
....................................................................................cacgucucuuucugucagc.......    3    0    seq
....................................................................................cacgucucuuucugucagU.......    1    1    seq
```

Provisional ID : MA_102607_16329
Score total : 2.2
Score for star read(s) : -1.3
Score for read counts : 0
Score for mfe : 1.9
Score for randfold : 1.6
Score for cons. seed :
Total read count : 2997
Mature read count : 2996
Loop read count : 1
Star read count : 0

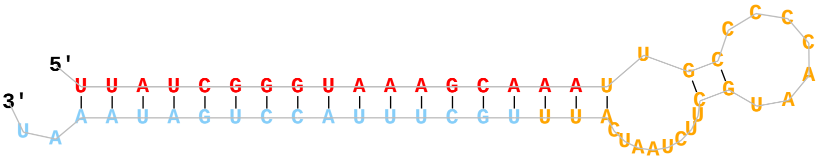

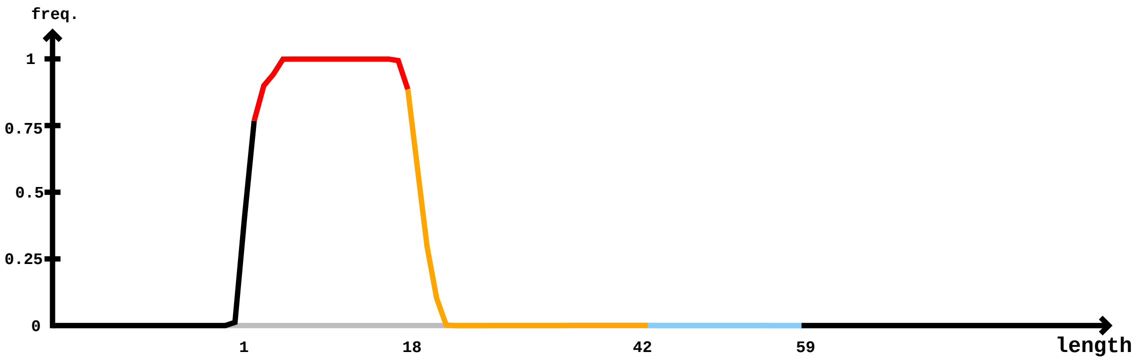

Mature Star

```
5'- gaccaucgauuaaauaauuuaucggguaaagcaaauugcccccaaugcuucuaaucauuugcuuuaccugauaaaaucauuuaaucaaugguucaauuuuacauccuc  -3'  exp
    (((((((.((((((((.((((((((((((((((((((.((.......))........)))))))))))))))))))).)))))))).)))))))..............  reads  mm  sample
    ................Cuuuaucggguaaagca...........................................................................  10     1   seq
    ................Nuuuaucggguaaagca...........................................................................  2      1   seq
    ................auuuaucggguaaagca...........................................................................  1      0   seq
    ................Guuuaucggguaaagca...........................................................................  4      1   seq
    ................Cuuuaucggguaaagcaa..........................................................................  2      1   seq
    ................Nuuuaucggguaaagcaa..........................................................................  2      1   seq
    ................Guuuaucggguaaagcaaa.........................................................................  1      1   seq
    ................Nuuuaucggguaaagcaaa.........................................................................  1      1   seq
    ................Cuuuaucggguaaagcaaa.........................................................................  4      1   seq
    ................auuuaucggguaaagcaaa.........................................................................  1      0   seq
    ................auuuaucggguaaagcaaau........................................................................  1      0   seq
    ................Cuuuaucggguaaagcaaau........................................................................  1      1   seq
    ................auuuaucggguaaagcaaauG.......................................................................  3      1   seq
    ................Uuuuaucggguaaagcaaauu.......................................................................  3      1   seq
    .................uuuaucggguaGagcaa..........................................................................  3      1   seq
    .................uuuaucggguaaagcaa..........................................................................  291    0   seq
    .................uuuaucgggGaaagcaa..........................................................................  1      1   seq
    .................Cuuaucggguaaagcaa..........................................................................  3      1   seq
    .................uuuaucggguGaagcaa..........................................................................  1      1   seq
    .................uuuaucggguaaagcaG..........................................................................  11     1   seq
    .................uuuaucggguaaagcGa..........................................................................  3      1   seq
    .................uuuaucggguaaagcCa..........................................................................  1      1   seq
    .................uuuGucggguaaagcaa..........................................................................  1      1   seq
    .................uuuaucggguaaGgcaa..........................................................................  2      1   seq
    .................Nuuaucggguaaagcaa..........................................................................  1      1   seq
    .................uuuaucggAuaaagcaa..........................................................................  1      1   seq
    .................uuuaucggguaaaUcaaa.........................................................................  1      1   seq
    .................uuAaucggguaaagcaaa.........................................................................  1      1   seq
    .................uCuaucggguaaagcaaa.........................................................................  1      1   seq
    .................Cuuaucggguaaagcaaa.........................................................................  2      1   seq
    .................uuuaucggguaaagcaaa.........................................................................  513    0   seq
    .................uuuUucggguaaagcaaa.........................................................................  1      1   seq
    .................uuuaucggguaaagcaaG.........................................................................  8      1   seq
    .................uuuauAggguaaagcaaa.........................................................................  1      1   seq
```

Mature Star

gaaccaucgauuaaauaauuuaucggguaaagcaaauugcccccaaugcuucuaaucauuugcuuuaccugauaaaucauuuaaucaaugguucaauuuuacauccuc			
..................uuuaucggguaaagcaGa..	1	1	seq
..................uuCaucggguaaagcaaa..	1	1	seq
..................uuuaucggguaaagcGaa..	2	1	seq
..................uuuaucggguaaagcaaU..	2	1	seq
..................uuuaucggguaaGgcaaa..	4	1	seq
..................uuuaucggguGaagcaaa..	1	1	seq
..................uuuaucggguCaagcaaa..	2	1	seq
..................uuuaucggguaGagcaaa..	1	1	seq
..................Cuuaucggguaaagcaaau...	1	1	seq
..................uuuaucggguaaagcaaau...	130	0	seq
..................uuuaCcggguaaagcaaau...	1	1	seq
..................uuuaucggguaGagcaaau...	1	1	seq
..................uuuaucggguaaagcaaaC...	7	1	seq
..................uuuaucggguGaagcaaau...	1	1	seq
..................uuuaucggguaaagcGaau...	1	1	seq
..................uuuaucggguaaagcaaaA...	1	1	seq
..................uuuaucggguaaagcaGau...	1	1	seq
..................uuuaucggguaaGgcaaau...	2	1	seq
..................uuuaucggguaaagcaaCu...	2	1	seq
..................uuuaucggguaaagcaaauu..	5	0	seq
..................uuuaucggguaaagcaaauC..	6	1	seq
..................uuuaucggguaaagcaaauA..	137	1	seq
..................uuuaucggguaaagcaaUuug...	1	1	seq
..................uuuaucggguaaagcaaauGg...	26	1	seq
..................uuuaucggguaaagcaaauGgc..	1	1	seq
...................uuGucggguaaagcaaa..	3	1	seq
...................uCaucggguaaagcaaa..	2	1	seq
...................uuaucggguaaagcaGa..	5	1	seq
...................uuaucggguaGagcaaa..	3	1	seq
...................uuaucggguaaGgcaaa..	2	1	seq
...................uuaucggguaUagcaaa..	1	1	seq
...................uuaCcggguaaagcaaa..	1	1	seq
...................uuaucggguaaagUaaa..	1	1	seq
...................uuaucggguaaagcGaa..	2	1	seq
...................uuUucggguaaagcaaa..	1	1	seq
...................Guaucggguaaagcaaa..	5	1	seq
...................uuaucggguaaagcaaG..	6	1	seq
...................uuaucggguaaagcaaa..	624	0	seq
...................Cuaucggguaaagcaaa..	3	1	seq
...................uuaucggguaaagcaaU..	3	1	seq
...................uuaAcggguaaagcaaa..	1	1	seq
...................uuaucggguaaagcaaaC...	12	1	seq
...................uAaucggguaaagcaaau...	2	1	seq
...................uuaucggguaaagcaaaA...	3	1	seq
...................uuaucggguaaagcaaau...	184	0	seq
...................uuaucgggGaaagcaaau...	1	1	seq
...................Cuaucggguaaagcaaau...	3	1	seq
...................uuaucggguaaagcaaGu...	1	1	seq
...................uuaucggguaaGgcaaau...	2	1	seq
...................uuaucggguaaagcaGau...	1	1	seq
...................Nuaucggguaaagcaaau...	1	1	seq
...................uuaucggguaaagcaaaG...	1	1	seq
...................uuaucggguaaagcaaauA..	156	1	seq
...................uuaucggguaaagcaaauu..	8	0	seq
...................uuaucggguaaagcaaauC..	3	1	seq
...................uuaucggguaaagcaaauGg...	40	1	seq
....................Aaucggguaaagcaaau...	1	1	seq
....................uaucgggCaaagcaaau...	1	1	seq
....................uaucggguaaGgcaaau...	1	1	seq
....................uaCcggguaaagcaaau...	1	1	seq
....................uaucggguaaagcaaau...	172	0	seq
....................uaucggguaaagcUaau...	1	1	seq
....................uaucggguaaagcaaaG...	3	1	seq
....................uaucggguaaagcaaaC...	8	1	seq
....................uaucggguaaagcaGau...	1	1	seq
....................uGucggguaaagcaaau...	3	1	seq
....................uaucggguaaagcaaauA..	146	1	seq
....................uaucggguaaagcaaauu..	5	0	seq
....................uaucggguaaagcaaauC..	5	1	seq
....................uaucggguaaagcaaauGg...	44	1	seq

```
                   Mature                                   Star
gaaccaucgauuaaauaauuuaucggguaaagcaaauugcccccaaugcuucuaaucauuugcuuuaccugauaaaucauuuaaucaaugguucaauuuuacauccuc
....................uaucggguaaagcaaauGgc....................................................................   3     1     seq
.....................aucggguaaagcaaauA......................................................................   100   1     seq
.....................aucggguaaagcaaauC......................................................................   2     1     seq
.....................aucggguaaagcaaauu......................................................................   4     0     seq
.....................aucggguaaagcaaauGg.....................................................................   22    1     seq
......................ucggguaaagcaaaAug.....................................................................   1     1     seq
......................ucggguaaagcaaauuA.....................................................................   1     1     seq
......................ucggguaaagcaaauGg.....................................................................   168   1     seq
.......................cggguaaagcaaauGgc....................................................................   1     1     seq
............................................aauCcuucuaaucauuu...............................................   1     1     seq
....................................................uaaucauuugcuuuaccCgau...................................   1     1     seq
```

Provisional ID : MA_10286645_32656
Score total : 7003.5
Score for star read(s) : 3.9
Score for read counts : 7003.6
Score for mfe : -1.6
Score for randfold : -2.2
Score for cons. seed :
Total read count : 13749
Mature read count : 13746
Loop read count : 0
Star read count : 3

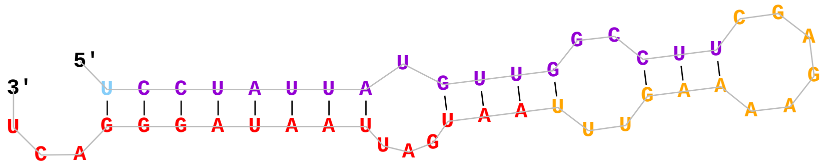

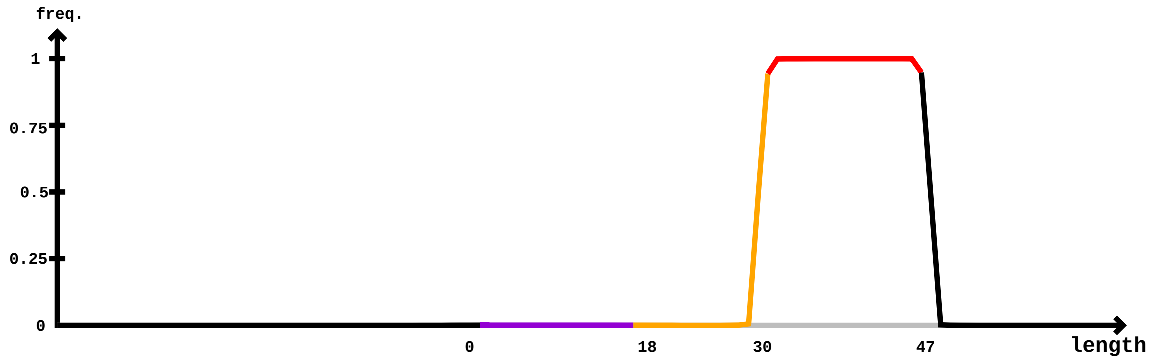

Star Mature

```
5'- augucuuaucgauuauggaaauuaggugugcuagggguaugguccuauuauguuggccuucgagaaaaguuuaaugauuaauagggacuauugagugcccuuccauccc  -3'  obs
    augucuuaucgauuauggaaauuaggugugcuagggguaugguccuauuauguuggccuucgagaaaaguuuaaugauuaauagggacuauugagugcccuuccauccc        exp
    ..............(((((....(((.(..((......((((((((((((.((((..(((......)))..))))...))))).)))))))..))..)))))))))...  reads  mm  sample
    ....................................guCugguccuauuauguuggc....................................................  1   1   seq
    .....................................uCugguccuauuauguu.......................................................  1   1   seq
    .......................................ugguccuauuGuguuggccuucga..............................................  4   1   seq
    ........................................gguccuauuGuguuggccuucga..............................................  1   1   seq
    ..........................................uccuauuauguuggccuu.................................................  1   0   seq
    ...........................................ccuauuauguuggccuu.................................................  1   0   seq
    ...........................................ccuauuCuguuggccuu.................................................  1   1   seq
    ....................................................................guGuaaugauuaauagggacu....................  2   1   seq
    ....................................................................gAuuaaugauuaauagggacu....................  3   1   seq
    .....................................................................uGuaaugauuaauaggga......................  1   1   seq
    .....................................................................Auuaaugauuaauagggacu....................  1   1   seq
    .....................................................................Nuuaaugauuaauagggacu....................  2   1   seq
    .....................................................................uGuaaugauuaauagggacu....................  4   1   seq
    ......................................................................Cuaaugauuaauaggga......................  1   1   seq
    ......................................................................uuaaugauuaauaggga......................  1   0   seq
    ......................................................................Nuaaugauuaauagggac.....................  1   1   seq
    ......................................................................uuaaugauuaauagggac.....................  1   0   seq
    ......................................................................Cuaaugauuaauagggacu....................  4   1   seq
    ......................................................................uuaaugauuaauagggacu....................  22  0   seq
    ......................................................................uuaaugauuaauagggacC....................  1   1   seq
    ......................................................................Nuaaugauuaauagggacu....................  14  1   seq
    ......................................................................uuaaugauuaauaggUacu....................  1   1   seq
    ......................................................................uuaaugauuaauagggacuG...................  7   1   seq
    ......................................................................Nuaaugauuaauagggacua...................  4   1   seq
    ......................................................................Auaaugauuaauagggacua...................  1   1   seq
    ......................................................................uuaaugauuaauagggacuGu..................  4   1   seq
    .......................................................................Naaugauuaauagggac.....................  2   1   seq
    .......................................................................uaaugauuaauaggUac.....................  1   1   seq
    .......................................................................uaaugauuaauagggac.....................  680 0   seq
    .......................................................................uaaugauuaaAagggac.....................  1   1   seq
    .......................................................................uaaugaCuaauagggac.....................  2   1   seq
    .......................................................................uaaugauuaGuagggac.....................  1   1   seq
    .......................................................................uaaugauuaauagggaG.....................  1   1   seq
```

Star **Mature**

```
augucuuaucgauuauggaaauuaggugugcuagggguauggucuauuauguuggccuucgagaaaaguuuaaugauuaauagggacuauugagugcccuuccauccc
.......................................................................uaaugauuaauagggaA.....................   2      1      seq
.......................................................................Gaaugauuaauagggac.....................   1      1      seq
.......................................................................Aaaugauuaauagggac.....................   2      1      seq
.......................................................................uaaugGuuaauagggac.....................   2      1      seq
.......................................................................uaaugauCaauagggac.....................   4      1      seq
.......................................................................uaaugauuaaCagggac.....................   1      1      seq
.......................................................................uaaugauuaaGagggac.....................   1      1      seq
.......................................................................uaGugauuaauagggac.....................   1      1      seq
.......................................................................uaaAgauuaauagggac.....................   1      1      seq
.......................................................................uaaAgauuaauagggacu....................   1      1      seq
.......................................................................Naaugauuaauagggacu....................   9      1      seq
.......................................................................uaaugauuaGuagggacu....................   13     1      seq
.......................................................................uaaugauuaauagggUcu....................   2      1      seq
.......................................................................uaaugauuaauagggacC....................   736    1      seq
.......................................................................uaaugaCuaauagggacu....................   4      1      seq
.......................................................................uaaugauuUauagggacu....................   1      1      seq
.......................................................................uaCugauuaauagggacu....................   1      1      seq
.......................................................................Aaaugauuaauagggacu....................   3      1      seq
.......................................................................uaaugauCaauagggacu....................   6      1      seq
.......................................................................uaaugauuaauagUgacu....................   3      1      seq
.......................................................................uaGugauuaauagggacu....................   18     1      seq
.......................................................................uaaugauAaauagggacu....................   3      1      seq
.......................................................................uaaugCuuaauagggacu....................   2      1      seq
.......................................................................uaUugauuaauagggacu....................   3      1      seq
.......................................................................uaaugauuaauagggaAu....................   1      1      seq
.......................................................................Gaaugauuaauagggacu....................   11     1      seq
.......................................................................uaaugauuaaCagggacu....................   5      1      seq
.......................................................................uaaugauuaUuagggacu....................   1      1      seq
.......................................................................uaaugauuaauUgggacu....................   4      1      seq
.......................................................................uNaugauuaauagggacu....................   1      1      seq
.......................................................................uaauAauuaauagggacu....................   2      1      seq
.......................................................................uaaugauuaauagggCcu....................   3      1      seq
.......................................................................uaaugauuaauagggacA....................   125    1      seq
.......................................................................uaaugauuaauagggacu....................   4484   0      seq
.......................................................................uaaCgauuaauagggacu....................   7      1      seq
.......................................................................uaaugauuaauagggacG....................   165    1      seq
.......................................................................uaaugGuuaauagggacu....................   17     1      seq
.......................................................................uaauUauuaauagggacu....................   1      1      seq
.......................................................................Aaaugauuaauagggacua...................   1      1      seq
.......................................................................uaaugauuaGuagggacua...................   1      1      seq
.......................................................................uaaugGuuaauagggacua...................   2      1      seq
.......................................................................uaaugauuaauagggacua...................   297    0      seq
.......................................................................uaaugauuaauagggacCa...................   7      1      seq
.......................................................................uaauAauuaauagggacua...................   1      1      seq
.......................................................................uaaugauuaauagggacGa...................   1      1      seq
.......................................................................uaaugauuaauagAgacua...................   2      1      seq
.......................................................................uaGugauuaauagggacua...................   2      1      seq
.......................................................................uaaugauuaauagggacuaA..................   1      1      seq
.......................................................................uaaugauuaauagggacuau..................   1      0      seq
........................................................................aCugauuaauagggacu....................   1      1      seq
........................................................................aaugaGuaauagggacu....................   1      1      seq
........................................................................Uaugauuaauagggacu....................   6      1      seq
........................................................................aaugauuaauCgggacu....................   2      1      seq
........................................................................aaugauuaGuagggacu....................   21     1      seq
........................................................................aaugauuaUuagggacu....................   3      1      seq
........................................................................aaugauuaauagggUcu....................   2      1      seq
........................................................................aaugauuaauagggCcu....................   1      1      seq
........................................................................aaugauuaauagggacC....................   817    1      seq
........................................................................aauUauuaauagggacu....................   2      1      seq
........................................................................aaugauuaauagggaAu....................   2      1      seq
........................................................................aaCgauuaauagggacu....................   7      1      seq
........................................................................aaAgauuaauagggacu....................   3      1      seq
........................................................................Caugauuaauagggacu....................   3      1      seq
........................................................................aGugauuaauagggacu....................   32     1      seq
........................................................................aaugauuaauagggacA....................   116    1      seq
........................................................................aaugGuuaauagggacu....................   18     1      seq
........................................................................aaugauuaauaggUacu....................   3      1      seq
........................................................................aaugaCuaauagggacu....................   15     1      seq
........................................................................aaugauuaauUgggacu....................   1      1      seq
........................................................................aUugauuaauagggacu....................   1      1      seq
```

```
                                           Star                        Mature
augucuuaucgauuauggaaauuaggugugcuagggguaugguccuauuauguuggccuucgagaaaaguuuaaugauuaauagggacuauugagugcccuuccauccc
........................................................................aaugauuaCuagggacu....................   1      1      seq
........................................................................aaugauCaauagggacu....................   10     1      seq
........................................................................Naugauuaauagggacu....................   11     1      seq
........................................................................aaugauuaauagggacG....................   169    1      seq
........................................................................aaugauuaauagUgacu....................   3      1      seq
........................................................................aaugauuaauagggacu....................   4753   0      seq
........................................................................aaugauuaauaggCacu....................   1      1      seq
........................................................................aaugauuaaCagggacu....................   5      1      seq
........................................................................aaGgauuaauagggacu....................   3      1      seq
........................................................................aaugGuuaauagggacua...................   1      1      seq
........................................................................aaugauuaauagggacAa...................   2      1      seq
........................................................................aaugauuaauagggacua...................   244    0      seq
........................................................................aaugauuaauagggacGa...................   2      1      seq
........................................................................aaugaCuaauagggacua...................   1      1      seq
........................................................................aaugauuaauagggacCa...................   2      1      seq
........................................................................aaugauuaauagggacuaC..................   1      1      seq
........................................................................aaugauuaauagggacuaGu.................   1      1      seq
........................................................................aaugauuaauagggacuauug................   1      0      seq
.........................................................................augauuaauagggacAa...................   1      1      seq
.........................................................................augaCuaauagggacua...................   1      1      seq
.........................................................................augauuaauagggacCa...................   8      1      seq
.........................................................................augauuaauagggacua...................   720    0      seq
.........................................................................augauuaGuagggacua...................   1      1      seq
.........................................................................augauuaauagggUcua...................   1      1      seq
.........................................................................Gugauuaauagggacua...................   3      1      seq
.........................................................................Uugauuaauagggacua...................   2      1      seq
.........................................................................Cugauuaauagggacua...................   1      1      seq
.........................................................................augauuaauagggGcua...................   3      1      seq
.........................................................................augauuaauagggaGua...................   1      1      seq
.........................................................................aCgauuaauagggacua...................   1      1      seq
.........................................................................augauuaauCgggacua...................   1      1      seq
.........................................................................augauuaauagUgacua...................   1      1      seq
.........................................................................augauuaauagggacGa...................   4      1      seq
.........................................................................augauuaauaggAacua...................   2      1      seq
.........................................................................augauuaauagggacuau..................   3      0      seq
.........................................................................augauuaauagggacuaC..................   1      1      seq
.........................................................................augauuaauagggacuaA..................   1      1      seq
.........................................................................augauuaauagggacuUuu.................   1      1      seq
..........................................................................ugauuaauagggacuau..................   3      0      seq
..........................................................................ugauuaauagggacuaC..................   1      1      seq
..........................................................................ugauuaauagggacuauA.................   1      1      seq
..........................................................................ugauuaauagggacuauG.................   1      1      seq
............................................................................auuaauagggacuauCga...............   1      1      seq
.............................................................................uuaauagggacuauCga...............   1      1      seq
```

Provisional ID	:	MA_10292869_33628
Score total	:	1
Score for star read(s)	:	-1.3
Score for read counts	:	0
Score for mfe	:	0.7
Score for randfold	:	1.6
Score for cons. seed	:	
Total read count	:	2451
Mature read count	:	2451
Loop read count	:	0
Star read count	:	0

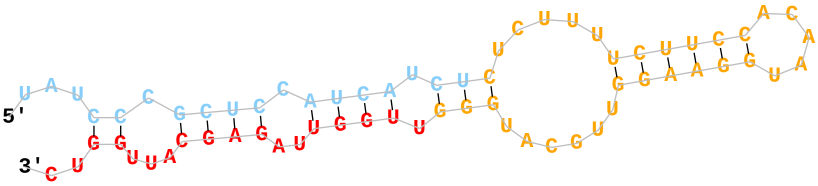

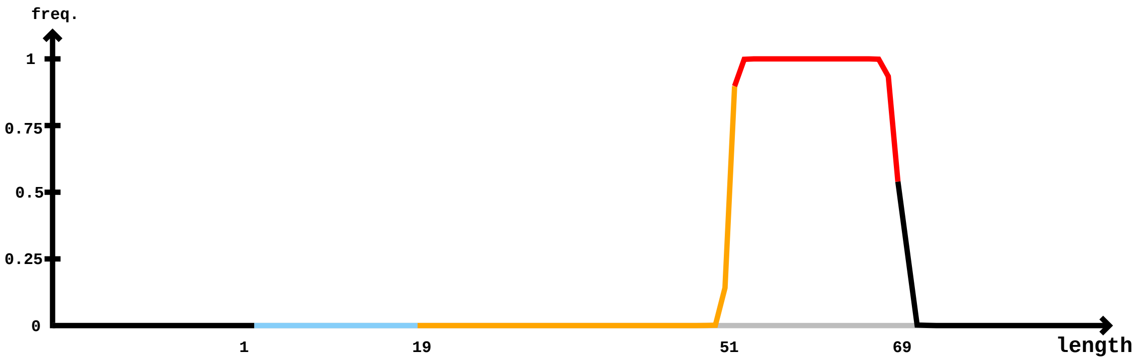

Star **Mature**

```
5'- gagcaacgucuugaaaggauaucccgcuccaucaucucucuuuucuuccacaauggaagguugcauggguugguuagagcauugguccucaagcaaaacuagcucgag   -3'   exp
    ((((...((.(((..(((((.....((((.((((.(((.....((((((.....))))))......))).))))..))))....)))))....))).))..))))...   reads   mm   sample
    ..................................................................Cgguugguuagagcauug........................   1   1   seq
    ...................................................................gguugguuagagcauug........................   2   0   seq
    ....................................................................guuAguuagagcauugg.......................   1   1   seq
    ....................................................................gCugguuagagcauugg.......................   1   1   seq
    ....................................................................guugguuagagcauugA.......................   7   1   seq
    ....................................................................guugguuagagcauGgg.......................   1   1   seq
    ....................................................................guCgguuagagcauugg.......................   1   1   seq
    ....................................................................guugguuagagcauugg.......................   141   0   seq
    ....................................................................guugguuagagcauugU.......................   1   1   seq
    ....................................................................guugguuagagcaCugg.......................   1   1   seq
    ....................................................................guugguuagUgcauugg.......................   1   1   seq
    ....................................................................guugguuagagcauugC.......................   3   1   seq
    ....................................................................guugguCagagcauuggu......................   1   1   seq
    ....................................................................guugguuGgagcauuggu......................   1   1   seq
    ....................................................................guugguuagagcauuggG......................   2   1   seq
    ....................................................................guugguuagGgcauuggu......................   1   1   seq
    ....................................................................guugguuagagcauuggA......................   2   1   seq
    ....................................................................gCugguuagagcauuggu......................   1   1   seq
    ....................................................................guugguuagagcauuggu......................   82   0   seq
    ....................................................................guuggCuagagcauuggu......................   1   1   seq
    ....................................................................guugguuagagcauugguc.....................   87   0   seq
    ....................................................................guugguuagagcauugguA.....................   2   1   seq
    ....................................................................guugguuagGgcauugguc.....................   1   1   seq
    ....................................................................guugguuagagcUuugguc.....................   1   1   seq
    ....................................................................guugguuagagcauuggucG....................   2   1   seq
    ....................................................................guugguuagagcauugguccc...................   1   0   seq
    ....................................................................guugguuagagcauugguccu...................   1   0   seq
    .....................................................................uugguuagagcauugAu......................   2   1   seq
    .....................................................................Cugguuagagcauuggu......................   3   1   seq
    .....................................................................uugguuagagcauugUu......................   1   1   seq
    .....................................................................uAgguuagagcauuggu......................   1   1   seq
    .....................................................................uugguuagagcauugCu......................   1   1   seq
    .....................................................................uugguuGgagcauuggu......................   2   1   seq
    .....................................................................uuAguuagagcauuggu......................   1   1   seq
```

Star Mature

```
gagcaacgucuugaaaggauaucccgcuccaucaucucucuuuucuuccacaauggaagguugcauggguugguuagagcauugguccucaagcaaaacuagcucgag
.....................................................................uugUuuagagcauuggu......................	1	1	seq
.....................................................................uugguuagagcauuggA......................	10	1	seq
.....................................................................uugguuagagcauuggG......................	26	1	seq
.....................................................................Nugguuagagcauuggu......................	1	1	seq
.....................................................................uugguuagagcaCuggu......................	2	1	seq
.....................................................................uugguCagagcauuggu......................	1	1	seq
.....................................................................uugguuagagcauAggu......................	1	1	seq
.....................................................................uugguuagaAcauuggu......................	1	1	seq
.....................................................................Gugguuagagcauuggu......................	1	1	seq
.....................................................................uugguuagagcauuggu......................	814	0	seq
.....................................................................uugguuagGgcauuggu......................	3	1	seq
.....................................................................uCgguuagagcauuggu......................	2	1	seq
.....................................................................uugguuagagUauuggu......................	1	1	seq
.....................................................................uugguAagagcauuggu......................	2	1	seq
.....................................................................uugguuagagcauuggGc.....................	9	1	seq
.....................................................................uugguuagGgcauugguc.....................	3	1	seq
.....................................................................uAgguuagagcauugguc.....................	1	1	seq
.....................................................................uuggCuagagcauugguc.....................	2	1	seq
.....................................................................uCgguuagagcauugguc.....................	2	1	seq
.....................................................................uugguuagagAauugguc.....................	1	1	seq
.....................................................................Cugguuagagcauugguc.....................	1	1	seq
.....................................................................uugguuagagcaCugguc.....................	3	1	seq
.....................................................................uuggGuagagcauugguc.....................	1	1	seq
.....................................................................uugguuagagcauugguG.....................	2	1	seq
.....................................................................uugguuaAagcauugguc.....................	1	1	seq
.....................................................................Gugguuagagcauugguc.....................	3	1	seq
.....................................................................uugguuagagUauugguc.....................	1	1	seq
.....................................................................uugguuagagcauuggAc.....................	3	1	seq
.....................................................................uugguuagagcauugAuc.....................	1	1	seq
.....................................................................uugguuGgagcauugguc.....................	1	1	seq
.....................................................................uuAguuagagcauugguc.....................	1	1	seq
.....................................................................uugguuagagcauuggCc.....................	10	1	seq
.....................................................................uugguuagagcauugguA.....................	17	1	seq
.....................................................................uugguuagagcauCgguc.....................	2	1	seq
.....................................................................Nugguuagagcauugguc.....................	1	1	seq
.....................................................................uugguuagagcauugguc.....................	841	0	seq
.....................................................................uugguuagagcUuugguc.....................	3	1	seq
.....................................................................uugguuagUgcauugguc.....................	1	1	seq
.....................................................................uugguCagagcauugguc.....................	1	1	seq
.....................................................................Augguuagagcauugguc.....................	1	1	seq
.....................................................................uugguuagagcauuggucA....................	8	1	seq
.....................................................................uugguuagagcauuggCcc....................	1	1	seq
.....................................................................Gugguuagagcauugguccc...................	1	1	seq
.....................................................................uugguuagagcauuggucc....................	37	0	seq
.....................................................................uugguuagagcauuggucG....................	14	1	seq
.....................................................................uugguuagagcauugguccu...................	2	0	seq
.....................................................................uugguuagagcauugguccuA..................	1	1	seq
......................................................................uUguuagagcauugguc.....................	1	1	seq
......................................................................ugguuagagcauugguA.....................	8	1	seq
......................................................................ugguuagagcauugguc.....................	213	0	seq
......................................................................ugguuaAagcauugguc.....................	1	1	seq
......................................................................ugguuagagcauGgguc.....................	1	1	seq
......................................................................ugguuagagcauuggCc.....................	2	1	seq
......................................................................ugguuaUagcauugguc.....................	1	1	seq
......................................................................ugguCagagcauugguc.....................	2	1	seq
......................................................................uggCuagagcauugguc.....................	2	1	seq
......................................................................ugguuagagcUuugguc.....................	2	1	seq
......................................................................ugguuagagcauCgguc.....................	1	1	seq
......................................................................ugguuGgagcauugguc.....................	1	1	seq
......................................................................ugguuagagcauuggucc....................	7	0	seq
......................................................................ugguuagagcauuggucG....................	4	1	seq
......................................................................ugguuagagcauugguccuA..................	1	1	seq
.......................................................................gguuagagcauuggucG....................	1	1	seq
.......................................................................gguuagagcauuggucc....................	3	0	seq
```

Provisional ID	:	MA_102951_19530
Score total	:	1.8
Score for star read(s)	:	-1.3
Score for read counts	:	0
Score for mfe	:	1.5
Score for randfold	:	1.6
Score for cons. seed	:	
Total read count	:	4750
Mature read count	:	4750
Loop read count	:	0
Star read count	:	0

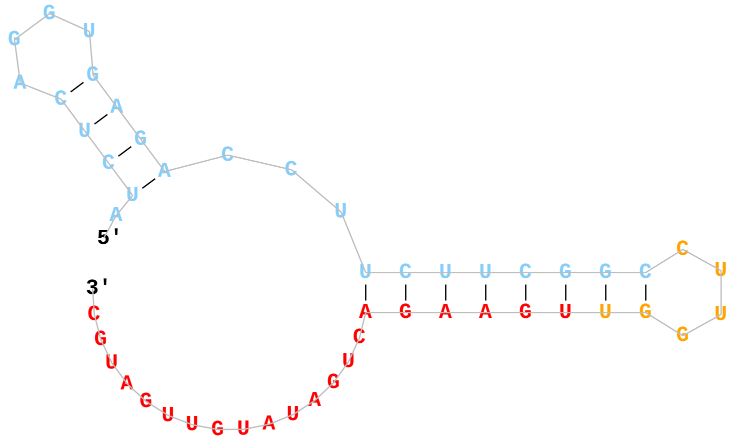

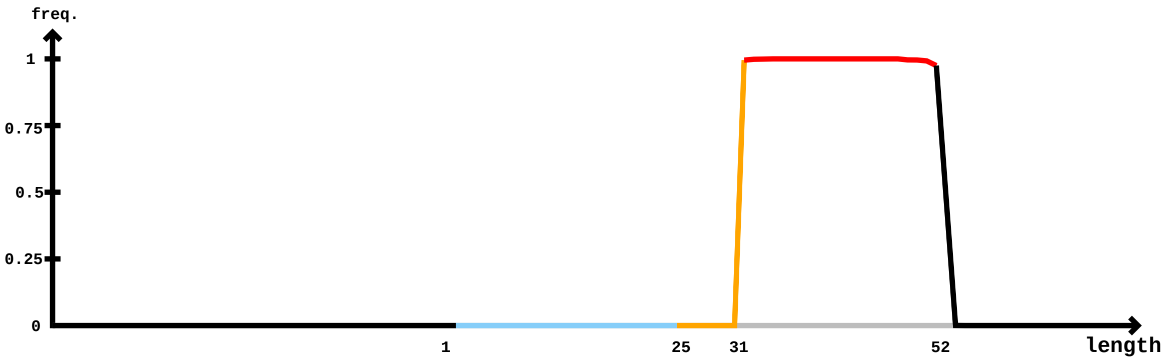

Star Mature

```
5'- ccuugccacaagaccaaauggucucuaugauccucauugcaucucaggugagaccuucuucggccuuggguugaagacugauauguugaugcuuugggguuuuuguugguug   -3'    exp
    ....((((((((((....)))).......(((((..(((((.((.((.((....((((((((....)))))))))).)).))..)))))..)))))...))).))))..   reads      mm        sample
    ......................................................................ugaagacugGuauguug........................   18         1         seq
    ......................................................................ugaagacugGuauguuga.......................   2          1         seq
    ......................................................................ugaagacugGuauguugau......................   15         1         seq
    ......................................................................ugaagacugGuauguugaug.....................   78         1         seq
    ......................................................................ugaagacugUuauguugaugc....................   2          1         seq
    ......................................................................ugaagacugauauguugaugc....................   4          0         seq
    ......................................................................ugaagacugauauguugaugU....................   2          1         seq
    ......................................................................ugaagacugGuauguugaugc....................   4594       1         seq
    ......................................................................ugaagacugGuauguugaugcu...................   11         1         seq
    ......................................................................ugaagacugGuauguugaugcuu..................   2          1         seq
    .......................................................................gaagacugGuauguugau......................   1          1         seq
    .......................................................................gaagacugGuauguugaug.....................   4          1         seq
    .......................................................................gaagacugGuauguugaugc....................   9          1         seq
    .......................................................................gaagacugGuauguugaugcu...................   1          1         seq
    ........................................................................aagacugGuauguugaug.....................   1          1         seq
    ........................................................................aagacugGuauguugaugc....................   1          1         seq
    ........................................................................aagacugGuauguugaugcu...................   1          1         seq
    .........................................................................agacugGuauguugaugc....................   4          1         seq
```

Provisional ID : MA_103350_6302
Score total : 0.4
Score for star read(s) : -1.3
Score for read counts : 0
Score for mfe : 0.1
Score for randfold : 1.6
Score for cons. seed :
Total read count : 88
Mature read count : 88
Loop read count : 0
Star read count : 0

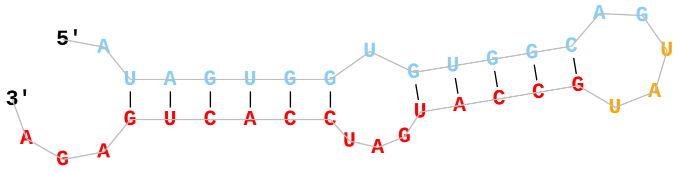

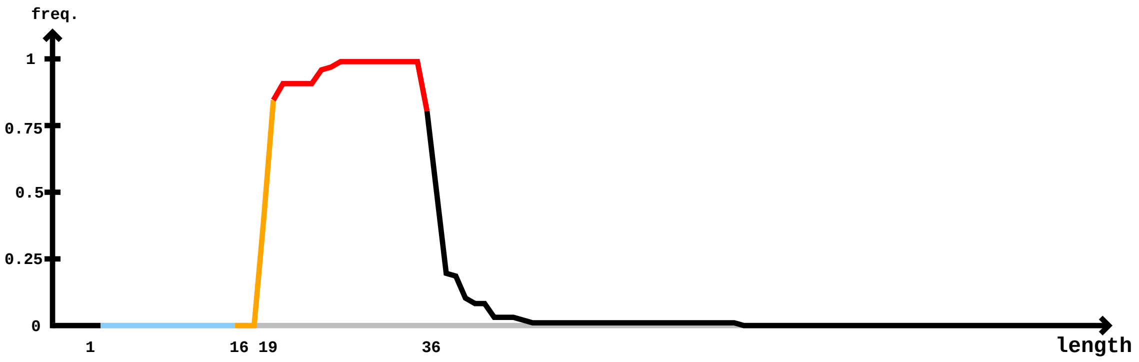

Star Mature

```
5'- aacauagugguguggcaguaugccaugauccacugagauucauaccuuugucaucuggauggaaacuucaaacauuuuuuccauccauuaaucaaauuuaguuccauc   -3'   exp
    ....((((((.(((((.....)))))...))))))...........((((.....((((((((((...........))))))))))......))))............   reads   mm   sample
    ....................ugccaGgauccacugag.......................................................................   1       1    seq
    ....................ugccaugauccacugag.......................................................................   13      0    seq
    ....................ugccaugauccGcugag.......................................................................   1       1    seq
    ....................Ngccaugauccacugag.......................................................................   1       1    seq
    ....................ugccaAgauccacugag.......................................................................   2       1    seq
    ....................ugccaAgauccacugaga......................................................................   1       1    seq
    ....................ugccaugauccacugaga......................................................................   14      0    seq
    ....................ugccaGgauccacugaga......................................................................   1       1    seq
    ....................ugccaugauccacugagau.....................................................................   3       0    seq
    ....................ugccaugauccacugagaA.....................................................................   1       1    seq
    ....................ugccaugauccacugagauuc...................................................................   1       0    seq
    .....................gccGugauccacugaga......................................................................   1       1    seq
    .....................gccaGgauccacugaga......................................................................   6       1    seq
    .....................gccaugauccGcugaga......................................................................   1       1    seq
    .....................gccaugauccacugaga......................................................................   24      0    seq
    .....................gccaugauccacugagG......................................................................   1       1    seq
    .....................gccaAgauccacugaga......................................................................   2       1    seq
    .....................gccaugauccacugagau.....................................................................   1       0    seq
    .....................gccaugauccacugagauu....................................................................   1       0    seq
    .....................gccaugauccacugagauuc...................................................................   4       0    seq
    .....................gccaugauccacugagauuca..................................................................   2       0    seq
    ......................ccaugauccacugagau.....................................................................   3       0    seq
    ......................ccaugauccacugagauuc...................................................................   3       0    seq
    ..........................gauccacugagauucaGa................................................................   5       1    seq
    ...........................auccacugagauucaGaccu.............................................................   1       1    seq
    ............................uccacugagauucaGacc...............................................................   1       1    seq
    ............................uccacugagauucaGaccuu.............................................................   1       1    seq
    ..............................................uuugucaucuggauggaaacuuca......................................   1       0    seq
```

Provisional ID	:	MA_10339386_8889
Score total	:	645.4
Score for star read(s)	:	3.9
Score for read counts	:	640
Score for mfe	:	0
Score for randfold	:	1.6
Score for cons. seed	:	
Total read count	:	1267
Mature read count	:	649
Loop read count	:	0
Star read count	:	618

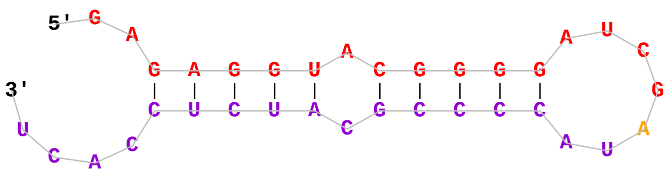

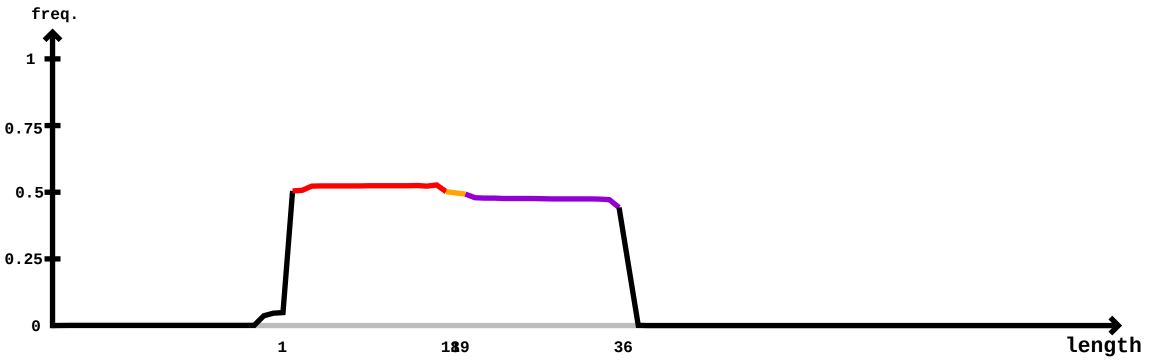

Mature **Star**

```
5'- gguagagcgcucgcuuagcaugugagagguacggggaucgauaccccgcaucuccacuuucaaaacagaccuuuuaauauauuugacauaaaaagucauaaaaaaguuau  -3'   obs
    gguagagcgcucgcuuagcaugugagagguacggggaucgauaccccgcaucuccacuuucaaaacagaccuuuuaauauauuugacauaaaaagucauaaaaaaguuau         exp
    .((.((((....)))).))..(((.(((((.(((((.......))))).))))))))..........(((.(((((.......((((.......))))))))).)))..
```

	reads	mm	sample
gguagagcgcucgcuuagca...	1	0	seq
....................Ngugagagguacgggga..	1	1	seq
....................ugugagagguacgggga..	10	0	seq
....................ugugagagguacggggG..	1	1	seq
....................ugugagaggCacggggauc..	1	1	seq
....................ugugagagguacggAgauc..	1	1	seq
....................ugAgagagguacggggauc..	2	1	seq
....................ugugagagguacggggauA..	1	1	seq
....................ugugagagguacggggauc..	21	0	seq
....................ugugagagguacggggauU..	1	1	seq
....................ugugagagguacggggaucg...	8	0	seq
....................ugugagagguacggggaucA...	1	1	seq
....................ugAgagagguacggggaucg...	1	1	seq
.....................gugagagguacggggaAc..	1	1	seq
.....................gAgagagguacggggauc..	1	1	seq
.....................gugagagguacggggauA..	1	1	seq
.....................gugagagguacggggauc..	7	0	seq
.....................gugagagguacggggaucg...	3	0	seq
......................Agagagguacggggauc..	1	1	seq
......................ugagagguacggAgauc..	1	1	seq
......................Ngagagguacggggauc..	1	1	seq
.......................gaAagguacggggaucg...	1	1	seq
.......................gagagguacggggaucg...	575	0	seq
.......................gagUgguacggggaucg...	1	1	seq
.......................gaUagguacggggaucg...	1	1	seq
.......................gagagguacAgggaucg...	2	1	seq
.......................gCgagguacggggaucg...	1	1	seq
.......................gGgagguacggggaucg...	3	1	seq
.......................gagagguacggggaCcg...	4	1	seq
.......................gagagguacggggauAg...	2	1	seq
.......................gagagguacggggaGcg...	2	1	seq
.......................gagagguacggggaAcg...	1	1	seq
.......................Nagagguacggggaucg...	1	1	seq

Mature **Star**

```
gguagagcgcucgcuuagcaugugagagguacggggaucgauaccccgcaucuccacuuucaaaacagaccuuuuaauauauuugacauaaaaagucauaaaaaguuau
.......................gagagguacggggCucg.....................................................................  2    1    seq
.......................gagagguacggggaucU.....................................................................  6    1    seq
.......................Uagagguacggggaucg.....................................................................  2    1    seq
.......................gagagguGcggggaucg.....................................................................  1    1    seq
.......................gagaggGacggggaucg.....................................................................  2    1    seq
.......................gagagguacggggaucgC....................................................................  1    1    seq
.......................gagagguacggggaucgUu...................................................................  1    1    seq
........................agagguacggggaucgU....................................................................  1    1    seq
........................agagguacggggaucgC....................................................................  1    1    seq
........................agagguacggggaucgaG...................................................................  1    1    seq
.........................gagguacggggaucgau...................................................................  7    0    seq
.........................gagguacggggaucgUu...................................................................  3    1    seq
.........................gagguacggggaucgCu...................................................................  2    1    seq
.........................gagguacggggaucgaG...................................................................  2    1    seq
.........................gagguacggggaucgGu...................................................................  2    1    seq
.........................gagguacggggaucgaua..................................................................  3    0    seq
.........................gagguacggggaucgauacc................................................................  1    0    seq
.........................gagguacggggaucgGuaccccgca...........................................................  1    1    seq
..........................agguacggggaucgUuacc................................................................  1    1    seq
...............................cggggaucgGuaccccgc............................................................  1    1    seq
....................................aucgCuaccccgcaucuccac....................................................  1    1    seq
.....................................ucgGuaccccgcaucucc......................................................  1    1    seq
.....................................ucgauaccccgcaucuccG.....................................................  1    1    seq
.....................................ucgGuaccccgcaucucca.....................................................  1    1    seq
.....................................ucgauaccccgcaucuccac....................................................  1    0    seq
.....................................ucgauaccccgcaucuccacu...................................................  5    0    seq
......................................cgUuaccccgcaucuccac....................................................  1    1    seq
......................................cgauaccccgcaucuccac....................................................  2    0    seq
......................................cgGuaccccgcaucuccacu...................................................  1    1    seq
......................................cgauaccccgcaucuccacu...................................................  2    0    seq
.......................................gauaccccgcaucucca.....................................................  1    0    seq
.......................................gauaccccgcaucuccac....................................................  3    0    seq
.......................................gauaccccgcaucuccacu...................................................  2    0    seq
........................................Cuaccccgcaucuccac....................................................  2    1    seq
........................................auGccccgcaucuccac....................................................  1    1    seq
........................................aAaccccgcaucuccac....................................................  1    1    seq
........................................auaccccgcaucuccGc....................................................  1    1    seq
........................................Guaccccgcaucuccac....................................................  4    1    seq
........................................auaccccgcaucuccac....................................................  21   0    seq
........................................auaccccgcauUuccac....................................................  1    1    seq
........................................Uuaccccgcaucuccacu...................................................  15   1    seq
........................................Cuaccccgcaucuccacu...................................................  2    1    seq
........................................Guaccccgcaucuccacu...................................................  2    1    seq
........................................auaccccgcaucuccacu...................................................  17   0    seq
.........................................uaccccgcaucCccacu...................................................  1    1    seq
.........................................uGccccgcaucuccacu...................................................  5    1    seq
.........................................uaccccgcaucuccCcu...................................................  4    1    seq
.........................................uaccccgcaucuccGcu...................................................  2    1    seq
.........................................uaUcccgcaucuccacu...................................................  1    1    seq
.........................................uaccccgcaucuccacG...................................................  12   1    seq
.........................................uaccccgcaAcuccacu...................................................  1    1    seq
.........................................uaccccgcaucuccUcu...................................................  1    1    seq
.........................................Gaccccgcaucuccacu...................................................  1    1    seq
.........................................Aaccccgcaucuccacu...................................................  3    1    seq
.........................................uaccccgcaucuccacu...................................................  499  0    seq
.........................................uaAcccgcaucuccacu...................................................  1    1    seq
.........................................uaccccgcaucuUcacu...................................................  1    1    seq
.........................................Caccccgcaucuccacu...................................................  7    1    seq
.........................................uaccccgcUucuccacu...................................................  1    1    seq
.........................................uacccAgcaucuccacu...................................................  1    1    seq
.........................................Naccccgcaucuccacu...................................................  1    1    seq
.........................................uaccccgcaucuccacuu..................................................  1    0    seq
.........................................uaccccgcaucuccacuC..................................................  1    1    seq
...........................................ccccgcaucuccacuCu.................................................  1    1    seq
```

Provisional ID	:	MA_103422_15052
Score total	:	1.6
Score for star read(s)	:	-1.3
Score for read counts	:	0
Score for mfe	:	1.3
Score for randfold	:	1.6
Score for cons. seed	:	
Total read count	:	2835
Mature read count	:	2835
Loop read count	:	0
Star read count	:	0

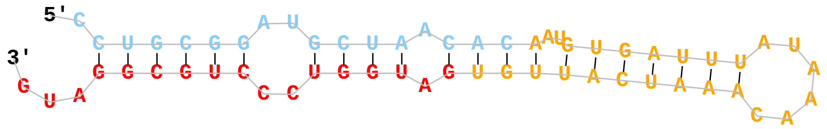

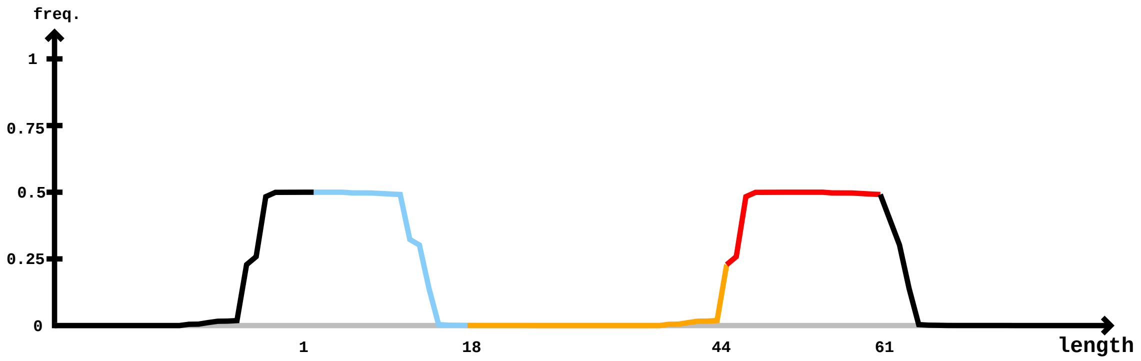


```
                              Star                                    Mature
5'- aauaaaacaaaucauugugauggucccugcggaugcuaacacaaugugauuuauaaacaaaucauugugauggucccugcggaugcuaacacaaugugauuuauaccc   -3'    exp
    .(((((.((...(((((((.((((..((((((..((((.((((..(((((((......))))))))))).)))).))))))..)))).))))))))).)))))....    reads   mm    sample
    ............cauugugauggucccAg...............................................................................    2       1     seq
    ............cauugugauggucccGg...............................................................................    2       1     seq
    ............cauugugauggucccug...............................................................................    26      0     seq
    ............cauugugauggucccugc..............................................................................    2       0     seq
    ............cauugugauggucccugcgg............................................................................    10      0     seq
    ............cauugugauggucccugcggau..........................................................................    2       0     seq
    ............cauugugauggucccugcggaug.........................................................................    6       0     seq
    ............cauugugauggucccugcggaugc........................................................................    2       0     seq
    ............cauugugauggucccugcggaugcu.......................................................................    4       0     seq
    .............auugugauggucccugcgg............................................................................    2       0     seq
    .............auugugauggucccugcgga...........................................................................    2       0     seq
    .............auugGgauggucccugcgga...........................................................................    2       1     seq
    ..............uugugauggucccugcg.............................................................................    2       0     seq
    ..............uugugauggucccugcgg............................................................................    8       0     seq
    ..............uugugauggucccugcgga...........................................................................    8       0     seq
    ..............uugugauggucccugcggau..........................................................................    6       0     seq
    ..............uugugauggucccugcggaC..........................................................................    4       1     seq
    ..............uugGgauggucccugcggau..........................................................................    2       1     seq
    ..............uugugauggucccugcggaug.........................................................................    18      0     seq
    ..............uugugauggucccugcggauA.........................................................................    4       1     seq
    ..............uugugauggucccugcggaugc........................................................................    4       0     seq
    ..............uugugauggucccugcggaugcu.......................................................................    10      0     seq
    ..............uugugauggucccugcggaugcuU......................................................................    2       1     seq
    ..............uugugauggucccugcggaugcua......................................................................    2       0     seq
    ...............ugugauggucccugcgg............................................................................    2       0     seq
    ...............ugugauggucccugcgga...........................................................................    6       0     seq
    ...............ugGgauggucccugcgga...........................................................................    2       1     seq
    ...............ugugauggucccugcggaC..........................................................................    2       1     seq
    ...............ugGgauggucccugcggaug.........................................................................    8       1     seq
    ...............ugugauggucccugcggauA.........................................................................    2       1     seq
    ...............ugugauggucccugcggaug.........................................................................    16      0     seq
    ...............ugugauggucccugcggaugc........................................................................    8       0     seq
    ...............ugugauggucccugcggaugcu.......................................................................    4       0     seq
    ...............ugAgauggucccugcggaugcu.......................................................................    2       1     seq
```

```
                         Star                                          Mature
aauaaaacaaaucauugugauggucccugcggaugcuaacacaaugugauuuauaaacaaaucauugugauggucccugcggaugcuaacacaaugugauuuauaccc
...............ugugauggucccugcggaugcua......................................................................   6      0    seq
................gugauggucccugcgga...........................................................................   4      0    seq
................gugauggucccugcggaug.........................................................................   2      0    seq
.................Ggauggucccugcggau..........................................................................   2      1    seq
.................ugauggucccugcggau..........................................................................   2      0    seq
.................Ngauggucccugcggaug.........................................................................   2      1    seq
.................Ggauggucccugcggaug.........................................................................   4      1    seq
.................ugauggucccugcggaug.........................................................................   8      0    seq
.................Ngauggucccugcggaugcu.......................................................................   2      1    seq
.................Agauggucccugcggaugcu.......................................................................   2      1    seq
..................gauggucccugcggGug.........................................................................   2      1    seq
..................gauggucccAgcggaug.........................................................................   2      1    seq
..................gauggCcccugcggaug.........................................................................   8      1    seq
..................Nauggucccugcggaug.........................................................................   4      1    seq
..................gauggucccugcggaug.........................................................................   1756   0    seq
..................gaAggucccugcggaug.........................................................................   2      1    seq
..................gauggucccugcggaGg.........................................................................   10     1    seq
..................Cauggucccugcggaug.........................................................................   4      1    seq
..................Aauggucccugcggaug.........................................................................   2      1    seq
..................gauggAcccugcggaug.........................................................................   2      1    seq
..................gauggucccGgcggaug.........................................................................   4      1    seq
..................gauggucccugcggauA.........................................................................   86     1    seq
..................gGuggucccugcggaug.........................................................................   10     1    seq
..................gaugguccGugcggaug.........................................................................   2      1    seq
..................gauAgucccugcggaug.........................................................................   2      1    seq
..................gauggucccugcggauC.........................................................................   10     1    seq
..................gauggucccugcggCug.........................................................................   4      1    seq
..................gaAggucccugcggaugc........................................................................   2      1    seq
..................gauggucccugcggaugc........................................................................   158    0    seq
..................gauggucccugcgCaugcu.......................................................................   2      1    seq
..................gGuggucccugcggaugcu.......................................................................   2      1    seq
..................gauggucccugcggauAcu.......................................................................   2      1    seq
..................gauggucccugcggaugcu.......................................................................   246    0    seq
..................gauggucccugcggaugcG.......................................................................   18     1    seq
..................gauggucccugcggaugcA.......................................................................   14     1    seq
..................gauggucccugcggGugcu.......................................................................   2      1    seq
..................gauggucccugcggaugcGa......................................................................   2      1    seq
..................gauggucUcugcggaugcua......................................................................   2      1    seq
..................gauggucccugcggaugcua......................................................................   80     0    seq
..................gauggucccugcggaugcuU......................................................................   18     1    seq
..................gauggucccugcgAaugcua......................................................................   2      1    seq
..................gauggucccugcggaugcuCa.....................................................................   2      1    seq
..................gauggucccugcggaugcuUaca...................................................................   2      1    seq
...................auggucccugcggaugc........................................................................   68     0    seq
...................augguUccugcggaugc........................................................................   2      1    seq
...................auggucccugcggaugcu.......................................................................   98     0    seq
...................auggucccugcggaugcG.......................................................................   12     1    seq
...................aCggucccugcggaugcu.......................................................................   2      1    seq
...................auggucccugcggCugcu.......................................................................   4      1    seq
...................auggucccugcggaugcCa......................................................................   4      1    seq
...................auggucccugcggaugcua......................................................................   114    0    seq
...................Nuggucccugcggaugcua......................................................................   2      1    seq
...................auggucccugcggaugcuU......................................................................   38     1    seq
...................auggucccugcggaugcuaa.....................................................................   4      0    seq
...................auggucccugcggaugcuUac....................................................................   2      1    seq
...................auggucccugcggaugcuaaca...................................................................   2      0    seq
...................auggucccugcggaugcuaacC...................................................................   2      1    seq
....................uggucccugcggGugcu.......................................................................   16     1    seq
....................uggucccugcUgaugcu.......................................................................   2      1    seq
....................uggucccugcggaugcu.......................................................................   1290   0    seq
....................uggucccugcggCugcu.......................................................................   10     1    seq
....................Cggucccugcggaugcu.......................................................................   8      1    seq
....................uggucccugcggaugcG.......................................................................   74     1    seq
....................uAgucccugcggaugcu.......................................................................   2      1    seq
....................Aggucccugcggaugcu.......................................................................   2      1    seq
....................Nggucccugcggaugcu.......................................................................   2      1    seq
....................uggucccugcggauAcu.......................................................................   2      1    seq
....................uggCcccugcggaugcu.......................................................................   4      1    seq
....................uggucccugcggaugGu.......................................................................   2      1    seq
....................uggucccugcggaugcA.......................................................................   70     1    seq
```

Star Mature

aauaaaacaaaucauugugauggucccugcggaugcuaacacaaugugauuuauaaacaaaucauugugauggucccugcggaugcuaacacaaugugauuuauaccc

....................ugguUccugcggaugcu...	8	1	seq
....................uggCcccugcggaugcua..	8	1	seq
....................uggucccugcggauUcua..	2	1	seq
....................uggucccugcggaugcAa..	4	1	seq
....................Gggucccugcggaugcua..	4	1	seq
....................uggucccugcggaCgcua..	4	1	seq
....................Cggucccugcggaugcua..	4	1	seq
....................uggucccugcggaugcCa..	14	1	seq
....................uggucccCgcggaugcua..	2	1	seq
....................uggucccugcggaugcuU..	200	1	seq
....................uggucccugUggaugcua..	2	1	seq
....................uggucccugcggaugcua..	880	0	seq
....................uggucccugcggaugcGa..	2	1	seq
....................uggucccugcggaugcuaU...	2	1	seq
....................uggucccugcggaugcuUa...	2	1	seq
....................uggucccugcggaugcuCa...	8	1	seq
....................uggucccugcggaugcuaa...	4	0	seq
....................uggucccugcggaugcuaac..	2	0	seq
....................uggucccugcggaugcuCaca...	2	1	seq
....................uggucccugcggaugcuaacacaaugug..	2	0	seq
.....................ggucccugcggaugcua..	136	0	seq
.....................ggucccugcggaugcCa..	2	1	seq
.....................ggucccugcggaugcuU..	48	1	seq
.....................Ugucccugcggaugcua..	2	1	seq
.....................ggucccugcggaugcAa..	2	1	seq
......................gucccugcggaugcuCa...	2	1	seq
.......................ucccugcggaugcuUac..	2	1	seq
........................cccugcggaugcuaacCc..	2	1	seq
....cauugugauggucccGg.............................	2	1	seq
....cauugugauggucccAg.............................	2	1	seq
....cauugugauggucccug.............................	26	0	seq
....cauugugauggucccugc............................	2	0	seq
....cauugugauggucccugcgg..........................	10	0	seq
....cauugugauggucccugcggau........................	2	0	seq
....cauugugauggucccugcggaug.......................	6	0	seq
....cauugugauggucccugcggaugc......................	2	0	seq
....cauugugauggucccugcggaugcu.....................	4	0	seq
....auugugauggucccugcgg..........................	2	0	seq
....auugugauggucccugcgga.........................	2	0	seq
....auugGgauggucccugcgga.........................	2	1	seq
....uugugauggucccugcg...........................	2	0	seq
....uugugauggucccugcgg..........................	8	0	seq
....uugugauggucccugcgga.........................	8	0	seq
....uugugauggucccugcggaC........................	4	1	seq
....uugugauggucccugcggau........................	6	0	seq
....uugGgauggucccugcggau........................	2	1	seq
....uugugauggucccugcggaug.......................	18	0	seq
....uugugauggucccugcggauA.......................	4	1	seq
....uugugauggucccugcggaugc......................	4	0	seq
....uugugauggucccugcggaugcu.....................	10	0	seq
....uugugauggucccugcggaugcuU....................	2	1	seq
....uugugauggucccugcggaugcua....................	2	0	seq
....ugugauggucccugcgg..........................	2	0	seq
....ugGgauggucccugcgga.........................	2	1	seq
....ugugauggucccugcgga.........................	6	0	seq
....ugugauggucccugcggaC........................	2	1	seq
....ugugauggucccugcggaug.......................	16	0	seq
....ugGgauggucccugcggaug.......................	8	1	seq
....ugugauggucccugcggauA.......................	2	1	seq
....ugugauggucccugcggaugc......................	8	0	seq
....ugAgauggucccugcggaugcu.....................	2	1	seq
....ugugauggucccugcggaugcu.....................	4	0	seq
....ugugauggucccugcggaugcua....................	6	0	seq
....gugauggucccugcgga.........................	4	0	seq
....gugauggucccugcggaug.......................	2	0	seq
....Ggauggucccugcggau........................	2	1	seq
....ugauggucccugcggau........................	2	0	seq
....Ngauggucccugcggaug.......................	2	1	seq
....Ggauggucccugcggaug.......................	4	1	seq
....ugauggucccugcggaug.......................	8	0	seq

Star / Mature			
aauaaaacaaaucauugugauggucccugcggaugcuaacacaaugugauuuauaaacaaaucauugugauggucccugcggaugcuaacacaaugugauuuauaccc			
....Ngauggucccugcggaugcu.....................	2	1	seq
....Agauggucccugcggaugcu.....................	2	1	seq
....gauggucccugcggauC.......................	10	1	seq
....gaugguccGugcggaug.......................	2	1	seq
....gauggucccugcggGug.......................	2	1	seq
....Cauggucccugcggaug.......................	4	1	seq
....gauggucccugcggaug.......................	1756	0	seq
....gaAggucccugcggaug.......................	2	1	seq
....gauggAcccugcggaug.......................	2	1	seq
....gauggucccugcggaGg.......................	10	1	seq
....Aauggucccugcggaug.......................	2	1	seq
....Nauggucccugcggaug.......................	4	1	seq
....gGuggucccugcggaug.......................	10	1	seq
....gauggucccugcggauA.......................	86	1	seq
....gauggucccGgcggaug.......................	4	1	seq
....gauggucccAgcggaug.......................	2	1	seq
....gauggucccugcggCug.......................	4	1	seq
....gauggCcccugcggaug.......................	8	1	seq
....gauAgucccugcggaug.......................	2	1	seq
....gauggucccugcggaugc......................	158	0	seq
....gaAggucccugcggaugc......................	2	1	seq
....gauggucccugcggGugcu.....................	2	1	seq
....gauggucccugcgCaugcu.....................	2	1	seq
....gauggucccugcggaugcG.....................	18	1	seq
....gauggucccugcggauAcu.....................	2	1	seq
....gGuggucccugcggaugcu.....................	2	1	seq
....gauggucccugcggaugcA.....................	14	1	seq
....gauggucccugcggaugcu.....................	246	0	seq
....gauggucccugcggaugcGa....................	2	1	seq
....gauggucccugcggaugcuU....................	18	1	seq
....gauggucUcugcggaugcua....................	2	1	seq
....gauggucccugcggaugcua....................	80	0	seq
....gauggucccugcgAaugcua....................	2	1	seq
....gauggucccugcggaugcuCa...................	2	1	seq
....gauggucccugcggaugcuUaca.................	2	1	seq
....auggucccugcggaugc......................	68	0	seq
....augguUccugcggaugc......................	2	1	seq
....auggucccugcggCugcu.....................	4	1	seq
....auggucccugcggaugcG.....................	12	1	seq
....aCggucccugcggaugcu.....................	2	1	seq
....auggucccugcggaugcu.....................	98	0	seq
....auggucccugcggaugcua....................	114	0	seq
....auggucccugcggaugcuU....................	38	1	seq
....Nuggucccugcggaugcua....................	2	1	seq
....auggucccugcggaugcCa....................	4	1	seq
....auggucccugcggaugcuaa...................	4	0	seq
....auggucccugcggaugcuUac..................	2	1	seq
....auggucccugcggaugcuaacC.................	2	1	seq
....auggucccugcggaugcuaaca.................	2	0	seq
....Aggucccugcggaugcu.....................	2	1	seq
....ugguUccugcggaugcu.....................	8	1	seq
....uggucccugcggGugcu.....................	16	1	seq
....uggucccugcggauAcu.....................	2	1	seq
....Nggucccugcggaugcu.....................	2	1	seq
....uggCcccugcggaugcu.....................	4	1	seq
....uggucccugcggCugcu.....................	10	1	seq
....uggucccugcggaugcu.....................	1290	0	seq
....Cggucccugcggaugcu.....................	8	1	seq
....uggucccugcggaugcA.....................	70	1	seq
....uggucccugcggaugcG.....................	74	1	seq
....uAgucccugcggaugcu.....................	2	1	seq
....uggucccugcUgaugcu.....................	2	1	seq
....uggucccugcggaugGu.....................	2	1	seq
....uggucccugcggaugcua....................	880	0	seq
....uggucccugcggaugcGa....................	2	1	seq
....uggCcccugcggaugcua....................	8	1	seq
....uggucccugcggauUcua....................	2	1	seq
....uggucccugcggaugcCa....................	14	1	seq
....uggucccugcggaugcuU....................	200	1	seq
....uggucccugcggaCgcua....................	4	1	seq

```
                         Star                                        Mature
aauaaaacaaaucauugugauggucccugcggaugcuaacacaaugugauuuauaaacaaaucauugugauggucccugcggaugcuaacacaaugugauuuauaccc
......................................................................Gggucccugcggaugcua....................     4       1       seq
......................................................................uggucccugUggaugcua....................     2       1       seq
......................................................................uggucccugcggaugcAa....................     4       1       seq
......................................................................Cggucccugcggaugcua....................     4       1       seq
......................................................................uggucccCgcggaugcua....................     2       1       seq
......................................................................uggucccugcggaugcuaa...................     4       0       seq
......................................................................uggucccugcggaugcuCa...................     8       1       seq
......................................................................uggucccugcggaugcuUa...................     2       1       seq
......................................................................uggucccugcggaugcuaU...................     2       1       seq
......................................................................uggucccugcggaugcuaac..................     2       0       seq
......................................................................uggucccugcggaugcuCaca.................     2       1       seq
......................................................................uggucccugcggaugcuaacacaaugug..........     2       0       seq
.......................................................................ggucccugcggaugcCa....................     2       1       seq
.......................................................................ggucccugcggaugcAa....................     2       1       seq
.......................................................................Ugucccugcggaugcua....................     2       1       seq
.......................................................................ggucccugcggaugcuU....................     48      1       seq
.......................................................................ggucccugcggaugcua....................     136     0       seq
........................................................................gucccugcggaugcuCa...................     2       1       seq
.........................................................................ucccugcggaugcuUac..................     2       1       seq
..........................................................................cccugcggaugcuaacCc................     2       1       seq
```

Provisional ID	:	MA_10378255_36883
Score total	:	2.2
Score for star read(s)	:	-1.3
Score for read counts	:	0
Score for mfe	:	1.9
Score for randfold	:	1.6
Score for cons. seed	:	
Total read count	:	16
Mature read count	:	16
Loop read count	:	0
Star read count	:	0

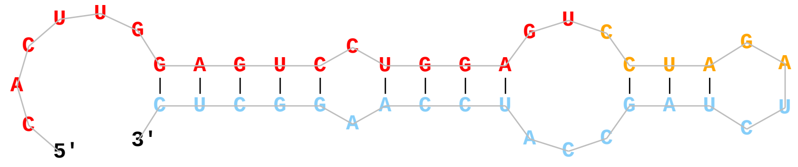

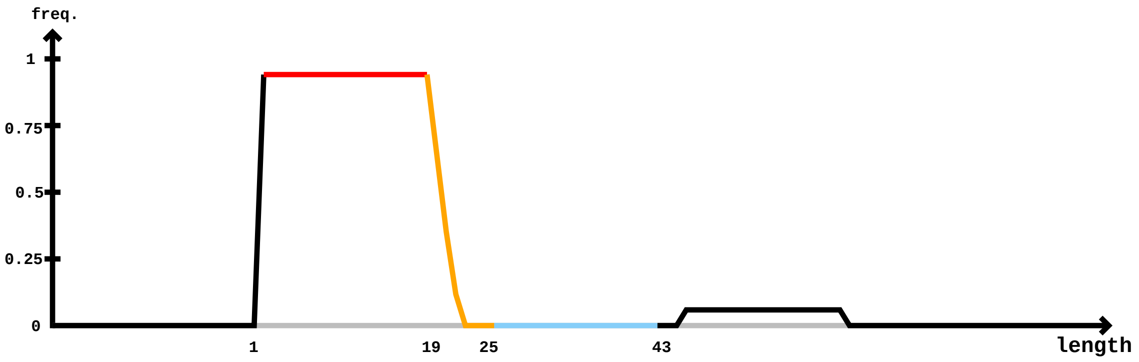

Mature **Star**

```
5'- ugaaggagguaacaucaaaccacuuggaguccuggaguccuagaucuagccauccaaggcucagagagagcucucuuagggcucucucuguuugaucuagcuaucugu   -3'    exp
    ...(((.(((.(.((((((..((((((...(((((........)))))...))))))....((((((((((((.....)))))))))))))))))).).))).)))..   reads      mm        sample
    ....................cacuuggaguccuggagu......................................................................   5          0         seq
    ....................cacuuggaguccuggaguc.....................................................................   4          0         seq
    ....................cacuuggaguccuggaguU.....................................................................   1          1         seq
    ....................cacuuggaguccuggagucA....................................................................   4          1         seq
    ....................cacuuggaguccuggagucAu...................................................................   2          1         seq
    ................................................................agagagcucucuGaggg...........................   1          1         seq
```

Provisional ID	:	MA_10380541_5424
Score total	:	0
Score for star read(s)	:	-1.3
Score for read counts	:	-0.8
Score for mfe	:	0.6
Score for randfold	:	1.6
Score for cons. seed	:	
Total read count	:	10
Mature read count	:	10
Loop read count	:	0
Star read count	:	0

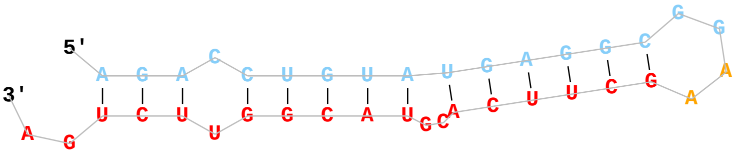

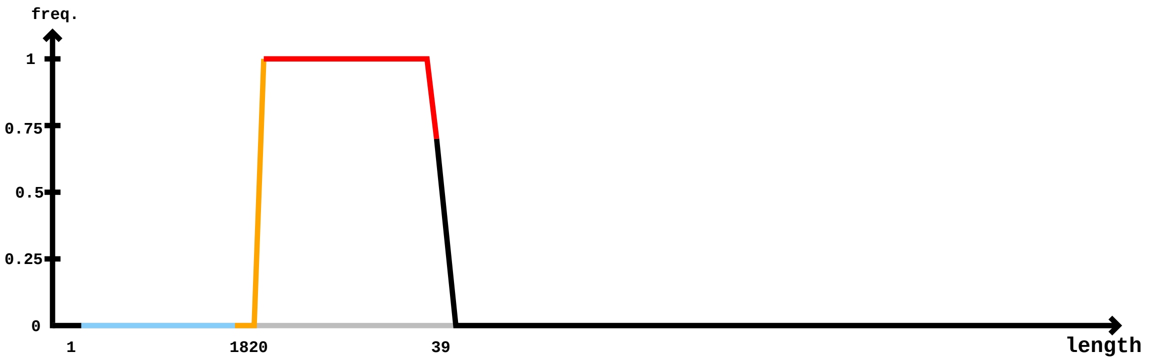

Star **Mature**

```
5'- cagaccuguaugaggcggaagcuucacguacgguucugaggacgaaccugagaucauucauaauagaaucuuuccucucccuugccuacugcaacagggauucacaaau   -3'    exp
    ((((.((((((((((((....))))))..))))).)))).(((.......(((((...((......))))))))))..((((((((.....)))..))))).........    reads      mm         sample
    ...................gcuucacguacgguucug........................................................................    3          0          seq
    ...................gUuucacguacgguucuga.......................................................................    1          1          seq
    ...................gcuucacguacgguucuga.......................................................................    6          0          seq
```

Provisional ID	:	MA_103849_12240
Score total	:	1.5
Score for star read(s)	:	-1.3
Score for read counts	:	0
Score for mfe	:	1.2
Score for randfold	:	1.6
Score for cons. seed	:	
Total read count	:	1420
Mature read count	:	1420
Loop read count	:	0
Star read count	:	0

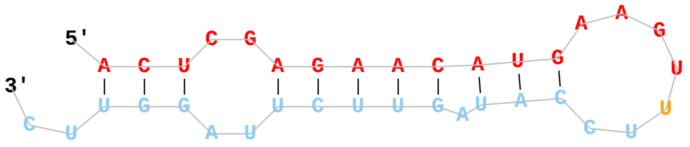

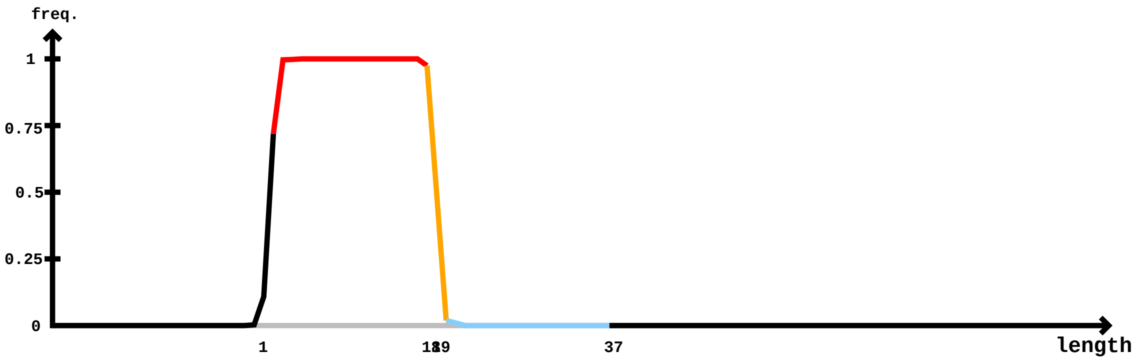

Mature Star

```
5'- uacugguagcauucauagagaacucgagaacaugaaguuuccauaguucuuagguuccauggaugucgcaaccacccaggcaugguuggaaaauauggugaugugauu   -3'   exp
    ((((.(..((((((((.(.(((((..((((((((.......))).)))))..))))))))))))))..)..(((.(((....))).))).......))))........    reads   mm   sample
    ...................gaacucgagaacaugaagu......................................................................    2       0    seq
    ...................gaacucgagaacaugaagG......................................................................    1       1    seq
    ...................Aaacucgagaacaugaaguu.....................................................................    1       1    seq
    ....................aacucgagaacaugaag.......................................................................    35      0    seq
    ....................aacucgGgaacaugaag.......................................................................    1       1    seq
    ....................Gacucgagaacaugaagu......................................................................    1       1    seq
    ....................aacucgagaacaugaagA......................................................................    2       1    seq
    ....................aacucgagaacaugaagC......................................................................    10      1    seq
    ....................aacucgagaacGugaagu......................................................................    1       1    seq
    ....................aacucgagaacaugaagu......................................................................    56      0    seq
    ....................aacucgagaacaugaaguC.....................................................................    5       1    seq
    ....................aacucgagaacaugaaguG.....................................................................    1       1    seq
    ....................aacucgagaacaugaaguu.....................................................................    36      0    seq
    ....................aacucgagaacaugaGguu.....................................................................    1       1    seq
    ....................aacAcgagaacaugaaguu.....................................................................    1       1    seq
    .....................acucgagaGcaugaagu......................................................................    2       1    seq
    .....................acucgagaacaugGagu......................................................................    1       1    seq
    .....................acucgaAaacaugaagu......................................................................    1       1    seq
    .....................acucgagaacGugaagu......................................................................    4       1    seq
    .....................acucgagaacaugaagu......................................................................    476     0    seq
    .....................acucgagaacaugaGgu......................................................................    2       1    seq
    .....................Gcucgagaacaugaagu......................................................................    4       1    seq
    .....................acucgagaacaugaagC......................................................................    62      1    seq
    .....................acucgGgaacaugaagu......................................................................    1       1    seq
    .....................acucgagaacaugaagG......................................................................    5       1    seq
    .....................acCcgagaacaugaagu......................................................................    2       1    seq
    .....................acucgagaacaugaagA......................................................................    3       1    seq
    .....................acucgagGacaugaagu......................................................................    2       1    seq
    .....................acucgagaacaugaaguA.....................................................................    3       1    seq
    .....................acucgaAaacaugaaguu.....................................................................    2       1    seq
    .....................acuGgagaacaugaaguu.....................................................................    1       1    seq
    .....................acucgagaacaugUaguu.....................................................................    1       1    seq
    .....................acucgagaacaugaaguG.....................................................................    3       1    seq
    .....................acucgagaacaCgaaguu.....................................................................    1       1    seq
```

Mature **Star**

uacugguagcauucauagagaacucgagaacaugaaguuuccauaguucuuagguuccauggaugucgcaaccacccaggcaugguuggaaaauauggugaugugauu

.....................Ucucgagaacaugaaguu...	1	1	seq
.....................Gcucgagaacaugaaguu...	2	1	seq
.....................acucgagaacaugaaguu...	252	0	seq
.....................acucgagaacaugaaguC...	30	1	seq
.....................acucgagaacaugaagGu...	1	1	seq
.....................acucgagaacaugaaguuu..	3	0	seq
.....................acucgagaacaugaaguuC..	1	1	seq
.....................acucgagaacaugaaguuuG...	1	1	seq
......................cucgagGacaugaaguu...	1	1	seq
......................cucgagaacaugGaguu...	1	1	seq
......................cucgagaacaugaagGu...	2	1	seq
......................cucgagaGcaugaaguu...	1	1	seq
......................cucgagaacaugaaguG...	4	1	seq
......................cucgagaacaugaGguu...	1	1	seq
......................cucgagaacGugaaguu...	2	1	seq
......................cucgagaacaugaaguC...	39	1	seq
......................cucgagaacUugaaguu...	1	1	seq
......................cucgagaacaugaagCu...	3	1	seq
......................cucgGgaacaugaaguu...	1	1	seq
......................cucgagaacaugaaguu...	315	0	seq
......................cucgagaacaugaaguA...	4	1	seq
......................cCcgagaacaugaaguu...	1	1	seq
......................cucgaAaacaugaaguu...	1	1	seq
......................cucgagaacaugaaguuu..	3	0	seq
......................cucgagaacaugaaguuC..	5	1	seq
......................cucgagaacaugaaguuGc...	9	1	seq
.......................ucgagaacaugaaguGu..	1	1	seq
.......................ucgagaacaugaaguuuG...	1	1	seq
........................cgagaacaugaaguuGc...	3	1	seq

Provisional ID	:	MA_103973_4748
Score total	:	6452.2
Score for star read(s)	:	3.9
Score for read counts	:	6444.3
Score for mfe	:	2.4
Score for randfold	:	1.6
Score for cons. seed	:	
Total read count	:	12652
Mature read count	:	11950
Loop read count	:	0
Star read count	:	702

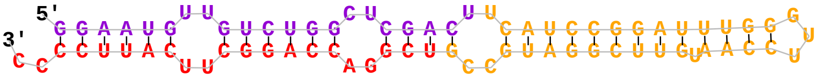

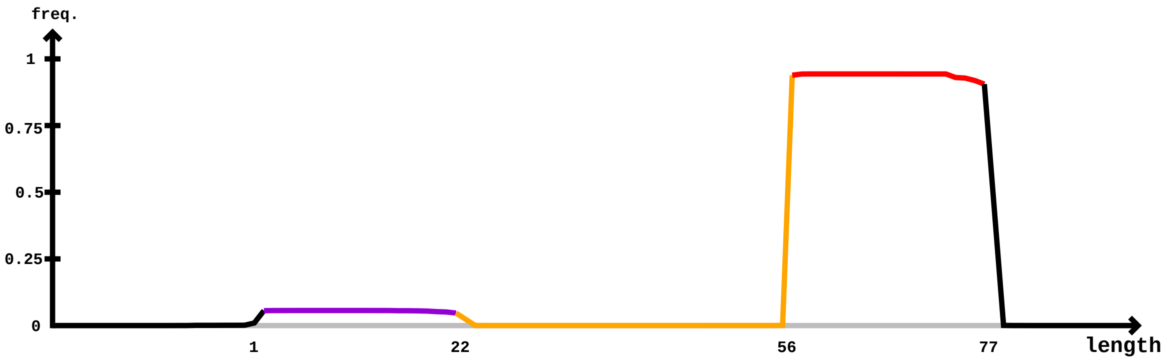

Star   Mature

```
5'- gaccucaauguugauugaggggaauguugucuggcucgacuucauccggauuuggguuccaauguucggaugccgucggaccaggcuucauuccccccaaucggagcucuu  -3'    obs
    gaccucaauguugauugaggggaauguugucuggcucgacuucauccggauuuggguuccaauguucggaugccgucggaccaggcuucauuccccccaaucggagcucuu         exp
    ((.(((.....((((((.((((((((..((((((..((((..((((((((((((...)))).)))))))))..))))..))))))..)))))))).))))))))).))..   reads   mm   sample
    ...........ugauugaggggaauguugucuggcucgacC......................................................................   1       1    seq
    .............auugaggggaauguugucu...............................................................................   2       0    seq
    .............auugaggggaauguugucugU.............................................................................   1       1    seq
    .............auugaggggaauguugucugg.............................................................................   7       0    seq
    ...............ugaggggaauguugucuggcucgacu......................................................................   1       0    seq
    ................gaggggaauguugucuggcuc..........................................................................   1       0    seq
    ................gaggggaauguugucuggcuU..........................................................................   1       1    seq
    ..................ggggaauguugucuggcu...........................................................................   1       0    seq
    ..................ggggaauguugucuggcucgacC......................................................................   1       1    seq
    ...................gggaauguugucuggcG...........................................................................   1       1    seq
    ...................gggaauguugucuggcC...........................................................................   4       1    seq
    ...................gggaauguugucuggcu...........................................................................   1       0    seq
    ...................gggaauguugucuggcuc..........................................................................   7       0    seq
    ...................gggaauguugucuggcucU.........................................................................   1       1    seq
    ...................gggaauguugucuggcucg.........................................................................   7       0    seq
    ...................gggaauguugucuggcucga........................................................................   1       0    seq
    ...................gggaauguugucuggcucgac.......................................................................   34      0    seq
    ...................gggaauguCgucuggcucgac.......................................................................   1       1    seq
    ...................gggaauguugucuggcucgaU.......................................................................   3       1    seq
    ...................gggaauguugucuggcGcgac.......................................................................   1       1    seq
    ...................gggaauguugucuggcucgacC......................................................................   6       1    seq
    ...................gggaauguugucuggcucgacu......................................................................   34      0    seq
    ...................gggaauguugucuggcucgacG......................................................................   1       1    seq
    ...................gggaauguugucuggcucgacA......................................................................   3       1    seq
    ....................ggaauguugucuggcuc..........................................................................   2       0    seq
    ....................ggaauguugucuggcucA.........................................................................   1       1    seq
    ....................ggaauguugucuggcucg.........................................................................   17      0    seq
    ....................ggaauguugucuggcucga........................................................................   14      0    seq
    ....................ggaauguugucuggcucgG........................................................................   1       1    seq
    ....................ggaauguugucuggcucgac.......................................................................   9       0    seq
    ....................ggGauguugucuggcucgacu......................................................................   1       1    seq
    ....................ggaauguugucuggcucgacu......................................................................   423     0    seq
    ....................ggaauguugucuggcuUgacu......................................................................   2       1    seq
```

Star | Mature

```
gaccucaauguugauugaggggaauguugucuggcucgacuucauccggauuuggguuccaauguucggaugccgucggaccaggcuucauuccccccaaucggagcucuu
....................ggaauguugucuggcucgaAu......................................................................  1    1  seq
....................ggaauguugucuggcucgacG......................................................................  16   1  seq
....................ggaauguugucuggcucgacC......................................................................  68   1  seq
....................ggaauguugucuggcucgGcu......................................................................  2    1  seq
....................ggaauguugucuggcucgaGu......................................................................  5    1  seq
....................ggaauguugucuggcucgaUu......................................................................  1    1  seq
....................ggaauguugucuggcuGgacu......................................................................  1    1  seq
....................ggaauguugucCggcucgacu......................................................................  1    1  seq
....................ggaauguugCcuggcucgacu......................................................................  1    1  seq
....................ggaauguugucugAcucgacu......................................................................  1    1  seq
....................ggaauguugucuggcuAgacu......................................................................  1    1  seq
....................ggaaugGugucuggcucgacu......................................................................  1    1  seq
....................ggaauguugucuggcucgacA......................................................................  12   1  seq
....................ggaauguugucuggUucgacu......................................................................  1    1  seq
....................Agaauguugucuggcucgacu......................................................................  1    1  seq
....................ggaauguugucuggcucgacuuc....................................................................  2    0  seq
....................ggaauguugucuggcucgacuCc....................................................................  1    1  seq
.....................gaauguugucuggcucA.........................................................................  1    1  seq
.....................gaauguugucuggcucgac.......................................................................  1    0  seq
.....................gaGuguugucuggcucgacu......................................................................  1    1  seq
.....................gaauguugucuggcucgacu......................................................................  1    0  seq
.....................gaauguugucuggcucgacuu.....................................................................  1    0  seq
.....................gaauguugucuggcucgacuC.....................................................................  1    1  seq
......................aauguugucuggcucgac.......................................................................  1    0  seq
......................aauguugucuggcucgacu......................................................................  1    0  seq
.......................auguugucuggcucgac.......................................................................  1    0  seq
...................................................................ggUugccgucggaccaggcu........................  1    1  seq
..........................................................................gucggaccaggcuucauuccc................  2    0  seq
..........................................................................gucggaccaggcuucauuccA................  2    1  seq
..........................................................................Cucggaccaggcuucauucccc...............  1    1  seq
...........................................................................ucggaccaggcuucGuu...................  2    1  seq
...........................................................................ucggaccaggcuucauC...................  15   1  seq
...........................................................................ucggGccaggcuucauu...................  1    1  seq
...........................................................................ucggaccaggcuucauA...................  1    1  seq
...........................................................................ucggaccaggcuucauu...................  146  0  seq
...........................................................................ucggaccaggcuucaCu...................  1    1  seq
...........................................................................ucggacUaggcuucauu...................  1    1  seq
...........................................................................ucggaccaggcuucauuc..................  21   0  seq
...........................................................................ucggaccaggcuucauuU..................  4    1  seq
...........................................................................ucggaccaggcuucauuA..................  1    1  seq
...........................................................................ucggaccaggcuucauucG.................  1    1  seq
...........................................................................ucggaccaggcuucauucU.................  10   1  seq
...........................................................................ucggaccaggcuucauuUc.................  1    1  seq
...........................................................................ucggacUaggcuucauucc.................  1    1  seq
...........................................................................ucggaUcaggcuucauucc.................  1    1  seq
...........................................................................ucAgaccaggcuucauucc.................  1    1  seq
...........................................................................ucggaccaggcuCcauucc.................  1    1  seq
...........................................................................ucggaccaggcuucauucc.................  88   0  seq
...........................................................................ucggaccaggcuucauucA.................  4    1  seq
...........................................................................ucggaccaggcuucaCucc.................  1    1  seq
...........................................................................ucggaccaggcuucauuccA................  4    1  seq
...........................................................................ucggaUcaggcuucauuccc................  1    1  seq
...........................................................................ucggaccaggcuucauCccc................  1    1  seq
...........................................................................ucggacAaggcuucauuccc................  1    1  seq
...........................................................................ucggaccaggcuucaGuccc................  1    1  seq
...........................................................................uUggaccaggcuucauuccc................  1    1  seq
...........................................................................ucggaccGggcuucauuccc................  1    1  seq
...........................................................................ucggaccaggcuucauuccc................  147  0  seq
...........................................................................ucgUaccaggcuucauuccc................  1    1  seq
...........................................................................ucggaccaggcuCcauuccc................  1    1  seq
...........................................................................ucCgaccaggcuucauuccc................  1    1  seq
...........................................................................ucggaccaggcuucauucAc................  1    1  seq
...........................................................................ucggaAcaggcuucauuccc................  1    1  seq
...........................................................................Ncggaccaggcuucauuccc................  1    1  seq
...........................................................................ucggacUaggcuucauucccc...............  10   1  seq
...........................................................................ucggaccaggcuucauCcccc...............  40   1  seq
...........................................................................ucggaccaggcuucCuucccc...............  4    1  seq
...........................................................................Acggaccaggcuucauucccc...............  7    1  seq
...........................................................................ucggaccaggcuucaGucccc...............  3    1  seq
...........................................................................ucggaccaggcuucauucccG...............  47   1  seq
```

```
                    Star                                                   Mature
gaccucaauguugauugaggggaauguugucuggcucgacuucauccggauuuggguuccaauguucggaugccgucggaccaggcuucauuccccccaaucggagcucuu
...........................................................................ucggaccaggcuucauucUcc...............   11    1  seq
...........................................................................ucggaccaggcAucauucccc...............   3     1  seq
...........................................................................ucggaccaggAuucauucccc...............   4     1  seq
...........................................................................ucggaccaggcuucauucGcc...............   1     1  seq
...........................................................................ucggaccaggcuuAauucccc...............   1     1  seq
...........................................................................ucggaccaggcuucauuAccc...............   2     1  seq
...........................................................................ucggaccaggcuucauAcccc...............   3     1  seq
...........................................................................ucggaccagUcuucauucccc...............   1     1  seq
...........................................................................ucggaccaggcuucauucccc...............   8863  0  seq
...........................................................................ucggaGcaggcuucauucccc...............   1     1  seq
...........................................................................ucggaccaggcuucauuUccc...............   5     1  seq
...........................................................................ucggGccaggcuucauucccc...............   33    1  seq
...........................................................................Ncggaccaggcuucauucccc...............   7     1  seq
...........................................................................ucggaccGggcuucauucccc...............   20    1  seq
...........................................................................ucggaccagCcuucauucccc...............   1     1  seq
...........................................................................ucggaccaggcuuUauucccc...............   9     1  seq
...........................................................................ucggaccaggcuucauuccNc...............   1     1  seq
...........................................................................uUggaccaggcuucauucccc...............   7     1  seq
...........................................................................ucgAaccaggcuucauucccc...............   5     1  seq
...........................................................................ucggaccaUgcuucauucccc...............   3     1  seq
...........................................................................ucggaccaggcuGcauucccc...............   1     1  seq
...........................................................................ucggaccaggcuAcauucccc...............   10    1  seq
...........................................................................ucggaccaggcuucauucccU...............   1444  1  seq
...........................................................................ucggaccaggcCucauucccc...............   25    1  seq
...........................................................................ucggUccaggcuucauucccc...............   2     1  seq
...........................................................................ucggaccagAcuucauucccc...............   3     1  seq
...........................................................................ucggaccaggcuucaCucccc...............   9     1  seq
...........................................................................ucggaccCggcuucauucccc...............   2     1  seq
...........................................................................ucAgaccaggcuucauucccc...............   9     1  seq
...........................................................................ucggaccaCgcuucauucccc...............   1     1  seq
...........................................................................ucggaAcaggcuucauucccc...............   4     1  seq
...........................................................................ucggaccaggcuucauuccAc...............   7     1  seq
...........................................................................Gcggaccaggcuucauucccc...............   12    1  seq
...........................................................................ucUgaccaggcuucauucccc...............   2     1  seq
...........................................................................ucggaccaggcuCcauucccc...............   34    1  seq
...........................................................................ucggaUcaggcuucauucccc...............   4     1  seq
...........................................................................ucggaccaggcuucauucAcc...............   10    1  seq
...........................................................................ucggaccaggGuucauucccc...............   4     1  seq
...........................................................................ucggaccaggcuucauucccA...............   323   1  seq
...........................................................................ucggaccaggcuucauuGccc...............   1     1  seq
...........................................................................ucggaccaAgcuucauucccc...............   7     1  seq
...........................................................................ucggaccaggcuucUuucccc...............   1     1  seq
...........................................................................ucggaccaggcuucauGcccc...............   1     1  seq
...........................................................................ucggaccUggcuucauucccc...............   5     1  seq
...........................................................................uAggaccaggcuucauucccc...............   1     1  seq
...........................................................................ucggaccaggUuucauucccc...............   5     1  seq
...........................................................................ucCgaccaggcuucauucccc...............   1     1  seq
...........................................................................ucggaccaggcuucGuucccc...............   34    1  seq
...........................................................................ucgUaccaggcuucauucccc...............   2     1  seq
...........................................................................ucggaccaggcCucauuccccc..............   1     1  seq
...........................................................................ucggaccaggcuucauuUcccc..............   1     1  seq
...........................................................................ucggaccaggcuucauuccGcc..............   1     1  seq
...........................................................................ucggaccaggcuucauuccccA..............   301   1  seq
...........................................................................ucggaccaggcuucauuccccG..............   21    1  seq
...........................................................................ucggaccaggcuucauuccccc..............   38    0  seq
...........................................................................ucggaccaggcuucauucccccA.............   1     1  seq
...........................................................................ucggaccaggcuucauucccccaC............   1     1  seq
............................................................................cggaccaggcuuUauuc..................   1     1  seq
............................................................................cggaccaggcuucauucc.................   6     0  seq
............................................................................Uggaccaggcuucauucc.................   1     1  seq
............................................................................cggaccaggcuucauuccc................   4     0  seq
............................................................................cggaccaggcuucauucccc...............   8     0  seq
............................................................................cggaccaggcuucauucccU...............   5     1  seq
............................................................................cggaccaggcuucauuccccc..............   23    0  seq
............................................................................cggaccaggcuucauuccccA..............   3     1  seq
............................................................................cggaccaggcuucauucccccA.............   2     1  seq
............................................................................cggaccaggcuucauucccccc.............   1     0  seq
............................................................................cggaccaggcuucauucccccU.............   2     1  seq
.............................................................................ggaccaggcuucauucccc...............   3     0  seq
```

Provisional ID	:	MA_10426348_22475
Score total	:	1.3
Score for star read(s)	:	-1.3
Score for read counts	:	0
Score for mfe	:	1
Score for randfold	:	1.6
Score for cons. seed	:	
Total read count	:	267
Mature read count	:	267
Loop read count	:	0
Star read count	:	0

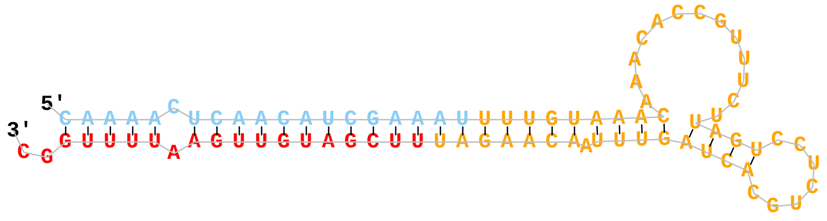

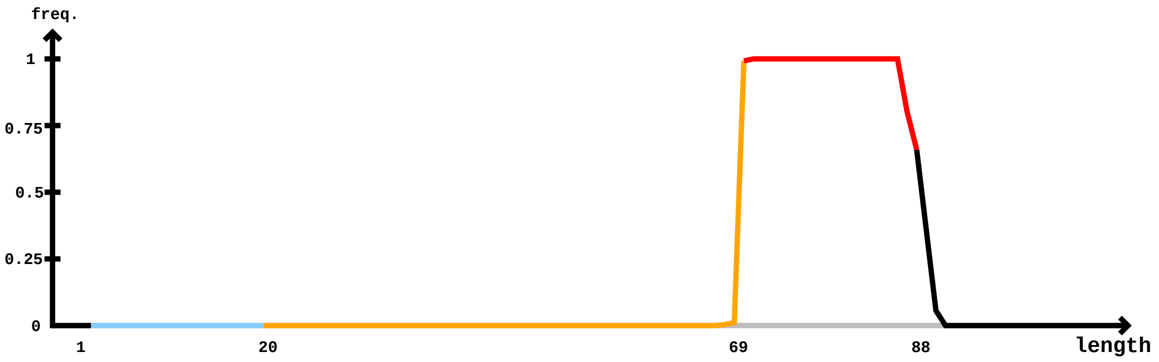

Star Mature

```
5'- gucaaaacucaacaucgaaauuuuguaaacaaacaccguuucuuaguccucugcacuaguuuaacaagauuucgauguugaauuuuggcgacaagguuaacuuuauucca   -3'    exp
    (((((((.(((((((((((((((((((((.............((((.......)))))))).))))))))))))))))))).)))))))...((((....))))......      reads      mm        sample
    ....................................................................Cuuucgauguugaauuuuggc.....................      1          1         seq
    .....................................................................Guucgauguugaauuuuggc.....................      2          1         seq
    ......................................................................uucgaCguugaauuuug.......................      1          1         seq
    ......................................................................uucgauguugaauuuug.......................      52         0         seq
    ......................................................................uucgauguugaauuuugC......................      2          1         seq
    ......................................................................uucgauguugaauuuugU......................      1          1         seq
    ......................................................................uucgauguugGauuuugg......................      1          1         seq
    ......................................................................uucgauguugaauuuugg......................      33         0         seq
    ......................................................................uucgauguugaGuuuugg......................      1          1         seq
    ......................................................................uucgauguugaauuuuggc.....................      119        0         seq
    ......................................................................Gucgauguugaauuuuggc.....................      2          1         seq
    ......................................................................uAcgauguugaauuuuggc.....................      1          1         seq
    ......................................................................uucgauguugaauuuugUc.....................      1          1         seq
    ......................................................................uucgauguugaauuuuggU.....................      25         1         seq
    ......................................................................uucgauguugaauuuuggcg....................      8          0         seq
    ......................................................................uucgauguugaauuuuggcga...................      11         0         seq
    ......................................................................uucgauguugaauuuuggcgC...................      2          1         seq
    ......................................................................uucgGuguugaauuuuggcga...................      1          1         seq
    ......................................................................uucgauguugaauuuuggcgG...................      1          1         seq
    .......................................................................ucgauguugaauuuuggc.....................      1          0         seq
    .......................................................................ucgauguugaauuuuggU.....................      1          1         seq
```

Provisional ID	:	MA_10426798_17777
Score total	:	1.4
Score for star read(s)	:	-1.3
Score for read counts	:	0
Score for mfe	:	1.1
Score for randfold	:	1.6
Score for cons. seed	:	
Total read count	:	15266
Mature read count	:	15266
Loop read count	:	0
Star read count	:	0

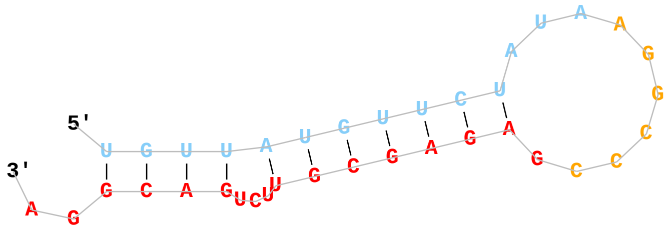

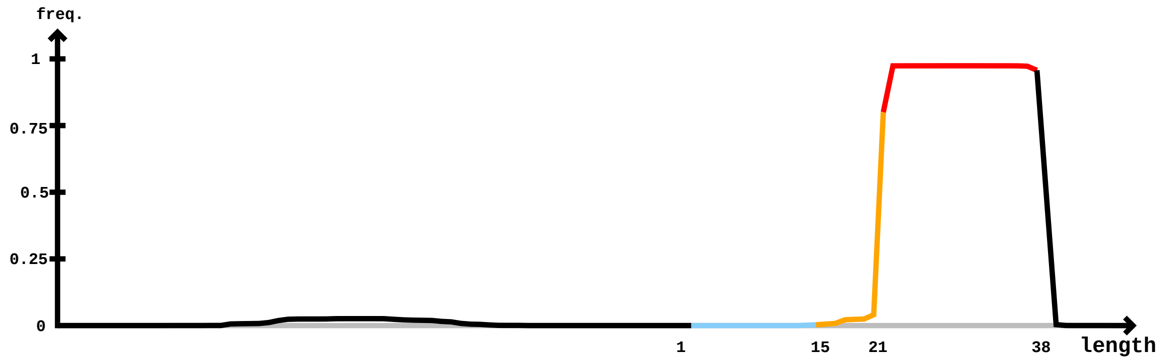

Star Mature

```
5'- uuugaucaugaccuuccuugaacaggaucuguucuauagguugaacaggcucuuguacgggaucuguuauguucuauaaggcccgagagcguucugacggauccguuucc -3'   exp
    ...................(.(((((((((((((........))))))).))))))).).((((((((((((((((..........)))))))...)))))))))......    reads   mm   sample
    ..........accuuGcuugaacaggaucugu..............................................................................    1    1    seq
    ..............uGcuugaacaggaucuguu.............................................................................    3    1    seq
    ..............uGcuugaacaggaucuguuc............................................................................    1    1    seq
    ................cuugaacaggaucuguC.............................................................................    3    1    seq
    ................cuugGacaggaucuguu.............................................................................    1    1    seq
    ................cuugaacaggaucuguu.............................................................................    29   0    seq
    ................cuCgaacaggaucuguu.............................................................................    1    1    seq
    ................cuugaacaggaucuguuA............................................................................    1    1    seq
    ................cuugaacaggaucuguuc............................................................................    25   0    seq
    ................cuugaacaggaucuguCc............................................................................    1    1    seq
    ................Guugaacaggaucuguuc............................................................................    1    1    seq
    ................cuugaacaggGucuguucu...........................................................................    1    1    seq
    ................cuuAaacaggaucuguucu...........................................................................    1    1    seq
    ................cuugaacaggaucuguucG...........................................................................    1    1    seq
    ................cuugaacaggaucuguucC...........................................................................    1    1    seq
    ................cuugaacaggaucuguucu...........................................................................    12   0    seq
    ................cuugaacaggaucuguucua..........................................................................    6    0    seq
    ................cuugaacaggaucuguucuau.........................................................................    1    0    seq
    ................cuugaacaggaucuguucuauagguug...................................................................    2    0    seq
    ................cuugaacaggaucuguucuauagguugUaca...............................................................    4    1    seq
    .................uugaacaggaucuguuc............................................................................    6    0    seq
    .................uugaacaggaucuguucua..........................................................................    1    0    seq
    .................uugaacaggaucuguucuau.........................................................................    1    0    seq
    .................uugaacaggaucuguucuauag.......................................................................    1    0    seq
    .................uugaacaggaucuguucuauagguug...................................................................    3    0    seq
    .................uugaacaggaucuguucuauagguugC..................................................................    1    1    seq
    ..................ugaacaggaucuguucu...........................................................................    1    0    seq
    ..................ugaacaggaucuguucua..........................................................................    1    0    seq
    ..................ugaacaggaucuguucuauagg......................................................................    1    0    seq
    ..................ugaacaggaucuguucuauaggu.....................................................................    2    0    seq
    ..................ugaacaggaucuguucuauagguug...................................................................    2    0    seq
    ..................ugaacaggaucuguucuauagguugC..................................................................    1    1    seq
    ..................ugaacaggaucuguucuauagguugUaca...............................................................    1    1    seq
    ...................gaacaggaucuguucua..........................................................................    2    0    seq
```

Star Mature

```
uuugaucaugaccuuccuugaacaggaucuguucuauagguugaacaggcucuuguacgggaucuguuauguucuauaaggcccgagagcguucugacggauccguuucc
...................gaacaggaucuguucuauagg......................................................................	1	0	seq
...................gaacaggaucuguucuauagguu....................................................................	1	0	seq
...................gaacaggaucuguucuauagguug...................................................................	1	0	seq
...................gaacaggaucuguucuauagguugG..................................................................	1	1	seq
...................gaacaggaucuguucuauagguugUaca...............................................................	1	1	seq
....................aacaggaucuguucuaC.........................................................................	3	1	seq
....................aacaggaucuguucuau.........................................................................	4	0	seq
....................aacaggaucuguucuauG........................................................................	1	1	seq
....................aacaggaucuguucuaua........................................................................	23	0	seq
....................aacaggaucuguucuauag.......................................................................	3	0	seq
....................aacaggaucuguucuauagg......................................................................	16	0	seq
....................Uacaggaucuguucuauagg......................................................................	1	1	seq
....................aacaggaucuguucuauaggu.....................................................................	4	0	seq
....................aacaggaucuguucuauaggC.....................................................................	1	1	seq
....................aacaggaucuguucuauagguug...................................................................	1	0	seq
.....................acaggaucugCucuaua........................................................................	1	1	seq
.....................acaggaucuguucuaua........................................................................	30	0	seq
.....................acaggaucuguucuauG........................................................................	1	1	seq
.....................acaggaucuguucuauag.......................................................................	19	0	seq
.....................acaggaucuguucuauaA.......................................................................	1	1	seq
.....................acGgaucuguucuauagg.......................................................................	1	1	seq
.....................acaggaucuguucuauagg......................................................................	31	0	seq
.....................acaggaucuguucuauaAgu.....................................................................	1	1	seq
.....................acaggaucuguucuauaggu.....................................................................	13	0	seq
.....................acaggaucuguucuauagguu....................................................................	3	0	seq
.....................acaggaucuguucuauagguG....................................................................	1	1	seq
.....................acaggaucuguucuauagguuA...................................................................	1	1	seq
.....................acaggaucuguucuauagguug...................................................................	13	0	seq
.....................acaggaucuguucuauagguugUaca...............................................................	4	1	seq
......................caggaucuguucuauag.......................................................................	3	0	seq
......................caggaucuguucuauagC......................................................................	2	1	seq
......................caggaucuguucuauagg......................................................................	32	0	seq
......................caggauUuguucuauagg......................................................................	1	1	seq
......................caggaucuguucuauaggu.....................................................................	19	0	seq
......................caggaucuguucuauagguu....................................................................	7	0	seq
......................caggaucCguucuauagguug...................................................................	1	1	seq
......................caggaucuguucuauagguug...................................................................	10	0	seq
......................caggaucuguucuauagguugG..................................................................	1	1	seq
......................caggaucuguucuauagguugC..................................................................	1	1	seq
.......................aggaucugCucuauagg......................................................................	1	1	seq
.......................aggaucuguucuauagg......................................................................	7	0	seq
.......................aggaucuguucuauaggu.....................................................................	5	0	seq
.......................aggaucuguucuauagguug...................................................................	1	0	seq
.......................aggaucuguucuauagguugC..................................................................	1	1	seq
........................ggaucuguucuauagguugC..................................................................	1	1	seq
..........................aucuguucuauagguug...................................................................	1	0	seq
..........................aucuguucuauagguugC..................................................................	1	1	seq
..........................aucuguucuauagguugG..................................................................	1	1	seq
...........................ucuguucuauagguugC..................................................................	11	1	seq
...........................ucuguucuauagguugG..................................................................	3	1	seq
...........................ucGguucuauagguuga..................................................................	1	1	seq
...........................ucuguucuauagguuga..................................................................	2	0	seq
............................cuguucuauagguugGa.................................................................	1	1	seq
............................................................................Gaaggcccgagagcguucugacgg..........	1	1	seq
............................................................................Gaaggcccgagagcguucugacgga.........	5	1	seq
............................................................................Gaaggcccgagagcguucugacggau........	12	1	seq
.............................................................................aaggcccgGgagcguucugacgg..........	1	1	seq
.............................................................................aaggcccgagagcguucugacgg..........	5	0	seq
.............................................................................aaggcccgagagcguucugacggG.........	1	1	seq
.............................................................................aaggccAgagagcguucugacgga.........	1	1	seq
.............................................................................aaggcccgagagcguucugacgga.........	7	0	seq
.............................................................................aaggccAgagagcguucugacggau........	4	1	seq
.............................................................................aaggcccgagagcguucugacggaC........	2	1	seq
.............................................................................aaggcccgagagcguucugacgCau........	1	1	seq
.............................................................................aaggccGgagagcguucugacggau........	1	1	seq
.............................................................................aaggcccgagagcguucugacggau........	9	0	seq
..............................................................................aggcccgagagcguucC...............	1	1	seq
..............................................................................aggcccgagagcguucuga.............	1	0	seq
..............................................................................aggcccgagagcguucugacgU..........	1	1	seq
..............................................................................aggcccgagagcguucugacgA..........	1	1	seq
```

Star Mature

uuugaucaugaccuuccuugaacaggaucuguucuauagguugaacaggcucuuguacgggaucuguuauguucuauaaggcccgagagcguucugacggauccguuucc			
....aggccAgagagcguucugacgg..........	2	1	seq
....aggcccgagagcguucugacgg..........	6	0	seq
....aggcccgagagcguucugacgga.........	20	0	seq
....aggccAgagagcguucugacgga.........	3	1	seq
....aggcUcgagagcguucugacgga.........	1	1	seq
....Nggcccgagagcguucugacgga.........	1	1	seq
....aggcccgagagcguucugacggaC........	3	1	seq
....aggcccgagagcguucugacggau........	21	0	seq
....aggccAgagagcguucugacggau........	5	1	seq
....aggcccgagaAcguucugacggau........	1	1	seq
....ggccAgagagcguucugacgg..........	1	1	seq
....ggcccgagagcguucugacgg..........	1	0	seq
....ggcccgagagcguucugacgga.........	4	0	seq
....ggUccgagagcguucugacgga.........	1	1	seq
....ggcccgagaAcguucugacgga.........	1	1	seq
....ggccAgagagcguucugacgga.........	1	1	seq
....ggccAgagagcguucugacggau........	1	1	seq
....ggcccgagagcguucugacggau........	1	0	seq
....gccAgagagcguucugac............	2	1	seq
....gcccgagagcguucugacg...........	8	0	seq
....gGccgagagcguucugacg...........	1	1	seq
....gccAgagagcguucugacg...........	2	1	seq
....gcccgagagcguucugacA...........	3	1	seq
....gccGgagagcguucugacg...........	2	1	seq
....gcccgagagcguucugacC...........	1	1	seq
....gcccgagagcguucugacgg..........	60	0	seq
....gccGgagagcguucugacgg..........	3	1	seq
....gccAgagagcguucugacgg..........	27	1	seq
....gcccgagagcgCucugacgg..........	1	1	seq
....gcGcgagagcguucugacgg..........	1	1	seq
....gcccgagagcguucugacgU..........	1	1	seq
....gcccgagagcguucugacgA..........	5	1	seq
....gcccgagagcguucugacgC..........	1	1	seq
....gcccgagagcguucugGcgg..........	1	1	seq
....gcUcgagagcguucugacgg..........	1	1	seq
....gcccgagaAcguucugacgg..........	2	1	seq
....gGccgagagcguucugacgga.........	1	1	seq
....gcccgagagcguucugacggG.........	2	1	seq
....gcccgagagcguucugacgga.........	46	0	seq
....gccAgagagcguucugacgga.........	15	1	seq
....gcccgagagcguucugacggU.........	1	1	seq
....gccGgagagcguucugacgga.........	7	1	seq
....gcUcgagagcguucugacggau........	2	1	seq
....gcccgagaAcguucugacggau........	3	1	seq
....gcccgagagcguucugacggau........	14	0	seq
....gcccgagagcguucugacggaC........	1	1	seq
....gcccgagagcguucugCcggau........	1	1	seq
....gccGgagagcguucugacggau........	1	1	seq
....gccAgagagcguucugacggau........	2	1	seq
....ccGgagagcguucugac............	1	1	seq
....cccgagagcguucugacg...........	3	0	seq
....Gccgagagcguucugacgg..........	1	1	seq
....ccAgagagcguucugacgg..........	1	1	seq
....cUcgagagcguucugacgg..........	1	1	seq
....cccgagagcguucugacgA..........	1	1	seq
....ccGgagagcguucugacgg..........	1	1	seq
....cccgagagcguucugacgg..........	2	0	seq
....cUcgagagcguucugacgga.........	1	1	seq
....Gccgagagcguucugacgga.........	2	1	seq
....ccGgagagcguucugacgga.........	4	1	seq
....cccgagagcguucugacgga.........	6	0	seq
....cccgagagcguucugacggau........	3	0	seq
....Gccgagagcguucugacggau........	2	1	seq
....cAgagagcguucugacgg..........	1	1	seq
....ccgagagcguucugacgg..........	7	0	seq
....cGgagagcguucugacgg..........	1	1	seq
....cAgagagcguucugacgga.........	1	1	seq
....cAgagagcguucugacggau........	2	1	seq
....Ucgagagcguucugacggau........	2	1	seq
....ccgagagcguucugacggau........	4	0	seq

Star Mature

```
uuugaucaugaccuuccuugaacaggaucuguucuauagguugaacaggcucuuguacgggaucuguuauguucuauaaggcccgagagcguucugacggauccguuucc
...................................................................................cgagaAcguucugacgg..........	98	1	seq
...................................................................................Ggagagcguucugacgg..........	2	1	seq
...................................................................................Agagagcguucugacgg..........	1	1	seq
...................................................................................cgagagcguucugacgg..........	5	0	seq
...................................................................................cgagagcguucugacgga.........	6	0	seq
...................................................................................cgagagcguucugacggU.........	1	1	seq
...................................................................................cgUgagcguucugacgga.........	1	1	seq
...................................................................................Ggagagcguucugacgga.........	1	1	seq
...................................................................................cgagaAcguucugacgga.........	50	1	seq
...................................................................................Ngagagcguucugacgga.........	1	1	seq
...................................................................................Agagagcguucugacggau........	1	1	seq
...................................................................................Ngagagcguucugacggau........	2	1	seq
...................................................................................cgagaAcguucugacggau........	77	1	seq
...................................................................................cgagagcguucugacggau........	10	0	seq
...................................................................................cgagaAcguucugacggauc.......	2	1	seq
....................................................................................gagagcguucugaAgga.........	1	1	seq
....................................................................................gagagcguucuAacgga.........	2	1	seq
....................................................................................gagagcguucugacgga.........	3389	0	seq
....................................................................................gaAagcguucugacgga.........	2	1	seq
....................................................................................gagaUcguucugacgga.........	1	1	seq
....................................................................................gagagcguucugacgAa.........	5	1	seq
....................................................................................gagagcguucGgacgga.........	3	1	seq
....................................................................................gagagcguucugCcgga.........	1	1	seq
....................................................................................gaUagcguucugacgga.........	2	1	seq
....................................................................................gagagcguucugacggU.........	31	1	seq
....................................................................................gagagcguCcugacgga.........	7	1	seq
....................................................................................gagagcguucugGcgga.........	11	1	seq
....................................................................................gagagcAuucugacgga.........	4	1	seq
....................................................................................gagagUguucugacgga.........	3	1	seq
....................................................................................gUgagcguucugacgga.........	2	1	seq
....................................................................................gagagcguucugacUga.........	1	1	seq
....................................................................................gagagGguucugacgga.........	1	1	seq
....................................................................................gagagcguucugacgCa.........	6	1	seq
....................................................................................gagagcguucugUcgga.........	2	1	seq
....................................................................................Uagagcguucugacgga.........	6	1	seq
....................................................................................gagagcguucugaGgga.........	1	1	seq
....................................................................................gagaAcguucugacgga.........	5043	1	seq
....................................................................................gagagcguAcugacgga.........	1	1	seq
....................................................................................gagagcguucAgacgga.........	1	1	seq
....................................................................................gagCgcguucugacgga.........	2	1	seq
....................................................................................gagagcguGcugacgga.........	2	1	seq
....................................................................................gagagcgCucugacgga.........	11	1	seq
....................................................................................gagagcgAucugacgga.........	1	1	seq
....................................................................................gagagcguucugacggC.........	4	1	seq
....................................................................................gagagcguucugacAga.........	3	1	seq
....................................................................................gGgagcguucugacgga.........	12	1	seq
....................................................................................Aagagcguucugacgga.........	4	1	seq
....................................................................................gagagcguucugacggG.........	94	1	seq
....................................................................................gagagcguuGugacgga.........	1	1	seq
....................................................................................gagaCcguucugacgga.........	1	1	seq
....................................................................................Nagagcguucugacgga.........	2	1	seq
....................................................................................gagUgcguucugacgga.........	3	1	seq
....................................................................................gagagcguucCgacgga.........	4	1	seq
....................................................................................gagagcguucugacggaA........	10	1	seq
....................................................................................gagaAcguucugacggau........	1909	1	seq
....................................................................................gGgagcguucugacggau........	5	1	seq
....................................................................................gaUagcguucugacggau........	1	1	seq
....................................................................................Uagagcguucugacggau........	1	1	seq
....................................................................................gagagcguucugacggau........	1325	0	seq
....................................................................................gagagcguucugacggCu........	4	1	seq
....................................................................................gaAagcguucugacggau........	1	1	seq
....................................................................................gagagcguucugacUgau........	1	1	seq
....................................................................................gagagcgCucugacggau........	1	1	seq
....................................................................................Aagagcguucugacggau........	5	1	seq
....................................................................................gagagcguucugaUggau........	2	1	seq
....................................................................................gagUgcguucugacggau........	1	1	seq
....................................................................................Nagagcguucugacggau........	4	1	seq
....................................................................................gagagcguucugGcggau........	10	1	seq
....................................................................................gagagcguCcugacggau........	4	1	seq
....................................................................................gagagcguucCgacggau........	1	1	seq
```

Star Mature

uuugaucaugaccuuccuugaacaggaucuguucuauagguugaacaggcucuuguacgggaucuguuauguucuauaaggcccgagagcguucugacggauccguuucc

....gagagcguucugacggGu........	5	1	seq
....gagagcguucugacAgau........	2	1	seq
....gagagcguucGgacggau........	1	1	seq
....gagagUguucugacggau........	3	1	seq
....gagagcguucugacggUu........	2	1	seq
....gagagcguucugacggaC........	182	1	seq
....gagagcguucugacggaG........	20	1	seq
....gagaAcguucugacggauc.......	10	1	seq
....gagagcguucugacggauG.......	1	1	seq
....gagagcguucugacggauA.......	4	1	seq
....gagagcguucugacggauU.......	5	1	seq
....gagagcguucugacggauc.......	8	0	seq
....agaAcguucugacggau........	1066	1	seq
....agagcAuucugacggau........	2	1	seq
....agagcguucugGcggau........	5	1	seq
....agagcguuGugacggau........	2	1	seq
....agagcgCucugacggau........	1	1	seq
....Cgagcguucugacggau........	1	1	seq
....agagcguucugacggUu........	1	1	seq
....agagcguucugacggaG........	30	1	seq
....agagcguuAugacggau........	1	1	seq
....Ngagcguucugacggau........	3	1	seq
....agagcguucugacgCau........	1	1	seq
....agagcguucCgacggau........	5	1	seq
....agagcguucugacggau........	1406	0	seq
....agagcguucugacggaC........	196	1	seq
....aAagcguucugacggau........	1	1	seq
....agagcguucugacggGu........	12	1	seq
....agagcguucugacggaA........	11	1	seq
....agagcguCcugacggau........	4	1	seq
....agagGguucugacggau........	1	1	seq
....agagcguucugacggCu........	2	1	seq
....agagUguucugacggau........	3	1	seq
....agagcguucugaGggau........	1	1	seq
....agagcgGucugacggau........	1	1	seq
....agagcguucugacgAau........	2	1	seq
....agagcguucugaUggau........	1	1	seq
....agagcguAcugacggau........	2	1	seq
....agagcguucugacAgau........	1	1	seq
....Ggagcguucugacggau........	2	1	seq
....Ugagcguucugacggau........	1	1	seq
....agaAcguucugacggauc.......	5	1	seq
....agagcguucugacggauA.......	3	1	seq
....agagcguucugacggauc.......	11	0	seq
....agagcguucugacggauU.......	2	1	seq
....agaAcguucugacggaucc......	1	1	seq
....agagcguucugacggauAc......	1	1	seq
....gagcguucugacggauU.......	1	1	seq
....gagcguucugacggauc.......	1	0	seq
....agcguucugacggaucU......	1	1	seq
....agcguucugacggaucc......	1	0	seq
....guucugacggauccCuuu..	1	1	seq

Provisional ID : MA_10427405_227
Score total : 0.7
Score for star read(s) : -1.3
Score for read counts : 0
Score for mfe : 0.4
Score for randfold : 1.6
Score for cons. seed :
Total read count : 299
Mature read count : 298
Loop read count : 1
Star read count : 0

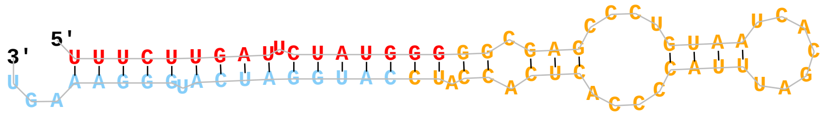

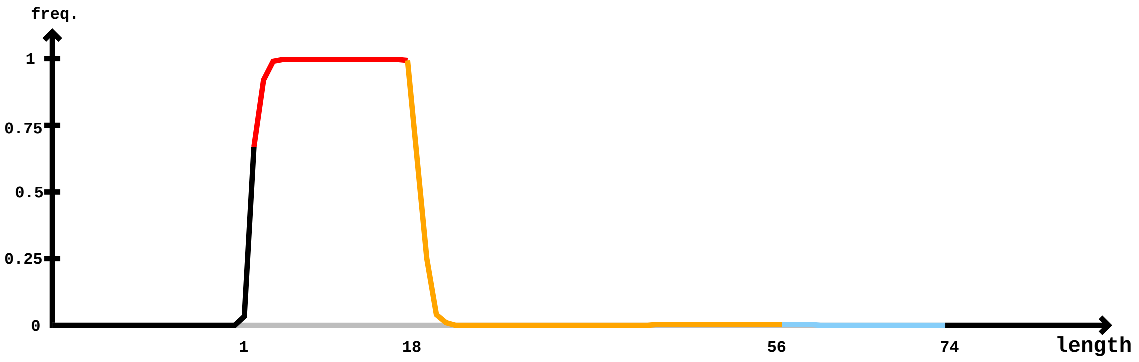

Mature Star

```
5'- uuaucaugcuuguccuuauuuucuugauucuaugggggcgagcccuguaaucacgauuuaccccacucaccauccauggaucaugggaaaguauucggagggucuann   -3'   exp
    .......((((.(((.((((((((((((.(((((((((.(((....((((.......))))....))).)).)))))))))))..))))))))...)))))))..... reads   mm   sample
    ..................uuuucuugGuucuaugg......................................................................... 1   1   seq
    ..................uuuucuugauucuaugggC....................................................................... 1   1   seq
    ..................uuuucuugauucuaugggUg...................................................................... 3   1   seq
    ..................Cuuucuugauucuauggggg...................................................................... 5   1   seq
    ...................Guucuugauucuauggg........................................................................ 1   1   seq
    ...................uuucuugauucuaGggg........................................................................ 2   1   seq
    ...................uuucuugauucuauggg........................................................................ 113   0   seq
    ...................uuucuAgauucuauggg........................................................................ 1   1   seq
    ...................uuucuugauucuaugggC....................................................................... 39   1   seq
    ...................uuucuugauucuaugggA....................................................................... 6   1   seq
    ...................uuucuugauucuaugggg....................................................................... 6   0   seq
    ...................uuucuugauucuauggggg...................................................................... 5   0   seq
    ...................uuucuugauucuaugggCg...................................................................... 11   1   seq
    ...................uuucuugauucuaugggAg...................................................................... 1   1   seq
    ...................uuucuugauucuauggggU...................................................................... 1   1   seq
    ...................uuucuugauucuauggggA...................................................................... 1   1   seq
    ...................uuucuugauucuaugggggc..................................................................... 1   0   seq
    ...................uuucuugauucuaugggggcg.................................................................... 1   0   seq
    ...................uuucuugauucuaugggggGg.................................................................... 1   1   seq
    ....................uucuugauucuaugggg....................................................................... 13   0   seq
    ....................uucuugauucuaugggA....................................................................... 4   1   seq
    ....................uucuugauucuaugggC....................................................................... 36   1   seq
    ....................uucuugauucuauggggg...................................................................... 8   0   seq
    ....................uucuugauucuaugggAg...................................................................... 3   1   seq
    ....................uucuugauucuaugggCg...................................................................... 7   1   seq
    ....................uucuugauucuaugggggG..................................................................... 3   1   seq
    ....................uucuugauucuaugggggA..................................................................... 1   1   seq
    .....................ucuugauucuaugggAg...................................................................... 3   1   seq
    .....................ucuugauucuauggggg...................................................................... 6   0   seq
    .....................ucuugauucuaugggCg...................................................................... 8   1   seq
    .....................ucuugauucuauggCgg...................................................................... 1   1   seq
    .....................ucuugauucuaugggggG..................................................................... 3   1   seq
    ......................cuugauucuaugggggG..................................................................... 1   1   seq
    ......................cuugauucuauggggUcg.................................................................... 1   1   seq
```

Mature Star

```
uuaucaugcuuguccuuauuuucuugauucuaugggggcgagcccuguaaucacgauuuaccccacucaccauccauggaucaugggaaaguauucggagggucuann
.............................................................cccacucaccauccauC..............................	1	1	seq
```

Provisional ID : MA_10427485_8045
Score total : 2.9
Score for star read(s) : -1.3
Score for read counts : 0
Score for mfe : 2.6
Score for randfold : 1.6
Score for cons. seed :
Total read count : 117
Mature read count : 116
Loop read count : 0
Star read count : 1

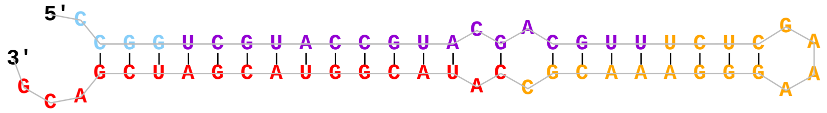

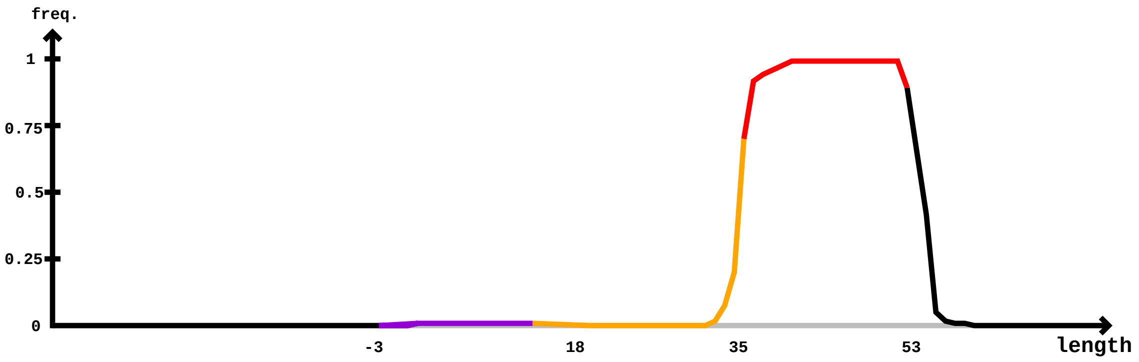

Star Mature

```
5'- ggaaauggguacgguacgaccgagaaucguugccggucguaccguacgacguuucucgaaagggaaacgccauacgguacgaucgacggcguuucccggucguacggu  -3'  obs
    ggaaauggguacgguacgaccgagaaucguugccggucguaccguacgacguuucucgaaagggaaacgccauacgguacgaucgacggcguuucccggucguacggu        exp
    ..........((.(((((((((.(((.(((((.(((((((((((((.(.((((((((....)))))))).).)))))))))))))).))))).))).))))))))).))   reads   mm   sample
    ....................................ucguaUcguacgacguu.......................................................   1       1    seq
    ...................................................................Ugccauacgguacgaucgacg....................   2       1    seq
    ....................................................................gccauacgguacgaucgac.....................   1       0    seq
    ....................................................................gccauacgguacgaucgacg....................   6       0    seq
    .....................................................................ccauacgguacgaucgac.....................   2       0    seq
    .....................................................................ccauacgguacgaucgacg....................   5       0    seq
    .....................................................................ccauacgguacgaucgacgU...................   1       1    seq
    .....................................................................ccauacgguacgaucgacgCc..................   1       1    seq
    .....................................................................ccauacgguacgaucgacgUc..................   6       1    seq
    ......................................................................cauacgguacgaucgac.....................   9       0    seq
    ......................................................................cauacgguacgaucgacA....................   2       1    seq
    ......................................................................cauacgguacgaucgacg....................   21      0    seq
    ......................................................................cauacgguacgaucgGcgg...................   1       1    seq
    ......................................................................cauacgguacgaucgacgC...................   1       1    seq
    ......................................................................cauacgguacgaucgacgU...................   4       1    seq
    ......................................................................cauacgguacgaucgacgUc..................   20      1    seq
    ......................................................................cauacgguacgaucgacgUcg.................   2       1    seq
    .......................................................................auacgguacgaucgacU....................   1       1    seq
    .......................................................................Guacgguacgaucgacg....................   1       1    seq
    .......................................................................auacgguacgaucgacg....................   10      0    seq
    .......................................................................auacgguacgaucgacgU...................   1       1    seq
    .......................................................................auacgguacgaucgacUg...................   1       1    seq
    .......................................................................auacgguacgaucgacgUc..................   12      1    seq
    ........................................................................uacgguacgaucgacgUc..................   2       1    seq
    ........................................................................uacgguacgaCcgacggc..................   1       1    seq
    .........................................................................acgguacgaucgacgUc..................   1       1    seq
    .........................................................................acgguacgaCcgacggc..................   1       1    seq
    ..........................................................................cgguacgaCcgacggcg.................   1       1    seq
    ..........................................................................cgguacgGucgacggcg.................   1       1    seq
    ...........................................................................gguacgaucgacgAcgu................   1       1    seq
    ...........................................................................gguacgGucgacggcguuu..............   1       1    seq
```

Provisional ID	:	MA_10428436_15219
Score total	:	1.6
Score for star read(s)	:	-1.3
Score for read counts	:	0
Score for mfe	:	1.3
Score for randfold	:	1.6
Score for cons. seed	:	
Total read count	:	1102
Mature read count	:	1102
Loop read count	:	0
Star read count	:	0

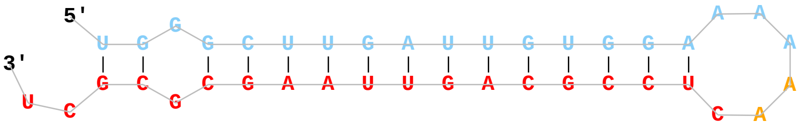

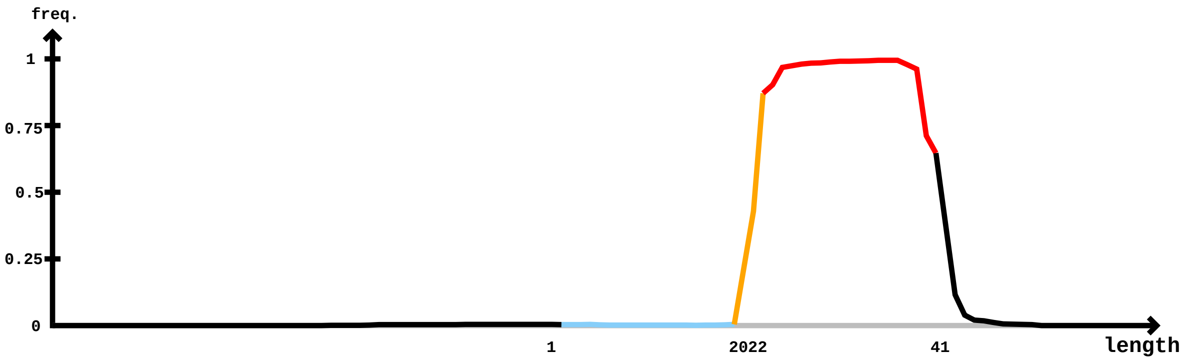

Star Mature

```
5'- nnnnnnnnnnnnnnnnnnnnnnnnnnuuugcaaaugggguagcaagcuaagugggcuugauuguggaaaaaacuccgcaguuaagcgcgcuugagccagaagcgucuugggau -3'   exp
    ...........................((.(((..((.((..(..((((((((.(((((((((((((......))))))))))))).)))))).)))..)..)).))))).)).    reads   mm   sample
    ...........................uugAaaaugggguagcaagcuaag..............................................................    1       1    seq
    ...............................aaaCgggguagcaagcuaaguggg..........................................................    1       1    seq
    ................................aaugggguagcaagcuaaguggg..........................................................    1       0    seq
    ................................aaugggguagGaagcuaagugggc.........................................................    1       1    seq
    .........................................gcaagcuaaguggAcuugauugug................................................    1       1    seq
    ......................................................gcuugauuguggaaaaaacucc.....................................    1       0    seq
    ..................................................................aaaGaacuccgcaguuaagcgcgcu......................    1       1    seq
    ....................................................................aaaacuccgcaguuaagcA..........................    1       1    seq
    .....................................................................Caacuccgcaguuaagcgcg........................    1       1    seq
    .....................................................................aaacuccgcaguuaagcgcgcuugGgcc................    1       1    seq
    ......................................................................aacuccgcaguuaagcg..........................    16      0    seq
    ......................................................................Nacuccgcaguuaagcg..........................    1       1    seq
    ......................................................................aacuccgcaguuaagcgc.........................    7       0    seq
    ......................................................................aacuccgcUguuaagcgc.........................    1       1    seq
    ......................................................................aacuccgcaguuaagcgcg........................    43      0    seq
    ......................................................................aacCccgcaguuaagcgcg........................    1       1    seq
    ......................................................................aacuccgcaguuaagcgcgc.......................    16      0    seq
    ......................................................................aacuccgcaguuaagcgcCc.......................    1       1    seq
    ......................................................................aacuccgcaguuaagcgcgcA......................    2       1    seq
    ......................................................................aacuccgGaguuaagcgcgcu......................    1       1    seq
    ......................................................................aacuccgcaguuaagcgcgcG......................    2       1    seq
    ......................................................................aacuccgcaguuaagcgcgcu......................    53      0    seq
    ......................................................................aacuccgcaguuaagcgcgcuu.....................    10      0    seq
    ......................................................................aacuccgcaguuaagcgcgcuug....................    17      0    seq
    ......................................................................aacuccgcaguuaagcgcgcuugC...................    1       1    seq
    ......................................................................aacuccgcaguuaagcgcgcuuga...................    2       0    seq
    .......................................................................acuccgcaguuaagcgc.........................    9       0    seq
    .......................................................................acuccgcaguuaagcgA.........................    2       1    seq
    .......................................................................acuccgcaguuaagcgcg........................    107     0    seq
    .......................................................................acCccgcaguuaagcgcg........................    1       1    seq
    .......................................................................acuccgcaguuGagcgcg........................    2       1    seq
    .......................................................................acuccgcaguGaagcgcg........................    1       1    seq
    .......................................................................Ucuccgcaguuaagcgcgc.......................    1       1    seq
    .......................................................................acuccgcUguuaagcgcgc.......................    1       1    seq
```

Star Mature

```
nnnnnnnnnnnnnnnnnnnnnnnnnnuuugcaaaugggguagcaagcuaagugggcuugauuguggaaaaaacuccgcaguuaagcgcgcuugagccagaagcgucuugggau
......................................................................acuccgcaguuaagcgcgc........................    17    0    seq
......................................................................acuccgcGguuaagcgcgcu.......................    1     1    seq
......................................................................acuccgcaguuaagcgcgcG.......................    3     1    seq
......................................................................Ncuccgcaguuaagcgcgcu.......................    1     1    seq
......................................................................acCccgcaguuaagcgcgcu.......................    1     1    seq
......................................................................Gcuccgcaguuaagcgcgcu.......................    2     1    seq
......................................................................acuccgcaguuaCgcgcgcu.......................    1     1    seq
......................................................................acuccgcaguuaagcgcgcu.......................    96    0    seq
......................................................................acuccgcaguuaagcgcgcA.......................    4     1    seq
......................................................................acuccgcaguuaagcgcgcuu......................    27    0    seq
......................................................................acuccgcaguuaagcgcgcuG......................    1     1    seq
......................................................................acuccgcUguuaagcgcgcuu......................    1     1    seq
......................................................................acuccgcaguuaagcgcgcuug.....................    16    0    seq
......................................................................acuccgcaguuaagcCcgcuug.....................    1     1    seq
......................................................................Ncuccgcaguuaagcgcgcuug.....................    1     1    seq
......................................................................acuccgcaguuaagcgcgcuuga....................    4     0    seq
......................................................................acuccgcaguuaagcgcgcuugUg...................    1     1    seq
......................................................................acuccgcaguuaagcgcgcuugCgc..................    1     1    seq
.......................................................................cuccgcaguuaaUcgcg.........................    1     1    seq
.......................................................................cuccgcaguuaagcgAg.........................    1     1    seq
.......................................................................cuccgcaguuaagcgcg.........................    119   0    seq
.......................................................................cuccgcagCuaagcgcg.........................    1     1    seq
.......................................................................cuccgGaguuaagcgcg.........................    1     1    seq
.......................................................................cuccgcaguuaagcgcC.........................    2     1    seq
.......................................................................cuccgcaguuaagcgcgc........................    37    0    seq
.......................................................................Nuccgcaguuaagcgcgcu.......................    1     1    seq
.......................................................................cuccgcaguuaagcgcgcA.......................    6     1    seq
.......................................................................cuccgcaguuaagcgcgcG.......................    9     1    seq
.......................................................................cuccgcaguuaaCcgcgcu.......................    1     1    seq
.......................................................................cAccgcaguuaagcgcgcu.......................    1     1    seq
.......................................................................cuccgcaguuaagcgcgcu.......................    207   0    seq
.......................................................................cuccgcaguuaagcgcgcAu......................    1     1    seq
.......................................................................cuccgcaguuaagcgcgcuu......................    64    0    seq
.......................................................................cuccgcaguuaagcgcgcuG......................    2     1    seq
.......................................................................cuccCcaguuaagcgcgcuug.....................    1     1    seq
.......................................................................cuccgcaguuaagcgcgcGug.....................    1     1    seq
.......................................................................cCccgcaguuaagcgcgcuug.....................    1     1    seq
.......................................................................cuccgcaguuaagcgcgcuug.....................    26    0    seq
.......................................................................cuccgcaguuaagcgcgcuuga....................    3     0    seq
.......................................................................cuccgcaguuaagcgcgcuugU....................    4     1    seq
.......................................................................cuccgcaguuaagcgcgcuugC....................    2     1    seq
.......................................................................cuccgcaguuaagcgcgcuugCg...................    1     1    seq
.......................................................................cuccgcaguuaagcgcgcuugCgc..................    1     1    seq
.......................................................................cuccgcaguuaagcgcgcuugagc..................    1     0    seq
.......................................................................cuccgcaguuaagcgcgcuugagA..................    1     1    seq
.......................................................................cuccgcaguuaagcgcgcuugagcc.................    1     0    seq
........................................................................uccgcaguuaagcgcgcA.......................    2     1    seq
........................................................................Accgcaguuaagcgcgcu.......................    1     1    seq
........................................................................uccgcaguuaagcgcgcG.......................    3     1    seq
........................................................................uccgcaguuaagcgcgcu.......................    22    0    seq
........................................................................uccgcaguuaagcgcgcuu......................    3     0    seq
........................................................................uccgcaguuaagcCcgcuu......................    1     1    seq
........................................................................uccgcaguuaagcgcgcuug.....................    4     0    seq
.........................................................................ccgcaguuaagcgcgcG.......................    2     1    seq
.........................................................................ccgcaguuaagcgcgcu.......................    37    0    seq
.........................................................................ccgcaguuaagcgcgcuu......................    20    0    seq
.........................................................................ccgcaguuaagcgcgcuG......................    1     1    seq
.........................................................................ccgcaguuaagcgcgcuuC.....................    1     1    seq
.........................................................................ccgcaguuaagcgcgcuug.....................    8     0    seq
.........................................................................ccgcaguuaagcgcgcuuga....................    1     0    seq
.........................................................................ccgcaguuaagcgcgcuugCgc..................    1     1    seq
.........................................................................ccgcaguuaagcgcgcuugagc..................    1     0    seq
.........................................................................ccgcaguuaagcgcgcuugagcc.................    1     0    seq
..........................................................................cgcaguuaagcgcgcuu......................    6     0    seq
..........................................................................cgcaguuaagcgcgcuug.....................    1     0    seq
...........................................................................gcaguuaagcgcgcuug.....................    8     0    seq
............................................................................caguuaagcgcgcuugU....................    1     1    seq
............................................................................caguuaagcgcgcuuga....................    3     0    seq
.............................................................................aguuaagcgcgcuugUg...................    1     1    seq
..............................................................................guuaagcgcgcuugUgc..................    1     1    seq
```

Star Mature

```
nnnnnnnnnnnnnnnnnnnnnnnnnnuuugcaaaugggguagcaagcuaagugggcuugauuguggaaaaaacuccgcaguuaagcgcgcuugagccagaagcgucuugggau
...............................................................................guuaagcgcgcuugagcc................    1    0    seq
...............................................................................guuaagcgcgcuugCgcca...............    1    1    seq
...............................................................................guuaagcgcgcuugGgccagaa............    1    1    seq
................................................................................uuaagcgcgcuugCgcc................    1    1    seq
................................................................................uuaagcgcgcuugagcA................    1    1    seq
................................................................................uuaagcgcgcuugagcUagaa............    1    1    seq
..................................................................................aagcgcgcuugagccag..............    1    0    seq
...................................................................................agcgcgcuugagccaga.............    1    0    seq
....................................................................................gcgcgcuugGgccagaa............    2    1    seq
```

Provisional ID	:	MA_10429402_6104
Score total	:	2.2
Score for star read(s)	:	-1.3
Score for read counts	:	0
Score for mfe	:	1.9
Score for randfold	:	1.6
Score for cons. seed	:	
Total read count	:	24189
Mature read count	:	24189
Loop read count	:	0
Star read count	:	0

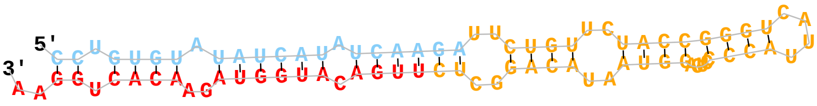

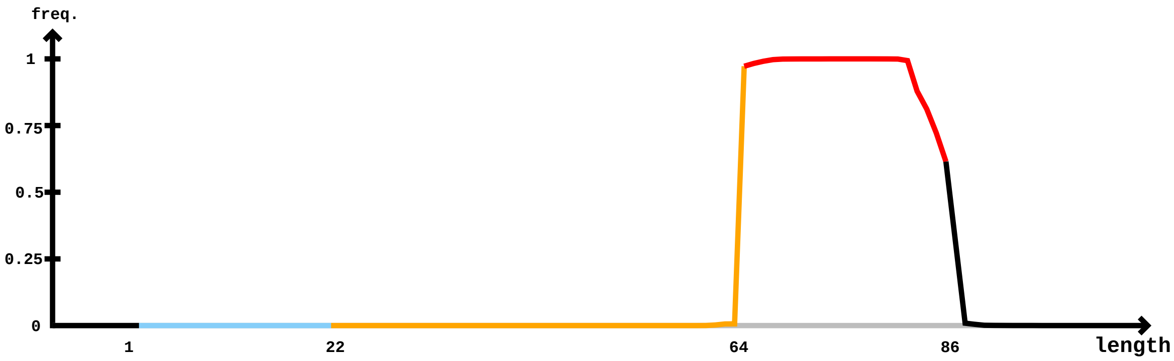

Star Mature

```
5'- ggggcuuccuguguauaucauaucaagauucuguucuaccgggucauuaccccugagguaauacaggcucuugacaugguagaacacuggaacccuccuccaugcaaugaca -3' exp
    ((((.((((.((((.((((((.((((((..((((..((((((((....))))....))))..))))..)))))).))))))..)))).))))))))................
```

	reads	mm	sample
....aUgcucuugacaugguagaaca..........................	1	1	seq
....gUucuugacaugguaga.............................	1	1	seq
....gUucuugacaugguagaaca..........................	3	1	seq
....Uucuugacaugguagaa............................	1	1	seq
....Uucuugacaugguagaac...........................	1	1	seq
....Uucuugacaugguagaaca..........................	4	1	seq
....Uucuugacaugguagaacac.........................	1	1	seq
....Uucuugacaugguagaacacu........................	14	1	seq
....Uucuugacaugguagaacacugg......................	2	1	seq
....Uucuugacaugguagaacacugga.....................	4	1	seq
....Uucuugacaugguagaacacuggaa....................	22	1	seq
....ucuugacaugguagaac...........................	1	0	seq
....ucuugacaugguagaaca..........................	3	0	seq
....ucuugacGugguagaacac.........................	1	1	seq
....ucuugacaugguagaacac.........................	1	0	seq
....ucuugacaugguagaacacu........................	3	0	seq
....ucuugacaugguagaacacug.......................	4	0	seq
....ucuugacaugguagaacacuA.......................	1	1	seq
....uUuugacaugguagaacacugg......................	5	1	seq
....ucuugacaugguagaacacugA......................	1	1	seq
....ucuCgacaugguagaacacugg......................	1	1	seq
....ucuugacaugguagaacacugg......................	44	0	seq
....ucuugacaugguagaacacugC......................	2	1	seq
....ucuugacaugguagaacacugga.....................	4	0	seq
....ucuugacaugguagaacacuggaa....................	21	0	seq
....Uuugacaugguagaacacu........................	1	1	seq
....cuuUacaugguagaacacugg......................	1	1	seq
....Uuugacaugguagaacacugga.....................	4	1	seq
....cuugacaugguagaacacugga.....................	2	0	seq
....uugacaugguagaacaA.........................	1	1	seq
....uugacaugguagaacac.........................	132	0	seq
....uugacaugguagaacCc.........................	1	1	seq
....uugacaugUuagaacac.........................	1	1	seq
....uugacUugguagaacac.........................	1	1	seq

Star Mature

```
ggggcuuccuguguauaucauaucaagauucuguucuaccgggucauuaccccugagguaauacaggcucuugacaugguagaacacuggaacccuccuccaugcaaugaca
......................................................................uugacGugguagaacac.........................    2      1    seq
......................................................................Augacaugguagaacac.........................    1      1    seq
......................................................................uuAacaugguagaacacu........................    2      1    seq
......................................................................Nugacaugguagaacacu........................    7      1    seq
......................................................................uugacaugguagaaUacu........................    1      1    seq
......................................................................uugacaCgguagaacacu........................    4      1    seq
......................................................................uCgacaugguagaacacu........................    10     1    seq
......................................................................uugacaugguagaacacA........................    42     1    seq
......................................................................Cugacaugguagaacacu........................    8      1    seq
......................................................................uugacaugCuagaacacu........................    1      1    seq
......................................................................uugacaugguagCacacu........................    1      1    seq
......................................................................uugacaGgguagaacacu........................    1      1    seq
......................................................................Gugacaugguagaacacu........................    6      1    seq
......................................................................uugacaAgguagaacacu........................    1      1    seq
......................................................................uugacauggCagaacacu........................    7      1    seq
......................................................................uugGcaugguagaacacu........................    8      1    seq
......................................................................uugacaugguagaacGcu........................    7      1    seq
......................................................................uugacauggAagaacacu........................    2      1    seq
......................................................................uAgacaugguagaacacu........................    3      1    seq
......................................................................uugacaugguagaUcacu........................    2      1    seq
......................................................................uugacUugguagaacacu........................    2      1    seq
......................................................................uugacaugguagaGcacu........................    5      1    seq
......................................................................uugacaugguagaacaAu........................    1      1    seq
......................................................................uugCcaugguagaacacu........................    1      1    seq
......................................................................uugacaugAuagaacacu........................    1      1    seq
......................................................................uugacaugguagaacacu........................    2227   0    seq
......................................................................uugacaugguagaacacC........................    370    1    seq
......................................................................uugacaugUuagaacacu........................    1      1    seq
......................................................................Augacaugguagaacacu........................    1      1    seq
......................................................................uugacGugguagaacacu........................    9      1    seq
......................................................................uugacaugguUgaacacu........................    2      1    seq
......................................................................uugacaugguagaacacAg.......................    6      1    seq
......................................................................uugacaugguagaacacug.......................    1310   0    seq
......................................................................uugacaugguagaGcacug.......................    5      1    seq
......................................................................Cugacaugguagaacacug.......................    4      1    seq
......................................................................uugacaugguagaacacuC.......................    9      1    seq
......................................................................uugacUugguagaacacug.......................    1      1    seq
......................................................................uugacaugguagaacGcug.......................    6      1    seq
......................................................................uugacauggAagaacacug.......................    1      1    seq
......................................................................uugacauggCagaacacug.......................    3      1    seq
......................................................................uugaGaugguagaacacug.......................    1      1    seq
......................................................................uugacauAguagaacacug.......................    1      1    seq
......................................................................uuAacaugguagaacacug.......................    1      1    seq
......................................................................uugacaAgguagaacacug.......................    3      1    seq
......................................................................uugacaugguGgaacacug.......................    5      1    seq
......................................................................uugacaugguCgaacacug.......................    1      1    seq
......................................................................uugacaugguagaacacuU.......................    66     1    seq
......................................................................Augacaugguagaacacug.......................    1      1    seq
......................................................................uugacaugguagaacacCg.......................    11     1    seq
......................................................................uugGcaugguagaacacug.......................    4      1    seq
......................................................................uugacaCgguagaacacug.......................    2      1    seq
......................................................................uugacGugguagaacacug.......................    5      1    seq
......................................................................uugUcaugguagaacacug.......................    1      1    seq
......................................................................uugacaugguagaacacuA.......................    57     1    seq
......................................................................Gugacaugguagaacacug.......................    1      1    seq
......................................................................uCgacaugguagaacacug.......................    6      1    seq
......................................................................uugacaugguagGacacug.......................    7      1    seq
......................................................................uugacaugguagaacacGg.......................    5      1    seq
......................................................................Nugacaugguagaacacug.......................    1      1    seq
......................................................................uuUacaugguagaacacug.......................    1      1    seq
......................................................................uugCcaugguagaacacug.......................    1      1    seq
......................................................................uugacaugguagaacacugA......................    115    1    seq
......................................................................uugGcaugguagaacacugg......................    2      1    seq
......................................................................uugacaugguagaacGcugg......................    9      1    seq
......................................................................uugacaugguaCaacacugg......................    1      1    seq
......................................................................uugacaugguagaacacCgg......................    4      1    seq
......................................................................uugacaugguagaGcacugg......................    6      1    seq
......................................................................uugacGugguagaacacugg......................    4      1    seq
......................................................................Augacaugguagaacacugg......................    1      1    seq
......................................................................uugacaAgguagaacacugg......................    1      1    seq
```

```
       Star                                                                Mature
ggggcuuccuguguauaucauaucaagauucuguucuaccgggucauuaccccugagguaaauacaggcucuugacaugguagaacacuggaacccuccuccaugcaaugaca
.......................................................................uugacaugguagaacacGgg......................    4     1     seq
.......................................................................uugacaugguagaaUacugg......................    1     1     seq
.......................................................................uuAacaugguagaacacugg......................    1     1     seq
.......................................................................uugacauggGagaacacugg......................    2     1     seq
.......................................................................uCgacaugguagaacacugg......................    4     1     seq
.......................................................................Nugacaugguagaacacugg......................    3     1     seq
.......................................................................uugacauUguagaacacugg......................    1     1     seq
.......................................................................uugacaugguagaacacugg......................    1733  0     seq
.......................................................................uugacaugguagaacacugU......................    21    1     seq
.......................................................................uugacaugguagaaGacugg......................    2     1     seq
.......................................................................uugacaugguagaacacugC......................    21    1     seq
.......................................................................uugacaCgguagaacacugg......................    3     1     seq
.......................................................................uugacaugguagUacacugg......................    1     1     seq
.......................................................................uugacaugguagaacacuAg......................    8     1     seq
.......................................................................Cugacaugguagaacacugg......................    4     1     seq
.......................................................................uugacauggu Ggaacacugg......................    5     1     seq
.......................................................................uugacaugguagaacacAgg......................    1     1     seq
.......................................................................uugacaugguCgaacacugg......................    1     1     seq
.......................................................................uugacaugguagaacacuUg......................    2     1     seq
.......................................................................uugacaugguagGacacugg......................    3     1     seq
.......................................................................uugacauggCagaacacugg......................    8     1     seq
.......................................................................Gugacaugguagaacacugg......................    1     1     seq
.......................................................................uugacauAguagaacacugg......................    1     1     seq
.......................................................................uugacauAguagaacacugga.....................    1     1     seq
.......................................................................uugacaugguagaacacuggC.....................    5     1     seq
.......................................................................uugacaugguagaacacuCga.....................    2     1     seq
.......................................................................uugGcaugguagaacacugga.....................    7     1     seq
.......................................................................uugacaCgguagaacacugga.....................    5     1     seq
.......................................................................uugacaugguagaacacugga.....................    2231  0     seq
.......................................................................Augacaugguagaacacugga.....................    3     1     seq
.......................................................................uugacaugguagaacaAugga.....................    1     1     seq
.......................................................................uugacaugguagaUcacugga.....................    3     1     seq
.......................................................................uugacaugAuagaacacugga.....................    1     1     seq
.......................................................................uugacaugguagGacacugga.....................    4     1     seq
.......................................................................uugacaugguagaacacugUa.....................    1     1     seq
.......................................................................uugacaugguagaacacAgga.....................    3     1     seq
.......................................................................uugacaugguagaacacuAga.....................    1     1     seq
.......................................................................uugacaugguagaacCcugga.....................    1     1     seq
.......................................................................uugacaugguagaacGcugga.....................    10    1     seq
.......................................................................Nugacaugguagaacacugga.....................    4     1     seq
.......................................................................uugacaugguagaacacuggG.....................    77    1     seq
.......................................................................uugacaugguagaacacuggU.....................    19    1     seq
.......................................................................uCgacaugguagaacacugga.....................    8     1     seq
.......................................................................uuUacaugguagaacacugga.....................    2     1     seq
.......................................................................uugacaugguagaGcacugga.....................    6     1     seq
.......................................................................Cugacaugguagaacacugga.....................    6     1     seq
.......................................................................uugacaugguagaacacCgga.....................    4     1     seq
.......................................................................uugacaugguagUacacugga.....................    2     1     seq
.......................................................................uugacCugguagaacacugga.....................    1     1     seq
.......................................................................Gugacaugguagaacacugga.....................    3     1     seq
.......................................................................uugacauggCagaacacugga.....................    6     1     seq
.......................................................................uugacaugguGgaacacugga.....................    7     1     seq
.......................................................................uugacaugguagaacaUugga.....................    1     1     seq
.......................................................................uAgacaugguagaacacugga.....................    1     1     seq
.......................................................................uugacaugguagCacacugga.....................    1     1     seq
.......................................................................uugacaugguagaacacugAa.....................    6     1     seq
.......................................................................uugacaugguagaacacugCa.....................    4     1     seq
.......................................................................uugacGugguagaacacugga.....................    10    1     seq
.......................................................................uugacUugguagaacacuggaa....................    2     1     seq
.......................................................................uugacGugguagaacacuggaa....................    47    1     seq
.......................................................................uugacaugguagaacacuCgaa....................    3     1     seq
.......................................................................uugacaugguCgaacacuggaa....................    2     1     seq
.......................................................................uuUacaugguagaacacuggaa....................    2     1     seq
.......................................................................uugacaugguagaacUcuggaa....................    2     1     seq
.......................................................................uugacaugguUgaacacuggaa....................    7     1     seq
.......................................................................uugacaugguagaaUacuggaa....................    7     1     seq
.......................................................................uugacaugguagaacacugAaa....................    7     1     seq
.......................................................................uugacaugguagaacacGggaa....................    5     1     seq
.......................................................................uugacaugUuagaacacuggaa....................    4     1     seq
.......................................................................uugacauggAagaacacuggaa....................    6     1     seq
```

```
                        Star                                                          Mature
ggggcuuccuguguauaucauaucaagauucuguucuaccgggucauuaccccugagguaauacaggcucuugacaugguagaacacuggaacccuccuccaugcaaugaca
......................................................................uugacauggCagaacacuggaa....................    36       1    seq
......................................................................uugGcaugguagaacacuggaa....................    56       1    seq
......................................................................uugacaugguagCacacuggaa....................    1        1    seq
......................................................................uugacaugguagaacGcuggaa....................    36       1    seq
......................................................................Augacaugguagaacacuggaa....................    14       1    seq
......................................................................uugacaCgguagaacacuggaa....................    23       1    seq
......................................................................uugacaugAuagaacacuggaa....................    11       1    seq
......................................................................uugacaugguagaacacuUgaa....................    3        1    seq
......................................................................uugacaugguagaacacAggaa....................    7        1    seq
......................................................................Cugacaugguagaacacuggaa....................    39       1    seq
......................................................................uGgacaugguagaacacuggaa....................    2        1    seq
......................................................................uugacauggGagaacacuggaa....................    3        1    seq
......................................................................uugacaugguGgaacacuggaa....................    41       1    seq
......................................................................uugacaugguagaacacuggaU....................    62       1    seq
......................................................................uugUcaugguagaacacuggaa....................    10       1    seq
......................................................................uugacaugguaUaacacuggaa....................    1        1    seq
......................................................................uugacaugguagaacacuAgaa....................    10       1    seq
......................................................................uugacaugguagaacacugUaa....................    3        1    seq
......................................................................uAgacaugguagaacacuggaa....................    2        1    seq
......................................................................uugacauUguagaacacuggaa....................    3        1    seq
......................................................................Gugacaugguagaacacuggaa....................    13       1    seq
......................................................................uugacaugguagaacacCggaa....................    31       1    seq
......................................................................uugacaugguagaacacuggaa....................    13172    0    seq
......................................................................uugCcaugguagaacacuggaa....................    2        1    seq
......................................................................uugacaugguagaaAacuggaa....................    2        1    seq
......................................................................uugacaugguagaacacuggUa....................    11       1    seq
......................................................................uugacaugguagGacacuggaa....................    46       1    seq
......................................................................uugaGaugguagaacacuggaa....................    4        1    seq
......................................................................Nugacaugguagaacacuggaa....................    19       1    seq
......................................................................uugacauAguagaacacuggaa....................    10       1    seq
......................................................................uugacaugguagaacaUuggaa....................    4        1    seq
......................................................................uugacaugguagaacacugCaa....................    3        1    seq
......................................................................uugacaugguagaacacuggGa....................    105      1    seq
......................................................................uugacCugguagaacacuggaa....................    2        1    seq
......................................................................uugacaugguagaaGacuggaa....................    2        1    seq
......................................................................uugacaugguagaUcacuggaa....................    3        1    seq
......................................................................uugaAaugguagaacacuggaa....................    2        1    seq
......................................................................uugacaAgguagaacacuggaa....................    7        1    seq
......................................................................uugacaugguagaacacuggCa....................    5        1    seq
......................................................................uugacaugguagUacacuggaa....................    3        1    seq
......................................................................uugacaugguagaGcacuggaa....................    43       1    seq
......................................................................uugacaugguagaCcacuggaa....................    2        1    seq
......................................................................uugacaugguagaacaAuggaa....................    3        1    seq
......................................................................uugacaugguagaacCcuggaa....................    2        1    seq
......................................................................uugacaugguagaacacuggaG....................    230      1    seq
......................................................................uuAacaugguagaacacuggaa....................    7        1    seq
......................................................................uCgacaugguagaacacuggaa....................    47       1    seq
......................................................................uugacaugCuagaacacuggaa....................    1        1    seq
......................................................................uugacaugguagaacacuggaC....................    7        1    seq
......................................................................uugacaugguagaacGcuggaac...................    1        1    seq
......................................................................uugacaugguagaaAacuggaac...................    1        1    seq
......................................................................uugacaugguagaacacuggaaU...................    104      1    seq
......................................................................uuAacaugguagaacacuggaac...................    1        1    seq
......................................................................uugacaugguagaacacuggaUc...................    5        1    seq
......................................................................uugacaugguagaacacuggaCc...................    1        1    seq
......................................................................uugacaugguagaacacuggaaA...................    114      1    seq
......................................................................uugacaugguagaacacuggaac...................    50       0    seq
......................................................................uugacaugguagaacacuggaaG...................    13       1    seq
......................................................................uugacaugguagaacacuggaacA..................    6        1    seq
......................................................................uugacaugguagaacacuggaacc..................    17       0    seq
......................................................................uugacaugguagaacacuggaaAc..................    1        1    seq
......................................................................uugacaugguagaacacuggaaGc..................    2        1    seq
......................................................................uugacaugguagaacacuggaaUc..................    10       1    seq
......................................................................uugacaugguagaacacuggaUcc..................    1        1    seq
......................................................................uugacaugguagaacacuggaacU..................    10       1    seq
......................................................................uugacaugguagaacacuggaacAc.................    1        1    seq
......................................................................uugacaugguagaacacuggaaAcc.................    1        1    seq
......................................................................uugacaugguagaacacuggaaccG.................    1        1    seq
......................................................................uugacaugguagaacacuggaaUcc.................    3        1    seq
......................................................................uugacaugguagaacacuggaaccA.................    16       1    seq
```

```
       Star                                                       Mature
ggggcuuccuguguauaucauaucaagauucuguucuaccgggucauuaccccugagguaauacaggcucuugacaugguagaacacuggaacccuccuccaugcaaugaca
......................................................................uugacaugguagaacacuggaaccU.................   1     1     seq
......................................................................uugacaugguagaacacuggaacAcu................   1     1     seq
......................................................................uugacaugguagaacacuggaacUcu................   1     1     seq
......................................................................uugacaugguagaacacuggaacccGc...............   1     1     seq
......................................................................uugacaugguagaacacuggaacccucc..............   1     0     seq
......................................................................uugacaugguagaacacuggaacccucU..............   1     1     seq
.......................................................................ugacUugguagaacacu........................   1     1     seq
.......................................................................ugacaugguagaacacA........................   1     1     seq
.......................................................................ugacaugguagaacacu........................   19    0     seq
.......................................................................ugacaugguagaacacC........................   5     1     seq
.......................................................................ugacaugguagaacacuA.......................   2     1     seq
.......................................................................ugacaugguagaacacug.......................   45    0     seq
.......................................................................ugacaugguagaacacAg.......................   1     1     seq
.......................................................................ugacaugguCgaacacug.......................   1     1     seq
.......................................................................ugacaugguagaacacGg.......................   1     1     seq
.......................................................................ugacaugguagaacacugg......................   35    0     seq
.......................................................................ugacaugguagaacacugU......................   2     1     seq
.......................................................................Cgacaugguagaacacugga.....................   1     1     seq
.......................................................................ugacaugguagaacacuggG.....................   2     1     seq
.......................................................................ugacaugguagaacacugga.....................   52    0     seq
.......................................................................ugacaugguagaacacuggaG....................   1     1     seq
.......................................................................ugacaugguagaacacuggaa....................   18    0     seq
.......................................................................ugacaugguagaacacuggaaG...................   3     1     seq
.......................................................................ugacaugguagaacacuggaac...................   12    0     seq
.......................................................................ugacaugguagaacacuggaaA...................   1     1     seq
.......................................................................ugacaugguagaacacuggaaU...................   52    1     seq
........................................................................gacaugguagaacacuA.......................   1     1     seq
........................................................................gacaugguagaacacug.......................   41    0     seq
........................................................................gacaugguagaacacugg......................   100   0     seq
........................................................................gacaugguagaacacugA......................   3     1     seq
........................................................................gacaugguagaacaUugg......................   1     1     seq
........................................................................gacaugguagaacacuAg......................   2     1     seq
........................................................................Aacaugguagaacacugg......................   1     1     seq
........................................................................gacaugguagaacacCgg......................   1     1     seq
........................................................................gGcaugguagaacacugg......................   1     1     seq
........................................................................gacaugguagaacacugga.....................   39    0     seq
........................................................................gacaugguUgaacacugga.....................   1     1     seq
........................................................................gacaugguagaacacuggG.....................   1     1     seq
........................................................................gacaugguagaacacuggaa....................   6     0     seq
........................................................................gacaugguagaacacuggaCc...................   1     1     seq
.........................................................................acaugguagaacacugg......................   11    0     seq
.........................................................................acaugguagaacGcugg......................   1     1     seq
.........................................................................acaugguagaacacugA......................   1     1     seq
.........................................................................acaugguagaacGcugga.....................   1     1     seq
.........................................................................acaugguagaacacugga.....................   16    0     seq
.........................................................................Gcaugguagaacacuggaa....................   1     1     seq
.........................................................................acaugguagaacacuggaa....................   4     0     seq
.........................................................................acaugguagaacacuggaaA...................   1     1     seq
.........................................................................acaugguagaacacuggaac...................   1     0     seq
.........................................................................acaugguagaacacuggaaUc..................   2     1     seq
.........................................................................acaugguagaacacuggaacc..................   43    0     seq
.........................................................................acaugAuagaacacuggaacc..................   1     1     seq
.........................................................................acaugguagaacacuggaacA..................   2     1     seq
.........................................................................acaugguagaacacuggaacU..................   7     1     seq
.........................................................................Gcaugguagaacacuggaacc..................   2     1     seq
.........................................................................acaugguagaacGcuggaaccc.................   1     1     seq
.........................................................................acaugguagaacacuggaaccU.................   12    1     seq
.........................................................................acaugguagaacacuggaaccA.................   1     1     seq
.........................................................................acaugguagaacacuggaaccc.................   37    0     seq
.........................................................................acaugguagaacacuggaacccuU...............   1     1     seq
..........................................................................caugguagaacacugga.....................   17    0     seq
..........................................................................caugguagGacacugga.....................   1     1     seq
..........................................................................caugguagaacacuggaa....................   6     0     seq
..........................................................................caugguagaaUacuggaa....................   1     1     seq
..........................................................................caugguagaacacuggGa....................   1     1     seq
..........................................................................caugguagaacacuggaac...................   1     0     seq
..........................................................................caugguagaacacuggaaccU.................   3     1     seq
..........................................................................caugguagaacacuggaaccc.................   6     0     seq
..........................................................................caugguagaacacuggaaccA.................   1     1     seq
..........................................................................caugguagaacacuggaaUcc.................   2     1     seq
```

Star Mature

```
ggggcuuccuguguauaucauaucaagauucuguucuaccgggucauuaccccugagguaauacaggcucuugacaugguagaacacuggaacccuccuccaugcaaugaca
..........................................................................caugguagaacacuggaacccC................	1	1	seq
..........................................................................caugguagaacacuggaacccu................	3	0	seq
..........................................................................caugguagaacacuggGacccu................	1	1	seq
..........................................................................caugguagaacacuggaacccA................	1	1	seq
..........................................................................caugguagaacacuggaaUccu................	1	1	seq
..........................................................................caugguagaacacuggaaUccuc...............	4	1	seq
..........................................................................caugguagaacacuggaaUccucc..............	1	1	seq
...........................................................................augguagaacacuggGa....................	1	1	seq
...........................................................................augguagaacacuggaa....................	7	0	seq
...........................................................................augguagaacacuggaaUccu................	1	1	seq
............................................................................ugguagaacacuggaac...................	1	0	seq
............................................................................ugguagaacacuggaaUccu................	1	1	seq
............................................................................ugguagaacacuggaacccucU..............	2	1	seq
..............................................................................gCagaacacuggaacccu................	1	1	seq
...............................................................................uagaacacuggaacccuUcucc...........	4	1	seq
```

Provisional ID : MA_10429804_1042
Score total : 0.1
Score for star read(s) : -1.3
Score for read counts : 0
Score for mfe : -0.1
Score for randfold : 1.6
Score for cons. seed :
Total read count : 22745
Mature read count : 22744
Loop read count : 1
Star read count : 0

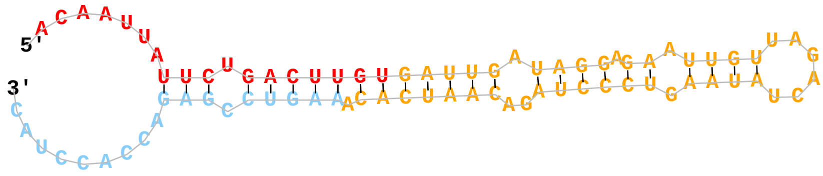

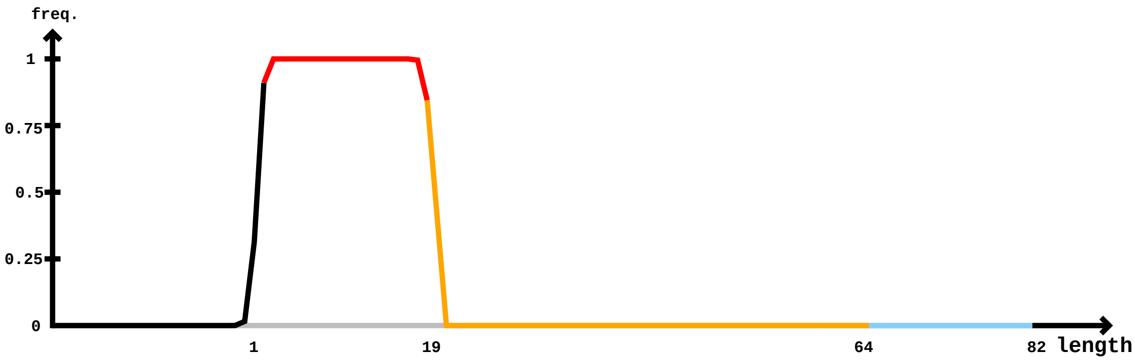

Mature **Star**

```
5'- guauuguuuccuuacuaguacaauuauucugacuugugauugauaggagaauuguuagacuauaagucccuagacaaucacaaaguccgagaccaccuacuuaugag   -3'    exp
    ...........((((..((((......(((.(((((((((((.((((.((.((((......)))).))))))..))))))).))))).)))......))))..))))   reads     mm     sample
    .................aUuacaauuauucugacuug......................................................................   1         1      seq
    .................Uguacaauuauucugacuug......................................................................   1         1      seq
    .................aUuacaauuauucugacuugu.....................................................................   2         1      seq
    ..................Uuacaauuauucugacuu.......................................................................   15        1      seq
    ..................Cuacaauuauucugacuug......................................................................   1         1      seq
    ..................Uuacaauuauucugacuug......................................................................   223       1      seq
    ..................guacaauuauucugacuug......................................................................   2         0      seq
    ..................Auacaauuauucugacuugu.....................................................................   1         1      seq
    ..................Nuacaauuauucugacuugu.....................................................................   2         1      seq
    ..................guacaauuauucugacuugu.....................................................................   1         0      seq
    ..................Uuacaauuauucugacuugu.....................................................................   109       1      seq
    ...................uacaauuauucugGcuu.......................................................................   1         1      seq
    ...................uacaauuauucugacuC.......................................................................   9         1      seq
    ...................uacaauuauucugacuu.......................................................................   77        0      seq
    ...................uacaauuauucCgacuug......................................................................   4         1      seq
    ...................uacaauuauucugUcuug......................................................................   1         1      seq
    ...................uacaauuauucugacuuA......................................................................   46        1      seq
    ...................Nacaauuauucugacuug......................................................................   1         1      seq
    ...................uacaauuauAcugacuug......................................................................   1         1      seq
    ...................uacaauuauCcugacuug......................................................................   2         1      seq
    ...................uacaauuauucugacCug......................................................................   5         1      seq
    ...................uGcaauuauucugacuug......................................................................   2         1      seq
    ...................uacaauuauucugacAug......................................................................   2         1      seq
    ...................uaAaauuauucugacuug......................................................................   2         1      seq
    ...................uacaauuauucugacuCg......................................................................   8         1      seq
    ...................uacaauuauucugaAuug......................................................................   2         1      seq
    ...................uacaGuuauucugacuug......................................................................   4         1      seq
    ...................uacaauuauucugaUuug......................................................................   1         1      seq
    ...................uacaauuaCucugacuug......................................................................   4         1      seq
    ...................uacaauuauucugacuGg......................................................................   9         1      seq
    ...................uacaauuauuAugacuug......................................................................   1         1      seq
    ...................uacaauCauucugacuug......................................................................   1         1      seq
    ...................Cacaauuauucugacuug......................................................................   2         1      seq
    ...................uacaaCuauucugacuug......................................................................   1         1      seq
```

```
                    Mature                                                Star
guuauuguuuccuuacuaguacaauuauucugacuugugauugauaggagaauuguuagacuauaagucccuagacaaucacaaaguccgagaccaccuacuuaugag
...................uacaauuauucugacuug.......................................................................	1213	0	seq
...................uacaauuauucugacuuU.......................................................................	9	1	seq
...................uacaauuaAucugacuug.......................................................................	1	1	seq
...................uacaauuauucugCcuug.......................................................................	1	1	seq
...................uacaauuauucugGcuug.......................................................................	6	1	seq
...................uacaauuauucuAacuug.......................................................................	2	1	seq
...................uacaUuuauucugacuug.......................................................................	1	1	seq
...................Aacaauuauucugacuug.......................................................................	2	1	seq
...................uacaauuauucugacuuC.......................................................................	6	1	seq
...................uacGauuauucugacuug.......................................................................	2	1	seq
...................uacaauuauucugacuAg.......................................................................	1	1	seq
...................uacCauuauucugacuug.......................................................................	1	1	seq
...................uUcaauuauucugacuug.......................................................................	1	1	seq
...................uacaauuauucugaGuug.......................................................................	1	1	seq
...................Nacaauuauucugacuugu......................................................................	2	1	seq
...................uacaauuauCcugacuugu......................................................................	1	1	seq
...................uacaauuauucugacuuAu......................................................................	1	1	seq
...................uacaauuauucugacuugu......................................................................	647	0	seq
...................uacGauuauucugacuugu......................................................................	3	1	seq
...................Gacaauuauucugacuugu......................................................................	1	1	seq
...................uacaauuUuucugacuugu......................................................................	1	1	seq
...................uacaaCuauucugacuugu......................................................................	2	1	seq
...................uacaauuauucugacuAgu......................................................................	1	1	seq
...................uacaauuauucugacuugG......................................................................	38	1	seq
...................uacaauuGuucugacuugu......................................................................	2	1	seq
...................uacaauuauucugacuugA......................................................................	336	1	seq
...................uacaauuauucugacuuUu......................................................................	4	1	seq
...................uacaauuauucugacuuCu......................................................................	2	1	seq
...................uacaauuauucCgacuugu......................................................................	3	1	seq
...................uacaauuauucugacuGgu......................................................................	2	1	seq
...................uacaauuauucuAacuugu......................................................................	1	1	seq
...................Cacaauuauucugacuugu......................................................................	3	1	seq
...................uacaauuauucugGcuugu......................................................................	2	1	seq
...................uacaauuaCucugacuugu......................................................................	2	1	seq
...................uacaauuauucugacAugu......................................................................	2	1	seq
...................uacaaAuauucugacuugu......................................................................	1	1	seq
...................uacaauuauucugacuugC......................................................................	4272	1	seq
...................uacaGuuauucugacuugu......................................................................	1	1	seq
...................uGcaauuauucugacuugu......................................................................	1	1	seq
...................uacaauuauucugacuugCg.....................................................................	17	1	seq
...................uacaauuauucugacuugAg.....................................................................	2	1	seq
...................uacaauuauucugacuugAga....................................................................	1	1	seq
....................acaauuauucugacuCg.......................................................................	18	1	seq
....................Ncaauuauucugacuug.......................................................................	2	1	seq
....................acaauuauucugCcuug.......................................................................	1	1	seq
....................acaauuauucugacuuC.......................................................................	10	1	seq
....................acaauuauucAgacuug.......................................................................	1	1	seq
....................acaGuuauucugacuug.......................................................................	3	1	seq
....................acaUuuauucugacuug.......................................................................	1	1	seq
....................acaauuauucugacCug.......................................................................	8	1	seq
....................acaauuauucugaUuug.......................................................................	1	1	seq
....................aGaauuauucugacuug.......................................................................	1	1	seq
....................acaauuGuucugacuug.......................................................................	1	1	seq
....................acaauuauuUugacuug.......................................................................	2	1	seq
....................acUauuauucugacuug.......................................................................	1	1	seq
....................acaauCauucugacuug.......................................................................	2	1	seq
....................acaauuauucCgacuug.......................................................................	11	1	seq
....................acaauuauucugacGug.......................................................................	2	1	seq
....................acaauuauucugacAug.......................................................................	1	1	seq
....................Ccaauuauucugacuug.......................................................................	1	1	seq
....................acaauuauucugacuGg.......................................................................	5	1	seq
....................acaauuauucugGcuug.......................................................................	4	1	seq
....................acGauuauucugacuug.......................................................................	7	1	seq
....................acaauuauucugacuug.......................................................................	1680	0	seq
....................Gcaauuauucugacuug.......................................................................	7	1	seq
....................acaauuauucugacuuA.......................................................................	86	1	seq
....................acaauuauucCgacuugu......................................................................	8	1	seq
....................acGauuauucugacuugu......................................................................	7	1	seq
....................acaauuauucugacuugG......................................................................	67	1	seq
....................Ncaauuauucugacuugu......................................................................	2	1	seq
```

Mature **Star**

```
guuauuguuuccuuacuaguacaauuauucugacuugugauugauaggagaauuguuagacuauaagucccuagacaaucacaaaguccgagaccaccuacuuaugag
....................Gcaauuauucugacuugu......................................................................  10     1  seq
....................Ucaauuauucugacuugu......................................................................  1      1  seq
....................Ccaauuauucugacuugu......................................................................  2      1  seq
....................acaauuauCcugacuugu......................................................................  4      1  seq
....................acaGuuauucugacuugu......................................................................  3      1  seq
....................acaauCauucugacuugu......................................................................  1      1  seq
....................acaauuauucugacCugu......................................................................  2      1  seq
....................acaauuauucugUcuugu......................................................................  2      1  seq
....................acaaGuauucugacuugu......................................................................  1      1  seq
....................acaUuuauucugacuugu......................................................................  1      1  seq
....................acaauuauucugacAugu......................................................................  1      1  seq
....................acaauuauucugacuuCu......................................................................  3      1  seq
....................acaauuauucugGcuugu......................................................................  2      1  seq
....................acaauuauuAugacuugu......................................................................  1      1  seq
....................acaauuauucugacuCgu......................................................................  1      1  seq
....................acaauuauucugacuugC......................................................................  10047  1  seq
....................acaauuauucugacuugu......................................................................  1488   0  seq
....................acaauuGuucugacuugu......................................................................  3      1  seq
....................acUauuauucugacuugu......................................................................  1      1  seq
....................acaauuauucugCcuugu......................................................................  1      1  seq
....................acaauuaCucugacuugu......................................................................  1      1  seq
....................acaauuauucugacuuAu......................................................................  2      1  seq
....................acaauuUuucugacuugu......................................................................  1      1  seq
....................acaaAuauucugacuugu......................................................................  1      1  seq
....................acaauuauucugacuugAg.....................................................................  8      1  seq
....................acaauuauucugacuugCg.....................................................................  36     1  seq
....................acaauuauucugacuuguA.....................................................................  1      1  seq
....................acaauuauucugacuuguU.....................................................................  2      1  seq
....................acaauuauucugacuugugU....................................................................  1      1  seq
.....................caauuauucugacuugu......................................................................  299    0  seq
.....................caauuauuUugacuugu......................................................................  1      1  seq
.....................caauuauucugUcuugu......................................................................  1      1  seq
.....................caauuauucugacuAgu......................................................................  1      1  seq
.....................caauuauucugacuuAu......................................................................  1      1  seq
.....................caauGauucugacuugu......................................................................  1      1  seq
.....................caauuaCucugacuugu......................................................................  3      1  seq
.....................caaAuauucugacuugu......................................................................  1      1  seq
.....................caauuauucugacuuCu......................................................................  1      1  seq
.....................caauuauCcugacuugu......................................................................  2      1  seq
.....................caCuuauucugacuugu......................................................................  1      1  seq
.....................caauuGuucugacuugu......................................................................  1      1  seq
.....................caauuauucugacuugC......................................................................  1701   1  seq
.....................caauuauucugacuugG......................................................................  15     1  seq
.....................caauuauucugacuuguA.....................................................................  1      1  seq
.....................caauuauucugacuugAg.....................................................................  1      1  seq
.....................caauuauucugacuugCg.....................................................................  4      1  seq
......................aauuauucugacuuguA.....................................................................  1      1  seq
.......................auuauucugacuuguAa....................................................................  1      1  seq
.......................auuauucugacuugAga....................................................................  1      1  seq
.......................................................uuagacuauGagucccuagacaau.............................  1      1  seq
..............................................................................ucacaaaguccgagaUcacc..........  1      1  seq
```

Provisional ID : MA_10431220_1032
Score total : 1.7
Score for star read(s) : -1.3
Score for read counts : 0
Score for mfe : 1.4
Score for randfold : 1.6
Score for cons. seed :
Total read count : 1805
Mature read count : 1805
Loop read count : 0
Star read count : 0

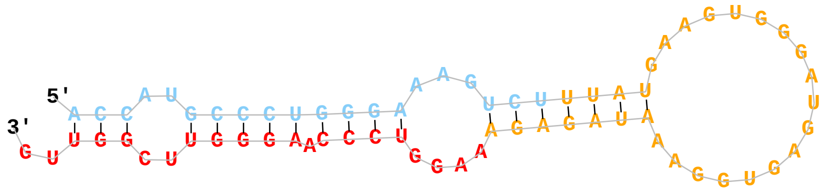

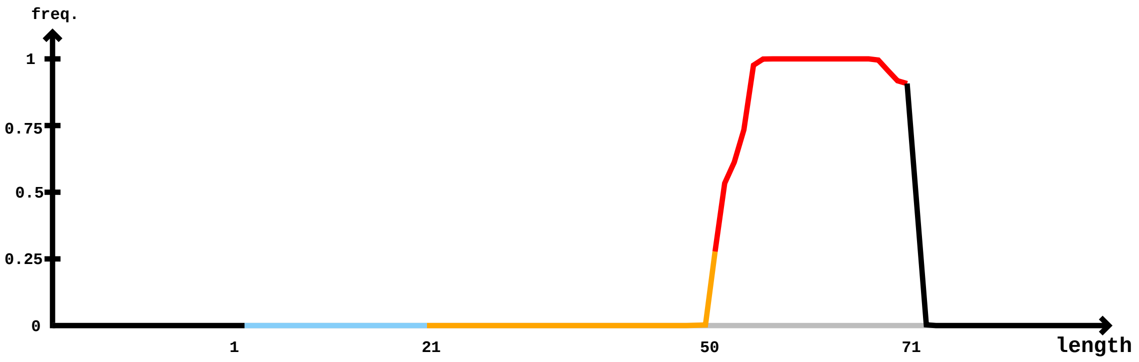

Star Mature

```
5'- acucguaucauuguccuuaccaugcccugggaaagucuuuaugaagugggaugaguggaaauagagaaaggucccaaggguucgguuggucacugauguaagcaaggg -3'   exp
.((..(((((.((.((..(((..((((((((...(((((((.................)))))))....)))).)))))..))).)).)).)))))..))......   reads   mm   sample
.................................................................gGaaggucccaaggguucgguug....................   2   1   seq
..................................................................Gaaggucccaaggguucgguugg...................   1   1   seq
..................................................................aaaggucccaaggguucgguugUu..................   1   1   seq
...................................................................aaggucccaaggguucg........................   8   0   seq
...................................................................aaggucccaaggguucgg.......................   19   0   seq
...................................................................Gaggucccaaggguucgg.......................   1   1   seq
...................................................................aaggucccaaggguucggu......................   15   0   seq
...................................................................aaggucccaaggguucgguG.....................   1   1   seq
...................................................................aaggucccaaggguucgguu.....................   4   0   seq
...................................................................aaggucccaaggguucgguug....................   391   0   seq
...................................................................aGggucccaaggguucgguug....................   4   1   seq
...................................................................aaggucccaGggguucgguug....................   3   1   seq
...................................................................Caggucccaaggguucgguug....................   2   1   seq
...................................................................aagAucccaaggguucgguug....................   1   1   seq
...................................................................aaggucccaaggguucgguGg....................   1   1   seq
...................................................................Uaggucccaaggguucgguug....................   1   1   seq
...................................................................aaggucccaUggguucgguug....................   1   1   seq
...................................................................aaggucccGaggguucgguug....................   1   1   seq
...................................................................aaggucccaaggguCcgguug....................   1   1   seq
...................................................................aaggucccaaggguucgguCg....................   8   1   seq
...................................................................aaggucccaaggguucgguuA....................   17   1   seq
...................................................................aaggucccaaggguucgguuU....................   2   1   seq
...................................................................aaggucccaaggguucAguug....................   1   1   seq
...................................................................aaggCcccaaggguucgguug....................   1   1   seq
...................................................................aaggucccaagggGucgguug....................   1   1   seq
...................................................................aaggucccaaggguucgCuug....................   1   1   seq
...................................................................aaggucccaaggguucgguugC...................   11   1   seq
...................................................................aaggucccaaggguucgguugg...................   1   0   seq
....................................................................aggucccaGggguucgg.......................   1   1   seq
....................................................................aggucccaaggguucgU.......................   1   1   seq
....................................................................aggAcccaaggguucgg.......................   1   1   seq
....................................................................aggucccaaggguucgg.......................   47   0   seq
....................................................................Uggucccaaggguucgg.......................   1   1   seq
....................................................................aggucccaaggguucggu......................   36   0   seq
```

Star Mature

```
acucguaucauuguccuuaccaugcccugggaaagucuuuaugaaguggaugaguggaaauagagaaaggucccaaggguucgguuggucacugauguaagcaaggg
...................................................................aggucccaaggguAcggu......................   1     1    seq
...................................................................aggucccaaggguucggG......................   2     1    seq
...................................................................Nggucccaaggguucggu......................   1     1    seq
...................................................................aggucAcaaggguucgguu.....................   1     1    seq
...................................................................aggucccaaggguucgguu.....................   10    0    seq
...................................................................aggucccaaggguucAguug....................   1     1    seq
...................................................................aggucccaaggguucgguuA....................   18    1    seq
...................................................................agguccUaaggguucgguug....................   1     1    seq
...................................................................aggucccaaggguucgguuU....................   2     1    seq
...................................................................aggucccaagggAucgguug....................   1     1    seq
...................................................................aggucccaaggguucgguCg....................   2     1    seq
...................................................................agAucccaaggguucgguug....................   2     1    seq
...................................................................aggCcccaaggguucgguug....................   1     1    seq
...................................................................aggucccGaggguucgguug....................   1     1    seq
...................................................................aggucccaaggguucgguug....................   309   0    seq
...................................................................aggucccaagAguucgguug....................   1     1    seq
...................................................................aggucccaaggguucggGug....................   1     1    seq
...................................................................aggucccaGggguucgguug....................   1     1    seq
...................................................................aggucccaaggguucgguGg....................   2     1    seq
...................................................................aggucccaagggCucgguug....................   1     1    seq
...................................................................aggucccaaggguucgguuC....................   2     1    seq
...................................................................aggucccaaggguucgguugg...................   3     0    seq
...................................................................aggucccaaggguucgguugA...................   1     1    seq
...................................................................aggucccaaggguucgguugC...................   10    1    seq
....................................................................ggucccaaggguucggu......................   14    0    seq
....................................................................ggucccaaggguucgguuA....................   4     1    seq
....................................................................ggucccaaggguucgguuC....................   1     1    seq
....................................................................ggucccaagggCucgguug....................   1     1    seq
....................................................................ggucccaaggguucgguug....................   105   0    seq
....................................................................ggucccaaggguucgguGg....................   1     1    seq
....................................................................ggucccGaggguucgguug....................   1     1    seq
....................................................................ggCcccaaggguucgguug....................   1     1    seq
....................................................................ggucccaaggguucgguugC...................   12    1    seq
....................................................................ggucccaaggguucgguugg...................   3     0    seq
.....................................................................gucccaaggguucgguA.....................   1     1    seq
.....................................................................gucccaaggguucgguu.....................   1     0    seq
.....................................................................Nucccaaggguucgguu.....................   1     1    seq
.....................................................................gucccaGggguucgguug....................   1     1    seq
.....................................................................gucccaaggguucgguug....................   176   0    seq
.....................................................................gucccaaggguucgguCg....................   1     1    seq
.....................................................................gucUcaaggguucgguug....................   1     1    seq
.....................................................................gucccaaggguucgguuC....................   2     1    seq
.....................................................................gucccaagggAucgguug....................   1     1    seq
.....................................................................gucccGaggguucgguug....................   1     1    seq
.....................................................................gucccaaggguucgguuA....................   13    1    seq
.....................................................................gucccaaggguucgguGg....................   1     1    seq
.....................................................................gucccaaggguucgguugC...................   12    1    seq
.....................................................................gucccaaggguucgguugg...................   4     0    seq
.....................................................................gucccaaggguucgguuAg...................   1     1    seq
.....................................................................gucccaaggguucgguugA...................   1     1    seq
.....................................................................gucccaaggguucgguuggC..................   1     1    seq
......................................................................ucccaaggCuucgguug....................   1     1    seq
......................................................................ucccaaggguAcgguug....................   2     1    seq
......................................................................ucccaaggguucgguGg....................   3     1    seq
......................................................................ucccaaAgguucgguug....................   1     1    seq
......................................................................ucccaaggguucggAug....................   1     1    seq
......................................................................ucccaaggguucgguuA....................   15    1    seq
......................................................................ucccaaggguucgguug....................   362   0    seq
......................................................................uccUaaggguucgguug....................   1     1    seq
......................................................................ucccaaggguucgguuC....................   1     1    seq
......................................................................ucccaagggGucgguug....................   1     1    seq
......................................................................ucccaGggguucgguug....................   1     1    seq
......................................................................ucccaaggguucgguCg....................   3     1    seq
......................................................................ucccaaggguucgguuU....................   1     1    seq
......................................................................ucccaagAguucgguug....................   2     1    seq
......................................................................ucccaagggCucgguug....................   2     1    seq
......................................................................ucUcaaggguucgguug....................   1     1    seq
......................................................................uUccaaggguucgguug....................   1     1    seq
......................................................................ucccaaggguucgguugC...................   26    1    seq
......................................................................ucccaaggguucgguugg...................   7     0    seq
```

```
                   Star                                              Mature
acucguaucauuguccuuaccaugcccugggaaagucuuuaugaaguggaugaguggaaauagagaaaggucccaaggguucgguuggucacugauguaagcaaggg
......................................................................ucccaaggguucgguugA...................    3           1           seq
......................................................................ucccaaggguucgguugCu..................   2           1           seq
.......................................................................cccaaggguucgguugC...................   38          1           seq
.......................................................................cccaaggguucgguugA...................   2           1           seq
.......................................................................cccaaggguucgguugg...................   2           0           seq
........................................................................ccaaggguucgguugCu..................   1           1           seq
```

Provisional ID	:	MA_10432105_13085
Score total	:	2.3
Score for star read(s)	:	-1.3
Score for read counts	:	0
Score for mfe	:	2
Score for randfold	:	1.6
Score for cons. seed	:	
Total read count	:	331
Mature read count	:	331
Loop read count	:	0
Star read count	:	0

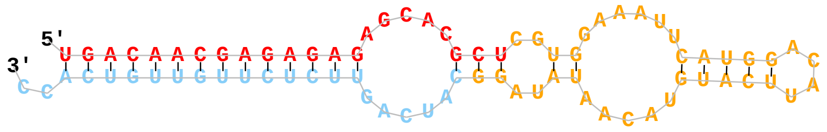

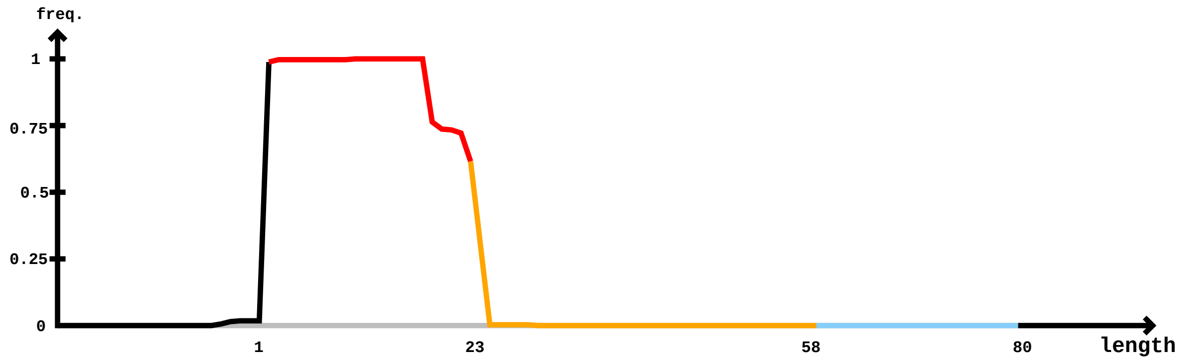

Mature Star

```
5'- uugguuggauaauauuugggugacaacgagagagagcacgcucguggaaauucauggacauucauguacaauauaggcaucaguucucuuguugucacccaaaauauauuca   -3'    exp
    .....((((((...(((((((((((((((((((.....(((..((......(((((....))))).....))..))).....)))))))))))))))))))...))))))   reads   mm   sample
    ...............uugggugacaacgagagagagc...........................................................................   2   0   seq
    ................ugggugacaacgagagagagU...........................................................................   1   1   seq
    ................ugggugacaacgagagagagc...........................................................................   2   0   seq
    .................gggugacaacgagagagagcacg........................................................................   1   0   seq
    ....................ugGcaacgagagagagc...........................................................................   1   1   seq
    ....................ugacaaUgagagagagc...........................................................................   1   1   seq
    ....................ugacaacgagagagagc...........................................................................   56   0   seq
    ....................ugacaacgagagagagA...........................................................................   1   1   seq
    ....................ugacaacgagagagagU...........................................................................   6   1   seq
    ....................ugacaaAgagagagagc...........................................................................   10   1   seq
    ....................ugacaacgagagagagca..........................................................................   7   0   seq
    ....................ugacaaAgagagagagca..........................................................................   1   1   seq
    ....................ugacaaAgagagagagcacg........................................................................   1   1   seq
    ....................ugacaacgagagagagcacg........................................................................   1   0   seq
    ....................ugacaacgagagagagcacgc.......................................................................   3   0   seq
    ....................ugacaacgagagagagcacgG.......................................................................   1   1   seq
    ....................ugacaaAgagagagagcacgc.......................................................................   32   1   seq
    ....................ugacaUcgagagagagcacgcu......................................................................   1   1   seq
    ....................ugacaacgGgagagagcacgcu......................................................................   1   1   seq
    ....................ugacaacgagagagagcacgUu......................................................................   1   1   seq
    ....................ugGcaacgagagagagcacgcu......................................................................   3   1   seq
    ....................Ngacaacgagagagagcacgcu......................................................................   1   1   seq
    ....................ugacaacgagagGgagcacgcu......................................................................   2   1   seq
    ....................ugacaacgagagagagcacgcu......................................................................   180   0   seq
    ....................ugacaacgagagagagcacgcA......................................................................   8   1   seq
    ....................ugacaacgagagagGgcacgcu......................................................................   2   1   seq
    ....................ugacaacgagagagagcacgcG......................................................................   5   1   seq
    ....................ugacUacgagagagagcacgcu......................................................................   1   1   seq
    ....................ugacaacgagagagagcacgcuG.....................................................................   1   1   seq
    ....................ugacaacgagagagagcacgcuA.....................................................................   1   1   seq
    .....................gacaacgagagagagca..........................................................................   1   0   seq
    .....................gacaacgagagagagcac.........................................................................   1   0   seq
    .....................gacaacgagagagagcacg........................................................................   1   0   seq
    .............................gagagagcacgcuUgugga................................................................   1   1   seq
```

Mature **Star**

uugguuggauaauauuugggugacaacgagagagagcacgcucguggaaauucauggacauucauguacaauauaggcaucaguucucuuguugucacccaaaauauauuca

Provisional ID	:	MA_10432205_7464
Score total	:	0.6
Score for star read(s)	:	-1.3
Score for read counts	:	0
Score for mfe	:	0.3
Score for randfold	:	1.6
Score for cons. seed	:	
Total read count	:	2371
Mature read count	:	2369
Loop read count	:	0
Star read count	:	2

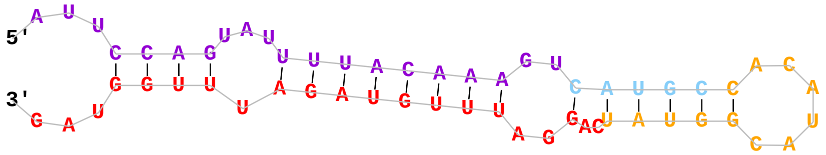

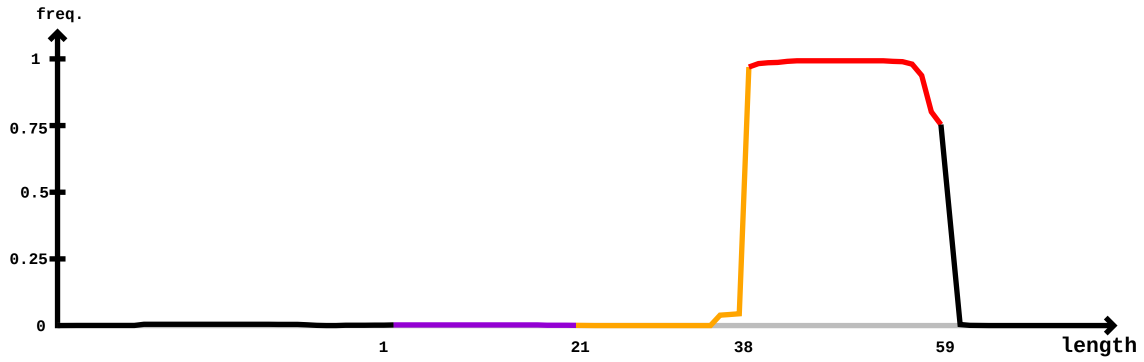

Star Mature

```
5'- cucgacuucauagugaagucaagggccaccauuauuccaguauuuuacaaagucaugccacauacgguaucaggauuuguagauuugguagauggguuuauaaagguu -3'   obs
    cucgacuucauagugaagucaagggccaccauuauuccaguauuuuacaaagucaugccacauacgguaucaggauuuguagauuugguagauggguuuauaaagguu        exp
    ((.((((((.....)))))).)).....(((((...((((...((((((((..((((((......)))))..)..))))))))).)))).)).)))))............   reads   mm   sample
    cucgacuucauGgugaaguca.......................................................................................   1   1   seq
    .......ucauagugaagucaagg....................................................................................   3   0   seq
    .......ucauGgugaagucaagg....................................................................................   1   1   seq
    .......ucauagugaagucaaggU...................................................................................   1   1   seq
    .......ucauagugaagucaaggg...................................................................................   2   0   seq
    .......ucauagugaagucaUggg...................................................................................   1   1   seq
    .......Ncauagugaagucaaggg...................................................................................   1   1   seq
    .......ucauagugaagucaagggc..................................................................................   1   0   seq
    .......ucauagugaagucaagggG..................................................................................   1   1   seq
    ............................ccauuauuccaguGuuuuaca...........................................................   1   1   seq
    ............................ccauCauuccaguauuuuaca...........................................................   2   1   seq
    ...............................uuauuccaguauuuuacaaaC........................................................   1   1   seq
    .................................auuccaguCuuuuacaaagu.......................................................   2   1   seq
    ...................................................................uaCcaggauuuguagauu.......................   3   1   seq
    ...................................................................uaCcaggauuuguagauuu......................   2   1   seq
    ...................................................................uaCcaggauuuguagauuug.....................   6   1   seq
    ...................................................................uaCcaggauuuguagauuugg....................   86   1   seq
    ....................................................................aCcaggauuuguagauu.......................   2   1   seq
    ....................................................................aCcaggauuuguagauuug.....................   2   1   seq
    ....................................................................aCcaggauuuguagauuugg....................   1   1   seq
    ....................................................................aCcaggauuuguagauuuggu...................   1   1   seq
    .....................................................................Ccaggauuuguagauuu......................   1   1   seq
    .....................................................................Ccaggauuuguagauuug.....................   1   1   seq
    .....................................................................Ccaggauuuguagauuugg....................   1   1   seq
    .....................................................................ucaggauuuguagauuuggGa..................   2   1   seq
    .....................................................................Ccaggauuuguagauuuggua..................   1   1   seq
    .....................................................................ucaggauuugAagauuuggua..................   1   1   seq
    ......................................................................caggauuuguagauuug.....................   13   0   seq
    ......................................................................caggauuuguagauuuC.....................   1   1   seq
    ......................................................................caggauuuguagauuugg....................   13   0   seq
    ......................................................................caggauuuguagauuugU....................   1   1   seq
    ......................................................................caggauuuguagauuugA....................   3   1   seq
    ......................................................................caggauuCguagauuuggu...................   1   1   seq
```

Star **Mature**

```
cucgacuucauagugaagucaagggccaccauuauuccaguauuuuacaaagucaugccacauacgguaucaggauuuguagauuugguagauggguuuauaaagguu
......................................................................caggauuuguagauuuggG...................    315     1     seq
......................................................................caggauuuguagauuuggu...................    4       0     seq
......................................................................caggauuuguagauuuggA...................    13      1     seq
......................................................................caggauuuguagauuuggC...................    2       1     seq
......................................................................caggauuuguagauuuggGa..................    104     1     seq
......................................................................caggauuuguagauuuggGag.................    1813    1     seq
......................................................................caggauuugAagauuugguag.................    1       1     seq
......................................................................caggauuuguagauuuggAag.................    1       1     seq
......................................................................caggauuuguagauuuggGaga................    13      1     seq
.......................................................................aggauuuguagauuugg....................    1       0     seq
.......................................................................aggauuuguagauuuUgua..................    1       1     seq
.......................................................................aggauuuguagauuuggGa..................    10      1     seq
.......................................................................aggauuuguagauuuggGag.................    4       1     seq
.......................................................................aggauuuguagauuuUguag.................    1       1     seq
.......................................................................aggauuuguagauuuUguaga................    2       1     seq
.......................................................................aggauuuguagauuuggGaga................    13      1     seq
........................................................................ggauuuguagauuuggA...................    2       1     seq
........................................................................ggauuuguagauuuUguag.................    1       1     seq
........................................................................ggauuuguagauuuUguagau...............    3       1     seq
........................................................................ggauuuguagauuuggGagau...............    2       1     seq
.........................................................................gauuuguagauuuggGag.................    3       1     seq
..........................................................................auuuguagauuuggGag.................    9       1     seq
..........................................................................auuuguagauuuggGagau...............    1       1     seq
...........................................................................uuuguagauuuggGaga................    1       1     seq
...........................................................................uuuguagauuuggGagau...............    2       1     seq
...........................................................................uuuguagauuuggGagaug..............    1       1     seq
...........................................................................uuuguagauuuggGagaugg.............    1       1     seq
```

Provisional ID	:	MA_10432709_25847
Score total	:	1.9
Score for star read(s)	:	-1.3
Score for read counts	:	0
Score for mfe	:	1.6
Score for randfold	:	1.6
Score for cons. seed	:	
Total read count	:	229
Mature read count	:	229
Loop read count	:	0
Star read count	:	0

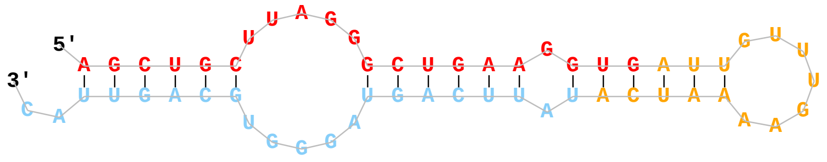

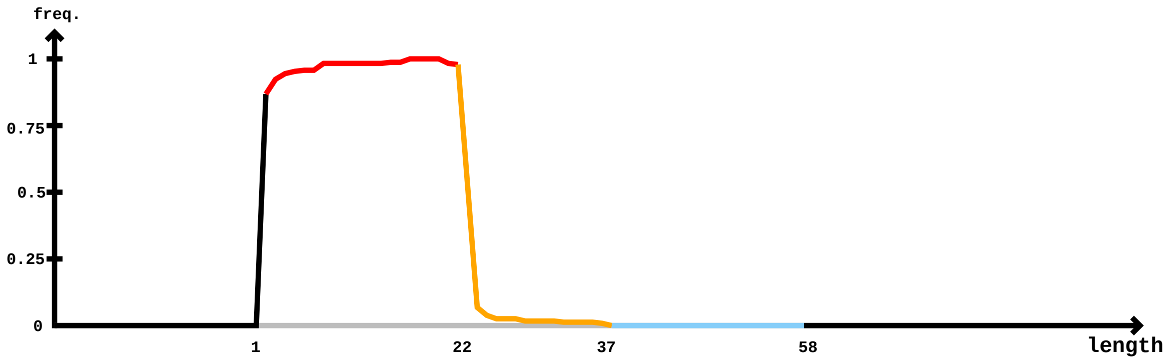

Mature Star

```
5'- ccgugauaugagcaaguuggagcugcuuagggcugaaggugauuguuugaaaaucauauucaguagggugcaguuaccacaucucauauggcucagaugggauaguugucc   -3'   exp
    ..((.(((((((...((.((((((((.....((((((.((((((.......)))))).)))))).....)))))).))))..))))))).)).......((((....))))   reads   mm   sample
    ....................agcugcuuagAgcugaagg..........................................................................   1       1    seq
    ....................agcugcuuagggcugaagg..........................................................................   1       0    seq
    ....................agcugcuuagggcugaaggGg........................................................................   1       1    seq
    ....................agcugcuuagAgcugaaggug........................................................................   150     1    seq
    ....................agcugcuuagggcugaaggug........................................................................   45      0    seq
    ....................agcugcuGagggcugaaggug........................................................................   1       1    seq
    ....................agUugcuuagggcugaaggug........................................................................   4       1    seq
    ....................agcugcuuaggUcugaaggug........................................................................   1       1    seq
    .....................gcugcuuagggcugUagg..........................................................................   1       1    seq
    .....................gcugcuuagAgcugaagg..........................................................................   1       1    seq
    .....................gcugcuuagAgcugaaggu.........................................................................   1       1    seq
    .....................gcugcuuagAgcugaaggug........................................................................   3       1    seq
    .....................gcugcuuagggcugaagguga.......................................................................   1       0    seq
    .....................gcugcuuagAgcugaagguga.......................................................................   5       1    seq
    .....................gcugcuuagAgcugaaggugauu.....................................................................   1       1    seq
    ......................cugcuuagAgcugaaggug........................................................................   1       1    seq
    ......................Uugcuuagggcugaaggugau......................................................................   1       1    seq
    ......................cugcuuagggcugaaggugau......................................................................   1       0    seq
    ......................cugcuuagAgcugaaggugau......................................................................   2       1    seq
    .......................ugcuuagggcugaaggug........................................................................   1       0    seq
    .......................ugcuuagggcugaaggugauu.....................................................................   1       0    seq
    ........................gcuuagggcugaaggug........................................................................   1       0    seq
    ..........................uuagggUugaaggugau......................................................................   2       1    seq
    ..........................uuagAgcugaaggugau......................................................................   1       1    seq
    ..........................uuagAgcugaaggugauu.....................................................................   1       1    seq
    ..........................uuagCgcugaaggugauuguu..................................................................   1       1    seq
    ..........................uGagggcugaaggugauuguu..................................................................   1       1    seq
    .................................ugaaggugauugGuugaa..............................................................   1       1    seq
    ...................................aaggugauuguuugaaaaGc..........................................................   1       1    seq
    ...................................aaggugauuguuugaaaaGca.........................................................   2       1    seq
```

Provisional ID	:	MA_10432994_8546
Score total	:	2.9
Score for star read(s)	:	-1.3
Score for read counts	:	0
Score for mfe	:	2.6
Score for randfold	:	1.6
Score for cons. seed	:	
Total read count	:	35056
Mature read count	:	34781
Loop read count	:	0
Star read count	:	275

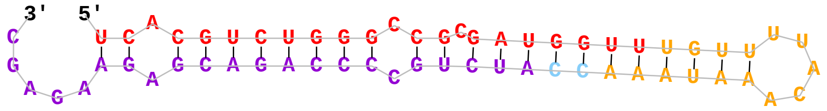

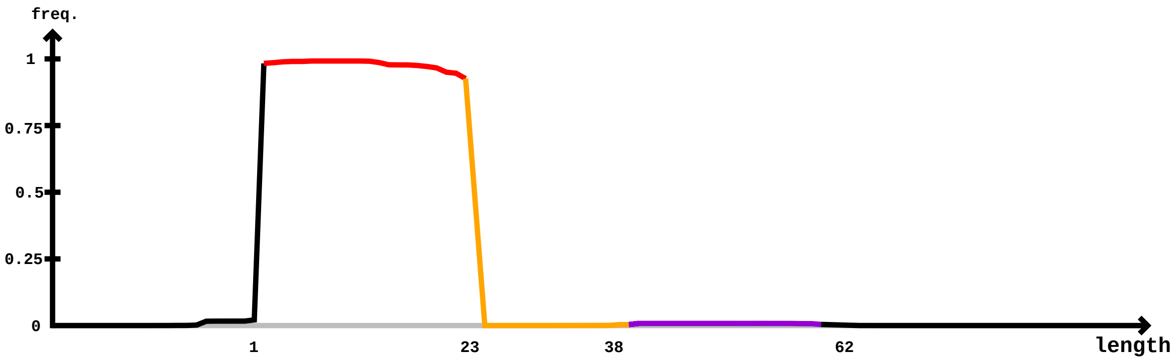

Mature Star

```
5'- gaagaaagcgaagguguucuucacgucugggccgcgaugguuuguuuuacaaauaaaccaucugccccagacgagaagagcaccuucgccuucguuuacauagauuguguau   -3'   obs
    gaagaaagcgaagguguucuucacgucugggccgcgaugguuuguuuuacaaauaaaccaucugccccagacgagaagagcaccuucgccuucguuuacauagauuguguau         exp
    ...(((.((((((((((((((.((((((((.((.(((((((((((.....))))))))))))).)))))))).)))))))))))))).)))...(((((.....))))).  reads  mm  sample
    ........cgaagguguucuuGacgucugggcc...............................................................................  1      1   seq
    .........gaagguguucuuGacgucugggcc...............................................................................  1      1   seq
    ...........agguguucuucacgucugggc................................................................................  1      0   seq
    ...........agguguucuuGacgucugggcc...............................................................................  13     1   seq
    ............gguguucuuGacgucugggcc...............................................................................  3      1   seq
    .............guguucuuGacgucugggc................................................................................  2      1   seq
    .............guguucuuGacgucugggcc...............................................................................  47     1   seq
    ..............uguucuuGacgucuggg.................................................................................  28     1   seq
    ..............uguucuuGacgucugggc................................................................................  163    1   seq
    ..............uguucuuGacgucugggcc...............................................................................  228    1   seq
    ..............uguucuuGacgucugggccg..............................................................................  17     1   seq
    ..............uguucuuGacgucugggccgc.............................................................................  2      1   seq
    ..............uguucuuGacgucugggccgcg............................................................................  76     1   seq
    ...............guucuuGacgucugggc................................................................................  10     1   seq
    ...............guucuuGacgucugggcc...............................................................................  4      1   seq
    ................uucuuGacgucugggcc...............................................................................  1      1   seq
    .................ucuucacgucugggccAcgaugg........................................................................  1      1   seq
    ...................uucacgucugggccgcg............................................................................  1      0   seq
    ...................uucacgucugggccAcg............................................................................  3      1   seq
    ...................uucacgucugggccAcga...........................................................................  1      1   seq
    ...................uucacgucugggccAcgaug.........................................................................  6      1   seq
    ...................uucacgucugggccAcgaugg........................................................................  6      1   seq
    ...................uucacgucugggccgcgauggu.......................................................................  1      0   seq
    ...................uucacgucugggccAcgauggu.......................................................................  138    1   seq
    ...................uucacgucugggccAcgaugguu......................................................................  1      1   seq
    ....................uUacgucugggccgcga...........................................................................  2      1   seq
    ....................ucacgucugggccAcga...........................................................................  129    1   seq
    ....................ucacgucugggccAcgau..........................................................................  188    1   seq
    ....................uAacgucugggccgcgau..........................................................................  2      1   seq
    ....................uAacgucugggccgcgaug.........................................................................  3      1   seq
    ....................ucacgucugggccgcgaug.........................................................................  4      0   seq
    ....................ucacgucugggccAcgaug.........................................................................  536    1   seq
    ....................uUacgucugggccgcgaug.........................................................................  6      1   seq
```

```
                    Mature                                 Star
gaagaaagcgaagguguucuucacgucugggccgcgaugguuuguuuuacaaauaaaccaucugccccagacgagaagagcaccuucgccuucguuuacauagauuguguau
....................ucacgucugggccAcgaugg........................................................................ 121 1 seq
....................uAacgucugggccgcgauggu....................................................................... 18 1 seq
....................uUacgucugggccgcgauggu....................................................................... 22 1 seq
....................ucacAucugggccgcgauggu....................................................................... 1 1 seq
....................ucacgucugggccAcgauggu....................................................................... 514 1 seq
....................ucacgucugggccgcgaugguu...................................................................... 117 0 seq
....................ucacgucugggccgcgaugguG...................................................................... 1 1 seq
....................ucacgucugggccgcgauggCu...................................................................... 2 1 seq
....................ucacgucuggAccgcgaugguu...................................................................... 1 1 seq
....................ucacgucugggccgcgaugguC...................................................................... 23 1 seq
....................ucacgucugggccUcgaugguu...................................................................... 22 1 seq
....................ucacgucugggccCcgaugguu...................................................................... 6 1 seq
....................ucacAucugggccgcgaugguu...................................................................... 6 1 seq
....................ucGcgucugggccgcgaugguu...................................................................... 1 1 seq
....................ucacgucugggccgUgaugguu...................................................................... 1 1 seq
....................ucacgucugggccAcgaugguu...................................................................... 31318 1 seq
....................uUacgucugggccgcgaugguu...................................................................... 764 1 seq
....................uAacgucugggccgcgaugguu...................................................................... 252 1 seq
....................uUacgucugggccgcgaugguuu..................................................................... 2 1 seq
....................ucacgucugggccAcgaugguuu..................................................................... 240 1 seq
....................ucacgucugggccgcgaugguuC..................................................................... 5 1 seq
....................uAacgucugggccgcgaugguuu..................................................................... 3 1 seq
.....................cacgucugggccAcgau.......................................................................... 1 1 seq
.....................cacgucugggccAcgaug......................................................................... 5 1 seq
.....................cacgucugggccAcgaugg........................................................................ 6 1 seq
.....................cacgucugggccAcgauggu....................................................................... 18 1 seq
.....................cacgucugggccAcgaugguu...................................................................... 22 1 seq
.....................Uacgucugggccgcgaugguu...................................................................... 6 1 seq
.....................cacgucugggccAcgaugguuu..................................................................... 33 1 seq
......................acgucugggccAcgaug......................................................................... 12 1 seq
......................acgucugggccAcgaugg........................................................................ 3 1 seq
......................acgucugggccAcgauggu....................................................................... 6 1 seq
......................acgucugggccAcgaugguu...................................................................... 76 1 seq
......................acgucugggccAcgaugguuu..................................................................... 22 1 seq
.......................cgucugggccgcgaugguC...................................................................... 1 1 seq
.......................cgucugggccAcgaugguu...................................................................... 44 1 seq
.......................cgucugggccAcgaugguuu..................................................................... 2 1 seq
.........................ucugggccCcgaugguu...................................................................... 50 1 seq
.........................ucuggAccgcgaugguu...................................................................... 1 1 seq
.........................ucCgggccgcgaugguu...................................................................... 3 1 seq
.........................ucugggccgcgaugguuA..................................................................... 1 1 seq
.........................ucugggccCcgaugguuu..................................................................... 1 1 seq
.........................ucugggccCcgaugguuug.................................................................... 1 1 seq
...............................................uacCaauaaaccaucug................................................ 1 1 seq
...................................................aauaaaccaucugcccU............................................ 1 1 seq
...................................................aauaaaccaucugccccag.......................................... 1 0 seq
...................................................aauaaaccaucugccccagacg....................................... 1 0 seq
....................................................auaaaccaucugccccagacgaga.................................... 1 0 seq
....................................................auaaaccaucugccccaUacgaga.................................... 2 1 seq
.....................................................uaaaccaucugccccaUacgaga.................................... 1 1 seq
......................................................aaaccaucugccccagacgagaag.................................. 1 0 seq
.......................................................aaccaucugAcccagacga...................................... 1 1 seq
.......................................................aaccaucugccccagacgaga.................................... 1 0 seq
.......................................................aaccaucugUcccagacgaga.................................... 1 1 seq
.......................................................aaccaucugccccaUacgaga.................................... 1 1 seq
.......................................................aaccaucugAcccagacgagaag.................................. 1 1 seq
........................................................accaucugAcccagacg....................................... 1 1 seq
........................................................accaucugccccaUacgag..................................... 1 1 seq
........................................................accaucugccccaUacgaga.................................... 1 1 seq
........................................................accaucugccccaUacgagaa................................... 3 1 seq
.........................................................ccaucugccccagacga...................................... 1 0 seq
.........................................................ccaucugccccagacgag..................................... 1 0 seq
.........................................................ccaucugccccagacgaA..................................... 1 1 seq
.........................................................ccaucugccccagacgaga.................................... 4 0 seq
.........................................................ccaucugccccaUacgaga.................................... 3 1 seq
.........................................................ccaucugAcccagacgaga.................................... 4 1 seq
.........................................................ccaucugccccagacgagaa................................... 1 0 seq
.........................................................ccaucugccccagacgagaaU.................................. 1 1 seq
.........................................................ccaucugccccagacgagaaA.................................. 1 1 seq
.........................................................ccaucugAcccagacgagaag.................................. 56 1 seq
```

```
                    Mature                                  Star
gaagaaagcgaagguguucuucacgucugggccgcgaugguuuguuuuacaaauaaaccaucugccccagacgagaagagcaccuucgccuucguuuacauagauuguguau
.........................................................ccaucugccccagacgagaaC..................................    1     1     seq
.........................................................ccaucugccccagacgagaag..................................    27    0     seq
.........................................................ccaucugUcccagacgagaag..................................    1     1     seq
.........................................................ccaucugccccagacgagaaga.................................    1     0     seq
.........................................................ccaucugccccagacgagaagU.................................    1     1     seq
.........................................................ccaucugccccagacgagaagG.................................    1     1     seq
..........................................................caucugAcccagacgagaag..................................    1     1     seq
..........................................................caucugccccagacgagaag..................................    3     0     seq
..........................................................caucugccccagaUgagaagagc...............................    1     1     seq
...........................................................aucugccccagacgaga....................................    1     0     seq
...........................................................aucugccccagacgagaag..................................    5     0     seq
...........................................................aucugAcccagacgagaag..................................    2     1     seq
...........................................................aucugccccagacgagaagU.................................    2     1     seq
...........................................................aucugccccagacgagaaga.................................    1     0     seq
...........................................................aucugccccagacgagaagag................................    1     0     seq
...........................................................aucugccccagacgagaagaC................................    1     1     seq
...........................................................aucugccccagacgagaagagU...............................    20    1     seq
...........................................................aucugccccagacgagaGgagc...............................    1     1     seq
...........................................................aucugccccagacgagaagagc...............................    110   0     seq
...........................................................aucugccccagacgagaagagA...............................    1     1     seq
...........................................................aucugccccagGcgagaagagc...............................    1     1     seq
...........................................................aucugccccagacgagaagaCc...............................    1     1     seq
...........................................................aucugccccGgacgagaagagc...............................    1     1     seq
...........................................................aucugccUcagacgagaagagc...............................    1     1     seq
...........................................................Gucugccccagacgagaagagc...............................    1     1     seq
............................................................ucugccccagacgagaaU..................................    1     1     seq
............................................................ucugccccagacgagaagagU...............................    1     1     seq
............................................................ucugccccagacgagaagagc...............................    2     0     seq
............................................................ucugccccagacgagaagagcacc............................    2     0     seq
.......................................................................cgUgaagagcaccuucgc.......................    1     1     seq
```

Provisional ID	:	MA_10433397_7580
Score total	:	54.6
Score for star read(s)	:	3.9
Score for read counts	:	47
Score for mfe	:	2
Score for randfold	:	1.6
Score for cons. seed	:	
Total read count	:	104
Mature read count	:	97
Loop read count	:	0
Star read count	:	7

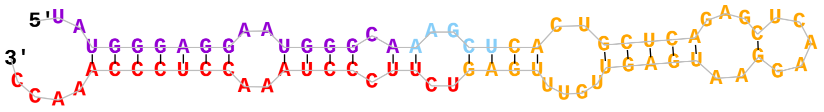

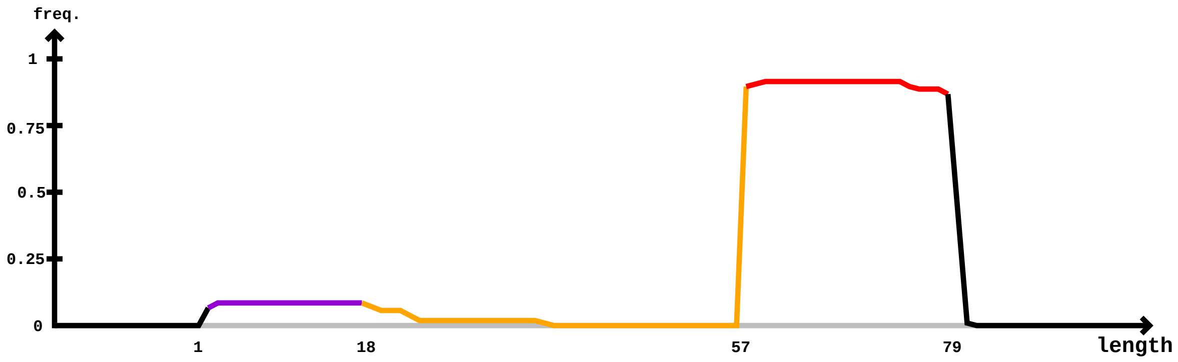


```
                                   Star                                                        Mature
5'- uggagggcaauggguaugggaggaaugggcaaagcucacugcucagagcucaaggaaugaguuguuugagucuucccuaaaccucccaaacccaucauuaccuuucacagcg  -3'   obs
    uggagggcaauggguaugggaggaaugggcaaagcucacugcucagagcucaaggaaugaguuguuugagucuucccuaaaccucccaaacccaucauuaccuuucacagcg        exp
    (((((((..((((((.(((((((..((((.((..((((..(((((...(.....)..)))))....))))..)).))))..))))))).)))))).....))))))).....   reads   mm   sample
    ..............uaugggaggaaugggca.................................................................................   2       0    seq
    ..............uaugggaggaaugggcU.................................................................................   1       1    seq
    ..............uaugggaggaaugggcaaagc.............................................................................   2       0    seq
    ..............uaugggaggaaugggcaaagcu............................................................................   1       0    seq
    ..............uaugggaggaaugggcaaagcA............................................................................   1       1    seq
    ...............augggaggaaugggcaaagcucacugcucagagc...............................................................   1       0    seq
    ...............augggaggaaugggcaaagcucacugcucagagcu..............................................................   1       0    seq
    ......................................................................ucuucccuaaaccucccU........................   1       1    seq
    ......................................................................ucuucccuaaaccuccc.........................   1       0    seq
    ......................................................................ucuucccuaaaccuccca........................   1       0    seq
    ......................................................................ucuucccuaaaccucccaaac.....................   1       0    seq
    ......................................................................ucuucccuaaaccucccaaaU.....................   1       1    seq
    ......................................................................Acuucccuaaaccucccaaacc....................   1       1    seq
    ......................................................................ucuCcccuaaaccucccaaacc....................   1       1    seq
    ......................................................................ucuucccuaaaccucccUaacc....................   1       1    seq
    ......................................................................ucuucccuaaaccucccaaacU....................   10      1    seq
    ......................................................................ucuucccuaaaccucccaaacc....................   73      0    seq
    ......................................................................ucuucccuaaaccCcccaaacc....................   1       1    seq
    ......................................................................ucuucccuaaaccucccaaacA....................   1       1    seq
    ......................................................................ucuucccuaaaccucccaaaAcc...................   1       1    seq
    ......................................................................ucuucccuaaaccucccaaaccca..................   1       0    seq
    .......................................................................cuucccuaaaccucccaaacc....................   1       0    seq
    ........................................................................uucccuaaaccucccaaacc....................   1       0    seq
```

Provisional ID	:	MA_10433644_5533
Score total	:	0.4
Score for star read(s)	:	-1.3
Score for read counts	:	-0.3
Score for mfe	:	0.5
Score for randfold	:	1.6
Score for cons. seed	:	
Total read count	:	11
Mature read count	:	11
Loop read count	:	0
Star read count	:	0

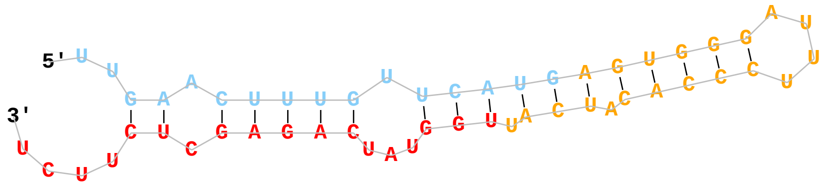

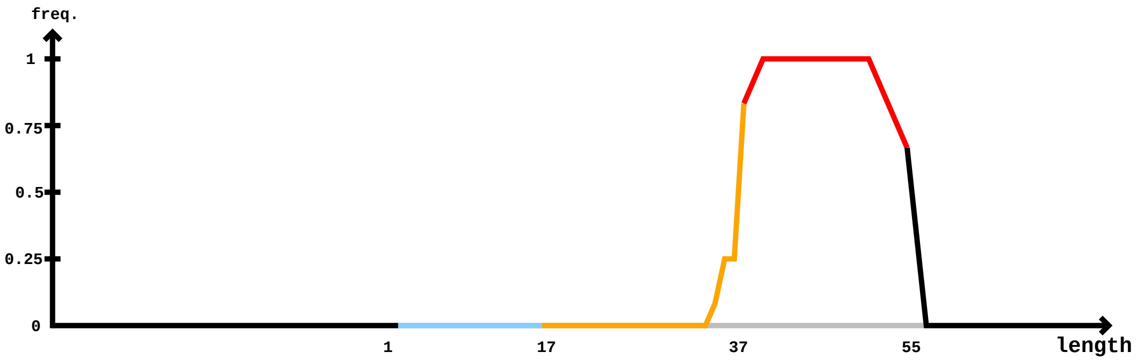

Star **Mature**

```
5'- aaaaagcaauuagagauuuuuucacacuuuggaguugaacuuuguucaugagugggauuucccacaucauugguaucagagcucuucuccacucucuuugguucaaau   -3'    exp
    ...((.(((..((((..............(((((..((.(((((.(((((((((((....))))).))).)))...))))).))..)))))))))..))).)).....    reads    mm    sample
    ...................................................................cauugguaucagagcuU........................    1        1     seq
    ....................................................................auugguaGcagagcucu.......................    1        1     seq
    ....................................................................Uuugguaucagagcucuu......................    1        1     seq
    ......................................................................ugguaCcagagcucuuc.....................    1        1     seq
    ......................................................................ugguaucagagcucuucu....................    4        0     seq
    ......................................................................ugguaucagagcucuGcu....................    1        1     seq
    ......................................................................uUguaucagagcucuucu....................    1        1     seq
    .......................................................................gguaucagagcucuGcu....................    1        1     seq
    ........................................................................guaucagagcucuGcuc...................    1        1     seq
```

Provisional ID	:	MA_10433715_2464
Score total	:	1.8
Score for star read(s)	:	-1.3
Score for read counts	:	0
Score for mfe	:	1.5
Score for randfold	:	1.6
Score for cons. seed	:	
Total read count	:	31
Mature read count	:	31
Loop read count	:	0
Star read count	:	0

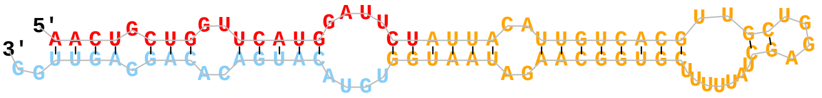

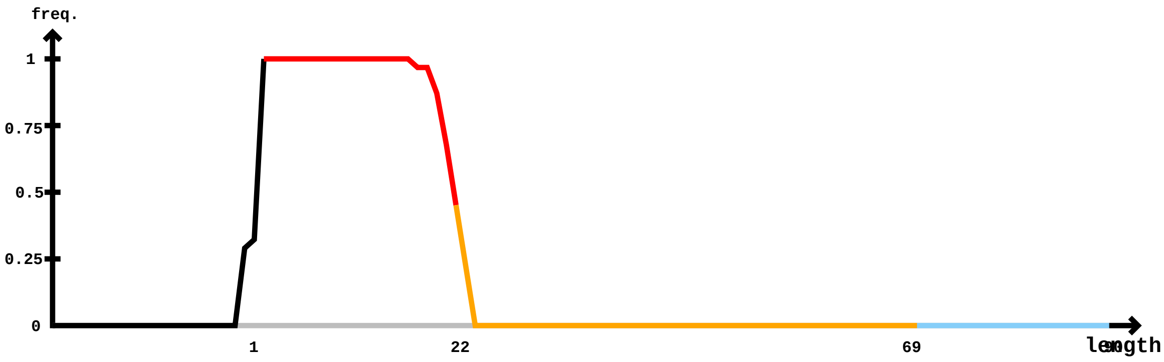

Mature **Star**

```
5'- uggcuguguagggaugauucaacugcugguucauggauucuauuacauugucacguugcuggagcuauuuuucguggcaagauaauggguacaugacacaggaguuggaa   -3'    exp
    .................(((((((.(((..(((((....((((((..((((((((..((....)).......))))))))..))))))....))))).))).))))))).    reads       mm         sample
    ..................ucaacugcugguucaugg...........................................................................    1           0          seq
    ..................ucaacugcugguucauggauC........................................................................    2           1          seq
    ..................ucaacugcugguucauggauu........................................................................    4           0          seq
    ..................ucaacugcugguucauggauuc.......................................................................    2           0          seq
    ...................caacugcugguucauggauuc.......................................................................    1           0          seq
    ....................aacugcugguucauggau.........................................................................    2           0          seq
    ....................aacugcugguucGuggau.........................................................................    1           1          seq
    ....................aacugcugguucauggauuc.......................................................................    4           0          seq
    ....................aacugcugguucauggauucu......................................................................    9           0          seq
    ....................aacugcugguucauggauucC......................................................................    3           1          seq
    ....................aacugcugguucauggauucA......................................................................    2           1          seq
```

Provisional ID	:	MA_10433985_8636
Score total	:	46.4
Score for star read(s)	:	3.9
Score for read counts	:	47.5
Score for mfe	:	-2.7
Score for randfold	:	-2.2
Score for cons. seed	:	
Total read count	:	105
Mature read count	:	72
Loop read count	:	1
Star read count	:	32

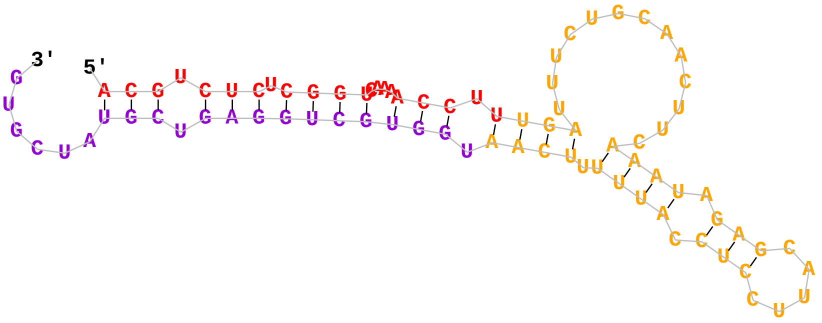

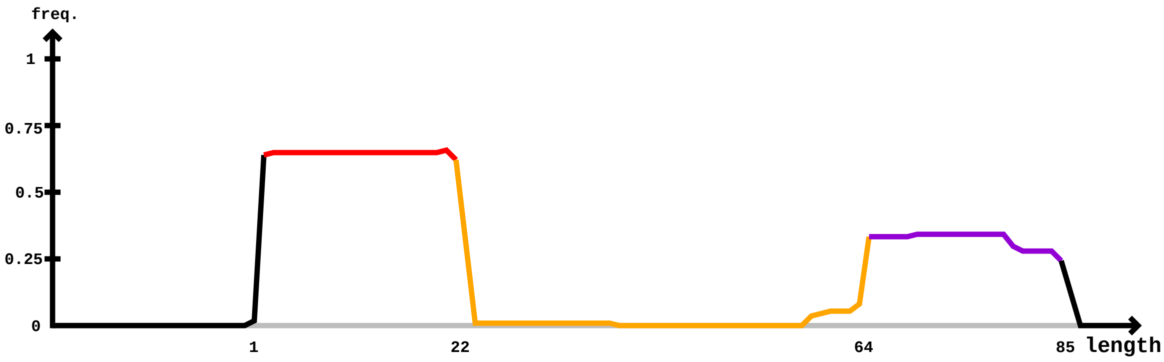

Mature Star

```
5'- gugguguucucaccuguuauacgucucucggucaaaaccuuugauuucugcaacuucaaauagagcauuccuccauuuuucaauggugcuggagucguaucgugguugauc   -3'    obs
    gugguguucucaccuguuauacgucucucggucaaaaccuuugauuucugcaacuucaaauagagcauuccuccauuuuucaauggugcuggagucguaucgugguugauc          exp
    .........(((.(..(.(((((.(((.((((....(((.((((.............((((.(((.....))).)))).)))).)))))))))).))))).)..).)))..  reads  mm  sample
    ...................uacgucucucggucaaaaccC.......................................................................  2  1  seq
    ....................acgucucucggucaaaaccA.......................................................................  1  1  seq
    ....................acgucucucggucaaaaccC.......................................................................  1  1  seq
    ....................acgucucucgUucaaaaccuu......................................................................  2  1  seq
    ....................acgucucucggucaaaaccuG......................................................................  3  1  seq
    ....................acgucucucggucaaaaccuC......................................................................  4  1  seq
    ....................acgucucucggucaaaUccuu......................................................................  1  1  seq
    ....................acgucucucggucaaaaccuu......................................................................  54  0  seq
    ....................acgCcucucggucaaaaccuu......................................................................  2  1  seq
    ....................acgucucucggucaaaaccuuC.....................................................................  1  1  seq
    .....................cgucucucggucaaaaccuuu.....................................................................  1  0  seq
    .......................................uuugaAuucugcaacuuc......................................................  1  1  seq
    .............................................................................uuucaauggugUuggagucgu.............  3  1  seq
    .............................................................................uuucaauggugcuggaUucgu.............  1  1  seq
    ..............................................................................uucaauggugcuggaUucgua............  1  1  seq
    ...............................................................................ucaauggugUuggagucgu.............  1  1  seq
    ..................................................................................auggugUuggagucgua............  1  1  seq
    ..................................................................................auggugUuggagucguaucgu........  2  1  seq
    ...................................................................................uggugUuggagucguaucgu........  2  1  seq
    ...................................................................................uggugUuggagucguaucgug.......  26  1  seq
    ........................................................................................cuggagucguGucgugg......  1  1  seq
```

Provisional ID	:	MA_10434003_2473
Score total	:	5780.8
Score for star read(s)	:	3.9
Score for read counts	:	5783.1
Score for mfe	:	-3.9
Score for randfold	:	-2.2
Score for cons. seed	:	
Total read count	:	11355
Mature read count	:	11354
Loop read count	:	0
Star read count	:	1

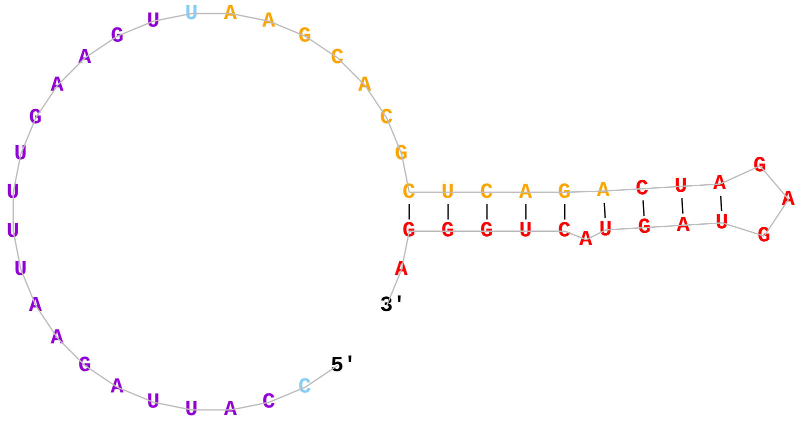

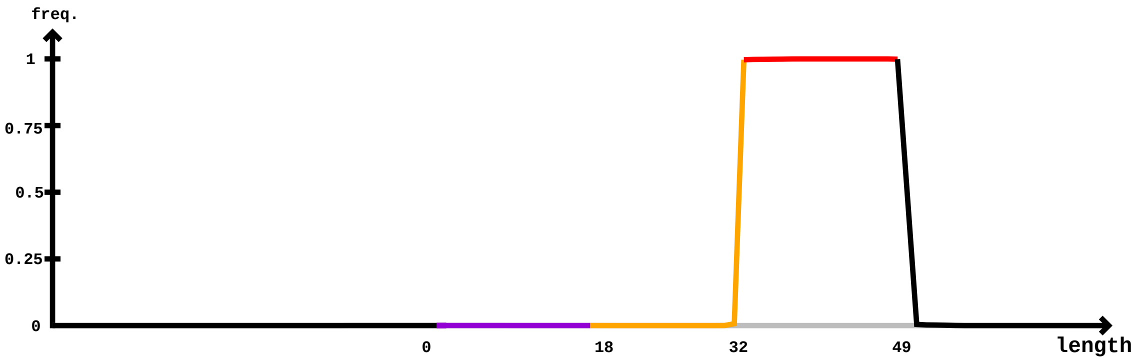


```
                                          Star                          Mature
5'- uuguacaugccuacaacauagugucaauucaccugaucccauuagaauuuugaaguuaagcacgcucagacuagaguaguacugggaagggugacuacuugggaaguc  -3'   obs
    uuguacaugccuacaacauagugucaauucaccugaucccauuagaauuuugaaguuaagcacgcucagacuagaguaguacugggaagggugacuacuugggaaguc         exp
    .........((((......((((.....((((((..(((((.((..(((((..((((.((....))..)))))))))..)).))))).)))))).)))))))).....   reads      mm      sample
    .............................caccugaucccauCaga..............................................................   1          1       seq
    .......................................cauuagaauuuugUagu....................................................   1          1       seq
    ....................................................................gacuagaguaguacuggga.....................   2          0       seq
    .....................................................................Ncuagaguaguacuggg......................   2          1       seq
    .....................................................................acAagaguaguacuggg......................   1          1       seq
    .....................................................................acuagaguaguacuggg......................   9          0       seq
    .....................................................................acGagaguaguacuggga.....................   1          1       seq
    .....................................................................acuagaguaguacuggga.....................   34         0       seq
    .....................................................................acuagaguaguacugggU.....................   1          1       seq
    .....................................................................Ncuagaguaguacuggga.....................   24         1       seq
    .....................................................................acuagaguaguacugggaC....................   3          1       seq
    .....................................................................acuagaguaguacugggaGg...................   1          1       seq
    ......................................................................cuagaguaguacuggga.....................   11182      0       seq
    ......................................................................cuagaguCguacuggga.....................   1          1       seq
    ......................................................................Nuagaguaguacuggga.....................   17         1       seq
    ......................................................................cuagaguaguacugggUa....................   1          1       seq
    ......................................................................cuagaguaguacugggaa....................   18         0       seq
    ......................................................................cCagaguaguacugggaa....................   1          1       seq
    ......................................................................cuagaguaguacugggGa....................   1          1       seq
    ......................................................................cuagGguaguacugggaa....................   4          1       seq
    ......................................................................cuagGguaguacugggaag...................   6          1       seq
    ......................................................................cuagaguaguacugggaaC...................   1          1       seq
    ......................................................................cuagaguaguacugggaag...................   6          0       seq
    ......................................................................cuagaguaguacugAgaag...................   1          1       seq
    ......................................................................cuagaguaguacugggaaA...................   1          1       seq
    ......................................................................cuagaguaguacugggaaCg..................   1          1       seq
    ......................................................................cuagaguaguacugggaagg..................   2          0       seq
    ......................................................................cuagaguaguacugggaaggg.................   3          0       seq
    ......................................................................cuagaguaguacugggaaggC.................   1          1       seq
    .......................................................................uagGguaguacugggaa....................   2          1       seq
    .......................................................................uagaguaguacugggaa....................   5          0       seq
    .......................................................................Cagaguaguacugggaag...................   1          1       seq
    .......................................................................uagaguaguacugggaag...................   3          0       seq
```

```
                                             Star                   Mature
uuguacaugccuacaacauagugucaauucaccugaucccauuagaauuuugaaguuaagcacgcucagacuagaguaguacugggaagggugacuacuugggaaguc
.......................................................................uagaguaguacugggaagggug...............	1	0	seq
........................................................................agaguaguacugggaag...................	1	0	seq
........................................................................agGguaguacugggaag...................	1	1	seq
........................................................................agaguaguacugggaaA...................	1	1	seq
........................................................................agaguaguacugggaaggCu................	1	1	seq
.........................................................................gaguaguacugggaagA..................	1	1	seq
.........................................................................gaguaguacugggaaggg.................	2	0	seq
.........................................................................gaguaguacugggaagggA................	1	1	seq
.........................................................................gaguaguacugggaagggug...............	1	0	seq
..........................................................................aguaguacugggaaggg.................	2	0	seq
..........................................................................aguaguacugggaagggu................	2	0	seq
..........................................................................aguaguacugggaagggC................	1	1	seq
...........................................................................guaguacugggaagggu................	3	0	seq
...........................................................................guaguacugggaagggC................	2	1	seq
...........................................................................guaguacugggaagggGg...............	1	1	seq
...........................................................................guaguacugggaagggug...............	1	0	seq
............................................................................uaguacugggaaggguU...............	1	1	seq
```

Provisional ID	:	MA_10434800_26919
Score total	:	191.6
Score for star read(s)	:	3.9
Score for read counts	:	192.8
Score for mfe	:	-2.8
Score for randfold	:	-2.2
Score for cons. seed	:	
Total read count	:	390
Mature read count	:	383
Loop read count	:	0
Star read count	:	7

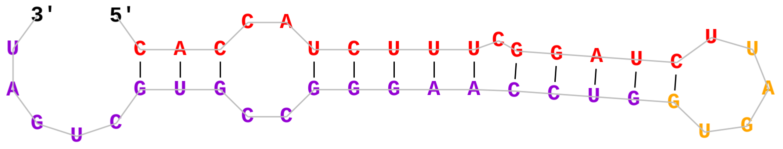

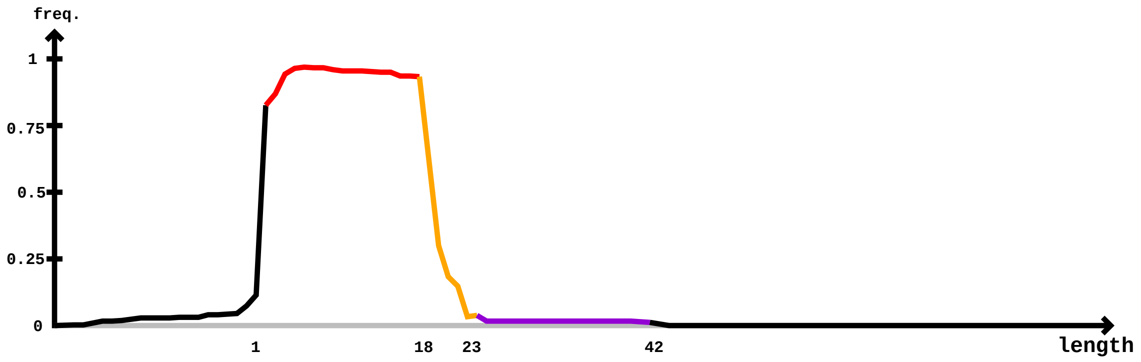

Mature **Star**

```
5'- ugaugaaauuguaaacauaacaccaucuuucggaucuuagugguccaagggccgugcugauaguuugcacuucugaggcaauauuuuaaaguaauuggaugucugcga  -3'  obs
    ugaugaaauuguaaacauaacaccaucuuucggaucuuagugguccaagggccgugcugauaguuugcacuucugaggcaauauuuuaaaguaauuggaugucugcga        exp
    ....(((..(((((((((..(((..(((((.(((((.....))))))))))..)))...)).))))))).)))....((((((((...((....)).))))).))).. reads  mm  sample
    Ggaugaaauuguaaaca........................................................................................... 1  1  seq
    ..augaaauuguaaacauaUca...................................................................................... 1  1  seq
    ..augaaauuguaaacauaUcaccauc................................................................................. 1  1  seq
    ..augaaauuguaaacauaUcaccaucuuucgga.......................................................................... 1  1  seq
    ...ugaaauuguaaacauaUc....................................................................................... 1  1  seq
    ...ugaaauuguaaacauaUcacc.................................................................................... 1  1  seq
    ...ugaaauuguaaacauaUcaccauc................................................................................. 1  1  seq
    .....aaauuguaaacauaUcaccauc................................................................................. 1  1  seq
    ......aauuguaaacauaUcacc.................................................................................... 1  1  seq
    ......aauuguaaacauaUcacca................................................................................... 1  1  seq
    .......auuguaaacauaUcacc.................................................................................... 1  1  seq
    .......auuguaaacauaUcaccaucu................................................................................ 1  1  seq
    ...........uaaacauaUcaccaucu................................................................................ 1  1  seq
    ..............acauaUcaccaucuuuc............................................................................. 1  1  seq
    ..............acauaUcaccaucuuucg............................................................................ 1  1  seq
    ..............acauaUcaccaucuuucgga.......................................................................... 2  1  seq
    ................auaUcaccaucuuucgga.......................................................................... 1  1  seq
    .................uaUcaccaucuuucgga.......................................................................... 2  1  seq
    ..................Cacaccaucuuucggaucuu...................................................................... 6  1  seq
    ..................Cacaccaucuuucggaucuua..................................................................... 6  1  seq
    ...................acaccaucuuucggauc........................................................................ 1  0  seq
    ...................acaccaucuuucggaucu....................................................................... 2  0  seq
    ...................acaccaucuuucggaucuu...................................................................... 3  0  seq
    ...................acaccaucuuucggaucuua..................................................................... 1  0  seq
    ...................acaccaucuuucggaucuuaU.................................................................... 1  1  seq
    ...................acaccaucuuucggaucuuaUu................................................................... 8  1  seq
    ...................acaccaucuuucggaucuuaUugg................................................................. 1  1  seq
    ....................caccaucCuucggaucu....................................................................... 2  1  seq
    ....................caccaucuuucggaAcu....................................................................... 40 1  seq
    ....................caccaucuuucggaucu....................................................................... 94 0  seq
    ....................caccaucuuCcggaucu....................................................................... 1  1  seq
    ....................caccaucuuucggaucC....................................................................... 25 1  seq
    ....................caccaucuuucggaucG....................................................................... 5  1  seq
```

Mature **Star**

```
ugaugaaauuguaaacauaacaccaucuuucggaucuuagugguccaagggccgugcugauaguuugcacuucugaggcaauauuuuaaaguaauuggaugucugcga
....................caccaucuuucggaucA.......................................................................   4   1   seq
....................caccaucuuucggaCcu.......................................................................   1   1   seq
....................caccauUuuucggaucu.......................................................................   1   1   seq
....................caccaucuuucggaucuu......................................................................   47  0   seq
....................caccaucuuucggaucAu......................................................................   1   1   seq
....................caccaucuuucggaAcuu......................................................................   14  1   seq
....................caccaucuuucggaucuC......................................................................   4   1   seq
....................caccaucuuucggaucuA......................................................................   1   1   seq
....................caccaucuuCcggaucuu......................................................................   1   1   seq
....................caccaucuuucggaAcuua.....................................................................   5   1   seq
....................caccaucuuucggaucuua.....................................................................   21  0   seq
....................caccaucuuucggaGcuua.....................................................................   1   1   seq
....................caccaucuuucggaucuuaU....................................................................   6   1   seq
....................caccaucuuucggaucuuaC....................................................................   1   1   seq
....................caccaucuuucggaucuuag....................................................................   1   0   seq
....................caccaucuuucggaucuuaUu...................................................................   16  1   seq
....................caccaucuuucggaucuuaUug..................................................................   2   1   seq
....................caccaucuuucggaucuuaUugg.................................................................   5   1   seq
.....................accaucuuucggaAcuu......................................................................   7   1   seq
.....................accaucuuucggaucuu......................................................................   5   0   seq
.....................accaucuuucggaucuC......................................................................   1   1   seq
.....................accaucuCucggaucuu......................................................................   1   1   seq
.....................accaucuuucggaAcuua.....................................................................   2   1   seq
.....................accaucuuucggaucuuaUu...................................................................   2   1   seq
.....................accaucuuucggaucuuaUugg.................................................................   1   1   seq
......................ccaucuuucggaucuua.....................................................................   11  0   seq
......................ccaucuuucggaAcuua.....................................................................   2   1   seq
......................ccaucuuucggaucuuaU....................................................................   3   1   seq
......................ccaucuuucggaucuuaUu...................................................................   13  1   seq
......................ccaucuuucggaucuuaUug..................................................................   2   1   seq
......................ccaucuuucggaucuuaUuggu................................................................   1   1   seq
.......................caucuuucggaucuuaC....................................................................   1   1   seq
.......................caucuuucggaucuuaU....................................................................   2   1   seq
.......................caucuuucggaucuuaUu...................................................................   6   1   seq
........................aucuuucggaucuuaUu...................................................................   3   1   seq
........................aucuuucggaucuuaUugg.................................................................   2   1   seq
..........................................gCccaagggccgugcug.................................................   1   1   seq
..........................................gCccaagggccgugcuga................................................   1   1   seq
..........................................gCccaagggccgugcugau...............................................   4   1   seq
...........................................ccaagggccgugcugauC...............................................   1   1   seq
```

Provisional ID	:	MA_10435728_7932
Score total	:	464.1
Score for star read(s)	:	3.9
Score for read counts	:	466.6
Score for mfe	:	-4.1
Score for randfold	:	-2.2
Score for cons. seed	:	
Total read count	:	927
Mature read count	:	921
Loop read count	:	0
Star read count	:	6

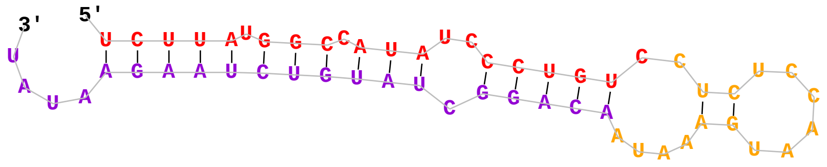

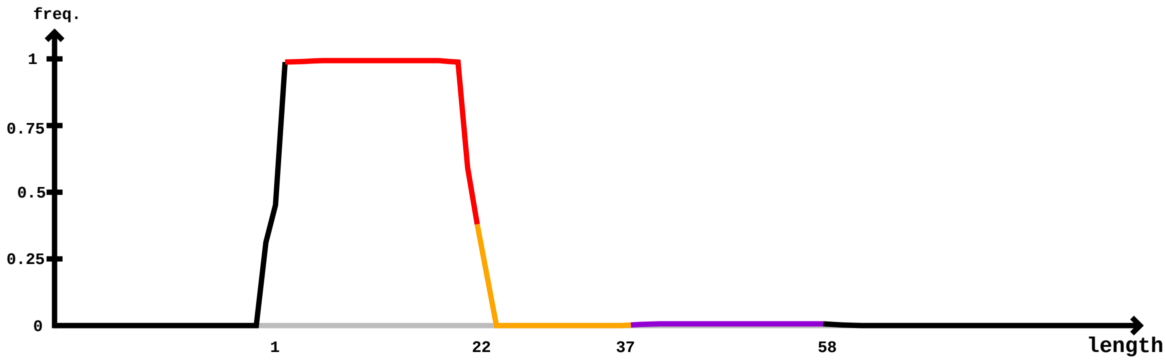

Mature Star

```
5'- uccagccugaaccgucgcaaauucuuauggccauaucccugucucuccaaugaaauaacaggcuaugucuaagaauauuugucguuccucuucaucaagugcaugguaac -3' obs
    uccagccugaaccgucgcaaauucuuauggccauaucccugucucuccaaugaaauaacaggcuaugucuaagaauauuugucguuccucuucaucaagugcaugguaac      exp
    ..........(((((.(((.(((((((.(((.(((..(((((..((......))....))))).)))))))))))))..(((..(........)..))).))))))))...  reads  mm  sample
    ....................auucuuauggccGuauccc........................................................................  1      1   seq
    ....................auucuuauggccGuaucccug......................................................................  283    1   seq
    ....................auucuuauggccauaucccug......................................................................  4      0   seq
    ....................auCcuuauggccauaucccug......................................................................  1      1   seq
    .....................uucuuauggccGuaucccug......................................................................  3      1   seq
    .....................uucuuauggccauaucccugu.....................................................................  1      0   seq
    .....................uucuuauggccGuaucccugu.....................................................................  125    1   seq
    .....................uGcuuauggccauaucccugu.....................................................................  1      1   seq
    ......................ucuuauggccGuauccc........................................................................  2      1   seq
    ......................ucuuauggccGuaucccu.......................................................................  2      1   seq
    ......................ucuuauggccGuaucccug......................................................................  76     1   seq
    ......................ucuuauggccGuaucccugu.....................................................................  70     1   seq
    ......................ucuuauggccGuaucccuguc....................................................................  347    1   seq
    .......................cuuauggccGuaucccuguc....................................................................  1      1   seq
    ........................uuauggccGuaucccuguc....................................................................  1      1   seq
    .........................uauggccGuaucccuguc....................................................................  2      1   seq
    ..........................auggccGuaucccuguc....................................................................  1      1   seq
    ..........................................................acaggcuaugucuaagaaCau................................  2      1   seq
    ...........................................................caggcGaugucuaagaauauu...............................  2      1   seq
    ............................................................aggcGaugucuaagaauauuu..............................  1      1   seq
    .............................................................ggcGaugucuaagaauauuug.............................  1      1   seq
```

Provisional ID	:	MA_10435792_7354
Score total	:	6590.9
Score for star read(s)	:	3.9
Score for read counts	:	6583
Score for mfe	:	2.4
Score for randfold	:	1.6
Score for cons. seed	:	
Total read count	:	12924
Mature read count	:	12349
Loop read count	:	0
Star read count	:	575

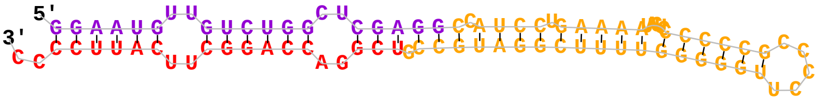

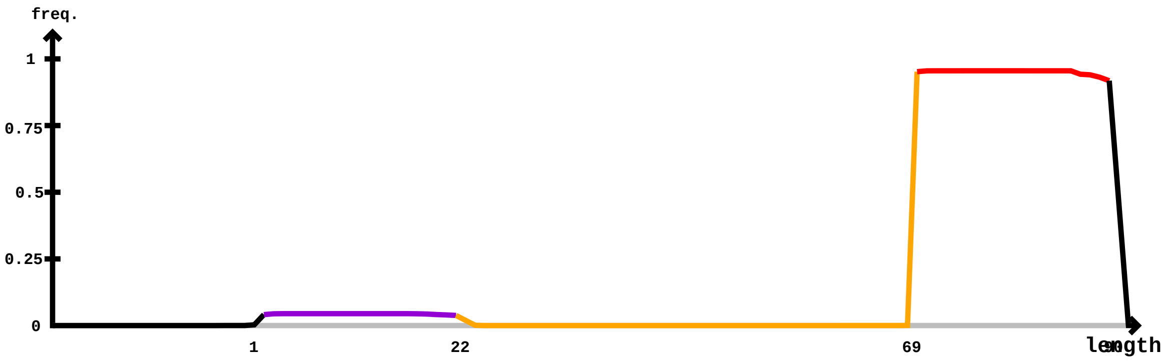

Star Mature

```
5'- aaggagaaauggguuugaggggaauguugucuggcucgaggccauccugaaaauaguccccccgccccuuggggguuuucggaugccgucggaccaggcuucauuccccuc  -3'   obs
    aaggagaaauggguuugaggggaauguugucuggcucgaggccauccugaaaauaguccccccgccccuuggggguuuucggaugccgucggaccaggcuucauuccccuc         exp
    ................((((((((((..((((((..((((((.((((.(((((.....((((((.....))))))))))))))))))).)))..))))))..))))))))))   reads   mm   sample
    ................gaggggaauguugucuggcuU..........................................................................   1       1    seq
    ................gaggggaauguugucuggcuc..........................................................................   1       0    seq
    ..................ggggaauguugucuggcu...........................................................................   1       0    seq
    ...................gggaauguugucuggcC...........................................................................   4       1    seq
    ...................gggaauguugucuggcu...........................................................................   1       0    seq
    ...................gggaauguugucuggcG...........................................................................   1       1    seq
    ...................gggaauguugucuggcuc..........................................................................   7       0    seq
    ...................gggaauguugucuggcucU.........................................................................   1       1    seq
    ...................gggaauguugucuggcucg.........................................................................   7       0    seq
    ...................gggaauguugucuggcucga........................................................................   1       0    seq
    ...................gggaauguugucuggcucgag.......................................................................   6       0    seq
    ...................gggaauguugucuggcucgaU.......................................................................   3       1    seq
    ...................gggaauguugucuggcucgagg......................................................................   1       0    seq
    ...................gggaauguugucuggcucgaCg......................................................................   1       1    seq
    ....................ggaauguugucuggcuc..........................................................................   2       0    seq
    ....................ggaauguugucuggcucg.........................................................................   17      0    seq
    ....................ggaauguugucuggcucA.........................................................................   1       1    seq
    ....................ggaauguugucuggcucga........................................................................   14      0    seq
    ....................ggaauguugucuggcucgG........................................................................   1       1    seq
    ....................ggaauguugucuggcucgag.......................................................................   14      0    seq
    ....................Ugaauguugucuggcucgag.......................................................................   1       1    seq
    ....................ggaauguugucuggcucgagC......................................................................   1       1    seq
    ....................ggaauguuguAuggcucgagg......................................................................   1       1    seq
    ....................ggaGuguugucuggcucgagg......................................................................   4       1    seq
    ....................ggaauguugucuggcucgagg......................................................................   379     0    seq
    ....................Cgaauguugucuggcucgagg......................................................................   1       1    seq
    ....................ggaauguCgucuggcucgagg......................................................................   1       1    seq
    ....................ggaauguugCcuggcucgagg......................................................................   1       1    seq
    ....................ggaauguugucuggcucgaAg......................................................................   2       1    seq
    ....................ggaauguugucuggcucgagU......................................................................   5       1    seq
    ....................ggaauguugucuggcucgGgg......................................................................   1       1    seq
    ....................ggaauguugucCggcucgagg......................................................................   1       1    seq
    ....................ggaauguugucuggcuUgagg......................................................................   1       1    seq
```

Star		Mature			
aaggagaaauggguuugaggggaauguugucuggcucgaggccauccugaaaauaguccccccgccccuuggggguuuucggaugccgucggaccaggcuucauucccccuc					
....................ggaauguugucuggcucgaCg..	16	1	seq		
....................ggaauguugucuggcucgagA..	27	1	seq		
....................ggGauguugucuggcucgagg..	1	1	seq		
....................ggaauguugucuggcucgaggc...	3	0	seq		
.....................gaauguugucuggcucA...	1	1	seq		
.....................gaauguugucuggcucgag...	1	0	seq		
.....................gaaCguugucuggcucgagg..	1	1	seq		
.....................gaauguugucuggcucgagg..	15	0	seq		
.....................gaauguugucuggcucgaggG...	2	1	seq		
.....................gaaugCugucuggcucgaggc...	1	1	seq		
.....................gaauguugucuggcucgaggc...	11	0	seq		
.....................gaauguugucuggcucgaggA...	2	1	seq		
.....................gaauguugucuggcucgaggcc..	4	0	seq		
.....................gaauguugucuggcucgaggcU..	1	1	seq		
......................aauguugucuggcucgaggcc..	3	0	seq		
......................aauguugucuggcucgaggcU..	3	1	seq		
.........................guugucuggcucgaggc...	1	0	seq		
....ggUugccgucggaccaggcu...........	1	1	seq		
....gucggaccaggcuucauuccA...	2	1	seq		
....gucggaccaggcuucauuccc...	2	0	seq		
....Cucggaccaggcuucauucccc..	1	1	seq		
....ucggaccaggcuucauC......	15	1	seq		
....ucggaccaggcuucauu......	146	0	seq		
....ucggacUaggcuucauu......	1	1	seq		
....ucggGccaggcuucauu......	1	1	seq		
....ucggaccaggcuucGuu......	2	1	seq		
....ucggaccaggcuucauA......	1	1	seq		
....ucggaccaggcuucaCu......	1	1	seq		
....ucggaccaggcuucauuA.....	1	1	seq		
....ucggaccaggcuucauuc.....	21	0	seq		
....ucggaccaggcuucauuU.....	4	1	seq		
....ucggaccaggcuucauucA....	4	1	seq		
....ucggaccaggcuucauucU....	10	1	seq		
....ucggaccaggcuucauucc....	88	0	seq		
....ucAgaccaggcuucauucc....	1	1	seq		
....ucggaccaggcuCcauucc....	1	1	seq		
....ucggacUaggcuucauucc....	1	1	seq		
....ucggaUcaggcuucauucc....	1	1	seq		
....ucggaccaggcuucauuUc....	1	1	seq		
....ucggaccaggcuucaCucc....	1	1	seq		
....ucggaccaggcuucauucG....	1	1	seq		
....ucggaccGggcuucauuccc...	1	1	seq		
....ucggaccaggcuucauucAc...	1	1	seq		
....ucgUaccaggcuucauuccc...	1	1	seq		
....ucggaccaggcuCcauuccc...	1	1	seq		
....ucggaAcaggcuucauuccc...	1	1	seq		
....uUggaccaggcuucauuccc...	1	1	seq		
....ucggacAaggcuucauuccc...	1	1	seq		
....ucCgaccaggcuucauuccc...	1	1	seq		
....ucggaccaggcuucauuccA...	4	1	seq		
....Ncggaccaggcuucauuccc...	1	1	seq		
....ucggaccaggcuucauCccc...	1	1	seq		
....ucggaccaggcuucaGuccc...	1	1	seq		
....ucggaccaggcuucauuccc...	147	0	seq		
....ucggaUcaggcuucauuccc...	1	1	seq		
....ucggaccaggcuucauuccNc..	1	1	seq		
....ucggaccaggcuAcauucccc..	10	1	seq		
....ucggaccaggcuucauAcccc..	3	1	seq		
....uUggaccaggcuucauucccc..	7	1	seq		
....ucCgaccaggcuucauucccc..	1	1	seq		
....ucggaccaAgcuucauucccc..	7	1	seq		
....ucggacUaggcuucauucccc..	10	1	seq		
....ucggaccaggcuucauuAccc..	2	1	seq		
....ucggaccaggcuuUauucccc..	9	1	seq		
....ucUgaccaggcuucauucccc..	2	1	seq		
....ucggaccaggcCucauucccc..	25	1	seq		
....ucggaccaggcuucauuUccc..	5	1	seq		
....ucggaccaggcuucaCucccc..	9	1	seq		
....ucggaccaggcuucauucccA..	323	1	seq		
....ucggaccaggcuucGuucccc..	34	1	seq		

Star	Mature

```
aaggagaaauggguuugaggggaauguugucuggcucgaggccauccugaaaauaguccccccgccccuuggggguuuucggaugccgucggaccaggcuucauucccc uc
........................................................................................ucggaccaggGuucauucccc..    4     1   seq
........................................................................................ucggaccaggAuucauucccc..    4     1   seq
........................................................................................ucggaccCggcuucauucccc..    2     1   seq
........................................................................................ucggaccaggcuucauucGcc..    1     1   seq
........................................................................................ucggaccaggcuucCuucccc..    4     1   seq
........................................................................................ucggaccaggcAucauucccc..    3     1   seq
........................................................................................Ncggaccaggcuucauucccc..    7     1   seq
........................................................................................ucggaccaggcuCcauucccc..    34    1   seq
........................................................................................ucggaccaggcuucauucccc..    8863  0   seq
........................................................................................ucggaccaggcuucauucccU..    1444  1   seq
........................................................................................ucggaccaggcuucUuucccc..    1     1   seq
........................................................................................ucggaccagCcuucauucccc..    1     1   seq
........................................................................................ucggaccaggcuucaGucccc..    3     1   seq
........................................................................................ucggGccaggcuucauucccc..    33    1   seq
........................................................................................ucggaccaggcuucauuccAc..    7     1   seq
........................................................................................ucggaccaggcuGcauucccc..    1     1   seq
........................................................................................ucggaccaggcuuAauucccc..    1     1   seq
........................................................................................ucggaccagAcuucauucccc..    3     1   seq
........................................................................................ucggaccaCgcuucauucccc..    1     1   seq
........................................................................................ucggaGcaggcuucauucccc..    1     1   seq
........................................................................................ucggaccaggcuucauucUcc..    11    1   seq
........................................................................................ucggUccaggcuucauucccc..    2     1   seq
........................................................................................Gcggaccaggcuucauucccc..    12    1   seq
........................................................................................ucAgaccaggcuucauucccc..    9     1   seq
........................................................................................ucggaccaggcuucauucccG..    47    1   seq
........................................................................................ucggaccaggcuucauGcccc..    1     1   seq
........................................................................................ucggaccGggcuucauucccc..    20    1   seq
........................................................................................ucggaAcaggcuucauucccc..    4     1   seq
........................................................................................ucggaccaggUuucauucccc..    5     1   seq
........................................................................................Acggaccaggcuucauucccc..    7     1   seq
........................................................................................ucggaccaggcuucauucAcc..    10    1   seq
........................................................................................ucggaccaggcuucauCcccc..    40    1   seq
........................................................................................uAggaccaggcuucauucccc..    1     1   seq
........................................................................................ucggaccagUcuucauucccc..    1     1   seq
........................................................................................ucggaccaUgcuucauucccc..    3     1   seq
........................................................................................ucgUaccaggcuucauucccc..    2     1   seq
........................................................................................ucggaccaggcuucauuGccc..    1     1   seq
........................................................................................ucggaccUggcuucauucccc..    5     1   seq
........................................................................................ucggaUcaggcuucauucccc..    4     1   seq
........................................................................................ucgAaccaggcuucauucccc..    5     1   seq
........................................................................................ucggaccaggcuucauuccccG.    21    1   seq
........................................................................................ucgAaccaggcuucauuccccu.    1     1   seq
........................................................................................ucggaccaggcuucauuccccA.    301   1   seq
........................................................................................ucggaccaggcCucauuccccu.    2     1   seq
........................................................................................ucggaccaggcuucauucGccu.    1     1   seq
........................................................................................ucggaccaggcuucauuccccu.    430   0   seq
........................................................................................ucggUccaggcuucauuccccu.    1     1   seq
........................................................................................ucggaccaggGuucauuccccu.    1     1   seq
........................................................................................ucAgaccaggcuucauuccccu.    2     1   seq
........................................................................................ucggaccaggcuucGuuccccu.    7     1   seq
........................................................................................ucggaccaggcuuUauuccccu.    1     1   seq
........................................................................................ucggaccaggcuucaCuccccu.    1     1   seq
........................................................................................Ncggaccaggcuucauuccccu.    2     1   seq
........................................................................................Acggaccaggcuucauuccccu.    1     1   seq
........................................................................................ucggaccaggcuucauucccUu.    1     1   seq
........................................................................................ucggaccaggcuucauuccccuA    2     1   seq
........................................................................................ucggaccaggcuucauuccccuG    4     1   seq
........................................................................................ucggaccaggcuucauuccccuc    1     0   seq
.........................................................................................cggaccaggcuuUauuc.....    1     1   seq
.........................................................................................cggaccaggcuucauucc....    6     0   seq
.........................................................................................Uggaccaggcuucauucc....    1     1   seq
.........................................................................................cggaccaggcuucauuccc...    4     0   seq
.........................................................................................cggaccaggcuucauucccc..    8     0   seq
.........................................................................................cggaccaggcuucauucccU..    5     1   seq
.........................................................................................cggaccaggcuucauucccAu.    1     1   seq
.........................................................................................cggaccaggcuucauuccccu.    10    0   seq
.........................................................................................cggaccaggcuucauuccccA.    3     1   seq
..........................................................................................ggaccaggcuucauucccc..    3     0   seq
.............................................................................................ccaggcuucauuccccu.    1     0   seq
```

Provisional ID : MA_10435792_7356
Score total : 6420.7
Score for star read(s) : 3.9
Score for read counts : 6412.7
Score for mfe : 2.5
Score for randfold : 1.6
Score for cons. seed :
Total read count : 12590
Mature read count : 11950
Loop read count : 5
Star read count : 635

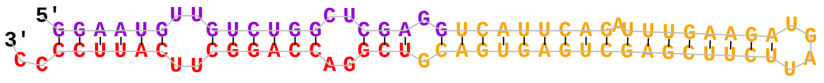

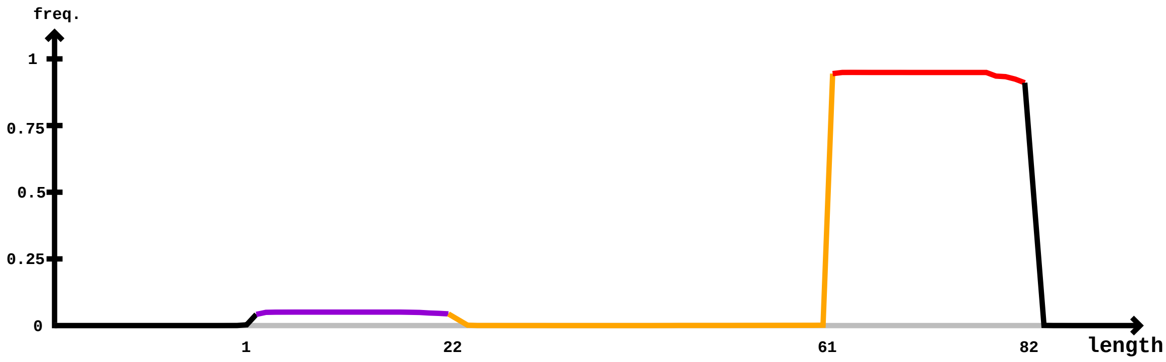

Star　　　　　　　　　　Mature

```
5'- ucuugguuuaugauugcggggaauguugucuggcucgaggucauucagauuugaagaugauucuucgagcugagugacgucggaccaggcuucauuccccccaacugugau  -3'  obs
    ucuugguuuaugauugcggggaauguugucuggcucgaggucauucagauuugaagaugauucuucgagcugagugacgucggaccaggcuucauuccccccaacugugau       exp
    .......((((..(((.((((((((..((((((..(((.(((((((((.((((((((....))))))))))))))))).)))..))))))..)))))))).)))..)))).  reads  mm  sample
    ................cggggaauguugucuggcucgaggucauCcagau..............................................................  1  1  seq
    .................ggggaauguugucuggcu.............................................................................  1  0  seq
    ..................gggaauguugucuggcC.............................................................................  4  1  seq
    ..................gggaauguugucuggcu.............................................................................  1  0  seq
    ..................gggaauguugucuggcG.............................................................................  1  1  seq
    ..................gggaauguugucuggcuc............................................................................  7  0  seq
    ..................gggaauguugucuggcucU...........................................................................  1  1  seq
    ..................gggaauguugucuggcucg...........................................................................  7  0  seq
    ..................gggaauguugucuggcucga..........................................................................  1  0  seq
    ..................gggaauguugucuggcucgag.........................................................................  6  0  seq
    ..................gggaauguugucuggcucgaU.........................................................................  3  1  seq
    ..................gggaauguugucuggcucgagg........................................................................  1  0  seq
    ..................gggaauguugucuggcucgaCg........................................................................  1  1  seq
    ...................ggaauguugucuggcuc............................................................................  2  0  seq
    ...................ggaauguugucuggcucA...........................................................................  1  1  seq
    ...................ggaauguugucuggcucg...........................................................................  17  0  seq
    ...................ggaauguugucuggcucga..........................................................................  14  0  seq
    ...................ggaauguugucuggcucgG..........................................................................  1  1  seq
    ...................Ugaauguugucuggcucgag.........................................................................  1  1  seq
    ...................ggaauguugucuggcucgag.........................................................................  14  0  seq
    ...................ggaauguugucuggcucgaCg........................................................................  16  1  seq
    ...................ggaauguugucCggcucgagg........................................................................  1  1  seq
    ...................ggaauguugucuggcucgagU........................................................................  5  1  seq
    ...................ggaGuguugucuggcucgagg........................................................................  4  1  seq
    ...................ggaauguugucuggcucgagg........................................................................  379  0  seq
    ...................ggaauguugucuggcucgagC........................................................................  1  1  seq
    ...................Cgaauguugucuggcucgagg........................................................................  1  1  seq
    ...................ggaauguugucuggcucgaAg........................................................................  2  1  seq
    ...................ggaauguugucuggcucgagA........................................................................  27  1  seq
    ...................ggGauguugucuggcucgagg........................................................................  1  1  seq
    ...................ggaauguugCcuggcucgagg........................................................................  1  1  seq
    ...................ggaauguuguAuggcucgagg........................................................................  1  1  seq
    ...................ggaauguCgucuggcucgagg........................................................................  1  1  seq
```

Star   Mature

```
ucuugguuuaugauugcggggaauguugucuggcucgaggucauucagauuugaagaugauucuucgagcugagugacgucggaccaggcuucauuccccccaacugugau
...................ggaauguugucuggcucgGgg.......................................................................	1	1	seq
...................ggaauguugucuggcuUgagg.......................................................................	1	1	seq
...................ggaauguugucuggcucgaggu......................................................................	1	0	seq
....................gaauguugucuggcucA..........................................................................	1	1	seq
....................gaauguugucuggcucgag........................................................................	1	0	seq
....................gaaCguugucuggcucgagg.......................................................................	1	1	seq
....................gaauguugucuggcucgagg.......................................................................	15	0	seq
....................gaauguugucuggcucgaggG......................................................................	2	1	seq
....................gaauguugucuggcucgaggu......................................................................	67	0	seq
....................gaauguugucuggcucgUggu......................................................................	1	1	seq
....................gaauguugucuggcucgaggA......................................................................	2	1	seq
....................gaauguuCucuggcucgaggu......................................................................	1	1	seq
....................gaauguugucuggcucgagguA.....................................................................	1	1	seq
....................gaauguugucuggcucgagguU.....................................................................	1	1	seq
....................gaauguugucuggcucgagguc.....................................................................	4	0	seq
.....................aauguugucuggcucgagguA.....................................................................	1	1	seq
.....................aauguugucuggcucgagguc.....................................................................	6	0	seq
.....................aauguugucuggcucgagguU.....................................................................	2	1	seq
......................auguAgucuggcucgaggu......................................................................	1	1	seq
.......................uguugucuggcucgaggucauu..................................................................	1	0	seq
.............................................................ucuucAagcugagugacg................................	1	1	seq
..............................................................cuucAagcugagugacgu...............................	1	1	seq
...............................................................uucAagcugagugacguc..............................	1	1	seq
...............................................................uucAagcugagugacgucg.............................	1	1	seq
...............................................................uucAagcugagugacgucgg............................	1	1	seq
.....................................................................cugagugacgucggacca........................	1	0	seq
......................................................................ugagugacAucggaccaggcu....................	1	1	seq
........................................................................agugacgucggaccaggcuucauu...............	2	0	seq
..........................................................................ugacgucggaccaggcuucauu...............	1	0	seq
............................................................................acgucggaccaggcuucauucccc...........	1	0	seq
..............................................................................gucggaccaggcuucauuccc............	2	0	seq
..............................................................................gucggaccaggcuucauuccA............	2	1	seq
..............................................................................Cucggaccaggcuucauucccc...........	1	1	seq
...............................................................................ucggGccaggcuucauu...............	1	1	seq
...............................................................................ucggaccaggcuucauA...............	1	1	seq
...............................................................................ucggaccaggcuucGuu...............	2	1	seq
...............................................................................ucggaccaggcuucauu...............	146	0	seq
...............................................................................ucggacUaggcuucauu...............	1	1	seq
...............................................................................ucggaccaggcuucaCu...............	1	1	seq
...............................................................................ucggaccaggcuucauC...............	15	1	seq
...............................................................................ucggaccaggcuucauuA..............	1	1	seq
...............................................................................ucggaccaggcuucauuU..............	4	1	seq
...............................................................................ucggaccaggcuucauuc..............	21	0	seq
...............................................................................ucggaccaggcuCcauucc.............	1	1	seq
...............................................................................ucggaccaggcuucauucU.............	10	1	seq
...............................................................................ucggaccaggcuucaCucc.............	1	1	seq
...............................................................................ucggaccaggcuucauuUc.............	1	1	seq
...............................................................................ucggaccaggcuucauucc.............	88	0	seq
...............................................................................ucggaUcaggcuucauucc.............	1	1	seq
...............................................................................ucggaccaggcuucauucG.............	1	1	seq
...............................................................................ucggaccaggcuucauucA.............	4	1	seq
...............................................................................ucggacUaggcuucauucc.............	1	1	seq
...............................................................................ucAgaccaggcuucauucc.............	1	1	seq
...............................................................................ucggaccaggcuCcauuccc............	1	1	seq
...............................................................................ucgUaccaggcuucauuccc............	1	1	seq
...............................................................................uUggaccaggcuucauuccc............	1	1	seq
...............................................................................ucggaccaggcuucauucAc............	1	1	seq
...............................................................................Ncggaccaggcuucauuccc............	1	1	seq
...............................................................................ucggaAcaggcuucauuccc............	1	1	seq
...............................................................................ucggaccaggcuucauuccc............	147	0	seq
...............................................................................ucCgaccaggcuucauuccc............	1	1	seq
...............................................................................ucggaccaggcuucauCccc............	1	1	seq
...............................................................................ucggaccaggcuucaGuccc............	1	1	seq
...............................................................................ucggaccGggcuucauuccc............	1	1	seq
...............................................................................ucggaUcaggcuucauuccc............	1	1	seq
...............................................................................ucggacAaggcuucauuccc............	1	1	seq
...............................................................................ucggaccaggcuucauuccA............	4	1	seq
...............................................................................ucggaccaggcuCcauucccc...........	34	1	seq
...............................................................................ucggaccaggcuucauuUccc...........	5	1	seq
...............................................................................Acggaccaggcuucauucccc...........	7	1	seq
```

Star	Mature			
ucuugguuuaugauugcggggaauguugucuggcucgaggucauucagauuugaagaugauucuucgagcugagugacgucggaccaggcuucauuccccccaacugugau				
....ucggaccaggcuucauucAcc...........	10	1	seq	
....ucggaccaggcuucaGucccc...........	3	1	seq	
....ucggaccaggcuucauuccNc...........	1	1	seq	
....ucggaUcaggcuucauucccc...........	4	1	seq	
....uUggaccaggcuucauucccc...........	7	1	seq	
....ucggaccaggcuucUuucccc...........	1	1	seq	
....ucgUaccaggcuucauucccc...........	2	1	seq	
....ucggaccaggcuucaCucccc...........	9	1	seq	
....ucggaccaggcuucCuucccc...........	4	1	seq	
....ucggaccaggcuucauuGccc...........	1	1	seq	
....ucggaccaggcuuAauucccc...........	1	1	seq	
....ucggaccaggcCucauucccc...........	25	1	seq	
....ucAgaccaggcuucauucccc...........	9	1	seq	
....ucggaccCggcuucauucccc...........	2	1	seq	
....ucggaccaggcuucauuccAc...........	7	1	seq	
....ucggaccaCgcuucauucccc...........	1	1	seq	
....ucUgaccaggcuucauucccc...........	2	1	seq	
....ucggaccaggcuucauGcccc...........	1	1	seq	
....ucggaccaggcuucGuucccc...........	34	1	seq	
....ucggaccaggGuucauucccc...........	4	1	seq	
....uAggaccaggcuucauucccc...........	1	1	seq	
....Gcggaccaggcuucauucccc...........	12	1	seq	
....ucggaccaggcuucauuAccc...........	2	1	seq	
....ucggUccaggcuucauucccc...........	2	1	seq	
....ucggGccaggcuucauucccc...........	33	1	seq	
....ucggaccagUcuucauucccc...........	1	1	seq	
....ucggaccaggcuucauucGcc...........	1	1	seq	
....ucggaccaggcuucauucccG...........	47	1	seq	
....ucggaAcaggcuucauucccc...........	4	1	seq	
....ucggaGcaggcuucauucccc...........	1	1	seq	
....ucggacUaggcuucauucccc...........	10	1	seq	
....ucggaccaggcuucauucccU...........	1444	1	seq	
....ucggaccaAgcuucauucccc...........	7	1	seq	
....ucggaccagCcuucauucccc...........	1	1	seq	
....ucggaccGggcuucauucccc...........	20	1	seq	
....ucggaccUggcuucauucccc...........	5	1	seq	
....ucggaccaggcuGcauucccc...........	1	1	seq	
....ucggaccaggcuucauCcccc...........	40	1	seq	
....ucggaccaggcuucauucUcc...........	11	1	seq	
....ucggaccaggcuuUauucccc...........	9	1	seq	
....ucggaccaggcuAcauucccc...........	10	1	seq	
....ucggaccaggcuucauAcccc...........	3	1	seq	
....ucggaccaggcuucauucccc...........	8863	0	seq	
....ucggaccaggcuucauucccA...........	323	1	seq	
....ucgAaccaggcuucauucccc...........	5	1	seq	
....ucggaccaggUuucauucccc...........	5	1	seq	
....ucggaccagAcuucauucccc...........	3	1	seq	
....ucggaccaggAuucauucccc...........	4	1	seq	
....ucCgaccaggcuucauucccc...........	1	1	seq	
....Ncggaccaggcuucauucccc...........	7	1	seq	
....ucggaccaUgcuucauucccc...........	3	1	seq	
....ucggaccaggcAucauucccc...........	3	1	seq	
....ucggaccaggcuucauuccccA..........	301	1	seq	
....ucggaccaggcuucauuccccG..........	21	1	seq	
....ucggaccaggcuucauuccGcc..........	1	1	seq	
....ucggaccaggcuucauuccccc..........	38	0	seq	
....ucggaccaggcCucauuccccc..........	1	1	seq	
....ucggaccaggcuucauuUcccc..........	1	1	seq	
....ucggaccaggcuucauucccccA.........	1	1	seq	
....ucggaccaggcuucauuccccccaC.......	1	1	seq	
....cggaccaggcuuUauuc..............	1	1	seq	
....Uggaccaggcuucauucc.............	1	1	seq	
....cggaccaggcuucauucc.............	6	0	seq	
....cggaccaggcuucauuccc............	4	0	seq	
....cggaccaggcuucauucccU...........	5	1	seq	
....cggaccaggcuucauucccc...........	8	0	seq	
....cggaccaggcuucauuccccc..........	23	0	seq	
....cggaccaggcuucauuccccA..........	3	1	seq	
....cggaccaggcuucauucccccc.........	1	0	seq	
....cggaccaggcuucauuccccccU.........	2	1	seq	

Star Mature

```
ucuugguuuaugauugcggggaauguugucuggcucgaggucauucagauuugaagaugauucuucgagcugagugacgucggaccaggcuucauuccccccaacugugau
..............................................................................cggaccaggcuucauucccccA.........	2	1	seq
...............................................................................ggaccaggcuucauucccc...........	3	0	seq
```

Provisional ID	:	MA_10436275_7981
Score total	:	0
Score for star read(s)	:	-1.3
Score for read counts	:	0
Score for mfe	:	-0.3
Score for randfold	:	1.6
Score for cons. seed	:	
Total read count	:	97468
Mature read count	:	97468
Loop read count	:	0
Star read count	:	0

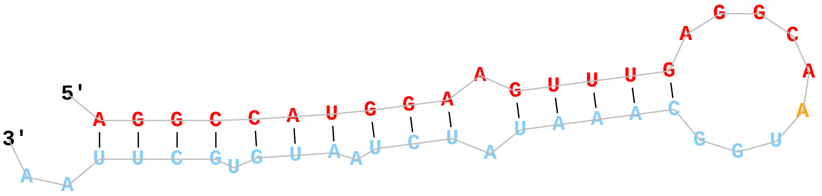

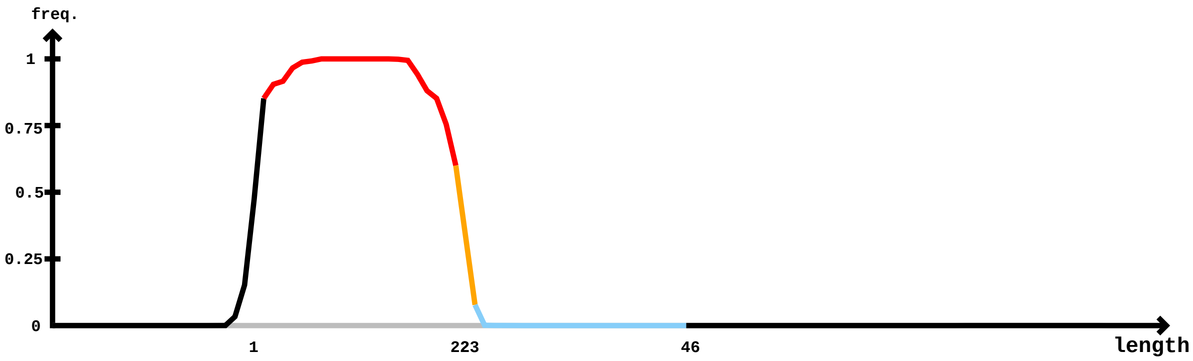

Mature **Star**

```
5'- uaaaaagacuauggucauuuaggccauggaaguuugaggcaauggcaaauaucuaaugugcuuaaauguucaaaauugcacaagcacuacacuaaugcagugaacgaguuuuuc   -3'    exp
    ...(((((((.((..((((((((((((((.(((((.........))))).))).))).)))))))))..))..((((((..((.......))..))))))).....))))))).   reads    mm    sample
    ...............cUuuuaggccauggaaguuug..............................................................................   1        1     seq
    ...............cauCuaggccauggaaguuug..............................................................................   1        1     seq
    ...............cauuuaggccauggaaguuuga.............................................................................   1        0     seq
    ...............Aauuuaggccauggaaguuugagg...........................................................................   1        1     seq
    ...............cUuuuaggccauggaaguuugagg...........................................................................   1        1     seq
    ...............cUuuuaggccauggaaguuugaggc..........................................................................   1        1     seq
    ...............cGuuuaggccauggaaguuugaggcaaug......................................................................   1        1     seq
    ................aAuuaggccauggaagu.................................................................................   1        1     seq
    ................Nuuuaggccauggaaguu................................................................................   1        1     seq
    ................Uuuuaggccauggaaguuugagg...........................................................................   2        1     seq
    ................Uuuuaggccauggaaguuugaggc..........................................................................   1        1     seq
    ................auGuaggccauggaaguuugaggc..........................................................................   1        1     seq
    ................Nuuuaggccauggaaguuugaggca.........................................................................   1        1     seq
    ................Uuuuaggccauggaaguuugaggca.........................................................................   2        1     seq
    ................Guuuaggccauggaaguuugaggcaaug......................................................................   1        1     seq
    .................uuuGggccauggaaguu................................................................................   1        1     seq
    .................uuuaggccauggaagAu................................................................................   2        1     seq
    .................uuuaggccauggaaguG................................................................................   2        1     seq
    .................Auuaggccauggaaguu................................................................................   1        1     seq
    .................uuuaggccauggaaguu................................................................................   127      0     seq
    .................uuuaggcUauggaaguu................................................................................   1        1     seq
    .................uuuaggccauggaagGu................................................................................   1        1     seq
    .................uuuaggccaCggaaguu................................................................................   1        1     seq
    .................uuCaggccauggaaguu................................................................................   1        1     seq
    .................uuuaggccauggaaguuu...............................................................................   81       0     seq
    .................uuuaggccauggGaguuu...............................................................................   1        1     seq
    .................uuuaggccauggaaguAu...............................................................................   1        1     seq
    .................uuuaggccGuggaaguuu...............................................................................   1        1     seq
    .................uuuaggccauggUaguuu...............................................................................   1        1     seq
    .................uuuaggccauggaaguCu...............................................................................   2        1     seq
    .................uuuaggccauggaaguuC...............................................................................   5        1     seq
    .................uuuaggcUauggaaguuug..............................................................................   2        1     seq
    .................uuuaggccauggaaguuuA..............................................................................   11       1     seq
    .................Guuaggccauggaaguuug..............................................................................   1        1     seq
```

Mature **Star**

uaaaaagacuauggucauuuaggccauggaaguuugaggcaauggcaaauaucuaaugugcuuaaauguucaaaauugcacaagcacuacacuaaugcagugaacgaguuuuuc			
.................uuuaggccauggaaguuug..	390	0	seq
.................Cuuaggccauggaaguuug..	2	1	seq
.................uuCaggccauggaaguuug..	1	1	seq
.................uuuaggccauggaaguuCg..	1	1	seq
.................uuuaggccauggaaguuuC..	1	1	seq
.................uuuaggccaAggaaguuug..	1	1	seq
.................uuuaggccauggaGguuug..	1	1	seq
.................uuuaggccauggGaguuug..	4	1	seq
.................uuuGggccauggaaguuug..	1	1	seq
.................Cuuaggccauggaaguuuga...	1	1	seq
.................uuuaggccauggGaguuuga...	1	1	seq
.................uuuaggccauggaaguuugG...	12	1	seq
.................uuuaggccaCggaaguuuga...	1	1	seq
.................uuuaggccauggaagCuuga...	1	1	seq
.................uuCaggccauggaaguuuga...	1	1	seq
.................uuuaggccauggaaguuuga...	327	0	seq
.................uuuaggccauggaaguuugU...	2	1	seq
.................uuuaggccGuggaaguuuga...	1	1	seq
.................uuuaggccauggaaguuugGg..	2	1	seq
.................uuuaggccauggaaguuugaC..	1	1	seq
.................Cuuaggccauggaaguuugag..	1	1	seq
.................uuuaggccUuggaaguuugag..	1	1	seq
.................Auuaggccauggaaguuugag..	1	1	seq
.................uuuaggccauggGaguuugag..	1	1	seq
.................uuuaggccauggaGguuugag..	2	1	seq
.................uuuaggccauggaaguuugag..	197	0	seq
.................uuuaggccaGggaaguuugagg...	1	1	seq
.................uuuagUccauggaaguuugagg...	2	1	seq
.................uuCaggccauggaaguuugagg...	1	1	seq
.................uuuaggccauggaaguuugagA...	15	1	seq
.................uuuaggccauggaagCuugagg...	1	1	seq
.................uuuaggccauggGaguuugagg...	1	1	seq
.................uuuaggccauggaaguuugagg...	179	0	seq
.................Guuaggccauggaaguuugagg...	1	1	seq
.................uuuaggcGauggaaguuugagg...	1	1	seq
.................uuuaggccaCggaaguuugagg...	1	1	seq
.................uuuaggccauggaaguuugagU...	3	1	seq
.................uuuaggccauggaaguuugUgg...	1	1	seq
.................uuuaggccauggaaguuugagC...	1	1	seq
.................uuuaggccGuggaaguuugaggc..	1	1	seq
.................uuuaggccauggaaguuugagCc..	2	1	seq
.................uuuGggccauggaaguuugaggc..	1	1	seq
.................Guuaggccauggaaguuugaggc..	1	1	seq
.................uuuaggccauggaaguuCgaggc..	1	1	seq
.................Cuuaggccauggaaguuugaggc..	3	1	seq
.................uuuaggccauggaaguuugGggc..	3	1	seq
.................uuuaggccauggaaguuugaggG..	3	1	seq
.................uuuaggccauggaaguuugUggc..	1	1	seq
.................uuuaggccauggaGguuugaggc..	2	1	seq
.................uuuaggccauggaaguuugCggc..	1	1	seq
.................uuuaggccauggaaguuugaggc..	314	0	seq
.................uuuaggccauggaagCuugaggc..	2	1	seq
.................uCuaggccauggaaguuugaggc..	1	1	seq
.................uuuaggccauggaaguuugagAca...	2	1	seq
.................Auuaggccauggaaguuugaggca...	1	1	seq
.................uuuaggccauggaagCuugaggca...	3	1	seq
.................uuuaggccUuggaaguuugaggca...	1	1	seq
.................uuuaggccauggaaguuugaggca...	1106	0	seq
.................uuuaggccauggGaguuugaggca...	3	1	seq
.................Nuuaggccauggaaguuugaggca...	1	1	seq
.................uuuGggccauggaaguuugaggca...	1	1	seq
.................uuuaggcUauggaaguuugaggca...	1	1	seq
.................uuuaggccauggaaguuugaCgca...	1	1	seq
.................uuuaggccauggaaguCugaggca...	5	1	seq
.................uuuaggccauggaaguuugaUgca...	1	1	seq
.................uuuaggccauggaGguuugaggca...	5	1	seq
.................Cuuaggccauggaaguuugaggca...	1	1	seq
.................uuuaggccauggaaguuugaggcG...	62	1	seq
.................uuuaggccauggaaguuCgaggca...	1	1	seq
.................uuGaggccauggaaguuugaggca...	1	1	seq

```
                    Mature      Star
uaaaaagacuauggucauuuaggccauggaaguuugaggcaauggcaaauaucuaaugugcuuaaauguucaaaauugcacaagcacuacacuaaugcagugaacgaguuuuuc
.................uuuaggccauggaaguuugagCca.........................................................................    4    1    seq
.................uCuaggccauggaaguuugaggca.........................................................................    3    1    seq
.................uuuagUccauggaaguuugaggca.........................................................................    1    1    seq
.................uuuaggccGuggaaguuugaggca.........................................................................    6    1    seq
.................uuCaggccauggaaguuugaggca.........................................................................    2    1    seq
.................uuuaggccauggUaguuugaggca.........................................................................    2    1    seq
.................uuuaggccauggaaguuugUggca.........................................................................    2    1    seq
.................uuuaggccauggaaguuugGggca.........................................................................    8    1    seq
.................uuuaggccaCggaaguuugaggca.........................................................................    5    1    seq
.................uuuaggccauggaaguuugaAgca.........................................................................    1    1    seq
.................uuuaggccauggaaguuuAaggca.........................................................................    1    1    seq
.................uuuaggccauggaaguuugaggcC.........................................................................    1    1    seq
.................uuuaggccauggaaguuugaggcU.........................................................................    16   1    seq
.................uuGaggccauggaaguuugaggcaa........................................................................    1    1    seq
.................uuuaggccauggaaguuugGggcaa........................................................................    1    1    seq
.................uuuGggccauggaaguuugaggcaa........................................................................    2    1    seq
.................uuuaggccauggaaguuugaggcaa........................................................................    129  0    seq
.................uuuaggccGuggaaguuugaggcaa........................................................................    1    1    seq
.................uuuagUccauggaaguuugaggcaa........................................................................    1    1    seq
.................uuuaggccauggaaguuugaggcaU........................................................................    3    1    seq
.................uuuaggccauggaaguCugaggcaa........................................................................    1    1    seq
.................uuuaggccauggaaguuugaggcGa........................................................................    1    1    seq
.................uuuaggccauggaaguuugaggcaG........................................................................    6    1    seq
.................uuuaggccauggaaguuugaggcaGu.......................................................................    1    1    seq
.................uuuaggccauggGaguuugaggcaau.......................................................................    2    1    seq
.................uuuaggccGuggaaguuugaggcaau.......................................................................    1    1    seq
.................Cuuaggccauggaaguuugaggcaau.......................................................................    1    1    seq
.................uuGaggccauggaaguuugaggcaau.......................................................................    1    1    seq
.................uuuaggccauggaaguuugaggcaaC.......................................................................    18   1    seq
.................uuuaggccauggaaguuuAaggcaau.......................................................................    1    1    seq
.................uuuaggccauggaaguuugaggcaau.......................................................................    187  0    seq
.................Nuuaggccauggaaguuugaggcaau.......................................................................    1    1    seq
.................uuuGggccauggaaguuugaggcaau.......................................................................    1    1    seq
.................uuuaggccauggaaguuCgaggcaau.......................................................................    2    1    seq
.................uuuaggccauggaaguuugaggcaaug......................................................................    1    0    seq
.................uuuaggccauggaaguuugGggcaaug......................................................................    1    1    seq
.................uuuaggccauggaaguuugaggcaauAgc....................................................................    1    1    seq
.................uuuaggccauggaaguuugaggcaauAgca...................................................................    1    1    seq
..................uuaggccauggaaguuG...............................................................................    2    1    seq
..................uuaggccauggUaguuu...............................................................................    1    1    seq
..................uuaggccaAggaaguuu...............................................................................    1    1    seq
..................uCaggccauggaaguuu...............................................................................    1    1    seq
..................uuaggccauggaaguuu...............................................................................    285  0    seq
..................uuaggccauggaGguuu...............................................................................    2    1    seq
..................uuaggccauggaaguuC...............................................................................    29   1    seq
..................uuaggccauggaaguCu...............................................................................    1    1    seq
..................uuaggccUuggaaguuu...............................................................................    1    1    seq
..................uuaggccauggGaguuu...............................................................................    1    1    seq
..................uuaggccauggaaguuAg..............................................................................    1    1    seq
..................uuaggccauggaaguuuU..............................................................................    3    1    seq
..................uuaggccauggaagCuug..............................................................................    2    1    seq
..................uCaggccauggaaguuug..............................................................................    1    1    seq
..................Nuaggccauggaaguuug..............................................................................    2    1    seq
..................uuaggccauggaGguuug..............................................................................    7    1    seq
..................uuaggccauggaaguCug..............................................................................    4    1    seq
..................uuaggccGuggaaguuug..............................................................................    5    1    seq
..................uuaggccauggGaguuug..............................................................................    4    1    seq
..................uuaggccaCggaaguuug..............................................................................    3    1    seq
..................uuaggccaugAaaguuug..............................................................................    1    1    seq
..................uuaggccauggaaguGug..............................................................................    1    1    seq
..................uuaggccauggaaguuuA..............................................................................    21   1    seq
..................uuaggccaAggaaguuug..............................................................................    1    1    seq
..................uuaggccauCgaaguuug..............................................................................    1    1    seq
..................uuaggcUauggaaguuug..............................................................................    2    1    seq
..................uuCggccauggaaguuug..............................................................................    1    1    seq
..................uuGggccauggaaguuug..............................................................................    5    1    seq
..................uGaggccauggaaguuug..............................................................................    1    1    seq
..................uuaggccauggaaguuuC..............................................................................    1    1    seq
..................uuagUccauggaaguuug..............................................................................    1    1    seq
..................uuaggccauggaaguuCg..............................................................................    3    1    seq
```

Mature **Star**

```
uaaaaagacuauggucauuuaggccauggaaguuugaggcaauggcaaauaucuaaugugcuuaaauguucaaaauugcacaagcacuacacuaaugcagugaacgaguuuuuc
..................uuaggccauUgaaguuug..............................................................................    2      1     seq
..................uuaggccauggaaguuug..............................................................................    1344   0     seq
..................uuaggccauggaaguuuAa.............................................................................    1      1     seq
..................uuaggccauggaaguuugG.............................................................................    24     1     seq
..................uuaggccauggaaguuGga.............................................................................    1      1     seq
..................uuaggccauggaUguuuga.............................................................................    1      1     seq
..................uuaggccauggaaguuuga.............................................................................    1104   0     seq
..................Auaggccauggaaguuuga.............................................................................    2      1     seq
..................Nuaggccauggaaguuuga.............................................................................    2      1     seq
..................uuaggccaGggaaguuuga.............................................................................    1      1     seq
..................uuaggccauggaaguuugU.............................................................................    11     1     seq
..................uuaggccauggaGguuuga.............................................................................    2      1     seq
..................uuaggccauggaaguCuga.............................................................................    2      1     seq
..................uuUggccauggaaguuuga.............................................................................    1      1     seq
..................uuaggccauggaagGuuga.............................................................................    2      1     seq
..................uuaggccauggaaguuuCa.............................................................................    2      1     seq
..................uuaggccauggaagCuuga.............................................................................    3      1     seq
..................Cuaggccauggaaguuuga.............................................................................    2      1     seq
..................uuaggccaCggaaguuuga.............................................................................    1      1     seq
..................Guaggccauggaaguuuga.............................................................................    1      1     seq
..................uuGggccauggaaguuuga.............................................................................    5      1     seq
..................uuaggccGuggaaguuuga.............................................................................    3      1     seq
..................uuaggccauggaaguuCga.............................................................................    1      1     seq
..................uuaggccauggGaguuuga.............................................................................    7      1     seq
..................uuaggccaAggaaguuuga.............................................................................    2      1     seq
..................uuCggccauggaaguuuga.............................................................................    1      1     seq
..................uuGggccauggaaguuugag............................................................................    3      1     seq
..................uuaggccauggaagCuugag............................................................................    1      1     seq
..................uuaggccauggaGguuugag............................................................................    1      1     seq
..................Nuaggccauggaaguuugag............................................................................    2      1     seq
..................uuaggccauggaaguuugag............................................................................    308    0     seq
..................uuaggccaCggaaguuugag............................................................................    1      1     seq
..................Auaggccauggaaguuugag............................................................................    1      1     seq
..................uuaggccauggaaguuCgag............................................................................    3      1     seq
..................uuagUccauggaaguuugag............................................................................    1      1     seq
..................uuaggccauggaaguuugGg............................................................................    3      1     seq
..................uuaggccauggaaguuugagA...........................................................................    55     1     seq
..................uuaggccaAggaaguuugagg...........................................................................    1      1     seq
..................Cuaggccauggaaguuugagg...........................................................................    3      1     seq
..................uuaggccauggaagCuugagg...........................................................................    1      1     seq
..................uuaggccauggaaguuugaCg...........................................................................    2      1     seq
..................uuagUccauggaaguuugagg...........................................................................    1      1     seq
..................uuaggccauggGaguuugagg...........................................................................    3      1     seq
..................uuaggccauggaaguuCgagg...........................................................................    3      1     seq
..................uuaggccauggaagGuugagg...........................................................................    2      1     seq
..................Guaggccauggaaguuugagg...........................................................................    3      1     seq
..................uuGggccauggaaguuugagg...........................................................................    2      1     seq
..................uuaggccauggaaguuUagg............................................................................    1      1     seq
..................uuaggccauggaaguuugagU...........................................................................    8      1     seq
..................uuaggccauggaaguuugagg...........................................................................    955    0     seq
..................uuaggccauggaaguuugaUg...........................................................................    1      1     seq
..................uuaggccaCggaaguuugagg...........................................................................    2      1     seq
..................uuaggccGuggaaguuugagg...........................................................................    5      1     seq
..................uCaggccauggaaguuugagg...........................................................................    1      1     seq
..................uuaggccauggaaguuugGgg...........................................................................    9      1     seq
..................uuaggccauggCaguuugagg...........................................................................    1      1     seq
..................uuaggccauggaaguCugagg...........................................................................    2      1     seq
..................uuaggccauUgaaguuugagg...........................................................................    1      1     seq
..................uuaggccauggaaguuugagC...........................................................................    3      1     seq
..................uuaggccauggaGguuugagg...........................................................................    1      1     seq
..................uuaggccauggaaguuugUgg...........................................................................    2      1     seq
..................uuaggccauggaUguuugaggc..........................................................................    1      1     seq
..................uuaggccauggaagGuugaggc..........................................................................    2      1     seq
..................uuaggccauggaaguCugaggc..........................................................................    1      1     seq
..................Nuaggccauggaaguuugaggc..........................................................................    2      1     seq
..................uuGggccauggaaguuugaggc..........................................................................    6      1     seq
..................uuaggccauggaaguuugaggc..........................................................................    1103   0     seq
..................Guaggccauggaaguuugaggc..........................................................................    2      1     seq
..................uuaggccauggaagCuugaggc..........................................................................    2      1     seq
..................uuaggccauggaaguAugaggc..........................................................................    1      1     seq
```

Mature **Star**

```
uaaaaagacuauggucauuuaggccauggaaguuugaggcaauggcaaauaucuaaugugcuuaaauguucaaaauugcacaagcacuacacuaaugcagugaacgaguuuuuc
..................uuaggccauggaaguuugUggc..........................................................................  1     1  seq
..................uuUggccauggaaguuugaggc..........................................................................  1     1  seq
..................uuaggccauggaaguuGgaggc..........................................................................  2     1  seq
..................uuaggccauggaGguuugaggc..........................................................................  3     1  seq
..................uuaggccauggaaguuugGggc..........................................................................  4     1  seq
..................uuaggccCuggaaguuugaggc..........................................................................  1     1  seq
..................uuaggccauggUaguuugaggc..........................................................................  1     1  seq
..................uuaggccauggaaguuugaggG..........................................................................  8     1  seq
..................uuaggccauggGaguuugaggc..........................................................................  6     1  seq
..................uuaggccGuggaaguuugaggc..........................................................................  2     1  seq
..................uuaggccauggaaguuugagCc..........................................................................  4     1  seq
..................uuaggccUuggaaguuugaggc..........................................................................  1     1  seq
..................Cuaggccauggaaguuugaggc..........................................................................  7     1  seq
..................uuaggccauggaaguuugaUgc..........................................................................  1     1  seq
..................uCaggccauggaaguuugaggc..........................................................................  1     1  seq
..................uuaggccauggaaguuugagAc..........................................................................  2     1  seq
..................uuaggccaCggaaguuugaggc..........................................................................  2     1  seq
..................uuaggccauggaaguuugaAgc..........................................................................  1     1  seq
..................uuaggccauggaagAuugaggc..........................................................................  1     1  seq
..................uuaggccaCggaaguuugaggca.........................................................................  8     1  seq
..................uuaggccauggaaguCugaggca.........................................................................  8     1  seq
..................Cuaggccauggaaguuugaggca.........................................................................  12    1  seq
..................uuaggccauggaagAuugaggca.........................................................................  1     1  seq
..................uuaggccauggUaguuugaggca.........................................................................  1     1  seq
..................uuaggccauggaaguGugaggca.........................................................................  2     1  seq
..................uuaggccGuggaaguuugaggca.........................................................................  8     1  seq
..................uuaggccauggaaguuugUggca.........................................................................  4     1  seq
..................uuaggccauggGaguuugaggca.........................................................................  14    1  seq
..................uuCggccauggaaguuugaggca.........................................................................  1     1  seq
..................uuaggccauUgaaguuugaggca.........................................................................  2     1  seq
..................uuUggccauggaaguuugaggca.........................................................................  1     1  seq
..................uuaggccaAggaaguuugaggca.........................................................................  3     1  seq
..................uuagCccauggaaguuugaggca.........................................................................  1     1  seq
..................uuaggccauggaGguuugaggca.........................................................................  11    1  seq
..................uuaggccauggaaguuugaggGa.........................................................................  1     1  seq
..................uuaggccauggaaguuugagUca.........................................................................  3     1  seq
..................uuaggccauggaaguuugGggca.........................................................................  25    1  seq
..................uuaggccauggaagCuugaggca.........................................................................  15    1  seq
..................uuaggccaGggaaguuugaggca.........................................................................  2     1  seq
..................uuaggccauggaaguuugaUgca.........................................................................  2     1  seq
..................uuaggccauggaaguuugaAgca.........................................................................  3     1  seq
..................Nuaggccauggaaguuugaggca.........................................................................  9     1  seq
..................uuGggccauggaaguuugaggca.........................................................................  19    1  seq
..................uuaggccauggaaguuuUaggca.........................................................................  1     1  seq
..................uuaggccauggaaguuuAaggca.........................................................................  1     1  seq
..................uuaggccauggaaguuugaggcU.........................................................................  43    1  seq
..................Auaggccauggaaguuugaggca.........................................................................  4     1  seq
..................uuaggccauggaagGuugaggca.........................................................................  4     1  seq
..................uuaggccUuggaaguuugaggca.........................................................................  4     1  seq
..................Guaggccauggaaguuugaggca.........................................................................  2     1  seq
..................uuaggccauggaaguuCgaggca.........................................................................  6     1  seq
..................uuaggccauggaaguAugaggca.........................................................................  2     1  seq
..................uuaggccauggaaguuugaggca.........................................................................  4630  0  seq
..................uuaggcUauggaaguuugaggca.........................................................................  7     1  seq
..................uuaggccauggaaguuAgaggca.........................................................................  1     1  seq
..................uuaggccauggaaCuuugaggca.........................................................................  1     1  seq
..................uuaggccauggaaguuugaggcG.........................................................................  210   1  seq
..................uuaggccauggaaguuugagCca.........................................................................  14    1  seq
..................uuaggccauggaaguuugagAca.........................................................................  7     1  seq
..................uuaggccaugUaaguuugaggca.........................................................................  1     1  seq
..................uuaggccaugAaaguuugaggca.........................................................................  4     1  seq
..................uuaggccauggaaguuugaggcC.........................................................................  18    1  seq
..................uuaggccauggaUguuugaggca.........................................................................  2     1  seq
..................uuaggccCuggaaguuugaggca.........................................................................  1     1  seq
..................uCaggccauggaaguuugaggca.........................................................................  5     1  seq
..................uuaggccauggaaguuugaggcGa........................................................................  3     1  seq
..................uuaggccauggGaguuugaggcaa........................................................................  2     1  seq
..................uuaggccauggaaguuugaggcaa........................................................................  552   0  seq
..................uuaggccauggaaguuCgaggcaa........................................................................  2     1  seq
..................uuaggccauggUaguuugaggcaa........................................................................  1     1  seq
```

Mature Star

```
uaaaaagacuauggucauuuaggccauggaaguuugaggcaauggcaaauaucuaaugugcuuaaauguucaaaauugcacaagcacuacacuaaugcagugaacgaguuuuuc
..................uuaggccauggaGguuugaggcaa........................................................................ 5 1 seq
..................uuaggccaCggaaguuugaggcaa........................................................................ 2 1 seq
..................uuaggccauggaaguuugaggcaG........................................................................ 6 1 seq
..................uuaggccauggaaguuugaggcaC........................................................................ 1 1 seq
..................uuaggccauggaaguuugaggcaU........................................................................ 4 1 seq
..................uuaggccauggaaguuugaggGaa........................................................................ 1 1 seq
..................uuaggccauggaUguuugaggcaa........................................................................ 1 1 seq
..................uuaggccauggaaguuugagAcaa........................................................................ 1 1 seq
..................uuaggccGuggaaguuugaggcaa........................................................................ 1 1 seq
..................uuaggccauggaaguCugaggcaa........................................................................ 1 1 seq
..................Cuaggccauggaaguuugaggcaa........................................................................ 3 1 seq
..................uuaggccauggaagAuugaggcaa........................................................................ 1 1 seq
..................uuGggccauggaaguuugaggcaa........................................................................ 1 1 seq
..................Auaggccauggaaguuugaggcaa........................................................................ 1 1 seq
..................uuaggccauggaagCuugaggcaa........................................................................ 1 1 seq
..................Guaggccauggaaguuugaggcaa........................................................................ 1 1 seq
..................uuaggccauggaaguuuAaggcaa........................................................................ 1 1 seq
..................uuaggccauggaaguuugaggcUa........................................................................ 1 1 seq
..................uuaggcUauggaaguuugaggcaa........................................................................ 1 1 seq
..................uuaggccauggaaguuugGggcaa........................................................................ 2 1 seq
..................uuaggGcauggaaguuugaggcaa........................................................................ 1 1 seq
..................uuaggccauggaaguuugaggcaaG....................................................................... 5 1 seq
..................uuaggccauggaaguuugaggcaGu....................................................................... 2 1 seq
..................uuGggccauggaaguuugaggcaau....................................................................... 2 1 seq
..................uuaggccauggaaguuugaggcGau....................................................................... 1 1 seq
..................uuaggccauggaaguuugaggcaaC....................................................................... 78 1 seq
..................Cuaggccauggaaguuugaggcaau....................................................................... 1 1 seq
..................uuaggccauggaaguuugaggcaau....................................................................... 630 0 seq
..................uuaggccauggaaguuCgaggcaau....................................................................... 1 1 seq
..................uuaggccaCggaaguuugaggcaau....................................................................... 1 1 seq
..................Guaggccauggaaguuugaggcaau....................................................................... 3 1 seq
..................uuaggccauggaaguuugagAcaau....................................................................... 1 1 seq
..................Nuaggccauggaaguuugaggcaau....................................................................... 1 1 seq
..................uuaggccauggaagAuugaggcaau....................................................................... 1 1 seq
..................uuaggccauggGaguuugaggcaau....................................................................... 1 1 seq
..................uuaggccauggaaguuugaggcaaA....................................................................... 4 1 seq
..................uuaggccauggUaguuugaggcaau....................................................................... 1 1 seq
..................uuaggccauggaUguuugaggcaau....................................................................... 1 1 seq
..................uuaggccauggaaguuugGggcaau....................................................................... 1 1 seq
..................uuaggccauggaGguuugaggcaau....................................................................... 4 1 seq
..................uuaggccaugAaaguuugaggcaau....................................................................... 1 1 seq
..................uuaggccGuggaaguuugaggcaau....................................................................... 4 1 seq
..................uuUggccauggaaguuugaggcaau....................................................................... 1 1 seq
..................uuaggcUauggaaguuugaggcaau....................................................................... 2 1 seq
..................uuaggccauggaagCuugaggcaau....................................................................... 1 1 seq
..................uuaggccauggaaguuugaggcaauU...................................................................... 5 1 seq
..................uuaggccauggGaguuugaggcaaug...................................................................... 1 1 seq
..................uuaggccauggaaguuugaggcaaug...................................................................... 6 0 seq
..................uuaggccauggaaguuugaggcaauAg..................................................................... 1 1 seq
..................uuaggccauggaaguuugaggcaaugAc.................................................................... 1 1 seq
..................uuaggccauggaaguuugaggcaaugAca................................................................... 1 1 seq
..................uuaggccauggaaguuugaggcaauAgca................................................................... 1 1 seq
...................uaggccauggaaguuAg.............................................................................. 3 1 seq
...................uaggccauggaaguGug.............................................................................. 3 1 seq
...................uaggccauggaGguuug.............................................................................. 10 1 seq
...................uaggccauggaagAuug.............................................................................. 3 1 seq
...................uaggccauggaaguuuA.............................................................................. 64 1 seq
...................uaggccauggaaguuGg.............................................................................. 3 1 seq
...................uaggccauggaagGuug.............................................................................. 1 1 seq
...................uaggccauggaUguuug.............................................................................. 1 1 seq
...................uaggccaCggaaguuug.............................................................................. 7 1 seq
...................uaggGcauggaaguuug.............................................................................. 1 1 seq
...................uUggccauggaaguuug.............................................................................. 4 1 seq
...................uaggccauggaaguAug.............................................................................. 2 1 seq
...................uaggccauggaaguCug.............................................................................. 8 1 seq
...................uaggccauggaaguuuC.............................................................................. 3 1 seq
...................uaggccauggaaguuug.............................................................................. 3268 0 seq
...................uaggccauggaaguuuU.............................................................................. 2 1 seq
...................uaggccauggaaguuCg.............................................................................. 13 1 seq
...................uGggccauggaaguuug.............................................................................. 8 1 seq
```

Mature Star

```
uaaaaagacuauggucauuuaggccauggaaguuugaggcaauggcaaauaucuaaugugcuuaaauguucaaaauugcacaagcacuacacuaaugcagugaacgaguuuuuc
...................uaggccauggUaguuug..............................................................................	1	1	seq
...................Naggccauggaaguuug..............................................................................	5	1	seq
...................uaggcUauggaaguuug..............................................................................	5	1	seq
...................uaggccauggaagCuug..............................................................................	6	1	seq
...................uCggccauggaaguuug..............................................................................	1	1	seq
...................uaggccaAggaaguuug..............................................................................	1	1	seq
...................uaggccaAggaaguuuga.............................................................................	1	1	seq
...................uaggccauggaGguuuga.............................................................................	3	1	seq
...................uaggcUauggaaguuuga.............................................................................	1	1	seq
...................uaggccauggaaguuAga.............................................................................	1	1	seq
...................uaggccauggaaguCuga.............................................................................	1	1	seq
...................uaggccauggaagCuuga.............................................................................	6	1	seq
...................uaggccauggaaguuGga.............................................................................	1	1	seq
...................uaggccaCggaaguuuga.............................................................................	3	1	seq
...................uGggccauggaaguuuga.............................................................................	2	1	seq
...................uagUccauggaaguuuga.............................................................................	3	1	seq
...................uUggccauggaaguuuga.............................................................................	2	1	seq
...................uaggccauggaaguuuga.............................................................................	1613	0	seq
...................uaggccauggaUguuuga.............................................................................	1	1	seq
...................uaggccauggaaguuCga.............................................................................	4	1	seq
...................uaggccauggCaguuuga.............................................................................	2	1	seq
...................uaggccauggaagGuugag............................................................................	1	1	seq
...................uaggcUauggaaguuugag............................................................................	1	1	seq
...................uaggccauggaaguuugag............................................................................	574	0	seq
...................uUggccauggaaguuugag............................................................................	1	1	seq
...................uaggccauggaaguuCgag............................................................................	2	1	seq
...................uaggccauggaaguuugUg............................................................................	2	1	seq
...................uaggccauggaaguuugaC............................................................................	1	1	seq
...................uaggccaCggaaguuugag............................................................................	2	1	seq
...................uaggccauggaaguuuCag............................................................................	2	1	seq
...................uaggccauggaaguAugag............................................................................	1	1	seq
...................uaggccauggaagCuugag............................................................................	1	1	seq
...................uaggccauggaaguCugag............................................................................	3	1	seq
...................uGggccauggaaguuugag............................................................................	2	1	seq
...................uaggccauggaGguuugag............................................................................	1	1	seq
...................uaggccauggGaguuugag............................................................................	5	1	seq
...................uaggccaugUaaguuugag............................................................................	1	1	seq
...................uaggccauUgaaguuugagg...........................................................................	1	1	seq
...................Naggccauggaaguuugagg...........................................................................	3	1	seq
...................uaggccauggaaguGugagg...........................................................................	1	1	seq
...................uaggccauggaaguuugagg...........................................................................	2045	0	seq
...................uaggccaAggaaguuugagg...........................................................................	2	1	seq
...................uaggcUauggaaguuugagg...........................................................................	1	1	seq
...................uGggccauggaaguuugagg...........................................................................	5	1	seq
...................uaggccaCggaaguuugagg...........................................................................	4	1	seq
...................uaggccauggaagCuugagg...........................................................................	5	1	seq
...................uCggccauggaaguuugagg...........................................................................	1	1	seq
...................uaggccauggaaguuugagU...........................................................................	18	1	seq
...................uaggccauggGaguuugagg...........................................................................	4	1	seq
...................uaggccauggaaguuugaUg...........................................................................	1	1	seq
...................uagUccauggaaguuugagg...........................................................................	2	1	seq
...................uaggccauggaaguuCgagg...........................................................................	8	1	seq
...................uaggccauggaaguCugagg...........................................................................	2	1	seq
...................uaggccauggaGguuugagg...........................................................................	8	1	seq
...................uaggccauggaaguuuAagg...........................................................................	1	1	seq
...................uaggccauggaaguuugaCg...........................................................................	1	1	seq
...................uaggccaugUaaguuugagg...........................................................................	2	1	seq
...................uaggccauggUaguuugagg...........................................................................	2	1	seq
...................uaggccauggaaguuugagC...........................................................................	7	1	seq
...................uaggccauggaCguuugagg...........................................................................	1	1	seq
...................uaggccauggaaguuugagA...........................................................................	137	1	seq
...................uaggccauggaaguuuGUgg...........................................................................	2	1	seq
...................uaggccauggaagAuugagg...........................................................................	1	1	seq
...................uaggccUuggaaguuugaggc..........................................................................	2	1	seq
...................uGggccauggaaguuugaggc..........................................................................	11	1	seq
...................uaggccauggaagCuugaggc..........................................................................	7	1	seq
...................uaggccauggaaguuugaggG..........................................................................	10	1	seq
...................uaggccauggaaguuugaggc..........................................................................	4862	0	seq
...................uaggccauggaaguuGgaggc..........................................................................	1	1	seq
...................uaggccaugUaaguuugaggc..........................................................................	1	1	seq
```

```
                   Mature     Star
uaaaaagacuauggucauuuaggccauggaaguuugaggcaauggcaaauaucuaaugugcuuaaauguucaaaauugcacaagcacuacacuaaugcagugaacgaguuuuuc
...................uaggccauggaaguuCgaggc..........................................................................  10     1  seq
...................uaggccauggaUguuugaggc..........................................................................  3      1  seq
...................uaggccaCggaaguuugaggc..........................................................................  9      1  seq
...................uaggccauggaaguAugaggc..........................................................................  1      1  seq
...................uagUccauggaaguuugaggc..........................................................................  2      1  seq
...................Naggccauggaaguuugaggc..........................................................................  6      1  seq
...................uaggccauggaagGuugaggc..........................................................................  3      1  seq
...................uaggcGauggaaguuugaggc..........................................................................  2      1  seq
...................uaggccauggaaguuugaUgc..........................................................................  1      1  seq
...................uaggccauggaaguuuAaggc..........................................................................  2      1  seq
...................uaggccauggaaguCugaggc..........................................................................  7      1  seq
...................uaggccauggaagAuugaggc..........................................................................  1      1  seq
...................uaggccauggaGguuugaggc..........................................................................  15     1  seq
...................uaggcUauggaaguuugaggc..........................................................................  8      1  seq
...................uaCgccauggaaguuugaggc..........................................................................  2      1  seq
...................uaggccauggaaguuugagCc..........................................................................  22     1  seq
...................uaggccCuggaaguuugaggc..........................................................................  1      1  seq
...................Caggccauggaaguuugaggc..........................................................................  16     1  seq
...................uUggccauggaaguuugaggc..........................................................................  1      1  seq
...................uaggccauggaaguuugagAc..........................................................................  17     1  seq
...................uaggccGuggaaguuugaggc..........................................................................  12     1  seq
...................uaggccauggaaguuugaAgc..........................................................................  3      1  seq
...................uaggccauggGaguuugaggc..........................................................................  13     1  seq
...................uaggccauggaaguuuUaggc..........................................................................  1      1  seq
...................uaggccaugAaaguuugaggc..........................................................................  2      1  seq
...................uaggccauggaaguuAgaggc..........................................................................  4      1  seq
...................uaggccauggUaguuugaggc..........................................................................  2      1  seq
...................uaggccauggaaguuugagUc..........................................................................  2      1  seq
...................uaggccauggaaguuugUggc..........................................................................  2      1  seq
...................uaggccauggaaguGugaggc..........................................................................  1      1  seq
...................uaggccauggaaguuugaCgc..........................................................................  3      1  seq
...................uaggccauggaaguuugaggcG.........................................................................  681    1  seq
...................uaggccauggaaguuugaggca.........................................................................  14443  0  seq
...................uaggccauggaGguuugaggca.........................................................................  46     1  seq
...................Gaggccauggaaguuugaggca.........................................................................  27     1  seq
...................uaggGcauggaaguuugaggca.........................................................................  3      1  seq
...................uaggccauggaaguuugaAgca.........................................................................  8      1  seq
...................uaggccauggaaguuugaggGa.........................................................................  10     1  seq
...................uaggccauggaaguAugaggca.........................................................................  2      1  seq
...................uaggccauggaaguuuAaggca.........................................................................  1      1  seq
...................uaggccauggaaguuugagCca.........................................................................  38     1  seq
...................uaggccauggaagGuugaggca.........................................................................  7      1  seq
...................uaggccauggaaguuugagAca.........................................................................  33     1  seq
...................uaggccauggaaguuugUggca.........................................................................  12     1  seq
...................uUggccauggaaguuugaggca.........................................................................  8      1  seq
...................uaggcGauggaaguuugaggca.........................................................................  2      1  seq
...................uCggccauggaaguuugaggca.........................................................................  4      1  seq
...................uaggccauggaaguuugaggcU.........................................................................  142    1  seq
...................uaggccauggaCguuugaggca.........................................................................  3      1  seq
...................Caggccauggaaguuugaggca.........................................................................  32     1  seq
...................uaggccUuggaaguuugaggca.........................................................................  5      1  seq
...................uaggccauggaaguuAgaggca.........................................................................  2      1  seq
...................uaggccauggaUguuugaggca.........................................................................  3      1  seq
...................uaggccaCggaaguuugaggca.........................................................................  21     1  seq
...................uaggccCuggaaguuugaggca.........................................................................  1      1  seq
...................uaggccauggGaguuugaggca.........................................................................  46     1  seq
...................uagCccauggaaguuugaggca.........................................................................  1      1  seq
...................uaggccauggaaCuuugaggca.........................................................................  1      1  seq
...................uaggccaAggaaguuugaggca.........................................................................  3      1  seq
...................uaggccauggaaguuugaUgca.........................................................................  4      1  seq
...................uaggccauggCaguuugaggca.........................................................................  2      1  seq
...................uaggccauCgaaguuugaggca.........................................................................  1      1  seq
...................uaggccauggaaguGugaggca.........................................................................  2      1  seq
...................uaggccauggaaguuugagUca.........................................................................  1      1  seq
...................uaggccaugAaaguuugaggca.........................................................................  5      1  seq
...................uaCgccauggaaguuugaggca.........................................................................  1      1  seq
...................uagUccauggaaguuugaggca.........................................................................  4      1  seq
...................uaggccauggaaguuGgaggca.........................................................................  4      1  seq
...................uaggccaugUaaguuugaggca.........................................................................  3      1  seq
...................uaggccauggaaguuCgaggca.........................................................................  22     1  seq
```

```
                   Mature     Star
uaaaaagacuauggucauuuaggccauggaaguuugaggcaauggcaaauaucuaaugugcuuaaauguucaaaauugcacaagcacuacacuaaugcagugaacgaguuuuuc
...................uaggccauUgaaguuugaggca.........................................................................    1      1    seq
...................uaggccauggaaguuugaCgca.........................................................................    5      1    seq
...................uaggccauggaaguCugaggca.........................................................................    13     1    seq
...................uaggccauggaaguuuUaggca.........................................................................    2      1    seq
...................uaggccauggaagAuugaggca.........................................................................    3      1    seq
...................uaggcUauggaaguuugaggca.........................................................................    11     1    seq
...................uaggccauggaagCuugaggca.........................................................................    41     1    seq
...................uGggccauggaaguuugaggca.........................................................................    27     1    seq
...................uaggccauggUaguuugaggca.........................................................................    7      1    seq
...................Naggccauggaaguuugaggca.........................................................................    17     1    seq
...................uaggccauggaaguuugaggcC.........................................................................    39     1    seq
...................uaggccGuggaaguuugaggca.........................................................................    41     1    seq
...................uaggccauggaaguuCgaggcaa........................................................................    2      1    seq
...................uaggccauggaaguuugagCcaa........................................................................    1      1    seq
...................Naggccauggaaguuugaggcaa........................................................................    4      1    seq
...................uaggccauggaaguGugaggcaa........................................................................    1      1    seq
...................uaggccauggCaguuugaggcaa........................................................................    1      1    seq
...................uagCccauggaaguuugaggcaa........................................................................    1      1    seq
...................uaggccauggaaguuugaAgcaa........................................................................    2      1    seq
...................uaggcGauggaaguuugaggcaa........................................................................    1      1    seq
...................Gaggccauggaaguuugaggcaa........................................................................    1      1    seq
...................uaggccauggaaguCugaggcaa........................................................................    2      1    seq
...................uaggccauggaaguuugaggcaC........................................................................    3      1    seq
...................uaggccauggaaguuugUggcaa........................................................................    3      1    seq
...................uaggccauggaaguuugaggcaU........................................................................    17     1    seq
...................Caggccauggaaguuugaggcaa........................................................................    3      1    seq
...................uaggccauggaGguuugaggcaa........................................................................    5      1    seq
...................uaggccauggaaguuugaggcaa........................................................................    1482   0    seq
...................uaggccaCggaaguuugaggcaa........................................................................    4      1    seq
...................uagUccauggaaguuugaggcaa........................................................................    1      1    seq
...................uaggcUauggaaguuugaggcaa........................................................................    1      1    seq
...................uaggccauggaaguuGgaggcaa........................................................................    1      1    seq
...................uaggccauggGaguuugaggcaa........................................................................    9      1    seq
...................uaggccauggaagGuugaggcaa........................................................................    2      1    seq
...................uaggccauggaaguuugaggcUa........................................................................    1      1    seq
...................uaggccGuggaaguuugaggcaa........................................................................    3      1    seq
...................uaggccauggaaguuuUaggcaa........................................................................    1      1    seq
...................uaggccauggaaguuugaggcCa........................................................................    2      1    seq
...................uGggccauggaaguuugaggcaa........................................................................    3      1    seq
...................uaggccauggaagCuugaggcaa........................................................................    3      1    seq
...................uaggccauggaaguuugaggcaG........................................................................    48     1    seq
...................uaggccauggaaguuugagUcaa........................................................................    1      1    seq
...................uaggccauggaaguuugaggcGa........................................................................    7      1    seq
...................uaggccauggaaguuugaggcaCu.......................................................................    1      1    seq
...................uGggccauggaaguuugaggcaau.......................................................................    4      1    seq
...................uaggccauggaGguuugaggcaau.......................................................................    4      1    seq
...................uaggccauggaaguuugaggcaaC.......................................................................    177    1    seq
...................uaggccauggaagGuugaggcaau.......................................................................    1      1    seq
...................uaggccauggaaguuugagAcaau.......................................................................    1      1    seq
...................uaggccauggaaguuCgaggcaau.......................................................................    5      1    seq
...................uaggccGuggaaguuugaggcaau.......................................................................    7      1    seq
...................uaggccauggaaguuugagUcaau.......................................................................    1      1    seq
...................uaggccauggaaguuugaggcaUu.......................................................................    1      1    seq
...................uaggccauggaaguuuAaggcaau.......................................................................    2      1    seq
...................uaggccauggaaguuugUggcaau.......................................................................    1      1    seq
...................Gaggccauggaaguuugaggcaau.......................................................................    1      1    seq
...................uagCccauggaaguuugaggcaau.......................................................................    1      1    seq
...................uaggccauggaagCuugaggcaau.......................................................................    10     1    seq
...................uaggccauggaaguuugaggcaau.......................................................................    1758   0    seq
...................uaggccauggaaguuugaggcaGu.......................................................................    4      1    seq
...................uaggccauggaaguuugaggcGau.......................................................................    6      1    seq
...................uaggccauggaaguGugaggcaau.......................................................................    1      1    seq
...................uaggccauggUaguuugaggcaau.......................................................................    1      1    seq
...................uaggccauggaaguuugaggcaaA.......................................................................    4      1    seq
...................uaggccauggaaguCugaggcaau.......................................................................    1      1    seq
...................uaggccauggGaguuugaggcaau.......................................................................    6      1    seq
...................uaggccauggaaguuugaggcaaG.......................................................................    27     1    seq
...................Caggccauggaaguuugaggcaau.......................................................................    4      1    seq
...................uaggcUauggaaguuugaggcaau.......................................................................    4      1    seq
...................uaggccauggaaguuugaggcaaCg......................................................................    1      1    seq
```

Mature **Star**

```
uaaaaagacuauggucauuuaggccauggaaguuugaggcaauggcaaauaucuaaugugcuuaaauguucaaaauugcacaagcacuacacuaaugcagugaacgaguuuuuc
...................uaggccauggaaguuugaggcaauU......................................................................   4     1   seq
...................uaggccauggaaguuugaggcaaug......................................................................   3     0   seq
...................uaggccauggaaguuugaggcaaAg......................................................................   1     1   seq
...................uaggccauggaaguuugaggcaauAgc....................................................................   1     1   seq
...................uaggccauggaaguuugaggcaaugAc....................................................................   2     1   seq
...................uaggccauggaaguuugaggcaauAgca...................................................................   3     1   seq
...................uaggccauggaaguuugaggcaaugAca...................................................................   4     1   seq
....................aggccauggaaguuuAa.............................................................................   2     1   seq
....................aggccauggCaguuuga.............................................................................   1     1   seq
....................aggccauggaaguCuga.............................................................................   12    1   seq
....................aggccauggaaguuuCa.............................................................................   1     1   seq
....................Cggccauggaaguuuga.............................................................................   1     1   seq
....................aCgccauggaaguuuga.............................................................................   1     1   seq
....................Nggccauggaaguuuga.............................................................................   4     1   seq
....................agUccauggaaguuuga.............................................................................   1     1   seq
....................aggccCuggaaguuuga.............................................................................   1     1   seq
....................aggccauggaagAuuga.............................................................................   1     1   seq
....................aggcGauggaaguuuga.............................................................................   1     1   seq
....................aggccauggaagGuuga.............................................................................   4     1   seq
....................aggccauggaaguuCga.............................................................................   5     1   seq
....................aggccUuggaaguuuga.............................................................................   2     1   seq
....................aggccauggaaguuuga.............................................................................   2992  0   seq
....................aggccauggaGguuuga.............................................................................   10    1   seq
....................aggccauggUaguuuga.............................................................................   1     1   seq
....................aggccaGggaaguuuga.............................................................................   1     1   seq
....................aggccaugAaaguuuga.............................................................................   2     1   seq
....................aggccauggaaguAuga.............................................................................   1     1   seq
....................aggccauggaaguuGga.............................................................................   3     1   seq
....................aggccauggaUguuuga.............................................................................   1     1   seq
....................aggccaCggaaguuuga.............................................................................   2     1   seq
....................aggccauggGaguuugag............................................................................   2     1   seq
....................aggcUauggaaguuugag............................................................................   2     1   seq
....................Uggccauggaaguuugag............................................................................   1     1   seq
....................aggccauggUaguuugag............................................................................   2     1   seq
....................aggccauggaaguuuAag............................................................................   1     1   seq
....................Nggccauggaaguuugag............................................................................   3     1   seq
....................aggccauggaaguuugaU............................................................................   2     1   seq
....................aggccauggaGguuugag............................................................................   3     1   seq
....................aggccauggaaguCugag............................................................................   1     1   seq
....................aggccUuggaaguuugag............................................................................   3     1   seq
....................aggccauggaaguuuCag............................................................................   3     1   seq
....................aggccaugUaaguuugag............................................................................   1     1   seq
....................aggccauggaaguuugag............................................................................   1308  0   seq
....................aggccaCggaaguuugag............................................................................   1     1   seq
....................aggcGauggaaguuugag............................................................................   1     1   seq
....................aggccauggaaguuCgag............................................................................   2     1   seq
....................aggccaAggaaguuugagg...........................................................................   1     1   seq
....................aggccauggaaguuugagC...........................................................................   35    1   seq
....................aggccauggaaguuugaUg...........................................................................   3     1   seq
....................aggccauggaaguuugagg...........................................................................   4154  0   seq
....................Uggccauggaaguuugagg...........................................................................   3     1   seq
....................aggccauggaaguuGgagg...........................................................................   3     1   seq
....................aggccauggaaguuugagA...........................................................................   241   1   seq
....................aggcUauggaaguuugagg...........................................................................   5     1   seq
....................aggcGauggaaguuugagg...........................................................................   1     1   seq
....................aggccauggGaguuugagg...........................................................................   17    1   seq
....................aggccaCggaaguuugagg...........................................................................   7     1   seq
....................agUccauggaaguuugagg...........................................................................   1     1   seq
....................aggccauggaaguuuUagg...........................................................................   1     1   seq
....................Nggccauggaaguuugagg...........................................................................   5     1   seq
....................aggccauggaaguuugaCg...........................................................................   5     1   seq
....................aggccauggaUguuugagg...........................................................................   1     1   seq
....................aggccauggaagGuugagg...........................................................................   1     1   seq
....................aggccauggaaguuugCgg...........................................................................   2     1   seq
....................Cggccauggaaguuugagg...........................................................................   3     1   seq
....................aggccauggaaguCugagg...........................................................................   1     1   seq
....................aggccauggaaguuAgagg...........................................................................   2     1   seq
....................aggccUuggaaguuugagg...........................................................................   1     1   seq
....................aggccaugAaaguuugagg...........................................................................   2     1   seq
....................aggccauggUaguuugagg...........................................................................   2     1   seq
```

```
                   Mature     Star
uaaaaagacuauggucauuuaggccauggaaguuugaggcaauggcaaauaucuaaugugcuuaaauguucaaaauugcacaagcacuacacuaaugcagugaacgaguuuuuc
....................Gggccauggaaguuugagg...........................................................................	14	1	seq
....................aggccauggaaguuCgagg...........................................................................	5	1	seq
....................aggccauggaGguuugagg...........................................................................	9	1	seq
....................aggccauggaaguCugaggc..........................................................................	5	1	seq
....................aggccaAggaaguuugaggc..........................................................................	1	1	seq
....................Uggccauggaaguuugaggc..........................................................................	3	1	seq
....................aggccCuggaaguuugaggc..........................................................................	1	1	seq
....................aggccauggaaguuugaCgc..........................................................................	2	1	seq
....................aggccaCggaaguuugaggc..........................................................................	8	1	seq
....................aggccauggaaguuugaggc..........................................................................	6437	0	seq
....................aggccauggaaguuUaggc...........................................................................	3	1	seq
....................aggGcauggaaguuugaggc..........................................................................	1	1	seq
....................aggccUuggaaguuugaggc..........................................................................	2	1	seq
....................Gggccauggaaguuugaggc..........................................................................	29	1	seq
....................agCccauggaaguuugaggc..........................................................................	1	1	seq
....................aggccauggaGguuugaggc..........................................................................	24	1	seq
....................aggccauggaUguuugaggc..........................................................................	6	1	seq
....................aggccauggaaguGugaggc..........................................................................	2	1	seq
....................aggccauggaagCuugaggc..........................................................................	17	1	seq
....................Nggccauggaaguuugaggc..........................................................................	7	1	seq
....................aggccauggaaguuugagCc..........................................................................	19	1	seq
....................aggccauggaaguuugagAc..........................................................................	16	1	seq
....................Cggccauggaaguuugaggc..........................................................................	5	1	seq
....................aggccauggaaguuCgaggc..........................................................................	10	1	seq
....................aggccauggaaguuAgaggc..........................................................................	1	1	seq
....................aggccauggaagGuugaggc..........................................................................	2	1	seq
....................aggccauggaaguuuCaggc..........................................................................	1	1	seq
....................aggccauCgaaguuugaggc..........................................................................	2	1	seq
....................aggccauggaaguAugaggc..........................................................................	2	1	seq
....................aggccaGggaaguuugaggc..........................................................................	1	1	seq
....................aggccauggGaguuugaggc..........................................................................	27	1	seq
....................aggccaugAaaguuugaggc..........................................................................	2	1	seq
....................aggccauggaCguuugaggc..........................................................................	2	1	seq
....................aggccauggaagAuugaggc..........................................................................	5	1	seq
....................aggccaugCaaguuugaggc..........................................................................	1	1	seq
....................aggccauggaaguuuAaggc..........................................................................	1	1	seq
....................aggcUauggaaguuugaggc..........................................................................	6	1	seq
....................aggccauggCaguuugaggc..........................................................................	1	1	seq
....................aggccauggaaguuugagUc..........................................................................	1	1	seq
....................agCccauggaaguuugaggca.........................................................................	1	1	seq
....................aggccauggaaguuuAaggca.........................................................................	4	1	seq
....................aggccauggaaguuugCggca.........................................................................	1	1	seq
....................Gggccauggaaguuugaggca.........................................................................	91	1	seq
....................aggccauCgaaguuugaggca.........................................................................	1	1	seq
....................aggccauggaaCuuugaggca.........................................................................	1	1	seq
....................aggccauggaaguuAgaggca.........................................................................	6	1	seq
....................aggccauggCaguuugaggca.........................................................................	2	1	seq
....................aggccauggaaguuugUggca.........................................................................	16	1	seq
....................aggccauggaaguuugagAca.........................................................................	35	1	seq
....................aggccauggaaguuugaggGa.........................................................................	14	1	seq
....................aggccaugCaaguuugaggca.........................................................................	2	1	seq
....................aggccauggaaguGugaggca.........................................................................	3	1	seq
....................Uggccauggaaguuugaggca.........................................................................	22	1	seq
....................aggccauggaaguuugaggcC.........................................................................	64	1	seq
....................aggccauUgaaguuugaggca.........................................................................	1	1	seq
....................aggccUuggaaguuugaggca.........................................................................	5	1	seq
....................aggccauggaaguuGgaggca.........................................................................	3	1	seq
....................aggccauggaaguuCgaggca.........................................................................	36	1	seq
....................aggccauggaUguuugaggca.........................................................................	5	1	seq
....................aggccauggaagCuugaggca.........................................................................	30	1	seq
....................aggccauggaaguuugaUgca.........................................................................	4	1	seq
....................aggccaCggaaguuugaggca.........................................................................	29	1	seq
....................aggccauggaagAuugaggca.........................................................................	8	1	seq
....................aggccauggaaguuugagUca.........................................................................	3	1	seq
....................aggccauggaaguuugaggcG.........................................................................	660	1	seq
....................aggccauggaaguuuUaggca.........................................................................	2	1	seq
....................aggccauggaGguuugaggca.........................................................................	66	1	seq
....................aggccauggGaguuugaggca.........................................................................	61	1	seq
....................aggcUauggaaguuugaggca.........................................................................	18	1	seq
....................aggccGuggaaguuugaggca.........................................................................	47	1	seq
```

Mature Star

```
uaaaaagacuauggucauuuaggccauggaaguuugaggcaauggcaaauaucuaaugugcuuaaauguucaaaauugcacaagcacuacacuaaugcagugaacgaguuuuuc
....................aggcGauggaaguuugaggca.........................................................................	3	1	seq
....................aggccauggaagGuugaggca.........................................................................	10	1	seq
....................aggccauggaaguuugaggca.........................................................................	16406	0	seq
....................aggccauggUaguuugaggca.........................................................................	8	1	seq
....................aggccauggaaguAugaggca.........................................................................	1	1	seq
....................aggccaugUaaguuugaggca.........................................................................	1	1	seq
....................agUccauggaaguuugaggca.........................................................................	4	1	seq
....................Nggccauggaaguuugaggca.........................................................................	14	1	seq
....................aggccaugAaaguuugaggca.........................................................................	6	1	seq
....................aggccauggaCguuugaggca.........................................................................	3	1	seq
....................aggccauggaaguuugagCca.........................................................................	50	1	seq
....................aggccauggaaguuugaCgca.........................................................................	4	1	seq
....................Cggccauggaaguuugaggca.........................................................................	8	1	seq
....................aggccauggaaguCugaggca.........................................................................	34	1	seq
....................aggccauggaaguuugaAgca.........................................................................	6	1	seq
....................aggccCuggaaguuugaggca.........................................................................	3	1	seq
....................aggccaAggaaguuugaggca.........................................................................	2	1	seq
....................aCgccauggaaguuugaggca.........................................................................	1	1	seq
....................aggccauggaaguuugagCcaa........................................................................	1	1	seq
....................aggccauggaaguuugaggcaU........................................................................	21	1	seq
....................aggccGuggaaguuugaggcaa........................................................................	5	1	seq
....................aggccauggGaguuugaggcaa........................................................................	8	1	seq
....................agUccauggaaguuugaggcaa........................................................................	1	1	seq
....................aggccauggaaguuugaggcaC........................................................................	5	1	seq
....................Nggccauggaaguuugaggcaa........................................................................	4	1	seq
....................aggccauggaUguuugaggcaa........................................................................	3	1	seq
....................aggccauUgaaguuugaggcaa........................................................................	1	1	seq
....................aggccauggaaguuugagAcaa........................................................................	1	1	seq
....................Gggccauggaaguuugaggcaa........................................................................	11	1	seq
....................aggccauggaGguuugaggcaa........................................................................	10	1	seq
....................Cggccauggaaguuugaggcaa........................................................................	3	1	seq
....................aggccauggaaguuugaggcaa........................................................................	2067	0	seq
....................aggccauggaagCuugaggcaa........................................................................	10	1	seq
....................aggccCuggaaguuugaggcaa........................................................................	1	1	seq
....................aggccauggaaguCugaggcaa........................................................................	8	1	seq
....................aggccauggaaguuugaggcaG........................................................................	44	1	seq
....................aggccauggaaguuugaggcCa........................................................................	2	1	seq
....................aggccUuggaaguuugaggcaa........................................................................	2	1	seq
....................aggccauggaaguuugaUgcaa........................................................................	1	1	seq
....................aggccaugUaaguuugaggcaa........................................................................	1	1	seq
....................aggccauggaaguuugaggcGa........................................................................	10	1	seq
....................aggccauggaaCuuugaggcaa........................................................................	1	1	seq
....................aggcUauggaaguuugaggcaa........................................................................	5	1	seq
....................Uggccauggaaguuugaggcaa........................................................................	3	1	seq
....................aggccauggaaguuugaggcUa........................................................................	2	1	seq
....................aggccaCggaaguuugaggcaa........................................................................	2	1	seq
....................aggccauggaaguuCgaggcaa........................................................................	3	1	seq
....................aggccaugCaaguuugaggcaa........................................................................	1	1	seq
....................aggccaAggaaguuugaggcaau.......................................................................	1	1	seq
....................aggccauggaaguuugagAcaau.......................................................................	2	1	seq
....................aggccauggaaguuGgaggcaau.......................................................................	1	1	seq
....................aggccauggUaguuugaggcaau.......................................................................	1	1	seq
....................aggccauggaUguuugaggcaau.......................................................................	1	1	seq
....................aggccauggaaguuugaggcaaG.......................................................................	24	1	seq
....................aggccauggGaguuugaggcaau.......................................................................	10	1	seq
....................aggccauggaaguuugagUcaau.......................................................................	1	1	seq
....................aggccauggaaguuugaAgcaau.......................................................................	2	1	seq
....................Uggccauggaaguuugaggcaau.......................................................................	6	1	seq
....................aggccauggaCguuugaggcaau.......................................................................	1	1	seq
....................aggccauggaaguuCgaggcaau.......................................................................	2	1	seq
....................aggccauggaaguuugaggcaaA.......................................................................	6	1	seq
....................aggccauggaaguuugaggcaGu.......................................................................	8	1	seq
....................aggccauggaaguuugaggcaaC.......................................................................	237	1	seq
....................Cggccauggaaguuugaggcaau.......................................................................	1	1	seq
....................aggccauggaagCuugaggcaau.......................................................................	3	1	seq
....................Nggccauggaaguuugaggcaau.......................................................................	2	1	seq
....................aggccauggaaguuugaggcGau.......................................................................	4	1	seq
....................agCccauggaaguuugaggcaau.......................................................................	1	1	seq
....................Gggccauggaaguuugaggcaau.......................................................................	10	1	seq
....................aggGcauggaaguuugaggcaau.......................................................................	1	1	seq
```

Mature **Star**

```
uaaaaagacuauggucauuuaggccauggaaguuugaggcaauggcaaauaucuaaugugcuuaaauguucaaaauugcacaagcacuacacuaaugcagugaacgaguuuuuc
....................aggccauggaaguuugaUgcaau.......................................................................	2	1	seq
....................aggccauggaagGuugaggcaau.......................................................................	1	1	seq
....................aggccauggaaguuugaggcaUu.......................................................................	2	1	seq
....................aggccauggaaguuugUggcaau.......................................................................	2	1	seq
....................aggccauggaaguuugaggNaau.......................................................................	1	1	seq
....................aggccauggaaguCugaggcaau.......................................................................	1	1	seq
....................aggcUauggaaguuugaggcaau.......................................................................	5	1	seq
....................aggccauggaaguuugaggcUau.......................................................................	2	1	seq
....................aggccaCggaaguuugaggcaau.......................................................................	1	1	seq
....................aggccauggaaguuugaggcaau.......................................................................	2096	0	seq
....................aggccauggaGguuugaggcaau.......................................................................	6	1	seq
....................aggccGuggaaguuugaggcaau.......................................................................	4	1	seq
....................aggccauggaaguuugaggcaaGg......................................................................	2	1	seq
....................aggccauggaaguuugaggcaauU......................................................................	4	1	seq
....................aggccauggaaguuugaggcaaAg......................................................................	1	1	seq
....................aggccauggaaguuugaggcaaug......................................................................	14	0	seq
....................aggccauggaaguuCgaggcaaug......................................................................	1	1	seq
....................aggccauggaaguuugaggcaauUg.....................................................................	1	1	seq
....................aggccauggaaguuugaggcaauAg.....................................................................	4	1	seq
....................aggccauggaaguuugaggcaaugC.....................................................................	1	1	seq
....................aggccauggaaguuugaggcaauAgc....................................................................	1	1	seq
....................aggccauggaaguuugaggcaauAgca...................................................................	2	1	seq
.....................ggGcauggaaguuugag............................................................................	2	1	seq
.....................ggccauggaaguuugag............................................................................	418	0	seq
.....................ggccauggaUguuugag............................................................................	1	1	seq
.....................ggccauggaGguuugag............................................................................	3	1	seq
.....................ggccauggaaguuugaC............................................................................	1	1	seq
.....................ggccauggGaguuugag............................................................................	1	1	seq
.....................ggccauggaagGuugag............................................................................	2	1	seq
.....................ggccaAggaaguuugag............................................................................	1	1	seq
.....................ggccauggaaguuuUag............................................................................	1	1	seq
.....................ggccauggUaguuugag............................................................................	1	1	seq
.....................Cgccauggaaguuugagg...........................................................................	1	1	seq
.....................ggccauggaaguuCgagg...........................................................................	2	1	seq
.....................ggccauggaaguuugaUg...........................................................................	1	1	seq
.....................ggccauggaaguuGgagg...........................................................................	1	1	seq
.....................ggccauggaaguuugagC...........................................................................	3	1	seq
.....................ggccauggaaguuugagg...........................................................................	1223	0	seq
.....................ggccaugAaaguuugagg...........................................................................	1	1	seq
.....................Ngccauggaaguuugagg...........................................................................	1	1	seq
.....................ggccaAggaaguuugagg...........................................................................	1	1	seq
.....................ggccaCggaaguuugagg...........................................................................	4	1	seq
.....................ggccauggaagGuugagg...........................................................................	1	1	seq
.....................ggccauggaaguGugagg...........................................................................	1	1	seq
.....................gUccauggaaguuugagg...........................................................................	1	1	seq
.....................ggccauggaaguCugagg...........................................................................	3	1	seq
.....................ggccauggaGguuugagg...........................................................................	5	1	seq
.....................ggccauggGaguuugagg...........................................................................	5	1	seq
.....................ggccauggaagGuugaggc..........................................................................	1	1	seq
.....................ggccaCggaaguuugaggc..........................................................................	3	1	seq
.....................ggccauggaaguuugagAc..........................................................................	2	1	seq
.....................ggccauggaaguuGgaggc..........................................................................	1	1	seq
.....................ggccauggaaguCugaggc..........................................................................	3	1	seq
.....................ggccauggaUguuugaggc..........................................................................	3	1	seq
.....................ggccauggaaguuugaggc..........................................................................	1023	0	seq
.....................ggccauggaaguuAgaggc..........................................................................	1	1	seq
.....................ggccauggaGguuugaggc..........................................................................	4	1	seq
.....................ggGcauggaaguuugaggc..........................................................................	1	1	seq
.....................Ngccauggaaguuugaggc..........................................................................	1	1	seq
.....................ggccauggaagAuugaggc..........................................................................	2	1	seq
.....................gUccauggaaguuugaggc..........................................................................	1	1	seq
.....................ggccauggaaguuugagCc..........................................................................	5	1	seq
.....................ggccauggaaguuCgaggc..........................................................................	2	1	seq
.....................ggccaugAaaguuugaggc..........................................................................	3	1	seq
.....................ggccaAggaaguuugaggc..........................................................................	1	1	seq
.....................ggccauggGaguuugaggc..........................................................................	7	1	seq
.....................ggccauggaaguuugCggc..........................................................................	1	1	seq
.....................ggccauggaaguuugagCca.........................................................................	11	1	seq
.....................ggccauggaaguuugaggcG.........................................................................	64	1	seq
.....................ggccauggaaguuAgaggca.........................................................................	2	1	seq
```

Mature **Star**

```
uaaaaagacuauggucauuuaggccauggaaguuugaggcaauggcaaauaucuaaugugcuuaaauguucaaaauugcacaagcacuacacuaaugcagugaacgaguuuuuc
.....................ggGcauggaaguuugaggca.........................................................................	2	1	seq
.....................ggccauggaagGuugaggca.........................................................................	1	1	seq
.....................ggccauggaaguAugaggca.........................................................................	2	1	seq
.....................ggccauggGaguuugaggca.........................................................................	8	1	seq
.....................ggccauggaaguuugagAca.........................................................................	1	1	seq
.....................ggccauggaaguCugaggca.........................................................................	1	1	seq
.....................ggccauggaaguuugUggca.........................................................................	1	1	seq
.....................gUccauggaaguuugaggca.........................................................................	4	1	seq
.....................ggccauggaaguuCgaggca.........................................................................	1	1	seq
.....................ggccauggaaguuugaggca.........................................................................	1710	0	seq
.....................ggccaCggaaguuugaggca.........................................................................	1	1	seq
.....................ggccauggaaguuugaggGa.........................................................................	1	1	seq
.....................ggccaugAaaguuugaggca.........................................................................	1	1	seq
.....................ggcUauggaaguuugaggca.........................................................................	2	1	seq
.....................ggccauggaaguuugCggca.........................................................................	1	1	seq
.....................ggccauggaaguuugaggcC.........................................................................	1	1	seq
.....................Ngccauggaaguuugaggca.........................................................................	1	1	seq
.....................ggccGuggaaguuugaggca.........................................................................	5	1	seq
.....................ggccauggaagCuugaggca.........................................................................	3	1	seq
.....................ggccauggaaguuugaAgca.........................................................................	1	1	seq
.....................ggccauggaGguuugaggca.........................................................................	3	1	seq
.....................Ngccauggaaguuugaggcaa........................................................................	1	1	seq
.....................ggccauggaaguuugagCcaa........................................................................	1	1	seq
.....................ggccaugAaaguuugaggcaa........................................................................	1	1	seq
.....................ggccauggaaguuugaggcGa........................................................................	2	1	seq
.....................ggccGuggaaguuugaggcaa........................................................................	1	1	seq
.....................ggccauggaaguuugagAcaa........................................................................	1	1	seq
.....................ggccauggaaguuugaggcaa........................................................................	286	0	seq
.....................ggGcauggaaguuugaggcaa........................................................................	1	1	seq
.....................ggccauggaaguuugaggcaU........................................................................	4	1	seq
.....................ggccauggUaguuugaggcaa........................................................................	1	1	seq
.....................ggccauggaaguuugUggcaa........................................................................	1	1	seq
.....................ggccauggGaguuugaggcaa........................................................................	1	1	seq
.....................ggccauggaaguuugaggcaG........................................................................	8	1	seq
.....................ggccauggaGguuugaggcaa........................................................................	1	1	seq
.....................ggccauggaaguuugaggcCa........................................................................	1	1	seq
.....................ggccauggaagCuugaggcaa........................................................................	1	1	seq
.....................ggccUuggaaguuugaggcaa........................................................................	1	1	seq
.....................ggccauggaGguuugaggcaau.......................................................................	1	1	seq
.....................ggccauggaaguuCgaggcaau.......................................................................	1	1	seq
.....................ggccauggaaguuugaggcaaC.......................................................................	40	1	seq
.....................Ngccauggaaguuugaggcaau.......................................................................	1	1	seq
.....................ggccauggaagCuugaggcaau.......................................................................	1	1	seq
.....................ggccauggaaguuugaggcaau.......................................................................	350	0	seq
.....................ggccaCggaaguuugaggcaau.......................................................................	1	1	seq
.....................ggccauggaaguuugaggcaaG.......................................................................	3	1	seq
.....................ggccauggaaguuugaggcaGu.......................................................................	1	1	seq
.....................ggcUauggaaguuugaggcaau.......................................................................	1	1	seq
.....................ggccauggaaguuugaggcGau.......................................................................	1	1	seq
.....................ggccGuggaaguuugaggcaau.......................................................................	1	1	seq
.....................ggGcauggaaguuugaggcaau.......................................................................	1	1	seq
.....................ggccauggaaguCugaggcaau.......................................................................	1	1	seq
.....................ggccauggaaguuugaUgcaau.......................................................................	1	1	seq
.....................ggccauggaaguuugaggcaaA.......................................................................	1	1	seq
.....................ggccauggaaguAugaggcaau.......................................................................	1	1	seq
.....................ggccauggGaguuugaggcaau.......................................................................	2	1	seq
.....................ggccauggaaguuugaggcaauU......................................................................	1	1	seq
.....................ggccauggaaguuugaggcaaugAc....................................................................	2	1	seq
.....................ggccauggaaguuugaggcaauAgc....................................................................	3	1	seq
.....................ggccauggaaguuugaggcaaugAca...................................................................	1	1	seq
......................gccauggaaguuugCgg...........................................................................	1	1	seq
......................gccauggaaguuugagg...........................................................................	479	0	seq
......................gccauggaagGuugagg...........................................................................	2	1	seq
......................gccauggaaguuCgagg...........................................................................	2	1	seq
......................gGcauggaaguuugagg...........................................................................	1	1	seq
......................gccaAggaaguuugagg...........................................................................	1	1	seq
......................gccauggaCguuugagg...........................................................................	1	1	seq
......................gccauggaaguuugagC...........................................................................	1	1	seq
......................gccaCggaaguuugagg...........................................................................	2	1	seq
......................gccauggaGguuugagg...........................................................................	1	1	seq
```

```
                    Mature      Star
uaaaaagacuauggucauuuaggccauggaaguuugaggcaauggcaaauaucuaaugugcuuaaauguucaaaauugcacaagcacuacacuaaugcagugaacgaguuuuuc
......................gccauggaUguuugagg...........................................................................    2      1     seq
......................gccauggGaguuugaggc..........................................................................    1      1     seq
......................gGcauggaaguuugaggc..........................................................................    2      1     seq
......................gccauggaaguuuUaggc..........................................................................    1      1     seq
......................gccauggaaguuugagCc..........................................................................    1      1     seq
......................gccauggaaguuugaggc..........................................................................    94     0     seq
......................gcUauggaaguuugaggc..........................................................................    2      1     seq
......................gccaugAaaguuugaggca.........................................................................    1      1     seq
......................gccauggaaguCugaggca.........................................................................    2      1     seq
......................gccauggaagGuugaggca.........................................................................    1      1     seq
......................gccGuggaaguuugaggca.........................................................................    2      1     seq
......................gccauggaaguuugagAca.........................................................................    1      1     seq
......................gccauggaUguuugaggca.........................................................................    1      1     seq
......................gcUauggaaguuugaggca.........................................................................    4      1     seq
......................gccUuggaaguuugaggca.........................................................................    1      1     seq
......................gccauggaaguuugaggca.........................................................................    437    0     seq
......................Cccauggaaguuugaggca.........................................................................    1      1     seq
......................Uccauggaaguuugaggca.........................................................................    1      1     seq
......................gccauggGaguuugaggca.........................................................................    3      1     seq
......................gccauggaaguuCgaggca.........................................................................    1      1     seq
......................gccauggaaguuugaggcG.........................................................................    23     1     seq
......................gccauggaGguuugaggca.........................................................................    2      1     seq
......................gccaCggaaguuugaggca.........................................................................    3      1     seq
......................Uccauggaaguuugaggcaa........................................................................    1      1     seq
......................gccauggaaguuugUggcaa........................................................................    1      1     seq
......................gccauggaaguuugaggcaa........................................................................    42     0     seq
......................gccauggaaguuugaggcGa........................................................................    2      1     seq
......................gccauggaaguuugaggcaU........................................................................    1      1     seq
......................gcUauggaaguuugaggcaa........................................................................    1      1     seq
......................gccauggaaguuugaggcaG........................................................................    1      1     seq
......................gccauggaaguuugaggcaau.......................................................................    33     0     seq
......................gccauggaaguuugaggcaaG.......................................................................    1      1     seq
......................Uccauggaaguuugaggcaau.......................................................................    1      1     seq
.......................Gcauggaaguuugaggc..........................................................................    3      1     seq
.......................ccaugAaaguuugaggc..........................................................................    1      1     seq
.......................ccaCggaaguuugaggc..........................................................................    3      1     seq
.......................ccauggaCguuugaggc..........................................................................    1      1     seq
.......................ccauggaagCuugaggc..........................................................................    1      1     seq
.......................ccauggaaguCugaggc..........................................................................    2      1     seq
.......................ccauggaaguuugagCc..........................................................................    4      1     seq
.......................ccaAggaaguuugaggc..........................................................................    1      1     seq
.......................ccauggUaguuugaggc..........................................................................    1      1     seq
.......................ccauggaaguuCgaggc..........................................................................    1      1     seq
.......................ccauggaaguuugaggc..........................................................................    1167   0     seq
.......................ccauggaaguuugCggc..........................................................................    2      1     seq
.......................ccauggaaguuugaCgc..........................................................................    2      1     seq
.......................ccauggaaguGugaggc..........................................................................    1      1     seq
.......................ccauggGaguuugaggc..........................................................................    3      1     seq
.......................ccauggaaguuugagUca.........................................................................    1      1     seq
.......................ccauggaaguuugaggcC.........................................................................    8      1     seq
.......................Gcauggaaguuugaggca.........................................................................    2      1     seq
.......................ccaCggaaguuugaggca.........................................................................    2      1     seq
.......................ccGuggaaguuugaggca.........................................................................    7      1     seq
.......................ccaugAaaguuugaggca.........................................................................    2      1     seq
.......................ccauUgaaguuugaggca.........................................................................    1      1     seq
.......................ccauggaaguuCgaggca.........................................................................    6      1     seq
.......................ccauggaaguuugaggcG.........................................................................    123    1     seq
.......................ccauggaaguuugUggca.........................................................................    2      1     seq
.......................ccauggaaguuugagCca.........................................................................    4      1     seq
.......................ccauggUaguuugaggca.........................................................................    1      1     seq
.......................ccauggaaguuugaAgca.........................................................................    3      1     seq
.......................ccauggaaguuugaggca.........................................................................    2767   0     seq
.......................ccaGggaaguuugaggca.........................................................................    1      1     seq
.......................ccauggaagCuugaggca.........................................................................    5      1     seq
.......................Ncauggaaguuugaggca.........................................................................    6      1     seq
.......................ccauggaUguuugaggca.........................................................................    1      1     seq
.......................ccauggGaguuugaggca.........................................................................    8      1     seq
.......................ccauggaaguCugaggca.........................................................................    6      1     seq
.......................ccauggaaguAugaggca.........................................................................    1      1     seq
.......................ccaAggaaguuugaggca.........................................................................    2      1     seq
```

Mature **Star**

uaaaaagacuauggucauuuaggccauggaaguuugaggcaauggcaaauaucuaaugugcuuaaauguucaaaauugcacaagcacuacacuaaugcagugaacgaguuuuuc			
......................ccUuggaaguuugaggca..	1	1	seq
......................ccauggaaguuuAaggca..	1	1	seq
......................ccauggaaguuugaggcGa...	2	1	seq
......................Gcauggaaguuugaggcaa...	1	1	seq
......................ccauggGaguuugaggcaa...	1	1	seq
......................ccauggaaguuugaggcaC...	1	1	seq
......................ccauggaaguuugaggcaG...	10	1	seq
......................ccauggaaguuugaggcaa...	355	0	seq
......................ccaCggaaguuugaggcaa...	1	1	seq
......................ccGuggaaguuugaggcaa...	1	1	seq
......................ccauggaagGuugaggcaa...	1	1	seq
......................ccauggaaguuCgaggcaau..	1	1	seq
......................ccauggaGguuugaggcaau..	4	1	seq
......................ccauggaaguuugaggcaaA..	2	1	seq
......................ccGuggaaguuugaggcaau..	2	1	seq
......................ccauggaaguCugaggcaau..	3	1	seq
......................ccauggaaguuugaggcaaG..	2	1	seq
......................ccaCggaaguuugaggcaau..	1	1	seq
......................ccauggaaguuugaggcaaC..	55	1	seq
......................ccauggaaguuugaggcGau..	1	1	seq
......................ccauggaCguuugaggcaau..	1	1	seq
......................ccauggaaguuugaggcaau..	417	0	seq
......................ccauggaaguuugaggcaaug...	3	0	seq
......................ccauggaaguuugaggcaUug...	2	1	seq
......................ccauggaaguuugaggcaaugU..	1	1	seq
......................ccauggaaguuugaggcaaugAc...	1	1	seq
.......................cauggaaguuugCggca..	1	1	seq
.......................cauggaaguuugaggcG..	63	1	seq
.......................Gauggaaguuugaggca..	1	1	seq
.......................cGuggaaguuugaggca..	5	1	seq
.......................cauggaagGuugaggca..	1	1	seq
.......................cauggaaguuCgaggca..	1	1	seq
.......................cauggaaguuugUggca..	1	1	seq
.......................Nauggaaguuugaggca..	1	1	seq
.......................cauggaaguuugagCca..	1	1	seq
.......................cauggaaguCugaggca..	2	1	seq
.......................cauggaaguuAgaggca..	1	1	seq
.......................cauggGaguuugaggca..	4	1	seq
.......................caugUaaguuugaggca..	1	1	seq
.......................cauggaaguuugaggcC..	7	1	seq
.......................cauggaaguuugaAgca..	1	1	seq
.......................cauggUaguuugaggca..	1	1	seq
.......................cauggaCguuugaggca..	1	1	seq
.......................cauggaagCuugaggca..	6	1	seq
.......................cCuggaaguuugaggca..	2	1	seq
.......................caCggaaguuugaggca..	7	1	seq
.......................cauggaaguuugaggca..	1556	0	seq
.......................cauggaaguuCgaggcaa...	1	1	seq
.......................cauggaaguuugaggcaU...	1	1	seq
.......................cGuggaaguuugaggcaa...	1	1	seq
.......................caCggaaguuugaggcaa...	1	1	seq
.......................cauggaaguuugaggcaG...	6	1	seq
.......................cauggaagCuugaggcaa...	1	1	seq
.......................cUuggaaguuugaggcaa...	1	1	seq
.......................cauggaaguuugaggcaa...	203	0	seq
.......................cauggaaguuugaAgcaau..	1	1	seq
.......................cauggGaguuugaggcaau..	2	1	seq
.......................cGuggaaguuugaggcaau..	1	1	seq
.......................cauggaaguuAgaggcaau..	1	1	seq
.......................cauggaaguuugaggcaau..	254	0	seq
.......................cauggaaguuugaggcaaA..	1	1	seq
.......................cauggaaguuugaggcaaC..	29	1	seq
.......................cauggaagCuugaggcaau..	1	1	seq
.......................cauggaaguuugaUgcaau..	1	1	seq
.......................cauggaaguuugaggcaUug...	1	1	seq
.......................cauggaaguuugaggcaaug...	1	0	seq
.......................cauggaaguuugaggcaaugAca..	2	1	seq
........................auggaaguAugaggcaa...	1	1	seq
........................auggaagCuugaggcaa...	1	1	seq
........................auggaaguuugaggcGa...	1	1	seq

Mature **Star**

```
uaaaaagacuauggucauuuaggccauggaaguuugaggcaauggcaaauaucuaaugugcuuaaauguucaaaauugcacaagcacuacacuaaugcagugaacgaguuuuuc
.........................auggaaguuugaggcaU........................................................................	1	1	seq
.........................auggaaguuugaggcaG........................................................................	2	1	seq
.........................auggGaguuugaggcaa........................................................................	1	1	seq
.........................auggaaguuugaggcaa........................................................................	166	0	seq
.........................aAggaaguuugaggcaau.......................................................................	1	1	seq
.........................auggaaguuugaggcaaA.......................................................................	1	1	seq
.........................auggaaguuugaggcaGu.......................................................................	1	1	seq
.........................auggaaguuCgaggcaau.......................................................................	2	1	seq
.........................auggaGguuugaggcaau.......................................................................	1	1	seq
.........................Guggaaguuugaggcaau.......................................................................	1	1	seq
.........................auggaaguuugaggcaaG.......................................................................	1	1	seq
.........................auCgaaguuugaggcaau.......................................................................	1	1	seq
.........................auggaagCuugaggcaau.......................................................................	2	1	seq
.........................auggaaguuugaggcaau.......................................................................	292	0	seq
.........................auggaaguuugaUgcaau.......................................................................	1	1	seq
.........................auggaaguuugaggcaaug......................................................................	2	0	seq
..........................uggaaguCugaggcaau.......................................................................	1	1	seq
..........................uggaGguuugaggcaau.......................................................................	4	1	seq
..........................uggaaguuugaAgcaau.......................................................................	1	1	seq
..........................uggaagCuugaggcaau.......................................................................	3	1	seq
..........................Aggaaguuugaggcaau.......................................................................	1	1	seq
..........................uggaaguuugaggcaGu.......................................................................	3	1	seq
..........................uggaaguuCgaggcaau.......................................................................	2	1	seq
..........................uggaaguuugaggcaaA.......................................................................	3	1	seq
..........................Cggaaguuugaggcaau.......................................................................	1	1	seq
..........................Gggaaguuugaggcaau.......................................................................	2	1	seq
..........................uggaaguuugaggcaaG.......................................................................	8	1	seq
..........................uggGaguuugaggcaau.......................................................................	2	1	seq
..........................uggaaguuugaggcaau.......................................................................	724	0	seq
..........................Nggaaguuugaggcaau.......................................................................	3	1	seq
..........................uggaaguuAgaggcaau.......................................................................	1	1	seq
..........................uggaaguuugUggcaaug......................................................................	1	1	seq
..........................uggaaguuugaggcaauU......................................................................	1	1	seq
..........................uggaaguuugaggcaaug......................................................................	7	0	seq
..........................uggaaguuugaggcaaugAc....................................................................	3	1	seq
..........................uggaaguuugaggcaaugAca...................................................................	1	1	seq
...........................ggaaguuugaggcaaug......................................................................	1	0	seq
............................gaaguuugaggcaauUg.....................................................................	1	1	seq
.......................................................................................uacacuaaugcaguCaacgaguuuu..	1	1	seq
...........................................................................................cuGaugcagugaacgag......	1	1	seq
...........................................................................................cuGaugcagugaacgagu.....	2	1	seq
...........................................................................................cuaaugcaguCaacgaguu....	1	1	seq
.............................................................................................aaugcaguCaacgaguu....	1	1	seq
```

Provisional ID	:	MA_1044870_28295
Score total	:	0.2
Score for star read(s)	:	-1.3
Score for read counts	:	0
Score for mfe	:	0
Score for randfold	:	1.6
Score for cons. seed	:	
Total read count	:	52
Mature read count	:	51
Loop read count	:	1
Star read count	:	0

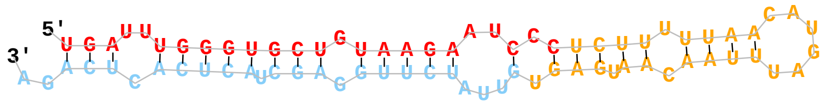

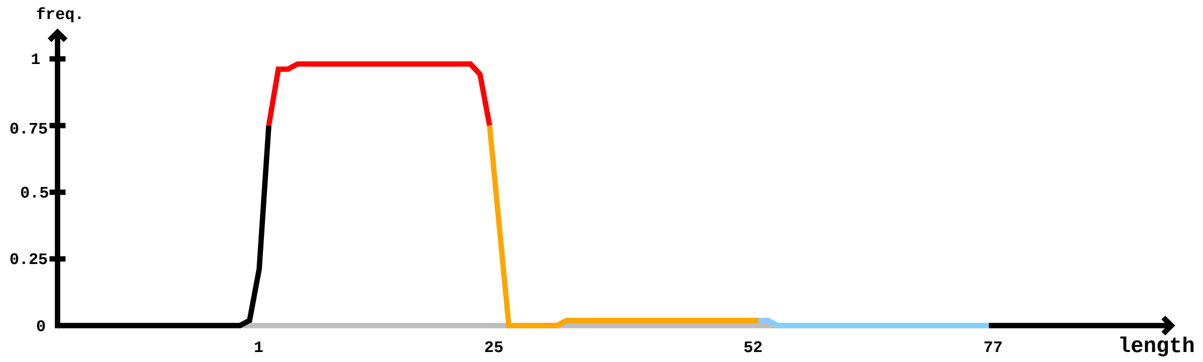

Mature Star

```
5'- uuuuuuuaaguuuuucaauuugauuugggugcuguaagaaucccucuuuuuaacaugauuuaacaaugaguguuaucuuggagcuacucacucagauccuggcagaugucagga   -3'    exp
    .................((((((..((((((((.(((((..(.(((((.((((......)))).)).))).)...))))).))).))))).))))))((((((....)))))).    reads    mm    sample
    ..................uuugauuugggugcuguaagaauc........................................................................    1        0     seq
    ...................uAgauuugggugcuguaagaaucc.......................................................................    6        1     seq
    ...................uugauuugggugcuguaagaaucc.......................................................................    3        0     seq
    ...................uugauuugggugUuguaagaaucc.......................................................................    1        1     seq
    ....................Agauuugggugcuguaagaauccc......................................................................    20       1     seq
    ....................ugauuugggugcuguaagaauccc......................................................................    8        0     seq
    .....................gauuugggugcuguaagaaucccC.....................................................................    2        1     seq
    .....................gauuugggugcuguaagaaucccG.....................................................................    1        1     seq
    .....................gauuugggugcuguaagaaucccu.....................................................................    8        0     seq
    .......................uuugggugUuguaagaauc........................................................................    1        1     seq
    ...................................................aaGaugauuuaacaaugagugu.........................................    1        1     seq
```

Provisional ID : MA_105155_10576
Score total : 0.3
Score for star read(s) : -1.3
Score for read counts : 0
Score for mfe : 0
Score for randfold : 1.6
Score for cons. seed :
Total read count : 99
Mature read count : 99
Loop read count : 0
Star read count : 0

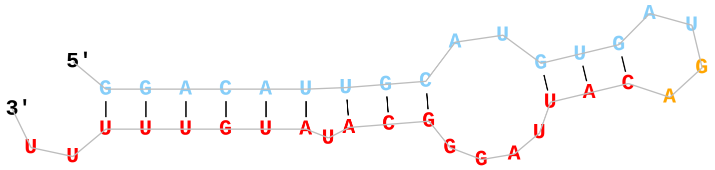

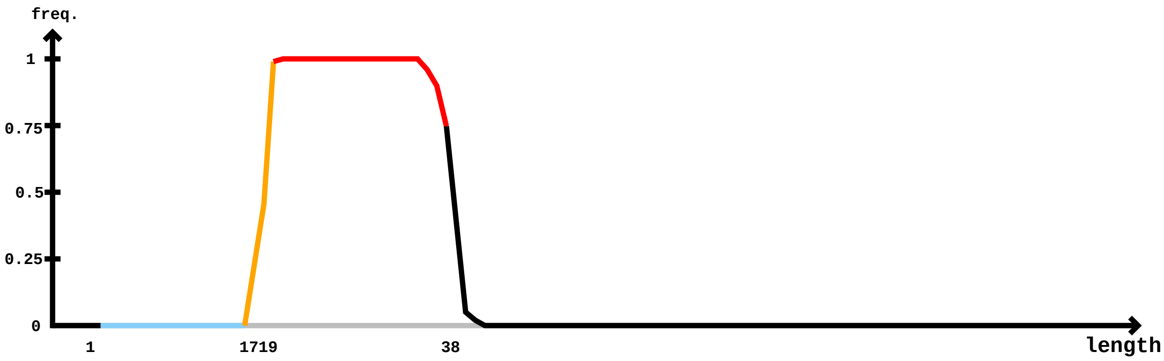

Star Mature

```
5'- gaaggacauugcaugugaugacauuagggcauauguuuuuauagaacauuucauuucauagauguugaaauuagugcagcuagaaaagccauucucugggaggauggcaua   -3'    exp
    .((((((((((..(((....)))....))).))))))))).(((..((((..((((((.......)))))).))))...))).....(((((((((...)))))))))...   reads   mm   sample
    ....................acauuagggcauauguC..........................................................................   3       1    seq
    ....................acauuagggcauauguu..........................................................................   1       0    seq
    ....................acauuagggcauauguuu.........................................................................   2       0    seq
    ....................acauuagggcauauguuA.........................................................................   1       1    seq
    ....................acauuagggcauauguuuu........................................................................   8       0    seq
    ....................acauuagggcauauguuAu........................................................................   1       1    seq
    ....................acauuagggcauauguuuuu.......................................................................   9       0    seq
    ....................acauuagggcauauguuuuG.......................................................................   1       1    seq
    ....................acauuagggcGuauguuuuua......................................................................   1       1    seq
    ....................acauuagggcauauguuuuua......................................................................   18      0    seq
    .....................cauuagggcauauguuu.........................................................................   2       0    seq
    .....................cauuagggcauauguuC.........................................................................   1       1    seq
    .....................cauuagggcauauguuuu........................................................................   5       0    seq
    .....................cauuagggcauauguuuuu.......................................................................   26      0    seq
    .....................cauuagggcauauguuuuG.......................................................................   1       1    seq
    .....................cauuagggcauauguuuuA.......................................................................   1       1    seq
    .....................cauuagggcGuauguuuuu.......................................................................   1       1    seq
    .....................cauuagggcauauguuuuua......................................................................   11      0    seq
    .....................cauuagggcauauguuuuuCu.....................................................................   3       1    seq
    .....................cauuagggcauauguuuuuGua....................................................................   1       1    seq
    .....................cauuagggcauauguuuuuUua....................................................................   1       1    seq
    ......................auuagggcauauguuuG........................................................................   1       1    seq
```

Provisional ID	:	MA_1061068_37017
Score total	:	1.8
Score for star read(s)	:	-1.3
Score for read counts	:	0
Score for mfe	:	1.5
Score for randfold	:	1.6
Score for cons. seed	:	
Total read count	:	621
Mature read count	:	621
Loop read count	:	0
Star read count	:	0

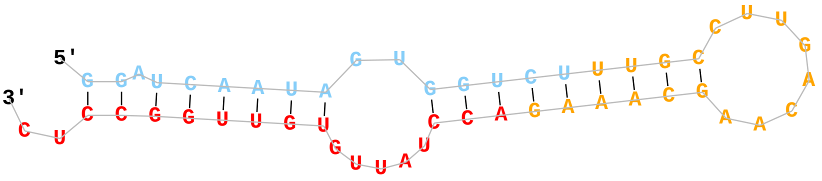

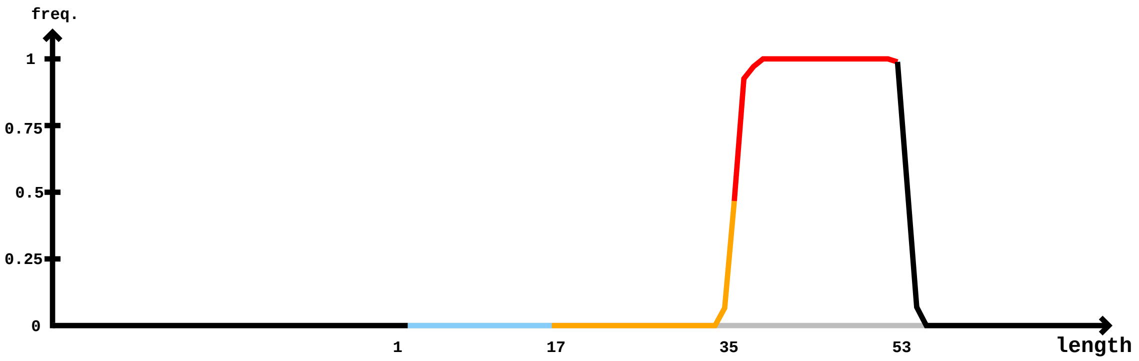

Star Mature

```
5'- gaggaaugcucugaguauuccugaaaauguuacgaggaucaauaguggucuuugccuugacaagcaaagaccuauuguguuggccuccuuuuaguuggguacccgaaa   -3'    exp
    .((((((((.....))))))))...........((((.((((((..(((((((((........))))))))).....))))))))))).......((((....))))..    reads      mm      sample
    ....................................................................gCccuauuguguuggccu......................    1          1       seq
    ....................................................................gUccuauuguguuggccuc.....................    35         1       seq
    ....................................................................gUccuauuguguuggccucc....................    4          1       seq
    ....................................................................gaccuauuguguuggccuUcu...................    1          1       seq
    .....................................................................Nccuauuguguuggccu......................    2          1       seq
    .....................................................................accuauuguguuggccu......................    2          0       seq
    .....................................................................acAuauuguguuggccu......................    1          1       seq
    .....................................................................Gccuauuguguuggccu......................    1          1       seq
    .....................................................................accuauuguguuggccuU.....................    1          1       seq
    .....................................................................Cccuauuguguuggccuc.....................    1          1       seq
    .....................................................................Uccuauuguguuggccuc.....................    228        1       seq
    .....................................................................Uccuauuguguuggccucc....................    11         1       seq
    .....................................................................accuauuguguuggccuUc....................    2          1       seq
    ......................................................................Ucuauuguguuggccuc.....................    1          1       seq
    ......................................................................ccuauuguguuggccuc.....................    196        0       seq
    ......................................................................ccuaCuguguuggccuc.....................    3          1       seq
    ......................................................................ccuauuguguuggccGc.....................    1          1       seq
    ......................................................................ccuauuguguuggccuG.....................    21         1       seq
    ......................................................................ccuauugCguuggccuc.....................    4          1       seq
    ......................................................................ccuauuguguCggccuc.....................    1          1       seq
    ......................................................................ccuauuguguuggccuA.....................    26         1       seq
    ......................................................................ccuauugugCuggccuc.....................    1          1       seq
    ......................................................................ccuauuguguuggccCc.....................    2          1       seq
    ......................................................................ccuauuguguuggcUuc.....................    1          1       seq
    ......................................................................ccuauuguguuggccuGc....................    2          1       seq
    ......................................................................ccuauuguguuggccucc....................    10         0       seq
    ......................................................................ccuauuguguuggccucU....................    1          1       seq
    ......................................................................ccuauuguguuggccuccA...................    1          1       seq
    ......................................................................ccuauuguguuggccuUcu...................    13         1       seq
    ......................................................................ccuauuguguuggccucGu...................    1          1       seq
    .......................................................................cuauuguguuggccucU....................    2          1       seq
    .......................................................................cuauuguguuggccuGc....................    3          1       seq
    .......................................................................Auauuguguuggccucc....................    1          1       seq
    .......................................................................cuauuguguuggccuAc....................    1          1       seq
```

Star Mature

```
gaggaaugcucugaguauuccugaaaauguuacgaggaucaauaguggucuuugccuugacaagcaaagaccuauuguguuggccuccuuuuaguuggguacccgaaa
.......................................................................cuauuguguuggccucc....................   11   0   seq
.......................................................................cuauCguguuggccucc....................   1    1   seq
.......................................................................cuauuguguuggccuUcu...................   6    1   seq
.......................................................................cuauuguguuggccuccG...................   2    1   seq
.......................................................................cuauuguguuggccuccA...................   1    1   seq
........................................................................uauuguguuggccuUcu...................   14   1   seq
........................................................................uauuguguuggccuccG...................   4    1   seq
```

Provisional ID	:	MA_10961_13189
Score total	:	22.7
Score for star read(s)	:	3.9
Score for read counts	:	14.9
Score for mfe	:	2.3
Score for randfold	:	1.6
Score for cons. seed	:	
Total read count	:	41
Mature read count	:	38
Loop read count	:	0
Star read count	:	3

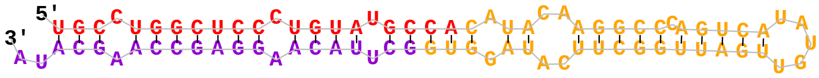

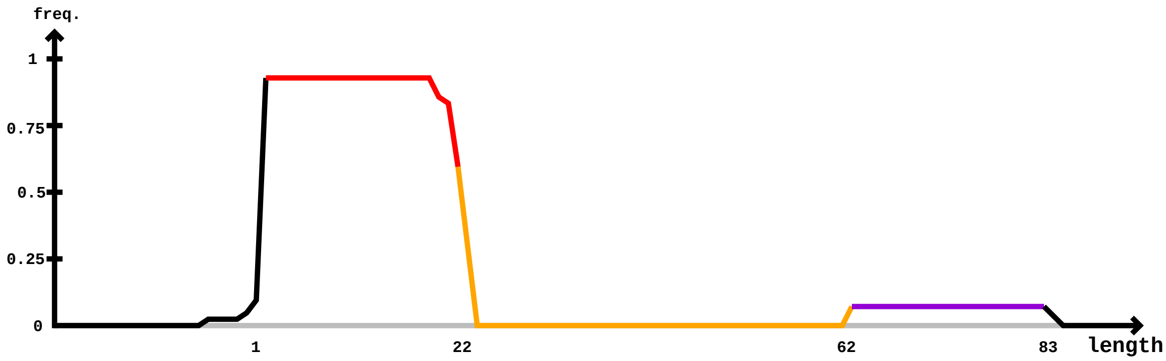

Mature **Star**

```
5'- uauaugcaaagguugaugugugccuggcucccuguaugccacauacaaggcccagucauauguugauuggcuucauaggugagcuuacaaggagccaagcauacguuuucuu   -3'   obs
    uauaugcaaagguugaugugugccuggcucccuguaugccacauacaaggcccagucauauguugauuggcuucauaggugagcuuacaaggagccaagcauacguuuucuu          exp
    .........(((..(((((((((.(((((((.((((.(((((.((..(((((.(((((.....))))))))))..)).))))).)))).))))))).)))))))))..)))   reads   mm   sample
    ..............gaugugugccuggcucccuguaCgcca......................................................................   1   1   seq
    ..................uCugccuggcucccuguaugc........................................................................   1   1   seq
    ...................gugccuggcucccuguaugcc.......................................................................   1   0   seq
    ...................Cugccuggcucccuguaugcc.......................................................................   1   1   seq
    ....................ugccuggcucccuguaug.........................................................................   2   0   seq
    ....................ugccuggcucUcuguaug.........................................................................   1   1   seq
    ....................ugccuggcucccCguaugcc.......................................................................   1   1   seq
    ....................ugccuggcucccuguaugcc.......................................................................   5   0   seq
    ....................ugccuggcucccuguaugcU.......................................................................   2   1   seq
    ....................ugccuggcucccuguaugcca......................................................................   21  0   seq
    ....................ugccuggcucccuguaugccU......................................................................   1   1   seq
    ....................Ngccuggcucccuguaugcca......................................................................   1   1   seq
    ....................ugccAggcucccuguaugcca......................................................................   1   1   seq
    .................................................................................gcuuacaaggagccaagcaua.........   3   0   seq
```

Provisional ID	:	MA_11004_2293
Score total	:	2.5
Score for star read(s)	:	-1.3
Score for read counts	:	0
Score for mfe	:	2.2
Score for randfold	:	1.6
Score for cons. seed	:	
Total read count	:	29
Mature read count	:	29
Loop read count	:	0
Star read count	:	0

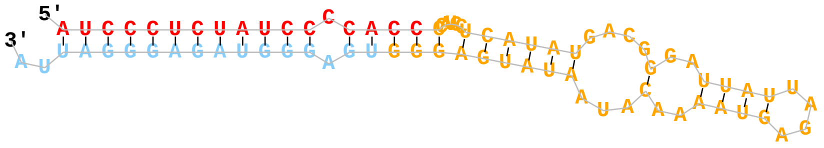

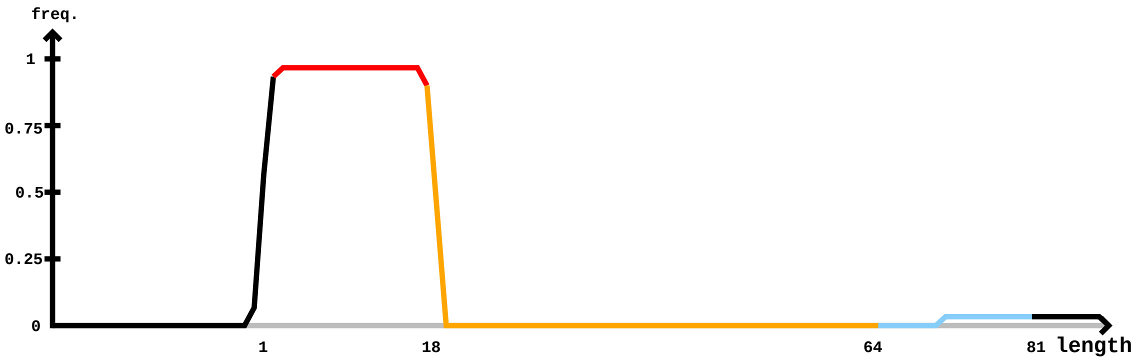

Mature **Star**

```
5'- auauuauguuuuacucuaauaaucccucuauccccaccccauccucauaugacgggauuauuagaguaaaacauaauaugagggugaggguagagggauuauuagagu   -3'    exp
    ............(((((((((((((((((((((.(((((.....((((((....(..((((....))))..)...))))))))))).)))))))))))))))))))))    reads       mm        sample
    ...................Aaaucccucuauccccacc......................................................................    2           1         seq
    ....................aaucccucuauccccac.......................................................................    2           0         seq
    ....................aaucccucuauccccacc......................................................................    8           0         seq
    ....................aaucccucuauccccacU......................................................................    1           1         seq
    ....................aaucccucuauccccaccG.....................................................................    1           1         seq
    ....................aaucccucuauccccaccA.....................................................................    3           1         seq
    .....................aucccucuauccccacA......................................................................    1           1         seq
    .....................aucccucuauccccacc......................................................................    9           0         seq
    .....................aucccucuauccccaccG.....................................................................    1           1         seq
    ......................ucccucuauccccaccG.....................................................................    1           1         seq
    ...........................................................................................agagggauuauuagaUu    1           1         seq
```

Provisional ID	:	MA_1111171_33820
Score total	:	2463
Score for star read(s)	:	3.9
Score for read counts	:	2462.1
Score for mfe	:	-0.7
Score for randfold	:	-2.2
Score for cons. seed	:	
Total read count	:	4841
Mature read count	:	3376
Loop read count	:	27
Star read count	:	1438

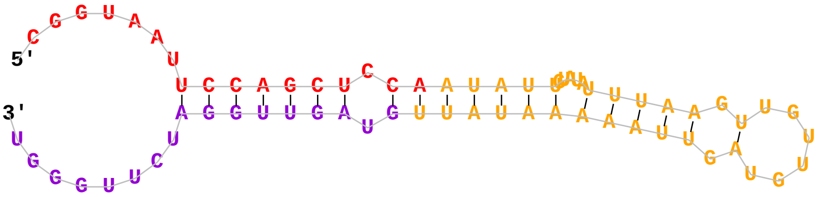

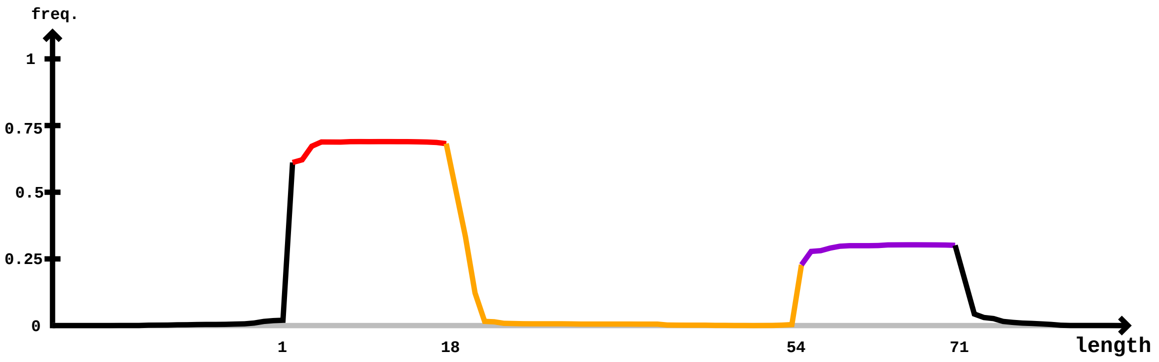


```
                          Mature                                            Star
5'- ggaaaguuuggugccaucagccacgguaauuccagcuccaauauuguauauuuaaguuguuguaguuaaaaaauauuguaguuggaucuuggguuguaaaggucaguccac  -3'   obs
    ggaaaguuuggugccaucagccacgguaauuccagcuccaauauuguauauuuaaguuguuguaguuaaaaaauauuguaguuggaucuuggguuguaaaggucaguccac        exp
    ........(((.(((..(((((.(((....(((((((.(((((((.....(((((.(......).))))))))))))).))))))).))))))))....)))....))).  reads     mm      sample
    .....guCuggugccaucagcc.........................................................................................  1         1       seq
    ........uggugccaGcagccacg......................................................................................  1         1       seq
    ........uggugccaGcagccacggu....................................................................................  1         1       seq
    ........uggugccaGcagccacggua...................................................................................  2         1       seq
    ........uggugccaGcagccacgguaauu................................................................................  1         1       seq
    ........uggugccaGcagccacgguaauuccagcuccaau.....................................................................  1         1       seq
    .........ggugccaucagccGcggua...................................................................................  1         1       seq
    ..........gugccaGcagccacgguaauuccagcuccaau.....................................................................  1         1       seq
    ...........ugccaucagccGcggua...................................................................................  1         1       seq
    ...........ugccaGcagccacgguaauucca.............................................................................  1         1       seq
    ...........ugccaGcagccacgguaauuccagcu..........................................................................  1         1       seq
    ...........ugccaGcagccacgguaauuccagcucca.......................................................................  2         1       seq
    ............gccaucagccGcgguaauu................................................................................  1         1       seq
    .............ccaucagccGcgguaauuccagc...........................................................................  1         1       seq
    .............ccaGcagccacgguaauuccagcu..........................................................................  1         1       seq
    .............ccaucagccGcgguaauuccagcuc.........................................................................  1         1       seq
    .............ccaucagccGcgguaauuccagcucca.......................................................................  1         1       seq
    ..............caucagccGcgguaauuccagcucca.......................................................................  1         1       seq
    ..............caGcagccacgguaauuccagcucca.......................................................................  1         1       seq
    ................ucagccGcgguaauuccagc...........................................................................  1         1       seq
    ................ucagccGcgguaauuccagcuc.........................................................................  1         1       seq
    .................cagccacgguaauuccagcu..........................................................................  1         0       seq
    .................cagccacgguaauuccagcuc.........................................................................  1         0       seq
    .................cagccacgguaauuccagcucc........................................................................  2         0       seq
    .................cagccGcgguaauuccagcuccaauau...................................................................  1         1       seq
    ..................agccacgguaauuccagcucc........................................................................  2         0       seq
    ..................agccacgguaauuccagcucA........................................................................  1         1       seq
    ..................agccacgguaauuccagcuccaa......................................................................  1         0       seq
    ...................gccUcgguaauuccagc...........................................................................  1         1       seq
    ...................gccUcgguaauuccagcuc.........................................................................  1         1       seq
    ...................gccacgguaauuccagcuc.........................................................................  1         0       seq
    ...................gccacgguaauuccagcucc........................................................................  6         0       seq
    ...................gcAacgguaauuccagcucca.......................................................................  1         1       seq
```

```
                       Mature                                                    Star
ggaaaguuuggugccaucagccacgguaauuccagcuccaauauuguauauuuaaguuguuguaguuaaaaaauauuguaguuggaucuuggguuguaaaggucaguccac
...................gccUcgguaauuccagcucca.......................................................................   4     1   seq
...................gccUcgguaauuccagcuccaau.....................................................................   1     1   seq
...................gccacgguaauuccagcuccaau.....................................................................   1     0   seq
....................ccUcgguaauuccagcu..........................................................................   1     1   seq
....................ccacgguaauuccagcuA.........................................................................   1     1   seq
....................ccacgguaauuccagcuc.........................................................................   2     0   seq
....................ccUcgguaauuccagcucc........................................................................   2     1   seq
....................ccacgguaauuccagcucc........................................................................   3     0   seq
....................ccacgguaauuccagcucU........................................................................   1     1   seq
....................ccCcgguaauuccagcucc........................................................................   1     1   seq
....................ccUcgguaauuccagcucca.......................................................................   1     1   seq
....................ccacgguaauuccagcucca.......................................................................   7     0   seq
....................ccCcgguaauuccagcucca.......................................................................   2     1   seq
....................ccaAgguaauuccagcuccaa......................................................................   1     1   seq
....................ccacgguaauuccagcuccaa......................................................................   1     0   seq
....................ccCcgguaauuccagcuccaau.....................................................................   1     1   seq
....................ccUcgguaauuccagcuccaau.....................................................................   1     1   seq
....................ccUcgguaauuccagcuccaaua....................................................................   1     1   seq
....................ccGcgguaauuccagcuccaauau...................................................................   3     1   seq
....................ccGcgguaauuccagcuccaauauu..................................................................   1     1   seq
.....................cUcgguaauuccagcucc........................................................................   1     1   seq
.....................cacgguaauuccagcucc........................................................................   1     0   seq
.....................cacgguaauuccagcucU........................................................................   1     1   seq
.....................cacgguaauuccagcucA........................................................................   1     1   seq
.....................cacgguaauCccagcucca.......................................................................   1     1   seq
.....................cCcgguaauuccagcucca.......................................................................   1     1   seq
.....................cUcgguaauuccagcucca.......................................................................   2     1   seq
.....................cUcgguaauuccagcuccaau.....................................................................   1     1   seq
.....................cacgguaauuccagcuccaau.....................................................................   1     0   seq
.....................cacgguaauuccagcuccaaua....................................................................   1     0   seq
.....................cGcgguaauuccagcuccaauau...................................................................   4     1   seq
......................acgguaauuccagcucca.......................................................................   1     0   seq
......................acgguaauuccagcuccC.......................................................................   1     1   seq
......................Ucgguaauuccagcucca.......................................................................   1     1   seq
......................Ccgguaauuccagcucca.......................................................................   1     1   seq
......................Ucgguaauuccagcuccaa......................................................................   1     1   seq
......................acgguaauuccagcuccaau.....................................................................   1     0   seq
......................Ncgguaauuccagcuccaaua....................................................................   1     1   seq
......................acgguaauuccagcuccaauaGu..................................................................   1     1   seq
.......................Agguaauuccagcucca.......................................................................   3     1   seq
.......................cgguaauuccagcCcca.......................................................................   2     1   seq
.......................cgguaauuccagcucca.......................................................................   1283  0   seq
.......................cCguaauuccagcucca.......................................................................   1     1   seq
.......................cgguaauuccUgcucca.......................................................................   1     1   seq
.......................cgguaauuccagcuccC.......................................................................   16    1   seq
.......................cggGaauuccagcucca.......................................................................   1     1   seq
.......................cgguaauuccagcuccU.......................................................................   16    1   seq
.......................cgguaauuccaUcucca.......................................................................   1     1   seq
.......................cgguaauuccagcuUca.......................................................................   2     1   seq
.......................Ngguaauuccagcucca.......................................................................   1     1   seq
.......................cgguaauAccagcucca.......................................................................   1     1   seq
.......................cgguaauuccagUucca.......................................................................   1     1   seq
.......................cgguaauuccGgcucca.......................................................................   6     1   seq
.......................cgguaauuUcagcucca.......................................................................   2     1   seq
.......................cgguaGuuccagcucca.......................................................................   2     1   seq
.......................cggCaauuccagcucca.......................................................................   2     1   seq
.......................cgguaaCuccagcucca.......................................................................   4     1   seq
.......................cgguaauuccagAucca.......................................................................   1     1   seq
.......................Ggguaauuccagcucca.......................................................................   1     1   seq
.......................cgguaauuccagcGcca.......................................................................   4     1   seq
.......................cgguaauuccagcuccG.......................................................................   49    1   seq
.......................cgguGauuccagcucca.......................................................................   2     1   seq
.......................cgguaauuccagcAcca.......................................................................   3     1   seq
.......................cgguaauuccagcuccCa......................................................................   1     1   seq
.......................cgguaGuuccagcuccaa......................................................................   1     1   seq
.......................cCguaauuccagcuccaa......................................................................   1     1   seq
.......................cgguaauuccagcuccGa......................................................................   1     1   seq
.......................cgguaauuccagcuccaa......................................................................   292   0   seq
.......................Ggguaauuccagcuccaa......................................................................   1     1   seq
.......................cggAaauuccagcuccaa......................................................................   1     1   seq
```

Mature **Star**

```
ggaaaguuuggugccaucagccacgguaauuccagcuccaauauuguauauuuaaguuguuguaguuaaaaaauauuguaguuggaucuuggguuguaaaggucaguccac
.......................cgguaauuccagcuccaG......................................................................  7    1  seq
.......................cggGaauuccagcuccaa......................................................................  1    1  seq
.......................cgguaauuGcagcuccaa......................................................................  1    1  seq
.......................cgguaauCccagcuccaa......................................................................  1    1  seq
.......................cgguaauuccagcCccaau.....................................................................  1    1  seq
.......................cgguaauuAcagcuccaau.....................................................................  1    1  seq
.......................cgguaaCuccagcuccaau.....................................................................  2    1  seq
.......................cgguaauuccagcuccaaA.....................................................................  4    1  seq
.......................cgCuaauuccagcuccaau.....................................................................  1    1  seq
.......................cgguaauuccagcuccaau.....................................................................  764  0  seq
.......................Ggguaauuccagcuccaau.....................................................................  3    1  seq
.......................cgguaGuuccagcuccaau.....................................................................  1    1  seq
.......................cgguaauuccagcuccaaC.....................................................................  74   1  seq
.......................cgguaauuccagcuccaGu.....................................................................  1    1  seq
.......................cgguaauuccagcAccaau.....................................................................  2    1  seq
.......................cUguaauuccagcuccaau.....................................................................  1    1  seq
.......................cgguaauuccagUuccaau.....................................................................  1    1  seq
.......................cgguaauuccGgcuccaau.....................................................................  2    1  seq
.......................cggAaauuccagcuccaau.....................................................................  1    1  seq
.......................cgguaauCccagcuccaau.....................................................................  1    1  seq
.......................cgguaauuccagcuUcaau.....................................................................  1    1  seq
.......................cgguaauuccagcuccaaG.....................................................................  10   1  seq
.......................cgguaauuccagcuccGau.....................................................................  1    1  seq
.......................cgguaaCuccagcuccaaua....................................................................  1    1  seq
.......................cgguaauuccagcCccaaua....................................................................  2    1  seq
.......................cgguaauCccagcuccaaua....................................................................  1    1  seq
.......................Ggguaauuccagcuccaaua....................................................................  1    1  seq
.......................cgguaGuuccagcuccaaua....................................................................  1    1  seq
.......................cgguaauuccagcuccaauG....................................................................  6    1  seq
.......................cgguGauuccagcuccaaua....................................................................  1    1  seq
.......................cgguaauuUcagcuccaaua....................................................................  1    1  seq
.......................cCguaauuccagcuccaaua....................................................................  1    1  seq
.......................cgguaauuccagcuccaaCa....................................................................  3    1  seq
.......................cgguaauuccagcuccaaua....................................................................  320  0  seq
.......................cgguaauuccagcuccaauaGu..................................................................  40   1  seq
.......................cgguaauuccagcuccaauaCu..................................................................  1    1  seq
.......................cgguaauuccagcuccaauaGug.................................................................  3    1  seq
.......................cgguaauuccagcuccaauaGugu................................................................  4    1  seq
.......................cgguaauuccagcuccaauaGugua...............................................................  1    1  seq
.......................cgguaauuccagcuccaauaGuguaua.............................................................  1    1  seq
.......................cgguaauuccagcuccaauaGuguauauuu..........................................................  1    1  seq
.......................cgguaauuccagcuccaauaGuguauauuua.........................................................  1    1  seq
........................gguaauuccagcuccaa......................................................................  8    0  seq
........................gguaauuccagcuccaG......................................................................  1    1  seq
........................gguaauuccagcuccaaC.....................................................................  2    1  seq
........................gguaauuccagcuccaau.....................................................................  23   0  seq
........................gguaauuccagcuccaauG....................................................................  1    1  seq
........................gguaauuccagcuccaaua....................................................................  15   0  seq
........................gguaauuccagcuccaauaGu..................................................................  1    1  seq
.........................guaauuccagcuccGau.....................................................................  1    1  seq
.........................guaauuccagcuccaaC.....................................................................  22   1  seq
.........................guaauucAagcuccaau.....................................................................  1    1  seq
.........................guaauuccagcuUcaau.....................................................................  1    1  seq
.........................Nuaauuccagcuccaau.....................................................................  1    1  seq
.........................guaauuccagcuccaaG.....................................................................  1    1  seq
.........................guaauuccagcuccaau.....................................................................  128  0  seq
.........................guaauuccGgcuccaau.....................................................................  1    1  seq
.........................guUauuccagcuccaaua....................................................................  1    1  seq
.........................guaauuccGgcuccaaua....................................................................  1    1  seq
.........................guaauuccagcuccaauG....................................................................  1    1  seq
.........................guaauuccagcuccaaua....................................................................  98   0  seq
..........................uaauuccagcCccaaua....................................................................  1    1  seq
..........................uaauuccagcuccaaua....................................................................  72   0  seq
..........................uaauCccagcuccaaua....................................................................  1    1  seq
..........................Caauuccagcuccaaua....................................................................  1    1  seq
..........................uaauuccagcuccaauG....................................................................  2    1  seq
..........................uaauuccagcuccGaua....................................................................  1    1  seq
..........................uaaCuccagcuccaaua....................................................................  1    1  seq
..........................uaauuccagcuccaauauCg.................................................................  1    1  seq
............................auccagcuccaauaCu...................................................................  1    1  seq
```

```
                          Mature                                             Star
ggaaaguuuggugccaucagccacgguaauuccagcuccaauauuguauauuuaaguuguuguaguuaaaaauauuguaguuggaucuuggguuguaaaggucaguccac
............................auuccagcuccaauauC.................................................................   1     1   seq
............................auuccagcuccaauauCg................................................................   1     1   seq
.............................uuccagcuccaauauCg................................................................   1     1   seq
.............................uuccagcuccaauaGugua..............................................................   1     1   seq
.............................uuccagcuccaauaGuguaua............................................................   1     1   seq
.............................uuccagcuccaauauCguaua............................................................   1     1   seq
.............................uuccagcuccaauaGuguauau...........................................................   1     1   seq
.............................uuccagcuccaauauCguauauu..........................................................   1     1   seq
.............................uuccagcuccaauaGuguauauu..........................................................   1     1   seq
.............................uuccagcuccaauauCguauauuuaaguuguug................................................   1     1   seq
..............................uccagcuccaauaGugua..............................................................   1     1   seq
...............................ccagcuccaauaGuguauau...........................................................   1     1   seq
................................cagcuccaauaGuguaua............................................................   1     1   seq
........................................auauCguauauuuaaguug...................................................   1     1   seq
.............................................guauauuuaaguuguug................................................   14    0   seq
.............................................guauauuuaaguuguuC................................................   1     1   seq
.............................................Uuauauuuaaguuguug................................................   1     1   seq
.............................................guauauuuaaguuguugu...............................................   1     0   seq
..............................................uauauuuaaguuguugu...............................................   1     0   seq
..............................................uauauuuaaguuguugua..............................................   1     0   seq
................................................uauuuaaguuguuguaguu...........................................   1     0   seq
................................................uauuuaaguuguuguaguC...........................................   1     1   seq
..................................................uuuaaguuguugGaguu...........................................   2     1   seq
..................................................uuuaaguuguuguaguuaa.........................................   2     0   seq
......................................................aguuguuguaguuaaaa.......................................   1     0   seq
........................................................................Cauuguaguuggaucuu.....................   1     1   seq
.........................................................................auAguaguuggaucuug....................   1     1   seq
..........................................................................uuguaguuggaucuugg...................   1     0   seq
..........................................................................uuguaguuggaucuugA...................   1     1   seq
..........................................................................Cuguaguuggaucuuggg..................   1     1   seq
..........................................................................uGguaguuggaucuuggg..................   1     1   seq
..........................................................................uuguaguuggaucuugggu.................   1     0   seq
..........................................................................uGguaguuggaucuugggu.................   1     1   seq
...........................................................................uguaguuggaucuuggg..................   2     0   seq
...........................................................................Gguaguuggaucuuggg..................   1     1   seq
...........................................................................uguaguuggaucuuggU..................   1     1   seq
...........................................................................uguaguuggaucuuggA..................   1     1   seq
...........................................................................Gguaguuggaucuugggu.................   2     1   seq
...........................................................................uguaguuggaucuugggu.................   3     0   seq
............................................................................guaguuggaucuugggG.................   30    1   seq
............................................................................guaguuggaucuAgggu.................   1     1   seq
............................................................................guaguuggaucuuggUu.................   2     1   seq
............................................................................Cuaguuggaucuugggu.................   1     1   seq
............................................................................guaguuggaucCugggu.................   1     1   seq
............................................................................guaguuggauUuugggu.................   1     1   seq
............................................................................guaguGggaucuugggu.................   1     1   seq
............................................................................guaguuggaucuuggCu.................   3     1   seq
............................................................................guaguCggaucuugggu.................   3     1   seq
............................................................................guaguuggaucuugggA.................   16    1   seq
............................................................................guaguAggaucuugggu.................   1     1   seq
............................................................................guaguuggaucuugggu.................   879   0   seq
............................................................................Nuaguuggaucuugggu.................   2     1   seq
............................................................................guaguugAaucuugggu.................   2     1   seq
............................................................................guagCuggaucuugggu.................   2     1   seq
............................................................................guaguuggaucuuAggu.................   3     1   seq
............................................................................guaguuggaucuCgggu.................   4     1   seq
............................................................................Uuaguuggaucuugggu.................   1     1   seq
............................................................................guaguCggaucuuggguu................   1     1   seq
............................................................................guaguuggaucuuggUuu................   1     1   seq
............................................................................guaguuggaucuuggguG................   8     1   seq
............................................................................guaguuggaucuuggguu................   135   0   seq
............................................................................guaguuggGucuuggguu................   1     1   seq
............................................................................Uuaguuggaucuuggguu................   1     1   seq
............................................................................guaguuggaucuuggguAg...............   4     1   seq
............................................................................guaguuggaucuuggguug...............   13    0   seq
............................................................................guaguuggaucuuggguugC..............   1     1   seq
............................................................................guaguuggaucuuggguugu..............   1     0   seq
............................................................................guaguuggaucuuggguGgu..............   1     1   seq
............................................................................guaguuggaucuuggguAgu..............   1     1   seq
............................................................................guaguuggaucuuggguugua.............   1     0   seq
```

```
                       Mature                                               Star
ggaaaguuuggugccaucagccacgguaauuccagcuccaauauuguauauuuaaguuguuguaguuaaaaauauuguaguuggaucuuggguuguaaaggucaguccac
............................................................................guaguuggaucuuggguGgua.............    1      1      seq
............................................................................guaguuggaucuuggguAgua.............    2      1      seq
............................................................................guaguuggaucuuggguuguaa............    2      0      seq
............................................................................guaguuggaucuuggguuguaaaggu........    1      0      seq
.............................................................................uGguuggaucuuggguu................    1      1      seq
.............................................................................uaguuggaucuuggguu................    167    0      seq
.............................................................................uaguuggaucGuggguu................    1      1      seq
.............................................................................uaguuggaucuuggAuu................    1      1      seq
.............................................................................uaguuggaucuuAgguu................    2      1      seq
.............................................................................uaguCggaucuuggguu................    2      1      seq
.............................................................................uaguuggaucCuggguu................    2      1      seq
.............................................................................uaguuggaucuuggguG................    9      1      seq
.............................................................................uaguuggaucuugggGu................    1      1      seq
.............................................................................uaguuggGucuuggguu................    1      1      seq
.............................................................................uaguuggaucuuggUuu................    1      1      seq
.............................................................................uaguuggaucuuggguuC...............    2      1      seq
.............................................................................uaguuggaucuuggguAg...............    11     1      seq
.............................................................................uaguuggaucuuggguGg...............    2      1      seq
.............................................................................uaguuggaucuuggguuA...............    3      1      seq
.............................................................................uaguuggaucuugggAug...............    1      1      seq
.............................................................................uaguuggaucuuggguug...............    25     0      seq
.............................................................................uaguuggaucuuggguugu..............    3      0      seq
.............................................................................uaguuggaucuuggguGgu..............    2      1      seq
.............................................................................uaguuggaucuuggguGgua.............    1      1      seq
.............................................................................uaguuggaucuuggguugua.............    5      0      seq
.............................................................................uaguuggGucuuggguugua.............    1      1      seq
.............................................................................uaguuggaucuuggguAgua.............    1      1      seq
.............................................................................uaguuggaucuuggguuguaa............    1      0      seq
.............................................................................uaguuggaucuuggguuguaaG...........    1      1      seq
.............................................................................uaguuggaucuuggguuguaaa...........    1      0      seq
.............................................................................uaguuggaucuuggguuguaaag..........    3      0      seq
.............................................................................uaguuggaucuuggguuguaaagguca......    1      0      seq
..............................................................................aguuggaucuuggguAg...............    1      1      seq
..............................................................................aguuggaucuuggguug...............    4      0      seq
..............................................................................aguuggaucuuggguugu..............    2      0      seq
..............................................................................aguuggaucuuggguugua.............    3      0      seq
..............................................................................aguuggaucuuggguuguaU............    1      1      seq
..............................................................................aguuggaucuuggguuguaaa...........    1      0      seq
..............................................................................aguuggaucuuggguuguaaagg.........    1      0      seq
..............................................................................aguuggaucuuggguuguaaagguc.......    1      0      seq
...............................................................................guuggaucuuggguugu..............    6      0      seq
...............................................................................guuggaucuuggguAgu..............    3      1      seq
...............................................................................guuggaucuuggguuguG.............    1      1      seq
...............................................................................guuggaucuuggguugua.............    17     0      seq
...............................................................................guuggaucuuggguAgua.............    3      1      seq
...............................................................................guuggaucuuggguuguaa............    6      0      seq
...............................................................................guuggaucuuggguGguaa............    1      1      seq
...............................................................................guuggaucuuggguuguaaa...........    3      0      seq
...............................................................................guuggaucuuggguuguaaag..........    1      0      seq
...............................................................................guuggaucuuggguuguaaagg.........    1      0      seq
...............................................................................guuggaucuuggguAguaaagg.........    1      1      seq
...............................................................................guuggaucuuggguGguaaaggu........    1      1      seq
...............................................................................guuggaucuuggguuguaaaggC........    1      1      seq
...............................................................................guuggaucuuggguuguaaaggu........    1      0      seq
...............................................................................guuggaucuuggguuguaaagguc.......    4      0      seq
................................................................................uuggaucuuggguAgua.............    5      1      seq
................................................................................uuggaucuuggguugua.............    11     0      seq
................................................................................uuggaucuuggguugCa.............    1      1      seq
................................................................................uuggaucuuggguuguC.............    1      1      seq
................................................................................uuggaucuuggguuguaa............    4      0      seq
................................................................................uuggaucuuggguGguaa............    1      1      seq
................................................................................uuggaucuuggguuguaaa...........    2      0      seq
................................................................................uuggaucuuggguuguaaag..........    3      0      seq
................................................................................uuggaucuuggguuguaaagg.........    2      0      seq
................................................................................uuggaucuuggguGguaaagg.........    1      1      seq
................................................................................uuggaucuuggguuguaaaggu........    2      0      seq
................................................................................uuggaucuuggguuguaaagguc.......    1      0      seq
.................................................................................uggaucuuggguuguaa............    2      0      seq
.................................................................................uggaucuuggguAguaa............    1      1      seq
.................................................................................uggaucuuggguuguaaa...........    3      0      seq
```

```
                        Mature                                                Star
ggaaaguuuggugccaucagccacgguaauuccagcuccaauauuguauauuuaaguuguuguaguuaaaaaauauuguaguuggaucuuggguuguaaaggucaguccac
..................................................................................uggaucuuggguGguaaa...........     1     1     seq
..................................................................................uggaucuuggguuguaaagg.........     1     0     seq
..................................................................................uggaucuuggguuguaaaggu........     1     0     seq
..................................................................................uggaucuuggguuguaaagguc.......     1     0     seq
.....................................................................................aucuuggguuguaaagg.........     2     0     seq
.....................................................................................aucuuggguuguaaagguc.......     1     0     seq
......................................................................................ucuuggguuguaaaggu........     2     0     seq
......................................................................................ucuuggguGguaaaggu........     1     1     seq
......................................................................................ucuuggguGguaaagguc.......     2     1     seq
......................................................................................ucuuggguuguaaagguc.......     3     0     seq
......................................................................................ucuuggguAguaaagguc.......     1     1     seq
......................................................................................ucuuggguuguaaagguca......     2     0     seq
......................................................................................ucuuggguuguaaaggucC......     1     1     seq
.......................................................................................cuuggguAguaaagguc.......     1     1     seq
........................................................................................uuggguuguaaagguca......     1     0     seq
..............................................................................................uCguaaaggucagucca.     1     1     seq
```

Provisional ID	:	MA_112405_24871
Score total	:	0.7
Score for star read(s)	:	-1.3
Score for read counts	:	0
Score for mfe	:	0.4
Score for randfold	:	1.6
Score for cons. seed	:	
Total read count	:	441
Mature read count	:	441
Loop read count	:	0
Star read count	:	0

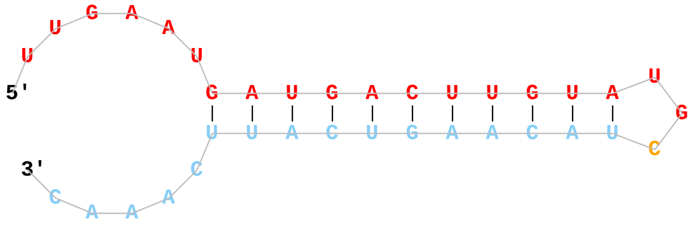

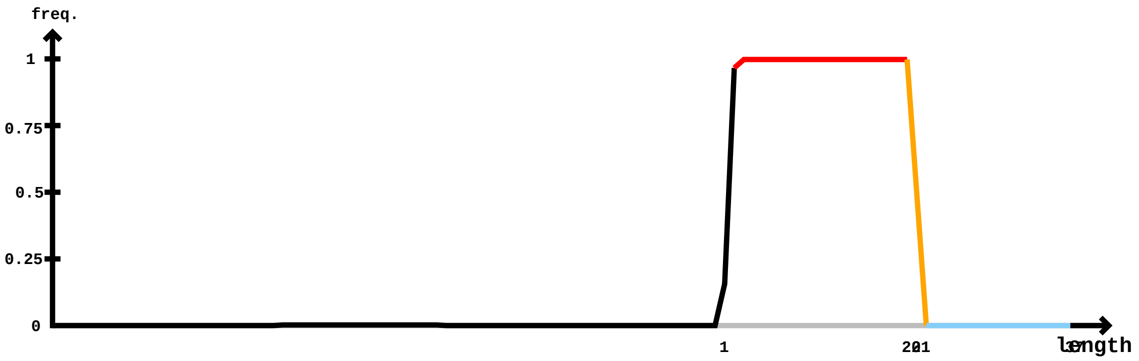

Mature Star

```
5'- agagaaucucaggauaaugccugcuaaugaugaacagauucagaauaaccuccagagaauaugaagaguuugaaugaugacuuguaugcuacaagucauucaaacucu   -3'    exp
    ...((((((((((......))))............))))))......................(((((((((...((((((((((...)))))))))))))))))))   reads     mm        sample
    ......................gcuaaugaugGacagau.....................................................................   1         1         seq
    ....................................................................uCugaaugaugacuuguaug....................   64        1         seq
    ....................................................................uCugaaugaugacuuguaugc...................   5         1         seq
    .....................................................................Gugaaugaugacuuguaug....................   1         1         seq
    .....................................................................Cugaaugaugacuuguaug....................   352       1         seq
    .....................................................................Cugaaugaugacuuguaugc...................   5         1         seq
    ......................................................................ugaaugaugacuuguaug....................   10        0         seq
    ......................................................................ugaaugaugacuuguaugU...................   4         1         seq
```

Provisional ID	:	MA_1131_3887
Score total	:	2.8
Score for star read(s)	:	-1.3
Score for read counts	:	0
Score for mfe	:	2.5
Score for randfold	:	1.6
Score for cons. seed	:	
Total read count	:	122
Mature read count	:	122
Loop read count	:	0
Star read count	:	0

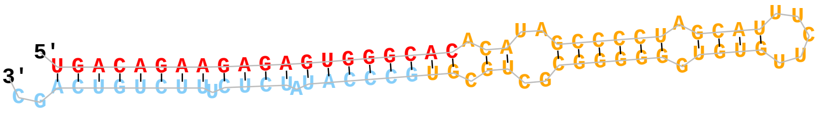

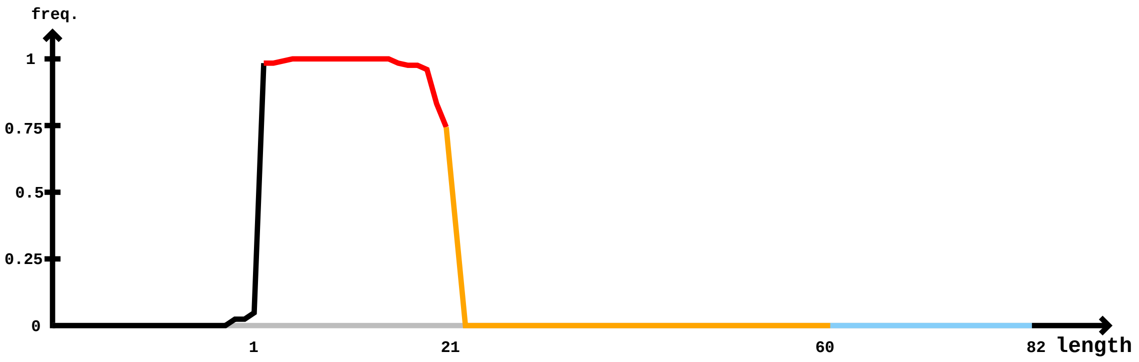

Mature   Star

```
5'- gggcuuguuggguaaggugcugacagaagagagugggcacacauagccccuagcauuucuuguguggggggcgcugcgugcccauaucucuuucugucagcucucaac -3'    exp
    ......((((((......((((((((((((((((((((((.((..((((((.((((.....)))).))))))..)).)))))))).)))).)))))))))).))))))    reads    mm    sample
    .................ugcugacagaagagagu..........................................................................    2    0    seq
    .................ugcugacagaagagagug.........................................................................    1    0    seq
    ...................cugacagaagagagugggc......................................................................    1    0    seq
    ...................cugacagaagagagugggca.....................................................................    1    0    seq
    ...................cugacagaagagagugggcac....................................................................    1    0    seq
    ....................ugacagaagagaguggg.......................................................................    2    0    seq
    ....................ugacagaagagUgugggc......................................................................    1    1    seq
    ....................ugacagaagagagugggU......................................................................    3    1    seq
    ....................ugacagaagagagugggc......................................................................    11    0    seq
    ....................ugacagaagagagugggcG.....................................................................    1    1    seq
    ....................ugacagaagGgagugggca.....................................................................    1    1    seq
    ....................ugacagaagagagugggca.....................................................................    7    0    seq
    ....................uUacagaagagagugggcac....................................................................    1    1    seq
    ....................ugacagaagagagugCgcac....................................................................    1    1    seq
    ....................ugacagaagagaguAggcac....................................................................    1    1    seq
    ....................ugacagaagagagugUgcac....................................................................    2    1    seq
    ....................ugacGgaagagagugggcac....................................................................    1    1    seq
    ....................Cgacagaagagagugggcac....................................................................    1    1    seq
    ....................ugacagaagagagugggcac....................................................................    75    0    seq
    ....................ugacagaagagagugggcaU....................................................................    4    1    seq
    ....................ugacagaagGgagugggcac....................................................................    1    1    seq
    ....................ugacagaagagagugggcaA....................................................................    2    1    seq
    ....................ugacagaagagagugggcacU...................................................................    2    1    seq
    ......................acagaagagagugggca.....................................................................    1    0    seq
    .......................cagaagagagugggcac....................................................................    1    0    seq
```

Provisional ID	:	MA_113136_22822
Score total	:	0
Score for star read(s)	:	3.9
Score for read counts	:	-0.3
Score for mfe	:	-1.4
Score for randfold	:	-2.2
Score for cons. seed	:	
Total read count	:	11
Mature read count	:	10
Loop read count	:	0
Star read count	:	1

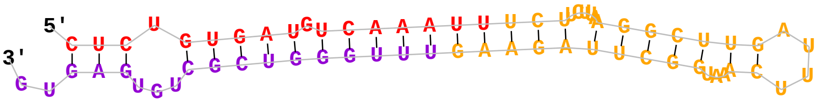

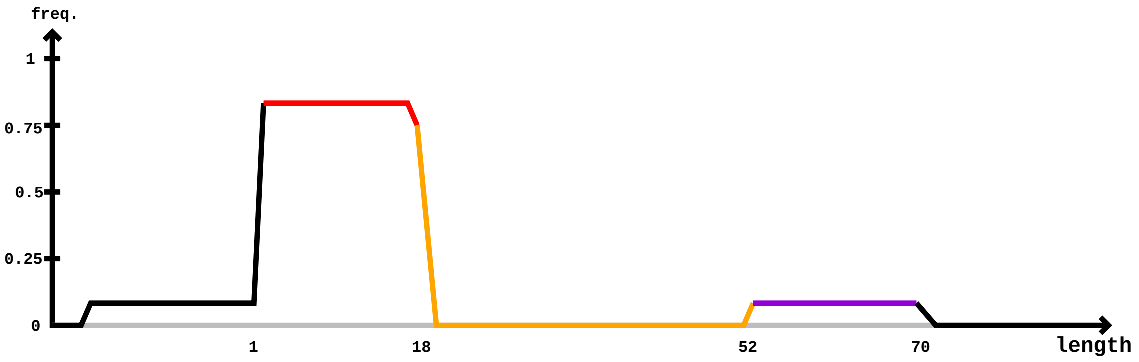


```
                         Mature                                          Star
5'- auauaaugcaaguguccuaacucugugaugucaaauuucuucuuaaggcuugauuucaaauggcuuagaaguuugggucgcugugagugaguauaugaucaucaacau  -3'  obs
    auauaaugcaaguguccuaacucugugaugucaaauuucuucuuaaggcuugauuucaaauggcuuagaaguuugggucgcugugagugaguauaugaucaucaacau       exp
    .....(((...((((.((.((((.(((((.((((((((((.....(((((((....))...))))))))))))))))))))...)))).)).))))...)))......   reads   mm   sample
    ..auaaCgcaaguguccua.........................................................................................   1       1    seq
    ..................acucuAugaugucaaau.........................................................................   1       1    seq
    ....................cucugugaugucaaauu.......................................................................   5       0    seq
    ....................cucugugaugucaaauuu......................................................................   4       0    seq
    .......................................................................uuugggucgcugGgagug...................   1       1    seq
```

Provisional ID	:	MA_1145681_28191
Score total	:	0
Score for star read(s)	:	-1.3
Score for read counts	:	0
Score for mfe	:	-0.3
Score for randfold	:	1.6
Score for cons. seed	:	
Total read count	:	88
Mature read count	:	88
Loop read count	:	0
Star read count	:	0

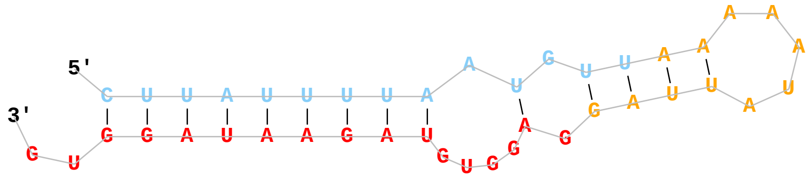

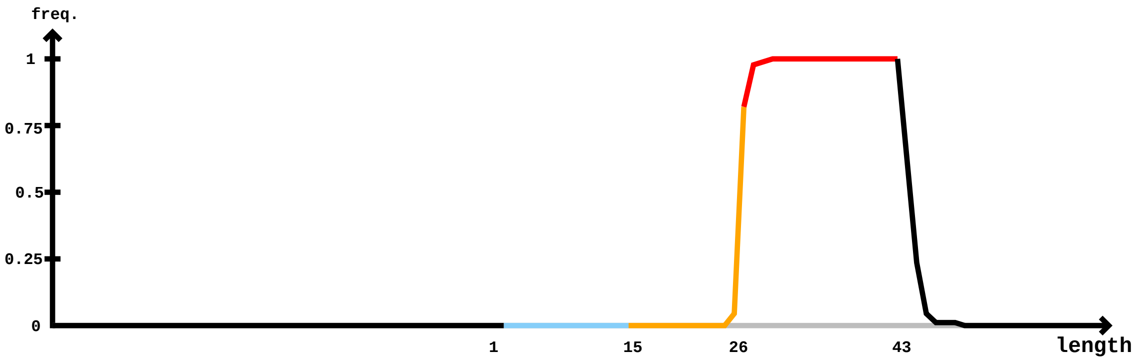

Star Mature

```
5'- gagucgaaaucucaagaggaauagugcaaggcaugcacaggugaacuuauuuuaauguuaaaaauauuaggagguguagaauagguggaggcugagaagucagcucac   -3'    exp
    ((((.((..(((((.........(((((.....)))))......((((((((((.(.((((.....)))).)....))))))))))......)))))..)).))))..   reads    mm    sample
    .....................................................................ggagguguagaauaAgugg....................   3        1     seq
    .....................................................................ggagguguagaauaggugU....................   1        1     seq
    ......................................................................gagguguagaauaggug.....................   36       0     seq
    ......................................................................gagguguUgaauaggug.....................   1        1     seq
    ......................................................................gagguguagaauagguA.....................   2        1     seq
    ......................................................................gagguguagaauagguU.....................   1        1     seq
    ......................................................................gagguguagaauaCgug.....................   2        1     seq
    ......................................................................gagguguagaauaCgugg....................   1        1     seq
    ......................................................................gagguguagaauaggugg....................   12       0     seq
    ......................................................................gagguguagaauaggugA....................   2        1     seq
    ......................................................................gagguguagaauagguggG...................   10       1     seq
    ......................................................................gagguguagaauagguggU...................   1        1     seq
    ......................................................................gagguguagaauagguggGg..................   1        1     seq
    .......................................................................agguguagaauaggugA....................   2        1     seq
    .......................................................................agguguagaauaggugg....................   5        0     seq
    .......................................................................agguguagaauagguggG...................   6        1     seq
    .......................................................................agguguagaauagguggaggcC...............   1        1     seq
    ........................................................................gguguagaauaAguggag..................   1        1     seq
    .........................................................................guguagaauaAguggag..................   1        1     seq
```

Provisional ID	:	MA_11561_10929
Score total	:	0.3
Score for star read(s)	:	-1.3
Score for read counts	:	0
Score for mfe	:	0
Score for randfold	:	1.6
Score for cons. seed	:	
Total read count	:	18296
Mature read count	:	18296
Loop read count	:	0
Star read count	:	0

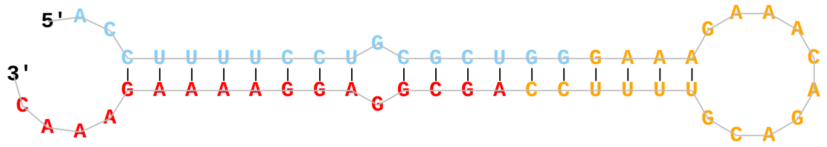

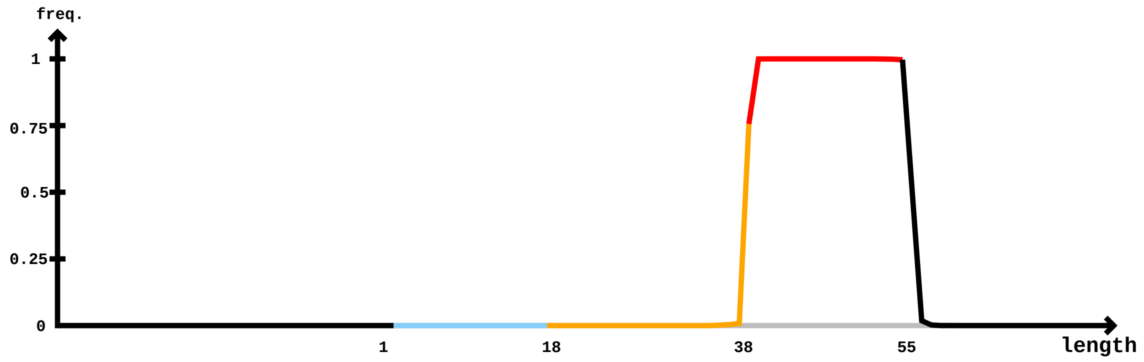

Star Mature

```
5'- aucaaaauccaaaauccgcaauacacugaacaaaccuuuuccugcgcugggaaagaaacagacguuuuccagcggaggaaaagaaacagacguugacacauccuacua   -3'    exp
    .((((..((.........((......)).......((((((((.((((((((((..........)))))))))).)))))))).....))..))))............    reads    mm    sample
    ...................................................................ucAagcggaggaaaaga........................    10    1    seq
    ...................................................................uUcagcggaggaaaaga........................    1    1    seq
    ...................................................................uUcagcggaggaaaagaa.......................    1    1    seq
    ...................................................................ucAagcggaggaaaagaa.......................    5    1    seq
    ...................................................................uUcagcggaggaaaagaaa......................    1    1    seq
    ...................................................................ucAagcggaggaaaagaaa......................    5    1    seq
    ...................................................................ucAagcggaggaaaagaaac.....................    12    1    seq
    ....................................................................cAagcggaggaaaagaa.......................    7    1    seq
    ....................................................................cAagcggaggaaaagaaa......................    13    1    seq
    ....................................................................Ucagcggaggaaaagaaac.....................    1    1    seq
    ....................................................................cAagcggaggaaaagaaac.....................    14    1    seq
    .....................................................................cagcggaggaaaagaaa......................    3    0    seq
    .....................................................................Uagcggaggaaaagaaa......................    7    1    seq
    .....................................................................Nagcggaggaaaagaaa......................    3    1    seq
    .....................................................................Gagcggaggaaaagaaac.....................    20    1    seq
    .....................................................................cagcggaggaaaagaaac.....................    6    0    seq
    .....................................................................Uagcggaggaaaagaaac.....................    6    1    seq
    .....................................................................Nagcggaggaaaagaaac.....................    6    1    seq
    .....................................................................Uagcggaggaaaagaaaca....................    1    1    seq
    .....................................................................cagcggaggaaaagaaacC....................    1    1    seq
    .....................................................................cagcggaggaaaagaaacU....................    21    1    seq
    .....................................................................Gagcggaggaaaagaaaca....................    1    1    seq
    ......................................................................agcggaggaaaagaaCc.....................    3    1    seq
    ......................................................................agcggaggaaaagGaac.....................    20    1    seq
    ......................................................................agcggaggaaaagaaGc.....................    34    1    seq
    ......................................................................agcggaggaaaagCaac.....................    1    1    seq
    ......................................................................agcggUggaaaagaaac.....................    1    1    seq
    ......................................................................Ugcggaggaaaagaaac.....................    4    1    seq
    ......................................................................agcUgaggaaaagaaac.....................    3    1    seq
    ......................................................................agcggaggaaUagaaac.....................    6    1    seq
    ......................................................................agcggaggGaaagaaac.....................    19    1    seq
    ......................................................................agcggaggaaaUgaaac.....................    2    1    seq
    ......................................................................agcggaggCaaagaaac.....................    1    1    seq
    ......................................................................agcggaggaaaagaaac.....................    6946    0    seq
```

Star   Mature

```
aucaaaauccaaaauccgcaauacacugaacaaaccuuuuccugcgcugggaaagaaacagacguuuuccagcggaggaaaagaaacagacguugacacauccuacua
......................................................................agcggaggaaaagaGac.....................  30    1  seq
......................................................................agcggaggUaaagaaac.....................  5     1  seq
......................................................................agcggagAaaaagaaac.....................  2     1  seq
......................................................................agcggaggaaaCgaaac.....................  1     1  seq
......................................................................agcggaggaaaagUaac.....................  1     1  seq
......................................................................Ngcggaggaaaagaaac.....................  10    1  seq
......................................................................agcggCggaaaagaaac.....................  1     1  seq
......................................................................agcggaggaUaagaaac.....................  1     1  seq
......................................................................agcggagUaaaagaaac.....................  1     1  seq
......................................................................agcggaCgaaaagaaac.....................  1     1  seq
......................................................................agcggaggaaGagaaac.....................  28    1  seq
......................................................................Cgcggaggaaaagaaac.....................  7     1  seq
......................................................................agcggaggaaaagaCac.....................  1     1  seq
......................................................................agcCgaggaaaagaaac.....................  1     1  seq
......................................................................Ggcggaggaaaagaaac.....................  34    1  seq
......................................................................aCcggaggaaaagaaac.....................  1     1  seq
......................................................................agcgUaggaaaagaaac.....................  3     1  seq
......................................................................agcggaggaaaagaaaca....................  377   0  seq
......................................................................agcAgaggaaaagaaaca....................  1     1  seq
......................................................................agcggaggGaaagaaaca....................  1     1  seq
......................................................................agcggaAgaaaagaaaca....................  1     1  seq
......................................................................agcggaggaaaagGaaca....................  4     1  seq
......................................................................Ggcggaggaaaagaaaca....................  3     1  seq
......................................................................agcggaggaaaagaaUca....................  1     1  seq
......................................................................agcggUggaaaagaaaca....................  1     1  seq
......................................................................agcggaggaaaagaaacC....................  5314  1  seq
......................................................................agcggaggaaaagaaGca....................  3     1  seq
......................................................................agcggaggaaaagaGaca....................  6     1  seq
......................................................................agcggGggaaaagaaaca....................  2     1  seq
......................................................................agcggaggaaaagaaacG....................  641   1  seq
......................................................................agcggaggaaaagaaCca....................  3     1  seq
......................................................................Ngcggaggaaaagaaaca....................  2     1  seq
......................................................................agcggaggaaGagaaaca....................  3     1  seq
......................................................................agcggaggaaaagaaacUg...................  150   1  seq
......................................................................agcggaggaaaagaaacag...................  1     0  seq
......................................................................agcggaggaaaagaaacCg...................  1     1  seq
......................................................................agcggaggaaaagaaacaU...................  8     1  seq
......................................................................agcggaggaaaagaaacUga..................  17    1  seq
......................................................................agcggaggaaaagaaacaUa..................  2     1  seq
......................................................................agcggaggaaaagaaacagC..................  1     1  seq
......................................................................agcggaggaaaagaaacaUac.................  1     1  seq
.......................................................................gcggaggaaaagaaaca....................  248   0  seq
.......................................................................gcggaggaaaagGaaca....................  2     1  seq
.......................................................................gcggaggaGaagaaaca....................  1     1  seq
.......................................................................gcggaggaaaagaaGca....................  4     1  seq
.......................................................................gcggaggaaaagaaCca....................  2     1  seq
.......................................................................gcggaggaaaagaaacC....................  3651  1  seq
.......................................................................gcggaggGaaagaaaca....................  2     1  seq
.......................................................................gcggaggaaaagaGaca....................  1     1  seq
.......................................................................Acggaggaaaagaaaca....................  1     1  seq
.......................................................................gcggaggaaaCgaaaca....................  1     1  seq
.......................................................................gcggaggaaaagaaacG....................  404   1  seq
.......................................................................gcggGggaaaagaaaca....................  1     1  seq
.......................................................................gcggagAaaaagaaaca....................  1     1  seq
.......................................................................gcggaggaaaagaaacaU...................  6     1  seq
.......................................................................gcggaggaaaagaaacag...................  1     0  seq
.......................................................................gcggaggaaaagaaacUg...................  127   1  seq
.......................................................................gcggaggaaaagaaacaUa..................  5     1  seq
.......................................................................gcggaggaaaagaaacUga..................  10    1  seq
.......................................................................gcggaggaaaagaaacagGcg................  1     1  seq
........................................................................cggaggaaaagaaacag...................  1     0  seq
........................................................................cggaggaaaagaaacUga..................  2     1  seq
.........................................................................ggaggaaaagaaacUga..................  2     1  seq
```

Provisional ID	:	MA_115738_10604
Score total	:	0.9
Score for star read(s)	:	-1.3
Score for read counts	:	0
Score for mfe	:	0.6
Score for randfold	:	1.6
Score for cons. seed	:	
Total read count	:	40
Mature read count	:	40
Loop read count	:	0
Star read count	:	0

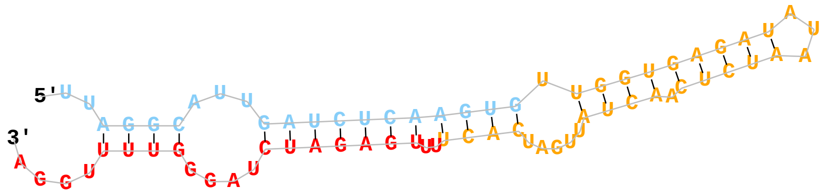

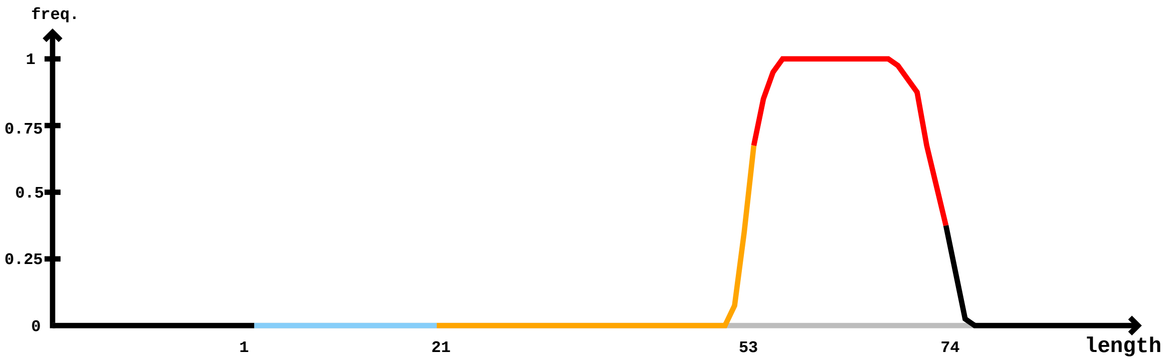

Star Mature

```
5'- uagugaauuaaauuaaaucuuaggcauugaucucaaguguuggugagauauaaucucaacuauugaucacuuuugagaucuaggguuuuggauuuaggguaaacucaacau -3'  exp
    ...(((.(((..((((((((.((((...(((((((((((.(((((((((...))))).)))).....))))..))))))))....)))).))))))))..)))..)))....  reads  mm  sample
    .....................................................................Guuuugagaucuagggu.........................  1  1  seq
    .....................................................................Guuuugagaucuaggguu........................  1  1  seq
    .....................................................................Guuuugagaucuaggguuuu......................  1  1  seq
    ......................................................................uuuugagaucuagggCu........................  1  1  seq
    ......................................................................uuuugagaucuaggguuuu......................  2  0  seq
    ......................................................................uuuugagaucuaggguuuug.....................  1  0  seq
    ......................................................................uuAugagaucuaggguuuugg....................  1  1  seq
    ......................................................................uuuugagaucuaggguuuugg....................  4  0  seq
    ......................................................................uuuugagaucuaggguuuuggU...................  2  1  seq
    .......................................................................uuugGgaucuaggguuu.......................  1  1  seq
    .......................................................................uuugagaucuaggguuu.......................  1  0  seq
    .......................................................................uuugagaucuaggguuuu......................  1  0  seq
    .......................................................................uuugagaucuaggguuuug.....................  2  0  seq
    .......................................................................uuugagaucuaggguuuuggC...................  2  1  seq
    .......................................................................uuugagaucuaggguuuuggU...................  6  1  seq
    ........................................................................uugagaucuaggguuuu......................  3  0  seq
    ........................................................................uugagaucuaggguuuC......................  1  1  seq
    ........................................................................uugagaucuaggguuuug.....................  2  0  seq
    ........................................................................uugagaucuaggguuuuggU...................  1  1  seq
    .........................................................................ugagaucuaggguuuug.....................  1  0  seq
    .........................................................................ugagaucuaggguuuuggU...................  2  1  seq
    .........................................................................ugagaucuaggguuuuggauC.................  1  1  seq
    ..........................................................................gagaucuaggguuuugg....................  1  0  seq
    ..........................................................................gagaucuaggguuuuggU...................  1  1  seq
```

Provisional ID	:	MA_11587_1958
Score total	:	1.6
Score for star read(s)	:	-1.3
Score for read counts	:	0
Score for mfe	:	1.3
Score for randfold	:	1.6
Score for cons. seed	:	
Total read count	:	91136
Mature read count	:	91136
Loop read count	:	0
Star read count	:	0

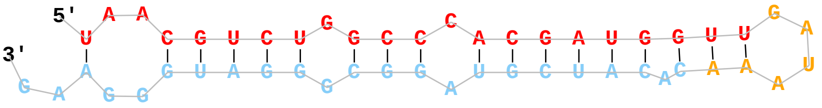

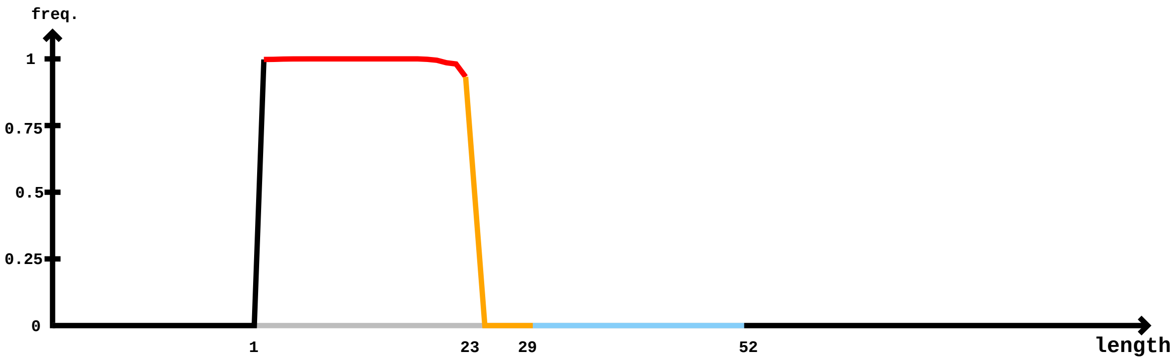

Mature Star

```
5'- auggcgggcaaauguguuuuuaacgucuggcccacgaugguugauaaacacaucguaggcgggaugggaagucgaugcagagcagaccguagaggaggaaagagcagaagga  -3'   exp
    ...(((((...((((((((..(((((.(((.((((((((....))).))))))).))).)))))..)))..))))))..))...))))......................   reads   mm   sample
    ...................uuaacgucuggGccacgaugg........................................................................   1       1    seq
    ...................uuaacgucuggGccacgauggu.......................................................................   1       1    seq
    ....................uaacgucuggGccacga...........................................................................   149     1    seq
    ....................uUacgucuggcccacga...........................................................................   1       1    seq
    ....................uaacgucuggGccacgau..........................................................................   312     1    seq
    ....................uaacgucuggUccacgau..........................................................................   1       1    seq
    ....................uaacgucuggAccacgau..........................................................................   3       1    seq
    ....................uaacgucuggGccacgaug.........................................................................   836     1    seq
    ....................uaacgucuggGccacgaugg........................................................................   418     1    seq
    ....................uGacgucuggcccacgaugg........................................................................   1       1    seq
    ....................uaacgucuggcccacgauggu.......................................................................   1       0    seq
    ....................uUacgucuggcccacgauggu.......................................................................   4       1    seq
    ....................uaacgucuggAccacgauggu.......................................................................   5       1    seq
    ....................uaacgucuggGccacgauggu.......................................................................   4375    1    seq
    ....................uaacgucuggAccacgaugguu......................................................................   101     1    seq
    ....................uaacgucuggcccacgaugguu......................................................................   19      0    seq
    ....................uaacgucuggcccacgaugguC......................................................................   4       1    seq
    ....................uaacgucuggGccacgaugguu......................................................................   84554   1    seq
    ....................uUacgucuggcccacgaugguu......................................................................   53      1    seq
    ....................uaacgucuggUccacgaugguu......................................................................   16      1    seq
    ....................uCacgucuggcccacgaugguu......................................................................   6       1    seq
    ....................uaacgucuggcccacgGugguu......................................................................   1       1    seq
    ....................uGacgucuggcccacgaugguu......................................................................   36      1    seq
    ....................uaacgucuggGccacgaugguug.....................................................................   36      1    seq
    ....................uaacgucuggcccacgaugguuU.....................................................................   1       1    seq
    ....................uaacgucuggGccacgaugguuga....................................................................   1       1    seq
    ....................uaacgucuggGccacgaugguugau...................................................................   1       1    seq
    .....................aacgucuggGccacgau..........................................................................   1       1    seq
    .....................aacgucuggGccacgaug.........................................................................   3       1    seq
    .....................aacgucuggGccacgaugg........................................................................   3       1    seq
    .....................aacgucuggGccacgauggu.......................................................................   3       1    seq
    .....................aacgucuggGccacgaugguu......................................................................   21      1    seq
    ......................acgucuggGccacgaug.........................................................................   12      1    seq
    ......................acgucuggGccacgaugg........................................................................   3       1    seq
```

Mature Star

```
auggcgggcaaauguguuuuuaacgucuggcccacgauggguugauaaacacaucguaggcgggaugggaagucgaugcagagcagaccguagaggaggaaagagcagaagga
......................acgucuggGccacgauggu.......................................................................   6    1   seq
......................acgucuggGccacgaugguu......................................................................   76   1   seq
......................acAucuggcccacgaugguu......................................................................   2    1   seq
......................acgucuggAccacgaugguu......................................................................   6    1   seq
......................acgucuggGccacgaugguug.....................................................................   1    1   seq
.......................cgucuggAccacgaugg........................................................................   1    1   seq
.......................cgucuggcccacgauggu.......................................................................   1    0   seq
.......................cgucuggAccacgaugguu......................................................................   1    1   seq
.......................cgucuggGccacgaugguu......................................................................   44   1   seq
.......................cAucuggcccacgaugguu......................................................................   1    1   seq
........................Aucuggcccacgaugguu......................................................................   1    1   seq
.........................Ccuggcccacgaugguu......................................................................   1    1   seq
.........................ucuggcccacgaugguu......................................................................   7    0   seq
.........................ucuggcccacgauggu C......................................................................   2    1   seq
.........................ucuggcccacgGugguu......................................................................   1    1   seq
.........................ucuggAccacgaugguug.....................................................................   2    1   seq
```

Provisional ID	:	MA_1161878_30608
Score total	:	1.6
Score for star read(s)	:	-1.3
Score for read counts	:	0
Score for mfe	:	1.3
Score for randfold	:	1.6
Score for cons. seed	:	
Total read count	:	1498
Mature read count	:	1497
Loop read count	:	1
Star read count	:	0

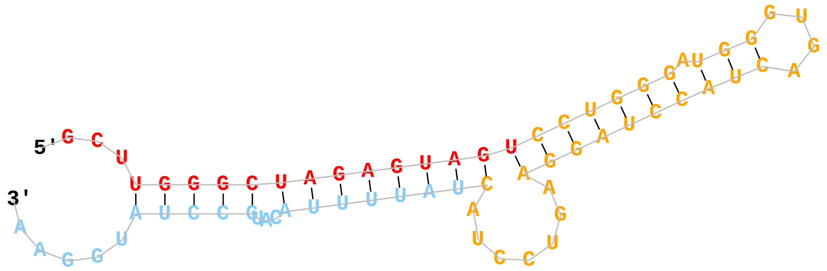

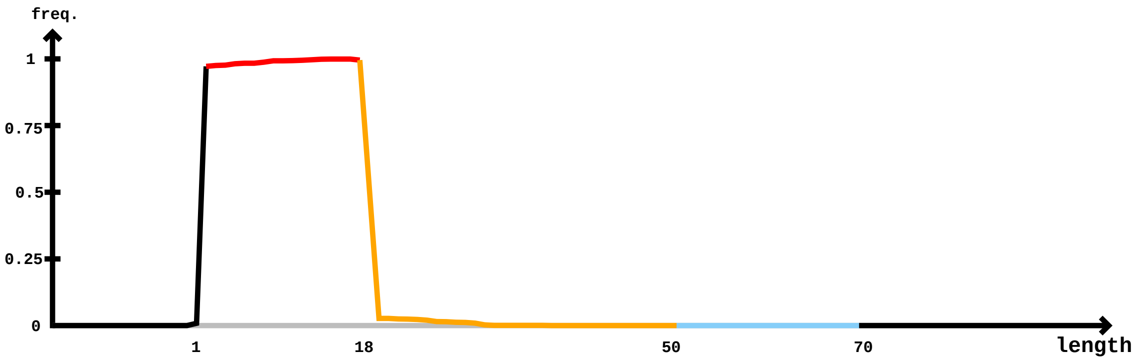

Mature **Star**

```
5'- uccauaguuaguuugcuugggcuagaguaguccugggaugggugacuaccuaggaaguccuacuauuuuacaugccuauggaauuguauuaacuuggacuaauuuauc  -3'   exp
    ((((.((((((((.(.((((((((((((((((((((.(((....))))))))))).......))))))))...))))).).))....)))))))))))).........  reads      mm        sample
    .............ugcuugggcuagaguag..............................................................................  3          0         seq
    .............Ngcuugggcuagaguag..............................................................................  3          1         seq
    .............ugcuugggcuagaguagu.............................................................................  1          0         seq
    .............Ngcuugggcuagaguagu.............................................................................  5          1         seq
    .............Cgcuugggcuagaguaguc............................................................................  1          1         seq
    ..............gcuugggcuagagGagu.............................................................................  1          1         seq
    ..............gcuugggcuagaguagu.............................................................................  1465       0         seq
    ..............Ncuugggcuagaguagu.............................................................................  5          1         seq
    ..............gcuugggcuagaguaUuc............................................................................  1          1         seq
    ..............gcuugggcuagaguaguc............................................................................  2          0         seq
    ..............gcuugggcuagaguaguccu..........................................................................  1          0         seq
    ..............gcuugggcuagaguaguccuggg.......................................................................  1          0         seq
    ...............cuugggcuagaguagAc............................................................................  1          1         seq
    ...............cuugggcuagaguaguc............................................................................  3          0         seq
    ...............cuugggcuagaguaguccuggga......................................................................  1          0         seq
    ................uugggcuagaguaguccugggaug....................................................................  1          0         seq
    ................uugggcuagaguaguccugggaugggu.................................................................  1          0         seq
    .................ugggcuagaguaguccC..........................................................................  2          1         seq
    .................ugggcuagaguaguccugg........................................................................  1          0         seq
    .................ugggcuagaguaguccuggg.......................................................................  2          0         seq
    .................ugggcuagaguaguccugggau.....................................................................  1          0         seq
    .................ugggcuagaguaguccugggauggg..................................................................  1          0         seq
    .................ugggcuagGguaguccugggaugggug................................................................  1          1         seq
    ..................gggcuagaguaguccGg.........................................................................  1          1         seq
    ..................gggcuagaguaguccugg........................................................................  1          0         seq
    ..................gggcuagaguaguccugggaugggC.................................................................  1          1         seq
    ....................gcuagaguaguccuggg.......................................................................  1          0         seq
    ....................gcuagaguaguccuggga......................................................................  4          0         seq
    ....................gcuagaguaguccugggaugg...................................................................  1          0         seq
    .....................cuagaguaguccuggga......................................................................  3          0         seq
    .....................cuagaguaguccugggauA....................................................................  1          1         seq
    .....................cuagaguaguccugggaug....................................................................  1          0         seq
    .....................cuagaguaguccugggauggC..................................................................  1          1         seq
    .....................cuagaguaguccugggauggg..................................................................  1          0         seq
```

Mature **Star**

```
uccauaguuaguuugcuugggcuagaguaguccugggaugggugacuaccuaggaaguccuacuauuuuacaugccuauggaauuguauuaacuuggacuaauuuauc
.....................cuagaguaguccugggaugggu.................................................................	1	0	seq
.......................agaguaguccugggaugggu.................................................................	1	0	seq
........................gaguaguccugggauggg..................................................................	1	0	seq
........................gaguaguccugggaugggu.................................................................	1	0	seq
.........................aguaguccugggaugggu.................................................................	3	0	seq
..........................guaguccugggaugggu.................................................................	2	0	seq
..........................guaguccugggaugggug................................................................	1	0	seq
...........................uaguccugggaugggug................................................................	1	0	seq
................................cugggaugggugacuacc..........................................................	1	0	seq
```

Provisional ID	:	MA_1168899_37313
Score total	:	6304.4
Score for star read(s)	:	3.9
Score for read counts	:	6302.6
Score for mfe	:	0.1
Score for randfold	:	-2.2
Score for cons. seed	:	
Total read count	:	12374
Mature read count	:	11608
Loop read count	:	162
Star read count	:	604

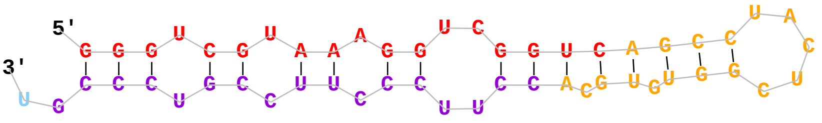

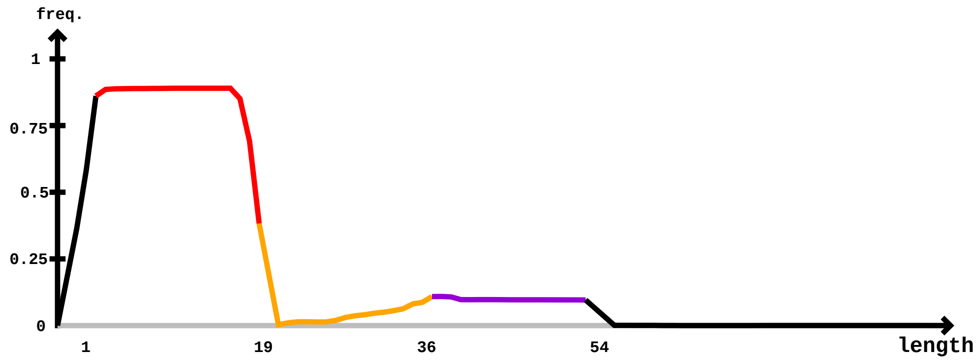

Mature **Star**

```
5'- uugggucguaaaggucggucagccuacucggugugcaccuucccuuccgucccguuugcugguggcaugcuucuggccuuaauuggcuggg   -3'   obs
    uugggucguaaaggucggucagccuacucggugugcaccuucccuuccgucccguuugcugguggcaugcuucuggccuuaauuggcuggg         exp
    ..(((.((.((.((..((((((((.....))).)).)))..)).)).)).)))...(((.....))).....(..(((......)))..).   reads   mm   sample
    uugggucgCaaaggucg..........................................................................   2       1    seq
    uugggucguaaUggucg..........................................................................   1       1    seq
    uGgggucguaaaggucg..........................................................................   1       1    seq
    Cugggucguaaaggucg..........................................................................   1       1    seq
    uugggucguaGaggucg..........................................................................   1       1    seq
    uugggucguaaGggucg..........................................................................   1       1    seq
    uAgggucguaaaggucg..........................................................................   1       1    seq
    Augggucguaaaggucg..........................................................................   1       1    seq
    uugggucguaUaggucg..........................................................................   1       1    seq
    uugggucguaaaggCcg..........................................................................   2       1    seq
    uugggucguaaaggucg..........................................................................   490     0    seq
    uugggucgGaaaggucg..........................................................................   1       1    seq
    uugggCcguaaaggucg..........................................................................   2       1    seq
    uugggucCuaaaggucg..........................................................................   1       1    seq
    uugggucguaaaggGcg..........................................................................   2       1    seq
    uugggucguaaGggucgg.........................................................................   4       1    seq
    uugggucguaaaggAcgg.........................................................................   1       1    seq
    uuAggucguaaaggucgg.........................................................................   4       1    seq
    uugggucguaaagguAgg.........................................................................   1       1    seq
    uugggucguaaagguGgg.........................................................................   2       1    seq
    Gugggucguaaaggucgg.........................................................................   2       1    seq
    uugggucgCaaaggucgg.........................................................................   4       1    seq
    uugggucguaaaggucCg.........................................................................   2       1    seq
    uugggucguaaaggCcgg.........................................................................   6       1    seq
    uCgggucguaaaggucgg.........................................................................   1       1    seq
    uugggucguaaaggucgg.........................................................................   1123    0    seq
    uugggucguaGaggucgg.........................................................................   5       1    seq
    uugggucguGaaggucgg.........................................................................   1       1    seq
    Cugggucguaaaggucgg.........................................................................   3       1    seq
    uugggucguaaaggGcgg.........................................................................   2       1    seq
    uuggUucguaaaggucgg.........................................................................   1       1    seq
    uugggucguaaaggucgC.........................................................................   7       1    seq
    uugggucguGaaggucggu........................................................................   6       1    seq
```

```
Mature                     Star
uugggucguaaaggucggucagccuacucggugugcaccuucccuuccgucccguuugcugguggcaugcuucuggccuuaauuggcuggg
uugggucguaaaggucggC........................................................................   255    1   seq
uugggucguaaaAgucggu........................................................................   5      1   seq
Augggucguaaaggucggu........................................................................   1      1   seq
uugggucguaaagguGggu........................................................................   1      1   seq
uugggucguaaaUgucggu........................................................................   1      1   seq
uugggucguaGaggucggu........................................................................   5      1   seq
uugggucguaaGggucggu........................................................................   5      1   seq
uugggCcguaaaggucggu........................................................................   6      1   seq
uugggAcguaaaggucggu........................................................................   1      1   seq
uugggucguaaaggAcggu........................................................................   5      1   seq
uuCggucguaaaggucggu........................................................................   1      1   seq
uugggucguaaaggucggu........................................................................   1438   0   seq
uugggucguaCaggucggu........................................................................   1      1   seq
uugggucguaaaggucggG........................................................................   41     1   seq
Gugggucguaaaggucggu........................................................................   3      1   seq
uugggucguaaaggucgCu........................................................................   4      1   seq
uugggucguaUaggucggu........................................................................   2      1   seq
uugggGcguaaaggucggu........................................................................   1      1   seq
uuAggucguaaaggucggu........................................................................   1      1   seq
uuggUucguaaaggucggu........................................................................   1      1   seq
Cugggucguaaaggucggu........................................................................   3      1   seq
uAgggucguaaaggucggu........................................................................   1      1   seq
uugggucguaaaggCcggu........................................................................   5      1   seq
uCgggucguaaaggucggu........................................................................   4      1   seq
uugggucguaaaggucggA........................................................................   19     1   seq
uugggucgCaaaggucggu........................................................................   2      1   seq
Gugggucguaaaggucgguc.......................................................................   1      1   seq
uugggucguaaaggAcgguc.......................................................................   2      1   seq
uugggucguaaaggCcgguc.......................................................................   3      1   seq
uugggucguaaaggucggGc.......................................................................   4      1   seq
uugggucguaaGggucgguc.......................................................................   6      1   seq
uugggucguaaaggucggCc.......................................................................   10     1   seq
uCgggucguaaaggucgguc.......................................................................   2      1   seq
uugggucguaaaggucgguG.......................................................................   5      1   seq
uugggAcguaaaggucgguc.......................................................................   2      1   seq
uugggucguaaaggucCguc.......................................................................   1      1   seq
uugggucguaaaggucgguA.......................................................................   23     1   seq
uugggucguaaaggGcgguc.......................................................................   1      1   seq
uugggCcguaaaggucgguc.......................................................................   5      1   seq
Nugggucguaaaggucgguc.......................................................................   2      1   seq
uugUgucguaaaggucgguc.......................................................................   1      1   seq
uugggucguaaaggucgguU.......................................................................   74     1   seq
Cugggucguaaaggucgguc.......................................................................   6      1   seq
uugggucguaaagAucgguc.......................................................................   1      1   seq
uugggucgCaaaggucgguc.......................................................................   4      1   seq
uuggUucguaaaggucgguc.......................................................................   1      1   seq
uuAggucguaaaggucgguc.......................................................................   1      1   seq
uugggucguGaaggucgguc.......................................................................   1      1   seq
uugggucguaaaggucgguc.......................................................................   1094   0   seq
uugggucguaGaggucgguc.......................................................................   1      1   seq
uugggucguaaaggucgguca......................................................................   4      0   seq
Cugggucguaaaggucgguca......................................................................   2      1   seq
.Cgggucguaaaggucgg.........................................................................   5      1   seq
.ugggucgCaaaggucgg.........................................................................   1      1   seq
.ugggucguaCaggucgg.........................................................................   1      1   seq
.ugggucguaaaggCcgg.........................................................................   4      1   seq
.ugggucguGaaggucgg.........................................................................   1      1   seq
.ugggAcguaaaggucgg.........................................................................   1      1   seq
.ugggucguaaagguAgg.........................................................................   1      1   seq
.Ngggucguaaaggucgg.........................................................................   1      1   seq
.Agggucguaaaggucgg.........................................................................   1      1   seq
.ugggucguaaagUucgg.........................................................................   1      1   seq
.ugggucguaaaggucCg.........................................................................   1      1   seq
.ugggucguaaaggucgC.........................................................................   5      1   seq
.ugggucguaGaggucgg.........................................................................   1      1   seq
.Ggggucguaaaggucgg.........................................................................   1      1   seq
.ugggucguaaagAucgg.........................................................................   2      1   seq
.ugggucguaaaggucgg.........................................................................   886    0   seq
.ugggCcguaaaggucgg.........................................................................   1      1   seq
.ugggucguaaGggucgg.........................................................................   6      1   seq
```

```
  Mature                             Star
uugggucguaaaggucggucagccuacucggugugcaccuucccuuccgucccguuugcugguggcaugcuucuggccuuaauuggcuggg
.ugggucguaaaggAcgg.........................................................................    1      1      seq
.ugggucguaaaggucggu........................................................................    679    0      seq
.ugggucguaaaggucggC........................................................................    92     1      seq
.ugggucguaaaggucggG........................................................................    18     1      seq
.ugggucgCaaaggucggu........................................................................    1      1      seq
.ugggucguaaaggucggA........................................................................    5      1      seq
.Cgggucguaaaggucggu........................................................................    1      1      seq
.uAggucguaaaggucggu........................................................................    1      1      seq
.uggguGguaaaggucggu........................................................................    1      1      seq
.Agggucguaaaggucggu........................................................................    2      1      seq
.ugggucguaaaggGcggu........................................................................    1      1      seq
.ugggucguaGaggucggu........................................................................    1      1      seq
.ugggCcguaaaggucggu........................................................................    6      1      seq
.ugggucguaaaUgucggu........................................................................    1      1      seq
.Ngggucguaaaggucggu........................................................................    1      1      seq
.uggUucguaaaggucggu........................................................................    1      1      seq
.ugggucguaaGggucggu........................................................................    2      1      seq
.ugggucguaaaggAcggu........................................................................    1      1      seq
.ugggCcguaaaggucgguc.......................................................................    1      1      seq
.Agggucguaaaggucgguc.......................................................................    1      1      seq
.ugggAcguaaaggucgguc.......................................................................    1      1      seq
.uAggucguaaaggucgguc.......................................................................    2      1      seq
.ugggucguaaaggucgguG.......................................................................    5      1      seq
.ugggucguaaaggCcgguc.......................................................................    4      1      seq
.ugggucguaaGggucgguc.......................................................................    5      1      seq
.ugggucguaaaggucgguU.......................................................................    66     1      seq
.Cgggucguaaaggucgguc.......................................................................    5      1      seq
.ugggucguaaaggucggCc.......................................................................    12     1      seq
.ugggucguaGaggucgguc.......................................................................    1      1      seq
.ugggucguaaUggucgguc.......................................................................    1      1      seq
.ugggucguaaaggucgguA.......................................................................    24     1      seq
.ugggucguaaaggucggGc.......................................................................    3      1      seq
.ugggucguaaaggucgguc.......................................................................    1007   0      seq
.ugggucguGaaggucgguc.......................................................................    5      1      seq
.ugggucguaaagAucgguc.......................................................................    1      1      seq
.Ggggucguaaaggucgguc.......................................................................    2      1      seq
.ugggucguaaaggucgguca......................................................................    1      0      seq
..gggucguaaaggucgCu........................................................................    2      1      seq
..gggucguaaaggucggA........................................................................    11     1      seq
..gggucguaaaggucggC........................................................................    156    1      seq
..ggguGguaaaggucggu........................................................................    1      1      seq
..gggucguaaUggucggu........................................................................    1      1      seq
..gggucguaaaggGcggu........................................................................    3      1      seq
..Nggucguaaaggucggu........................................................................    2      1      seq
..gggucguaaaCgucggu........................................................................    1      1      seq
..gggucguaaGggucggu........................................................................    5      1      seq
..gggucguaaaAgucggu........................................................................    2      1      seq
..gggucguaaaggCcggu........................................................................    4      1      seq
..gggucguaaaggAcggu........................................................................    3      1      seq
..gggucgCaaaggucggu........................................................................    2      1      seq
..Aggucguaaaggucggu........................................................................    2      1      seq
..gggucguaaaggucggG........................................................................    30     1      seq
..gggucguaaagAucggu........................................................................    1      1      seq
..gggucguGaaggucggu........................................................................    2      1      seq
..gggucguaGaggucggu........................................................................    5      1      seq
..gggucguaaaggucggu........................................................................    1159   0      seq
..gUgucguaaaggucggu........................................................................    1      1      seq
..gggCcguaaaggucggu........................................................................    4      1      seq
..gggucguaUaggucgguc.......................................................................    2      1      seq
..gggucgCaaaggucgguc.......................................................................    1      1      seq
..gggucguGaaggucgguc.......................................................................    3      1      seq
..gggAcguaaaggucgguc.......................................................................    1      1      seq
..gggCcguaaaggucgguc.......................................................................    5      1      seq
..Cggucguaaaggucgguc.......................................................................    1      1      seq
..gggucguaaaggucggCc.......................................................................    30     1      seq
..gggucguaaaggCcgguc.......................................................................    4      1      seq
..gggucguaaaggucggGc.......................................................................    7      1      seq
..gggucguaGaggucgguc.......................................................................    4      1      seq
..gggucguaaagAucgguc.......................................................................    2      1      seq
..gggucguaaagguGgguc.......................................................................    1      1      seq
```

Mature **Star**

```
uugggucguaaaggucggucagccuacucggugugcaccuucccuuccgucccguuugcugguggcaugcuucuggccuuaauuggcuggg
..Aggucguaaaggucgguc.......................................................................   4     1   seq
..gggucguaaaAgucgguc.......................................................................   2     1   seq
..gggucguaaagguAgguc.......................................................................   1     1   seq
..gggucguaaaggucgguc.......................................................................   1943  0   seq
..gggucguaaaggucgCuc.......................................................................   1     1   seq
..gggucguaaaggucgguA.......................................................................   28    1   seq
..gggucguaaGggucgguc.......................................................................   16    1   seq
..gggucguaaaggucgguG.......................................................................   9     1   seq
..gCgucguaaaggucgguc.......................................................................   1     1   seq
..gggucguaaaggucggAc.......................................................................   1     1   seq
..gggucguaaaggucgguU.......................................................................   136   1   seq
..Nggucguaaaggucgguc.......................................................................   2     1   seq
..gggucguCaaggucgguc.......................................................................   1     1   seq
..gggucguaaaggucgguca......................................................................   4     0   seq
..gggucguaaaggucggucag.....................................................................   1     0   seq
...ggucguaaGggucgguc.......................................................................   1     1   seq
...ggucguaaaggucggGc.......................................................................   6     1   seq
...gUucguaaaggucgguc.......................................................................   3     1   seq
...ggCcguaaaggucgguc.......................................................................   1     1   seq
...ggucguaaaggucgguc.......................................................................   264   0   seq
...ggucguaaaggucgguA.......................................................................   2     1   seq
...ggucguaaaggucggCc.......................................................................   4     1   seq
...ggucguaaaggucgguG.......................................................................   1     1   seq
...Ugucguaaaggucgguc.......................................................................   1     1   seq
...ggucguaGaggucgguc.......................................................................   1     1   seq
...ggucguaaaggucgguU.......................................................................   25    1   seq
...ggucguaaGggucgguca......................................................................   1     1   seq
...ggucguaaaggucgguca......................................................................   9     0   seq
...ggucguaaaggucggucagc....................................................................   1     0   seq
....gucguaaaggucggCca......................................................................   1     1   seq
....gucguaGaggucgguca......................................................................   1     1   seq
....gucguaaaggucgguca......................................................................   23    0   seq
....gucguaaaggucggucag.....................................................................   3     0   seq
....gucguaaaggucggucagccu..................................................................   2     0   seq
.....ucguaaaggucggucag.....................................................................   7     0   seq
.....ucguaaGggucggucag.....................................................................   1     1   seq
......cguaaaggucggucagcc...................................................................   1     0   seq
......cguaaaggucggucagccC..................................................................   2     1   seq
......cguaaaggucggucagccu..................................................................   1     0   seq
......cguaaaggucggucagccG..................................................................   1     1   seq
......cguaaaggucggucagccua.................................................................   1     0   seq
........uaaaggucggucagccu..................................................................   3     0   seq
........uaaaggucggucagccua.................................................................   1     0   seq
........uaaaggucggucagccuaUucggu...........................................................   1     1   seq
.........aaaggucggucagccuaUu...............................................................   1     1   seq
..........aaggucggucagccuaU................................................................   1     1   seq
..........aaggucggucagccuaUu...............................................................   2     1   seq
..........aaggucggucagccuaUuc..............................................................   1     1   seq
..........aaggucggucagccuaUucgg............................................................   1     1   seq
..........aaggucggucagccuaUucggug..........................................................   2     1   seq
..........aaggucggucagccuaUucggugu.........................................................   1     1   seq
.....................gccuaUucggugugcac.....................................................   1     1   seq
.....................gccuaUucggugugcacc....................................................   4     1   seq
.....................gccuaUucggugugcaccu...................................................   6     1   seq
.....................gccuaUucggugugcaccuu..................................................   1     1   seq
......................ccuaUucggugugcacc....................................................   20    1   seq
......................ccuaUucggugugcaccu...................................................   75    1   seq
......................ccuaUucggugugcaccuu..................................................   2     1   seq
.......................cuaUucggugugcaccu...................................................   51    1   seq
.......................cuaUucggugugcaccuu..................................................   1     1   seq
.......................cuaUucggugugcaccuucc................................................   1     1   seq
........................uaUucggugugcaccuu..................................................   1     1   seq
........................uaUucggugugcaccuuccc...............................................   1     1   seq
..........................Uucggugugcaccuuccc...............................................   1     1   seq
..........................cucggugugcaccuucccu..............................................   1     0   seq
..........................Uucggugugcaccuucccuu.............................................   1     1   seq
..........................Uucggugugcaccuucccuuccg..........................................   1     1   seq
...........................ucggugugcaccuuccc...............................................   1     0   seq
...........................ucggugugcaccuucccu..............................................   1     0   seq
...........................ucggugugcaccGucccu..............................................   1     1   seq
```

Mature **Star**

uugggucguaaaggucggucagccuacucggugugcaccuucccuuccgucccguuugcugguggcaugcuucuggccuuaauuggcuggg			
...........................ucggugugcaccuCcccu..	1	1	seq
...........................ucggugugcaccuCcccuucc...	1	1	seq
...........................ucggugugcaccuGcccuuccgucccg.....................................	70	1	seq
...........................ucggugugcaccuGcccuuccgucccgu....................................	2	1	seq
...........................ucggugugcaccuGcccuuccgucccguu...................................	1	1	seq
............................cggugugcaccuucccu..	1	0	seq
............................cggugugcaccuucccuu...	1	0	seq
............................cggugugcaccuucccuuccgucccC.....................................	1	1	seq
............................cggugugcaccuGcccuuccgucccg.....................................	137	1	seq
............................cggugugcaccuGcccuuccgucccgu....................................	7	1	seq
.............................ggugugcaccuGcccuuccgucccg.....................................	79	1	seq
.............................ggugugcaccuGcccuuccgucccgu....................................	2	1	seq
..............................gugugcaccuCcccuuccgu...	1	1	seq
..............................gugugcaccuGcccuuccgucccg.....................................	50	1	seq
..............................gugugcaccuGcccuuccgucccgu....................................	2	1	seq
...............................ugugcaccuucccuuccgucc.......................................	1	0	seq
...............................ugugcaccuCcccuuccgucc.......................................	3	1	seq
...............................ugugcaccuGcccuuccgucccg.....................................	68	1	seq
...............................ugugcaccuGcccuuccgucccgu....................................	1	1	seq
................................gugcaccuucccuuccgucc.......................................	1	0	seq
................................gugcaccuGcccuuccgucccg.....................................	49	1	seq
................................gugcaccuGcccuuccgucccgu....................................	2	1	seq
.................................ugcaccuGcccuuccgucccg.....................................	72	1	seq
.................................ugcaccuGcccuuccgucccgu....................................	2	1	seq
.................................ugcaccuGcccuuccgucccguu...................................	1	1	seq
..................................gcaccuucccuuccguU..	1	1	seq
..................................gcaccuGcccuuccgucccg.....................................	94	1	seq
..................................gcaccuGcccuuccgucccgu....................................	1	1	seq
...................................caccuucccuuccgucc.......................................	1	0	seq
...................................caccuGcccuuccgucccg.....................................	228	1	seq
...................................caccuGcccuuccgucccgu....................................	6	1	seq
...................................caccuGcccuuccgucccguu...................................	2	1	seq
....................................accuGcccuuccgucccg.....................................	76	1	seq
....................................accuGcccuuccgucccgu....................................	3	1	seq
.....................................ccuGcccuuccgucccg.....................................	271	1	seq
.....................................ccuAcccuuccgucccg.....................................	1	1	seq
.....................................ccuGcccuuccgucccgu....................................	7	1	seq
.....................................ccuGcccuuccgucccguu...................................	1	1	seq
......................................cuGcccuuccgucccgu....................................	4	1	seq
......................................cuGcccuuccgucccguu...................................	1	1	seq
......................................cuGcccuuccgucccguuug.................................	1	1	seq
....uGcccuuccgucccguu...................................	2	1	seq
....cccuuccgucccUuuugcug..............................	1	1	seq
....ccuuccgucccUuuugcug..............................	2	1	seq
....ccuuccgucccUuuugcugg.............................	1	1	seq
....ccuuccgucccUuuugcuggu............................	1	1	seq
....cuuccgucccUuuugcug..............................	2	1	seq
....ccgucccUuuugcuggu............................	1	1	seq
....Ncuggccuuaauuggcu...	1	1	seq
....cuggccuuaauuggcug..	1	0	seq
....uggccuuaaCuggcuggg	1	1	seq

Provisional ID	:	MA_1173_7470
Score total	:	2.7
Score for star read(s)	:	-1.3
Score for read counts	:	0
Score for mfe	:	2.4
Score for randfold	:	1.6
Score for cons. seed	:	
Total read count	:	39
Mature read count	:	39
Loop read count	:	0
Star read count	:	0

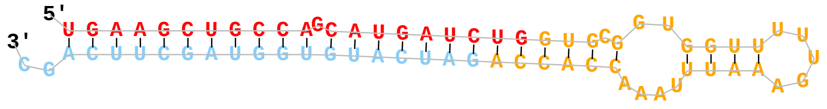

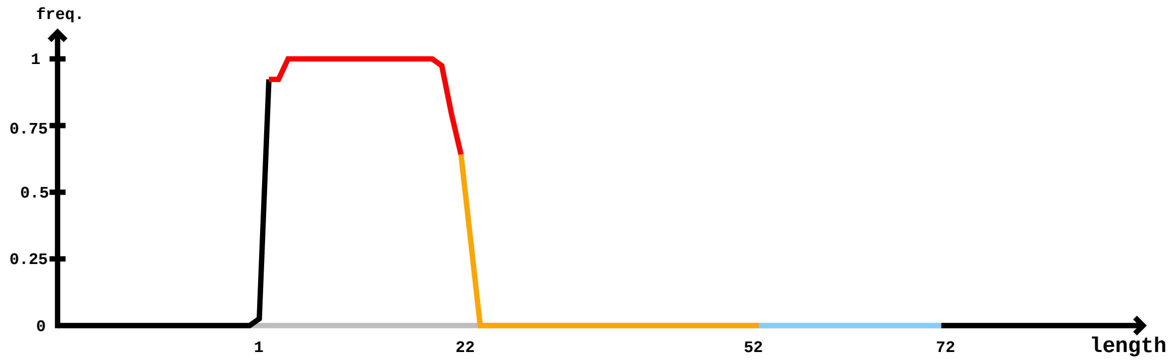

Mature Star

```
5'- uucuuguccgguggaacagcugaagcugccagcaugaucuggugcgguggguuuuugaaauuuaaaccaccagaucaugugguagcuucagcuucucccucauggacaugug  -3'    exp
    ....((((((..(((..((((((((((((((.(((((((((((((.(..((((.....))))...))))))))))))))))))))))))))))..)))....))))))....   reads    mm    sample
    ...................cugaagcugccagcaugaucug.......................................................................   1        0     seq
    ....................ugaagcugccagcaugau..........................................................................   1        0     seq
    ....................ugaagcugccGgcaugauc.........................................................................   1        1     seq
    ....................ugaagcugccagcaugauA.........................................................................   1        1     seq
    ....................ugaagcugccagcaCgauc.........................................................................   1        1     seq
    ....................ugaagcugccagcaugauc.........................................................................   4        0     seq
    ....................ugaagcugccagcaugaucu........................................................................   6        0     seq
    ....................ugaagcAgccagcaugaucug.......................................................................   1        1     seq
    ....................ugaagcugccagcaugaucug.......................................................................   11       0     seq
    ....................ugGagcugccagcaugaucugg......................................................................   1        1     seq
    ....................ugaagAugccagcaugaucugg......................................................................   1        1     seq
    ....................ugaagcugccagcaugaucugg......................................................................   7        0     seq
    ......................aagcugccagcaugaucug.......................................................................   3        0     seq
```

Provisional ID	: MA_117905_16855
Score total	: 0.9
Score for star read(s)	: -1.3
Score for read counts	: -0.3
Score for mfe	: 1
Score for randfold	: 1.6
Score for cons. seed	:
Total read count	: 11
Mature read count	: 11
Loop read count	: 0
Star read count	: 0

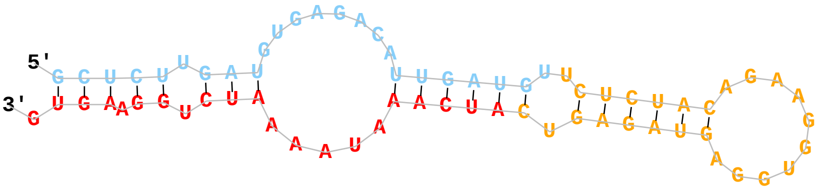

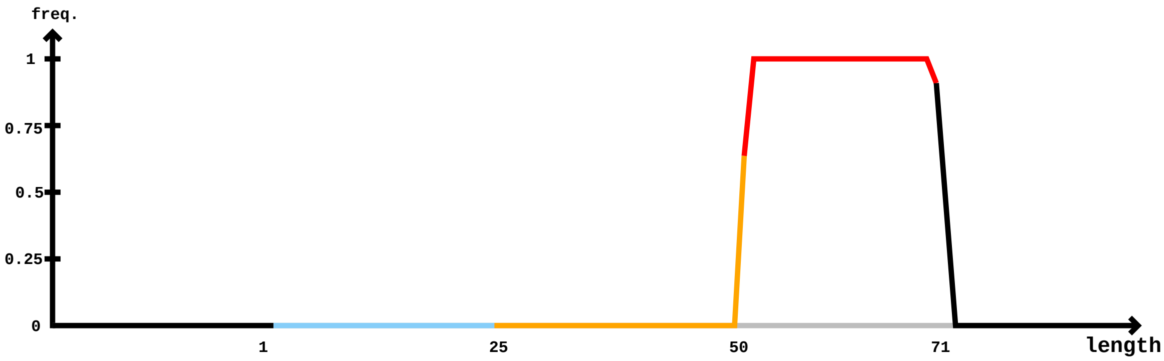

Star Mature

```
5'- caauauggaugaugguaacuagcucuugaugugagacauugauguucucuacagaagguggaguagagucaucaaauaaaaucuggaagugguugucaucccauaaacaac   -3'    exp
    ...(((((..((((..(((((..(((.(((........((((((..((((((..........)))))).))))))).....))).)))..)))))..)))))))))......   reads    mm    sample
    ......................................................................aucaaauaaaaucuggaagu.....................   1        0     seq
    ......................................................................aucaaauaaaaucuggaaguA....................   1        1     seq
    ......................................................................aucaaauaaaaucuggaagug....................   5        0     seq
    .......................................................................ucaaauaaaaucuggaagugU...................   3        1     seq
    .......................................................................ucaaauaaaaucuggaagugC...................   1        1     seq
```

Provisional ID	:	MA_119585_19652
Score total	:	194
Score for star read(s)	:	3.9
Score for read counts	:	186.7
Score for mfe	:	1.8
Score for randfold	:	1.6
Score for cons. seed	:	
Total read count	:	378
Mature read count	:	346
Loop read count	:	1
Star read count	:	31

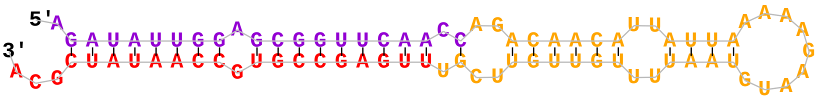

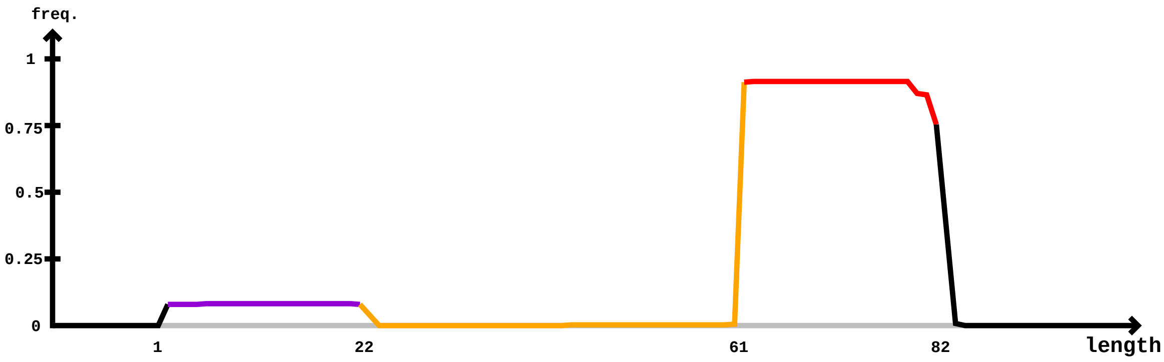

Star　　Mature

```
5'- agccgucuugagauauuggagcgguucaaccagacaacauuauuaaaaagaauguaauuuuguuguucguuugagccgugccaauaucgcacuagacuugcuuguuccauc  -3'  obs
    agccgucuugagauauuggagcgguucaaccagacaacauuauuaaaaagaauguaauuuuguuguucguuugagccgugccaauaucgcacuagacuugcuuguuccauc        exp
    (((.((((((.((((((((.(((((((((.(..((((((..((((.........))))..))))))..).))))))))).)))))))).))..))))..)))......... reads  mm  sample
    ..........agauauuggagcgguucaac................................................................................. 1    0   seq
    ..........agauauuggagcgguucaacc................................................................................ 25   0   seq
    ..........agauauuggagcgguucaacU................................................................................ 2    1   seq
    ..........Ngauauuggagcgguucaacc................................................................................ 1    1   seq
    ..........agauauuggagcgguCcaacc................................................................................ 1    1   seq
    ..............auuggagcgguucaacc................................................................................ 1    0   seq
    ....................................................uguaauuGuguuguucgu......................................... 1    1   seq
    .....................................................................Cuugagccgugccaauaucgca.................... 1    1   seq
    ......................................................................uugagccgugccaauauU....................... 1    1   seq
    ......................................................................uugagccgugccaauauc....................... 16   0   seq
    ......................................................................uugagccgugccaauaucg...................... 2    0   seq
    ......................................................................uugagccgugccaauaucgU..................... 10   1   seq
    ......................................................................uugagccgugccGauaucgc..................... 1    1   seq
    ......................................................................uugagccgugccaauaucgc..................... 28   0   seq
    ......................................................................uugagccAugccaauaucgc..................... 1    1   seq
    ......................................................................uugagccgugccaauaucgA..................... 1    1   seq
    ......................................................................uugUgccgugccaauaucgc..................... 1    1   seq
    ......................................................................uugagccgugccaauaucgca.................... 129  0   seq
    ......................................................................uCgagccgugccaauaucgca.................... 1    1   seq
    ......................................................................uugagccgugccaauaucgcG.................... 7    1   seq
    ......................................................................uugagccgugccaauaucgcC.................... 1    1   seq
    ......................................................................Nugagccgugccaauaucgca.................... 1    1   seq
    ......................................................................uugagccgugccaauaucgcU.................... 2    1   seq
    ......................................................................Cugagccgugccaauaucgcac................... 2    1   seq
    ......................................................................uugagccgugccaauaucgcac................... 119  0   seq
    ......................................................................uugagccgugccaauaucgcaA................... 3    1   seq
    ......................................................................uugagccgAgccaauaucgcac................... 1    1   seq
    ......................................................................uugagccgugccaauaCcgcac................... 1    1   seq
    ......................................................................uugagccgugccaauaucgcaG................... 1    1   seq
    ......................................................................uugagccgugccaauaucgcaU................... 12   1   seq
    ......................................................................uugagccgugccaauaucgcacC.................. 1    1   seq
    ......................................................................uugagccgugccaauaucgcacA.................. 1    1   seq
    ......................................................................uugagccgugccaauaucgcacu.................. 1    0   seq
```

Star Mature

```
agccgucuugagauauuggagcgguucaaccagacaacauuauuaaaaagaauguaauuuuguuguucguuugagccgugccaauaucgcacuagacuugcuuguuccauc
.......................................................................ugagccgugccaauaucAcac...................   1   1   seq
```

Provisional ID	:	MA_12028_169
Score total	:	1.3
Score for star read(s)	:	-1.3
Score for read counts	:	0
Score for mfe	:	1
Score for randfold	:	1.6
Score for cons. seed	:	
Total read count	:	6072
Mature read count	:	6072
Loop read count	:	0
Star read count	:	0

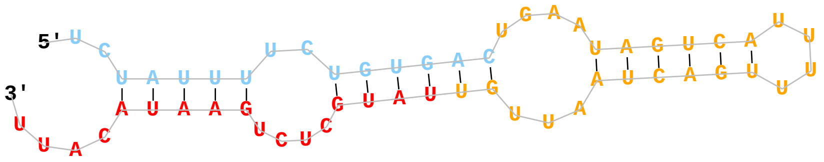

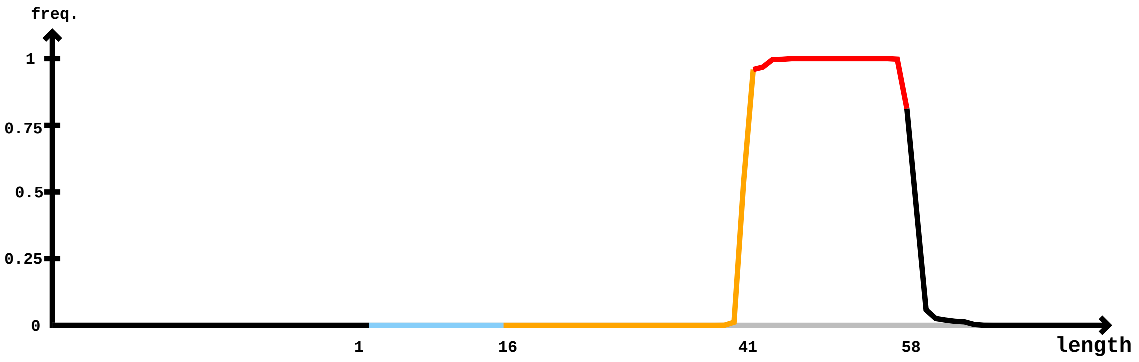

Star Mature

```
5'- uuguuugaggccaugcucagaugagcccuagucuauuuucugugacugaauagucauuuuugacuaauuguuaugcucugaauacauugggaugggguuucagauccau -3'  exp
..((((((((((..((((....))))((((((.(((((..((((((....((((((....))))))...))))))....))))).))))))...))))))))))....  reads  mm  sample
....................................................................Gguuaugcucugaauacau......................  2     1  seq
.....................................................................Nuuaugcucugaauaca.......................  5     1  seq
.....................................................................guuaugcucugaauaca.......................  8     0  seq
.....................................................................Nuuaugcucugaauacau......................  3     1  seq
.....................................................................guuaugcucugaauacaC......................  1     1  seq
.....................................................................guuaugcucugaauacau......................  2     0  seq
.....................................................................Nuuaugcucugaauacauu.....................  10    1  seq
.....................................................................guuaugcucugaauacauu.....................  4     0  seq
.....................................................................Cuuaugcucugaauacauug....................  9     1  seq
.....................................................................Cuuaugcucugaauacauugg...................  26    1  seq
.....................................................................guuaugcucugaauacauuggCaug...............  1     1  seq
......................................................................uuaugcucugaauacaG......................  14    1  seq
......................................................................uuaugcucugaauacaC......................  140   1  seq
......................................................................uuaugUucugaauacau......................  4     1  seq
......................................................................uuaugcucuUaauacau......................  1     1  seq
......................................................................uuaugcAcugaauacau......................  1     1  seq
......................................................................uuaugcucugaaGacau......................  1     1  seq
......................................................................uuaugcucugaauGcau......................  5     1  seq
......................................................................uuUugcucugaauacau......................  1     1  seq
......................................................................uuaugcucCgaauacau......................  3     1  seq
......................................................................uuaugcucugGauacau......................  2     1  seq
......................................................................uuauAcucugaauacau......................  2     1  seq
......................................................................uuaugcucugaauacau......................  944   0  seq
......................................................................uuaugcucugaauacaA......................  4     1  seq
......................................................................Nuaugcucugaauacau......................  5     1  seq
......................................................................uuaugcCcugaauacau......................  3     1  seq
......................................................................uuaCgcucugaauacau......................  1     1  seq
......................................................................uAaugcucugaauacau......................  1     1  seq
......................................................................uuauCcucugaauacau......................  1     1  seq
......................................................................uuaugcucugaUuacauu.....................  1     1  seq
......................................................................uuaugcucugaauacauu.....................  1794  0  seq
......................................................................uuaugcucugaauacaAu.....................  1     1  seq
......................................................................uuaugAucugaauacauu.....................  2     1  seq
......................................................................uuaugcucCgaauacauu.....................  2     1  seq
```

Star Mature

```
uuguuugaggccaugcucagaugagcccuagucuauuuucugugacugaauagucauuuuugacuaauuguuaugcucugaauacauugggauggguuucagauccau
......................................................................uuaugUucugaauacauu....................	1	1	seq
......................................................................uuaugcucGgaauacauu....................	1	1	seq
......................................................................Guaugcucugaauacauu....................	2	1	seq
......................................................................uuaugcucugaauacauA....................	7	1	seq
......................................................................uuaugcCcugaauacauu....................	2	1	seq
......................................................................uuaCgcucugaauacauu....................	7	1	seq
......................................................................Nuaugcucugaauacauu....................	5	1	seq
......................................................................uuaugcucugaauacaGu....................	2	1	seq
......................................................................uuaugcucugUauacauu....................	1	1	seq
......................................................................uuauAcucugaauacauu....................	2	1	seq
......................................................................uuaugcucugaauGcauu....................	3	1	seq
......................................................................Auaugcucugaauacauu....................	2	1	seq
......................................................................uuaugcucugaauaAauu....................	1	1	seq
......................................................................uuaugcucugaauCcauu....................	1	1	seq
......................................................................uuaugcucugaauacauG....................	9	1	seq
......................................................................uuaugcucugaauacaCu....................	6	1	seq
......................................................................uuaugcucugaauUcauu....................	1	1	seq
......................................................................uuaugcucugaauacauC....................	190	1	seq
......................................................................uuaugcucugGauacauu....................	9	1	seq
......................................................................uuaugcucugaauaGauu....................	1	1	seq
......................................................................uuaugcucugaauacauuU...................	3	1	seq
......................................................................uuaugcucugaauacauug...................	6	0	seq
......................................................................uuaugcucugaauacauugC..................	2	1	seq
......................................................................uuaugcucCgaauacauugg..................	1	1	seq
......................................................................uuaugcucugaauacauugg..................	10	0	seq
......................................................................uuaugcucugaauacauuUg..................	10	1	seq
......................................................................uuaugcucugaauacauugA..................	1	1	seq
......................................................................uuaugcucugaauacauuCg..................	2	1	seq
......................................................................uuaugcucugaauacauuggU.................	2	1	seq
......................................................................uuaugcucugaauacauuggC.................	8	1	seq
......................................................................uuaugcucugaauacauuggCa................	1	1	seq
......................................................................uuaugcucugaauacauuggCau...............	2	1	seq
......................................................................uuaugcucugaauacauuggCaug..............	1	1	seq
......................................................................uuaugcucugaauacauuggCaugg.............	2	1	seq
.......................................................................uaugcucugaauacauu....................	2272	0	seq
.......................................................................uaugcucugaauaGauu....................	1	1	seq
.......................................................................uaugcucugaauacauC....................	207	1	seq
.......................................................................uaugUucugaauacauu....................	5	1	seq
.......................................................................uaugcucugaauacauA....................	11	1	seq
.......................................................................Naugcucugaauacauu....................	1	1	seq
.......................................................................uUugcucugaauacauu....................	2	1	seq
.......................................................................Gaugcucugaauacauu....................	2	1	seq
.......................................................................Aaugcucugaauacauu....................	2	1	seq
.......................................................................uaugcGcugaauacauu....................	1	1	seq
.......................................................................uaugcCcugaauacauu....................	7	1	seq
.......................................................................uaugcucugaauacaGu....................	2	1	seq
.......................................................................uaugcucCgaauacauu....................	2	1	seq
.......................................................................uaAgcucugaauacauu....................	3	1	seq
.......................................................................uaugcucugaauacaCu....................	5	1	seq
.......................................................................uaCgcucugaauacauu....................	6	1	seq
.......................................................................uaugcucugaauGcauu....................	2	1	seq
.......................................................................uaugcucugaauacUuu....................	1	1	seq
.......................................................................uaugcucugaCuacauu....................	2	1	seq
.......................................................................uaugcucugGauacauu....................	5	1	seq
.......................................................................uaugcucugaauacaGug...................	1	1	seq
.......................................................................uaugcucugaauGcauug...................	1	1	seq
.......................................................................uaugcucugaauacauAg...................	1	1	seq
.......................................................................uaugcucugaauacauug...................	3	0	seq
.......................................................................uaugcucugaauacauuC...................	1	1	seq
.......................................................................uaugcucugaCuacauugg..................	2	1	seq
.......................................................................uaugcucugaauacauuCg..................	1	1	seq
.......................................................................uaugcucugaauacauugg..................	16	0	seq
.......................................................................uaugcucugUauacauugg..................	1	1	seq
.......................................................................uaugcucugaauacauuUg..................	5	1	seq
.......................................................................uaugcucugaauacauuggU.................	2	1	seq
.......................................................................uaugcucugaauacauuggC.................	7	1	seq
.......................................................................uaugcucugaauacauuggCa................	6	1	seq
.......................................................................uaugcucugaauacauuggCau...............	4	1	seq
.......................................................................uaugcucugaauacauuggCaug..............	1	1	seq
.......................................................................uaugcucugaauacauuggCaugg.............	3	1	seq
```

```
                               Star                      Mature
uuguuugaggccaugcucagaugagcccuagucuauuuucugugacugaauagucauuuuugacuaauuguuaugcucugaauacauugggaugggguuucagauccau
.......................................................................uaugcucugaauacauuggCauggg............  1   1   seq
........................................................................augcucugaauacauug...................  9   0   seq
........................................................................augcucugaauacauAg...................  2   1   seq
........................................................................augcucugaauacauuC...................  1   1   seq
........................................................................augcucugaauacauugA..................  1   1   seq
........................................................................Nugcucugaauacauugg..................  1   1   seq
........................................................................augcucugaauacauuUg..................  5   1   seq
........................................................................augcucugaauacauugg..................  12  0   seq
........................................................................augcucugaauacauuggC.................  5   1   seq
........................................................................augcucugaauacauuggCa................  8   1   seq
........................................................................augcucugaauacauuAggau...............  1   1   seq
........................................................................augcucugaauacauuggCau...............  1   1   seq
........................................................................augcucugaauacauuggCaug..............  8   1   seq
........................................................................augcucugaauacauuggCaugg.............  1   1   seq
.........................................................................ugcucugaauacauuCg..................  3   1   seq
.........................................................................ugcucugaauacauuUg..................  26  1   seq
.........................................................................ugcucugaauacauugA..................  5   1   seq
.........................................................................Cgcucugaauacauugg..................  1   1   seq
.........................................................................ugcucugaauacauugg..................  69  0   seq
.........................................................................ugcucugaauacauuggC.................  10  1   seq
.........................................................................ugcucugaauacauuggCa................  10  1   seq
.........................................................................ugcucugaauacauuggCau...............  1   1   seq
.........................................................................ugcucugaauacauuAggau...............  1   1   seq
.........................................................................ugcucugaauacauuggCaug..............  39  1   seq
.........................................................................ugcucugaauacauuggCaugg.............  8   1   seq
..........................................................................gcucugaauacauuggC.................  2   1   seq
..........................................................................gcucugaauacauuggCa................  3   1   seq
..........................................................................gcucugaauacauuggCaug..............  1   1   seq
..........................................................................gcucugaauacauuggCaugg.............  1   1   seq
...........................................................................cucugaauacauuAggau...............  2   1   seq
...........................................................................cucugaauacauuggCau...............  1   1   seq
...........................................................................cucugaauacauuggCaug..............  9   1   seq
...........................................................................cucugaauacauuggCaugg.............  4   1   seq
```

Provisional ID	:	MA_121504_18444
Score total	:	2.1
Score for star read(s)	:	-1.3
Score for read counts	:	0
Score for mfe	:	1.8
Score for randfold	:	1.6
Score for cons. seed	:	
Total read count	:	92
Mature read count	:	92
Loop read count	:	0
Star read count	:	0

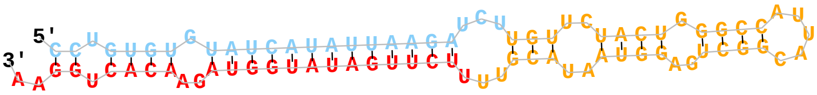

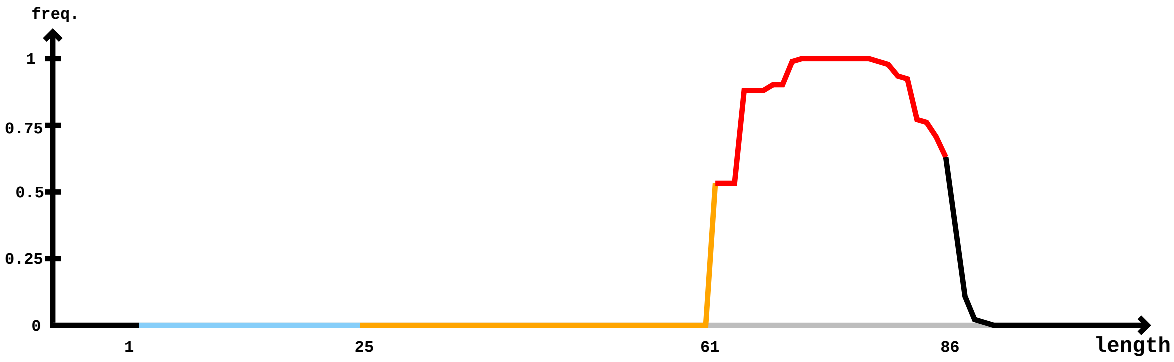

Star **Mature**

```
5'- gaggcuuccuguguguaucauauuaagaucuuguucuacugggccauuacggcugagguaauacguuuucuugauaugguagaacacuggaacacuccuccaugcauugaca   -3'    exp
    ((((.((((.((((.((((((((((((...(((..((((.((((.....))))..))))..)))...))))))))))))..)))).)))).))))....)))).............   reads   mm   sample
    ..................................................................uucuugaCaugguagaa............................   1   1   seq
    ..................................................................uucuugaCaugguagaac...........................   1   1   seq
    ..................................................................uucuugaCaugguagaaca..........................   4   1   seq
    ..................................................................uucuugaCaugguagaacac.........................   1   1   seq
    ..................................................................uucuugaCaugguagaacacu........................   14   1   seq
    ..................................................................uucuugaCaugguagaacacugg......................   2   1   seq
    ..................................................................uucuugaCaugguagaacacugga.....................   4   1   seq
    ..................................................................uucuugaCaugguagaacacuggaa....................   22   1   seq
    .....................................................................uugaGaugguagaacacug.......................   1   1   seq
    .....................................................................uugauaugguagaacacugg......................   1   0   seq
    .....................................................................uugauaugguagaaUacugg......................   1   1   seq
    .....................................................................uugauaugguagaacacugU......................   1   1   seq
    .....................................................................uugauaugguagaacacugga.....................   3   0   seq
    .....................................................................uugauaugguagaacacCggaa....................   1   1   seq
    .....................................................................uugaAaugguagaacacuggaa....................   2   1   seq
    .....................................................................uugauaugguagaacacuggaa....................   10   0   seq
    .....................................................................uugaGaugguagaacacuggaa....................   4   1   seq
    .....................................................................uugaCaugguagaacacuggaaca..................   6   1   seq
    .....................................................................uugaCaugguagaacacuggaacac.................   1   1   seq
    .....................................................................uugaCaugguagaacacuggaacacu................   1   1   seq
    ........................................................................aCaugguagaacacuggaaca..................   2   1   seq
    ..........................................................................augguagaacacuggGa....................   1   1   seq
    ..........................................................................augguagaacacuggaa....................   7   0   seq
    ...........................................................................ugguagaacacuggaac...................   1   0   seq
```

Provisional ID	:	MA_12301_3845
Score total	:	795.4
Score for star read(s)	:	3.9
Score for read counts	:	789.8
Score for mfe	:	0.1
Score for randfold	:	1.6
Score for cons. seed	:	
Total read count	:	1561
Mature read count	:	1559
Loop read count	:	1
Star read count	:	1

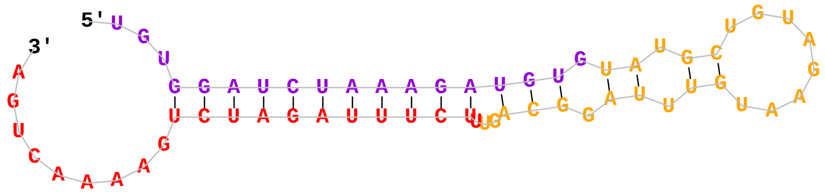

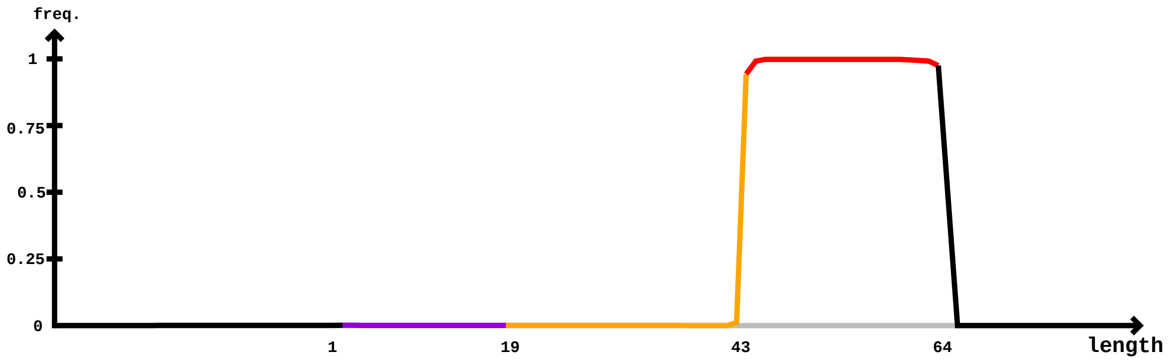

Star **Mature**

```
5'- gaauuugcuucagaucuaguuugaaugauguggaucuaaagauguguaugcuguagaauguuuaggcaguuucuuuagaucugaaaacugauuauguuauagaucugcugu  -3'   obs
    gaauuugcuucagaucuaguuugaaugauguggaucuaaagauguguaugcuguagaauguuuaggcaguuucuuuagaucugaaaacugauuauguuauagaucugcugu        exp
    ......((..((((((((......(((((((((((((((((((((.((.((........)).)).)))...)))))))))))....))..)))))....))))))))..))  reads    mm    sample
    .........uAagaucuaguuugaaugaug.................................................................................  1        1     seq
    ............................uguggaucGaaagaugug.................................................................  1        1     seq
    ...............................................augcuguagaCuguuua...............................................  1        1     seq
    .....................................................................Auucuuuagaucugaaaac.......................  1        1     seq
    .....................................................................Auucuuuagaucugaaaacug.....................  16       1     seq
    ......................................................................uucuuuagaucugaaaa........................  3        0     seq
    ......................................................................uucuuuagaucugaaaac.......................  1        0     seq
    ......................................................................uucuuuagaucugaaaacC......................  1        1     seq
    ......................................................................Gucuuuagaucugaaaacu......................  1        1     seq
    ......................................................................uucuuuagaucugaaaacu......................  1        0     seq
    ......................................................................uucuuuagaucugaaaacug.....................  9        0     seq
    ......................................................................uucuuuagaucugaaaacugU....................  149      1     seq
    ......................................................................uucuuuagaucugaaaacuga....................  19       0     seq
    ......................................................................uucuuuagaucugaaaacugC....................  1270     1     seq
    ......................................................................uucCuuagaucugaaaacuga....................  1        1     seq
    ......................................................................uucuuCagaucugaaaacuga....................  1        1     seq
    .......................................................................ucuuuagaucugaaaac.......................  1        0     seq
    .......................................................................ucuuuagaucugaaaacu......................  1        0     seq
    .......................................................................ucuuuagaucugaaaacug.....................  1        0     seq
    .......................................................................ucuuuagaucugaaaacuga....................  1        0     seq
    .......................................................................ucuuuagaucugaaaacugC....................  61       1     seq
    .......................................................................ucuuuagaucugaaaacugU....................  11       1     seq
    ........................................................................cuuuagaucugaaaacugC....................  10       1     seq
```

Provisional ID	:	MA_12306_7547
Score total	:	1.8
Score for star read(s)	:	-1.3
Score for read counts	:	0
Score for mfe	:	1.5
Score for randfold	:	1.6
Score for cons. seed	:	
Total read count	:	1169
Mature read count	:	1169
Loop read count	:	0
Star read count	:	0

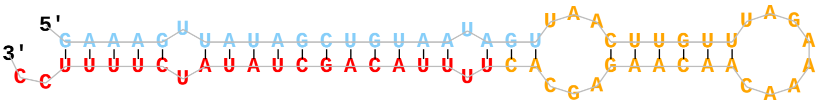

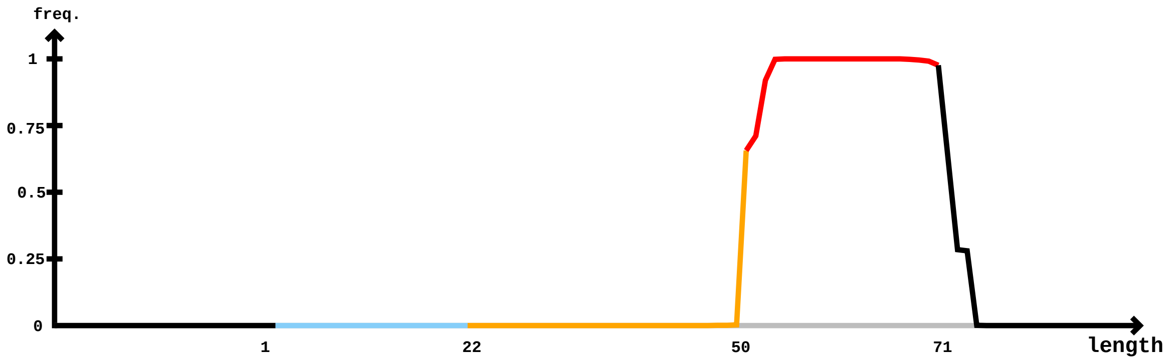

Star Mature

```
5'- caucgguaaacugcaagaagagaaaguuauagcuguaaauaguuaacuuguuuagaaaacaacaagagcacuuuuacagcuauaucuuuuccaucuagcucauuuacugcua   -3'   exp
    ...(((((((..((.(((.(..((((.(((((((((((.(((...((((((........))))))...))).))))))))))).)))).).))).))...)))))))...   reads   mm   sample
    ....................................................................cacuuuuacagcuauaucuuu.......................   1   0   seq
    ......................................................................cuuuuacagcuauaucuuuu......................   1   0   seq
    ......................................................................cuuuuacagcuauaucuuuuc.....................   1   0   seq
    .......................................................................uuuuacagcuauaucuu........................   2   0   seq
    .......................................................................uuuuacagcuauaucuuu.......................   2   0   seq
    .......................................................................uuuuacagcuauaucuuuu......................   3   0   seq
    .......................................................................uuuuacagcuauaucuuuuc.....................   13   0   seq
    .......................................................................uuuuacagcuauaucuuuuU.....................   1   1   seq
    .......................................................................uCuuacagcuauaucuuuucc....................   4   1   seq
    .......................................................................uuuuacagcuauaucCuuucc....................   5   1   seq
    .......................................................................uuuuacagcuauGucuuuucc....................   2   1   seq
    .......................................................................uuuuGcagcuauaucuuuucc....................   1   1   seq
    .......................................................................uuuuacagcuauaucuuCucc....................   6   1   seq
    .......................................................................uuuuacGgcuauaucuuuucc....................   4   1   seq
    .......................................................................uuCuacagcuauaucuuuucc....................   2   1   seq
    .......................................................................uuuuacagcuauaucuCuucc....................   2   1   seq
    .......................................................................uuuuacagcuGuaucuuuucc....................   4   1   seq
    .......................................................................uuuuacagcAauaucuuuucc....................   1   1   seq
    .......................................................................uuuuacagcuauaucuuuucG....................   1   1   seq
    .......................................................................uuuuacagcuauaucuuuucU....................   46   1   seq
    .......................................................................Cuuuacagcuauaucuuuucc....................   2   1   seq
    .......................................................................uuuuacagcuauaucuuuucA....................   24   1   seq
    .......................................................................uuuCacagcuauaucuuuucc....................   1   1   seq
    .......................................................................uuuuacCgcuauaucuuuucc....................   5   1   seq
    .......................................................................uuuuacagcuauaucuuuucc....................   618   0   seq
    .......................................................................Nuuuacagcuauaucuuuucc....................   1   1   seq
    .......................................................................uuuuacagGuauaucuuuucc....................   1   1   seq
    .......................................................................Auuuacagcuauaucuuuucc....................   1   1   seq
    .......................................................................uuuuacagcuauaCcuuuucc....................   1   1   seq
    .......................................................................uuuuaUagcuauaucuuuucc....................   1   1   seq
    .......................................................................uuuuacagcuauaucuuuuccU...................   3   1   seq
    .......................................................................uuuuacUgcuauaucuuuucca...................   2   1   seq
    .......................................................................uuuuacagcAauaucuuuucca...................   1   1   seq
    .......................................................................uuuuacagcuauaucuuuucca...................   2   0   seq
```

Star　　　　　　　　　　　　　　　　　　　　　　　　Mature

```
caucgguaaacugcaagaagagaaaguuauagcuguaauaguuaacuuguuuagaaaacaacaagagcacuuuuacagcuauaucuuuuccaucuagcucauuuacugcua
......................................................................uuuuacagcuauaucuuuuccauc.................    2     0    seq
......................................................................uuuuacagcuauaucuuuuccauU.................    1     1    seq
.......................................................................uuuacagcuauaucuuuA......................    1     1    seq
.......................................................................uuuacagcuauaucuuuuc.....................    2     0    seq
.......................................................................uuuacagcuauaucuuuucc....................    14    0    seq
.......................................................................uuuacCgcuauaucuuuucc....................    2     1    seq
.......................................................................uuuacagcuauaucuuuucU....................    1     1    seq
.......................................................................uuuacUgcuauaucuuuucca...................    11    1    seq
.......................................................................uuuacagcuauauGuuuucca...................    1     1    seq
.......................................................................uuuacagcuauaucuuuuccU...................    1     1    seq
.......................................................................uuuacagcuauaucuuuucca...................    26    0    seq
.......................................................................uuuacagcuauaucuuuuccG...................    3     1    seq
.......................................................................uuuacagcuauaucuuuuccau..................    1     0    seq
.......................................................................uuuacagcuauaucuuuuccauU.................    1     1    seq
........................................................................uuacagcuauaucuuuuc.....................    1     0    seq
........................................................................uuacagcuauaucuuuucc....................    4     0    seq
........................................................................uuacagcuauaucuuuucca...................    4     0    seq
........................................................................uuacagcuauaucuuuuccau..................    3     0    seq
........................................................................uuacagcuauaucuuuuccaC..................    1     1    seq
........................................................................Guacagcuauaucuuuuccauc.................    1     1    seq
........................................................................uuacagcuauaucuuuuccauA.................    2     1    seq
........................................................................uuacagcuauaucCuuuccauc.................    1     1    seq
........................................................................uuacagcuauaCcuuuuccauc.................    1     1    seq
........................................................................uuacagcuauaucuuuuccauU.................    9     1    seq
........................................................................uuacagcuauaucuuuuccauc.................    213   0    seq
........................................................................uuacagcCauaucuuuuccauc.................    1     1    seq
........................................................................uuacagcuauGucuuuuccauc.................    1     1    seq
........................................................................uuacagcuauaucuuuuccaCc.................    2     1    seq
.........................................................................uacagcuauaucuuuucU....................    1     1    seq
.........................................................................uacagcuauaucuuuuccauU.................    4     1    seq
.........................................................................uacagcuauaucuuuuccauc.................    82    0    seq
.........................................................................uacagcuauaucuuuuccaCc.................    1     1    seq
.........................................................................uacagUuauaucuuuuccauc.................    1     1    seq
.........................................................................uacagcuauaucuuuuccauA.................    2     1    seq
.........................................................................uacagcuauaucuuuuccaucu................    1     0    seq
..........................................................................acagcuauaucuuuuccauc.................    1     0    seq
..........................................................................acagcuauaucuuuuccauU.................    1     1    seq
```

Provisional ID : MA_123592_21867
Score total : 0.4
Score for star read(s) : -1.3
Score for read counts : 0
Score for mfe : 0.1
Score for randfold : 1.6
Score for cons. seed :
Total read count : 60
Mature read count : 60
Loop read count : 0
Star read count : 0

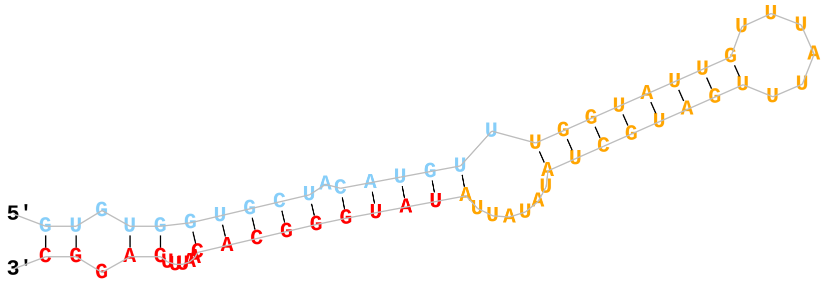

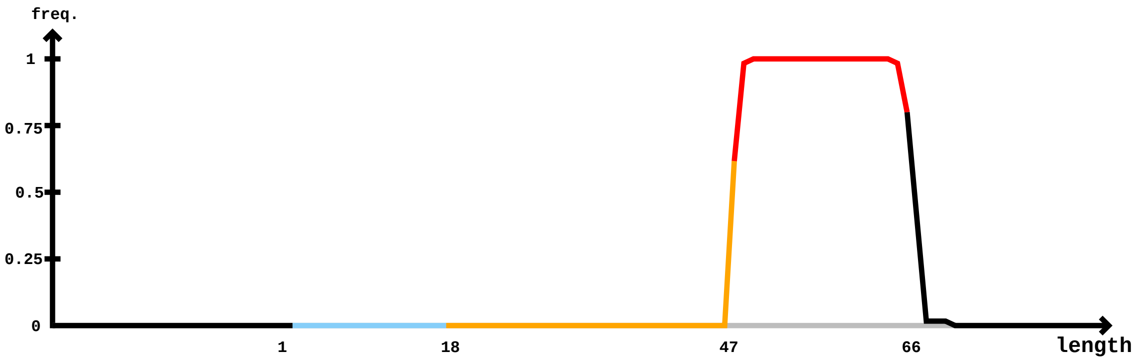

Star Mature

```
5'- ggcaugguuguuuugagcuaugugugugguggcuacauguuugguauuguuuauuugaugcuauauauuauaugggcacaauuucaggcaacuagcuagcaaauuauga   -3'    exp
    ..(((((((......((((((((.((..(((((.(((((.((((((((......))))))))......))))))))))).....)).)))..)))))....))))))).   reads   mm   sample
    .....................................................................uaugggcacaauuucag......................   1   0   seq
    .....................................................................uaugggcacaauuucagg.....................   6   0   seq
    .....................................................................uaugggcacaauuucagA.....................   1   1   seq
    .....................................................................uaugggcacaauuucaggc....................   17   0   seq
    .....................................................................uaugggcacaauuucaggA....................   1   1   seq
    .....................................................................uaugggcacaauuucaggU....................   3   1   seq
    .....................................................................uaugggcacaaAuucaggca...................   1   1   seq
    .....................................................................uaugggcacaauuucaggca...................   6   0   seq
    .....................................................................uaugggcacaauuucaggcaGcu................   1   1   seq
    ......................................................................augggcacaauuucagg.....................   3   0   seq
    ......................................................................Cugggcacaauuucagg.....................   1   1   seq
    ......................................................................Cugggcacaauuucaggc....................   3   1   seq
    ......................................................................augggcacaauuucaggc....................   10   0   seq
    ......................................................................Gugggcacaauuucaggc....................   1   1   seq
    ......................................................................augggcacaauuucaggca...................   4   0   seq
    .......................................................................ugggcacaauuucaggca...................   1   0   seq
```

Provisional ID	:	MA_125535_24568
Score total	:	11.2
Score for star read(s)	:	3.9
Score for read counts	:	3.7
Score for mfe	:	2
Score for randfold	:	1.6
Score for cons. seed	:	
Total read count	:	19
Mature read count	:	18
Loop read count	:	0
Star read count	:	1

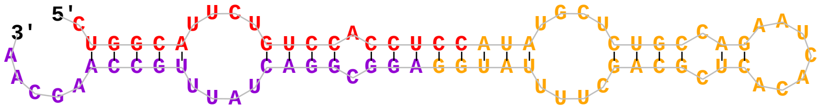

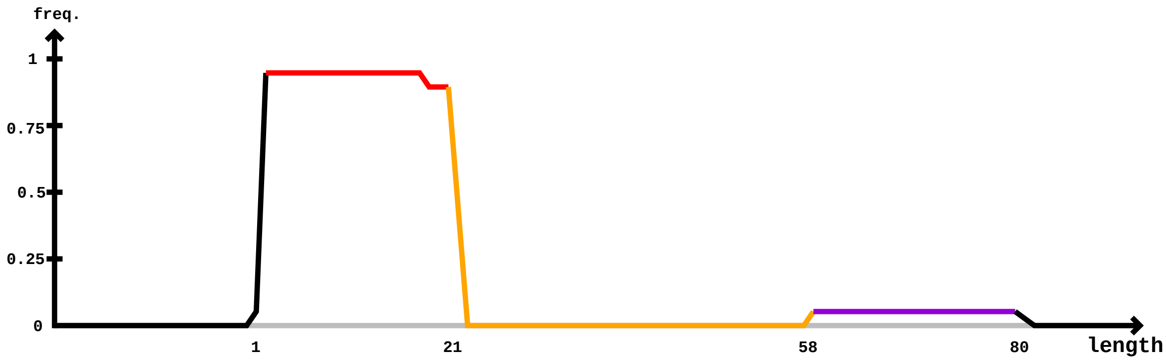


```
                    Mature                                                 Star
5'- aaaacuccuuucagagaguacuggcauucuguccaccuccauaugcucugccagaaucacacucgcagcuuuuauggaggcggacuauuugccaagcaagcucugccaggc   -3'    obs
    aaaacuccuuucagagaguacuggcauucuguccaccuccauaugcucugccagaaucacacucgcagcuuuuauggaggcggacuauuugccaagcaagcucugccaggc          exp
    ......(((..(((((..(.((((((....((((.((((((((....((((.((.......)).))))....)))))))).))))....)))).)).)..))))).))).    reads     mm     sample
    ...................acuggcauucuguccaccucU.......................................................................    1         1      seq
    ....................cuggcauucuguccacc..........................................................................    1         0      seq
    ....................cuggcauucuguccaccucU.......................................................................    3         1      seq
    ....................Guggcauucuguccaccucc.......................................................................    1         1      seq
    ....................cuggcauucuguccaccucc.......................................................................    5         0      seq
    ....................cuggcauucCguccaccucca......................................................................    2         1      seq
    ....................cuggcauucuguccaccuccC......................................................................    1         1      seq
    ....................cuggcauucuguccaccucca......................................................................    4         0      seq
    .............................................................................aggcggacuauuugccaagcaa............    1         0      seq
```

Provisional ID	:	MA_125829_18275
Score total	:	0.5
Score for star read(s)	:	-1.3
Score for read counts	:	0
Score for mfe	:	0.2
Score for randfold	:	1.6
Score for cons. seed	:	
Total read count	:	16
Mature read count	:	16
Loop read count	:	0
Star read count	:	0

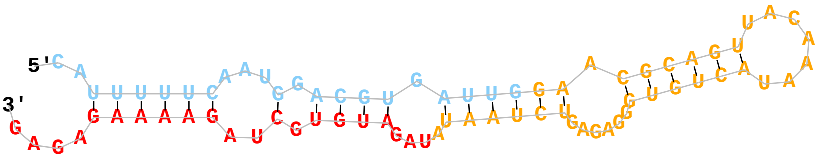

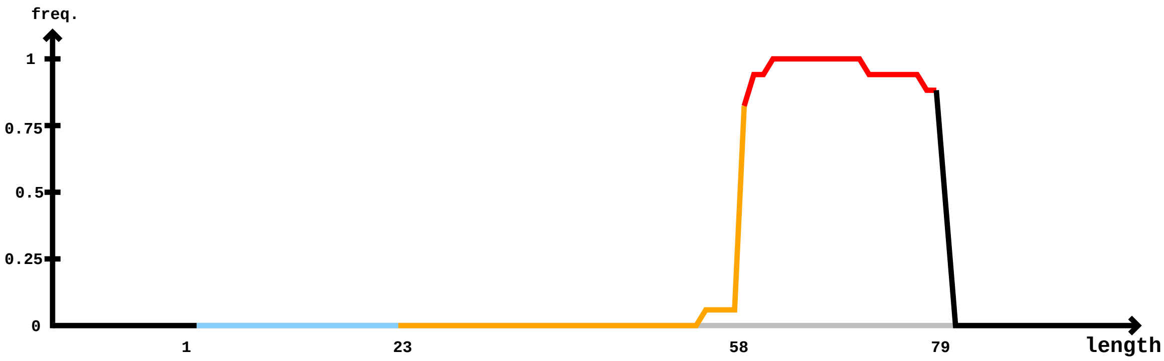

Star Mature

```
5'- cuugacaucugcucauuuuucaauggacgugauuggaacgcaguuacaaauacugugggagagucuaauauagaugugcuagaaaagagagggcuugucaacaaagacaaa   -3'    exp
    .((((((..(.(((.((((((...(.((((.((((((.((((((.......))))))......))))))....)))).)..)))))).))).)..))))))..........   reads     mm      sample
    ..................................................................aauauUgaugugcuaga............................   1         1       seq
    ......................................................................uagaugugcuagaaaagag......................   1         0       seq
    ......................................................................uagaugugcuagaaaagagag....................   12        0       seq
    .......................................................................agaugugcuagaaaagagagU...................   1         1       seq
    .......................................................................agaugugcuagaaaagagagC...................   1         1       seq
    .........................................................................augugcuagaaaagagagC...................   1         1       seq
```

Provisional ID	:	MA_1285879_43465
Score total	:	0.9
Score for star read(s)	:	-1.3
Score for read counts	:	0
Score for mfe	:	0.6
Score for randfold	:	1.6
Score for cons. seed	:	
Total read count	:	4626
Mature read count	:	4622
Loop read count	:	4
Star read count	:	0

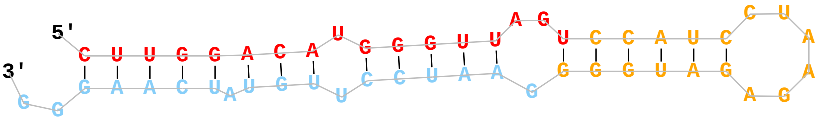

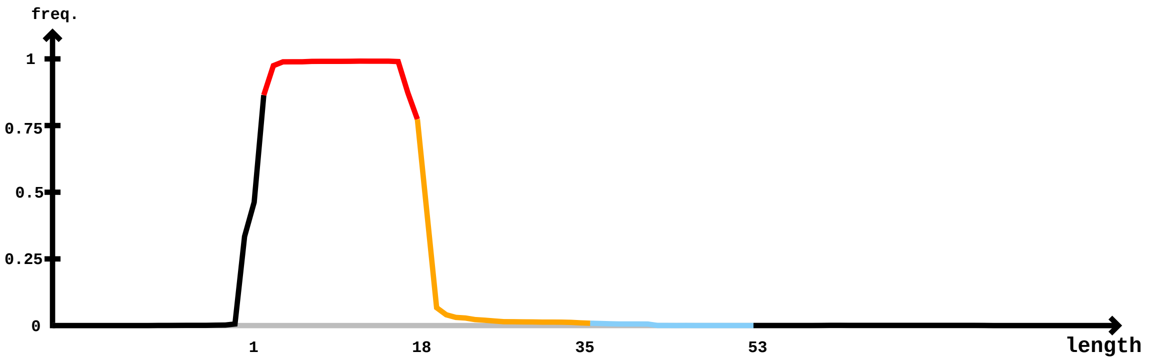

Mature Star

```
5'- aauguuccaugugaaaaucacuuggacauggguuaguccauccuaagagaugggggaauccuuguaucaaggguguacucugcaugaacaucgaaagauaauuggguuaa   -3'    exp
    ..(((((.((((((..(((.((((((((.(((((..((((((......)))))).))))).))).))))))))....)).)))))))))((....))............   reads   mm   sample
    .........ugugaaaaGcacuugga...................................................................................   1       1    seq
    .........ugugaaaaGcacuuggacaugg..............................................................................   1       1    seq
    ...........ugaaaaGcacuuggaca.................................................................................   1       1    seq
    ............gaaaaGcacuuggacaugg..............................................................................   1       1    seq
    ...............aaGcacuuggacaugggu............................................................................   1       1    seq
    ...............aaGcacuuggacauggguu...........................................................................   1       1    seq
    ...............aaGcacuuggacauggguua..........................................................................   1       1    seq
    ................aucacuuggacauggguu...........................................................................   1       0    seq
    ................aCcacuuggacauggguu...........................................................................   1       1    seq
    .................Ncacuuggacauggguu...........................................................................   1       1    seq
    .................ucacuuggacauggguu...........................................................................   2       0    seq
    .................Ccacuuggacauggguu...........................................................................   1       1    seq
    .................Ccacuuggacauggguua..........................................................................   1       1    seq
    .................Ncacuuggacauggguua..........................................................................   1       1    seq
    .................ucacuuggacauggguua..........................................................................   1       0    seq
    .................ucacuuggacauggguuag.........................................................................   1       0    seq
    .................Ncacuuggacauggguuag.........................................................................   1       1    seq
    .................ucacuuggacauggguuagu........................................................................   2       0    seq
    .................Gcacuuggacauggguuagucc......................................................................   4       1    seq
    .................Gcacuuggacauggguuagucca.....................................................................   1       1    seq
    .................ucacuuggacauggguuagucGauccuaagagaug.........................................................   1       1    seq
    .................ucacuuggacauggguuagucGauccuaagagaugggg......................................................   2       1    seq
    ..................cacuuggacaCggguua..........................................................................   1       1    seq
    ..................cacuuggacGuggguua..........................................................................   2       1    seq
    ..................cacuuggacaugggguGa.........................................................................   1       1    seq
    ..................cacuuggaUauggguua..........................................................................   1       1    seq
    ..................cacuuggacauggguua..........................................................................   534     0    seq
    ..................cacuCggacauggguua..........................................................................   3       1    seq
    ..................cacuuggacauggguuC..........................................................................   1       1    seq
    ..................cacuuggacaugCguua..........................................................................   2       1    seq
    ..................cGcuuggacauggguua..........................................................................   2       1    seq
    ..................Nacuuggacauggguua..........................................................................   1       1    seq
    ..................Gacuuggacauggguua..........................................................................   2       1    seq
    ..................cacuuggacaugUguua..........................................................................   1       1    seq
```

Mature Star

aauguuccaugugaaaaucacuuggacauggguuaguccauccuaagagaugggaauccuuguaucaaggguguacucugcaugaacaucgaaagauaauuggguuaa			
..................cacAuggacauggguua..	1	1	seq
..................cacuuggacauggguuag...	320	0	seq
..................Nacuuggacauggguuag...	1	1	seq
..................cacuuggacauggguuaA...	3	1	seq
..................Gacuuggacauggguuag...	1	1	seq
..................cacuuggacauggUuuag...	1	1	seq
..................cacuuggacauggguAag...	1	1	seq
..................cacCuggacauggguuag...	1	1	seq
..................cacuGggacauggguuag...	1	1	seq
..................cacuuggacauggguCag...	1	1	seq
..................cacuuggacGuggguuag...	1	1	seq
..................cacuuggacaAggguuag...	1	1	seq
..................cacuuggacauggguuaU...	1	1	seq
..................cacuuggacaugggGuagu..	1	1	seq
..................cacuuggacaugggAuagu..	1	1	seq
..................cacuuggacauggguuagA..	1	1	seq
..................cacCuggacauggguuagu..	2	1	seq
..................cacuuggacaCggguuagu..	1	1	seq
..................cacuCggacauggguuagu..	1	1	seq
..................cacuuggacaugggCuagu..	1	1	seq
..................cacuuggacauggguuagG..	8	1	seq
..................cacuuggacauggguuagC..	56	1	seq
..................cacuuggacGuggguuagu..	2	1	seq
..................cacuuggacauggguuagu..	364	0	seq
..................cacuuggacauggguCagu..	1	1	seq
..................cacuuggacaCggguuaguc...	1	1	seq
..................cacuuggacauggguuagAc...	1	1	seq
..................cUcuuggacauggguuaguc...	2	1	seq
..................cacuuggacauggguuaguc...	195	0	seq
..................cacuCggacauggguuaguc...	1	1	seq
..................cacuuggCcauggguuaguc...	1	1	seq
..................cacuuggacauggguuagCc...	2	1	seq
..................cacuuggacauggguuGguc...	1	1	seq
..................cacuuggacauggguuagucc..	6	0	seq
..................cacuuggacauggguuagucU..	10	1	seq
..................cacuuggacaugggCuagucc..	1	1	seq
..................cacuuggacauggguuaguccU...	1	1	seq
..................cacuuggacauggguuagucca...	10	0	seq
..................cacuuggacauggguGagucca...	1	1	seq
..................cacuuggacauggguuaguccCu..	1	1	seq
..................cacuuggacauggguuagucUauc...	2	1	seq
..................cacuuggacauggguuagucUaucc..	1	1	seq
..................cacuuggacauggguuagucUauccu...	1	1	seq
..................cacuuggacauggguuagucUauccua..	2	1	seq
..................cacuuggacauggguuagucUauccuaagagaugg..	1	1	seq
...................acuuggacauggguCag...	1	1	seq
...................acGuggacauggguuag...	1	1	seq
...................acuuggacauggguuaA...	7	1	seq
...................acuuggacauggguuaU...	1	1	seq
...................Ncuuggacauggguuag...	1	1	seq
...................acuuggacauggguuag...	128	0	seq
...................acuuggacaugUguuag...	1	1	seq
...................acuuggUcauggguuag...	1	1	seq
...................acuuggacaCggguuagu..	1	1	seq
...................acuuggacauggguuagA..	2	1	seq
...................acuuggacauggguuaAu..	1	1	seq
...................acuCggacauggguuagu..	1	1	seq
...................acuuggacauggguuagC..	35	1	seq
...................acuuggacauggguuagu..	245	0	seq
...................acuuggacGuggguuagu..	1	1	seq
...................acuuggacaugggCuagu..	1	1	seq
...................acAuggacauggguuagu..	1	1	seq
...................acuuggacauggguuagG..	5	1	seq
...................Ccuuggacauggguuagu..	1	1	seq
...................Gcuuggacauggguuagu..	1	1	seq
...................acuuggacauggguuagCc...	3	1	seq
...................acuuggacauggUuuaguc...	1	1	seq
...................acuuggacauggguuaguc...	152	0	seq
...................acCuggacauggguuaguc...	1	1	seq
...................acuuggacauUgguuaguc...	1	1	seq

Mature Star

```
aauguuccaugugaaaaucacuuggacauggguuaguccauccuaagagaugggggaauccuuguaucaaggguguacucugcaugaacaucgaaagauaauuggguuaa
...................acuuggacGuggguuaguc.......................................................................  1    1  seq
...................acAuggacauggguuaguc.......................................................................  1    1  seq
...................acuuggacauggguuagGc.......................................................................  1    1  seq
...................acuuggacauggguuagucc......................................................................  3    0  seq
...................acuuggacauggguuagucU......................................................................  8    1  seq
...................acuuggacauggguuagucca.....................................................................  2    0  seq
...................acuuggacauggguuagucUauccuaagag............................................................  1    1  seq
...................acuuggacauggguuagucUauccuaagagauggg.......................................................  1    1  seq
....................cuuggacauggguCagu........................................................................  1    1  seq
....................cuuggacaugggCuagu........................................................................  3    1  seq
....................cuuggacauggguuGgu........................................................................  3    1  seq
....................cuuggacauggguuagG........................................................................  24   1  seq
....................cuCggacauggguuagu........................................................................  1    1  seq
....................cuuggacauAgguuagu........................................................................  2    1  seq
....................cuuggacaugggAuagu........................................................................  1    1  seq
....................cuugAacauggguuagu........................................................................  1    1  seq
....................cuuggacauggguuagC........................................................................  143  1  seq
....................cuuggacGuggguuagu........................................................................  3    1  seq
....................cuuggacauggguuagu........................................................................  872  0  seq
....................cuGggacauggguuagu........................................................................  1    1  seq
....................cuuggUcauggguuagu........................................................................  1    1  seq
....................cuuggacauggguuagA........................................................................  6    1  seq
....................cuuggacaugAguuaguc.......................................................................  1    1  seq
....................cuuggacauAgguuaguc.......................................................................  1    1  seq
....................cuuggacauggUuuaguc.......................................................................  1    1  seq
....................cuuggacaugggCuaguc.......................................................................  1    1  seq
....................cuuggacauggguuaguc.......................................................................  702  0  seq
....................cuuggacauggguCaguc.......................................................................  1    1  seq
....................cuAggacauggguuaguc.......................................................................  1    1  seq
....................cuuggacGuggguuaguc.......................................................................  1    1  seq
....................cuuggacauggguuagCc.......................................................................  8    1  seq
....................Nuuggacauggguuaguc.......................................................................  3    1  seq
....................cuuggacaugggGuaguc.......................................................................  1    1  seq
....................cuuggacaugCguuaguc.......................................................................  1    1  seq
....................cuuggacauggguuagGc.......................................................................  2    1  seq
....................cuuggacaAggguuaguc.......................................................................  2    1  seq
....................cuCggacauggguuaguc.......................................................................  5    1  seq
....................cCuggacauggguuaguc.......................................................................  1    1  seq
....................cuuggacauggguuagucU......................................................................  28   1  seq
....................cuuggacauggguuagucc......................................................................  26   0  seq
....................cuuggacauggguuagCcc......................................................................  1    1  seq
....................cuuggacauggguuaguUc......................................................................  1    1  seq
....................cuuggacauggguuaguccG.....................................................................  1    1  seq
....................cuuggacauggguuagucca.....................................................................  16   0  seq
....................cuuggacauggguuagucUa.....................................................................  3    1  seq
....................cuuggacauggguuaguccC.....................................................................  2    1  seq
....................cuuggacauggguuGgucca.....................................................................  1    1  seq
....................cuuggacauggguuagucUau....................................................................  5    1  seq
....................cuuggacauggguuaguccCu....................................................................  1    1  seq
....................cuuggacauggguuaguccauc...................................................................  4    0  seq
....................cuuggacauggguuagucUauc...................................................................  6    1  seq
....................cuuggacauggguuaguccaCc...................................................................  1    1  seq
....................cuuggacauggguuaguccaucc..................................................................  2    0  seq
....................cuuggacauggguuagucUaucc..................................................................  1    1  seq
....................cuuggacauggguuaguccauccu.................................................................  3    0  seq
....................cuuggacauggguuagucUauccu.................................................................  5    1  seq
....................cuuggacauggguuaguccauccua................................................................  1    0  seq
....................cuuggacauggguuagucUauccua................................................................  3    1  seq
....................cuuggacauggguuagucUauccuaagagaug.........................................................  1    1  seq
....................cuuggacauggguuaguccauccuaagagaugg........................................................  2    0  seq
....................cuuggacauggguuagucUauccuaagagaugg........................................................  2    1  seq
....................cuuggacauggguuagucUauccuaagagauggg.......................................................  2    1  seq
....................cuuggacauggguuagucUauccuaagagaugggg......................................................  2    1  seq
....................cuuggacauggguuaguccauccuaagagaugggga.....................................................  1    0  seq
.....................uuggacauUgguuaguc.......................................................................  1    1  seq
.....................Guggacauggguuaguc.......................................................................  1    1  seq
.....................Cuggacauggguuaguc.......................................................................  5    1  seq
.....................uuggacGuggguuaguc.......................................................................  2    1  seq
.....................uuggacaugggCuaguc.......................................................................  1    1  seq
.....................uuggacauggguuGguc.......................................................................  1    1  seq
```

```
                    Mature                           Star
aauguuccaugugaaaaucacuuggacauggguuaguccauccuaagagaugggggaauccuuguaucaaggguguacucugcaugaacaucgaaagauaauuggguuaa
.....................Auggacauggguuaguc.......................................................................  1    1  seq
.....................uuggacauggguuaguc.......................................................................  452  0  seq
.....................uuggUcauggguuaguc.......................................................................  1    1  seq
.....................uuggacauggguuagCc.......................................................................  6    1  seq
.....................uuggacauggguCaguc.......................................................................  1    1  seq
.....................uuggacaugUguuaguc.......................................................................  1    1  seq
.....................uuAgacauggguuagucc......................................................................  1    1  seq
.....................uuggacauggguuaguUc......................................................................  2    1  seq
.....................uuggacauggguuagucc......................................................................  17   0  seq
.....................uuggacauggguuaguccа.....................................................................  4    0  seq
.....................uuggacauggguuagucUau....................................................................  6    1  seq
.....................uuggacauggguuagucUauc...................................................................  8    1  seq
.....................uuggacauggguuaguccauc...................................................................  1    0  seq
.....................uuggacauggguuagucUaucc..................................................................  7    1  seq
.....................uuggacauggguuagucUauccu.................................................................  5    1  seq
.....................uuggacauggguuagucUauccua................................................................  1    1  seq
.....................uuggacauggguuagucUauccuaaga.............................................................  1    1  seq
.....................uuggacauggguuaguccauccuaagagau..........................................................  1    0  seq
......................uggacauggguuagucc......................................................................  22   0  seq
......................Gggacauggguuagucca.....................................................................  1    1  seq
......................uggacauggguuagucca.....................................................................  8    0  seq
......................uggacauAgguuagucca.....................................................................  1    1  seq
......................uggacauggguuagucUau....................................................................  2    1  seq
......................uggacauggguuaguccau....................................................................  1    0  seq
......................uggacauggguuagucUauc...................................................................  9    1  seq
......................uggacauggguuaguccauc...................................................................  1    0  seq
......................uggacauggguuagucUaucc..................................................................  2    1  seq
......................uggacauggguuaguccaucc..................................................................  1    0  seq
......................uggacauggguuagucUauccu.................................................................  4    1  seq
......................uggacauggguuagucUauccua................................................................  5    1  seq
......................uggacauggguuaguccauccua................................................................  1    0  seq
......................uggacauggguuagucUauccuaa...............................................................  1    1  seq
......................uggacauggguuagucUauccuaag..............................................................  2    1  seq
......................uggacauggguuagucUauccuaagag............................................................  2    1  seq
......................uggacauggguuagucUauccuaagagaugg........................................................  4    1  seq
......................uggacauggguuaguccauccuaagagaugggga.....................................................  1    0  seq
.......................ggacauggguuaguccGuc...................................................................  1    1  seq
.........................acauggguuaguccauc...................................................................  3    0  seq
.........................acauggguuaguccauU...................................................................  1    1  seq
.........................acauggguuaguccaucc..................................................................  1    0  seq
.........................acauggguuagucUauccuaagagauggg.......................................................  1    1  seq
.........................acauggguuagucUauccuaagagaugggga.....................................................  1    1  seq
..........................cauggguuaguccaucc..................................................................  2    0  seq
............................uggguuaguccauccuaagagauggg.......................................................  1    0  seq
.............................ggguuagucUauccuaagagaugggga.....................................................  1    1  seq
..............................gguuaguccauccuaagagaug.........................................................  2    0  seq
..............................gguuaguccauccuaagagaugggga.....................................................  1    0  seq
...............................guuagucUauccuaagagauggg.......................................................  1    1  seq
.................................uagucUauccuaagagaugggg......................................................  1    1  seq
.......................................auccuaagagauggggaa....................................................  2    0  seq
.......................................auccuaagagauggggaaAccu................................................  1    1  seq
........................................uccuaagagauggggaa....................................................  2    0  seq
........................................uccuaagagauggggaaAccu................................................  3    1  seq
.........................................ccuaagagauggggaaAccu................................................  5    1  seq
..........................................cuaagagauggggaaCcc.................................................  1    1  seq
..........................................cuaagagauggggaaAccu................................................  8    1  seq
...........................................uaagagauggggaaAccu................................................  3    1  seq
...........................................uaagagauggggaaCccu................................................  1    1  seq
...........................................uaagagauggggaauccCug..............................................  1    1  seq
...........................................uaagagauggggaaAccuugua............................................  1    1  seq
............................................aagagauggggaaAccu................................................  4    1  seq
................................................................aucaUggguguacucug............................  1    1  seq
..............................................................................cugcGugaacaucgaaa..............  1    1  seq
...............................................................................ugcGugaacaucgaaag.............  2    1  seq
```

Provisional ID	:	MA_128665_21455
Score total	:	1.8
Score for star read(s)	:	-1.3
Score for read counts	:	0
Score for mfe	:	1.5
Score for randfold	:	1.6
Score for cons. seed	:	
Total read count	:	36184
Mature read count	:	36184
Loop read count	:	0
Star read count	:	0

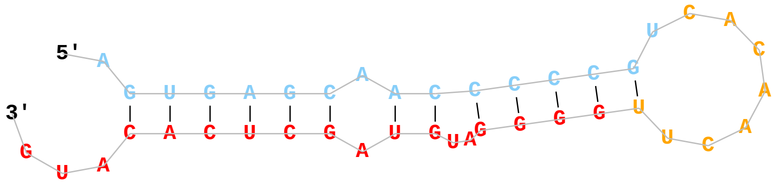

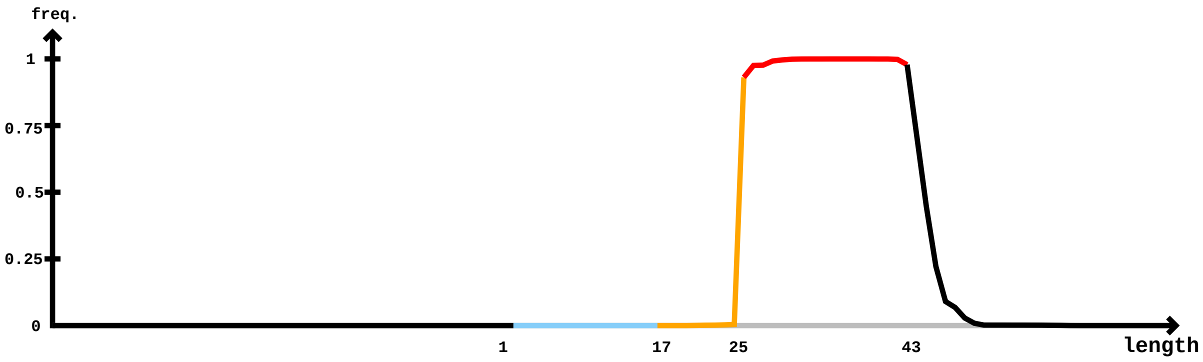

Star Mature

```
5'- uggcauuuuacugagcaaccaccgucacaagacuacucugaaauuuagugagcaacccccgucacaacuuggggauguagcucacaugguagagugcucgcuuugcaugcgggag  -3'    exp
    .(((((((((((...........(((....)))..............((((((.(((((((........)))))..)).))))))..)))))))))))(((.......)))....   reads     mm        sample
    ................................................................Aaacuuggggauguagcucaca.............................   1         1         seq
    .................................................................aaUuuggggauguagcuc................................   1         1         seq
    .................................................................aacuuUgggauguagcuca...............................   1         1         seq
    .................................................................aacuCggggauguagcucac..............................   1         1         seq
    .................................................................aacuuUgggauguagcucaca.............................   2         1         seq
    .................................................................aacuCggggauguagcucaca.............................   3         1         seq
    .................................................................aacuuggggauguagcucacG.............................   1         1         seq
    .................................................................aaAuuggggauguagcucaca.............................   2         1         seq
    .................................................................aacuCggggauguagcucacaug...........................   4         1         seq
    .................................................................aaGuuggggauguagcucacauggua........................   2         1         seq
    .................................................................aacuCggggauguagcucacauggua........................   1         1         seq
    .................................................................aacuCggggauguagcucacaugguagag.....................   1         1         seq
    .................................................................aacuuggggauguagcucacaugguagagCgcucgcuuu...........   1         1         seq
    .................................................................aacuCggggauguagcucacaugguagagugcucgcuuu...........   1         1         seq
    ..................................................................acuuggggauguagcuca...............................   2         0         seq
    ..................................................................acuCggggauguagcuca...............................   1         1         seq
    ..................................................................acuuggggauguagcucaca.............................   3         0         seq
    ..................................................................acuugUggauguagcucaca.............................   1         1         seq
    ..................................................................aGuuggggauguagcucacaug...........................   1         1         seq
    ..................................................................acuuggggauguagcucacaug...........................   1         0         seq
    ..................................................................acuugUggauguagcucacaug...........................   1         1         seq
    ..................................................................acuugUggauguagcucacaugg..........................   1         1         seq
    ..................................................................acuuggggauguagcucacauggC.........................   1         1         seq
    ..................................................................acuuggggauguagcucacauggu.........................   1         0         seq
    ..................................................................acuuggggauguagcucacaugguagagCg...................   1         1         seq
    ..................................................................acuuggggauguagcucacaugguagagCgcucgcu.............   2         1         seq
    ..................................................................acuuggggauguagcucacaugguagagCgcucgcuu............   5         1         seq
    ..................................................................aUuuggggauguagcucacaugguagagugcucgcuuu...........   1         1         seq
    ..................................................................acuuggggauguagcucacaugguagagCgcucgcuuu...........   5         1         seq
    ...................................................................cuuUgggauguagcuca...............................   1         1         seq
    ...................................................................cuuggggauguagcuca...............................   3         0         seq
    ...................................................................cuugUggauguagcucaca.............................   2         1         seq
    ...................................................................cuugUggauguagcucacaug...........................   1         1         seq
    ...................................................................cuuggggauguagcucacaug...........................   1         0         seq
```

Star Mature

uggcauuuuacugagcaaccaccgucacaagacuacucugaaauuuagugagcaaccccgucacaacuuggggauguagcucacaugguagagugcucgcuuugcaugcgggag			
....Guuggggauguagcucacaug...........................	1	1	seq
....cuugUggauguagcucacaugg..........................	1	1	seq
....Auuggggauguagcucacaugguagagugcucgcu.............	1	1	seq
....uuggggauguagcucacG.............................	1	1	seq
....uuggggauguGgcucaca.............................	1	1	seq
....uuggggauguagcucaca.............................	17	0	seq
....uGggggauguagcucaca.............................	2	1	seq
....uuggggauguagcucacGug...........................	1	1	seq
....uuggggauguagcucacaCg...........................	1	1	seq
....uuggggaCguagcucacaug...........................	1	1	seq
....uuggggauguagcucacaug...........................	5	0	seq
....uuggggauguagcucacauC...........................	1	1	seq
....uuggggauguagcucacaCgg..........................	1	1	seq
....uuggggauguagcucacaCggu.........................	1	1	seq
....uuggggauguagcucacauggC.........................	1	1	seq
....uuggggauguagcucacaugUua........................	1	1	seq
....uuggggauguagcucacaugguagagugcucg...............	1	0	seq
....uuggggauguagcucacaugguagagCgcucgcuu............	1	1	seq
....uggggauguagcuUaca.............................	1	1	seq
....Aggggauguagcucaca.............................	2	1	seq
....Gggggauguagcucaca.............................	1	1	seq
....ugUggauguagcucaca.............................	1	1	seq
....uggggauguagcucaca.............................	25	0	seq
....uggggaugCagcucacaug...........................	1	1	seq
....Gggggauguagcucacaug...........................	2	1	seq
....uggggCuguagcucacaug...........................	1	1	seq
....uggggauguagcucacaug...........................	9	0	seq
....uggggauguagcucacGug...........................	1	1	seq
....uggggauguagcucacaugg..........................	2	0	seq
....uggggauguagcucacauggC.........................	1	1	seq
....uggggauguagcucacaugUu.........................	4	1	seq
....uggggaugAagcucacauggu.........................	1	1	seq
....uggggauguagcucacauggu.........................	1	0	seq
....Aggggauguagcucacauggua........................	1	1	seq
....uggggauguagcucacauggua........................	3	0	seq
....Gggggauguagcucacauggua........................	1	1	seq
....Aggggauguagcucacaugguag.......................	1	1	seq
....uggggauguagcucacaugguaga......................	1	0	seq
....uggggauguagcucacaugguagagugcucgcu.............	1	0	seq
....uggggaugCagcucacaugguagagugcucgcuu............	1	1	seq
....ggggauguagcucacaA............................	19	1	seq
....ggggauguagcuUacau............................	1	1	seq
....ggggaCguagcucacau............................	3	1	seq
....ggggauguagcucGcau............................	12	1	seq
....ggggauguagcucacaG............................	25	1	seq
....ggggauguagcucacCu............................	8	1	seq
....gUggauguagcucacau............................	1	1	seq
....ggggaAguagcucacau............................	1	1	seq
....ggggauguagcucUcau............................	1	1	seq
....ggggauguagcucacau............................	548	0	seq
....ggggauguagcCcacau............................	3	1	seq
....ggggauguagcucacGu............................	114	1	seq
....ggggauguagcucacUu............................	1	1	seq
....ggggauguagcucUcaug...........................	2	1	seq
....ggggaugGagcucacaug...........................	1	1	seq
....ggggauguagcucacaGg...........................	39	1	seq
....gUggauguagcucacaug...........................	6	1	seq
....ggggauguagcucacauC...........................	32	1	seq
....ggggauguagcucacUug...........................	4	1	seq
....ggggauguagcucacGug...........................	1295	1	seq
....ggAgauguagcucacaug...........................	9	1	seq
....ggggauguagcucacaCg...........................	994	1	seq
....ggggaCguagcucacaug...........................	15	1	seq
....ggggauguagcucGcaug...........................	27	1	seq
....ggggauguagcucCcaug...........................	2	1	seq
....Ngggauguagcucacaug...........................	12	1	seq
....ggggaugAagcucacaug...........................	5	1	seq
....ggggaAguagcucacaug...........................	1	1	seq
....ggggCuguagcucacaug...........................	1	1	seq
....ggggauguagcucacauA...........................	417	1	seq

```
                                                              Star                   Mature
uggcauuuuacugagcaaccaccgucacaagacuacucugaaauuuagugagcaacccccgucacaacuuggggauguagcucacaugguagagugcucgcuuugcaugcgggag
......................................................................ggggauguagcucacCug...........................	16	1	seq
......................................................................ggggauguagcCcacaug...........................	35	1	seq
......................................................................ggggauguaAcucacaug...........................	11	1	seq
......................................................................ggggauguagcAcacaug...........................	3	1	seq
......................................................................ggggauguagcuGacaug...........................	3	1	seq
......................................................................Cgggauguagcucacaug...........................	5	1	seq
......................................................................ggggaGguagcucacaug...........................	3	1	seq
......................................................................ggggauguGgcucacaug...........................	35	1	seq
......................................................................ggggauguUgcucacaug...........................	4	1	seq
......................................................................ggggauguagcuUacaug...........................	5	1	seq
......................................................................ggggauguagcucacaAg...........................	18	1	seq
......................................................................gggUauguagcucacaug...........................	3	1	seq
......................................................................ggggauguagcuAacaug...........................	3	1	seq
......................................................................ggggUuguagcucacaug...........................	1	1	seq
......................................................................ggggauguagcucacaug...........................	9353	0	seq
......................................................................ggggaugCagcucacaug...........................	12	1	seq
......................................................................ggggauguagAucacaug...........................	2	1	seq
......................................................................ggggauguagcucacauU...........................	39	1	seq
......................................................................ggggauguagUucacaug...........................	13	1	seq
......................................................................ggggauUuagcucacaug...........................	3	1	seq
......................................................................ggggauguagGucacaug...........................	1	1	seq
......................................................................gggAauguagcucacaug...........................	2	1	seq
......................................................................ggggauguagcucGcaugg..........................	16	1	seq
......................................................................ggggauguagcucacaAgg..........................	1	1	seq
......................................................................ggggauguagcucacaugU..........................	99	1	seq
......................................................................ggggauguagcCcacaugg..........................	12	1	seq
......................................................................ggggaugAagcucacaugg..........................	6	1	seq
......................................................................ggggauguagcucacGugg..........................	232	1	seq
......................................................................ggggauguagcucCcaugg..........................	2	1	seq
......................................................................ggggCuguagcucacaugg..........................	1	1	seq
......................................................................ggggauguagcucacauAg..........................	24	1	seq
......................................................................ggggauguagcucacCugg..........................	1	1	seq
......................................................................ggggauguagcucacauUg..........................	28	1	seq
......................................................................ggggauguagcucacUugg..........................	3	1	seq
......................................................................Cgggauguagcucacaugg..........................	2	1	seq
......................................................................ggggauguagcuUacaugg..........................	2	1	seq
......................................................................ggggauguagcucacaugC..........................	51	1	seq
......................................................................gggUauguagcucacaugg..........................	3	1	seq
......................................................................ggggauguaAcucacaugg..........................	8	1	seq
......................................................................gCggauguagcucacaugg..........................	3	1	seq
......................................................................ggggauguUgcucacaugg..........................	4	1	seq
......................................................................ggggauguagcucacaugg..........................	5383	0	seq
......................................................................ggggauguagcucacauCg..........................	2	1	seq
......................................................................ggggUuguagcucacaugg..........................	2	1	seq
......................................................................ggggauguagcAcacaugg..........................	2	1	seq
......................................................................ggggauguagGucacaugg..........................	2	1	seq
......................................................................ggggaCguagcucacaugg..........................	15	1	seq
......................................................................ggggauguagcucUcaugg..........................	3	1	seq
......................................................................ggggauguaCcucacaugg..........................	2	1	seq
......................................................................ggggaugCagcucacaugg..........................	7	1	seq
......................................................................gggAauguagcucacaugg..........................	4	1	seq
......................................................................ggggauguagcucacaGgg..........................	13	1	seq
......................................................................ggAgauguagcucacaugg..........................	2	1	seq
......................................................................Ngggauguagcucacaugg..........................	7	1	seq
......................................................................ggggauUuagcucacaugg..........................	2	1	seq
......................................................................ggggauguagUucacaugg..........................	9	1	seq
......................................................................ggggauguGgcucacaugg..........................	14	1	seq
......................................................................ggggauguagcucacaCgg..........................	460	1	seq
......................................................................ggggauguagcuGacaugg..........................	1	1	seq
......................................................................gggAauguagcucacauggu.........................	1	1	seq
......................................................................ggggauguagcucacaugUu.........................	6	1	seq
......................................................................ggggauguagcucacauAgu.........................	7	1	seq
......................................................................ggggauguaCcucacauggu.........................	1	1	seq
......................................................................ggggauguagcucacauggu.........................	5727	0	seq
......................................................................ggggauguagcucacaCggu.........................	284	1	seq
......................................................................ggggauguagcCcacauggu.........................	19	1	seq
......................................................................gggUauguagcucacauggu.........................	1	1	seq
......................................................................ggggauguagcuUacauggu.........................	3	1	seq
......................................................................ggggauguagcucacCuggu.........................	2	1	seq
......................................................................ggAgauguagcucacauggu.........................	6	1	seq
```

```
                                                              Star                    Mature
uggcauuuuacugagcaaccaccgucacaagacuacucugaaauuuagugagcaaccccgucacaacuuggggauguagcucacaugguagagugcucgcuuugcaugcgggag
.....................................................................ggggauguagcucacauggC.........................  1021  1  seq
.....................................................................ggggauUuagcucacauggu.........................  6     1  seq
.....................................................................ggggaugCagcucacauggu.........................  9     1  seq
.....................................................................ggggauguagcuGacauggu.........................  1     1  seq
.....................................................................ggggauguagcucacGuggu.........................  198   1  seq
.....................................................................ggggauguagcucGcauggu.........................  18    1  seq
.....................................................................ggggauguagUucacauggu.........................  8     1  seq
.....................................................................Ngggauguagcucacauggu.........................  8     1  seq
.....................................................................ggggauguUgcucacauggu.........................  2     1  seq
.....................................................................ggggaAguagcucacauggu.........................  1     1  seq
.....................................................................ggggauguGgcucacauggu.........................  13    1  seq
.....................................................................ggggaugAagcucacauggu.........................  1     1  seq
.....................................................................gUggauguagcucacauggu.........................  4     1  seq
.....................................................................ggggaCguagcucacauggu.........................  13    1  seq
.....................................................................ggggauguagcucUcauggu.........................  3     1  seq
.....................................................................ggggaugGagcucacauggu.........................  1     1  seq
.....................................................................ggggauguagcucacauCgu.........................  1     1  seq
.....................................................................ggggauguagAucacauggu.........................  4     1  seq
.....................................................................ggggauguagcucacaGggu.........................  4     1  seq
.....................................................................ggggauguagcucacauggG.........................  192   1  seq
.....................................................................ggggauguagcucacUuggu.........................  3     1  seq
.....................................................................ggggauguagcAcacauggu.........................  4     1  seq
.....................................................................ggUgauguagcucacauggu.........................  2     1  seq
.....................................................................ggggauguagcucacauggA.........................  101   1  seq
.....................................................................ggggauguagcucacaugCu.........................  9     1  seq
.....................................................................ggggauCuagcucacauggu.........................  1     1  seq
.....................................................................ggggaGguagcucacauggu.........................  1     1  seq
.....................................................................ggggauguagcucacaAggu.........................  1     1  seq
.....................................................................ggggauguagGucacauggu.........................  2     1  seq
.....................................................................ggggauguaAcucacauggu.........................  5     1  seq
.....................................................................ggggauguagcucacauggGa........................  22    1  seq
.....................................................................ggggaugGagcucacauggua........................  1     1  seq
.....................................................................ggggauguagcucacaugguU........................  21    1  seq
.....................................................................ggggaGguagcucacauggua........................  1     1  seq
.....................................................................ggggaCguagcucacauggua........................  16    1  seq
.....................................................................ggggauguagcucacaAggua........................  1     1  seq
.....................................................................ggggauguagcucacauggua........................  3735  0  seq
.....................................................................ggggauguagcucacCuggua........................  2     1  seq
.....................................................................ggggauguagcucacaCggua........................  144   1  seq
.....................................................................gUggauguagcucacauggua........................  8     1  seq
.....................................................................ggggauguagcuUacauggua........................  2     1  seq
.....................................................................ggggauguagcucacUuggua........................  1     1  seq
.....................................................................ggggauguagcucacaugguG........................  72    1  seq
.....................................................................ggggauguagcucGcauggua........................  12    1  seq
.....................................................................ggggauguaAcucacauggua........................  3     1  seq
.....................................................................ggggCuguagcucacauggua........................  1     1  seq
.....................................................................ggggauguGgcucacauggua........................  11    1  seq
.....................................................................ggggauguagcCcacauggua........................  16    1  seq
.....................................................................Ngggauguagcucacauggua........................  5     1  seq
.....................................................................ggggauguagcucacGuggua........................  78    1  seq
.....................................................................ggggauguNgcucacauggua........................  1     1  seq
.....................................................................ggggauguagcucacaugguC........................  3     1  seq
.....................................................................ggggauguagUucacauggua........................  5     1  seq
.....................................................................ggggauguagcucUcauggua........................  1     1  seq
.....................................................................ggggauguagcucacauCgua........................  1     1  seq
.....................................................................ggggauguagcucacauggAa........................  2     1  seq
.....................................................................ggggauguagcucacaGggua........................  4     1  seq
.....................................................................ggggaugCagcucacauggua........................  15    1  seq
.....................................................................ggggauguagAucacauggua........................  2     1  seq
.....................................................................ggggauguagcucacaugUua........................  2     1  seq
.....................................................................ggggauguagcAcacauggua........................  1     1  seq
.....................................................................gggAauguagcucacauggua........................  3     1  seq
.....................................................................ggggauguagcucacauUgua........................  1     1  seq
.....................................................................ggggauguagcucacauggCa........................  58    1  seq
.....................................................................ggggaugAagcucacauggua........................  2     1  seq
.....................................................................ggggauguagcucCcauggua........................  1     1  seq
.....................................................................ggggauguagcCcacaugguag.......................  2     1  seq
.....................................................................ggggauguagcucacaugguag.......................  689   0  seq
.....................................................................ggAgauguagcucacaugguag.......................  1     1  seq
.....................................................................ggggauguUgcucacaugguag.......................  1     1  seq
```

```
                                                              Star                    Mature
uggcauuuuacugagcaaccaccgucacaagacuacucugaaauuuagugagcaacccccgucacaacuuggggauguagcucacaugguagagugcucgcuuugcaugcgggag
......................................................................ggggauguagcucacauggCag.......................  3     1  seq
......................................................................ggggauguagcucacaugguaA.......................  33    1  seq
......................................................................ggggaCguagcucacaugguag.......................  4     1  seq
......................................................................ggggauguagcucacaugguaC.......................  2     1  seq
......................................................................ggggauguagcucacGugguag.......................  15    1  seq
......................................................................ggggauguagcucacaugguCg.......................  1     1  seq
......................................................................ggggauguagcucGcaugguag.......................  1     1  seq
......................................................................ggggauguagcucacauggAag.......................  2     1  seq
......................................................................ggggaugCagcucacaugguag.......................  1     1  seq
......................................................................ggggauguagcucacaugUuag.......................  2     1  seq
......................................................................ggggauguagcucacaugguGg.......................  2     1  seq
......................................................................ggggauguGgcucacaugguag.......................  1     1  seq
......................................................................ggggauguagcucacaCgguag.......................  27    1  seq
......................................................................ggggauguagcucacaugUuaga......................  1     1  seq
......................................................................ggggauguagcucUcaugguaga......................  1     1  seq
......................................................................ggggauguagcucacCugguaga......................  3     1  seq
......................................................................ggggaCguagcucacaugguaga......................  6     1  seq
......................................................................ggggauguaAcucacaugguaga......................  1     1  seq
......................................................................ggggauguagcucGcaugguaga......................  3     1  seq
......................................................................ggUgauguagcucacaugguaga......................  1     1  seq
......................................................................ggggauguagcucacaugguaga......................  1107  0  seq
......................................................................ggggauguagcucacaugguaCa......................  1     1  seq
......................................................................ggggauguagcucacaugguagU......................  8     1  seq
......................................................................ggggauguagcucacaCgguaga......................  40    1  seq
......................................................................ggggauguagcucacGugguaga......................  17    1  seq
......................................................................ggggauguagcucacauggCaga......................  2     1  seq
......................................................................Ngggauguagcucacaugguaga......................  2     1  seq
......................................................................ggggauguGgcucacaugguaga......................  4     1  seq
......................................................................ggggauguagcucacaugguUga......................  2     1  seq
......................................................................ggggUuguagcucacaugguaga......................  1     1  seq
......................................................................ggggaugCagcucacaugguaga......................  2     1  seq
......................................................................ggggauguagAucacaugguaga......................  1     1  seq
......................................................................ggggauguagcuUacaugguaga......................  1     1  seq
......................................................................ggggauguagUucacaugguaga......................  2     1  seq
......................................................................ggggauguagcucacaugguagC......................  3     1  seq
......................................................................ggggauguagcCcacaugguaga......................  3     1  seq
......................................................................ggggauguagcucacaugguagG......................  26    1  seq
......................................................................ggggauguagcucacaugguGga......................  5     1  seq
......................................................................ggggauguUgcucacaugguaga......................  1     1  seq
......................................................................ggggauguagcucacaugguagUg.....................  1     1  seq
......................................................................Agggauguagcucacaugguagag.....................  2     1  seq
......................................................................ggggaAguagcucacaugguagag.....................  1     1  seq
......................................................................ggggaCguagcucacaugguagag.....................  1     1  seq
......................................................................ggggauguagUucacaugguagag.....................  1     1  seq
......................................................................ggggauguGgcucacaugguagag.....................  2     1  seq
......................................................................ggggauguagcucacaugguagaA.....................  18    1  seq
......................................................................ggggauguagcucacGugguagag.....................  6     1  seq
......................................................................ggggauguagcucacaugguagag.....................  626   0  seq
......................................................................ggggauguagcucacaugguagaU.....................  2     1  seq
......................................................................ggggauguagcucacaugguagaC.....................  5     1  seq
......................................................................ggggauguagcucacaugguGgag.....................  2     1  seq
......................................................................Cgggauguagcucacaugguagag.....................  1     1  seq
......................................................................ggggauguagcucacauAguagag.....................  1     1  seq
......................................................................ggggauguagcucacaAgguagag.....................  1     1  seq
......................................................................ggggauguagcucacaugguagGg.....................  2     1  seq
......................................................................ggggauguagcucacaugguagCg.....................  1     1  seq
......................................................................ggggauguagcucacaCgguagag.....................  20    1  seq
......................................................................ggggauguagcucGcaugguagag.....................  1     1  seq
......................................................................ggggauguagcucGcaugguagagu....................  1     1  seq
......................................................................ggggauguagGucacaugguagagu....................  1     1  seq
......................................................................ggggauUuagcucacaugguagagu....................  1     1  seq
......................................................................ggggauguagcucacaugguagGgu....................  1     1  seq
......................................................................ggggauguagcuUacaugguagagu....................  1     1  seq
......................................................................gUggauguagcucacaugguagagu....................  3     1  seq
......................................................................ggggauguagcucacaugguagUgu....................  1     1  seq
......................................................................ggggaCguagcucacaugguagagu....................  1     1  seq
......................................................................Ngggauguagcucacaugguagagu....................  1     1  seq
......................................................................ggggauguagcucacaugguagagu....................  223   0  seq
......................................................................ggggauguagcucacGugguagagu....................  3     1  seq
......................................................................ggggauguagcucacaugguagaAu....................  1     1  seq
```

Star Mature

```
uggcauuuuacugagcaaccaccgucacaagacuacucugaaauuuagugagcaaccccgucacaacuuggggauguagcucacaugguagagugcucgcuuugcaugcgggag
.....................................................................ggggauguagcucacaugguagagG....................	3	1	seq
.....................................................................ggggauguagcucacaCgguagagu....................	4	1	seq
.....................................................................ggggaGguagcucacaugguagagu....................	1	1	seq
.....................................................................Agggauguagcucacaugguagagu....................	1	1	seq
.....................................................................ggggauguagcucacaugguagaguA...................	1	1	seq
.....................................................................ggggauguagcucacaugguagagug...................	1	0	seq
.....................................................................ggggauguagcucacaugguagagugc..................	1	0	seq
.....................................................................ggggauguagcucacaugguagagAgc..................	1	1	seq
.....................................................................ggggauguagcucacaugguagagugcu.................	1	0	seq
.....................................................................ggggauguagcucacaugguagagugcuc................	1	0	seq
.....................................................................ggggauguagcucacaugguagagAgcuc................	1	1	seq
.....................................................................ggggauguagcucacaugguagagugcucg...............	1	0	seq
.....................................................................ggggauguagcucacaugguagaguAcucg...............	1	1	seq
.....................................................................ggggauguagcucacaugguagagAgcucgc..............	1	1	seq
.....................................................................ggggauguagcucacaugguagagAgcucgcu.............	8	1	seq
.....................................................................ggggauguagcucacaugguagagGgcucgcu.............	1	1	seq
.....................................................................ggggauguagcucacaugguagaguAcucgcu.............	1	1	seq
.....................................................................ggggauguagcucacaugguagagugcucgcu.............	1	0	seq
.....................................................................ggggauguagcucacaugguagagAgcucgcuu............	2	1	seq
.....................................................................ggggauguagcucacaugguagagugcucgcuuu...........	1	0	seq
.....................................................................ggggauguagcucacaugguagagAgcucgcuuug..........	1	1	seq
.....................................................................ggggauguagcucacaugguagagugcucgcuuug..........	1	0	seq
.....................................................................ggggauguagcucacaugguagagugcucgcuuugc.........	1	0	seq
......................................................................gggauguagcucacCug...........................	1	1	seq
......................................................................gggauguGgcucacaug...........................	2	1	seq
......................................................................gggaugCagcucacaug...........................	1	1	seq
......................................................................gggauguagcucacaAg...........................	1	1	seq
......................................................................ggUauguagcucacaug...........................	1	1	seq
......................................................................Nggauguagcucacaug...........................	2	1	seq
......................................................................gggauguagcucacaGg...........................	2	1	seq
......................................................................gggauguagcucacauU...........................	3	1	seq
......................................................................gggauguagcucacaug...........................	445	0	seq
......................................................................gggauguagUucacaug...........................	1	1	seq
......................................................................gggauguagcucacauA...........................	20	1	seq
......................................................................ggCauguagcucacaug...........................	1	1	seq
......................................................................gggauguagcucacaCg...........................	57	1	seq
......................................................................gggauguagcCcacaug...........................	1	1	seq
......................................................................gggauguagcucacGug...........................	65	1	seq
......................................................................gggauguUgcucacaug...........................	3	1	seq
......................................................................gggauguagcucacauAg..........................	2	1	seq
......................................................................gggauguagcucacaCgg..........................	18	1	seq
......................................................................gggaCguagcucacaugg..........................	1	1	seq
......................................................................gggauguagcucacaugU..........................	2	1	seq
......................................................................gggauguagcucacaGgg..........................	2	1	seq
......................................................................gggaugCagcucacaugg..........................	2	1	seq
......................................................................gggauguagcucacaugg..........................	250	0	seq
......................................................................gggauguagcucGcaugg..........................	1	1	seq
......................................................................gggauguagcCcacaugg..........................	2	1	seq
......................................................................gggauguagcucacGugg..........................	12	1	seq
......................................................................gggauguagcucacauggG.........................	14	1	seq
......................................................................gggauguagcucacauggu.........................	311	0	seq
......................................................................gggauguagcucacaGggu.........................	1	1	seq
......................................................................gggauguagcucacaugCu.........................	1	1	seq
......................................................................gggaugAagcucacauggu.........................	1	1	seq
......................................................................gggauguagcucacauggC.........................	44	1	seq
......................................................................gggauguagcucacGuggu.........................	7	1	seq
......................................................................gAgauguagcucacauggu.........................	1	1	seq
......................................................................gggauguagcucacauggA.........................	9	1	seq
......................................................................gggauguagcucacaCggu.........................	10	1	seq
......................................................................gggauguagcuUacauggu.........................	1	1	seq
......................................................................gggauguagcAcacauggu.........................	1	1	seq
......................................................................gggauguagcCcacauggu.........................	2	1	seq
......................................................................gggauguagcucacauggGa........................	1	1	seq
......................................................................gggauguagcucacaCggua........................	5	1	seq
......................................................................gggauguagcCcacauggua........................	2	1	seq
......................................................................gggauguagcucacaugguU........................	1	1	seq
......................................................................gggauguagcucacaugUua........................	2	1	seq
......................................................................gggaugCagcucacauggua........................	1	1	seq
......................................................................Uggauguagcucacauggua........................	2	1	seq
......................................................................gggauguagcucacGuggua........................	4	1	seq
```

Star Mature

uggcauuuuacugagcaaccaccgucacaagacuacucugaaauuuagugagcaacccccgucacaacuuggggauguagcucacaugguagagugcucgcuuugcaugcgggag

....gggauguagcucacaugguG........................	2	1	seq
....gggauguagcucacauggCa........................	1	1	seq
....gggauguGgcucacauggua........................	1	1	seq
....gggauguagcucacauggua........................	233	0	seq
....gggauguaAcucacaugguag.......................	1	1	seq
....gggauguagcucacaugguag.......................	15	0	seq
....gggauguagcucacaCgguag.......................	4	1	seq
....gggauguagcucacaugguaA.......................	1	1	seq
....gggauguagcCcacaugguag.......................	1	1	seq
....gggauguagcucacaugguaga......................	41	0	seq
....gggauguagcucacaugguagG......................	1	1	seq
....gggauguagcucacaugguagU......................	1	1	seq
....gggauguagcucacaCgguaga......................	5	1	seq
....Uggauguagcucacaugguaga......................	1	1	seq
....gggauguagcucacGugguaga......................	2	1	seq
....ggAauguagcucacaugguaga......................	1	1	seq
....gggauguagcucacaugguagag.....................	27	0	seq
....gggauguagcucacaCgguagagu....................	1	1	seq
....gggauguagcucacaugguagagu....................	4	0	seq
....gggauguagcucacGugguagagu....................	1	1	seq
....ggauguagcucacaCgg..........................	2	1	seq
....ggauguagcucacaugg..........................	5	0	seq
....ggauguagcucacauggu.........................	14	0	seq
....ggauguagcucacauggC.........................	1	1	seq
....ggauguagcucacGuggu.........................	1	1	seq
....ggauguagcucacauggua........................	12	0	seq
....ggauguagcCcacauggua........................	1	1	seq
....ggauguagcucacaugguaga......................	4	0	seq
....ggauguagcucacaugguagagu....................	1	0	seq
....gauguagcucGcauggu.........................	1	1	seq
....gaugCagcucacauggu.........................	1	1	seq
....gauguGgcucacauggu.........................	1	1	seq
....gauguagcucacaCggu.........................	12	1	seq
....gauguagcucacauggA.........................	2	1	seq
....gauguagcucacauggu.........................	234	0	seq
....gauguaAcucacauggu.........................	2	1	seq
....gauguagcucacGuggu.........................	6	1	seq
....gauguagUucacauggu.........................	1	1	seq
....gauguagcucacauggC.........................	33	1	seq
....gauguGgcucacauggua........................	1	1	seq
....gauguagcucacauggCa........................	1	1	seq
....gauguagcucacaCggua........................	6	1	seq
....gauguagcucacGuggua........................	3	1	seq
....gauguagcucacaugguG........................	3	1	seq
....Uauguagcucacauggua........................	1	1	seq
....gauguagcucacaugguC........................	1	1	seq
....Aauguagcucacauggua........................	1	1	seq
....gauguagcucUcauggua........................	1	1	seq
....gauguagcCcacauggua........................	1	1	seq
....gaAguagcucacauggua........................	1	1	seq
....gauguagcucacauggua........................	228	0	seq
....gaCguagcucacaugguag.......................	1	1	seq
....gauguagcucacGugguag.......................	1	1	seq
....Uauguagcucacaugguag.......................	1	1	seq
....gauguagcucacaugguag.......................	7	0	seq
....gauguagcucacaugguagG......................	1	1	seq
....gauguagcucacaugguagU......................	1	1	seq
....gauguagcucacaugguaga......................	20	0	seq
....gauguagcucacaugguagag.....................	3	0	seq
....gauguagcucacaCgguagag.....................	2	1	seq
....gauguagcucacaugguagagu....................	2	0	seq
....gauguagcucacaugguagagugcucgcuu............	1	0	seq
....auguagcucacaugguU........................	2	1	seq
....aCguagcucacauggua........................	1	1	seq
....auguagcucacGuggua........................	2	1	seq
....auguagcucacaCggua........................	3	1	seq
....auguagcucGcauggua........................	1	1	seq
....auguagcucacauggCa........................	3	1	seq
....auguagcucacauggua........................	96	0	seq
....auguagcucacaCgguag.......................	1	1	seq

Star Mature

uggcauuuuacugagcaaccaccgucacaagacuacucugaaauuuagugagcaacccccgucacaacuuggggauguagcucacauggguagagugcucgcuuugcaugcgggag			
....auguagcucacaugguag.......................	5	0	seq
....auguagcucacaugguaga......................	35	0	seq
....auguagcucacaCgguaga......................	2	1	seq
....auguagcucacauAguaga......................	1	1	seq
....auguagcucacaugguagagugcucgU..............	1	1	seq
....uguagcucacauggGag.......................	1	1	seq
....uguagcucacaugguag.......................	6	0	seq
....uguagcucacauggua A.......................	2	1	seq
....uguagcucacauUguaga......................	1	1	seq
....uguagcucacauCguaga......................	1	1	seq
....uguagcucacauggCaga......................	1	1	seq
....uguagcucacaugguagG......................	5	1	seq
....uguagcucacaugguaga......................	76	0	seq
....uguagcucacaCgguaga......................	2	1	seq
....uguagcucacaugguagag.....................	3	0	seq
....uguagcucacaugguagagugcucgcuuug..........	1	0	seq
....guagcucacaugguaga......................	24	0	seq
....guagcucacaugguagag.....................	1	0	seq
....guagcuUacaugguagag.....................	1	1	seq
....guagcucacaugguagagugcucgc..............	1	0	seq
....uagcucacaugguagag.....................	3	0	seq
....Cagcucacaugguagagugc..................	1	1	seq
....agcucacaugguagagugcucgcuuu...........	1	0	seq
....gcucacaugguagagugcucgcu.............	1	0	seq
....cucacaugguagagugcucg...............	1	0	seq
....cucacaugguagagugcucgcuuug..........	1	0	seq
....augguagagugcucgcuuu...........	2	0	seq
....aAgguagagugcucgcuuu...........	1	1	seq
....ugguagaguUcucgcuu............	1	1	seq
....ugguagagugcucgcuuu...........	7	0	seq

Provisional ID	:	MA_128793_23063
Score total	:	2058.6
Score for star read(s)	:	3.9
Score for read counts	:	2069.5
Score for mfe	:	-12.5
Score for randfold	:	-2.2
Score for cons. seed	:	
Total read count	:	4071
Mature read count	:	4056
Loop read count	:	0
Star read count	:	15

5' 3'

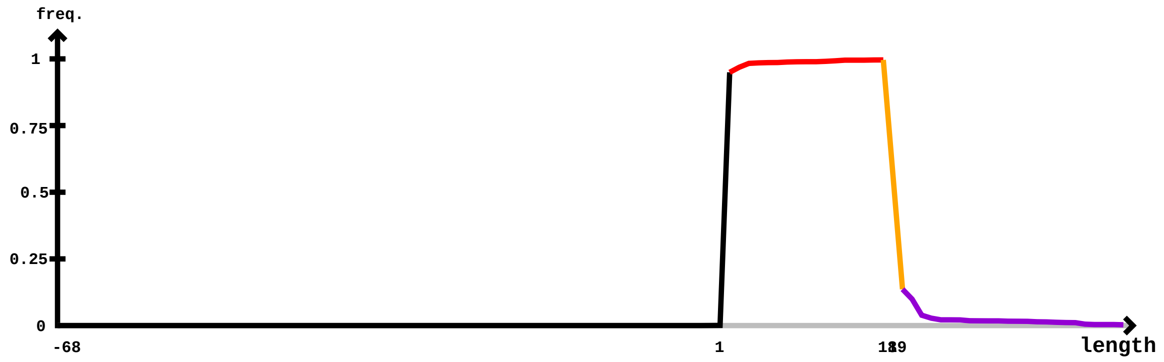

Mature Star

```
5'- uucuuugcauauuccguauuacgagaggaauauaaauaaugaauuuaaaauuaauauuuaaaccaacugacaguuuggccgagugg ucuaaggcgccagauuuaggcucu -3'  obs
    uucuuugcauauuccguauuacgagaggaauauaaauaaugaauuuaaaauuaauauuuaaaccaacugacaguuuggccgaguggucuaaggcgccagauuuaggcucu      exp
    ........(((((((...........)))))))...................................((((..((((((((.((....))...).))))))))))).....   reads  mm  sample
    ..................................................................Augacaguuuggccgagu..........................   1     1   seq
    ...................................................................ugacaguuuggccgaguggucu.....................   1     0   seq
    ....................................................................gacaguuuggccgaAug.........................   2     1   seq
    ....................................................................gacaguuuggccgagAg.........................   5     1   seq
    ....................................................................gacaguuuggccgGgug.........................   9     1   seq
    ....................................................................gacaguuuggccgagCg.........................   12    1   seq
    ....................................................................gacagCuuggccgagug.........................   2     1   seq
    ....................................................................gacaguuuAgccgagug.........................   2     1   seq
    ....................................................................gacaguuuggccgaCug.........................   1     1   seq
    ....................................................................gacaguuCggccgagug.........................   7     1   seq
    ....................................................................gacaguuugUccgagug.........................   3     1   seq
    ....................................................................gacaguuuCgccgagug.........................   1     1   seq
    ....................................................................Aacaguuuggccgagug.........................   2     1   seq
    ....................................................................gacGguuuggccgagug.........................   6     1   seq
    ....................................................................gaUaguuuggccgagug.........................   3     1   seq
    ....................................................................Cacaguuuggccgagug.........................   1     1   seq
    ....................................................................gacaguuuggccgaguC.........................   7     1   seq
    ....................................................................gacaguuuggccgaguU.........................   14    1   seq
    ....................................................................gacaguuuggccAagug.........................   1     1   seq
    ....................................................................gacaguuuggccgUgug.........................   3     1   seq
    ....................................................................gaAaguuuggccgagug.........................   2     1   seq
    ....................................................................gacaguuuggccgagGg.........................   5     1   seq
    ....................................................................gacaguuuggccCagug.........................   1     1   seq
    ....................................................................gacaguuugCccgagug.........................   1     1   seq
    ....................................................................gacUguuuggccgagug.........................   1     1   seq
    ....................................................................gacaguuuggccUagug.........................   2     1   seq
    ....................................................................Nacaguuuggccgagug.........................   3     1   seq
    ....................................................................gacaguuuggccgCgug.........................   1     1   seq
    ....................................................................gacaguCuggccgagug.........................   8     1   seq
    ....................................................................gacaguuuggUcgagug.........................   2     1   seq
    ....................................................................gacaguuuggccgagug.........................   1868  0   seq
    ....................................................................gacaguuuggGcgagugg........................   1     1   seq
    ....................................................................gacaguCuggccgagugg........................   4     1   seq
```

```
                                                                     Mature   Star
uucuuugcauauuccguauuacgagaggaauauaaauaaugaauuuaaaauuaauauuuaaaccaacugacaguuuggccgaguggucuaaggcgccagauuuaggcucu
....................................................................gacaguuuggccAagugg.........................  1     1  seq
....................................................................gacagGuuggccgagugg.........................  1     1  seq
....................................................................gacaguuuggccgagugC.........................  14    1  seq
....................................................................gacaguuuggccgagugg.........................  1337  0  seq
....................................................................Uacaguuuggccgagugg.........................  1     1  seq
....................................................................gacaguuuggccgaguCg.........................  1     1  seq
....................................................................gacaguuuggccgagugA.........................  106   1  seq
....................................................................gacaguuuAgccgagugg.........................  2     1  seq
....................................................................gacaguuuggccgagAgg.........................  2     1  seq
....................................................................gUcaguuuggccgagugg.........................  2     1  seq
....................................................................gacaguuuggcAgagugg.........................  1     1  seq
....................................................................gacaguuuggccgCgugg.........................  1     1  seq
....................................................................gacaguuCggccgagugg.........................  1     1  seq
....................................................................gaUaguuuggccgagugg.........................  3     1  seq
....................................................................gacaguuuggccgagugU.........................  17    1  seq
....................................................................gacaguuuggccgaguUg.........................  3     1  seq
....................................................................gacaAuuuggccgagugg.........................  1     1  seq
....................................................................gacaguuuggccgagCgg.........................  3     1  seq
....................................................................gacaguuuggccgGgugg.........................  4     1  seq
....................................................................Nacaguuuggccgagugg.........................  3     1  seq
....................................................................gacaguuAggccgagugg.........................  1     1  seq
....................................................................gacaguuuggUcgagugg.........................  1     1  seq
....................................................................gacaguuuggccUagugg.........................  1     1  seq
....................................................................gacGguuuggccgagugg.........................  3     1  seq
....................................................................gacaguuuggccgUgugg.........................  2     1  seq
....................................................................gacaguuuggccgagGgg.........................  5     1  seq
....................................................................Aacaguuuggccgagugg.........................  1     1  seq
....................................................................gacUguuuggccgagugg.........................  1     1  seq
....................................................................gacagAuuggccgagugg.........................  1     1  seq
....................................................................gacagCuuggccgagugg.........................  1     1  seq
....................................................................gacaguuugCccgaguggu........................  1     1  seq
....................................................................gacaguuuggccgaguggC........................  20    1  seq
....................................................................gacaguuuggccgagugCu........................  1     1  seq
....................................................................gacaguuuggccgaguggA........................  2     1  seq
....................................................................gacaguuuggccgaguggu........................  114   0  seq
....................................................................gacaguuugUccgaguggu........................  1     1  seq
....................................................................gacaguuuggccgaguggG........................  2     1  seq
....................................................................gacGguuuggccgaguggu........................  1     1  seq
....................................................................gacaguuuggccgaguggCc.......................  1     1  seq
....................................................................gacaguCuggccgagugguc.......................  2     1  seq
....................................................................gacaguuugCccgagugguc.......................  1     1  seq
....................................................................gacaguuuggccgagugguA.......................  5     1  seq
....................................................................gacaguuugUccgagugguc.......................  1     1  seq
....................................................................gacaguuuggccgagugguc.......................  184   0  seq
....................................................................gacaguuuggUcgagugguc.......................  1     1  seq
....................................................................gacaguuuggccgagugAuc.......................  1     1  seq
....................................................................gacaguuuggGcgagugguc.......................  1     1  seq
....................................................................gacaguuuggccgaguggucG......................  2     1  seq
....................................................................gacaguuuggccgaguggAcu......................  1     1  seq
....................................................................gacaguuuggccgaguggucA......................  2     1  seq
....................................................................gacaguuuggccgaguggucC......................  4     1  seq
....................................................................gacaguuuggccgagugCucu......................  1     1  seq
....................................................................gacaguuuggccUaguggucu......................  1     1  seq
....................................................................gacaguuuggccgaguggucu......................  35    0  seq
....................................................................gacaguuuggccgaguggCcu......................  1     1  seq
....................................................................gacaguuuggccgaguUgucua.....................  1     1  seq
....................................................................gacaguuuggccgaguggucCa.....................  1     1  seq
....................................................................gacaguuuggccgaguggucua.....................  20    0  seq
....................................................................gacaguuuggccgaguggucuaa....................  1     0  seq
....................................................................gacaguuuggccgaguggucuaag...................  1     0  seq
....................................................................gacaguuuggccgaguggucuaagg..................  12    0  seq
....................................................................gacaguuuggccgaguggucuaagA..................  1     1  seq
....................................................................gacaguuuggcAgaguggucuaaggc.................  1     1  seq
....................................................................gacaguuuggccgaguggucuaaggcgcca.............  1     0  seq
....................................................................gacaguuuggccgagCggucuaaggcgccag............  1     1  seq
....................................................................gacaguuuggccgaguggucuaaggcgccaga...........  2     0  seq
....................................................................gacaguuuggccgaguggucuaaggcgccagau..........  1     0  seq
....................................................................gacaguuuggccgaguggucuaaggcUccagauu.........  1     1  seq
....................................................................gacaguuuggccgaguggucuaaggcgccagauu.........  1     0  seq
....................................................................gacaguuuggccgaguggucuaaggcgccagauuuaggcucA  1     1  seq
```

Mature Star

```
uucuuugcauauuccguauuacgagaggaauauaaauaaugaauuuaaaauuaaauauuuaaaccaacugacaguuuggccgaguggucuaaggcgccagauuuaggcucu
.....................................................................gacaguuuggccgaguggucuaaggcgccagauuuaggcucC	1	1	seq
.....................................................................gacaguuuggccgaguggucuaaggcgccagauuuaggcucu	1	0	seq
......................................................................acaguuuggccgagugg........................	58	0	seq
......................................................................acUguuuggccgagugg........................	1	1	seq
......................................................................acaguuuggccgagugA........................	7	1	seq
......................................................................Ucaguuuggccgagugg........................	1	1	seq
......................................................................acaguuuggccgagugU........................	1	1	seq
......................................................................acaguuuggccgaguggu.......................	7	0	seq
......................................................................acaguuuggccgagugguc......................	3	0	seq
......................................................................acaguuuggccgaguggucua....................	1	0	seq
.......................................................................caguuuggccgaguggC.......................	1	1	seq
.......................................................................caguuuggccgaguggu.......................	5	0	seq
.......................................................................caguAuggccgagugguc......................	2	1	seq
.......................................................................caguuuggccgagugguc......................	46	0	seq
.......................................................................caguuuggccgaguggucua....................	5	0	seq
.......................................................................caguuuggccgaguggucuaaggcgccagauuuaggcuU.	1	1	seq
........................................................................aguuuggccgagugguc......................	5	0	seq
........................................................................aguuuggccgaAugguc......................	1	1	seq
........................................................................aguuuggccgaguggucuaaggcgccagauuuag.....	1	0	seq
........................................................................aguuuggccgaguggucuaaggcgccagauuuaggcucu	1	0	seq
.........................................................................guuuggccgaguggucua....................	1	0	seq
.........................................................................guuuggccgaguggucuaaggcg...............	1	0	seq
.........................................................................guuuggccUaguggucuaaggcgcc.............	1	1	seq
.........................................................................guuuggccgaguggucuaaggcgccagauuuag.....	1	0	seq
..........................................................................uuuggccgaguggucuaaggcgcU.............	1	1	seq
...........................................................................uuggccgaguggucuaaggcgccaga..........	1	0	seq
...........................................................................uuggccgaguggucuaaggcUccaga..........	1	1	seq
...........................................................................uuggccgaguggucuaaggcgccagauuua......	1	0	seq
...........................................................................uuggccgaguggucuaaggcgccagauuuag.....	4	0	seq
...........................................................................uuggccgaguggucuaaggcgccagauuuagU....	1	1	seq
............................................................................uggccgaguggucuaaggcgcc.............	1	0	seq
............................................................................uggccgaguggucuaaggcgccagauu........	1	0	seq
............................................................................uggccgaguggucuaaggcgccagauuuag.....	2	0	seq
.............................................................................ggccgaguggucuaaggcgccagauuuag.....	1	0	seq
...............................................................................ccgaguggucuaaggcgcU.............	1	1	seq
...............................................................................ccgaguggucuaaggcgcc.............	1	0	seq
...............................................................................ccgaguggucuaaggcgccaga..........	1	0	seq
...............................................................................ccgaguggucuaaggcgccagauuu.......	2	0	seq
...............................................................................ccgaguggucuaaggcgccagauuuag.....	1	0	seq
................................................................................cgaguggucuaaggcgccagau.........	1	0	seq
................................................................................cgaguggucuaaggcgccagauu........	1	0	seq
................................................................................cgaguggucuaaggcgccagauuua......	1	0	seq
................................................................................cgaguggucuaaggcgccagauuuag.....	2	0	seq
................................................................................cgaguggucuaGggcgccagauuuagg....	1	1	seq
................................................................................cgaguggucuaaggcgccagauuuagA....	1	1	seq
................................................................................cgaguggucuaaggcgccagauuuaggcuU.	1	1	seq
.................................................................................gaguggucuaaggcgccaga..........	1	0	seq
.................................................................................gagugguc Aaggcgccagauuu.......	1	1	seq
.................................................................................gaguggucuaaggAgccagauuuag.....	1	1	seq
.................................................................................gaguggucuaaggcgccagauuuag.....	6	0	seq
.................................................................................gaguggucuaaggcgccagauuuagg....	1	0	seq
....................................................................................uggucuaaggcgcGaga..........	1	1	seq
....................................................................................uggucuaaggcgcGagauu........	1	1	seq
....................................................................................uggucuaaggcgccagauuuag.....	1	0	seq
.....................................................................................ggucuaaggcgccagauu........	1	0	seq
.....................................................................................ggucuaaggcgccagauuGag.....	1	1	seq
.......................................................................................ucuaaggcgccagaCuu.......	1	1	seq
.......................................................................................ucuaaggcgcGagauuuaggcucu	3	1	seq
........................................................................................cuaaggcgcGagauuuaggcucu	1	1	seq
.........................................................................................uaaggcgccagauuuag.....	1	0	seq
.........................................................................................uaaCgcgccagauuuag.....	1	1	seq
.........................................................................................uaaggcgccagauuuagA....	1	1	seq
.........................................................................................uaaggcAccagauuuagg....	1	1	seq
..........................................................................................aaggcgcGagauuuaggcuc.	1	1	seq
..........................................................................................aaggcgcGagauuuaggcucu	1	1	seq
...........................................................................................aggcgcGagauuuaggcucu	2	1	seq
...........................................................................................aggcgAcagauuuaggcucu	1	1	seq
............................................................................................ggcgcGagauuuaggcuc.	1	1	seq
```

Provisional ID : MA_128838_19713
Score total : 97.7
Score for star read(s) : 3.9
Score for read counts : 95.5
Score for mfe : 0.5
Score for randfold : -2.2
Score for cons. seed :
Total read count : 199
Mature read count : 194
Loop read count : 0
Star read count : 5

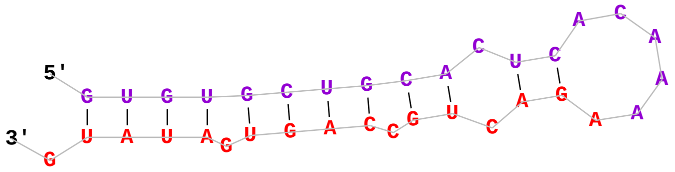

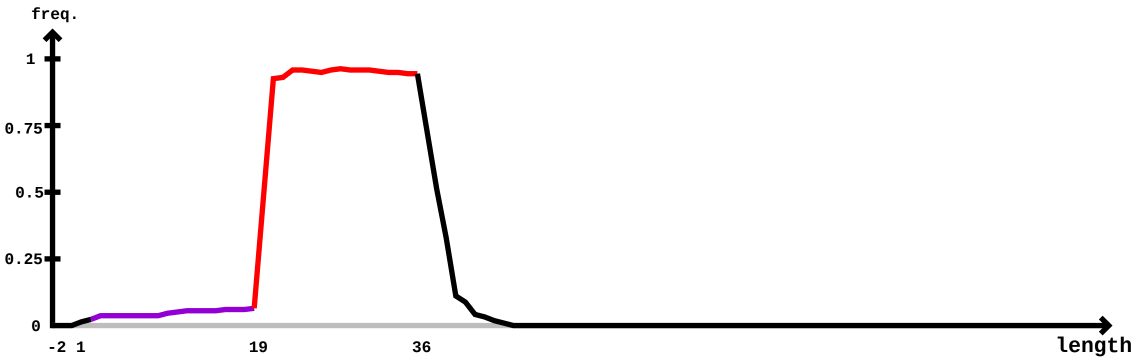

Star Mature

```
5'- gugugugcugcacucacaaaagacugccagugauaugcuggaggaaagcuuguucuguccuuccgugcaucucugugauggguaaauuuccaugagaaaggggugacu -3'  obs
    gugugugcugcacucacaaaagacugccagugauaugcuggaggaaagcuuguucuguccuuccgugcaucucugugauggguaaauuuccaugagaaaggggugacu       exp
    ((((((((((((.((......)).)).)))).)))))).((((((.((......)).)))))).((.(((((((.(.(((((......))))).)...))))))))).   reads  mm  sample
    .ugCgugcugcacucacaaaa.......................................................................................   1      1   seq
    .ugugugcugcacucacaaGagacug..................................................................................   1      1   seq
    .ugugugcugcacucacaaGagacugcc................................................................................   1      1   seq
    ..gCgugcugcacucacaaa........................................................................................   2      1   seq
    ...Cgugcugcacucacaaa........................................................................................   2      1   seq
    ...ugugcugcacucacaaGagacu...................................................................................   1      1   seq
    ..........cacucacaaUagacugccagug............................................................................   1      1   seq
    ..........cacucacaaaagacugccaguga...........................................................................   1      0   seq
    ...........acucacaaUagacugcca...............................................................................   1      1   seq
    ............cucacaaUagacugccagugaua.........................................................................   1      1   seq
    ................caaaagacugccagugauaugcug....................................................................   1      0   seq
    ...................aagacugccagugauaugc......................................................................   1      0   seq
    ....................agacugccagugauauA.......................................................................   1      1   seq
    ....................Ngacugccagugauaug.......................................................................   1      1   seq
    ....................agacugUcagugauaug.......................................................................   1      1   seq
    ....................agacugccagugauaug.......................................................................   58     0   seq
    ....................aUacugccagugauaug.......................................................................   1      1   seq
    ....................agacugccagugauaugc......................................................................   27     0   seq
    ....................agacugccagugauGugc......................................................................   1      1   seq
    ....................Ggacugccagugauaugc......................................................................   1      1   seq
    ....................agacugccagugGuaugcu.....................................................................   1      1   seq
    ....................agacugccagugauaugcu.....................................................................   30     0   seq
    ....................agacugccagugauaugcC.....................................................................   6      1   seq
    ....................agacugccGgugauaugcu.....................................................................   1      1   seq
    ....................agacugccagCgauaugcu.....................................................................   1      1   seq
    ....................agacugccagugauaugcGg....................................................................   1      1   seq
    ....................agacugccagugauaugcug....................................................................   40     0   seq
    ....................agacugccagugauaugcugg...................................................................   2      0   seq
    ....................agacugccagugauaugcugga..................................................................   4      0   seq
    ....................agacugccagugauaugcuggag.................................................................   1      0   seq
    ....................agacugccagugauaugcuggagg................................................................   1      0   seq
    ....................agacugccagugauaugcuggagga...............................................................   1      0   seq
    .....................gacugccagugauaugc......................................................................   1      0   seq
```

Star **Mature**

```
gugugugcugcacucacaaaagacugccagugauaugcuggaggaaagcuuguucuguccuuccgugcaucucugugauggguaaauuuccaugagaaaggggugacu
.....................gacugccagugauaugcC.....................................................................   1   1   seq
.....................gacugccagugauaugcug....................................................................   4   0   seq
.....................gacugccagugauaugcugg...................................................................   1   0   seq
.....................gacugccagugauaugcugga..................................................................   2   0   seq
.....................gacugccagugauaugcuggag.................................................................   1   0   seq
.....................gacugccagugauaugcuggagga...............................................................   1   0   seq
......................acugccagugauaCgcug....................................................................   1   1   seq
.......................cugccagugaCaugcugg...................................................................   1   1   seq
.......................cugccagugauaugcugg...................................................................   1   0   seq
.......................cugccagugauaugcugga..................................................................   4   0   seq
...........................cagugauaugcuggagg................................................................   2   0   seq
............................agugauaugcuggaggaa..............................................................   2   0   seq
```

Provisional ID	:	MA_129978_20848
Score total	:	1.5
Score for star read(s)	:	-1.3
Score for read counts	:	0
Score for mfe	:	1.2
Score for randfold	:	1.6
Score for cons. seed	:	
Total read count	:	23847
Mature read count	:	23842
Loop read count	:	0
Star read count	:	5

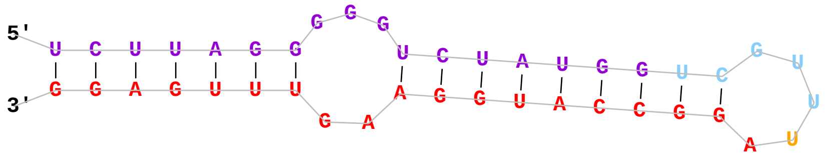

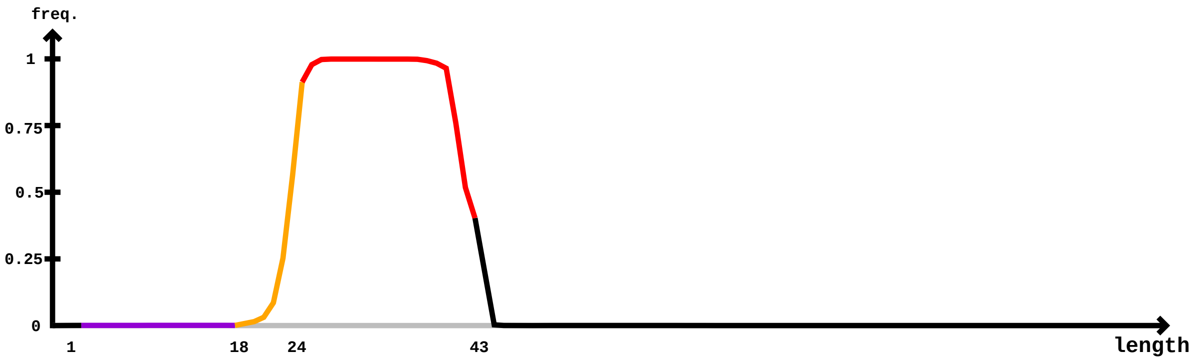

Star **Mature**

```
5'- uucuuaggggucuauggucguuuaggccauggaaguuugaggaaauaauagguaugugaugcucuuacauguucuaggccacaugcaugcuacauugauguaguaaaaaaggc   -3'   obs
    uucuuaggggucuauggucguuuaggccauggaaguuugaggaaauaauagguaugugaugcucuuacauguucuaggccacaugcaugcuacauugauguaguaaaaaaggc         exp
    ((((((((...(((((((((.....)))))))))..))))))))........((((((..((.((..........)))))))))).(((((((....)))))))........    reads      mm      sample
    uucuuagggggAcuaug................................................................................................  6          1       seq
    uucuuagggggAcuaugg...............................................................................................  4          1       seq
    .ucuuagAgggucuaugg...............................................................................................  1          1       seq
    .ucuuagggggAcuaugg...............................................................................................  3          1       seq
    .......gggucuauggCcguuua.........................................................................................  1          1       seq
    .......gggucuauggCcguuuaggccaug..................................................................................  1          1       seq
    .......gggucuauggCcguuuaggccauggaaguuug..........................................................................  1          1       seq
    .......gggucuauggCcguuuaggccauggaaguuugagg.......................................................................  1          1       seq
    ..............uggucguuuaggccauggaaguuugagg.......................................................................  1          0       seq
    ................gCcguuuaggccauggaaguuugagga......................................................................  1          1       seq
    .................ucguuuaggccauggaagC.............................................................................  1          1       seq
    .................ucguuuaggccauggaaguuug..........................................................................  1          0       seq
    .................Ncguuuaggccauggaaguuugag........................................................................  1          1       seq
    .................Ncguuuaggccauggaaguuugagg.......................................................................  1          1       seq
    .................Ccguuuaggccauggaaguuugagga......................................................................  4          1       seq
    ..................cguuuaggccauggaag..............................................................................  17         0       seq
    ..................cguuuagCccauggaagu.............................................................................  1          1       seq
    ..................cguuuaggccauggaagu.............................................................................  55         0       seq
    ..................cguuuaggccauggaagC.............................................................................  6          1       seq
    ..................cguuuaggccauggaagG.............................................................................  2          1       seq
    ..................cguuuaggccauggGaguu............................................................................  1          1       seq
    ..................cguuuaggccauggaaguu............................................................................  45         0       seq
    ..................cguuuaggccauggaaguC............................................................................  7          1       seq
    ..................cguuuaggccauggaaguG............................................................................  1          1       seq
    ..................cguuuaggccGuggaaguu............................................................................  1          1       seq
    ..................cguCuaggccauggaaguuu...........................................................................  1          1       seq
    ..................cguuuaggccauggaaguuC...........................................................................  8          1       seq
    ..................cguuuaggccauggaaguuu...........................................................................  33         0       seq
    ..................cgCuuaggccauggaaguuug..........................................................................  1          1       seq
    ..................cguuuaggccauggaagCuug..........................................................................  1          1       seq
    ..................cguuuaggccauggaaguuuA..........................................................................  2          1       seq
    ..................cUuuuaggccauggaaguuug..........................................................................  1          1       seq
    ..................cguuuaggccauggaaguuug..........................................................................  62         0       seq
```

Star **Mature**

```
uucuuaggggucuauggucguuuaggccauggaaguuugaggaaauaaauagguaugugaugcucuuacauguucuaggccacaugcaugcuacauugauguaguaaaaaaggc
..................cguuuaggUcauggaaguuug........................................................................... 1 1 seq
..................cguuuaggccauggaaguuGga.......................................................................... 1 1 seq
..................cguuuaggccauggaaguuuga.......................................................................... 38 0 seq
..................cguuuaggccauggaaguuugag......................................................................... 26 0 seq
..................cguuuaggccauggaGguuugag......................................................................... 1 1 seq
..................cguuuaggccauggaaguuugaA......................................................................... 1 1 seq
..................cguuuaggccauggaaguuugagA........................................................................ 3 1 seq
..................cguuuaggccauggaaguuugagg........................................................................ 49 0 seq
..................cUuuuaggccauggaaguuugagg........................................................................ 1 1 seq
..................cguuuaggccauggaaguuugaggU....................................................................... 5 1 seq
..................cguuuaggccauggaaguuugagga....................................................................... 1 0 seq
...................gNuuaggccauggaagu.............................................................................. 1 1 seq
...................guuuaggccauggaagu.............................................................................. 61 0 seq
...................guuuaggccauggaagA.............................................................................. 1 1 seq
...................guuuaggccauggaagC.............................................................................. 10 1 seq
...................guuuaggccauggaagCu............................................................................. 1 1 seq
...................guuuaggccauggaaguu............................................................................. 50 0 seq
...................guuuaggccGuggaaguu............................................................................. 1 1 seq
...................Nuuuaggccauggaaguu............................................................................. 1 1 seq
...................guuuaUgccauggaaguu............................................................................. 1 1 seq
...................guuuaggccauggaaguG............................................................................. 1 1 seq
...................guuuaggccauggaagGu............................................................................. 1 1 seq
...................guuuaggccauggaaguuu............................................................................ 36 0 seq
...................guuuaggccauggaaguuC............................................................................ 2 1 seq
...................guuuaggccauggaaguuuA........................................................................... 1 1 seq
...................guuuaAgccauggaaguuug........................................................................... 1 1 seq
...................guuuaggccauggaaguuug........................................................................... 23 0 seq
...................guuuaggccauggaaguuuU........................................................................... 1 1 seq
...................guuuaggccauggaUguuuga.......................................................................... 1 1 seq
...................guuuaggccauggaaguuugG.......................................................................... 1 1 seq
...................guuuaggccauggaaguuugU.......................................................................... 1 1 seq
...................guCuaggccauggaaguuuga.......................................................................... 2 1 seq
...................guuuaggcUauggaaguuuga.......................................................................... 1 1 seq
...................guuuaggccauggaaguuuga.......................................................................... 49 0 seq
...................guuuaggccGuggaaguuuga.......................................................................... 1 1 seq
...................guuuaggccauggaaguuugag......................................................................... 64 0 seq
...................guuuaggccauggaUguuugag......................................................................... 1 1 seq
...................guuuaggccauggaaguuugGg......................................................................... 1 1 seq
...................guuuaggccauggaaguuugagA........................................................................ 4 1 seq
...................guuuaggccauggaaguuugGgg........................................................................ 1 1 seq
...................guuuaggccauggaaguuugagU........................................................................ 1 1 seq
...................guuuaggccauggaaguuugagg........................................................................ 89 0 seq
...................guuuaggccauggaGguuugagg........................................................................ 1 1 seq
...................Uuuuaggccauggaaguuugagg........................................................................ 2 1 seq
...................guuuaggccauggaaguCugagg........................................................................ 1 1 seq
...................guuuaggccauggaaguuugagga....................................................................... 6 0 seq
...................guuuaggccauggaaguuugaggG....................................................................... 3 1 seq
...................guuuaggccauggaaguuugaggaa...................................................................... 1 0 seq
...................guuuaggccauggaaguuugaggaaC..................................................................... 1 1 seq
....................uuuaggccauggaaguG............................................................................. 2 1 seq
....................uuuaggccauggaaguu............................................................................. 127 0 seq
....................uuuaggccaCggaaguu............................................................................. 1 1 seq
....................uuuaggccauggaagAu............................................................................. 2 1 seq
....................uuuaggccauggaagGu............................................................................. 1 1 seq
....................uuCaggccauggaaguu............................................................................. 1 1 seq
....................uuuGggccauggaaguu............................................................................. 1 1 seq
....................Auuaggccauggaaguu............................................................................. 1 1 seq
....................uuuaggcUauggaaguu............................................................................. 1 1 seq
....................uuuaggccauggaaguAu............................................................................ 1 1 seq
....................uuuaggccauggaaguCu............................................................................ 2 1 seq
....................uuuaggccauggGaguuu............................................................................ 1 1 seq
....................uuuaggccauggaaguuu............................................................................ 81 0 seq
....................uuuaggccauggaaguuC............................................................................ 5 1 seq
....................uuuaggccauggUaguuu............................................................................ 1 1 seq
....................uuuaggccGuggaaguuu............................................................................ 1 1 seq
....................uuuaggccaAggaaguuug........................................................................... 1 1 seq
....................uuCaggccauggaaguuug........................................................................... 1 1 seq
....................uuuaggccauggaaguuuA........................................................................... 11 1 seq
....................uuuaggccauggGaguuug........................................................................... 4 1 seq
....................uuuGggccauggaaguuug........................................................................... 1 1 seq
```

Star Mature

```
uucuuagggggucuauggucguuuaggccauggaaguuugaggaaauaaauagguaugugaugcucuuacauguucuaggccacaugcaugcuacauugauguaguaaaaaaggc
.....................uuuaggccauggaGguuug...........................................................................  1     1  seq
.....................Cuuaggccauggaaguuug...........................................................................  2     1  seq
.....................uuuaggccauggaaguuCg...........................................................................  1     1  seq
.....................uuuaggccauggaaguuug...........................................................................  390   0  seq
.....................Guuaggccauggaaguuug...........................................................................  1     1  seq
.....................uuuaggcUauggaaguuug...........................................................................  2     1  seq
.....................uuuaggccauggaaguuuC...........................................................................  1     1  seq
.....................uuuaggccGuggaaguuuga..........................................................................  1     1  seq
.....................Cuuaggccauggaaguuuga..........................................................................  1     1  seq
.....................uuuaggccauggaaguuugG..........................................................................  12    1  seq
.....................uuuaggccaCggaaguuuga..........................................................................  1     1  seq
.....................uuCaggccauggaaguuuga..........................................................................  1     1  seq
.....................uuuaggccauggaaguuuga..........................................................................  327   0  seq
.....................uuuaggccauggGaguuuga..........................................................................  1     1  seq
.....................uuuaggccauggaaguuugU..........................................................................  2     1  seq
.....................uuuaggccauggaagCuuga..........................................................................  1     1  seq
.....................uuuaggccauggaaguuugaC.........................................................................  1     1  seq
.....................Auuaggccauggaaguuugag.........................................................................  1     1  seq
.....................uuuaggccUuggaaguuugag.........................................................................  1     1  seq
.....................uuuaggccauggaGguuugag.........................................................................  2     1  seq
.....................uuuaggccauggaaguuugag.........................................................................  197   0  seq
.....................uuuaggccauggGaguuugag.........................................................................  1     1  seq
.....................uuuaggccauggaaguuugGg.........................................................................  2     1  seq
.....................Cuuaggccauggaaguuugag.........................................................................  1     1  seq
.....................uuCaggccauggaaguuugagg........................................................................  1     1  seq
.....................uuuaggccauggaagCuugagg........................................................................  1     1  seq
.....................uuuaggccaCggaaguuugagg........................................................................  1     1  seq
.....................uuuaggcGauggaaguuugagg........................................................................  1     1  seq
.....................uuuaggccaGggaaguuugagg........................................................................  1     1  seq
.....................Guuaggccauggaaguuugagg........................................................................  1     1  seq
.....................uuuaggccauggaaguuugagC........................................................................  1     1  seq
.....................uuuaggccauggGaguuugagg........................................................................  1     1  seq
.....................uuuagUccauggaaguuugagg........................................................................  2     1  seq
.....................uuuaggccauggaaguuugUgg........................................................................  1     1  seq
.....................uuuaggccauggaaguuugagA........................................................................  15    1  seq
.....................uuuaggccauggaaguuugagg........................................................................  179   0  seq
.....................uuuaggccauggaaguuugagU........................................................................  3     1  seq
.....................uuuaggccauggaaguuugaggG.......................................................................  3     1  seq
.....................uuuaggccauggaaguuugagga.......................................................................  6     0  seq
.....................uuuaggccauggCaguuugagga.......................................................................  1     1  seq
.....................uuuaggccauggaaguuugaggaa......................................................................  2     0  seq
......................uuaggccauggaaguuG............................................................................  2     1  seq
......................uuaggccauggaGguuu............................................................................  2     1  seq
......................uCaggccauggaaguuu............................................................................  1     1  seq
......................uuaggccauggUaguuu............................................................................  1     1  seq
......................uuaggccauggGaguuu............................................................................  1     1  seq
......................uuaggccauggaaguuu............................................................................  285   0  seq
......................uuaggccauggaaguCu............................................................................  1     1  seq
......................uuaggccUuggaaguuu............................................................................  1     1  seq
......................uuaggccauggaaguuC............................................................................  29    1  seq
......................uuaggccaAggaaguuu............................................................................  1     1  seq
......................uuaggccauggaaguuAg...........................................................................  1     1  seq
......................Nuaggccauggaaguuug...........................................................................  2     1  seq
......................uuaggccauggaaguuuA...........................................................................  21    1  seq
......................uuaggccauggaaguGug...........................................................................  1     1  seq
......................uuaggccaAggaaguuug...........................................................................  1     1  seq
......................uuaggccauCgaaguuug...........................................................................  1     1  seq
......................uuaggccauggGaguuug...........................................................................  4     1  seq
......................uuaggccauggaGguuug...........................................................................  7     1  seq
......................uuaggccaugAaaguuug...........................................................................  1     1  seq
......................uuaggccauggaaguuCg...........................................................................  3     1  seq
......................uGaggccauggaaguuug...........................................................................  1     1  seq
......................uuaggccauggaaguuug...........................................................................  1344  0  seq
......................uuCggccauggaaguuug...........................................................................  1     1  seq
......................uuaggccauggaaguCug...........................................................................  4     1  seq
......................uuaggccauggaaguuuC...........................................................................  1     1  seq
......................uuaggccauggaaguuuU...........................................................................  3     1  seq
......................uuGggccauggaaguuug...........................................................................  5     1  seq
......................uuagUccauggaaguuug...........................................................................  1     1  seq
......................uuaggccGuggaaguuug...........................................................................  5     1  seq
```

Star **Mature**

uucuuaggggucuauggucguuuaggccauggaaguuugaggaaauaaauagguaugugaugcucuuacauguucuaggccacaugcaugcuacauugauguaguaaaaaaggc

.....................uuaggccaCggaaguuug...	3	1	seq
.....................uuaggccauggaagCuug...	2	1	seq
.....................uuaggccauUgaaguuug...	2	1	seq
.....................uCaggccauggaaguuug...	1	1	seq
.....................uuaggcUauggaaguuug...	2	1	seq
.....................uuaggccaCggaaguuuga..	1	1	seq
.....................uuaggccauggaaguCuga..	2	1	seq
.....................uuUggccauggaaguuuga..	1	1	seq
.....................uuaggccauggaaguuugG..	24	1	seq
.....................uuaggccauggaaguuuCa..	2	1	seq
.....................Nuaggccauggaaguuuga..	2	1	seq
.....................uuaggccauggaUguuuga..	1	1	seq
.....................uuaggccauggaGguuuga..	2	1	seq
.....................uuaggccauggaaguuugU..	11	1	seq
.....................uuGggccauggaaguuuga..	5	1	seq
.....................uuaggccaGggaaguuuga..	1	1	seq
.....................uuaggccauggaaguuuAa..	1	1	seq
.....................uuaggccauggaagCuuga..	3	1	seq
.....................uuaggccauggaaguuCga..	1	1	seq
.....................uuaggccGuggaaguuuga..	3	1	seq
.....................Cuaggccauggaaguuuga..	2	1	seq
.....................Auaggccauggaaguuuga..	2	1	seq
.....................uuaggccauggaagGuuga..	2	1	seq
.....................uuaggccauggGaguuuga..	7	1	seq
.....................Guaggccauggaaguuuga..	1	1	seq
.....................uuaggccauggaaguuGga..	1	1	seq
.....................uuaggccaAggaaguuuga..	2	1	seq
.....................uuCggccauggaaguuuga..	1	1	seq
.....................uuaggccauggaaguuuga..	1104	0	seq
.....................uuaggccauggaGguuugag...	1	1	seq
.....................uuaggccaCggaaguuugag...	1	1	seq
.....................uuGggccauggaaguuugag...	3	1	seq
.....................uuaggccauggaaguuugGg...	3	1	seq
.....................uuagUccauggaaguuugag...	1	1	seq
.....................Auaggccauggaaguuugag...	1	1	seq
.....................uuaggccauggaagCuugag...	1	1	seq
.....................uuaggccauggaaguuugag...	308	0	seq
.....................uuaggccauggaaguuCgag...	3	1	seq
.....................Nuaggccauggaaguuugag...	2	1	seq
.....................uuaggccauggaaguuugGgg..	9	1	seq
.....................uuaggccauggaaguuugUgg..	2	1	seq
.....................uuagUccauggaaguuugagg..	1	1	seq
.....................uuaggccauggaaguuugagA..	55	1	seq
.....................uuaggccauggaaguuugaCg..	2	1	seq
.....................uCaggccauggaaguuugagg..	1	1	seq
.....................uuaggccaCggaaguuugagg..	2	1	seq
.....................uuGggccauggaaguuugagg..	2	1	seq
.....................uuaggccauggCaguuugagg..	1	1	seq
.....................uuaggccauggaaguuuUagg..	1	1	seq
.....................uuaggccGuggaaguuugagg..	5	1	seq
.....................uuaggccauggGaguuugagg..	3	1	seq
.....................uuaggccaAggaaguuugagg..	1	1	seq
.....................uuaggccauggaagCuugagg..	1	1	seq
.....................Cuaggccauggaaguuugagg..	3	1	seq
.....................uuaggccauggaaguuugagU..	8	1	seq
.....................uuaggccauUgaaguuugagg..	1	1	seq
.....................uuaggccauggaaguCugagg..	2	1	seq
.....................uuaggccauggaaguuCgagg..	3	1	seq
.....................uuaggccauggaaguuugaUg..	1	1	seq
.....................uuaggccauggaagGuugagg..	2	1	seq
.....................Guaggccauggaaguuugagg..	3	1	seq
.....................uuaggccauggaaguuugagC..	3	1	seq
.....................uuaggccauggaaguuugagg..	955	0	seq
.....................uuaggccauggaGguuugagg..	1	1	seq
.....................uuaggccauggaaguuugaggG...	8	1	seq
.....................uuaggccauggaaguuugagCa...	1	1	seq
.....................uuaggccauggaaguuugagga...	35	0	seq
.....................uuaggccauggaaguuugGggaa..	1	1	seq
.....................uuaggccauggaaguuugaggGa..	1	1	seq
.....................uuaggccauggaaguuugaggaG..	2	1	seq

Star **Mature**

```
uucuuaggggucuauggucguuuaggccauggaaguuugaggaaauaaauagguaugugaugcucuuacauguucuaggccacaugcaugcuacauuugauguaguaaaaaaggc
.....................uuaggccGuggaaguuugaggaa.......................................................................    1     1    seq
.....................uuaggccauggaaguuugaggaa.......................................................................    4     0    seq
.....................uuaggccauggaaguuugaggGaa......................................................................    1     1    seq
.....................uuaggccauggaaguuugaggaaaua....................................................................    1     0    seq
......................Naggccauggaaguuug............................................................................    5     1    seq
......................uaggccauggaaguuug............................................................................    3268  0    seq
......................uaggccauggaaguGug............................................................................    3     1    seq
......................uUggccauggaaguuug............................................................................    4     1    seq
......................uCggccauggaaguuug............................................................................    1     1    seq
......................uaggccauggaaguuuC............................................................................    3     1    seq
......................uaggGcauggaaguuug............................................................................    1     1    seq
......................uaggccauggaUguuug............................................................................    1     1    seq
......................uGggccauggaaguuug............................................................................    8     1    seq
......................uaggccauggaaguuuA............................................................................    64    1    seq
......................uaggccauggaagGuug............................................................................    1     1    seq
......................uaggccauggaaguAug............................................................................    2     1    seq
......................uaggccauggaagCuug............................................................................    6     1    seq
......................uaggccauggaaguuCg............................................................................    13    1    seq
......................uaggccauggaaguuuU............................................................................    2     1    seq
......................uaggccaAggaaguuug............................................................................    1     1    seq
......................uaggccaCggaaguuug............................................................................    7     1    seq
......................uaggccauggaaguCug............................................................................    8     1    seq
......................uaggccauggUaguuug............................................................................    1     1    seq
......................uaggccauggaaguuAg............................................................................    3     1    seq
......................uaggccauggaagAuug............................................................................    3     1    seq
......................uaggccauggaaguuGg............................................................................    3     1    seq
......................uaggcUauggaaguuug............................................................................    5     1    seq
......................uaggccauggaGguuug............................................................................    10    1    seq
......................uaggccauggaUguuuga...........................................................................    1     1    seq
......................uUggccauggaaguuuga...........................................................................    2     1    seq
......................uaggccauggaaguuuga...........................................................................    1613  0    seq
......................uaggccaCggaaguuuga...........................................................................    3     1    seq
......................uagUccauggaaguuuga...........................................................................    3     1    seq
......................uaggcUauggaaguuuga...........................................................................    1     1    seq
......................uaggccauggCaguuuga...........................................................................    2     1    seq
......................uaggccauggaaguuAga...........................................................................    1     1    seq
......................uaggccauggaaguuCga...........................................................................    4     1    seq
......................uaggccaAggaaguuuga...........................................................................    1     1    seq
......................uaggccauggaGguuuga...........................................................................    3     1    seq
......................uaggccauggaagCuuga...........................................................................    6     1    seq
......................uaggccauggaaguCuga...........................................................................    1     1    seq
......................uGggccauggaaguuuga...........................................................................    2     1    seq
......................uaggccauggaaguuGga...........................................................................    1     1    seq
......................uUggccauggaaguuugag..........................................................................    1     1    seq
......................uaggccaCggaaguuugag..........................................................................    2     1    seq
......................uaggccauggaaguuugaC..........................................................................    1     1    seq
......................uaggccauggaGguuugag..........................................................................    1     1    seq
......................uaggccaugUaaguuugag..........................................................................    1     1    seq
......................uaggccauggaaguCugag..........................................................................    3     1    seq
......................uaggccauggaagCuugag..........................................................................    1     1    seq
......................uaggccauggaaguuuCag..........................................................................    2     1    seq
......................uGggccauggaaguuugag..........................................................................    2     1    seq
......................uaggccauggaaguuCgag..........................................................................    2     1    seq
......................uaggccauggaaguuugUg..........................................................................    2     1    seq
......................uaggcUauggaaguuugag..........................................................................    1     1    seq
......................uaggccauggaaguAugag..........................................................................    1     1    seq
......................uaggccauggaaguuugag..........................................................................    574   0    seq
......................uaggccauggaagGuugag..........................................................................    1     1    seq
......................uaggccauggGaguuugag..........................................................................    5     1    seq
......................uaggcUauggaaguuugagg.........................................................................    1     1    seq
......................uaggccauggaaguCugagg.........................................................................    2     1    seq
......................uaggccauggaaguuuAagg.........................................................................    1     1    seq
......................uaggccauggaaguuCgagg.........................................................................    8     1    seq
......................uaggccauggaagAuugagg.........................................................................    1     1    seq
......................uaggccauggGaguuugagg.........................................................................    4     1    seq
......................uaggccauggaGguuugagg.........................................................................    8     1    seq
......................uaggccauggaCguuugagg.........................................................................    1     1    seq
......................uaggccauUgaaguuugagg.........................................................................    1     1    seq
......................uGggccauggaaguuugagg.........................................................................    5     1    seq
......................uaggccauggaaguuugagC.........................................................................    7     1    seq
```

Star **Mature**

```
uucuuagggggucuaugggucguuuaggccauggaaguuugaggaaauaauagguaugugaugcucuuacauguucuaggccacaugcaugcuacauugauguaguaaaaaaggc
.......................uaggccauggaaguuugUgg.......................................................................   2      1   seq
.......................uaggccauggaaguuugagU.......................................................................   18     1   seq
.......................uaggccauggUaguuugagg.......................................................................   2      1   seq
.......................Naggccauggaaguuugagg.......................................................................   3      1   seq
.......................uaggccaCggaaguuugagg.......................................................................   4      1   seq
.......................uaggccauggaaguuugagA.......................................................................   137    1   seq
.......................uaggccauggaaguGugagg.......................................................................   1      1   seq
.......................uagUccauggaaguuugagg.......................................................................   2      1   seq
.......................uaggccaAggaaguuugagg.......................................................................   2      1   seq
.......................uaggccauggaaguuugaUg.......................................................................   1      1   seq
.......................uCggccauggaaguuugagg.......................................................................   1      1   seq
.......................uaggccauggaaguuugaCg.......................................................................   1      1   seq
.......................uaggccauggaaguuugagg.......................................................................   2045   0   seq
.......................uaggccaugUaaguuugagg.......................................................................   2      1   seq
.......................uaggccauggaagCuugagg.......................................................................   5      1   seq
.......................uaggccauggaaguuugagCa......................................................................   2      1   seq
.......................uaggccauggGaguuugagga......................................................................   1      1   seq
.......................uaggccauggaaguuugagga......................................................................   129    0   seq
.......................uaggcUauggaaguuugagga......................................................................   1      1   seq
.......................uaggccauggaaguuugUgga......................................................................   1      1   seq
.......................uaggccaCggaaguuugagga......................................................................   2      1   seq
.......................uaggccauggaaguuugaggG......................................................................   10     1   seq
.......................uaggccauggaaguuugaggaG.....................................................................   2      1   seq
.......................uaggccauggaaguuugaggGa.....................................................................   10     1   seq
.......................uaggccauggaaguuugaggaC.....................................................................   1      1   seq
.......................uaggccauggaaguuugaggaa.....................................................................   5      0   seq
.......................uaggccauggaaguuugaggaaau...................................................................   1      0   seq
.......................uaggccauggaaguuugaggaaaua..................................................................   1      0   seq
........................Cggccauggaaguuuga.........................................................................   1      1   seq
........................Nggccauggaaguuuga.........................................................................   4      1   seq
........................aggccaugAaaguuuga.........................................................................   2      1   seq
........................aggccauggaaguuGga.........................................................................   3      1   seq
........................aggccauggaaguuuga.........................................................................   2992   0   seq
........................aggccUuggaaguuuga.........................................................................   2      1   seq
........................aggccauggaGguuuga.........................................................................   10     1   seq
........................aCgccauggaaguuuga.........................................................................   1      1   seq
........................aggccaGggaaguuuga.........................................................................   1      1   seq
........................aggccauggaaguCuga.........................................................................   12     1   seq
........................aggccauggaUguuuga.........................................................................   1      1   seq
........................aggccauggaaguuCga.........................................................................   5      1   seq
........................agUccauggaaguuuga.........................................................................   1      1   seq
........................aggccauggaaguuuCa.........................................................................   1      1   seq
........................aggccauggaaguuuAa.........................................................................   2      1   seq
........................aggccauggaagGuuga.........................................................................   4      1   seq
........................aggccauggaaguAuga.........................................................................   1      1   seq
........................aggcGauggaaguuuga.........................................................................   1      1   seq
........................aggccaCggaaguuuga.........................................................................   2      1   seq
........................aggccCuggaaguuuga.........................................................................   1      1   seq
........................aggccauggaagAuuga.........................................................................   1      1   seq
........................aggccauggCaguuuga.........................................................................   1      1   seq
........................aggccauggUaguuuga.........................................................................   1      1   seq
........................aggccauggGaguuugag........................................................................   2      1   seq
........................aggccUuggaaguuugag........................................................................   3      1   seq
........................aggccauggaaguuugaU........................................................................   2      1   seq
........................aggccaugUaaguuugag........................................................................   1      1   seq
........................aggccauggaGguuugag........................................................................   3      1   seq
........................aggccauggaaguCugag........................................................................   1      1   seq
........................aggccauggaaguuCgag........................................................................   2      1   seq
........................Uggccauggaaguuugag........................................................................   1      1   seq
........................aggccauggaaguuugag........................................................................   1308   0   seq
........................aggccauggUaguuugag........................................................................   2      1   seq
........................aggccauggaaguuuCag........................................................................   3      1   seq
........................aggccaCggaaguuugag........................................................................   1      1   seq
........................aggccauggaaguuuAag........................................................................   1      1   seq
........................Nggccauggaaguuugag........................................................................   3      1   seq
........................aggcUauggaaguuugag........................................................................   2      1   seq
........................aggcGauggaaguuugag........................................................................   1      1   seq
........................aggccauggaaguuuUagg.......................................................................   1      1   seq
........................aggcUauggaaguuugagg.......................................................................   5      1   seq
........................aggccaCggaaguuugagg.......................................................................   7      1   seq
```

Star **Mature**

```
uucuuagggggucuauggucguuuaggccauggaaguuugaggaaauaaauagguaugugaugcucuuacauguucuaggccacaugcaugcuacauugauguaguaaaaaaggc
........................Nggccauggaaguuugagg........................................................................ 5 1 seq
........................aggccaAggaaguuugagg........................................................................ 1 1 seq
........................aggccUuggaaguuugagg........................................................................ 1 1 seq
........................aggccauggaaguuGgagg........................................................................ 3 1 seq
........................Uggccauggaaguuugagg........................................................................ 3 1 seq
........................aggccauggaaguuAgagg........................................................................ 2 1 seq
........................Gggccauggaaguuugagg........................................................................ 14 1 seq
........................aggccauggaUguuugagg........................................................................ 1 1 seq
........................aggccauggaaguCugagg........................................................................ 1 1 seq
........................aggccauggaaguuugCgg........................................................................ 2 1 seq
........................aggccauggaaguuugaCg........................................................................ 5 1 seq
........................aggccauggaaguuugaUg........................................................................ 3 1 seq
........................agUccauggaaguuugagg........................................................................ 1 1 seq
........................aggccaugAaaguuugagg........................................................................ 2 1 seq
........................aggccauggUaguuugagg........................................................................ 2 1 seq
........................aggccauggaaguuugagg........................................................................ 4154 0 seq
........................aggccauggaGguuugagg........................................................................ 9 1 seq
........................aggccauggaaguuCgagg........................................................................ 5 1 seq
........................aggccauggaaguuugagC........................................................................ 35 1 seq
........................Cggccauggaaguuugagg........................................................................ 3 1 seq
........................aggccauggaagGuugagg........................................................................ 1 1 seq
........................aggccauggGaguuugagg........................................................................ 17 1 seq
........................aggcGauggaaguuugagg........................................................................ 1 1 seq
........................aggccauggaaguuugagA........................................................................ 241 1 seq
........................agAccauggaaguuugagga....................................................................... 1 1 seq
........................aggccauggaaguuugagCa....................................................................... 1 1 seq
........................aggccauAgaaguuugagga....................................................................... 1 1 seq
........................aggccauggGaguuugagga....................................................................... 1 1 seq
........................aggccauggaaguuugagga....................................................................... 173 0 seq
........................aggccauggaaguuuAagga....................................................................... 1 1 seq
........................Uggccauggaaguuugaggaa...................................................................... 1 1 seq
........................aggccauggaaguuugaggaG...................................................................... 2 1 seq
........................aggccauggaaguuugaggaU...................................................................... 1 1 seq
........................aggccauggaaguuugaggaa...................................................................... 12 0 seq
........................aggccauggaaguuugaggGa...................................................................... 14 1 seq
........................aggccauggaaguuugaggaCa..................................................................... 1 1 seq
........................aUgccauggaaguuugaggaaau.................................................................... 1 1 seq
........................aggccauggaaguuugaggNaau.................................................................... 1 1 seq
.........................ggGcauggaaguuugag......................................................................... 2 1 seq
.........................ggccauggaagGuugag......................................................................... 2 1 seq
.........................ggccaAggaaguuugag......................................................................... 1 1 seq
.........................ggccauggaUguuugag......................................................................... 1 1 seq
.........................ggccauggaGguuugag......................................................................... 3 1 seq
.........................ggccauggaaguuugaC......................................................................... 1 1 seq
.........................ggccauggGaguuugag......................................................................... 1 1 seq
.........................ggccauggaaguuugag......................................................................... 418 0 seq
.........................ggccauggaaguuuUag......................................................................... 1 1 seq
.........................ggccauggUaguuugag......................................................................... 1 1 seq
.........................ggccauggaaguuugagg........................................................................ 1223 0 seq
.........................ggccauggaGguuugagg........................................................................ 5 1 seq
.........................ggccauggaaguuGgagg........................................................................ 1 1 seq
.........................gUccauggaaguuugagg........................................................................ 1 1 seq
.........................ggccaugAaaguuugagg........................................................................ 1 1 seq
.........................ggccauggaaguuCgagg........................................................................ 2 1 seq
.........................ggccauggaaguGugagg........................................................................ 1 1 seq
.........................Cgccauggaaguuugagg........................................................................ 1 1 seq
.........................ggccaAggaaguuugagg........................................................................ 1 1 seq
.........................Ngccauggaaguuugagg........................................................................ 1 1 seq
.........................ggccauggGaguuugagg........................................................................ 5 1 seq
.........................ggccauggaaguuugagC........................................................................ 3 1 seq
.........................ggccaCggaaguuugagg........................................................................ 4 1 seq
.........................ggccauggaaguuugaUg........................................................................ 1 1 seq
.........................ggccauggaagGuugagg........................................................................ 1 1 seq
.........................ggccauggaaguCugagg........................................................................ 3 1 seq
.........................ggccauggaaguuugagga....................................................................... 21 0 seq
.........................ggccauggaaguuugaggGa...................................................................... 1 1 seq
.........................ggccauggaaguuugaggaC...................................................................... 1 1 seq
..........................gccaAggaaguuugagg........................................................................ 1 1 seq
..........................gccauggaGguuugagg........................................................................ 1 1 seq
..........................gccauggaaguuCgagg........................................................................ 2 1 seq
```

Star **Mature**

```
uucuuagggggucuauggucguuuaggccauggaaguuugaggaaauaauagguaugugaugcucuuacauguucuaggccacaugcaugcuacauugauguaguaaaaaaaggc
..........................gccauggaCguuugagg........................................................................ 1 1 seq
..........................gccaCggaaguuugagg........................................................................ 2 1 seq
..........................gccauggaaguuugCgg........................................................................ 1 1 seq
..........................gccauggaUguuugagg........................................................................ 2 1 seq
..........................gccauggaaguuugagg........................................................................ 479 0 seq
..........................gccauggaagGuugagg........................................................................ 2 1 seq
..........................gccauggaaguuugagC........................................................................ 1 1 seq
..........................gGcauggaaguuugagg........................................................................ 1 1 seq
..........................gccauggaaguuugagCa....................................................................... 1 1 seq
..........................gccauggaaguuugagga....................................................................... 7 0 seq
...........................ccauggaaguuugagga....................................................................... 40 0 seq
...........................ccauggaaguuugaggaa...................................................................... 2 0 seq
...........................ccauggaaguuugaggaCa..................................................................... 1 1 seq
...............................................aauagguCugugaugcu................................................... 1 1 seq
.................................................uagguCugugaugcucuu................................................ 1 1 seq
..................................................agguCugugaugcucuu................................................ 1 1 seq
..................................................agguCugugaugcucuua............................................... 1 1 seq
```

Provisional ID : MA_130420_14025
Score total : 1.9
Score for star read(s) : -1.3
Score for read counts : 0
Score for mfe : 1.6
Score for randfold : 1.6
Score for cons. seed :
Total read count : 1300
Mature read count : 1299
Loop read count : 1
Star read count : 0

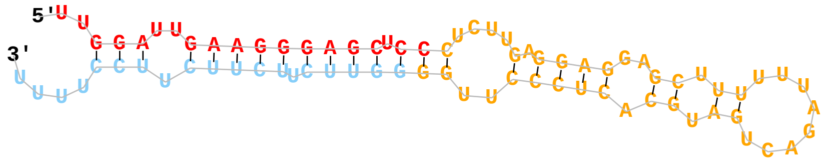

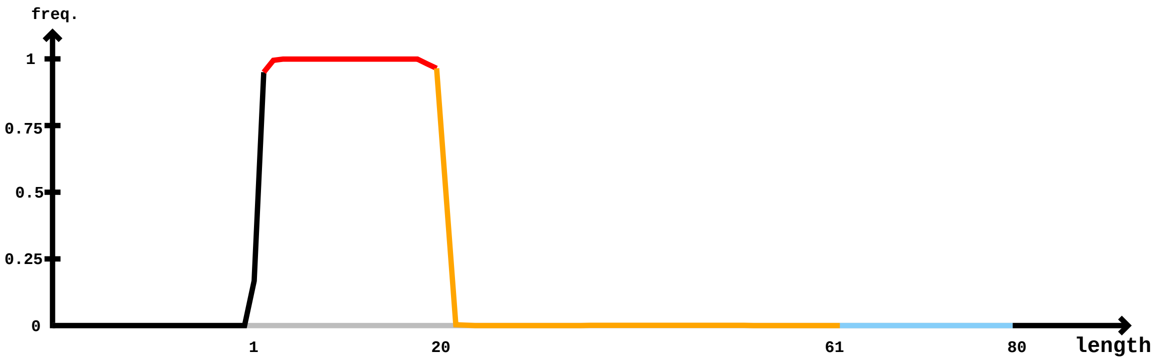

Mature Star

```
5'- ggcaggagcuuccugggauuuuggauugaagggagcucccucuugaggaggagcuuuuuuagacugaugcacucccuugggguucuucuucuucccuuuuaggcugcuucc -3'    exp
    ....(((((..(((((((....(((..(((((((((.(((....(.((((..((.((........)).)).)))))..))))))).))))).))))))))))..))))).    reads   mm   sample
    ...................uuuggUuugaagggagcucc........................................................................    1       1    seq
    ...................uuuggauugaagggagcucc........................................................................    1       0    seq
    ...................Nuuggauugaagggagcucc........................................................................    3       1    seq
    ...................Auuggauugaagggagcuccc.......................................................................    1       1    seq
    ...................Cuuggauugaagggagcuccc.......................................................................    208     1    seq
    ...................uuuggauugaagggagcuccA.......................................................................    4       1    seq
    ....................uuggauugaagggagcC..........................................................................    4       1    seq
    ....................uuggauugaagggagcu..........................................................................    17      0    seq
    ....................uuggauugaagggagcG..........................................................................    2       1    seq
    ....................Cuggauugaagggagcuc.........................................................................    1       1    seq
    ....................uuggauugaagggagcuc.........................................................................    18      0    seq
    ....................uuggauugaagggagcuU.........................................................................    3       1    seq
    ....................uuggauCgaagggagcucc........................................................................    1       1    seq
    ....................uuggGuugaagggagcucc........................................................................    1       1    seq
    ....................uuggauugaagggagcuUc........................................................................    1       1    seq
    ....................uuggauugaagggagcucG........................................................................    2       1    seq
    ....................uuggauugaagggGgcucc........................................................................    1       1    seq
    ....................uuggauugaagAgagcucc........................................................................    1       1    seq
    ....................Auggauugaagggagcucc........................................................................    1       1    seq
    ....................uuggauugaagggaUcucc........................................................................    1       1    seq
    ....................uuggauugaagggagcuAc........................................................................    1       1    seq
    ....................Guggauugaagggagcucc........................................................................    1       1    seq
    ....................uuggauugaaggAagcucc........................................................................    1       1    seq
    ....................Cuggauugaagggagcucc........................................................................    7       1    seq
    ....................uAggauugaagggagcucc........................................................................    2       1    seq
    ....................uuggauugaagggagcuGc........................................................................    1       1    seq
    ....................uuggauuAaagggagcucc........................................................................    1       1    seq
    ....................uugAauugaagggagcucc........................................................................    2       1    seq
    ....................uuggauugCagggagcucc........................................................................    1       1    seq
    ....................uuggauugaagggagcucc........................................................................    851     0    seq
    ....................uuggauugaaUggagcucc........................................................................    1       1    seq
    ....................uuggauugaagggagcuccc.......................................................................    26      0    seq
    ....................uuggauugaagggagcuccG.......................................................................    64      1    seq
    ....................uuggauugaagggagcucUcu......................................................................    1       1    seq
```

```
                    Mature                                                        Star
ggcaggagcuuccugggauuuuggauugaagggagcucccucuugaggaggagcuuuuuuagacugaugcacucccuuggggguucuucuucuuccuuuuaggcugcuucc
....................uuggauugaagggagcuccAu.....................................................................     1     1     seq
....................uuggauugaagggagcuccAuc....................................................................     1     1     seq
....................uuggauugaagggagcuccUuc....................................................................     1     1     seq
.....................uggauugaagggagcucc.......................................................................     53    0     seq
.....................uUgauugaagggagcucc.......................................................................     1     1     seq
.....................uggauugaagggGgcucc.......................................................................     1     1     seq
.....................uggauugaagggagcuccG......................................................................     1     1     seq
.....................uggauugaagggagcuccc......................................................................     2     0     seq
......................ggauugaagggagcucc.......................................................................     6     0     seq
......................................................uuuuuuGgacugaugca.......................................     1     1     seq
```

Provisional ID	:	MA_1304376_43039
Score total	:	162020.3
Score for star read(s)	:	3.9
Score for read counts	:	162021.5
Score for mfe	:	-2.8
Score for randfold	:	-2.2
Score for cons. seed	:	
Total read count	:	317810
Mature read count	:	314677
Loop read count	:	3025
Star read count	:	108

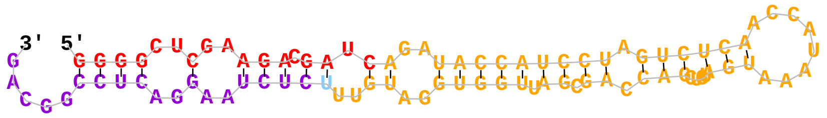

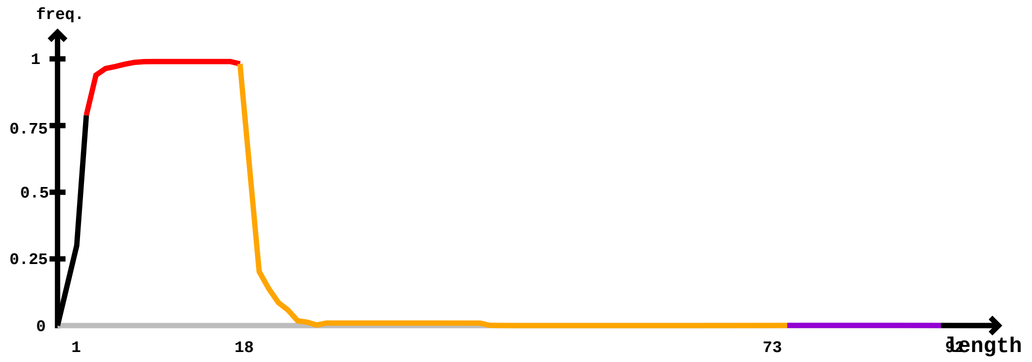


```
         Mature                                                                     Star
5'- ggggggcucgaagacgaucagauaccauccuagucucaaccauaaaaugaugucgaccagcgauugguggauguuucucuaaggacuccggcagcacc   -3'   obs
    ggggggcucgaagacgaucagauaccauccuagucucaaccauaaaaugaugucgaccagcgauugguggauguuucucuaaggacuccggcagcacc         exp
    .((((..(..(((.((.((..((((((((.((((((........)))....))).)).)).)))))..))..)))))..)..)))).........    reads    mm     sample
    Uggggcucgaagacgau.................................................................................    16       1      seq
    gggggcGcgaagacgau.................................................................................    1        1      seq
    gggggcuGgaagacgau.................................................................................    1        1      seq
    ggUggcucgaagacgau.................................................................................    4        1      seq
    gggggcucgaagacgaC.................................................................................    337      1      seq
    gggggcucgaUgacgau.................................................................................    1        1      seq
    gggggUucgaagacgau.................................................................................    3        1      seq
    gggggcucgaagacUau.................................................................................    1        1      seq
    gAgggcucgaagacgau.................................................................................    2        1      seq
    gggggcucgaagUcgau.................................................................................    1        1      seq
    gggggcucgaagacgGu.................................................................................    25       1      seq
    gggggcucgaGgacgau.................................................................................    10       1      seq
    Nggggcucgaagacgau.................................................................................    1        1      seq
    gggUgcucgaagacgau.................................................................................    2        1      seq
    gggggcAcgaagacgau.................................................................................    1        1      seq
    gggggcucgaagacgaA.................................................................................    14       1      seq
    gUgggcucgaagacgau.................................................................................    1        1      seq
    gggggcucgaagacgCu.................................................................................    1        1      seq
    gggggcucgaagacgaG.................................................................................    33       1      seq
    ggAggcucgaagacgau.................................................................................    5        1      seq
    gggggcCcgaagacgau.................................................................................    5        1      seq
    gggggcucgaagacgau.................................................................................    2308     0      seq
    gggggcucgaagacgUu.................................................................................    2        1      seq
    gggggcucgGagacgau.................................................................................    10       1      seq
    gggggGucgaagacgauc................................................................................    4        1      seq
    gggggcGcgaagacgauc................................................................................    5        1      seq
    gggCgcucgaagacgauc................................................................................    1        1      seq
    gggAgcucgaagacgauc................................................................................    17       1      seq
    gggggcucCaagacgauc................................................................................    8        1      seq
    gggggcucgaagacgGuc................................................................................    168      1      seq
    gggggcucgaaAacgauc................................................................................    20       1      seq
    gggggcucgaagacgaCc................................................................................    422      1      seq
    gUgggcucgaagacgauc................................................................................    39       1      seq
```

Mature **Star**

gggggcucgaagacgaucagauaccauccuagucucaaccauaaaaugaugucgaccagcgauugguggauguuucucuaaggacuccggcagcacc

gAgggcucgaagacgauc...	29	1	seq
gggggcCcgaagacgauc...	135	1	seq
gggggcucgaaCacgauc...	5	1	seq
gggggAucgaagacgauc...	8	1	seq
gggggcucgaagUcgauc...	12	1	seq
gggggcucgaagacgCuc...	9	1	seq
ggCggcucgaagacgauc...	2	1	seq
ggUggcucgaagacgauc...	24	1	seq
Nggggcucgaagacgauc...	58	1	seq
gggggcuGgaagacgauc...	1	1	seq
ggggCcucgaagacgauc...	4	1	seq
gggggcucgaGgacgauc...	208	1	seq
gggggcucgaagaGgauc...	6	1	seq
gggggcucgaCgacgauc...	9	1	seq
gggggcucgaagaAgauc...	7	1	seq
gggggcuAgaagacgauc...	10	1	seq
gCgggcucgaagacgauc...	1	1	seq
ggAggcucgaagacgauc...	28	1	seq
gggggcucgaagCcgauc...	8	1	seq
gggUgcucgaagacgauc...	23	1	seq
ggggUcucgaagacgauc...	8	1	seq
gggggcucgaagacgUuc...	31	1	seq
ggggAcucgaagacgauc...	41	1	seq
gggggcucgaagacgaAc...	18	1	seq
gggggcAcgaagacgauc...	18	1	seq
gggggcucgaagacgaGc...	76	1	seq
gggggcucgaagacUauc...	13	1	seq
gggggcucUaagacgauc...	27	1	seq
gggggcucgaagacgauc...	47726	0	seq
Uggggcucgaagacgauc...	169	1	seq
gggggcucgCagacgauc...	8	1	seq
gggggcucgaagacgauA...	352	1	seq
gggggcucNaagacgauc...	1	1	seq
gggggcucgaaUacgauc...	8	1	seq
Cggggcucgaagacgauc...	12	1	seq
gggggcucgaagacCauc...	8	1	seq
gggggcucgaUgacgauc...	25	1	seq
gggggcucgUagacgauc...	10	1	seq
gggggUucgaagacgauc...	31	1	seq
gggggcucgGagacgauc...	140	1	seq
gggggcucgaagacgauU...	1371	1	seq
gggggcucgUagacgauca..	6	1	seq
gggggcucgaaAacgauca..	5	1	seq
gggggGucgaagacgauca..	3	1	seq
Nggggcucgaagacgauca..	25	1	seq
ggCggcucgaagacgauca..	6	1	seq
gggggcuGgaagacgauca..	1	1	seq
ggggAcucgaagacgauca..	19	1	seq
gggggcucgaagacgaAca..	10	1	seq
gggggcucgaagacCauca..	6	1	seq
gggggcucgaagacgUuca..	21	1	seq
Cggggcucgaagacgauca..	16	1	seq
gggggcucgaaCacgauca..	2	1	seq
gggggUucgaagacgauca..	23	1	seq
gggggcucgaagacgaucU..	144	1	seq
gggggcCcgaagacgauca..	73	1	seq
gggggcucgaagacgauca..	28744	0	seq
gggUgcucgaagacgauca..	13	1	seq
gggggcucgaaUacgauca..	1	1	seq
gggggcucgaagacgaucC..	27	1	seq
gggggcucgaagacgGuca..	95	1	seq
gggggcucgaagacUauca..	8	1	seq
ggAggcucgaagacgauca..	25	1	seq
ggggUcucgaagacgauca..	4	1	seq
gggggcucgaagacgaCca..	183	1	seq
gggggcucgaGgacgauca..	106	1	seq
ggUggcucgaagacgauca..	24	1	seq
gggggcucgaUgacgauca..	11	1	seq
gggggcucCaagacgauca..	2	1	seq
gggggcucgaagacgaucG..	784	1	seq

Mature **Star**

```
ggggggcucgaagacgaucagauaccauccuagucucaaccauaaaaugaugucgaccagcgauugguggauguuucucuaaggacuccggcagcacc
gggggcucgaagaGgauca.............................................................................    9      1    seq
gggggcucgaagCcgauca.............................................................................    3      1    seq
gUgggcucgaagacgauca.............................................................................    12     1    seq
gggggcAcgaagacgauca.............................................................................    14     1    seq
gggggcucgaagacgauUa.............................................................................    30     1    seq
Uggggcucgaagacgauca.............................................................................    178    1    seq
gggggcucgaagacgauGa.............................................................................    4      1    seq
gggggAucgaagacgauca.............................................................................    10     1    seq
gggggcucgGagacgauca.............................................................................    101    1    seq
gggggcucUaagacgauca.............................................................................    7      1    seq
gggggcucgaagacgCuca.............................................................................    8      1    seq
gggAgcucgaagacgauca.............................................................................    18     1    seq
gggggcuAgaagacgauca.............................................................................    1      1    seq
gggggcucgaagacgauAa.............................................................................    6      1    seq
gggggcucgCagacgauca.............................................................................    1      1    seq
gggggcucgaCgacgauca.............................................................................    7      1    seq
ggggCcucgaagacgauca.............................................................................    2      1    seq
gAgggcucgaagacgauca.............................................................................    18     1    seq
gggggcucgaagacgaGca.............................................................................    36     1    seq
gggggcucgaagUcgauca.............................................................................    6      1    seq
gggggcucgaagaAgauca.............................................................................    3      1    seq
gggggcucgaagacCaucag............................................................................    1      1    seq
gggggcucgaagacgaucUg............................................................................    1      1    seq
ggAggcucgaagacgaucag............................................................................    2      1    seq
gggggAucgaagacgaucag............................................................................    1      1    seq
gggggcucgaagacgaCcag............................................................................    9      1    seq
gggggcucgaagacgaucCg............................................................................    2      1    seq
gggggcucgaagacgaucaC............................................................................    7      1    seq
Cggggcucgaagacgaucag............................................................................    2      1    seq
gggggcucUaagacgaucag............................................................................    1      1    seq
gggggcucgaagacgauAag............................................................................    1      1    seq
gggggcucgaagacgGucag............................................................................    14     1    seq
gggggcucgaagacgaAcag............................................................................    1      1    seq
Nggggcucgaagacgaucag............................................................................    6      1    seq
gggggcucgaagacgaucGg............................................................................    21     1    seq
Aggggcucgaagacgaucag............................................................................    10     1    seq
gggggcAcgaagacgaucag............................................................................    2      1    seq
gggggUucgaagacgaucag............................................................................    1      1    seq
gggggcucgaagacgauUag............................................................................    4      1    seq
gggAgcucgaagacgaucag............................................................................    3      1    seq
Uggggcucgaagacgaucag............................................................................    15     1    seq
gggggcucgGagacgaucag............................................................................    13     1    seq
gggggcucgaCgacgaucag............................................................................    1      1    seq
gggggcucgaaAacgaucag............................................................................    1      1    seq
gggggGucgaagacgaucag............................................................................    1      1    seq
gggggcucgaaUacgaucag............................................................................    2      1    seq
ggUggcucgaagacgaucag............................................................................    4      1    seq
gCgggcucgaagacgaucag............................................................................    1      1    seq
gggggcucgaagacUaucag............................................................................    1      1    seq
gggggcCcgaagacgaucag............................................................................    10     1    seq
gggggcucgaagacgaucag............................................................................    4480   0    seq
ggggUcucgaagacgaucag............................................................................    1      1    seq
ggggAcucgaagacgaucag............................................................................    3      1    seq
gggggcucgaGgacgaucag............................................................................    15     1    seq
ggggCcucgaagacgaucag............................................................................    1      1    seq
gUgggcucgaagacgaucag............................................................................    3      1    seq
gggggcucgaagGcgaucag............................................................................    13     1    seq
gAgggcucgaagacgaucag............................................................................    4      1    seq
gggggcucgaagaGgaucag............................................................................    1      1    seq
gggggcucgaagacgUucag............................................................................    4      1    seq
gggggcucgaagacgaGcag............................................................................    4      1    seq
gggggcucgaagacgaucaCa...........................................................................    2      1    seq
gggggcucgaagacgaucagG...........................................................................    61     1    seq
Aggggcucgaagacgaucaga...........................................................................    8      1    seq
gggggcucgaagacgaucCga...........................................................................    2      1    seq
gggggcucgaagacgaCcaga...........................................................................    7      1    seq
gggggcucgaagacgaucaga...........................................................................    3095   0    seq
gggggcucgaagacgGucaga...........................................................................    11     1    seq
gggggcucgaagacgaucGga...........................................................................    18     1    seq
gggggcucCaagacgaucaga...........................................................................    1      1    seq
```

```
 Mature                                                                        Star
gggggcucgaagacgaucagauaccauccuagucucaaccauaaaaugaugucgaccagcgauugguggauguuucucuaaggacuccggcagcacc
gAgggcucgaagacgaucaga............................................................................   1      1      seq
gggggcucgaagacUaucaga............................................................................   2      1      seq
gUgggcucgaagacgaucaga............................................................................   2      1      seq
gggggcucgaagacgCucaga............................................................................   1      1      seq
gggggcucgaagacgauUaga............................................................................   4      1      seq
gggggcucgaGgacgaucaga............................................................................   9      1      seq
gggAgcucgaagacgaucaga............................................................................   1      1      seq
ggUggcucgaagacgaucaga............................................................................   1      1      seq
ggAggcucgaagacgaucaga............................................................................   5      1      seq
gggggUucgaagacgaucaga............................................................................   2      1      seq
gggggcucgaagacgaGcaga............................................................................   1      1      seq
gggggcucgaagGcgaucaga............................................................................   11     1      seq
Uggggcucgaagacgaucaga............................................................................   14     1      seq
gggggcucgaaCacgaucaga............................................................................   2      1      seq
Nggggcucgaagacgaucaga............................................................................   2      1      seq
gggUgcucgaagacgaucaga............................................................................   4      1      seq
ggggAcucgaagacgaucaga............................................................................   2      1      seq
gggggcucgaUgacgaucaga............................................................................   2      1      seq
gggggcCcgaagacgaucaga............................................................................   5      1      seq
gggggcucgaagacgaucUga............................................................................   2      1      seq
gggggcucgaagacgaucagU............................................................................   15     1      seq
gggggcucgaagacgaucagC............................................................................   3      1      seq
gggggcucgaagacgauAaga............................................................................   1      1      seq
gggggcucgGagacgaucaga............................................................................   13     1      seq
ggggAcucgaagacgaucagau...........................................................................   1      1      seq
gggggcucgaagacgaucagaG...........................................................................   9      1      seq
ggCggcucgaagacgaucagau...........................................................................   1      1      seq
Uggggcucgaagacgaucagau...........................................................................   6      1      seq
ggggUcucgaagacgaucagau...........................................................................   2      1      seq
gggUgcucgaagacgaucagau...........................................................................   2      1      seq
gggggcucgGagacgaucagau...........................................................................   2      1      seq
gggggcucgaagacgaucUgau...........................................................................   1      1      seq
gggggcucgaagacgaucGgau...........................................................................   6      1      seq
gggAgcucgaagacgaucagau...........................................................................   2      1      seq
gggggcucgaagacgaucagau...........................................................................   1149   0      seq
ggAggcucgaagacgaucagau...........................................................................   2      1      seq
gggggcucgCagacgaucagau...........................................................................   1      1      seq
gggggcucgaagUcgaucagau...........................................................................   1      1      seq
gggggcucgaagacgaucagaC...........................................................................   133    1      seq
gggggcucgaGgacgaucagau...........................................................................   3      1      seq
gggggcucgaagGcgaucagau...........................................................................   3      1      seq
gUgggcucgaagacgaucagau...........................................................................   2      1      seq
Aggggcucgaagacgaucagau...........................................................................   3      1      seq
gggggUucgaagacgaucagau...........................................................................   1      1      seq
gggCgcucgaagacgaucagau...........................................................................   1      1      seq
gggggcCcgaagacgaucagau...........................................................................   1      1      seq
gggggcucgaagacgaCcagau...........................................................................   1      1      seq
gggggcucgaagacgGucagau...........................................................................   2      1      seq
gggggcucgaagacgaAcagau...........................................................................   1      1      seq
Nggggcucgaagacgaucagau...........................................................................   2      1      seq
gggggcucgaagacgaucagaA...........................................................................   3      1      seq
gggggcAcgaagacgaucagau...........................................................................   1      1      seq
gggggcuAgaagacgaucagau...........................................................................   1      1      seq
gggggcucgaagacgaucagGu...........................................................................   6      1      seq
gggggcucgaagGcgaucagaua..........................................................................   1      1      seq
gCgggcucgaagacgaucagaua..........................................................................   2      1      seq
gggggcucgaagacgaucagauG..........................................................................   9      1      seq
Aggggcucgaagacgaucagaua..........................................................................   1      1      seq
gggggcucgaagacgCucagaua..........................................................................   1      1      seq
gggggcucgaagCcgaucagaua..........................................................................   1      1      seq
gggggcucgaagacgaucagauU..........................................................................   4      1      seq
gggggcCcgaagacgaucagaua..........................................................................   2      1      seq
gggggcucgaagacUaucagaua..........................................................................   1      1      seq
ggAggcucgaagacgaucagaua..........................................................................   1      1      seq
gggggcucgaagacgaucagaCa..........................................................................   16     1      seq
ggggCcucgaagacgaucagaua..........................................................................   1      1      seq
gggAgcucgaagacgaucagaua..........................................................................   1      1      seq
gggggcucgaagacgaucagCua..........................................................................   1      1      seq
gggggcucgGagacgaucagaua..........................................................................   9      1      seq
gggggcucgaagUcgaucagaua..........................................................................   1      1      seq
```

Mature **Star**

gggggcucgaagacgaucagauaccauccuagucucaaccauaaaaugaugucgaccagcgauugguggauguuucucuaaggacuccggcagcacc			
gggggcucgaagacgaAcagaua..	1	1	seq
gggggcucgCagacgaucagaua..	1	1	seq
gggggcucgaGgacgaucagaua..	4	1	seq
Cggggcucgaagacgaucagaua..	1	1	seq
gggggcucgaagacgauUagaua..	2	1	seq
gggggcucgaagacgaucGgaua..	8	1	seq
gggggcucgaagacgaCcagaua..	2	1	seq
gggggcucgaagacgaucagGua..	5	1	seq
ggUggcucgaagacgaucagaua..	1	1	seq
ggggAcucgaagacgaucagaua..	3	1	seq
gggggcucgaagacgGucagaua..	5	1	seq
gUgggcucgaagacgaucagaua..	2	1	seq
Uggggcucgaagacgaucagaua..	8	1	seq
Nggggcucgaagacgaucagaua..	1	1	seq
gggggcucgaagacgaucagauC..	1	1	seq
gggggcucgaagacgaucagaua..	1428	0	seq
Uggggcucgaagacgaucagauac...	2	1	seq
gggggcucgaagacgaucGgauac...	1	1	seq
gggggcucgaagacgaucagauCc...	1	1	seq
gggggcucgaagacgaucagGuac...	1	1	seq
gggggcucgaagacgaucagauaU...	11	1	seq
gggggcucgaagacgaucagauac...	165	0	seq
gggggcucgaCgacgaucagauac...	1	1	seq
gggggcucgGagacgaucagauac...	2	1	seq
gggggcuUgaagacgaucagauacc..	1	1	seq
gggggcucgaagacgaucagauacA..	33	1	seq
gggggcucgaCgacgaucagauacc..	1	1	seq
gggggcucgaagacgGucagauacc..	3	1	seq
gggggcucgaagacgaucGgauacc..	4	1	seq
gggggcucgaagacgaucagauacG..	1	1	seq
gAgggcucgaagacgaucagauacc..	1	1	seq
gggggcucgaGgacgaucagauacc..	5	1	seq
gggggUucgaagacgaucagauacc..	2	1	seq
gggggcucgaagacgaucagauUcc..	1	1	seq
gggggcucgaagacgaCcagauacc..	1	1	seq
gggggcucgaagacgaucagauGcc..	3	1	seq
gggggcucgaagGcgaucagauacc..	2	1	seq
gggggcucgaagacgaucagaCacc..	1	1	seq
gggggcucgaagacgaucUgauacc..	2	1	seq
Uggggcucgaagacgaucagauacc..	4	1	seq
gggggcucUaagacgaucagauacc..	1	1	seq
gggggcCcgaagacgaucagauacc..	3	1	seq
gggggcucgGagacgaucagauacc..	1	1	seq
gggggcucgaagacgaucagauacc..	829	0	seq
gggggcucgaagacgaucagGuacc..	1	1	seq
gggggcucgaagacgaucagauaccU...	13	1	seq
gggggcucgaagacgaucagGuacca...	1	1	seq
gggggcucgaagacgaucagaCacca...	1	1	seq
gggggcucgaagacgaucagauacca...	82	0	seq
gggggcucgaagacgauUagauacca...	1	1	seq
Uggggcucgaagacgaucagauacca...	1	1	seq
gggggcucgaagacgaucagauaccC...	9	1	seq
gggggcucgaagacgaucagauGcca...	1	1	seq
gggggcucgaagacgaucagauaccauU...	1	1	seq
gggggcucgaagacgaucagauaccCucc..	6	1	seq
gggggcucgaagacgaucagauaccaucc..	1	0	seq
gggggcucgaagacgaucagauaccauccu...	3	0	seq
gggggcucgaagacgaucagauaccauccA...	1	1	seq
gggggcucgaagacgaucagauaccUuccu...	1	1	seq
.ggggcucgaagacgCuc...	20	1	seq
.ggggcucgaagacgGuc...	282	1	seq
.ggAgcucgaagacgauc...	49	1	seq
.ggggcucgaagacgaAc...	42	1	seq
.Agggcucgaagacgauc...	128	1	seq
.ggggcucgaagacgauc...	78575	0	seq
.ggggcuGgaagacgauc...	3	1	seq
.gAggcucgaagacgauc...	50	1	seq
.ggggcucgaaCacgauc...	13	1	seq
.gggUcucgaagacgauc...	18	1	seq
.ggggcucgaUgacgauc...	28	1	seq

Mature **Star**

```
gggggcucgaagacgaucagauaccauccuagucucaaccauaaaaugaugucgaccagcgauugguggauguuucucuaaggacuccggcagcacc
.ggggcucgaagacgauA...............................................................................   545    1    seq
.ggggcucgaagCcgauc...............................................................................   22     1    seq
.ggggcucgNagacgauc...............................................................................   1      1    seq
.gggAcucgaagacgauc...............................................................................   73     1    seq
.ggCgcucgaagacgauc...............................................................................   6      1    seq
.ggggcucgaaAacgauc...............................................................................   27     1    seq
.ggggcGcgaagacgauc...............................................................................   13     1    seq
.ggggcucgaagaGgauc...............................................................................   21     1    seq
.ggggcucgaaUacgauc...............................................................................   17     1    seq
.gUggcucgaagacgauc...............................................................................   32     1    seq
.ggggcucgaagacgaCc...............................................................................   641    1    seq
.ggggcucgCagacgauc...............................................................................   12     1    seq
.ggggcucCaagacgauc...............................................................................   6      1    seq
.ggggcucgaagacUauc...............................................................................   24     1    seq
.ggggcucgaagaAgauc...............................................................................   15     1    seq
.Ngggcucgaagacgauc...............................................................................   93     1    seq
.ggggcuAgaagacgauc...............................................................................   6      1    seq
.ggggcCcgaagacgauc...............................................................................   216    1    seq
.ggggcucgaagacgauU...............................................................................   2303   1    seq
.ggggAucgaagacgauc...............................................................................   1      1    seq
.ggggcucgGagacgauc...............................................................................   301    1    seq
.ggggcucgaGgacgauc...............................................................................   284    1    seq
.ggggcucgaagacgUuc...............................................................................   44     1    seq
.Cgggcucgaagacgauc...............................................................................   26     1    seq
.ggggcAcgaagacgauc...............................................................................   41     1    seq
.gCggcucgaagacgauc...............................................................................   2      1    seq
.ggggGucgaagacgauc...............................................................................   13     1    seq
.ggggcucgaagacCauc...............................................................................   7      1    seq
.ggggUucgaagacgauc...............................................................................   57     1    seq
.ggggcucgUagacgauc...............................................................................   25     1    seq
.ggggcucUaagacgauc...............................................................................   33     1    seq
.gggCcucgaagacgauc...............................................................................   1      1    seq
.ggggcucgaagacgaGc...............................................................................   123    1    seq
.ggggcucgaagUcgauc...............................................................................   35     1    seq
.ggUgcucgaagacgauc...............................................................................   32     1    seq
.ggggcucgaaAacgauca..............................................................................   13     1    seq
.ggggcucgaagacgaucG..............................................................................   1299   1    seq
.ggggcucgaagacgaAca..............................................................................   23     1    seq
.ggggcucgaagaGgauca..............................................................................   11     1    seq
.ggggcucgaagacgauGa..............................................................................   5      1    seq
.Agggcucgaagacgauca..............................................................................   87     1    seq
.ggggcucgaagacgaucC..............................................................................   67     1    seq
.ggggcucgaGgacgauca..............................................................................   175    1    seq
.gggCcucgaagacgauca..............................................................................   1      1    seq
.ggggcucgaagacgUuca..............................................................................   21     1    seq
.ggUgcucgaagacgauca..............................................................................   14     1    seq
.ggggcucgaaCacgauca..............................................................................   4      1    seq
.ggggcucgaagacCauca..............................................................................   7      1    seq
.ggggGucgaagacgauca..............................................................................   12     1    seq
.ggggcucgaagUcgauca..............................................................................   12     1    seq
.ggggcucgaagaAgauca..............................................................................   11     1    seq
.ggggcucgaagacgCuca..............................................................................   17     1    seq
.gggAcucgaagacgauca..............................................................................   49     1    seq
.ggggcucUaagacgauca..............................................................................   10     1    seq
.gAggcucgaagacgauca..............................................................................   40     1    seq
.gggUcucgaagacgauca..............................................................................   10     1    seq
.ggggcucgaaUacgauca..............................................................................   13     1    seq
.ggggcucgaagacgaGca..............................................................................   59     1    seq
.ggggcGcgaagacgauca..............................................................................   11     1    seq
.ggggcucgaagCcgauca..............................................................................   8      1    seq
.Cgggcucgaagacgauca..............................................................................   19     1    seq
.ggAgcucgaagacgauca..............................................................................   30     1    seq
.ggggcCcgaagacgauca..............................................................................   146    1    seq
.ggggcucgaagacgaucU..............................................................................   231    1    seq
.ggggcuAgaagacgauca..............................................................................   3      1    seq
.ggggcAcgaagacgauca..............................................................................   21     1    seq
.ggCgcucgaagacgauca..............................................................................   3      1    seq
.ggggcucgaagacgaCca..............................................................................   281    1    seq
.gUggcucgaagacgauca..............................................................................   33     1    seq
.ggggcucgaagacgauAa..............................................................................   9      1    seq
```

Mature **Star**

ggggcucgaagacgaucagauaccauccuagucucaaccauaaaaugaugucgaccagcgauugguggauguuucucuaaggacuccggcagcacc			
.ggggAucgaagacgauca...	2	1	seq
.ggggcucgaagacUauca...	12	1	seq
.Ngggcucgaagacgauca...	51	1	seq
.ggggcuGgaagacgauca...	2	1	seq
.ggggcucgaUgacgauca...	15	1	seq
.ggggcucgaagacgauUa...	54	1	seq
.ggggcucgUagacgauca...	19	1	seq
.ggggcucCaagacgauca...	7	1	seq
.ggggUucgaagacgauca...	36	1	seq
.ggggcucgaagacgGuca...	165	1	seq
.gCggcucgaagacgauca...	7	1	seq
.ggggcucgaagacgauca...	49675	0	seq
.ggggcucgCagacgauca...	9	1	seq
.ggggcucgGagacgauca...	205	1	seq
.ggggcucgaagacgauGag..	1	1	seq
.ggUgcucgaagacgaucag..	1	1	seq
.ggggcucgUagacgaucag..	4	1	seq
.ggggcucgaagacgaucCg..	4	1	seq
.Ngggcucgaagacgaucag..	10	1	seq
.ggggcucgaagacgaucaC..	26	1	seq
.ggggcuAgaagacgaucag..	1	1	seq
.ggggUucgaagacgaucag..	6	1	seq
.ggAgcucgaagacgaucag..	8	1	seq
.ggggcucgaGgacgaucag..	35	1	seq
.ggggcucgaagacgauUag..	9	1	seq
.gggAcucgaagacgaucag..	7	1	seq
.ggggcucgaagacCaucag..	1	1	seq
.Agggcucgaagacgaucag..	15	1	seq
.ggggcucgGagacgaucag..	31	1	seq
.gggUcucgaagacgaucag..	3	1	seq
.gCggcucgaagacgaucag..	2	1	seq
.ggggcucgaagGcgaucag..	32	1	seq
.ggggcucgaaUacgaucag..	1	1	seq
.ggggcucgaagacgauAag..	1	1	seq
.ggggcucgaagacgaGcag..	3	1	seq
.ggggcuGgaagacgaucag..	1	1	seq
.ggggcucgaagacgaucag..	8794	0	seq
.ggggcucgaUgacgaucag..	4	1	seq
.ggggcucgaagaGgaucag..	3	1	seq
.gUggcucgaagacgaucag..	5	1	seq
.Ugggcucgaagacgaucag..	8	1	seq
.ggggcucgaagacgGucag..	31	1	seq
.ggggcucgaagacgaAcag..	2	1	seq
.Cgggcucgaagacgaucag..	3	1	seq
.ggggcucUaagacgaucag..	2	1	seq
.ggggcucgaagacgaCcag..	16	1	seq
.ggggcucgaagacgaucGg..	45	1	seq
.ggggcCcgaagacgaucag..	24	1	seq
.ggggcucgaagUcgaucag..	2	1	seq
.gAggcucgaagacgaucag..	8	1	seq
.ggggcucgaagaAgaucag..	5	1	seq
.ggggcucgaagacgUucag..	5	1	seq
.ggggcAcgaagacgaucag..	4	1	seq
.ggggcucgaagacUaucag..	1	1	seq
.ggggcucgaagacgaucUg..	8	1	seq
.ggggcucgaaAacgaucag..	4	1	seq
.ggggcucgaagacgGucaga...	17	1	seq
.ggggcucgaagacgaGcaga...	2	1	seq
.ggggcucgaCgacgaucaga...	1	1	seq
.ggggcucgaagacgaucagG...	119	1	seq
.gggCcucgaagacgaucaga...	2	1	seq
.ggggcGcgaagacgaucaga...	1	1	seq
.ggggcucgaagacgaAcaga...	3	1	seq
.ggggcucgaagacgaucagC...	7	1	seq
.ggggcucgCagacgaucaga...	1	1	seq
.ggggcucgaagaAgaucaga...	1	1	seq
.ggggcucgUagacgaucaga...	2	1	seq
.ggAgcucgaagacgaucaga...	2	1	seq
.Ugggcucgaagacgaucaga...	8	1	seq
.ggggcAcgaagacgaucaga...	1	1	seq

Mature **Star**

```
gggggcucgaagacgaucagauaccauccuagucucaaccauaaaaugaugucgaccagcgauugguggauguuucucuaaggacuccggcagcacc
.ggggUucgaagacgaucaga............................................................................    4      1    seq
.ggggcucgaagacgaucaga............................................................................    5516   0    seq
.ggggcucgaagacgUucaga............................................................................    6      1    seq
.ggUgcucgaagacgaucaga............................................................................    5      1    seq
.gggUcucgaagacgaucaga............................................................................    1      1    seq
.ggggcucgaagCcgaucaga............................................................................    1      1    seq
.ggggcucgaagacgaucUga............................................................................    3      1    seq
.gCggcucgaagacgaucaga............................................................................    1      1    seq
.ggggcucgaaAacgaucaga............................................................................    5      1    seq
.gggAcucgaagacgaucaga............................................................................    5      1    seq
.ggggcucgaUgacgaucaga............................................................................    2      1    seq
.ggggcucgaagacgaucGga............................................................................    22     1    seq
.ggggcucgaaUacgaucaga............................................................................    2      1    seq
.ggggcCcgaagacgaucaga............................................................................    20     1    seq
.ggggcucgaagacUaucaga............................................................................    1      1    seq
.ggggcucgaagacgCucaga............................................................................    1      1    seq
.ggggcucgaagUcgaucaga............................................................................    4      1    seq
.ggggcucgaagacgauUaga............................................................................    4      1    seq
.Ngggcucgaagacgaucaga............................................................................    5      1    seq
.ggggcucgaagacgaCcaga............................................................................    25     1    seq
.ggggGucgaagacgaucaga............................................................................    2      1    seq
.ggggcucgGagacgaucaga............................................................................    19     1    seq
.gAggcucgaagacgaucaga............................................................................    4      1    seq
.ggggcucgaagacgaucagU............................................................................    25     1    seq
.ggggcucgaagacgaucCga............................................................................    2      1    seq
.ggggcucgaagGcgaucaga............................................................................    18     1    seq
.Cgggcucgaagacgaucaga............................................................................    1      1    seq
.Agggcucgaagacgaucaga............................................................................    6      1    seq
.ggggcucgaGgacgaucaga............................................................................    24     1    seq
.ggggcucgaagacgaucaCa............................................................................    2      1    seq
.gUggcucgaagacgaucaga............................................................................    1      1    seq
.Ugggcucgaagacgaucagau...........................................................................    6      1    seq
.ggggcCcgaagacgaucagau...........................................................................    3      1    seq
.ggggcucgaagacgaCcagau...........................................................................    3      1    seq
.ggUgcucgaagacgaucagau...........................................................................    1      1    seq
.ggggcucgaagaGgaucagau...........................................................................    1      1    seq
.gggUcucgaagacgaucagau...........................................................................    3      1    seq
.gUggcucgaagacgaucagau...........................................................................    1      1    seq
.ggggcucgaUgacgaucagau...........................................................................    1      1    seq
.ggggcucgaagGcgaucagau...........................................................................    3      1    seq
.Ngggcucgaagacgaucagau...........................................................................    1      1    seq
.ggggcucgaagacgaucagGu...........................................................................    12     1    seq
.ggggcucgGagacgaucagau...........................................................................    6      1    seq
.ggggcucUaagacgaucagau...........................................................................    1      1    seq
.ggggcucgaagacgaucagaA...........................................................................    11     1    seq
.ggggUucgaagacgaucagau...........................................................................    2      1    seq
.ggggcAcgaagacgaucagau...........................................................................    1      1    seq
.ggggcucgaGgacgaucagau...........................................................................    8      1    seq
.ggggcucgaaAacgaucagau...........................................................................    3      1    seq
.Cgggcucgaagacgaucagau...........................................................................    1      1    seq
.gAggcucgaagacgaucagau...........................................................................    1      1    seq
.Agggcucgaagacgaucagau...........................................................................    1      1    seq
.ggggcucgaagacgauUagau...........................................................................    2      1    seq
.ggggcucgaagacgaucGgau...........................................................................    5      1    seq
.ggggcucgaagacgaGcagau...........................................................................    1      1    seq
.ggggcucgaagacgaucagaG...........................................................................    16     1    seq
.ggggcucgaagacgaucagaC...........................................................................    225    1    seq
.ggggcucgaagacgaucagau...........................................................................    1736   0    seq
.ggggcucgaagacgGucagau...........................................................................    1      1    seq
.ggggcucgaagacgaucagCu...........................................................................    1      1    seq
.ggggcucgaagacgCucagau...........................................................................    2      1    seq
.ggggcuGgaagacgaucagaua..........................................................................    1      1    seq
.ggggcucgaagacgaAcagaua..........................................................................    1      1    seq
.Ugggcucgaagacgaucagaua..........................................................................    5      1    seq
.ggggcucgaGgacgaucagaua..........................................................................    7      1    seq
.Agggcucgaagacgaucagaua..........................................................................    6      1    seq
.ggggcucgGagacgaucagaua..........................................................................    11     1    seq
.ggggcucgaaAacgaucagaua..........................................................................    4      1    seq
.Ngggcucgaagacgaucagaua..........................................................................    3      1    seq
.ggggcucgaagCcgaucagaua..........................................................................    1      1    seq
```

```
                       Mature                                                        Star
gggggcucgaagacgaucagauaccauccuagucucaaccauaaaaugaugucgaccagcgauugguggauguuucucuaaggacuccggcagcacc
.ggggcucgaagacgaucagUua..........................................................................    1       1    seq
.ggggcucgaaUacgaucagaua..........................................................................    1       1    seq
.ggggcuAgaagacgaucagaua..........................................................................    1       1    seq
.ggggcucgaagacgaucagauG..........................................................................    21      1    seq
.ggUgcucgaagacgaucagaua..........................................................................    2       1    seq
.ggggcucgaagUcgaucagaua..........................................................................    1       1    seq
.ggggcucgaagacgaucCgaua..........................................................................    1       1    seq
.ggggcucgaagacgaucagaua..........................................................................    2404    0    seq
.gggAcucgaagacgaucagaua..........................................................................    3       1    seq
.ggggcucgaagacgGucagaua..........................................................................    3       1    seq
.ggggcucgaagacgaucagGua..........................................................................    6       1    seq
.ggggcucgaagGcgaucagaua..........................................................................    3       1    seq
.ggAgcucgaagacgaucagaua..........................................................................    1       1    seq
.ggggcucgaagacgaucGgaua..........................................................................    11      1    seq
.ggggcCcgaagacgaucagaua..........................................................................    7       1    seq
.ggggcucgaagacgaucagaCa..........................................................................    21      1    seq
.ggggcucgUagacgaucagaua..........................................................................    2       1    seq
.ggggcucgaagacUaucagaua..........................................................................    2       1    seq
.ggggcucgaagacgauGagaua..........................................................................    1       1    seq
.ggggcucgaagacgaucagauU..........................................................................    9       1    seq
.ggggcucgaagacgaCcagaua..........................................................................    6       1    seq
.ggggcucgaUgacgaucagaua..........................................................................    1       1    seq
.Cgggcucgaagacgaucagaua..........................................................................    1       1    seq
.ggggcucUaagacgaucagaua..........................................................................    2       1    seq
.gUggcucgaagacgaucagaua..........................................................................    2       1    seq
.ggggcucgaagaGgaucagaua..........................................................................    1       1    seq
.ggggcucgaagacgaucagaGa..........................................................................    1       1    seq
.gggUcucgaagacgaucagaua..........................................................................    1       1    seq
.ggggcucgaagacgaucagCua..........................................................................    1       1    seq
.ggggcucgaagacgauUagaua..........................................................................    5       1    seq
.gAggcucgaagacgaucagaua..........................................................................    4       1    seq
.ggggcucgaagacgaucagauaA.........................................................................    1       1    seq
.ggggcucgaagacgaucagauac.........................................................................    278     0    seq
.ggggcucgaGgacgaucagauac.........................................................................    2       1    seq
.ggggcucgaagGcgaucagauac.........................................................................    3       1    seq
.ggggcucgaagacgaucagauGc.........................................................................    2       1    seq
.ggggcucgaagacgaucGgauac.........................................................................    1       1    seq
.ggggcCcgaagacgaucagauac.........................................................................    1       1    seq
.Agggcucgaagacgaucagauac.........................................................................    1       1    seq
.ggggcucgaagacgaucagaCac.........................................................................    3       1    seq
.ggggcucgaUgacgaucagauac.........................................................................    1       1    seq
.ggggcuGgaagacgaucagauac.........................................................................    1       1    seq
.ggggcucgaagacgaucagauaU.........................................................................    9       1    seq
.ggggcucgGagacgaucagauac.........................................................................    1       1    seq
.ggggcucgaagacgaucagaCacc........................................................................    1       1    seq
.ggggcucgaagacgaucagUuacc........................................................................    1       1    seq
.ggggcucgaagacgaCcagauacc........................................................................    1       1    seq
.ggggcucgaagacgaGcagauacc........................................................................    1       1    seq
.ggggcucgaagacgaucagauaAc........................................................................    1       1    seq
.ggggcucgaagacgaucGgauacc........................................................................    2       1    seq
.ggggcucgaagacgaucagauacc........................................................................    577     0    seq
.ggggcucgaagacgaucagauacG........................................................................    1       1    seq
.ggggcucgaagacgaucagauacA........................................................................    13      1    seq
.ggggcucgaagacgaAcagauacc........................................................................    1       1    seq
.ggggcucgaagacgGucagauacc........................................................................    2       1    seq
.ggggcAcgaagacgaucagauacc........................................................................    1       1    seq
.ggggcucgaGgacgaucagauacc........................................................................    1       1    seq
.ggggcuUgaagacgaucagauacc........................................................................    2       1    seq
.ggggcucgaagGcgaucagauacc........................................................................    2       1    seq
.ggggUucgaagacgaucagauacc........................................................................    1       1    seq
.ggggcucgaagacgaucagGuacc........................................................................    1       1    seq
.gUggcucgaagacgaucagauacc........................................................................    1       1    seq
.ggggcucgaagacgaucagauacca.......................................................................    120     0    seq
.ggggcucgGagacgaucagauacca.......................................................................    1       1    seq
.ggggcucgaagacgaucagauaccC.......................................................................    11      1    seq
.ggggcucgaagacgaCcagauacca.......................................................................    1       1    seq
.ggggcucUaagacgaucagauacca.......................................................................    1       1    seq
.ggggcucgaagacgaucagauaccU.......................................................................    19      1    seq
.ggggcucgaagacgaucagauacAa.......................................................................    1       1    seq
.ggggcucgaagacgaucagGuacca.......................................................................    1       1    seq
```

Mature **Star**

```
gggggcucgaagacgaucagauaccauccuagucucaaccauaaaugaugucgaccagcgauugguggauguuucucuaaggacuccggcagcacc
.ggggcucgaagacgaucagauacGa......................................................................   1      1   seq
.ggggcucgaGgacgaucagauacca......................................................................   2      1   seq
.ggggcucgaagacgaucagauaccauc....................................................................   1      0   seq
.ggggcucgaagacgaucagauaccUuc....................................................................   1      1   seq
.ggggcucgaagacgaucagauaccaAc....................................................................   1      1   seq
.ggggcucgaagacgaucagauaccCucc...................................................................   5      1   seq
.ggggcucgaagacgaucagauaccauccua.................................................................   1      0   seq
.ggggcucgaagacgaucagauaccUuccua.................................................................   1      1   seq
..gggcucgaagaGgauca.............................................................................   3      1   seq
..Cggcucgaagacgauca.............................................................................   17     1   seq
..gCgcucgaagacgauca.............................................................................   3      1   seq
..gggcucgaagacgauUa.............................................................................   16     1   seq
..gggcucgaagacgauca.............................................................................   30483  0   seq
..ggCcucgaagacgauca.............................................................................   1      1   seq
..gggcucUaagacgauca.............................................................................   5      1   seq
..gggcucgaagacgaucG.............................................................................   868    1   seq
..gggcCcgaagacgauca.............................................................................   74     1   seq
..gggcucgaagacgCuca.............................................................................   11     1   seq
..gggcucgaaUacgauca.............................................................................   1      1   seq
..ggAcucgaagacgauca.............................................................................   44     1   seq
..gggcucgaagacgaAca.............................................................................   15     1   seq
..gggcucgCagacgauca.............................................................................   3      1   seq
..gggcucgaagacgaucU.............................................................................   174    1   seq
..gggcucgaagCcgauca.............................................................................   3      1   seq
..gggcucgaagacgauGa.............................................................................   6      1   seq
..gUgcucgaagacgauca.............................................................................   8      1   seq
..gggcucgaagUcgauca.............................................................................   4      1   seq
..gggcAcgaagacgauca.............................................................................   13     1   seq
..gggcucCaagacgauca.............................................................................   1      1   seq
..gggcucgaGgacgauca.............................................................................   125    1   seq
..Nggcucgaagacgauca.............................................................................   34     1   seq
..gggcucgUagacgauca.............................................................................   7      1   seq
..gggcucgaagacgaCca.............................................................................   190    1   seq
..gggcuGgaagacgauca.............................................................................   1      1   seq
..gggcucgaagacgGuca.............................................................................   123    1   seq
..gggcucgGagacgauca.............................................................................   81     1   seq
..gggGucgaagacgauca.............................................................................   7      1   seq
..gggcucgaagacgaucC.............................................................................   41     1   seq
..gggcucgaaAacgauca.............................................................................   8      1   seq
..Uggcucgaagacgauca.............................................................................   23     1   seq
..gggcucgaagacgUuca.............................................................................   16     1   seq
..gggcucgaagacUauca.............................................................................   12     1   seq
..gggcucgaagacgaGca.............................................................................   35     1   seq
..gggcGcgaagacgauca.............................................................................   3      1   seq
..gggcucgaUgacgauca.............................................................................   10     1   seq
..gggcucgaagacgauAa.............................................................................   3      1   seq
..gggUucgaagacgauca.............................................................................   28     1   seq
..Aggcucgaagacgauca.............................................................................   49     1   seq
..ggUcucgaagacgauca.............................................................................   8      1   seq
..gggcuAgaagacgauca.............................................................................   3      1   seq
..gggcucgaagacgaucCg............................................................................   2      1   seq
..gggcucUaagacgaucag............................................................................   1      1   seq
..gggcuGgaagacgaucag............................................................................   1      1   seq
..gggcucgaagacUaucag............................................................................   1      1   seq
..Aggcucgaagacgaucag............................................................................   11     1   seq
..gggcucgaagGcgaucag............................................................................   15     1   seq
..gggcucgaagacgaucUg............................................................................   4      1   seq
..gggGucgaagacgaucag............................................................................   1      1   seq
..gggcucgaagacgauUag............................................................................   6      1   seq
..ggUcucgaagacgaucag............................................................................   1      1   seq
..gggcucgaagacgaGcag............................................................................   1      1   seq
..gUgcucgaagacgaucag............................................................................   2      1   seq
..gggcucgaagacgUucag............................................................................   3      1   seq
..gggcucgGagacgaucag............................................................................   23     1   seq
..Nggcucgaagacgaucag............................................................................   1      1   seq
..gggUucgaagacgaucag............................................................................   5      1   seq
..gCgcucgaagacgaucag............................................................................   2      1   seq
..gggcucgaaUacgaucag............................................................................   3      1   seq
..gggcucgaGgacgaucag............................................................................   19     1   seq
..gggcucgaagacgauGag............................................................................   1      1   seq
```

Mature **Star**

ggggcucgaagacgaucagauaccauccuagucucaaccauaaaugaugucgaccagcgauugguggauguuucucuaaggacuccggcagcacc			
..gAgcucgaagacgaucag...	4	1	seq
..gggcucgaagacgaAcag...	1	1	seq
..gggcucgaagacgGucag...	14	1	seq
..gggcucgaagacgaucGg...	30	1	seq
..gggcucgaagacgaucag...	5766	0	seq
..gggcucgaagUcgaucag...	1	1	seq
..gggcucgUagacgaucag...	2	1	seq
..gggcCcgaagacgaucag...	14	1	seq
..Cggcucgaagacgaucag...	1	1	seq
..gggcuAgaagacgaucag...	1	1	seq
..ggAcucgaagacgaucag...	5	1	seq
..gggcucgaagacgCucag...	1	1	seq
..gggcucgaagaAgaucag...	2	1	seq
..gggcucgaagacgaCcag...	16	1	seq
..gggcAcgaagacgaucag...	2	1	seq
..gggcucgaagacgaucaC...	14	1	seq
..Uggcucgaagacgaucag...	12	1	seq
..gggcucgaUgacgaucaga..	7	1	seq
..gggcucgaGgacgaucaga..	16	1	seq
..gggcucgaagacgaAcaga..	3	1	seq
..Cggcucgaagacgaucaga..	4	1	seq
..gggcucgaagCcgaucaga..	2	1	seq
..Nggcucgaagacgaucaga..	6	1	seq
..gggcucgaagacUaucaga..	1	1	seq
..gggcuGgaagacgaucaga..	1	1	seq
..ggUcucgaagacgaucaga..	2	1	seq
..gggcucUaagacgaucaga..	1	1	seq
..Aggcucgaagacgaucaga..	14	1	seq
..gggcucgaagacgaucUga..	3	1	seq
..gggcucgGagacgaucaga..	17	1	seq
..gUgcucgaagacgaucaga..	2	1	seq
..gggcucgaagacgaucagG..	88	1	seq
..gggcucgaagacgauGaga..	2	1	seq
..gggcucgaagacgaucagC..	4	1	seq
..gggcucgaagacgauUaga..	7	1	seq
..gggcuAgaagacgaucaga..	1	1	seq
..gggcGcgaagacgaucaga..	2	1	seq
..gAgcucgaagacgaucaga..	4	1	seq
..gggcucCaagacgaucaga..	1	1	seq
..gggcucgaagacgaucaga..	4731	0	seq
..gggcucgaagacgaCcaga..	10	1	seq
..gggcucgaagacgGucaga..	15	1	seq
..gggcucgaagacgaucCga..	3	1	seq
..gggcAcgaagacgaucaga..	3	1	seq
..gggcucgaagacgaucaCa..	6	1	seq
..gggcucgaagacgaGcaga..	2	1	seq
..gggcucgaagaGgaucaga..	1	1	seq
..gggcucgaagacgaucagU..	29	1	seq
..ggAcucgaagacgaucaga..	8	1	seq
..gggcucgaagGcgaucaga..	16	1	seq
..gggcucgaagacgaucGga..	15	1	seq
..gggcCcgaagacgaucaga..	16	1	seq
..Uggcucgaagacgaucaga..	5	1	seq
..gggUucgaagacgaucagau...	2	1	seq
..gggcucgaagacgaucagaA...	12	1	seq
..gggcucgaagacgGucagau...	7	1	seq
..gggcucgaagacgaucagCu...	1	1	seq
..gggcucgaagacgaucagaC...	206	1	seq
..Uggcucgaagacgaucagau...	1	1	seq
..gggcucgaagGcgaucagau...	4	1	seq
..ggCcucgaagacgaucagau...	1	1	seq
..gggcucgaUgacgaucagau...	1	1	seq
..Aggcucgaagacgaucagau...	4	1	seq
..ggAcucgaagacgaucagau...	2	1	seq
..gggcCcgaagacgaucagau...	4	1	seq
..gggcucgUagacgaucagau...	2	1	seq
..gggcucgaagacgaucagGu...	7	1	seq
..gggcucgaagacgaucaCau...	1	1	seq
..gggcucgGagacgaucagau...	7	1	seq
..gggcucgaagacgaCcagau...	3	1	seq

Mature   Star

```
ggggcucgaagacgaucagauaccauccuagucucaaccauaaaugaugucgaccagcgauugguggauguuucucuaaggacuccggcagcacc
..gggcucgaagacgaAcagau.........................................................................	1	1	seq
..gggcucgaagacgaucagaG.........................................................................	20	1	seq
..Nggcucgaagacgaucagau.........................................................................	3	1	seq
..gggcucgaGgacgaucagau.........................................................................	10	1	seq
..gggcucgaagacgaucagUu.........................................................................	1	1	seq
..gggcucgaagacgauGagau.........................................................................	1	1	seq
..ggUcucgaagacgaucagau.........................................................................	1	1	seq
..gggcucgaaUacgaucagau.........................................................................	1	1	seq
..gggcucgaagacgaucagau.........................................................................	1622	0	seq
..gggcucgaagacgaucGgau.........................................................................	1	1	seq
..gggcucgaagacgaucUgau.........................................................................	4	1	seq
..gggcucgaagacgaucCgaua........................................................................	1	1	seq
..gggcucgaagacgGucagaua........................................................................	9	1	seq
..gggcucgGagacgaucagaua........................................................................	11	1	seq
..ggAcucgaagacgaucagaua........................................................................	3	1	seq
..Aggcucgaagacgaucagaua........................................................................	3	1	seq
..Uggcucgaagacgaucagaua........................................................................	1	1	seq
..gggUucgaagacgaucagaua........................................................................	2	1	seq
..gggcuAgaagacgaucagaua........................................................................	1	1	seq
..gggcucgaGgacgaucagaua........................................................................	12	1	seq
..gggcucgaagacgaCcagaua........................................................................	7	1	seq
..gggcucgaagacgaucagaua........................................................................	2652	0	seq
..gggcCcgaagacgaucagaua........................................................................	4	1	seq
..Cggcucgaagacgaucagaua........................................................................	2	1	seq
..gggGucgaagacgaucagaua........................................................................	1	1	seq
..gggcucgaagacgaucagaGa........................................................................	1	1	seq
..gAgcucgaagacgaucagaua........................................................................	6	1	seq
..gggcucgaagacgaucGgaua........................................................................	6	1	seq
..gggcucgaagGcgaucagaua........................................................................	7	1	seq
..gggcucgaagacgaucagGua........................................................................	7	1	seq
..gggcucgaagacgaucagauU........................................................................	6	1	seq
..gggcucgaaUacgaucagaua........................................................................	1	1	seq
..gggcucgaagacgaucagUua........................................................................	1	1	seq
..gggcucgaagacgaucagaCa........................................................................	13	1	seq
..gggcucgaagacgaGcagaua........................................................................	1	1	seq
..gggcucgaagacgaucagauG........................................................................	24	1	seq
..gggAucgaagacgaucagaua........................................................................	1	1	seq
..gggcucgaagacgCucagaua........................................................................	1	1	seq
..gggcucgaagacgaucUgaua........................................................................	1	1	seq
..gggcucgUagacgaucagaua........................................................................	2	1	seq
..gUgcucgaagacgaucagaua........................................................................	1	1	seq
..gggcucgaaUacgaucagauac.......................................................................	1	1	seq
..gggcucgaagUcgaucagauac.......................................................................	1	1	seq
..Nggcucgaagacgaucagauac.......................................................................	1	1	seq
..gggcucgaagacgaucagauac.......................................................................	244	0	seq
..gggcucgaagaGgaucagauac.......................................................................	1	1	seq
..gggcucgaagacgaucagauGc.......................................................................	2	1	seq
..gggcucgaagacgaucagauaU.......................................................................	9	1	seq
..gggcucgaagacgaucGgauac.......................................................................	2	1	seq
..gggcucgaagacgaucagauaA.......................................................................	2	1	seq
..gggcucgaagacgaucagauacG......................................................................	1	1	seq
..gggcucgaagacgaucagauacc......................................................................	103	0	seq
..gggcucgaagacgaucagaCacc......................................................................	1	1	seq
..gggcucgaagacgaucagauacA......................................................................	4	1	seq
..gggcucgaagacgaucagauaccC.....................................................................	3	1	seq
..gggcucgaagacgaucagauacca.....................................................................	9	0	seq
..gggcucgaagacgaucagauaccU.....................................................................	1	1	seq
..gggcucgaagacgaucagaCacca.....................................................................	1	1	seq
..gggcucgaagacgaucagauaccaucc..................................................................	1	0	seq
..gggcucgaagacgaucagauaccUuccua................................................................	2	1	seq
..gggcucgaagacgaucagauaccauccuagu..............................................................	1	0	seq
..gggcucgaagacgaucagauaccauccuagucu............................................................	1	0	seq
...ggGucgaagacgaucag...........................................................................	1	1	seq
...gAcucgaagacgaucag...........................................................................	2	1	seq
...ggcCcgaagacgaucag...........................................................................	1	1	seq
...Ugcucgaagacgaucag...........................................................................	2	1	seq
...ggcucgaagacgaucGg...........................................................................	5	1	seq
...ggcucgaagacgaucag...........................................................................	927	0	seq
...ggcucgaGgacgaucag...........................................................................	7	1	seq
...ggcucgaagacgaCcag...........................................................................	1	1	seq
```

```
 Mature                                                                         Star
gggggcucgaagacgaucagauaccauccuagucucaaccauaaaaugaugucgaccagcgauugguggauguuucucuaaggacuccggcagcacc
...ggcucgaaAacgaucag.............................................................................  1     1   seq
...ggcucgaagacgGucag.............................................................................  3     1   seq
...ggcAcgaagacgaucag.............................................................................  1     1   seq
...ggcucgaagaAgaucag.............................................................................  2     1   seq
...Ngcucgaagacgaucag.............................................................................  1     1   seq
...ggcucgaagGcgaucag.............................................................................  4     1   seq
...ggcucgaagacgUucag.............................................................................  2     1   seq
...ggcucgaagacgaucaC.............................................................................  2     1   seq
...ggcucgGagacgaucag.............................................................................  7     1   seq
...Agcucgaagacgaucag.............................................................................  1     1   seq
...ggcucgaagacgauUag.............................................................................  3     1   seq
...ggcucgaagacgaucUg.............................................................................  1     1   seq
...ggcucgaagacgaucaga............................................................................  2212  0   seq
...gUcucgaagacgaucaga............................................................................  1     1   seq
...ggcCcgaagacgaucaga............................................................................  7     1   seq
...Ngcucgaagacgaucaga............................................................................  1     1   seq
...ggGucgaagacgaucaga............................................................................  3     1   seq
...ggcucgaagacgaucGga............................................................................  8     1   seq
...gAcucgaagacgaucaga............................................................................  6     1   seq
...Cgcucgaagacgaucaga............................................................................  2     1   seq
...ggcucgaagacgaucagC............................................................................  3     1   seq
...ggcucgGagacgaucaga............................................................................  9     1   seq
...ggcucgaagacUaucaga............................................................................  2     1   seq
...ggcucgaagacgaucUga............................................................................  2     1   seq
...ggcucgaagacgaCcaga............................................................................  7     1   seq
...Ugcucgaagacgaucaga............................................................................  1     1   seq
...Agcucgaagacgaucaga............................................................................  2     1   seq
...ggcucgaagGcgaucaga............................................................................  7     1   seq
...ggcucgaagacgGucaga............................................................................  6     1   seq
...ggcucgaagacgUucaga............................................................................  2     1   seq
...ggcucgaagacgaucaCa............................................................................  2     1   seq
...ggcucgaGgacgaucaga............................................................................  3     1   seq
...ggcucgaagacgaucagU............................................................................  13    1   seq
...ggUucgaagacgaucaga............................................................................  3     1   seq
...ggcucgaagacgCucaga............................................................................  2     1   seq
...ggcucgaagacgaucagG............................................................................  42    1   seq
...ggcuGgaagacgaucaga............................................................................  1     1   seq
...ggcAcgaagacgaucaga............................................................................  3     1   seq
...ggcGcgaagacgaucaga............................................................................  2     1   seq
...ggcucgaaUacgaucaga............................................................................  1     1   seq
...ggcucgaagacgaucCga............................................................................  1     1   seq
...ggcucgaUgacgaucaga............................................................................  1     1   seq
...ggcucgaagacgauUaga............................................................................  2     1   seq
...ggcucgaUgacgaucagau...........................................................................  1     1   seq
...ggcucgaagacgaucagau...........................................................................  2185  0   seq
...ggcucgCagacgaucagau...........................................................................  1     1   seq
...ggcucgaagacgaucagCu...........................................................................  1     1   seq
...gAcucgaagacgaucagau...........................................................................  1     1   seq
...ggcAcgaagacgaucagau...........................................................................  6     1   seq
...ggcucgaGgacgaucagau...........................................................................  4     1   seq
...ggcucgaagacgaCcagau...........................................................................  4     1   seq
...ggcucgaagacgaucagaG...........................................................................  20    1   seq
...ggcucgGagacgaucagau...........................................................................  8     1   seq
...ggcucgaagacgaucagaA...........................................................................  7     1   seq
...ggcucgaagGcgaucagau...........................................................................  2     1   seq
...Agcucgaagacgaucagau...........................................................................  3     1   seq
...ggcucgaagacgGucagau...........................................................................  4     1   seq
...ggcucgaagacgaucagGu...........................................................................  9     1   seq
...Ngcucgaagacgaucagau...........................................................................  4     1   seq
...ggcucgUagacgaucagau...........................................................................  1     1   seq
...ggcucgaagUcgaucagau...........................................................................  1     1   seq
...ggcucgaagacgaucUgau...........................................................................  1     1   seq
...ggcucgaagacgaGcagau...........................................................................  1     1   seq
...ggcucgaagacgUucagau...........................................................................  2     1   seq
...Ugcucgaagacgaucagau...........................................................................  2     1   seq
...ggcuGgaagacgaucagau...........................................................................  3     1   seq
...ggcucgaagacgaucGgau...........................................................................  8     1   seq
...ggcucgaagacUaucagau...........................................................................  1     1   seq
...ggGucgaagacgaucagau...........................................................................  1     1   seq
...ggcGcgaagacgaucagau...........................................................................  2     1   seq
```

Mature **Star**

```
gggggcucgaagacgaucagauaccauccuagucucaaccauaaaaugaugucgaccagcgauugguggauguuucucuaaggacuccggcagcacc
...ggcucgaagacgCucagau...........................................................................  1     1  seq
...ggcucgaagacgauUagau...........................................................................  3     1  seq
...ggcucgaagaAgaucagau...........................................................................  1     1  seq
...ggcCcgaagacgaucagau...........................................................................  2     1  seq
...ggcucgaagacgaucagaC...........................................................................  261   1  seq
...Agcucgaagacgaucagaua..........................................................................  2     1  seq
...ggcucgaagacgGucagaua..........................................................................  6     1  seq
...ggcucgaagacgaucagauG..........................................................................  14    1  seq
...ggcucgaagGcgaucagaua..........................................................................  5     1  seq
...ggcucgaagacgaGcagaua..........................................................................  1     1  seq
...ggcucgaagaGgaucagaua..........................................................................  2     1  seq
...gAcucgaagacgaucagaua..........................................................................  2     1  seq
...ggcucgaagacgaAcagaua..........................................................................  1     1  seq
...Ugcucgaagacgaucagaua..........................................................................  2     1  seq
...ggcucgaagacgaucCgaua..........................................................................  1     1  seq
...ggcucgaagacgauUagaua..........................................................................  3     1  seq
...ggcuGgaagacgaucagaua..........................................................................  1     1  seq
...ggcucgaagacgaucagaCa..........................................................................  12    1  seq
...ggcucgaUgacgaucagaua..........................................................................  2     1  seq
...ggcucgaagacgaucagaGa..........................................................................  2     1  seq
...ggcucgaagacgaCcagaua..........................................................................  4     1  seq
...ggcucgaagacgaucagaua..........................................................................  1727  0  seq
...ggcucgGagacgaucagaua..........................................................................  4     1  seq
...ggcucgaagacgaucGgaua..........................................................................  3     1  seq
...ggcucgaagacgaucagGua..........................................................................  2     1  seq
...Ngcucgaagacgaucagaua..........................................................................  3     1  seq
...ggcCcgaagacgaucagaua..........................................................................  10    1  seq
...ggcucgaGgacgaucagaua..........................................................................  7     1  seq
...ggcucgaagacgaucagauU..........................................................................  2     1  seq
...gUcucgaagacgaucagaua..........................................................................  1     1  seq
...gNcucgaagacgaucagaua..........................................................................  1     1  seq
...ggcucgaagaAgaucagaua..........................................................................  1     1  seq
...ggcucgaagacgaucagaAa..........................................................................  1     1  seq
...ggcAcgaagacgaucagaua..........................................................................  2     1  seq
...ggGucgaagacgaucagaua..........................................................................  1     1  seq
...ggcucgaagacUaucagaua..........................................................................  1     1  seq
...ggUucgaagacgaucagaua..........................................................................  1     1  seq
...ggcucgaaAacgaucagaua..........................................................................  1     1  seq
...ggcCcgaagacgaucagauac.........................................................................  1     1  seq
...ggcucgaGgacgaucagauac.........................................................................  2     1  seq
...ggcucgaagacgaucagauac.........................................................................  196   0  seq
...ggcucgaagUcgaucagauac.........................................................................  1     1  seq
...ggcucgaagacgaCcagauac.........................................................................  1     1  seq
...ggcucgaUgacgaucagauac.........................................................................  2     1  seq
...ggcucgaagacgaucagaCac.........................................................................  1     1  seq
...ggcucgaagacgaucCgauac.........................................................................  1     1  seq
...Ngcucgaagacgaucagauac.........................................................................  1     1  seq
...ggcucgaagacgaucagauaU.........................................................................  6     1  seq
...ggcucgaagacgaucagauaA.........................................................................  1     1  seq
...ggcucgaagGcgaucagauac.........................................................................  1     1  seq
...ggcucgaGgacgaucagauacc........................................................................  2     1  seq
...ggcCcgaagacgaucagauacc........................................................................  1     1  seq
...ggcucgaagacgaucagauacG........................................................................  1     1  seq
...ggcucgaagacgaucagauacA........................................................................  1     1  seq
...ggcucgaagaGgaucagauacc........................................................................  1     1  seq
...ggcucgaagacgaucagauacc........................................................................  45    0  seq
...ggcucgaagacgaucagauaccC.......................................................................  2     1  seq
...ggcucgaagacgaucagauacca.......................................................................  16    0  seq
...ggcucgaagacgaucagauaccU.......................................................................  3     1  seq
...ggcucgaagacgaucagauaccCucc....................................................................  1     1  seq
...ggcucgaagacgaucagauaccauccu...................................................................  1     0  seq
...ggcucgaagacgaucagauaccauccua..................................................................  1     0  seq
....Ncucgaagacgaucaga............................................................................  2     1  seq
....gcucgaagGcgaucaga............................................................................  1     1  seq
....gcucgGagacgaucaga............................................................................  1     1  seq
....gcucgaagacgaGcaga............................................................................  1     1  seq
....gcucgaagacgaucagG............................................................................  5     1  seq
....gcucgaagacgaucaga............................................................................  255   0  seq
....Ucucgaagacgaucaga............................................................................  1     1  seq
....gcucgaagacgGucaga............................................................................  1     1  seq
```

```
 Mature                                                                        Star
gggggcucgaagacgaucagauaccauccuagucucaaccauaaaugaugucgaccagcgauugguggauguuucucuaaggacuccggcagcacc
....gcucgaagacgaAcaga...........................................................................    2     1    seq
....gcucgaagacgaucagU...........................................................................    1     1    seq
....gcAcgaagacgaucagau..........................................................................    1     1    seq
....gAucgaagacgaucagau..........................................................................    1     1    seq
....gcucgaagGcgaucagau..........................................................................    2     1    seq
....gcucgaagacgaucagau..........................................................................    356   0    seq
....gcucgaagacgGucagau..........................................................................    2     1    seq
....gcucgaagacgaucGgau..........................................................................    1     1    seq
....gcucgGagacgaucagau..........................................................................    1     1    seq
....gcucgaagacgaGcagau..........................................................................    1     1    seq
....gcucgaagacgaucUgau..........................................................................    1     1    seq
....gcucgaagCcgaucagau..........................................................................    1     1    seq
....gcucgaagacgaucagaA..........................................................................    2     1    seq
....gcucgaagacgUucagau..........................................................................    1     1    seq
....gcucgaagacgaucagaC..........................................................................    59    1    seq
....gcucgaagacgaucagaG..........................................................................    4     1    seq
....gcucgGagacgaucagaua.........................................................................    2     1    seq
....gcuAgaagacgaucagaua.........................................................................    1     1    seq
....gcucgaagGcgaucagaua.........................................................................    4     1    seq
....gcucgUagacgaucagaua.........................................................................    1     1    seq
....gcucgaagacgaucagGua.........................................................................    3     1    seq
....Ucucgaagacgaucagaua.........................................................................    2     1    seq
....gcucgaagacgaCcagaua.........................................................................    1     1    seq
....gcucgaagacgaucagUua.........................................................................    1     1    seq
....gcucgaagacgaucagauG.........................................................................    12    1    seq
....gcucgaagacgaucagauU.........................................................................    3     1    seq
....gcucgaagacgauGagaua.........................................................................    1     1    seq
....gcucgaagacgauUagaua.........................................................................    3     1    seq
....gcucgaGgacgaucagaua.........................................................................    2     1    seq
....gcucCaagacgaucagaua.........................................................................    1     1    seq
....gcucgaagacgaucagaua.........................................................................    1185  0    seq
....gcCcgaagacgaucagaua.........................................................................    1     1    seq
....gcucgaagacUaucagaua.........................................................................    1     1    seq
....gcucgaagacgaucGgaua.........................................................................    1     1    seq
....gcucgaagacgGucagaua.........................................................................    3     1    seq
....gcucgaagacgaGcagaua.........................................................................    1     1    seq
....gUucgaagacgaucagaua.........................................................................    2     1    seq
....gcucgaagacgaucagaCa.........................................................................    7     1    seq
....Acucgaagacgaucagaua.........................................................................    3     1    seq
....gcucgaagacgaucUgaua.........................................................................    1     1    seq
....gcucgaagacgaucagaAa.........................................................................    1     1    seq
....Ucucgaagacgaucagauac........................................................................    2     1    seq
....Ncucgaagacgaucagauac........................................................................    1     1    seq
....gcucgaGgacgaucagauac........................................................................    3     1    seq
....gcucgaagacgaucGgauac........................................................................    1     1    seq
....gcucgaagacgaucagauaU........................................................................    4     1    seq
....gcucgaagacgaucagauaG........................................................................    1     1    seq
....gcucgaagacgaucagauaA........................................................................    1     1    seq
....gcucgaagacgaucagGuac........................................................................    1     1    seq
....gcucgaagacgaucUgauac........................................................................    1     1    seq
....gcucgaagacgaucagauac........................................................................    138   0    seq
....gcucgaagacgaucagUuacc.......................................................................    1     1    seq
....gcucgaagacgaucagauGcc.......................................................................    3     1    seq
....gcucgaGgacgaucagauacc.......................................................................    1     1    seq
....gcucgCagacgaucagauacc.......................................................................    1     1    seq
....Ucucgaagacgaucagauacc.......................................................................    1     1    seq
....gcucgaagacgaucagauacc.......................................................................    273   0    seq
....gcucgaagacgaucagauacA.......................................................................    7     1    seq
....gcucgGagacgaucagauacc.......................................................................    1     1    seq
....gcucgaagacgauUagauacc.......................................................................    1     1    seq
....gcAcgaagacgaucagauacc.......................................................................    1     1    seq
....gcucgaagacgaCcagauacc.......................................................................    1     1    seq
....gcucgaagGcgaucagauacc.......................................................................    1     1    seq
....gcucgaagacgaucagauaccC......................................................................    1     1    seq
....gcucgaagacgaucagauacca......................................................................    22    0    seq
....gcucgaagacgaucagauaccU......................................................................    4     1    seq
....Acucgaagacgaucagauacca......................................................................    1     1    seq
....gcucgaagacgaucagauaccCucc...................................................................    1     1    seq
....gcucgaagacgaucagauaccUuccua.................................................................    1     1    seq
.....cucgaGgacgaucagau..........................................................................    2     1    seq
```

Mature   Star

```
gggggcucgaagacgaucagauaccauccuagucucaaccauaaaugaugucgaccagcgauugguggauguuucucuaaggacuccggcagcacc
.....cucgaagacgaucagaA..........................................................................    1      1    seq
.....Aucgaagacgaucagau..........................................................................    1      1    seq
.....cucgaagacgGucagau..........................................................................    1      1    seq
.....Uucgaagacgaucagau..........................................................................    1      1    seq
.....cucgGagacgaucagau..........................................................................    1      1    seq
.....cucgaagacgaucagaG..........................................................................    4      1    seq
.....cucgaagacgaucagau..........................................................................    627    0    seq
.....cucgaagacgaucagGu..........................................................................    2      1    seq
.....cuGgaagacgaucagau..........................................................................    1      1    seq
.....cucgaagacgaucGgau..........................................................................    1      1    seq
.....cucgaagacgaucagaC..........................................................................    87     1    seq
.....cucgaagGcgaucagau..........................................................................    2      1    seq
.....Nucgaagacgaucagau..........................................................................    1      1    seq
.....cucgaagacgaGcagaua.........................................................................    1      1    seq
.....cucgaagacgaucaCaua.........................................................................    1      1    seq
.....cCcgaagacgaucagaua.........................................................................    7      1    seq
.....cucgaagacgaucagUua.........................................................................    1      1    seq
.....Uucgaagacgaucagaua.........................................................................    2      1    seq
.....cAcgaagacgaucagaua.........................................................................    1      1    seq
.....cucgaagacgaucagaCa.........................................................................    11     1    seq
.....cucgaGgacgaucagaua.........................................................................    3      1    seq
.....cucgaagacgaucagaua.........................................................................    1365   0    seq
.....cucgaagacgGucagaua.........................................................................    4      1    seq
.....cucgaagacgaucUgaua.........................................................................    1      1    seq
.....cucgaagacgaucagauU.........................................................................    5      1    seq
.....Gucgaagacgaucagaua.........................................................................    2      1    seq
.....cucgaagacgaucagGua.........................................................................    3      1    seq
.....cucgaagacgaCcagaua.........................................................................    1      1    seq
.....cucgaagGcgaucagaua.........................................................................    5      1    seq
.....cucgaagacgaucagauG.........................................................................    16     1    seq
.....cuAgaagacgaucagaua.........................................................................    1      1    seq
.....cucgaaAacgaucagaua.........................................................................    1      1    seq
.....cucgaagacgaucGgaua.........................................................................    6      1    seq
.....cucgaagacgaucagaGa.........................................................................    2      1    seq
.....cucgGagacgaucagaua.........................................................................    6      1    seq
.....cucgaagGcgaucagauac........................................................................    1      1    seq
.....cucgaagacgaucagauaA........................................................................    1      1    seq
.....cucgaagacgaucUgauac........................................................................    1      1    seq
.....cucgaagacgaucagauaU........................................................................    9      1    seq
.....cucgaagacgaucagauac........................................................................    211    0    seq
.....Aucgaagacgaucagauac........................................................................    1      1    seq
.....cucgaagacgaucagGuac........................................................................    1      1    seq
.....cucgaagacgGucagauac........................................................................    1      1    seq
.....cucgaagacgaucGgauacc.......................................................................    3      1    seq
.....cucgaagacgaucagauaUc.......................................................................    2      1    seq
.....cucgaGgacgaucagauacc.......................................................................    1      1    seq
.....cucgaagacgaucagaCacc.......................................................................    1      1    seq
.....cCcgaagacgaucagauacc.......................................................................    1      1    seq
.....cucgaagacgaucagauacc.......................................................................    374    0    seq
.....cucgaagacgaucagauacA.......................................................................    8      1    seq
.....cucgGagacgaucagauacc.......................................................................    1      1    seq
.....cucgaagacgaucUgauacc.......................................................................    1      1    seq
.....cucCaagacgaucagauacc.......................................................................    1      1    seq
.....Gucgaagacgaucagauacc.......................................................................    1      1    seq
.....cucgaagacgaCcagauacc.......................................................................    1      1    seq
.....cucgaagacgaucagGuacc.......................................................................    1      1    seq
.....cucgaagacgaucagauGcc.......................................................................    2      1    seq
.....cucgaagacgaucagauacca......................................................................    27     0    seq
.....cucgaagacgaucagauaccU......................................................................    3      1    seq
.....cucgaagacgaucGgauacca......................................................................    1      1    seq
.....cucgaagacgaucagauaccC......................................................................    6      1    seq
......ucgaagacgaucUgaua.........................................................................    2      1    seq
......ucgaagacgaCcagaua.........................................................................    4      1    seq
......ucgaagacgaucagauG.........................................................................    7      1    seq
......ucgaagacgaucGgaua.........................................................................    1      1    seq
......ucgaagacgaucagGua.........................................................................    2      1    seq
......ucgaagacgGucagaua.........................................................................    3      1    seq
......Ncgaagacgaucagaua.........................................................................    2      1    seq
......ucgCagacgaucagaua.........................................................................    1      1    seq
......uGgaagacgaucagaua.........................................................................    1      1    seq
```

```
 Mature                                                                    Star
gggggcucgaagacgaucagauaccauccuagucucaaccauaaaugaugucgaccagcgauugguggauguuucucuaaggacuccggcagcacc
......ucgUagacgaucagaua.........................................................................    1       1       seq
......ucgaGgacgaucagaua.........................................................................    6       1       seq
......ucgaagacgaucagauC.........................................................................    1       1       seq
......ucgaagacgaucagaGa.........................................................................    1       1       seq
......ucgaagacgaucagCua.........................................................................    1       1       seq
......ucgaagacgaAcagaua.........................................................................    1       1       seq
......ucgaagGcgaucagaua.........................................................................    3       1       seq
......ucgGagacgaucagaua.........................................................................    3       1       seq
......ucgaagacgaucagaCa.........................................................................    8       1       seq
......ucgaagacgaucagaua.........................................................................    1583    0       seq
......Ccgaagacgaucagaua.........................................................................    3       1       seq
......ucgaagacgaucagauU.........................................................................    3       1       seq
......Acgaagacgaucagaua.........................................................................    3       1       seq
......Gcgaagacgaucagaua.........................................................................    3       1       seq
......ucgaagacUaucagaua.........................................................................    1       1       seq
......ucgaagacgaucagaCac........................................................................    1       1       seq
......ucgGagacgaucagauac........................................................................    1       1       seq
......ucgaagacgaucagauaG........................................................................    1       1       seq
......ucgaagacgGucagauac........................................................................    1       1       seq
......ucgaagacgaucagauaU........................................................................    7       1       seq
......ucgaagacgaucagauGc........................................................................    1       1       seq
......ucgaagacgaucagGuac........................................................................    1       1       seq
......ucgaagacgaucagauac........................................................................    176     0       seq
......ucgaagacgaucagauacc.......................................................................    342     0       seq
......Ncgaagacgaucagauacc.......................................................................    2       1       seq
......ucgGagacgaucagauacc.......................................................................    2       1       seq
......ucgaagacgaucagGuacc.......................................................................    1       1       seq
......ucgaGgacgaucagauacc.......................................................................    4       1       seq
......ucgaagacgaGcagauacc.......................................................................    1       1       seq
......ucgaagacgaucagauGcc.......................................................................    1       1       seq
......ucgaagacgGucagauacc.......................................................................    3       1       seq
......ucCaagacgaucagauacc.......................................................................    1       1       seq
......ucgaCgacgaucagauacc.......................................................................    1       1       seq
......ucgaagacgaucagauacG.......................................................................    1       1       seq
......ucgaagacgaucagauacA.......................................................................    10      1       seq
......Ccgaagacgaucagauacc.......................................................................    1       1       seq
......ucgaagacgaucUgauacc.......................................................................    1       1       seq
......ucgaagacgaucagaGacc.......................................................................    1       1       seq
......ucgaagacgaucGgauacc.......................................................................    5       1       seq
......ucgaagacgaucagauaccC......................................................................    3       1       seq
......ucgaagacgaucagauacca......................................................................    19      0       seq
......ucgGagacgaucagauacca......................................................................    1       1       seq
......ucgaagacgaucagauaccU......................................................................    2       1       seq
......ucgaagacgaucagauaccau.....................................................................    1       0       seq
......ucgaagacgaucagauaccaC.....................................................................    1       1       seq
.......cgaagGcgaucagauac........................................................................    1       1       seq
.......cgaagacgaucagauaA........................................................................    1       1       seq
.......cgaagacgaucCgauac........................................................................    1       1       seq
.......cgaagacgaucagauac........................................................................    194     0       seq
.......cgaagacgaCcagauac........................................................................    2       1       seq
.......cgaagacgaucagauaU........................................................................    12      1       seq
.......cgaagGcgaucagauacc.......................................................................    2       1       seq
.......Ggaagacgaucagauacc.......................................................................    1       1       seq
.......cgaUgacgaucagauacc.......................................................................    1       1       seq
.......cgaagacgaucGgauacc.......................................................................    3       1       seq
.......cgGagacgaucagauacc.......................................................................    1       1       seq
.......cgaagacgaucagauacc.......................................................................    589     0       seq
.......cgaagacgaucCgauacc.......................................................................    1       1       seq
.......cgaagacgaucagauaUc.......................................................................    1       1       seq
.......Agaagacgaucagauacc.......................................................................    1       1       seq
.......cgaagacgaucagauacA.......................................................................    22      1       seq
.......cgaGgacgaucagauacc.......................................................................    1       1       seq
.......cgaagacgGucagauacc.......................................................................    2       1       seq
.......cgaaAacgaucagauacc.......................................................................    1       1       seq
.......cgaagacgaucagGuacc.......................................................................    1       1       seq
.......Ngaagacgaucagauacc.......................................................................    1       1       seq
.......cgaagacgaucagauacca......................................................................    21      0       seq
.......Ugaagacgaucagauacca......................................................................    1       1       seq
.......cgaagacgaucagauacAa......................................................................    1       1       seq
.......cgaagacgaucagauaccU......................................................................    4       1       seq
```

Mature **Star**

gggggcucgaagacgaucagauaccauccuagucucaaccauaaaugaugucgaccagcgauugguggauguuucucuaaggacuccggcagcacc

.......cgaagacgaucagauaccC..	5	1	seq
.......cgaagacgaucagauaccUuccua...	2	1	seq
........gaagacgaucGgauacc...	1	1	seq
........gaagacgaCcagauacc...	1	1	seq
........Uaagacgaucagauacc...	1	1	seq
........gaagGcgaucagauacc...	1	1	seq
........gaagacgaucagaCacc...	1	1	seq
........gaagacgaucagauaUc...	1	1	seq
........gaagacgaucagauacA...	2	1	seq
........gaagacgaucagauacc...	102	0	seq
........gaagacUaucagauacc...	1	1	seq
........gaagacgaucagauaccC..	1	1	seq
........gaagacgaucagauacca..	3	0	seq
........gaagacgaucagauaccU..	1	1	seq
........gaagacgaucagauaccaGc..	1	1	seq
........gaagacgaucagauaccauccuagucuc..	1	0	seq
.........aagacgaucagauaccU..	3	1	seq
.........aagacgaucagauacca..	6	0	seq
.........aagacUaucagauacca..	1	1	seq
.........aagacgaucagauaccUuc..	1	1	seq
..........agacgaucagauaccauA..	1	1	seq
..........agacgaucagauaccauccuagucucaacca...	1	0	seq
...........gacgaucagauaccauccuagucucaacc..	1	0	seq
.............cgaucagauaccauccuagucuA..	1	1	seq
..............gaucagauaccauccuagucu...	1	0	seq
...............aucagauaccauccuagucucaacca...	1	0	seq
.................cagauaccauccuagucucaaccaua...	1	0	seq
..................agauaccauccuagucucaaccaua...	1	0	seq
...................gauaccauccuagucucaac...	1	0	seq
...................gauaccauccuagucucaacc..	1	0	seq
...................gauaccauccuagucucaaccaA..	1	1	seq
...................gauaccauccuagucucaaccaC..	1	1	seq
...................gauaccauccuagucucaaccaua...	8	0	seq
...................gauaccGuccuagucucaaccauaaaugau...	1	1	seq
...................gauaccGuccuagucucaaccauaaaugaug..	1	1	seq
....................auaccauccuagucuca...	1	0	seq
....................auaccauccuagucucaac...	2	0	seq
....................auaccauccuagucucaacc..	3	0	seq
....................auaccauccuagucucaacca...	3	0	seq
....................auaccauccuagucucaaccaua...	7	0	seq
....................auaccauccuagucucaaccauaaa...	1	0	seq
.....................uaccauccuagucucaac...	1	0	seq
.....................uaccCuccuagucucaacc..	1	1	seq
.....................uaccauccuagucucaacc..	6	0	seq
.....................uaccauccuagucucaacU..	1	1	seq
.....................uaccauccuagucucaacA..	1	1	seq
.....................uaccauccuagucucaacca...	12	0	seq
.....................uaccauccuagucucaaccU...	1	1	seq
.....................uaccCuccuagucucaacca...	1	1	seq
.....................uaccauccuagucucaaccau..	1	0	seq
.....................uaccauccuGgucucaaccau..	1	1	seq
.....................uaccCuccuagucucaaccaua...	1	1	seq
.....................uaccauccuagucucaaccaua...	26	0	seq
.....................uaccauccuagucucaaccauaG..	1	1	seq
.....................uaccCuccuagucucaaccauaa..	1	1	seq
.....................uaccauccuagucucaaccauaaa...	3	0	seq
.....................uaccGuccuagucucaaccauaaauga..	1	1	seq
......................accauccuagucucaacc..	1	0	seq
......................accauccuagucucaacca...	3	0	seq
......................Gccauccuagucucaaccaua...	1	1	seq
......................accauccuagucucaaccaua...	7	0	seq
......................accauccuagucucaaccauaa..	1	0	seq
......................accCuccuagucucaaccauaa..	1	1	seq
.......................ccCuccuagucucaacc..	4	1	seq
.......................ccauccuagucucaacc..	3	0	seq
.......................ccauccuagucucaacca...	3	0	seq
.......................ccauccuagucucGaccaua...	1	1	seq
.......................ccCuccuagucucaaccaua...	2	1	seq
.......................ccauccuagucucaaccaua...	11	0	seq
........................cauccuagucucaacca...	2	0	seq

Mature **Star**

ggggcucgaagacgaucagauaccauccuagucucaaccauaaaugaugucgaccagcgauugguggauguuucucuaaggacuccggcagcacc			
.......................cCuccuagucucaaccaua...	2	1	seq
.......................cauccuagucucaaccaua...	2	0	seq
........................Nuccuagucucaaccaua...	1	1	seq
........................Nuccuagucucaaccauaa..	1	1	seq
.........................uccuagucucCaccaua...	1	1	seq
.........................uccuagucucaUccaua...	1	1	seq
.........................ucUuagucucaaccaua...	2	1	seq
.........................uGcuagucucaaccaua...	1	1	seq
.........................uccuaguUucaaccaua...	1	1	seq
.........................uccuagCcucaaccaua...	7	1	seq
.........................uccuagucucaaccaua...	2509	0	seq
.........................uccuagAcucaaccaua...	1	1	seq
.........................Gccuagucucaaccaua...	4	1	seq
.........................uccuagucucaaccUua...	1	1	seq
.........................uccuagucucaaccGua...	5	1	seq
.........................Nccuagucucaaccaua...	3	1	seq
.........................uccuagucucaaccaGa...	6	1	seq
.........................uccuagucucaaccaCa...	18	1	seq
.........................uccCagucucaaccaua...	6	1	seq
.........................uccuGgucucaaccaua...	2	1	seq
.........................uccuagucucaaccCua...	1	1	seq
.........................uUcuagucucaaccaua...	3	1	seq
.........................uccuagucucUaccaua...	2	1	seq
.........................uccuagucucaaUcaua...	2	1	seq
.........................uccuagucCcaaccaua...	6	1	seq
.........................uccuagucucaacGaua...	1	1	seq
.........................uccuUgucucaaccaua...	1	1	seq
.........................uccuagucucaaccaAa...	2	1	seq
.........................uccuagucAcaaccaua...	3	1	seq
.........................uccuagucucGaccaua...	5	1	seq
.........................uccuaAucucaaccaua...	1	1	seq
.........................uccuagCcucaaccauaa..	2	1	seq
.........................uccuagucucaaGcauaa..	1	1	seq
.........................uccuagucucaaccauaG..	2	1	seq
.........................uccuagucucaaccauaa..	161	0	seq
.........................uccCagucucaaccauaa..	1	1	seq
.........................uccuGgucucaaccauaaa...	1	1	seq
.........................uccuagucucaaccauaaU...	1	1	seq
.........................uccuagucucaaccauaaG...	1	1	seq
.........................uccuagucucaaUcauaaa...	1	1	seq
.........................uccuagucucaaGcauaaa...	1	1	seq
.........................uccuagucucaaccauaaa...	76	0	seq
.........................uccuagucucaaccauaaau..	3	0	seq
.........................uccuagucucaaccauaaaA..	1	1	seq
.........................uccuagucucaaccauaaaug..	3	0	seq
.........................uccuagucucaaccauaaaugaug...	1	0	seq
..........................ccuagucucaaccauaa..	2	0	seq
..........................ccuaguGucaaccauaaa...	1	1	seq
...........................cuagucucaaccauaaa...	25	0	seq
...........................cuagucucaaccauaaaA..	1	1	seq
...........................cuagucucaaccauaaau..	1	0	seq
...........................cuagucucaaccauaaaugaugCcgacca.......................................	1	1	seq
............................uagucucaaccauaaau..	1	0	seq
............................uagucucaaccauaaGu..	1	1	seq
.............................agucucaaccauaaaug...	1	0	seq
.............................agucucaaccauaaaugaug..	1	0	seq
....................................accauaaaCgaugucgaccagcg....................................	1	1	seq
....gguggauguuGcucuaaggacu...........	2	1	seq
....gguggauguuGcucuaaggacuc..........	1	1	seq
....gguggauguuGcucuaaggacucc.........	4	1	seq
....guggauguuGcucuaaggacu...........	1	1	seq
....guggauguuGcucuaaggacucc.........	2	1	seq
....uggauguuGcucuaaggacuc..........	1	1	seq
....uggauguuGcucuaaggacucc.........	1	1	seq
....ggauguuAcucuaaggacu...........	1	1	seq
....ggauguuCcucuaaggacu...........	1	1	seq
....ggauguuAcucuaaggacuccg........	2	1	seq
....ggauguuGcucuaaggacuccggc......	1	1	seq
....gauguuAcucuaaggacu...........	1	1	seq
....gauguuAcucuaaggacucc.........	1	1	seq

Mature **Star**

```
ggggcucgaagacgaucagauaccauccuagucucaaccauaaaaugaugucgaccagcgauugguggauguuucucuaaggacuccggcagcacc
...................................................................gauguuAcucuaaggacuccg........	1	1	seq
...................................................................gauguuGcucuaaggacuccgg.......	1	1	seq
...................................................................gauguuGcucuaaggacuccggca.....	2	1	seq
...................................................................gauguuGcucuaaggacuccggcagca..	1	1	seq
....................................................................auguuucucuaaggacu...........	3	0	seq
....................................................................auguuAcucuaaggacu...........	1	1	seq
....................................................................auguuucucuaaggacuccg........	2	0	seq
.....................................................................uguuAcucuaaggacucc.........	2	1	seq
.....................................................................uguuCcucuaaggacucc.........	1	1	seq
.....................................................................uguuAcucuaaggacuccg........	4	1	seq
.....................................................................uguuucucuaaggacuccg........	2	0	seq
.....................................................................uguuGcucuaaggacuccgg.......	4	1	seq
.....................................................................uguuGcucuaaggacuccggc......	3	1	seq
.....................................................................uguuGcucuaaggacuccggca.....	2	1	seq
.....................................................................uguuGcucuaaggacuccggcagcac.	1	1	seq
......................................................................guuucucuaaggacuccg........	1	0	seq
......................................................................guuGcucuaaggacuccgg.......	3	1	seq
......................................................................guuGcucuaaggacuccggc......	3	1	seq
......................................................................guuGcucuaaggacuccggca.....	2	1	seq
.......................................................................uuAcucuaaggacuccg........	4	1	seq
.......................................................................uuucucuaaggacuccC........	1	1	seq
.......................................................................uuCcucuaaggacuccg........	1	1	seq
.......................................................................uuucucuaaggacuccg........	3	0	seq
.......................................................................uuGcucuaaggacuccgg.......	8	1	seq
.......................................................................uuGcucuaaggacuccggc......	2	1	seq
.......................................................................uuGcucuaaggacuccggca.....	2	1	seq
.......................................................................uuGcucuaaggacuccggcag....	1	1	seq
.......................................................................uuGcucuaaggacuccggcagc...	1	1	seq
.......................................................................uuGcucuaaggacuccggcagca..	2	1	seq
.......................................................................uuGcucuaaggacuccggcagcac.	1	1	seq
........................................................................uGcucuaaggacuccgg.......	14	1	seq
........................................................................uGcucuaaggacuccggc......	3	1	seq
........................................................................uGcucuaaggacuccggca.....	5	1	seq
.........................................................................Gcucuaaggacuccggc......	10	1	seq
.........................................................................Gcucuaaggacuccggca.....	3	1	seq
.........................................................................Gcucuaaggacuccggcagc...	1	1	seq
..........................................................................cucuaaggacuccgUca.....	5	1	seq
..........................................................................cCcuaaggacuccggca.....	1	1	seq
..........................................................................cucuaaggacuccgAca.....	6	1	seq
..........................................................................cucuaaggacuccggca.....	3	0	seq
..........................................................................cucuaaggacuccgAcag....	1	1	seq
..........................................................................cucuaaggacuccgUcag....	14	1	seq
..........................................................................cucuaaggacuccggcag....	2	0	seq
..........................................................................cucuaaggacuccggcagc...	1	0	seq
..........................................................................cucuaaggacuccggcagca..	1	0	seq
..........................................................................cucuaaggacuccgAcagca..	1	1	seq
..........................................................................cucuaaggacuccgUcagca..	1	1	seq
..........................................................................cucuaaggacuccgUcagcacc	1	1	seq
..........................................................................cucuaaggacuccggcagcacc	2	0	seq
..........................................................................cucuaaggacuccgAcagcacc	2	1	seq
...........................................................................ucuaaggacuccgUcag....	1	1	seq
...........................................................................ucuaaggacuccgAcagca..	1	1	seq
...........................................................................ucuaaggacuccgUcagcacc	3	1	seq
............................................................................cuaaggacuccgUcagc...	3	1	seq
............................................................................cuaaggacuccgUcagca..	1	1	seq
............................................................................cuaaggacuccgAcagca..	2	1	seq
............................................................................cuaaggacuccggcagca..	1	0	seq
............................................................................cuaaggacuccggcagcaU.	1	1	seq
............................................................................cuaaggacuccgAcagcacc	1	1	seq
............................................................................cuaaggacuccggcagcacc	1	0	seq
............................................................................cuaaggacuccgUcagcacc	4	1	seq
.............................................................................uaaggacuccgUcagca..	2	1	seq
.............................................................................uaaggacuccggcagcacc	1	0	seq
.............................................................................uaaggacuccgUcagcacc	1	1	seq
..............................................................................aaggacuccgAcagcacc	1	1	seq
..............................................................................aaggacuccgUcagcacc	4	1	seq
..............................................................................aaggacuccggcaCcacc	1	1	seq
...............................................................................aggacuccgUcagcacc	7	1	seq
...............................................................................aggacuccgAcagcacc	1	1	seq
...............................................................................aggacuccggcagcacc	2	0	seq
```

Mature Star

ggggcucgaagacgaucagauaccauccuagucucaaccauaaaugaugucgaccagcgauugguggauguuucucuaaggacuccggcagcacc

Provisional ID	:	MA_131684_21667
Score total	:	1.9
Score for star read(s)	:	-1.3
Score for read counts	:	-0.3
Score for mfe	:	2
Score for randfold	:	1.6
Score for cons. seed	:	
Total read count	:	11
Mature read count	:	11
Loop read count	:	0
Star read count	:	0

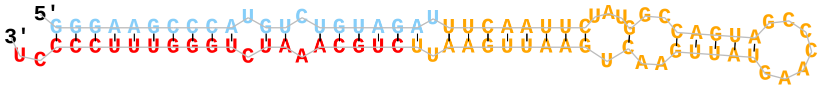

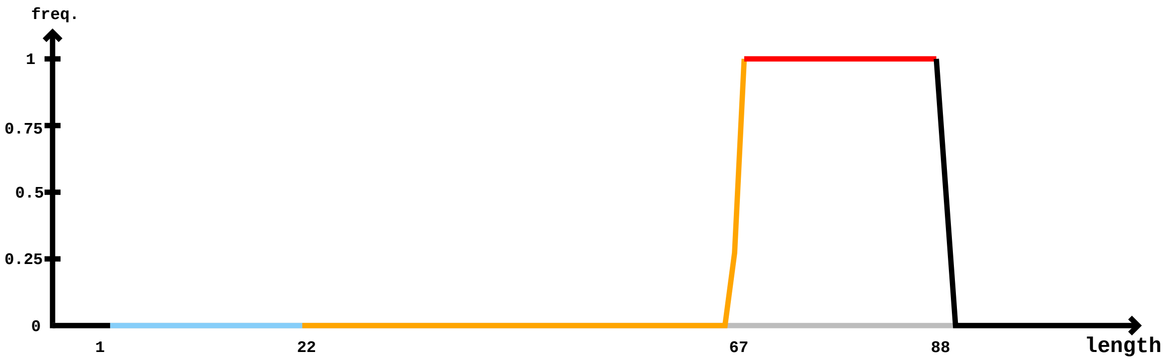

Star Mature

```
5'- cugggggaagcccaugucuguagauuucaauucuauggccaguagcccaaguauugaacugaauugaauucugcaaaucuggguuuccccuugagagucguaaaguuuugu   -3'    exp
    (.(((((((((((.((.((((((.((((((((...(..(((((.......)))))..).)))))))).)))))).)).)))))))))).).....................   reads    mm    sample
    .....................................................................ucugcaaaucuggguuuccccu....................   3        0     seq
    ......................................................................cugcaaaucuggguuuccccC....................   2        1     seq
    ......................................................................cugcaaaucuggguuuccccu....................   5        0     seq
    ......................................................................cugcaaaucuggguuuccccuu...................   1        0     seq
```

Provisional ID	:	MA_13321_462
Score total	:	1.2
Score for star read(s)	:	-1.3
Score for read counts	:	0
Score for mfe	:	0.9
Score for randfold	:	1.6
Score for cons. seed	:	
Total read count	:	36
Mature read count	:	36
Loop read count	:	0
Star read count	:	0

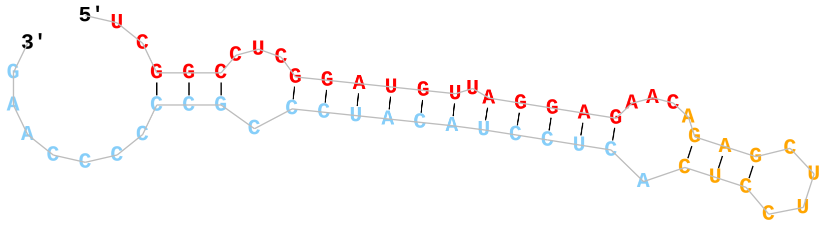

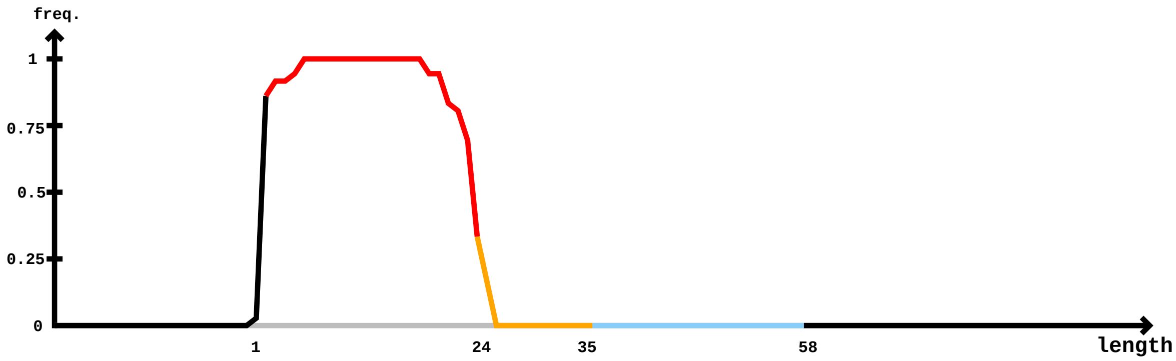

Mature Star

```
5'- cuauucagagcuucuuuucuucggccucggauguuaggagaacagagcuuccucacuccuacaucccgcccccaaggucauacaucugaaucccccucugguacucgagga  -3'   exp
    ..((((((........((((.(((...((((((.(((((....(((....))).))))))))))).)))....)))).......)))))))...((((........)))).  reads    mm    sample
    ...................uucggccucggauguuaggaga......................................................................  1        0     seq
    ....................ucggccuAggauguuag..........................................................................  1        1     seq
    ....................ucggccucggauguuag..........................................................................  1        0     seq
    ....................ucggccucggaugGuagga........................................................................  1        1     seq
    ....................ucggccucggauguuagga........................................................................  1        0     seq
    ....................ucggccucgUauguuagga........................................................................  1        1     seq
    ....................ucggccucggauguuaggag.......................................................................  1        0     seq
    ....................ucggccucggauguuaggaga......................................................................  2        0     seq
    ....................ucggccucgCauguuaggagaa.....................................................................  1        1     seq
    ....................ucggccucggauguuaggagaa.....................................................................  9        0     seq
    ....................ucggccucgUauguuaggagaa.....................................................................  1        1     seq
    ....................ucggccucgAauguuaggagaac....................................................................  11       1     seq
    .....................Uggccucggauguuagga........................................................................  1        1     seq
    .....................cggccucgAauguuaggagaac....................................................................  1        1     seq
    .......................gccucggaugGuaggaga......................................................................  1        1     seq
    ........................ccucggauguuaggagUa.....................................................................  1        1     seq
    ........................ccucggauguuaggagaG.....................................................................  1        1     seq
```

Provisional ID	:	MA_1336053_42400
Score total	:	9188.6
Score for star read(s)	:	3.9
Score for read counts	:	9186.2
Score for mfe	:	0.7
Score for randfold	:	-2.2
Score for cons. seed	:	
Total read count	:	18030
Mature read count	:	17996
Loop read count	:	7
Star read count	:	27

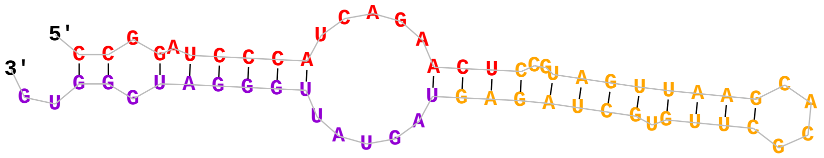

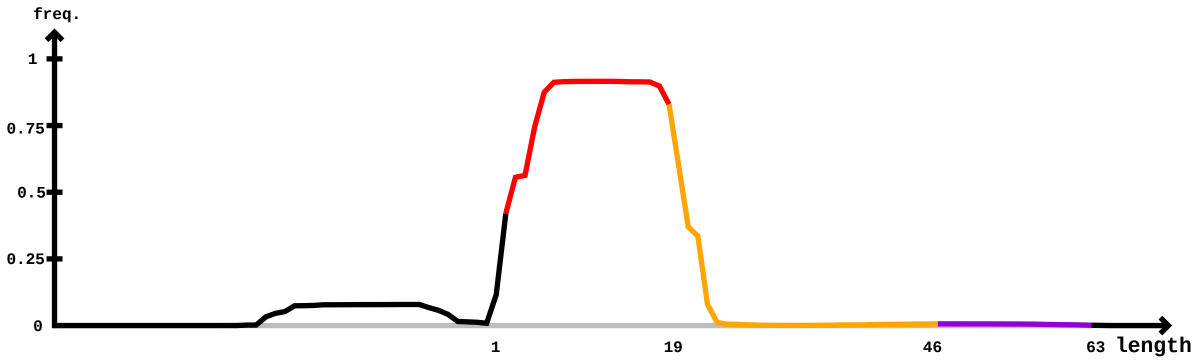

Mature Star

```
5'- agggggcgugauucuugcgugugcgaucauaccagcguuaauguaccggaucccaucagaacuccguaguuaagcacgcuugugcuagaguaguauugggauggguguaucuccc  -3'   obs
    agggggcgugauucuugcgugugcgaucauaccagcguuaauguaccggaucccaucagaacuccguaguuaagcacgcuugugcuagaguaguauugggauggguguaucuccc        exp
    .(((((..((((.((.(.(((((....)))))))).))))...((((.(.(((((.....((((..((((((((....)))).)))))))).....)))))).))))..)))))  reads   mm   sample
    ................gAgugugcgaucauaccagcguu...........................................................................  1       1    seq
    .................cgugugcgaucauaccag...............................................................................  1       0    seq
    ..................gugugcgaucauaccag...............................................................................  1       0    seq
    ..................gugugcgaucauaccagc..............................................................................  1       0    seq
    ..................gugugcgaucauaccagcg.............................................................................  5       0    seq
    ..................gugugcgaucauaccagcgu............................................................................  1       0    seq
    ..................gugugcgaucauaccagcgC............................................................................  1       1    seq
    ..................gugugcgaucauaccagcguu...........................................................................  2       0    seq
    ..................gugugcgaucauaccagcguua..........................................................................  3       0    seq
    ..................gugugcgaucauaccagcguuaaug.......................................................................  1       0    seq
    ..................gGgugcgaucauaccagcguuaaugu......................................................................  3       1    seq
    ..................gGgugcgaucauaccagcguuaauguaccggau...............................................................  1       1    seq
    ..................gGgugcgaucauaccagcguuaauguaccggaucc.............................................................  2       1    seq
    ..................gugugcgaucauaccagcguuaaugCaccggaucccauc.........................................................  2       1    seq
    ..................gGgugcgaucauaccagcguuaauguaccggaucccauc.........................................................  5       1    seq
    ..................gGgugcgaucauaccagcguuaauguaccggaucccaucagaacu...................................................  1       1    seq
    ..................gugugcgaucauaccagcguuaaugCaccggaucccaucagaacuc..................................................  1       1    seq
    ..................gGgugcgaucauaccagcguuaauguaccggaucccaucagaacuc..................................................  1       1    seq
    ...................Ngugcgaucauaccagcg.............................................................................  1       1    seq
    ...................ugugcgaucauaccagcguu...........................................................................  2       0    seq
    ...................ugugcgaucauaccagcguuaaugCaccggaucccauc.........................................................  1       1    seq
    ....................gugcgGucauaccagcg.............................................................................  2       1    seq
    ....................gugcgaucauaccagcg.............................................................................  227     0    seq
    ....................Nugcgaucauaccagcg.............................................................................  1       1    seq
    ....................guUcgaucauaccagcg.............................................................................  1       1    seq
    ....................gugcgaucauaccagGg.............................................................................  1       1    seq
    ....................gugcgaucauaccagcgu............................................................................  125     0    seq
    ....................Nugcgaucauaccagcgu............................................................................  1       1    seq
    ....................gugcgaucauaccagcgG............................................................................  4       1    seq
    ....................gugcgGucauaccagcguu...........................................................................  2       1    seq
    ....................gugcgaucauaccagcguu...........................................................................  131     0    seq
    ....................gugcgaucauaccUgcguu...........................................................................  1       1    seq
    ....................gugcgaucauaccagcguG...........................................................................  4       1    seq
```

```
                                          Mature                                    Star
agggggcgugauucuugcgugugcgaucauaccagcguuaauguaccggaucccaucagaacuccguaguuaagcacgcuugugcuagaguaguauugggaugggugaucuccc
....................gGgcgaucauaccagcguu...........................................................................    1      1      seq
....................gCgcgaucauaccagcguua..........................................................................    1      1      seq
....................gugcgaucCuaccagcguua..........................................................................    1      1      seq
....................gugcgaucauaccagcguuU..........................................................................    1      1      seq
....................gugcgaucauaccagcguua..........................................................................    80     0      seq
....................Nugcgaucauaccagcguuaa.........................................................................    1      1      seq
....................gugcgaucauaccagcguuaa.........................................................................    8      0      seq
....................gugcgaucauaccagcguuaau........................................................................    6      0      seq
....................gugcgaucauaccagcguuaaug.......................................................................    8      0      seq
....................gugcgaucauaccagcguuaaugu......................................................................    4      0      seq
.....................ugcgaucauaccagcgG............................................................................    1      1      seq
.....................ugcgaucauaccagcgu............................................................................    78     0      seq
.....................ugcgaucauaccagGguu...........................................................................    1      1      seq
.....................Cgcgaucauaccagcguu...........................................................................    2      1      seq
.....................ugcgGucauaccagcguu...........................................................................    1      1      seq
.....................ugcgaucauaccagcguu...........................................................................    101    0      seq
.....................Agcgaucauaccagcguu...........................................................................    1      1      seq
.....................ugcgaucauaccagcguG...........................................................................    5      1      seq
.....................ugcgaucauaccagcguua..........................................................................    38     0      seq
.....................ugcgGucauaccagcguua..........................................................................    1      1      seq
.....................ugcgaucauaccagcguuaa.........................................................................    5      0      seq
.....................ugcgaucauaccagcguuaau........................................................................    5      0      seq
.....................ugcgaucauaccagcguuaaug.......................................................................    21     0      seq
.....................ugcgaucauaccagcguuaauguacc...................................................................    1      0      seq
......................gcgaucauaccagcgGu...........................................................................    1      1      seq
......................gcgaucauaccagcguu...........................................................................    54     0      seq
......................gcgaucauaccagcguua..........................................................................    63     0      seq
......................gcgGucauaccagcguua..........................................................................    1      1      seq
......................Ucgaucauaccagcguuaa.........................................................................    1      1      seq
......................gcgaucauaccagcguuaa.........................................................................    7      0      seq
......................gcgaucauaccagcguuaau........................................................................    2      0      seq
......................gcgaucauaccagcguuaaug.......................................................................    3      0      seq
......................gcUaucauaccagcguuaaug.......................................................................    1      1      seq
......................gcgaucauaccagcguuaaugCaccggaucccaucagaacuccgu...............................................    1      1      seq
.......................cgaucauaccagcguuC..........................................................................    2      1      seq
.......................cgGucauaccagcguua..........................................................................    2      1      seq
.......................cgaucauaccagcguua..........................................................................    337    0      seq
.......................cgUucauaccagcguua..........................................................................    1      1      seq
.......................cgaucauaccagcguuU..........................................................................    5      1      seq
.......................cgaucauaccUgcguua..........................................................................    2      1      seq
.......................cgaucauaccagcguuaa.........................................................................    35     0      seq
.......................cgaucauaccagcguuaau........................................................................    23     0      seq
.......................cgaucauaccagcguuaaug.......................................................................    21     0      seq
.......................cgaucauaccagcguuaaugu......................................................................    2      0      seq
.......................cgaucauaccagcguuaauguaccgga................................................................    1      0      seq
.......................cgaucauaccagcguuaaugCaccggaucccaucagaacuccgu...............................................    1      1      seq
........................gaucauaccagcguuaU.........................................................................    1      1      seq
........................gaucauaccagcguuaa.........................................................................    2      0      seq
........................gaucauaccagcguuaau........................................................................    1      0      seq
........................gaucauaccagcguuaaug.......................................................................    2      0      seq
.........................aucauaccagcguuaau........................................................................    9      0      seq
.........................aucauaccagcguuaaug.......................................................................    9      0      seq
.........................aucauaccagcguuaauguaccggaucccauc.........................................................    1      0      seq
..........................ucauaccagcguuaaug.......................................................................    42     0      seq
..........................ucauaccagcguuaaugu......................................................................    1      0      seq
..........................ucGuaccagcguuaauguaccggauccc............................................................    1      1      seq
..........................Acauaccagcguuaauguaccggauccc............................................................    1      1      seq
..........................ucauaccagcguuaauguaccggaucccau..........................................................    1      0      seq
..........................ucauaccagcguuaauguaccggaucccaucagaac....................................................    1      0      seq
...........................cauaccagcguuaaugu......................................................................    1      0      seq
...........................cauaccagcguuaauguaccgg.................................................................    1      0      seq
............................auaccagcguuaauguaccggaucccauc.........................................................    1      0      seq
............................auaccagcguuaaugAaccggaucccauca........................................................    1      1      seq
.............................uaccagcguuaauguaccggaucccauc.........................................................    1      0      seq
.............................uaccagcguuaauguaccggaucccauca........................................................    2      0      seq
.............................uaccagcguuaauguaccggaucccaucagaa.....................................................    1      0      seq
.............................uaccagcguuaauguaccggaucccaucagaacu...................................................    2      0      seq
..............................accagcguuaauguacc...................................................................    1      0      seq
..............................accagcguuaauguaccggaucccaucagaacuc..................................................    1      0      seq
..............................accagcguuaauguaccggaucccaucagaacuccgCagu............................................    1      1      seq
```

Mature Star

```
agggggcgugauucuugcgugugcgaucauaccagcguuaauguaccggaucccaucagaacuccguaguuaagcacgcuugugcuagaguaguauugggauggguguaucuccc
................................cagcguuaauguaccggaucccau..........................................................   1   0   seq
................................cagcguuaauguaccggaucccaucagaacu...................................................   1   0   seq
.................................agcguuaauguaccgga................................................................   1   0   seq
.................................agcguuaauguaccggaucccaucagaacu...................................................   1   0   seq
.................................agcguuaauguaccggaucccaucagaacC...................................................   1   1   seq
..................................gcguuaauguaccggaucc.............................................................   1   0   seq
..................................gcguuaauguaccggauccU............................................................   1   1   seq
..................................gcguuaauguaccggaucccaucag.......................................................   1   0   seq
..................................gcguuaauguaccggaucccaucagaacu...................................................   1   0   seq
...................................cguuaaugAaccggaucccau..........................................................   1   1   seq
...................................cguuaauguaccggaucccaucagaacuccgCagu............................................   1   1   seq
...................................cguuaauguaccggaucccaucagaacuccgCaguu...........................................   1   1   seq
....................................guuaauguaccggaucccauc.........................................................   1   0   seq
.....................................uuaauguaccggaucccauca........................................................   1   0   seq
.....................................uuaauguaccggaucccaucaga......................................................   2   0   seq
.....................................uuaauguaccggaucccaucagaacu...................................................   1   0   seq
......................................uaauguaccggauccca...........................................................   2   0   seq
......................................uaauguaccggaucccauc.........................................................   1   0   seq
......................................uaauguaccggaucccauca........................................................   1   0   seq
......................................uaauguaccggaucccaucagaa.....................................................   1   0   seq
......................................uaauguaccggaucccaucagaacu...................................................   1   0   seq
......................................uaauguaccggaucccaucagaacuccgCaguua..........................................   1   1   seq
.......................................aauguaccggaucccauca........................................................   1   0   seq
.......................................aauguaccggaucccaucagaac....................................................   1   0   seq
.......................................aauguaccggaucccaucagaacC...................................................   1   1   seq
.......................................aauguaccggaucccaucagaacu...................................................   2   0   seq
........................................auguaccggaucccauc.........................................................   1   0   seq
........................................auguaccggaucccauca........................................................   4   0   seq
........................................auguaccggaucccaucag.......................................................   2   0   seq
........................................auguaccggaucccaucaga......................................................   2   0   seq
........................................auguaccggaucccaucagaacu...................................................   2   0   seq
........................................augAaccggaucccaucagaacu...................................................   1   1   seq
........................................auguaccggaucccaucagaacuc..................................................   1   0   seq
........................................auguaccggaucccaucagaacuccgC...............................................   1   1   seq
........................................auguaccggaucccaucagaacuccgCaguu...........................................   1   1   seq
.........................................uguaccggaucccauca........................................................   7   0   seq
.........................................uguaccggaucccaucag.......................................................   2   0   seq
.........................................uguaccggaucccaucaga......................................................   1   0   seq
.........................................ugAaccggaucccaucaga......................................................   1   1   seq
.........................................uguaccggaucccaucagaa.....................................................   1   0   seq
.........................................uguaccggaucccaucagaacu...................................................   5   0   seq
.........................................uguaccggaucccaucagaacC...................................................   2   1   seq
.........................................ugAaccggaucccaucagaacuc..................................................   1   1   seq
.........................................uguaccggaucccaucagaacuc..................................................   2   0   seq
.........................................uguaccggaucccaucagaacucc.................................................   1   0   seq
.........................................uguaccggaucccaucagaacuccgu...............................................   1   0   seq
.........................................uguaccggaucccaucagaacuccgCa..............................................   4   1   seq
.........................................uguaccggaucccaucagaacuccgCagu............................................   3   1   seq
.........................................uguaccggaucccaucagaacuccgCaguu...........................................   1   1   seq
..........................................guaccggaucccaucagaa.....................................................   1   0   seq
..........................................guaccggaucccaucagaac....................................................   2   0   seq
..........................................guaccggaucccaucagaacC...................................................   2   1   seq
..........................................Cuaccggaucccaucagaacu...................................................   1   1   seq
..........................................guaccggaucccaucagaacu...................................................   4   0   seq
..........................................guaccggaucccaucagaacuc..................................................   3   0   seq
..........................................guaccggaucccaucagaacuccgCaguua..........................................   1   1   seq
...........................................uaccggaucccaucaga......................................................   3   0   seq
...........................................Naccggaucccaucaga......................................................   2   1   seq
...........................................Aaccggaucccaucaga......................................................   2   1   seq
...........................................Naccggaucccaucagaa.....................................................   2   1   seq
...........................................uaccggaucccaucagaa.....................................................   3   0   seq
...........................................uaccggaucccaucagaac....................................................   5   0   seq
...........................................uaccggaucccaucagaacu...................................................   4   0   seq
...........................................Naccggaucccaucagaacu...................................................   1   1   seq
...........................................uaccggaucccaucagaGcu...................................................   1   1   seq
...........................................Aaccggaucccaucagaacu...................................................   2   1   seq
...........................................uaccggaucccaucagaacG...................................................   1   1   seq
...........................................Naccggaucccaucagaacuc..................................................   4   1   seq
...........................................uaccggaucccaucagaacuc..................................................   4   0   seq
...........................................uaccggaucccaucagaacuccgCa..............................................   1   1   seq
```

```
                                             Mature                                     Star
agggggcgugauucuugcgugugcgaucauaccagcguuaauguaccggaucccaucagaacuccguaguuaagcacgcuugugcuagaguaguauugggaugggugaucuccc
...........................................uaccggaucccaucagaacuccgCagu............................................  1     1  seq
...........................................uaccggaucccaucagaacuccgCaguuaag........................................  1     1  seq
............................................accggaucccUucagaa.....................................................  1     1  seq
............................................accggaucccaucagUa.....................................................  1     1  seq
............................................Cccggaucccaucagaa.....................................................  2     1  seq
............................................accggGucccaucagaa.....................................................  3     1  seq
............................................accggaucccaucagaU.....................................................  1     1  seq
............................................accggaCcccaucagaa.....................................................  1     1  seq
............................................accggaucccaucagaa.....................................................  285   0  seq
............................................accggaucccaucagaaA....................................................  1     1  seq
............................................accggaucccaucagaac....................................................  385   0  seq
............................................accggaCcccaucagaac....................................................  1     1  seq
............................................accggGucccaucagaacu...................................................  2     1  seq
............................................Cccggaucccaucagaacu...................................................  1     1  seq
............................................accggCucccaucagaacu...................................................  1     1  seq
............................................accggaCcccaucagaacu...................................................  1     1  seq
............................................accggaucccaucagaacC...................................................  126   1  seq
............................................accggaucccaucagaacu...................................................  733   0  seq
............................................Uccggaucccaucagaacuc..................................................  1     1  seq
............................................Cccggaucccaucagaacuc..................................................  2     1  seq
............................................accggaucccaucagaaAuc..................................................  1     1  seq
............................................accggUucccaucagaacuc..................................................  1     1  seq
............................................accggaucccUucagaacuc..................................................  1     1  seq
............................................accggaCcccaucagaacuc..................................................  1     1  seq
............................................accggaucAcaucagaacuc..................................................  2     1  seq
............................................accggaucccaucagaacuc..................................................  528   0  seq
............................................accggaucccaucagaacCc..................................................  7     1  seq
............................................Nccggaucccaucagaacuc..................................................  1     1  seq
............................................accggaucccaucagaacucc.................................................  28    0  seq
............................................accggaucccaucagaacuccg................................................  9     0  seq
............................................accggaucccaucagaacCccg................................................  1     1  seq
............................................accggauAccaucagaacuccgu...............................................  1     1  seq
............................................accggaucccaucagaacuccgA...............................................  1     1  seq
............................................accggaucccaucagaacuccgu...............................................  3     0  seq
............................................accggaucccaucagaGcuccgua..............................................  1     1  seq
.............................................ccggaucccaAcagaac....................................................  1     1  seq
.............................................ccggaucccaucagaaA....................................................  4     1  seq
.............................................ccggaucAcaucagaac....................................................  1     1  seq
.............................................ccggauccGaucagaac....................................................  1     1  seq
.............................................ccggGucccaucagaac....................................................  5     1  seq
.............................................ccggaucccaGcagaac....................................................  1     1  seq
.............................................ccggaCcccaucagaac....................................................  1     1  seq
.............................................ccggaucccaucagaac....................................................  958   0  seq
.............................................ccggaucccaucagaacu...................................................  2743  0  seq
.............................................ccggGucccaucagaacu...................................................  6     1  seq
.............................................ccggaucccaGcagaacu...................................................  2     1  seq
.............................................ccggaucccaucCgaacu...................................................  1     1  seq
.............................................ccggCucccaucagaacu...................................................  1     1  seq
.............................................ccggauGccaucagaacu...................................................  1     1  seq
.............................................ccggaucccaucagaacC...................................................  442   1  seq
.............................................ccggaucccUucagaacu...................................................  1     1  seq
.............................................ccggaucccaucagUacu...................................................  1     1  seq
.............................................Ncggaucccaucagaacu...................................................  4     1  seq
.............................................ccUgaucccaucagaacu...................................................  2     1  seq
.............................................ccggaCcccaucagaacu...................................................  5     1  seq
.............................................ccggauccGaucagaacu...................................................  2     1  seq
.............................................ccCgaucccaucagaacu...................................................  1     1  seq
.............................................ccggUucccaucagaacuc..................................................  1     1  seq
.............................................ccggaucccaucagaacuc..................................................  1678  0  seq
.............................................Ncggaucccaucagaacuc..................................................  2     1  seq
.............................................ccggaucccaucagaUcuc..................................................  1     1  seq
.............................................ccggaucccaucCgaacuc..................................................  1     1  seq
.............................................ccggaGcccaucagaacuc..................................................  1     1  seq
.............................................ccggaucccaucagaacuG..................................................  1     1  seq
.............................................ccggaucccaucagUacuc..................................................  2     1  seq
.............................................ccggaCcccaucagaacuc..................................................  5     1  seq
.............................................ccggGucccaucagaacuc..................................................  7     1  seq
.............................................ccggauccGaucagaacuc..................................................  1     1  seq
.............................................ccggaucccaucagaacCc..................................................  17    1  seq
.............................................ccggaucGcaucagaacucc.................................................  1     1  seq
```

```
                                              Mature                                      Star
agggggcgugauucuugcgugugcgaucauaccagcguuaaugua ccggaucccaucagaacu ccguaguuaagcacgcuugugcuagag uaguauugggaugggug aucuccc
.............................................ccggaucccaucagaacCcc.................................................   1      1   seq
.............................................ccggaucccaucagaacucc.................................................   59     0   seq
.............................................ccggaucccaucagaacuccU................................................   2      1   seq
.............................................ccggaucccaucagaacuccg................................................   32     0   seq
.............................................ccggaucccaucagaacuccgG...............................................   1      1   seq
.............................................ccUgaucccaucagaacuccgu...............................................   1      1   seq
.............................................ccggaucccaucagaacuccCu...............................................   1      1   seq
.............................................ccggaucccaucagaacuccgA...............................................   3      1   seq
.............................................ccggaucccaucagaacuccgu...............................................   49     0   seq
.............................................ccggGucccaucagaacuccgu...............................................   1      1   seq
.............................................ccggaucccaucagaacuccgua..............................................   4      0   seq
..............................................cggaucccaucagaacC...................................................   224    1   seq
..............................................cggaCcccaucagaacu...................................................   4      1   seq
..............................................cggaucccCucagaacu...................................................   1      1   seq
..............................................cggGucccaucagaacu...................................................   7      1   seq
..............................................Nggaucccaucagaacu...................................................   2      1   seq
..............................................cggaucccaucagaacu...................................................   1460   0   seq
..............................................cggaucccaucagaacuc..................................................   875    0   seq
..............................................cggaCcccaucagaacuc..................................................   3      1   seq
..............................................cggaucccaucagaUcuc..................................................   1      1   seq
..............................................cggaucccUucagaacuc..................................................   1      1   seq
..............................................cggaucAcaucagaacuc..................................................   1      1   seq
..............................................cggaucccaucagaacCc..................................................   12     1   seq
..............................................cggGucccaucagaacuc..................................................   4      1   seq
..............................................cggaucccaucagCacuc..................................................   1      1   seq
..............................................cggaucccaucagaacucc.................................................   27     0   seq
..............................................cggaucccaucagaacuccC................................................   1      1   seq
..............................................cggaucccUucagaacuccg................................................   1      1   seq
..............................................cggaucccaucagaacuccg................................................   27     0   seq
..............................................cggaucccaucagaacuccU................................................   1      1   seq
..............................................cggaucccaucagaacuccgA...............................................   5      1   seq
..............................................Gggaucccaucagaacuccgu...............................................   1      1   seq
..............................................cggaucccaucagaacuccCu...............................................   1      1   seq
..............................................cggaCcccaucagaacuccgu...............................................   1      1   seq
..............................................cggaucccaucaAaacuccgu...............................................   1      1   seq
..............................................cggaucccaucagaacuccgu...............................................   25     0   seq
..............................................cggaucccaucagaacucAgua..............................................   1      1   seq
..............................................cggaucccaucagaacuccguC..............................................   1      1   seq
..............................................cggaucccaucagaacuccgua..............................................   4      0   seq
..............................................cggaucccaucagaacuccgGa..............................................   1      1   seq
...............................................ggaucccaucagaacuc..................................................   132    0   seq
...............................................ggaucccaucagaacCc..................................................   2      1   seq
...............................................ggaCcccaucagaacuc..................................................   1      1   seq
...............................................ggaucccaucagaacucc.................................................   3      0   seq
...............................................ggaucccaucagaacuccg................................................   1      0   seq
...............................................ggCucccaucagaacuccg................................................   1      1   seq
...............................................ggaucccaucagaacuccgu...............................................   3      0   seq
...............................................Ugaucccaucagaacuccguaguu...........................................   1      1   seq
................................................gaucccaucagaacCcc.................................................   2      1   seq
................................................gaCcccaucagaacucc.................................................   2      1   seq
................................................gaucccaucagaacucc.................................................   561    0   seq
................................................gaucccaAcagaacucc.................................................   1      1   seq
................................................gaCcccaucagaacuccg................................................   7      1   seq
................................................gaucccaucagaUcuccg................................................   1      1   seq
................................................gaucccaucagaacCccg................................................   7      1   seq
................................................gaucccaucagaaGuccg................................................   1      1   seq
................................................gaucccaucagaacuccg................................................   2549   0   seq
................................................gaucccaucaCaacuccg................................................   1      1   seq
................................................gaucccCucagaacuccg................................................   1      1   seq
................................................gaucccaucagCacuccg................................................   1      1   seq
................................................Naucccaucagaacuccg................................................   4      1   seq
................................................gaucccaucagaacuccgu...............................................   342    0   seq
................................................gaucccaucagaacucGgu...............................................   1      1   seq
................................................gaucccaucagaacuccgA...............................................   71     1   seq
................................................gaucccaucagaacuccgG...............................................   15     1   seq
................................................gaucccaucagGacuccgu...............................................   1      1   seq
................................................gauccUaucagaacuccgu...............................................   1      1   seq
................................................gaCcccaucagaacuccgu...............................................   1      1   seq
................................................gaucccaucagaGcuccgu...............................................   1      1   seq
................................................gaucccaucagaacGccgu...............................................   1      1   seq
```

```
                                            Mature                                      Star
agggggcgugauucuugcgugugcgaucauaccagcguuaauguaccggaucccaucagaacuccguaguuaagcacgcuugugcuagaguaguauugggaugggugaucuccc
................................................gaucccaucagaacuccgGa..............................................    4      1   seq
................................................gaucccaucagaacuccguG..............................................    5      1   seq
................................................gaucccaucagaacuccgua..............................................    19     0   seq
................................................gaucccaucagaacuccgAa..............................................    1      1   seq
................................................gaucccaucagaacuccguag.............................................    2      0   seq
................................................gaucccaucagaacuccgGag.............................................    1      1   seq
................................................gaucccaucagaacuccguagu............................................    4      0   seq
................................................gaucccaucagaacuccgAagu............................................    2      1   seq
................................................gaucccaucagaacuccguaguA...........................................    1      1   seq
.................................................aCcccaucagaacuccg................................................    8      1   seq
.................................................aucccCucagaacuccg................................................    1      1   seq
.................................................aucccaucagaacCccg................................................    6      1   seq
.................................................aucccUucagaacuccg................................................    1      1   seq
.................................................Cucccaucagaacuccg................................................    2      1   seq
.................................................Nucccaucagaacuccg................................................    3      1   seq
.................................................aucccaucagaacuccg................................................    2403   0   seq
.................................................aucccaucGgaacuccgu...............................................    2      1   seq
.................................................aucccaucagaacuccgG...............................................    4      1   seq
.................................................Gucccaucagaacuccgu...............................................    1      1   seq
.................................................aucccaucagaGcuccgu...............................................    1      1   seq
.................................................auUccaucagaacuccgu...............................................    1      1   seq
.................................................aucccaucUgaacuccgu...............................................    1      1   seq
.................................................aucccaucagaacuccgu...............................................    125    0   seq
.................................................aAcccaucagaacuccgu...............................................    1      1   seq
.................................................Gucccaucagaacuccgua..............................................    1      1   seq
.................................................aucccaucagaacuccgGa..............................................    3      1   seq
.................................................aucccaucagaacuccgua..............................................    7      0   seq
.................................................aucccaucagaacuccguaUu............................................    1      1   seq
.................................................aucccaucagaacuccguagu............................................    4      0   seq
.................................................aucccaucagaacuccguaguu...........................................    1      0   seq
.................................................aucccaucagaacuccguaguuaag........................................    1      0   seq
..................................................ucccaucagaacuccgG...............................................    21     1   seq
..................................................ucccaucUgaacuccgu...............................................    1      1   seq
..................................................ucccaucagaacuccgu...............................................    641    0   seq
..................................................ucccaucagaacCccgu...............................................    1      1   seq
..................................................Ccccaucagaacuccgu...............................................    3      1   seq
..................................................ucccaucagaGcuccgu...............................................    2      1   seq
..................................................Ncccaucagaacuccgu...............................................    3      1   seq
..................................................ucccaucagaacuGcgu...............................................    1      1   seq
..................................................ucccaucagGacuccgu...............................................    5      1   seq
..................................................ucccUucagaacuccgu...............................................    1      1   seq
..................................................ucccaucagaacucGgu...............................................    2      1   seq
..................................................ucccaucGgaacuccgu...............................................    3      1   seq
..................................................ucccaucagaacuccguG..............................................    2      1   seq
..................................................ucccaucagaacuccgua..............................................    32     0   seq
..................................................ucccaucagaacuccguC..............................................    1      1   seq
..................................................ucccaucagaacuccgGa..............................................    10     1   seq
..................................................ucccaucagaacuccguag.............................................    5      0   seq
..................................................ucccaucagaacuccguagu............................................    2      0   seq
..................................................ucccaucagaacuccguaguC...........................................    1      1   seq
..................................................ucccaucagaacuccguaguu...........................................    3      0   seq
..................................................ucccaucagaacuccguaguua..........................................    5      0   seq
..................................................ucccaucagaacuccguaguuaagc.......................................    2      0   seq
...................................................cccaucagaacuccgGa..............................................    3      1   seq
...................................................cccaucagaacuccguC..............................................    1      1   seq
...................................................cccaucagaacuccguG..............................................    4      1   seq
...................................................cccaucagaacuccgua..............................................    25     0   seq
...................................................cccaucagaacuccguag.............................................    1      0   seq
...................................................cccaucagaacuccgGag.............................................    1      1   seq
...................................................cccaucagaacuccguagu............................................    3      0   seq
...................................................cccaucagaacuccguagG............................................    1      1   seq
...................................................cccaucagaacuccgGagu............................................    2      1   seq
...................................................cccaucagaacuccgGaguu...........................................    1      1   seq
...................................................cccaucagaacuccguaguu...........................................    5      0   seq
...................................................cccaucagaacucGguaguua..........................................    1      1   seq
...................................................cccaucagaacuccguaguua..........................................    1      0   seq
...................................................cccaucagaacuccguaguuaa.........................................    1      0   seq
....................................................ccaucagaacuccguag.............................................    5      0   seq
....................................................ccaucagaacuccguagu............................................    4      0   seq
....................................................ccaucagaacuccgGagu............................................    1      1   seq
```

```
                                             Mature                                    Star
agggggcgugauucuugcgugugcgaucauaccagcguuaauguaccggaucccaucagaacuccguaguuaagcacgcuugugcuagaguaguauugggauggguggaucuccc
....................................................ccaucagaacuccguaguu...........................................    4    0    seq
....................................................ccaucagaacuccguaguua..........................................    2    0    seq
....................................................ccaucagaacuccguaguuaagc.......................................    1    0    seq
.....................................................caucagaacuccguagu............................................    1    0    seq
.....................................................caucagaacuccguaguu...........................................    1    0    seq
.....................................................caucagaacuccguaguua..........................................    1    0    seq
.....................................................caucagaacuccguaguuaa.........................................    1    0    seq
......................................................aucagaacuccguaguu...........................................    1    0    seq
......................................................aucagaacuccguaguua..........................................    2    0    seq
......................................................aucagaacuccgGaguua..........................................    1    1    seq
......................................................aucagaacuccgGaguuaagc.......................................    1    1    seq
.......................................................ucagaacuccguaguua..........................................    5    0    seq
.......................................................ucagaacuccguaguuaag........................................    1    0    seq
.......................................................ucagaacuccguaguuaagcC......................................    1    1    seq
........................................................cagaacuccguaguuaagc.......................................    1    0    seq
.........................................................agaacuccguaguuaagc.......................................    1    0    seq
...................................................................aguuaagcGcgcuugug..............................    1    1    seq
....................................................................guuaagcGcgcuugugc.............................    1    1    seq
....................................................................guuaagcGcgcuugugcuaga.........................    1    1    seq
....................................................................guuaagcGcgcuugugcuagagu.......................    1    1    seq
....................................................................guuaagcGcgcuugugcuagagua......................    2    1    seq
............................................................................cgcuugugcuagaguaguaC..................    1    1    seq
............................................................................cgcuugugcuagaguaguaCug................    1    1    seq
............................................................................cgcuugugcuagaguaguaCuggga.............    1    1    seq
.............................................................................gcuugugcuagaguagu....................    1    0    seq
.............................................................................gcuugugcuagaguaguaCugg...............    1    1    seq
.............................................................................gcuugugcuagaguaguaCugggau............    1    1    seq
.............................................................................gcuugugcuagaguaguaCugggaug...........    1    1    seq
..............................................................................cuugugcuagaguaguaCu.................    2    1    seq
..............................................................................cuugugcuagaguaguaCuggg..............    1    1    seq
..............................................................................cuugugcuagaguaguaCuggga.............    2    1    seq
..............................................................................cuugugcuagaguaguaCugggaug...........    2    1    seq
..............................................................................cuugugcuagaguaguaCugggaugg..........    1    1    seq
..............................................................................cuugugcuagaguaguaCugggaugggug.......    1    1    seq
...............................................................................uugugcuagaguaguaC..................    1    1    seq
...............................................................................uugugcuagaguaguaCu.................    1    1    seq
...............................................................................uugugcuagaguaguaCug................    1    1    seq
...............................................................................uugugcuagaguaguaCugggau............    1    1    seq
...............................................................................uugugcuagaguaguaCugggaug...........    1    1    seq
...............................................................................uugugcuagaguaguaCugggaugggug.......    1    1    seq
................................................................................ugugcuagaguaguaCug................    2    1    seq
................................................................................ugugcuagaguaguaCugg...............    2    1    seq
................................................................................ugugcuagaguaguaCuggga.............    3    1    seq
................................................................................ugugcuagaguaguaCugggau............    1    1    seq
................................................................................ugugcuagaguaguaCugggaug...........    3    1    seq
................................................................................ugugcuagaguaguaCugggaugg..........    1    1    seq
................................................................................ugugcuagaguaguaCugggauggg.........    2    1    seq
................................................................................ugugcuagaguaguaCugggaugggu........    4    1    seq
................................................................................ugugcuagaguaguaCugggaugggug.......    2    1    seq
...................................................................................gcuagaguaguauuggg..............    2    0    seq
...................................................................................gcuagaguaguaGuggg..............    1    1    seq
...................................................................................gcuagaguaguaGuggga.............    3    1    seq
...................................................................................gcuagaguaguauuggga.............    6    0    seq
...................................................................................gcuagaguaguauugggau............    2    0    seq
...................................................................................gcuagaguaguauugggaug...........    3    0    seq
...................................................................................gcuagaguaguauugggaugg..........    1    0    seq
...................................................................................gcuagaguaguauugggauggg.........    2    0    seq
...................................................................................gcuagaguaguauugggaugggu........    1    0    seq
....................................................................................cuagaguaguauuggga.............    3    0    seq
....................................................................................cuagaguaguauugggau............    1    0    seq
....................................................................................cuagaguaguauugggaug...........    3    0    seq
....................................................................................cuagaguaguauugggauggg.........    2    0    seq
....................................................................................cuagaguaguauugggaugggu........    5    0    seq
....................................................................................cuagaguaguauugggaugggug.......    2    0    seq
.....................................................................................uagaguaguauugggau............    4    0    seq
.....................................................................................uagaguaguauugggaug...........    2    0    seq
.....................................................................................uagaguaguauugggauggg.........    3    0    seq
......................................................................................agaguaguauugggaug...........    1    0    seq
......................................................................................agaguaguauugggauggg.........    1    0    seq
......................................................................................agaguaguauugggaugggu........    1    0    seq
```

Mature **Star**

```
agggggcgugauucuugcgugugcgaucauaccagcguuaauguaccggaucccaucagaacuccguaguuaagcacgcuugugcuagaguaguauugggaugggugaucuccc
.......................................................................................gaguaguauugggaugg..........	1	0	seq
.......................................................................................gaguaguauugggauggg.........	4	0	seq
.......................................................................................gaguaguauugggaugggC........	1	1	seq
.......................................................................................gaguaguauugggaugggu........	1	0	seq
........................................................................................aguaguauugggauggg.........	2	0	seq
........................................................................................aguaguauugggaugggu........	3	0	seq
........................................................................................aguaguauugggaugggug.......	5	0	seq
.........................................................................................guaguauugggaugggu........	5	0	seq
.........................................................................................guaguauugggaugggug.......	3	0	seq
..........................................................................................uaguauugggaugggug.......	8	0	seq
..........................................................................................uaguauugggaugCgug.......	1	1	seq
```

Provisional ID	:	MA_13409_14525
Score total	:	0.5
Score for star read(s)	:	-1.3
Score for read counts	:	0
Score for mfe	:	0.2
Score for randfold	:	1.6
Score for cons. seed	:	
Total read count	:	229
Mature read count	:	229
Loop read count	:	0
Star read count	:	0

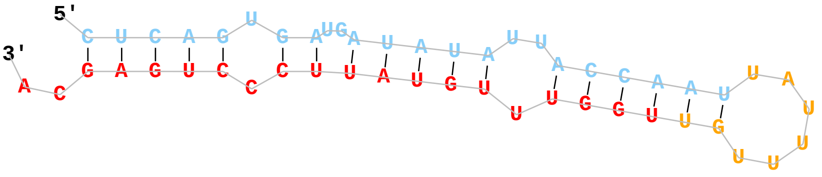

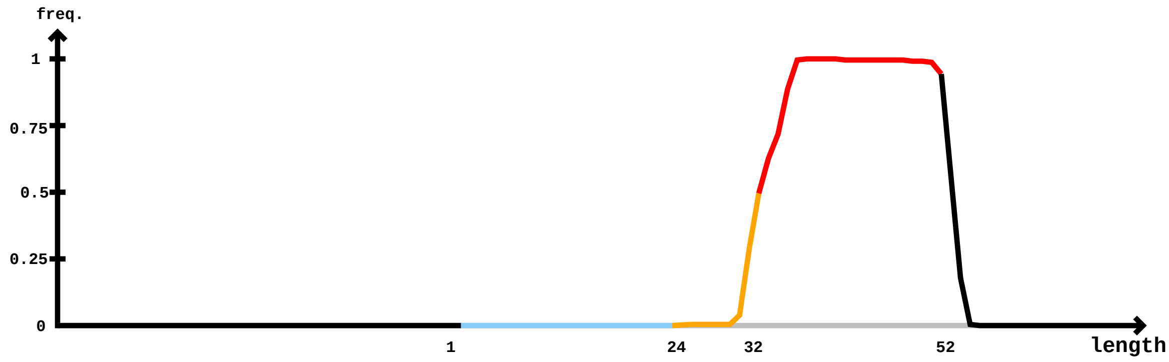

Star Mature

5'- gcuguggaaaaucuuuaggcaaacuguauugucaccaauucucagugaugauauauuaccaauuauuuuguugguuuguauucccugagcauucuauuuccauggaaaaaa -3'	exp		
.(((((((((.......(((((......)))))...(((.(((((.((..(((((..((((((......)))))).))))))).))))).)))...)))))))))......	reads	mm	sample
....uauuuuguugguuugua...............................	1	0	seq
....Auugguuuguauucccugagca....................	6	1	seq
....Auugguuuguauucccugagcau...................	2	1	seq
....uugguuuguauucccug........................	1	0	seq
....uugguuuguauucccugagc.....................	2	0	seq
....uugguuuguauucccugagca....................	15	0	seq
....uugguuuguauucccugagcG....................	2	1	seq
....uugguuuguauucccCgagca....................	1	1	seq
....uugguuugCauucccugagca....................	1	1	seq
....uugguuuguauucccugaCca....................	1	1	seq
....uugguuuguauucccugagcau...................	4	0	seq
....uugguuuguauucccugagcaC...................	1	1	seq
....uugguuuguauucccugagcauC..................	26	1	seq
....uugguuuguauucccugagcauu..................	2	0	seq
....uugguuuguauucccugagcauCc.................	1	1	seq
....ugguuuguauucccugag......................	1	0	seq
....ugguuuguauucccugagU.....................	1	1	seq
....ugguuuguauucccugagc.....................	4	0	seq
....ugguuuguauucccugagcC....................	1	1	seq
....ugguuuguauucccugagcU....................	1	1	seq
....ugguuuguauucccugagca....................	29	0	seq
....ugguuuguauucccugagcau...................	7	0	seq
....ugguuuguauucccugagcGu...................	1	1	seq
....ugguuuguauucccugagcaG...................	1	1	seq
....ugguuuguauucccugagcauu..................	2	0	seq
....gguuuguauucccugagU.....................	1	1	seq
....gguuuguauucccugagc.....................	1	0	seq
....gguuuguauucccugagca....................	15	0	seq
....Aguuuguauucccugagca....................	1	1	seq
....gUuuuguauucccugagcau...................	5	1	seq
....gguuuguauucccugagcau...................	5	0	seq
....gguuuguauucccugUgcau...................	1	1	seq
....gUuuuguauucccugagcauu..................	1	1	seq
....guuuguauucccugagc.....................	1	0	seq

```
                                        Star                            Mature
gcuguggaaaaucuuuaggcaaacuguauugucaccaauucucagugaugauauauuaccaauuauuuuguugguuuguauucccugagcauucuauuuccauggaaaaaa
.........................................................................guuuguauucccugGgca....................  1    1    seq
.........................................................................guuuguauucccugagca....................  10   0    seq
.........................................................................Uuuuguauucccugagca....................  6    1    seq
.........................................................................Uuuuguauucccugagcau...................  1    1    seq
.........................................................................guuuguauucccugagcau...................  2    0    seq
..........................................................................uuuguauucccugagcC....................  1    1    seq
..........................................................................uuuguauucccugagcG....................  2    1    seq
..........................................................................uuCguauucccugagca....................  1    1    seq
..........................................................................uuuguauucccugagca....................  18   0    seq
..........................................................................uuuguauucccugagcaA...................  1    1    seq
..........................................................................uuuguauucccugagcaC...................  4    1    seq
..........................................................................Cuuguauucccugagcau...................  1    1    seq
..........................................................................uuuguauucccugagcau...................  6    0    seq
..........................................................................Nuuguauucccugagcau...................  1    1    seq
..........................................................................uuuguauucccugagcauu..................  1    0    seq
..........................................................................uuuguauucccugagcauA..................  2    1    seq
..........................................................................Nuuguauucccugagcauu..................  1    1    seq
...........................................................................uuguauucccugagcaA...................  1    1    seq
...........................................................................uuguauucccugagcau...................  15   0    seq
...........................................................................uuguauucccugagcaG...................  1    1    seq
...........................................................................Nuguauucccugagcau...................  1    1    seq
...........................................................................uuguauucccugagcaC...................  3    1    seq
...........................................................................uuguauucccugagcauu..................  2    0    seq
...........................................................................uuguauucccugagcauA..................  2    1    seq
............................................................................uguauucccugagcauA..................  1    1    seq
```

Provisional ID	:	MA_13504_3665
Score total	:	1.5
Score for star read(s)	:	-1.3
Score for read counts	:	0
Score for mfe	:	1.2
Score for randfold	:	1.6
Score for cons. seed	:	
Total read count	:	17
Mature read count	:	17
Loop read count	:	0
Star read count	:	0

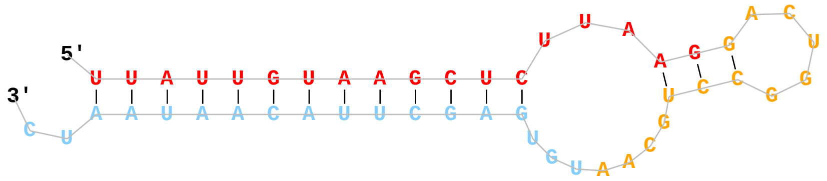

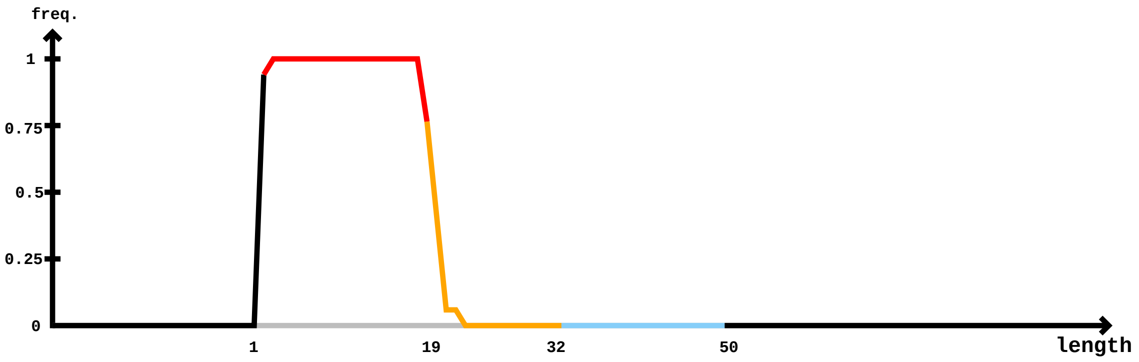

Mature Star

```
5'- aauuucugacaacaaaaggauuauuguaagcucuuaaggacuggccugcaaugugagcuuacaauaaauccucaccagaaauuguugcaauaaaucuuacaagacuggu   -3'    exp
    ...............((((((((((((((((...(((.....)))......))))))))))))))))).(((((...((((............))))..)))))    reads        mm          sample
    ...................uuauuguaagcucuuaC...................................................................    2            1           seq
    ...................uuauuguaagcucuuaa...................................................................    2            0           seq
    ...................uuauuguaagcucuuaaC..................................................................    5            1           seq
    ...................uuauuguaagcucuuaag..................................................................    4            0           seq
    ...................uuauuguaagcucuuaagC.................................................................    2            1           seq
    ...................uuauuguaagcucuuaagUac...............................................................    1            1           seq
    ....................uauuguaagcucuuaag..................................................................    1            0           seq
```

Provisional ID	:	MA_135470_9724
Score total	:	22.3
Score for star read(s)	:	3.9
Score for read counts	:	25.6
Score for mfe	:	-4.9
Score for randfold	:	-2.2
Score for cons. seed	:	
Total read count	:	62
Mature read count	:	60
Loop read count	:	1
Star read count	:	1

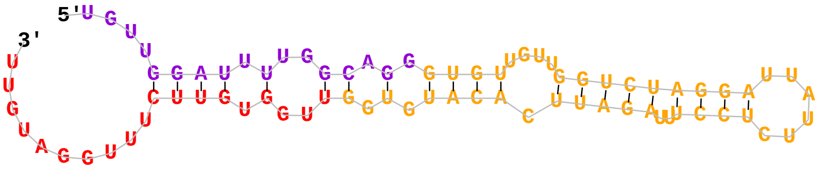

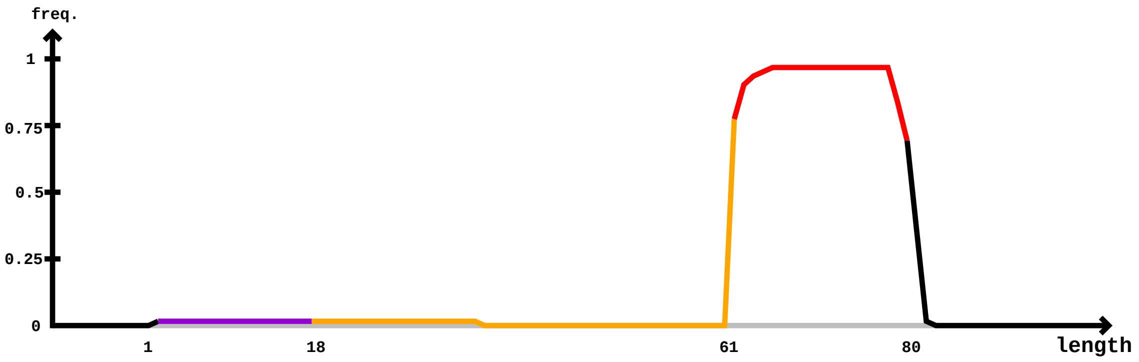

Star Mature

```
5'- uaugaaggauguuggauuuuggcaggguguuguuggucuaggauuauucuccuuuagauucacauguggguugguguucuuuggauguucaagaugcuauaguauuauc  -3'  obs
    uaugaaggauguuggauuuuggcagggguguuguuggucuaggauuauucuccuuuagauucacauguggguugguguucuuuggauguucaagaugcuauaguauuauc       exp
    ......((((((((((.(..((.(.((((....((((((((......))))..))))).)))).).))..).))))....)))))))..(((((....)))))...   reads   mm   sample
    .........uguuggaAuuuggcagg..................................................................................   1       1    seq
    ..........................guguuguCggucuagga.................................................................   1       1    seq
    .....................................................................Augguguucuuuggaug......................   8       1    seq
    .....................................................................Augguguucuuuggaugu.....................   9       1    seq
    .....................................................................Augguguucuuuggauguu....................   30      1    seq
    .....................................................................uugguguucuuuggauguuG...................   1       1    seq
    ......................................................................uggCguucuuuggauguu....................   1       1    seq
    ......................................................................ugguguucuuuggauguu....................   4       0    seq
    ......................................................................ugguguucuuuggauguC....................   1       1    seq
    ......................................................................ugguguucuuuggauguuG...................   2       1    seq
    .......................................................................gguguucuuuggauguu....................   1       0    seq
    .......................................................................gguguucuuuggauguC....................   1       1    seq
    ........................................................................guguucuuuggauguuG...................   1       1    seq
    .........................................................................uguucuuuggauguuGa..................   1       1    seq
```

Provisional ID	:	MA_136180_22594
Score total	:	19630.4
Score for star read(s)	:	3.9
Score for read counts	:	19622.8
Score for mfe	:	2.2
Score for randfold	:	1.6
Score for cons. seed	:	
Total read count	:	38501
Mature read count	:	19558
Loop read count	:	0
Star read count	:	18943

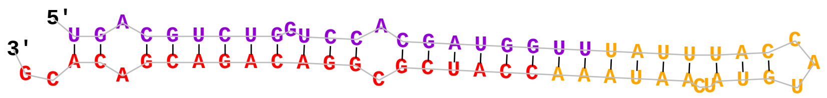

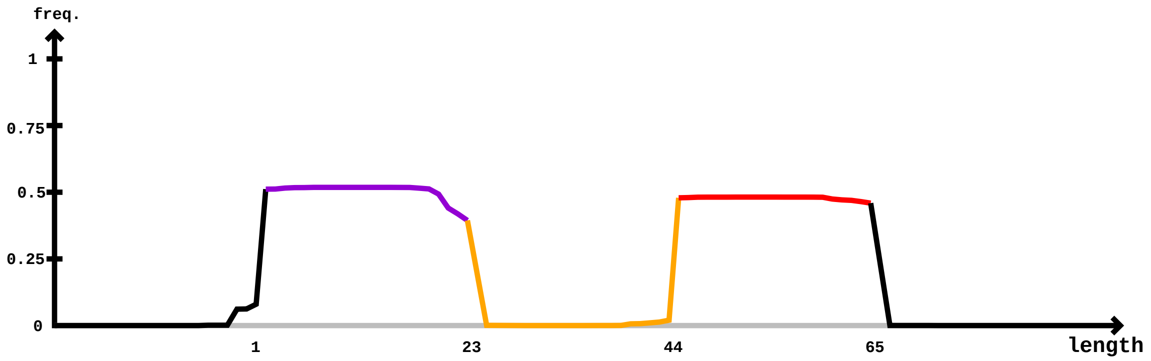

Star Mature

```
5'- aaagaaagcgaaggugguc uugacgucugguccacgaugguuuauuuaccauguaucaauaaaccaucgcggacagacgacacgagcaccuucaccuucguguacaugg  -3'   obs
    aaagaaagcgaaggugguc uugacgucugguccacgaugguuuauuuaccauguaucaauaaaccaucgcggacagacgacacgagcaccuucaccuucguguacaugg         exp
    ...(((.(.(((((((.((.((.((((((.(((.(((((((((((((((...)))..)))))))))))).))))))))).)).)).))))))).).))).........   reads   mm   sample
    ..............uggucuugacgucuggAcc............................................................................   2       1    seq
    ..............ugUcuugacgucuggucca............................................................................   3       1    seq
    ..............uggucuugacgucuggAccac..........................................................................   4       1    seq
    ..............uggucuugacgucuggAccacg.........................................................................   21      1    seq
    ..............uggucuugacgucuggGccacg.........................................................................   8       1    seq
    ..............ugUcuugacgucugguccacg..........................................................................   14      1    seq
    ...............gUucuugacgucugguccac..........................................................................   1       1    seq
    .................ucuugacgucuggGcca...........................................................................   6       1    seq
    .................ucuugacgucuggGccac..........................................................................   3       1    seq
    .................ucuugacgucuggGccacg.........................................................................   27      1    seq
    .................ucuugacgucuggAccacga........................................................................   63      1    seq
    .................ucuugacgucuggGccacga........................................................................   2       1    seq
    .................ucuugacgucuggAccacgau.......................................................................   678     1    seq
    .................ucuugacgucuggGccacgau.......................................................................   6       1    seq
    .................ucuugacgucuggAccacgaug......................................................................   1507    1    seq
    .................ucuugacgucuggGccacgaug......................................................................   157     1    seq
    .................ucuugacgucuggAccacgaugg.....................................................................   1       1    seq
    .................ucuugacgucuggAccacgauggu....................................................................   38      1    seq
    .................ucuugacgucuggAccacgaugguu...................................................................   11      1    seq
    .................ucuugacgucuggAccacgaugguuu..................................................................   3       1    seq
    ..................cuugacgucuggGccacg.........................................................................   26      1    seq
    ..................cuugacgucuggAccacgau.......................................................................   1       1    seq
    ..................cuugacgucuggAccacgaug......................................................................   1       1    seq
    ..................cuugacgucuggAccacgauggu....................................................................   1       1    seq
    ...................uugacgucuggGccacg.........................................................................   12      1    seq
    ...................uugacgucuggAccacga........................................................................   8       1    seq
    ...................uugacgucuggAccacgau.......................................................................   28      1    seq
    ...................uugacgucuggAccacgaug......................................................................   41      1    seq
    ...................uugacgucuggGccacgaugg.....................................................................   2       1    seq
    ...................uugacgucuggAccacgaugg.....................................................................   597     1    seq
    ...................uugacgucuggGccacgauggu....................................................................   6       1    seq
    ...................uugacgucuggAccacgauggu....................................................................   16      1    seq
    ...................uugacgucuggAccacgaugguu...................................................................   14      1    seq
```

Star Mature

```
aaagaaagcgaagguggucuugacgucugguccacgaugguuauuuaccauguaucaauaaaccaucgcggacagacgacacgagcaccuucaccuucguguacaugg
...................Augacgucugguccacgauggu...................................................................    6    1    seq
...................Cugacgucugguccacgauggu...................................................................    3    1    seq
...................uugacgucuggAccacgaugguuu.................................................................    4    1    seq
...................uugacgucuggGccacgaugguuu.................................................................    1    1    seq
....................ugacgucugguccacUa.......................................................................    1    1    seq
....................ugacgucuggGccacga.......................................................................    1    1    seq
....................ugacgucugguccacCa.......................................................................    2    1    seq
....................ugacgucugguccacga.......................................................................    39   0    seq
....................ugacgucCgguccacga.......................................................................    1    1    seq
....................ugaGgucugguccacga.......................................................................    1    1    seq
....................ugacgucugguccGcgau......................................................................    1    1    seq
....................ugacgucugguccacgaG......................................................................    4    1    seq
....................uAacgucugguccacgau......................................................................    1    1    seq
....................ugacgucuggAccacgau......................................................................    2    1    seq
....................ugacgucugguccacgGu......................................................................    1    1    seq
....................ugacgucuggucUacgau......................................................................    1    1    seq
....................ugacgucugguccacgaC......................................................................    16   1    seq
....................Cgacgucugguccacgau......................................................................    1    1    seq
....................ugacgucugguccacgau......................................................................    60   0    seq
....................ugacgucuggGccacgau......................................................................    3    1    seq
....................ugacgucugguccacgaCg.....................................................................    3    1    seq
....................ugacgucuggucAacgaug.....................................................................    1    1    seq
....................ugacgucugguccacgauU.....................................................................    6    1    seq
....................ugacgucugguccacgGug.....................................................................    1    1    seq
....................ugacgucugguccacgaug.....................................................................    399  0    seq
....................ugacgucugguccacgaAg.....................................................................    1    1    seq
....................ugacgucuggGccacgaug.....................................................................    1    1    seq
....................ugacgucugguccGcgaug.....................................................................    2    1    seq
....................ugGcgucugguccacgaug.....................................................................    4    1    seq
....................ugacgucugguccacgauC.....................................................................    1    1    seq
....................ugacgucCgguccacgaug.....................................................................    2    1    seq
....................ugacgucugguccacgauA.....................................................................    16   1    seq
....................ugacgucugguccacgaGg.....................................................................    1    1    seq
....................ugacCucugguccacgaug.....................................................................    1    1    seq
....................ugacgucuggAccacgaug.....................................................................    3    1    seq
....................Agacgucugguccacgaugg....................................................................    1    1    seq
....................ugaUgucugguccacgaugg....................................................................    1    1    seq
....................ugacgucugguccacAaugg....................................................................    1    1    seq
....................ugacgucuggGccacgaugg....................................................................    1    1    seq
....................ugacgucuggAccacgaugg....................................................................    8    1    seq
....................ugacgCcugguccacgaugg....................................................................    1    1    seq
....................ugacgucugguccacgaugC....................................................................    5    1    seq
....................ugacgucugguccacgaCgg....................................................................    1    1    seq
....................Cgacgucugguccacgaugg....................................................................    1    1    seq
....................ugacgucugguccacgaugA....................................................................    16   1    seq
....................ugacgucCgguccacgaugg....................................................................    1    1    seq
....................ugGcgucugguccacgaugg....................................................................    1    1    seq
....................ugacgucugguccacgaugg....................................................................    257  0    seq
....................ugacgucugguUcacgaugg....................................................................    2    1    seq
....................ugacgucugguccacgaugU....................................................................    7    1    seq
....................ugacgucuggCccacgaugg....................................................................    1    1    seq
....................ugacgucugguccacgGuggu...................................................................    3    1    seq
....................ugacgucuggAccacgauggu...................................................................    172  1    seq
....................ugacgucugguccacgauggC...................................................................    108  1    seq
....................ugacgucugguccacgauggG...................................................................    9    1    seq
....................ugacgucugguccacgaugAu...................................................................    5    1    seq
....................ugacgucuCguccacgauggu...................................................................    1    1    seq
....................Ggacgucugguccacgauggu...................................................................    1    1    seq
....................ugacgucuggGccacgauggu...................................................................    8    1    seq
....................ugacgucugguccacgaugUu...................................................................    2    1    seq
....................ugacgucuAguccacgauggu...................................................................    1    1    seq
....................ugacgucugAuccacgauggu...................................................................    1    1    seq
....................Cgacgucugguccacgauggu...................................................................    1    1    seq
....................ugacgucugguccUcgauggu...................................................................    1    1    seq
....................ugaUgucugguccacgauggu...................................................................    2    1    seq
....................Agacgucugguccacgauggu...................................................................    1    1    seq
....................ugacgucugguccacgaugCu...................................................................    3    1    seq
....................ugacgucugguccacgauggA...................................................................    7    1    seq
....................ugGcgucugguccacgauggu...................................................................    2    1    seq
....................ugacgucugguccacgauggu...................................................................    611  0    seq
```

Star **Mature**

aaagaaagcgaaggugucuugacgucugguccacgaugguuuauuuaccauguaucaauaaaccaucgcggacagacgacacgagcaccuucaccuucguguacaugg			
...................ugacgucugguccGcgauggu..	1	1	seq
...................ugacgucuggucUacgauggu..	1	1	seq
...................ugacgucuggCccacgaugguu...	36	1	seq
...................uUacgucugguccacgaugguu...	34	1	seq
...................ugacgucugCuccacgaugguu...	2	1	seq
...................ugacgucugguccUcgaugguu...	12	1	seq
...................ugacgucugguccacgaugguA...	198	1	seq
...................ugacgucugguGcacgaugguu...	1	1	seq
...................ugacguAugguccacgaugguu...	1	1	seq
...................ugacgucugguccacgauggAu...	22	1	seq
...................ugacgucugguccacgauggCu...	70	1	seq
...................ugacgucuUguccacgaugguu...	2	1	seq
...................ugacgucugguccacgaugUuu...	2	1	seq
...................Ggacgucugguccacgaugguu...	24	1	seq
...................ugacgucugguccacgaCgguu...	18	1	seq
...................ugacgucugguccacgaAgguu...	5	1	seq
...................ugacgucCgguccacgaugguu...	22	1	seq
...................ugacgucugUuccacgaugguu...	2	1	seq
...................ugacgucugguccacgauAguu...	10	1	seq
...................ugacgucugguccacgaugAuu...	15	1	seq
...................ugacgucAgguccacgaugguu...	9	1	seq
...................uCacgucugguccacgaugguu...	5	1	seq
...................ugacgucugguccGcgaugguu...	38	1	seq
...................ugacgucugguccacgaugguu...	12393	0	seq
...................ugacgGcugguccacgaugguu...	4	1	seq
...................ugGcgucugguccacgaugguu...	54	1	seq
...................ugacgucugguccacAaugguu...	14	1	seq
...................ugacgucGgguccacgaugguu...	1	1	seq
...................ugacgucugguccacUaugguu...	1	1	seq
...................ugacguUugguccacgaugguu...	10	1	seq
...................ugacgucugguccaGgaugguu...	1	1	seq
...................ugacgucugguccacgGugguu...	39	1	seq
...................ugacgucugguccacgauggGu...	35	1	seq
...................ugacgucugguAcacgaugguu...	2	1	seq
...................ugacgucugAuccacgaugguu...	12	1	seq
...................ugacCucugguccacgaugguu...	2	1	seq
...................ugacgCcugguccacgaugguu...	30	1	seq
...................uAacgucugguccacgaugguu...	16	1	seq
...................Agacgucugguccacgaugguu...	14	1	seq
...................ugacgucuggucGacgaugguu...	3	1	seq
...................Cgacgucugguccacgaugguu...	36	1	seq
...................ugacgucugguccacgCugguu...	1	1	seq
...................ugacgucugguccacgaugCuu...	1	1	seq
...................ugUcgucugguccacgaugguu...	5	1	seq
...................ugacgucuggucUacgaugguu...	22	1	seq
...................Ngacgucugguccacgaugguu...	18	1	seq
...................ugacgucugguccacgaugguC...	1935	1	seq
...................ugacgucugguUcacgaugguu...	10	1	seq
...................ugacgucugguccacgaugguG...	251	1	seq
...................uNacgucugguccacgaugguu...	1	1	seq
...................ugaUgucugguccacgaugguu...	12	1	seq
...................ugacgucugguccacgUugguu...	5	1	seq
...................ugacUucugguccacgaugguu...	1	1	seq
...................ugacgucuAguccacgaugguu...	15	1	seq
...................ugacgucugguccCcgaugguu...	3	1	seq
...................ugacgucuggGccacgaugguu...	219	1	seq
...................ugacgucugguccacgaGgguu...	3	1	seq
...................ugacgucuggAccacgaugguu...	15	1	seq
...................ugaAgucugguccacgaugguu...	2	1	seq
...................ugaGgucugguccacgaugguu...	1	1	seq
...................ugacAucugguccacgaugguu...	14	1	seq
...................ugacgucugguccacgauUguu...	3	1	seq
...................ugacgucugguccaAgaugguu...	3	1	seq
...................ugacgucugguccacgaugguAu..	7	1	seq
...................ugacgucugguccGcgaugguuu..	1	1	seq
...................ugacgucugguccacgaugguuu..	55	0	seq
...................ugacgucugguccacgaugguuC..	244	1	seq
...................ugacgucuggAccacgaugguuu..	4	1	seq
...................ugacgucugguccacgaugguuG..	3	1	seq
...................ugacgucuggGccacgaugguuu..	3	1	seq

Star Mature

```
aaagaaagcgaagguggucuugacgucugguccacgaugguuuauuuaccauguaucaauaaaccaucgcggacagacgacacgagcaccuucaccuucguguacaugg
....................ugacgucugguccacgaugguuA..................................................................	7	1	seq
....................ugacgucugguccacgaugguuCa.................................................................	1	1	seq
....................ugacgucugguccacgaugguuGa.................................................................	1	1	seq
....................ugacgucugguccacgaugguuuC.................................................................	2	1	seq
....................ugacgucugguccacgaugguuuG.................................................................	1	1	seq
....................ugacgucugguccacgaugguuuU.................................................................	3	1	seq
....................ugacgucugguccacgaugguuuCu................................................................	2	1	seq
....................ugacgucugguccacgaugguuGau................................................................	1	1	seq
....................ugacgucugguccacgaugguuuUu................................................................	2	1	seq
....................ugacgucugguccacgaugguuuaCu...............................................................	1	1	seq
....................ugacgucugguccacgaugguuCauu...............................................................	1	1	seq
....................ugacgucugguccacgaugguuuauu...............................................................	1	0	seq
.....................gacgucuggAccacgau.......................................................................	1	1	seq
.....................gacgucugguccacgaug......................................................................	1	0	seq
.....................gacgucuggAccacgaugguu...................................................................	1	1	seq
.....................Uacgucugguccacgaugguu...................................................................	1	1	seq
.....................gacgucugguccacgaugguu...................................................................	6	0	seq
.....................gacgucuggGccacgaugguu...................................................................	2	1	seq
.....................gacgucugguccacAaugguu...................................................................	1	1	seq
.....................gacgucugguccacgaugguuC..................................................................	1	1	seq
.....................gacgucuggAccacgaugguuu..................................................................	1	1	seq
......................acgucuggGccacgaug......................................................................	12	1	seq
......................acgucugguccacgaug......................................................................	2	0	seq
......................acgucuggGccacgaugg.....................................................................	3	1	seq
......................acgucugguccacgaugg.....................................................................	1	0	seq
......................acgucuggGccacgauggu....................................................................	6	1	seq
......................acgucugguccacgauggA....................................................................	1	1	seq
......................acgucuggGccacgaugguu...................................................................	76	1	seq
......................acgucuggAccacgaugguu...................................................................	6	1	seq
......................acgucugguccacgaugguC...................................................................	2	1	seq
......................acgucugguccacgaugguu...................................................................	4	0	seq
......................acgucuggGccacgaugguuu..................................................................	22	1	seq
......................acgucuggAccacgaugguuu..................................................................	21	1	seq
.......................cgucuggAccacgaugg.....................................................................	1	1	seq
.......................cgucugguccacgaugg.....................................................................	2	0	seq
.......................cgucAgguccacgauggu....................................................................	1	1	seq
.......................cgucugguccacgauggu....................................................................	7	0	seq
.......................cgucuggAccacgaugguu...................................................................	1	1	seq
.......................cgucugguccacgaugguu...................................................................	4	0	seq
.......................cgucugguccacgaugguC...................................................................	1	1	seq
.......................cgucuggGccacgaugguu...................................................................	44	1	seq
.......................cgucuggGccacgaugguuu..................................................................	2	1	seq
.......................cgucuggAccacgaugguuu..................................................................	3	1	seq
........................gucugguccacgauggu....................................................................	3	0	seq
........................gucugguccacgaugguu...................................................................	3	0	seq
........................gucugguccacgaugguC...................................................................	1	1	seq
.........................ucugguccacgaugguu...................................................................	13	0	seq
.........................ucugguccacgaugguC...................................................................	3	1	seq
.........................ucugguccacgaugguuu..................................................................	1	0	seq
.........................ucugguccacgaugguuC..................................................................	1	1	seq
.........................ucugguccacgaugguuua.................................................................	1	0	seq
.........................ucugguccacgaugguuuauu...............................................................	1	0	seq
.........................ucuggAccacgaugguuuauu...............................................................	7	1	seq
.........................ucugguccacgaugguuuauuu..............................................................	4	0	seq
.........................ucuggGccacgaugguuuauuu..............................................................	2	1	seq
.........................ucugguccacgauggCuuauuu..............................................................	1	1	seq
...................................................ugCaucaauaaaccaucgcggac...................................	1	1	seq
.....................................................Caucaauaaaccaucgcgga....................................	1	1	seq
......................................................aucaauaaaccaucgcggacagacg..............................	1	0	seq
......................................................aucaauaaaccaucgcggacagacga.............................	1	0	seq
......................................................aucaauaaaccaucgcggacagacgacU...........................	1	1	seq
.......................................................ucaauaaaccaucgcggaca..................................	1	0	seq
.......................................................ucaauaaaccaucgcggacagac...............................	1	0	seq
.......................................................ucaauaaaccaucgcggacagacgacaUg.........................	1	1	seq
.........................................................aauaaaccaucgcggacagGc...............................	1	1	seq
.........................................................aauaaaccaucgcggacagac...............................	9	0	seq
.........................................................aauaaaccaucgcggacagaUg..............................	1	1	seq
.........................................................aauaaaccaucgcggacagacg..............................	1	0	seq
..........................................................auaaaccaucgcggaca..................................	1	0	seq
..........................................................auaaaccaucgcggacagU................................	1	1	seq
```

Star Mature

```
aaagaaagcgaaggugguc uugacgucugguccacgaugguuuauuuaccauguaucaauaaaccaucgcggacagacgacacgagcaccuucaccuucguguacaugg
..........................................................auaaaccaucgcggacagaU...............................   2     1   seq
..........................................................auaaaccaucgcggacagac...............................   4     0   seq
..........................................................Nuaaaccaucgcggacagacg..............................   1     1   seq
..........................................................auaaaccaucgcggGcagacg..............................   1     1   seq
..........................................................auaaaccauUgcggacagacg..............................   2     1   seq
..........................................................auaaaccaucgcggacagacU..............................   2     1   seq
..........................................................auaaaccaucgcggacagacC..............................   2     1   seq
..........................................................auaaaccaucgcggacCgacg..............................   1     1   seq
..........................................................auaaaccaucgcggacagacg..............................   204   0   seq
..........................................................auaaaccaucgcggacagaAg..............................   1     1   seq
..........................................................auaaaccaucgcggacagaUg..............................   1     1   seq
..........................................................auaaaccaucgcggacagGcg..............................   2     1   seq
..........................................................auaaaccaucgcggacagacA..............................   18    1   seq
..........................................................auaaaccaucgUggacagacg..............................   1     1   seq
..........................................................auaaaccaucUcggacagacg..............................   1     1   seq
..........................................................auaaaccaucgcggacGgacg..............................   2     1   seq
..........................................................auaaaccaucgcggacagacga.............................   1     0   seq
..........................................................auaaaccaucgcggacagacgC.............................   1     1   seq
...........................................................uaaaccCucgcggacag.................................   1     1   seq
...........................................................uaaaccaucgcggacagacg..............................   20    0   seq
...........................................................uaaaccaucgcggacagacA..............................   1     1   seq
...........................................................uaaaccaucgcggacagaAg..............................   1     1   seq
...........................................................uaaaccaucgcggacagacgac............................   1     0   seq
...........................................................uaaaccaucgcggacagacgaca...........................   1     0   seq
...........................................................uaaaccaucgcggacagacgaAa...........................   1     1   seq
...........................................................uaaaccaucgcggacagacgacaUg.........................   2     1   seq
............................................................aaaccaucgcggacagacg..............................   5     0   seq
............................................................aaaccaucgcggacagacU..............................   1     1   seq
............................................................aaaccaucgcggacagacga.............................   2     0   seq
............................................................aaaccaucgcggacGgacgac............................   1     1   seq
............................................................aaaccaucgcggacagacgaU............................   1     1   seq
............................................................aaaccaucgcggacagacgac............................   17    0   seq
............................................................aaaccaucgcggacagacgaca...........................   2     0   seq
............................................................aaaccaucgcggacagacgacaUg.........................   94    1   seq
.............................................................aaccaucgcggacagacg..............................   4     0   seq
.............................................................aaccaucgcggacagacga.............................   1     0   seq
.............................................................aaccaucgcggacagacgac............................   4     0   seq
.............................................................aaccaucgcggacagacgacG...........................   1     1   seq
.............................................................aaccaucgcggacagacgaca...........................   33    0   seq
.............................................................aaccaucgcggacagacgacaUg.........................   83    1   seq
.............................................................aaccaucgcggacagacgacacg.........................   1     0   seq
.............................................................aaccaucgcggacagacgacaGg.........................   1     1   seq
..............................................................accaucgcggacagacg..............................   5     0   seq
..............................................................accaucgcggacagacgU.............................   1     1   seq
..............................................................accaucgcggacagacga.............................   1     0   seq
..............................................................accaucgcggacagacgaca...........................   4     0   seq
..............................................................accGucgcggacagacgacac..........................   1     1   seq
..............................................................accaucgcggacagacgacaU..........................   3     1   seq
..............................................................accaucgcggacagacgacacg.........................   4     0   seq
..............................................................accaucgcggacagacgacaUg.........................   270   1   seq
..............................................................accaucgcggacagacgacaGg.........................   2     1   seq
..............................................................accaucgcggacagacgacacA.........................   1     1   seq
...............................................................ccaucgcggacagacga.............................   112   0   seq
...............................................................ccaucgcggacagacgG.............................   5     1   seq
...............................................................ccaucgcggacagacgC.............................   1     1   seq
...............................................................cUaucgcggacagacga.............................   1     1   seq
...............................................................ccaucgcgAacagacga.............................   1     1   seq
...............................................................ccaCcgcggacagacga.............................   1     1   seq
...............................................................ccaucgcggacGgacga.............................   1     1   seq
...............................................................ccaucgcggacagacgU.............................   1     1   seq
...............................................................ccGucgcggacagacga.............................   1     1   seq
...............................................................ccaucgGggacagacgac............................   1     1   seq
...............................................................ccaucgcUgacagacgac............................   1     1   seq
...............................................................Gcaucgcggacagacgac............................   1     1   seq
...............................................................ccaucgcggacagUcgac............................   1     1   seq
...............................................................ccaucgcggacagacgaU............................   4     1   seq
...............................................................Acaucgcggacagacgac............................   1     1   seq
...............................................................ccaucgcggacagacgac............................   41    0   seq
...............................................................ccaucgcggGcagacgac............................   2     1   seq
...............................................................ccaucgcggaGagacgac............................   1     1   seq
```

```
                    Star                                        Mature
aaagaaagcgaagguggucuugacgucugguccacgaugguuuauuuaccauguaucaauaaaccaucgcggacagacgacacgagcaccuucaccuucguguacaugg
...............................................................ccaucgcggacagacgacG...........................   6      1   seq
...............................................................ccaucgcggacagacgaca...........................   138    0   seq
...............................................................ccaucgcggacagCcgaca...........................   1      1   seq
...............................................................ccaucgcggacagacgaUa...........................   1      1   seq
...............................................................ccaucgcggGcagacgaca...........................   1      1   seq
...............................................................ccaucgcggacagGcgaca...........................   1      1   seq
...............................................................ccaucgcggacagacgaAa...........................   1      1   seq
...............................................................ccaucgcgAacagacgacac..........................   1      1   seq
...............................................................ccaucgcggacagacgacaG..........................   9      1   seq
...............................................................ccaucgcggacagacgacaA..........................   2      1   seq
...............................................................ccaucgcggacagacgacac..........................   23     0   seq
...............................................................ccaucgcggacagacgacaU..........................   193    1   seq
...............................................................ccauGgcggacagacgacacg.........................   1      1   seq
...............................................................ccaucgcggacagacgacacC.........................   2      1   seq
...............................................................ccaucgcggacagacgacGcg.........................   1      1   seq
...............................................................ccaucgcggacagacgacacg.........................   126    0   seq
...............................................................cUaucgcggacagacgacacg.........................   1      1   seq
...............................................................ccaucgcggacagacgacaAg.........................   25     1   seq
...............................................................Ucaucgcggacagacgacacg.........................   1      1   seq
...............................................................ccaucgcggacagGcgacacg.........................   2      1   seq
...............................................................ccaucgcggacagacgacaGg.........................   72     1   seq
...............................................................ccaAcgcggacagacgacacg.........................   1      1   seq
...............................................................ccaucgcggacagacgacacA.........................   2      1   seq
...............................................................ccaucgcggacagacgacaUg.........................   18236  1   seq
...............................................................ccaucgcggacagacgacaUga........................   1      1   seq
...............................................................ccaucgcggacagacgacacCa........................   1      1   seq
...............................................................ccaucgcggacagacgacaUgag.......................   5      1   seq
...............................................................ccaucgcggacagacgacaUgagcaccuu.................   1      1   seq
................................................................caucgcggacagacgaca...........................   2      0   seq
................................................................caucgcggacagacgacaUg.........................   35     1   seq
.................................................................aucgcggacagacgaca...........................   2      0   seq
.................................................................aucgcggacagacgacaUg.........................   59     1   seq
.................................................................aucgcggacagacgacaUga........................   2      1   seq
..................................................................ucgcggacagacgacaUg.........................   5      1   seq
...................................................................cgcggacagacgacaUg.........................   2      1   seq
....................................................................gcggacagacgacaUgagcacc...................   1      1   seq
.....................................................................cggacagacgacaUgagcacc...................   5      1   seq
.....................................................................cggacagacgacaUgagcaccu..................   1      1   seq
```

Provisional ID : MA_13638_3678
Score total : 1.8
Score for star read(s) : -1.3
Score for read counts : 0
Score for mfe : 1.5
Score for randfold : 1.6
Score for cons. seed :
Total read count : 9023
Mature read count : 8817
Loop read count : 0
Star read count : 206

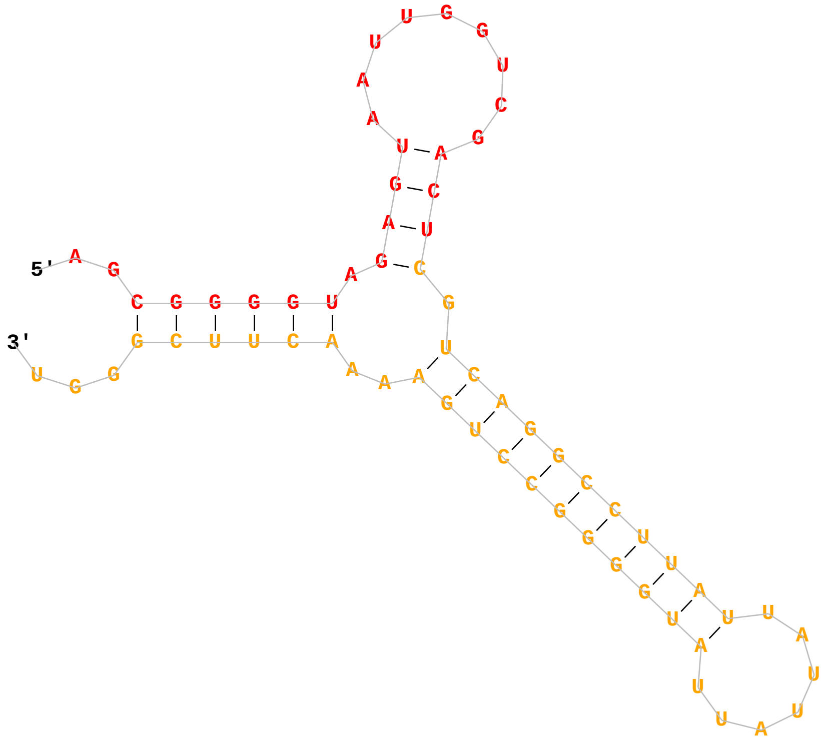

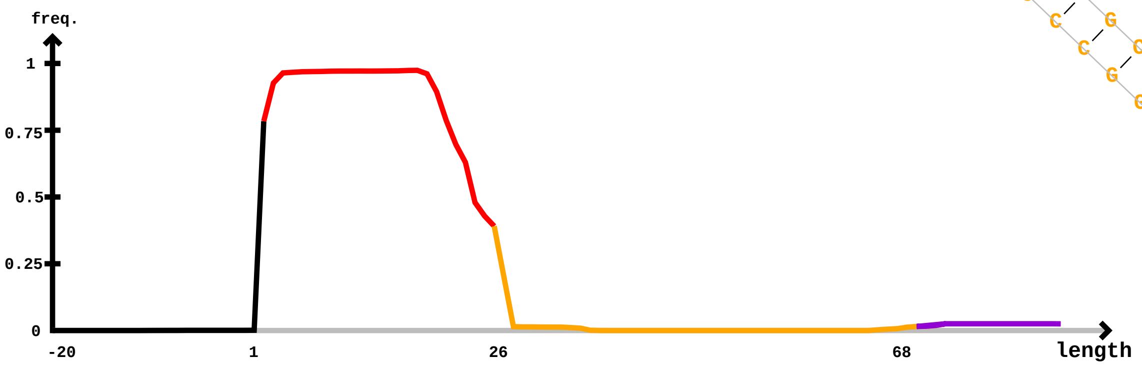

Mature Star

```
5'- acagggaaauaacgaacgauagcgggguagaguaauuggucgacucgucaggccuuauuauuauuauggggccugaaaacuucggguucgaauccugcccucgccucc -3'  obs
    acagggaaauaacgaacgauagcgggguagaguaauuggucgacucgucaggccuuauuauuauuauggggccugaaaacuucggguucgaauccugcccucgccucc      exp
    .....................(((((((((.(.......(((((((((((((((((((.......)))))))))))......)))).))))..))))))).))).... reads mm sample
    ........auaacgaacgauagcgggguaga............................................................................. 1 0 seq
    ........auaacgaacgauagcgggguagag............................................................................ 1 0 seq
    .........uaacgaacgauagcgggguagag............................................................................ 1 0 seq
    ..........aacgaacgauagcgggguagagua.......................................................................... 1 0 seq
    ............cgaacgauagcgggguagaguaauuggu.................................................................... 1 0 seq
    ............cgaacgauagcgggguagaguaauuggucgacucgucaggccCu.................................................... 1 1 seq
    ...................Gagcgggguagaguaauugguc................................................................... 1 1 seq
    ...................Cagcgggguagaguaauuggucg.................................................................. 2 1 seq
    ....................agcgggguagaguaauu....................................................................... 104 0 seq
    ....................agcgggguagCguaauu....................................................................... 1 1 seq
    ....................agcgggguagagCaauu....................................................................... 1 1 seq
    ....................agcggggCagaguaauu....................................................................... 2 1 seq
    ....................agcgggguagaguGauu....................................................................... 1 1 seq
    ....................agcgggguagaguaauA....................................................................... 1 1 seq
    ....................agcggggGagaguaauu....................................................................... 1 1 seq
    ....................agcgggguagaguaauC....................................................................... 9 1 seq
    ....................agcAggguagaguaauug...................................................................... 1 1 seq
    ....................agcgggguagGguaauug...................................................................... 3 1 seq
    ....................Ggcgggguagaguaauug...................................................................... 2 1 seq
    ....................agcgggguagaguaauuU...................................................................... 2 1 seq
    ....................agcggggCagaguaauug...................................................................... 2 1 seq
    ....................agcgggguagaguaauAg...................................................................... 1 1 seq
    ....................agcgggguagaguaauug...................................................................... 454 0 seq
    ....................agcggUguagaguaauug...................................................................... 1 1 seq
    ....................agcgggguagagCaauug...................................................................... 3 1 seq
    ....................agcggggGagaguaauug...................................................................... 1 1 seq
    ....................agcgggguagaguaauGg...................................................................... 1 1 seq
    ....................agcgggguagaguaGuug...................................................................... 2 1 seq
    ....................agcgggguagaguaaCug...................................................................... 2 1 seq
    ....................agcgggguagaguaauCg...................................................................... 2 1 seq
    ....................agcgggguagaguaauuA...................................................................... 13 1 seq
    ....................agcgggguagCguaauug...................................................................... 1 1 seq
    ....................agcgggguagaguaauugA..................................................................... 50 1 seq
```

Mature Star

acagggaaauaacgaacgauagcgggguagaguaauuggucgacucgucaggccuuauuauuauuauggggccugaaaacuucggguucgaauccugcccucgccucc			
....................agcgggUuagaguaauugg...	1	1	seq
....................aAcgggguagaguaauugg...	1	1	seq
....................Ngcgggguagaguaauugg...	2	1	seq
....................agcgggguagaguaauugg...	676	0	seq
....................agcgggguagGguaauugg...	5	1	seq
....................agcgggguGgaguaauugg...	1	1	seq
....................agcgggguagaAuaauugg...	1	1	seq
....................agUgggguagaguaauugg...	1	1	seq
....................agcgggguagaguaauCgg...	2	1	seq
....................agcgggguagaguGauugg...	2	1	seq
....................agcgggguagaguaauuAg...	2	1	seq
....................agcgggguagagGaauugg...	1	1	seq
....................agcgggguagagCaauugg...	1	1	seq
....................agcgUgguagaguaauugg...	2	1	seq
....................agcgggguagaguaaCugg...	3	1	seq
....................Ggcgggguagaguaauugg...	4	1	seq
....................agcgggguagaguaauugU...	6	1	seq
....................agcgggguagaguaGuugg...	4	1	seq
....................agcgggguagaguaauugC...	5	1	seq
....................agcggggGagaguaauugg...	3	1	seq
....................Cgcgggguagaguaauugg...	1	1	seq
....................agcgggguagCguaauugg...	1	1	seq
....................agcggggCagaguaauuggu..	3	1	seq
....................Ngcgggguagaguaauuggu..	1	1	seq
....................agcgggguagaguaauuAgu..	1	1	seq
....................agcggggAagaguaauuggu..	1	1	seq
....................agcgggguagaguaaGuggu..	1	1	seq
....................agcgggguGgaguaauuggu..	2	1	seq
....................agcgggguagaguaauugCu..	1	1	seq
....................agcgggguagaAuaauuggu..	1	1	seq
....................agcgggguagaguaauuggA..	6	1	seq
....................agcgggguagaguaauuggG..	14	1	seq
....................agcUggguagaguaauuggu..	1	1	seq
....................agcggggGagaguaauuggu..	1	1	seq
....................agcgggguagaguaauCggu..	1	1	seq
....................agcgggguagaguaauUggu..	1	1	seq
....................agcgggguagaguaauuggu..	514	0	seq
....................agcggUguagaguaauuggu..	1	1	seq
....................agcgggguagaUuaauuggu..	1	1	seq
....................agcgggguagaguaauuggC..	95	1	seq
....................agcgggguagaguaaCuggu..	1	1	seq
....................Ggcgggguagaguaauuggu..	2	1	seq
....................agcgggguagaguaauugguc...	441	0	seq
....................agcgggguagaguaauugguA...	7	1	seq
....................Ngcgggguagaguaauugguc...	1	1	seq
....................Ggcgggguagaguaauugguc...	1	1	seq
....................agcgggguagaguaGuugguc...	2	1	seq
....................agcgggguagaguaauugguG...	3	1	seq
....................agcggggCagaguaauugguc...	1	1	seq
....................agcggggGagaguaauugguc...	1	1	seq
....................agcgggguagGguaauugguc...	1	1	seq
....................agcgUgguagaguaauugguc...	1	1	seq
....................agcgggguagaguaauCgguc...	1	1	seq
....................agcgggguGgaguaauugguc...	1	1	seq
....................agcgggguagaguaauuAguc...	2	1	seq
....................agcgggguagaguaauuggCc...	3	1	seq
....................Ugcgggguagaguaauugguc...	2	1	seq
....................agcgggguagaguaUuugguc...	1	1	seq
....................agcgggguagaguaaCugguc...	2	1	seq
....................agcggggAagaguaauugguc...	2	1	seq
....................agcAggguagaguaauugguc...	1	1	seq
....................agcgggguagaguaauAgguc...	1	1	seq
....................Cgcgggguagaguaauuggucg..	1	1	seq
....................agcgggguagaguaauuggAcg..	2	1	seq
....................agcgggguagagGaauuggucg..	2	1	seq
....................agcgggguagGguaauuggucg..	6	1	seq
....................agcgggguagagCaauuggucg..	3	1	seq
....................agUgggguagaguaauuggucg..	4	1	seq
....................agcgggguagaguaauuggucU..	12	1	seq
....................agcgggguagaguaauAggucg..	1	1	seq

Mature   Star

```
acagggaaauaacgaacgauagcgggguagaguaauuggucgacucgucaggccuuauuauuauuauggggccugaaaacuucggguucgaauccugcccucgccucc
....................agcgggguagagAaauuggucg..................................................................	3	1	seq
....................agcgggguagaguaGuuggucg..................................................................	2	1	seq
....................agcggAguagaguaauuggucg..................................................................	1	1	seq
....................agcgggguagaguaauuggucC..................................................................	10	1	seq
....................agcgggguagaguaauuggCcg..................................................................	10	1	seq
....................agAgggguagaguaauuggucg..................................................................	1	1	seq
....................agcgggguagaguGauuggucg..................................................................	1	1	seq
....................Ugcgggguagaguaauuggucg..................................................................	2	1	seq
....................agcgggguagaguaauuggucg..................................................................	908	0	seq
....................agcggggAagaguaauuggucg..................................................................	1	1	seq
....................agcgggguagaguaauuUgucg..................................................................	1	1	seq
....................Ngcgggguagaguaauuggucg..................................................................	1	1	seq
....................agcggggGagaguaauuggucg..................................................................	3	1	seq
....................agcgggguGgaguaauuggucg..................................................................	1	1	seq
....................agcgggguagaguaauuAgucg..................................................................	4	1	seq
....................agcggggCagaguaauuggucg..................................................................	6	1	seq
....................agcgggguagaguaauuggGcg..................................................................	3	1	seq
....................agcgggguagaguaauugguAg..................................................................	2	1	seq
....................agcgggAuagaguaauuggucg..................................................................	1	1	seq
....................Ggcgggguagaguaauuggucg..................................................................	4	1	seq
....................agcgggguagaguaauuggCcga.................................................................	2	1	seq
....................agcgggguagagAaauuggucga.................................................................	1	1	seq
....................aAcgggguagaguaauuggucga.................................................................	1	1	seq
....................agAgggguagaguaauuggucga.................................................................	1	1	seq
....................Ggcgggguagaguaauuggucga.................................................................	1	1	seq
....................agcgUgguagaguaauuggucga.................................................................	1	1	seq
....................agcgggguagaguaauuggucga.................................................................	299	0	seq
....................agcggggCagaguaauuggucga.................................................................	1	1	seq
....................agcgggguagaguaauuggucgU.................................................................	4	1	seq
....................agcgggguagaguaauuggucgC.................................................................	1	1	seq
....................agcgggguagaguaauuggucgG.................................................................	7	1	seq
....................agcggggCagaguaauuggucgac................................................................	2	1	seq
....................agcgggguagaguaauuggucgac................................................................	231	0	seq
....................agcgggguagaguaauuggucgaG................................................................	3	1	seq
....................agcUggguagaguaauuggucgac................................................................	1	1	seq
....................agGgggguagaguaauuggucgac................................................................	1	1	seq
....................agcggAguagaguaauuggucgac................................................................	1	1	seq
....................agcgggguagaAuaauuggucgac................................................................	2	1	seq
....................agcgggguagaguaauuggucgGc................................................................	1	1	seq
....................Ggcgggguagaguaauuggucgac................................................................	2	1	seq
....................agcgggguagaguGauuggucgac................................................................	2	1	seq
....................agcgggguGgaguaauuggucgac................................................................	1	1	seq
....................agcgggguagaguaauuggucgaU................................................................	13	1	seq
....................agcgggguagaguaauuggucgaA................................................................	1	1	seq
....................agcgggCuagaguaauuggucgac................................................................	1	1	seq
....................agcgggguagaguaaCuggucgacu...............................................................	2	1	seq
....................agcggggCagaguaauuggucgacu...............................................................	6	1	seq
....................agcgggguagaguaauuggGcgacu...............................................................	1	1	seq
....................aNcgggguagaguaauuggucgacu...............................................................	1	1	seq
....................agcgggguagaguaaAuggucgacu...............................................................	1	1	seq
....................agcgggguUgaguaauuggucgacu...............................................................	1	1	seq
....................agcgggguagaguaauuggucgacA...............................................................	67	1	seq
....................agcgggguagagAaauuggucgacu...............................................................	1	1	seq
....................agcgggguagaguaauuggucgacC...............................................................	329	1	seq
....................agcgggguagagCaauuggucgacu...............................................................	4	1	seq
....................agcgggguagaguaCuuggucgacu...............................................................	1	1	seq
....................agcgggguGgaguaauuggucgacu...............................................................	2	1	seq
....................agcgggguagaguaauuggucgaGu...............................................................	1	1	seq
....................agcgggguagaguaUuuggucgacu...............................................................	1	1	seq
....................agcgggguagUguaauuggucgacu...............................................................	1	1	seq
....................agcgggguagaguaauugCucgacu...............................................................	1	1	seq
....................agUgggguagaguaauuggucgacu...............................................................	1	1	seq
....................agcgggguagaguaGuuggucgacu...............................................................	3	1	seq
....................agcgggguagaguGauuggucgacu...............................................................	3	1	seq
....................agcgggguagaguaauugguAgacu...............................................................	1	1	seq
....................agcAggguagaguaauuggucgacu...............................................................	3	1	seq
....................agcgggguagaguaauuggucgacG...............................................................	62	1	seq
....................Ugcgggguagaguaauuggucgacu...............................................................	1	1	seq
....................agcgggguagaguaauuggAcgacu...............................................................	2	1	seq
....................Ngcgggguagaguaauuggucgacu...............................................................	5	1	seq
```

```
                    Mature                                                                Star
acagggaaauaacgaacgauagcgggguagaguaauuggucgacucgucaggccuuauuauuauuauggggccugaaaacuucggguucgaauccugcccucgccucc
....................agcgggguagCguaauuggucgacu...............................................................   1      1     seq
....................agcgggguagGguaauuggucgacu...............................................................   8      1     seq
....................agcggggGagaguaauuggucgacu...............................................................   2      1     seq
....................aUcgggguagaguaauuggucgacu...............................................................   1      1     seq
....................agcgggguagaguaauuAgucgacu...............................................................   2      1     seq
....................agcggggAagaguaauuggucgacu...............................................................   1      1     seq
....................agcgggguagaguaauuggucgGcu...............................................................   11     1     seq
....................agcgAgguagaguaauuggucgacu...............................................................   1      1     seq
....................agcgggguagaguaauuggCcgacu...............................................................   4      1     seq
....................aAcgggguagaguaauuggucgacu...............................................................   1      1     seq
....................agcgggguagagGaauuggucgacu...............................................................   1      1     seq
....................agcgggguagaguaauCggucgacu...............................................................   7      1     seq
....................agcgggguagaguaauuggucgacu...............................................................   1853   0     seq
....................Ggcgggguagaguaauuggucgacu...............................................................   8      1     seq
....................agcgUgguagaguaauuggucgacu...............................................................   1      1     seq
....................agcgggguagaguaauuggucgaGuc..............................................................   1      1     seq
....................agcgggguagaguaauuggucgacuU..............................................................   29     1     seq
....................agcgggguagaguaauuggucgacuc..............................................................   536    0     seq
....................agcgggguagagCaauuggucgacuc..............................................................   1      1     seq
....................agcgggguagaAuaauuggucgacuc..............................................................   1      1     seq
....................agcgggguGgaguaauuggucgacuc..............................................................   1      1     seq
....................agcgggguagaguaauuggCcgacuc..............................................................   3      1     seq
....................agcgggguagaguaaCuggucgacuc..............................................................   2      1     seq
....................agcgggguagaguaauuggucUacuc..............................................................   1      1     seq
....................agAgggguagaguaauuggucgacuc..............................................................   1      1     seq
....................agUgggguagaguaauuggucgacuc..............................................................   1      1     seq
....................aAcgggguagaguaauuggucgacuc..............................................................   1      1     seq
....................Ggcgggguagaguaauuggucgacuc..............................................................   1      1     seq
....................agcgggguagaguaGuuggucgacuc..............................................................   2      1     seq
....................agcgggguagaguaauugAucgacuc..............................................................   1      1     seq
....................agcggggGagaguaauuggucgacuc..............................................................   1      1     seq
....................agcgggguagGguaauuggucgacuc..............................................................   1      1     seq
....................agcgggguagaguaauuggucgacuA..............................................................   11     1     seq
....................agcAggguagaguaauuggucgacuc..............................................................   2      1     seq
....................agcgggguagaguGauuggucgacuc..............................................................   3      1     seq
....................agcgggguagaguaauuggucgacCc..............................................................   7      1     seq
....................agcggggCagaguaauuggucgacuc..............................................................   1      1     seq
....................agcgggguUgaguaauuggucgacuc..............................................................   1      1     seq
....................agcgggguagaguaauuggucgacucA.............................................................   7      1     seq
....................agcgggguagaguaauuggucgacucAuc...........................................................   1      1     seq
....................agcgggguagaguaauuggucgacucguc...........................................................   2      0     seq
....................agcgggguagaguaauuggucgacucUuc...........................................................   1      1     seq
....................agcgggguagaguaauuggucgacucgCca..........................................................   1      1     seq
....................agcgggguagaguaauuggucgacucAucagg........................................................   2      1     seq
....................agcgggguagaguaauuggucgacucgucagg........................................................   1      0     seq
....................agcgggguagaguaauuggucgacucUucagg........................................................   3      1     seq
....................agcgggguagaguaauuggucgacucCucagg........................................................   1      1     seq
....................agcgggguagaguaauuggucgacucgucagC........................................................   1      1     seq
....................agcgggguagGguaauuggucgacucgucaggc.......................................................   1      1     seq
....................agcgggguagaguaauuggucgacucgucaggc.......................................................   4      0     seq
....................agcgggguagaguaauuggucgacucCucaggc.......................................................   1      1     seq
....................agcgggguagaguaauuggucgacucgucaggU.......................................................   1      1     seq
....................agcgggguagaguGauuggucgacucgucaggc.......................................................   1      1     seq
....................agcgggguagaguaauuggucgacucUucaggc.......................................................   1      1     seq
....................agcgggguagaguaauuggucgacucCucaggcc......................................................   6      1     seq
....................agcgggguagaguaauuggucgacucAucaggcc......................................................   2      1     seq
....................agcgggguagaguaauuggucgacucUucaggcc......................................................   11     1     seq
....................agcgggguagaguaauuggucgacucgucaggcA......................................................   3      1     seq
....................agcgggguagaguaauuggucgacucgucaggcc......................................................   19     0     seq
....................agcgggguagaguaauuggucgacucgucaggcU......................................................   8      1     seq
....................agcgggguagaguaauuggucgacucgucaggccu.....................................................   2      0     seq
.....................gcgggguagaguaauug......................................................................   109    0     seq
.....................gcgggguagaguaauCg......................................................................   3      1     seq
.....................gcgggguagaguaauuA......................................................................   3      1     seq
.....................gcggggAagaguaauug......................................................................   2      1     seq
.....................gcAgggguagaguaauug.....................................................................   1      1     seq
.....................gcggggCagaguaauug......................................................................   1      1     seq
.....................gcgggguagaguGauug......................................................................   2      1     seq
.....................Ccgggguagaguaauug......................................................................   1      1     seq
.....................Acgggguagaguaauug......................................................................   1      1     seq
```

```
                  Mature                                                     Star
acagggaaauaacgaacgauagcgggguagaguaauuggucgacucgucaggccuuauuauuauuauggggccugaaaacuucggguucgaauccugcccucgccucc
.....................gcgggguagaguaauugC.....................................................................   1     1     seq
.....................gcgggguaUaguaauugg.....................................................................   1     1     seq
.....................gUgggguagaguaauugg.....................................................................   1     1     seq
.....................gcgggguagaguaauugA.....................................................................   11    1     seq
.....................gcgggguagaguaauugU.....................................................................   2     1     seq
.....................gcggAguagaguaauugg.....................................................................   1     1     seq
.....................gcgggguagGguaauugg.....................................................................   1     1     seq
.....................gcgggguagaguaauugg.....................................................................   133   0     seq
.....................gcgggguagaguaauuCg.....................................................................   1     1     seq
.....................gcgggguagaguaauuggG....................................................................   1     1     seq
.....................gcAggguagaguaauuggu....................................................................   1     1     seq
.....................gcgggguagGguaauuggu....................................................................   1     1     seq
.....................gcgggguagaguaauuggA....................................................................   2     1     seq
.....................gcgggguagaguaauuggu....................................................................   110   0     seq
.....................gcgggguagaguGauuggu....................................................................   1     1     seq
.....................gcgggguagaguaauuggC....................................................................   12    1     seq
.....................Acgggguagaguaauugguc...................................................................   1     1     seq
.....................gcgggguagGguaauugguc...................................................................   1     1     seq
.....................gcgggguagaguaauugguc...................................................................   84    0     seq
.....................gcgggguagaguaauugguA...................................................................   3     1     seq
.....................gcggggCagaguaauugguc...................................................................   1     1     seq
.....................gcgggguagaguaaCugguc...................................................................   1     1     seq
.....................gcgggguagaguaauuUgucg..................................................................   1     1     seq
.....................gUgggguagaguaauuggucg..................................................................   1     1     seq
.....................gcgggguagaguaauuggucg..................................................................   251   0     seq
.....................gcgggguagGguaauuggucg..................................................................   1     1     seq
.....................gcgggguGgaguaauuggucg..................................................................   1     1     seq
.....................gcgggguagaguaauuggucU..................................................................   5     1     seq
.....................gcgggguagagAaauuggucg..................................................................   1     1     seq
.....................gcgggguagaguaaCuggucg..................................................................   1     1     seq
.....................gcggggCagaguaauuggucg..................................................................   1     1     seq
.....................gcgggCuagaguaauuggucg..................................................................   1     1     seq
.....................gcgggguagaguaauCggucg..................................................................   1     1     seq
.....................gcgggguagaguaaGuggucg..................................................................   1     1     seq
.....................gcAggguagaguaauuggucg..................................................................   1     1     seq
.....................gcggggGagaguaauuggucg..................................................................   1     1     seq
.....................gcgggguagaguaauuAgucg..................................................................   1     1     seq
.....................gcgggguagaguaauuggCcg..................................................................   1     1     seq
.....................gcgggguagaguaUuuggucga.................................................................   1     1     seq
.....................gcgggguagaguaauuggucgG.................................................................   1     1     seq
.....................gcgggguagaguaauuggucga.................................................................   98    0     seq
.....................Ncgggguagaguaauuggucga.................................................................   1     1     seq
.....................Ucgggguagaguaauuggucga.................................................................   2     1     seq
.....................gcgggguagagCaauuggucga.................................................................   1     1     seq
.....................gcgggguGgaguaauuggucga.................................................................   1     1     seq
.....................gcggggCagaguaauuggucga.................................................................   1     1     seq
.....................gcgggguagaguaauuggucgaU................................................................   8     1     seq
.....................gcgggguagaguaauCggucgac................................................................   1     1     seq
.....................gcgggguagagCaauuggucgac................................................................   1     1     seq
.....................gcgggguagaguaauuggucgaA................................................................   1     1     seq
.....................gcgggguagaguaauuggucgac................................................................   48    0     seq
.....................gcgggguGgaguaauuggucgacu...............................................................   1     1     seq
.....................gcgggguagaguaauuggucgacu...............................................................   234   0     seq
.....................gcgggguagGguaauuggucgacu...............................................................   1     1     seq
.....................gcgggguagaguaauuggucgCcu...............................................................   1     1     seq
.....................gcggggAagaguaauuggucgacu...............................................................   1     1     seq
.....................gcgggguagaAuaauuggucgacu...............................................................   1     1     seq
.....................gcgggguagaguaauuggucgacG...............................................................   7     1     seq
.....................gUgggguagaguaauuggucgacu...............................................................   1     1     seq
.....................gcgggguagaguaauuggucgaUu...............................................................   2     1     seq
.....................gcgggguagaguaauuggucgacA...............................................................   10    1     seq
.....................Ccgggguagaguaauuggucgacu...............................................................   1     1     seq
.....................gcgggguagaguaauuggucgacC...............................................................   50    1     seq
.....................gcgggguagaguaauCggucgacu...............................................................   1     1     seq
.....................gcgAgguagaguaauuggucgacu...............................................................   1     1     seq
.....................gcAggguagaguaauuggucgacu...............................................................   1     1     seq
.....................gcgggguagaguaauuggAcgacu...............................................................   1     1     seq
.....................gcgggguagaguGauuggucgacuc..............................................................   1     1     seq
.....................gcgggguagaguaauuggucgacuA..............................................................   2     1     seq
.....................gcgggguagaguaauuggucgacuU..............................................................   2     1     seq
```

```
                    Mature                                                Star
acagggaaauaacgaacgauagcgggguagaguaauuggucgacucgucaggccuuauuauuauuauggggccugaaaacuucggguucgaauccugcccucgccucc
.....................gcgggguagGguaauuggucgacuc..............................................................	1	1	seq
.....................gcgggguagaguaauuggucgacuc..............................................................	43	0	seq
.....................gcgggguagaguaauuggucgacucAuc...........................................................	1	1	seq
.....................gcgggguagaguaauuggucgacucUucagg.......................................................	1	1	seq
.....................gcgggguagaguaauuggucgacucAucaggcc.....................................................	1	1	seq
.....................gcgggguagaguaauuggucgacucCucaggcc.....................................................	7	1	seq
.....................gcgggguagaguaauuggucgacucUucaggcc.....................................................	5	1	seq
.....................gcgggguagaguaauuggucgacucgucaggcc.....................................................	4	0	seq
......................cgggguagaguGauugg.....................................................................	1	1	seq
......................cgggguagaguaauugg.....................................................................	54	0	seq
......................cgggguagaguaauugA.....................................................................	3	1	seq
......................Ggggguagaguaauugg.....................................................................	1	1	seq
......................cgggguagaguaauuggC....................................................................	7	1	seq
......................cgggguagaguaauuggu....................................................................	35	0	seq
......................cgggguagaguaauuggG....................................................................	1	1	seq
......................cgggguagaguaauugguc...................................................................	37	0	seq
......................cgggguagGguaauugguc...................................................................	2	1	seq
......................cgggguagaguaauCgguc...................................................................	1	1	seq
......................cgggguagaguaauuggucU..................................................................	1	1	seq
......................cgggguagaguaauugguGg..................................................................	1	1	seq
......................cgggguagaguaauuggucg..................................................................	92	0	seq
......................cgggguagaguaauuAgucg..................................................................	2	1	seq
......................cAggguagaguaauuggucg..................................................................	1	1	seq
......................cgggguagaguaauuggucC..................................................................	1	1	seq
......................cgggguagGguaauuggucg..................................................................	1	1	seq
......................Ugggguagaguaauuggucg..................................................................	1	1	seq
......................cgggguagaguaauuggucgU.................................................................	1	1	seq
......................cgggguagaguaGuuggucga.................................................................	1	1	seq
......................cgggguagaguaauuggucga.................................................................	26	0	seq
......................cggAguagaguaauuggucgac................................................................	1	1	seq
......................cgggguagaguaauuggucgac................................................................	19	0	seq
......................cgggguagaguaGuuggucgac................................................................	1	1	seq
......................cgggguagaguaauuggucgacA...............................................................	1	1	seq
......................cgggguagaguaauuggucgacu...............................................................	39	0	seq
......................cgggguagaguaauuggucgacG...............................................................	1	1	seq
......................cgggguagaguaauuggucgacC...............................................................	6	1	seq
......................cgggguagaguaauuggucgacuc..............................................................	7	0	seq
......................cgggguagaguaauuggucgacucAucaggcc.....................................................	1	1	seq
......................cgggguagaguaauuggucgacucUucaggcc.....................................................	1	1	seq
......................cgggguagaguaauuggucgacucgucaggcc.....................................................	1	0	seq
.......................gggguagaguaauuggC....................................................................	1	1	seq
.......................gggguagaguaauuggu....................................................................	1	0	seq
.......................gggguagaguaauugguc...................................................................	4	0	seq
.......................gggguagaguaauuggucg..................................................................	7	0	seq
.......................gggguagaguaauuggucga.................................................................	1	0	seq
.......................gggguagaguaauuggucgac................................................................	5	0	seq
.......................Cggguagaguaauuggucgacu...............................................................	1	1	seq
.......................gggguagaguaauuggucgacu...............................................................	1	0	seq
........................ggguagaguaauugguc...................................................................	4	0	seq
........................ggguagaguaauugguA...................................................................	1	1	seq
........................ggguagaguaauuggCc...................................................................	1	1	seq
........................ggguagaguaauuggucg..................................................................	4	0	seq
........................ggguagaguaauuggucga.................................................................	5	0	seq
........................ggguagaguaauuggucgU.................................................................	1	1	seq
........................ggguagaguaauuggucgac................................................................	1	0	seq
........................ggguagaguaauuggucgaU................................................................	1	1	seq
........................ggguagaguaauuggucgacu...............................................................	2	0	seq
.........................gguagaguaauuggucg..................................................................	1	0	seq
.........................gguagaguaauuggucga.................................................................	3	0	seq
.........................gguagaguaauuggucgacuc..............................................................	1	0	seq
..........................guagaguaauuggucga.................................................................	1	0	seq
..........................guagaguaauuggucgacC...............................................................	1	1	seq
..........................guagaguaauuggucgacu...............................................................	2	0	seq
..........................guagaguaauuggucgacG...............................................................	1	1	seq
...........................uagaguaauuggucgacC...............................................................	1	1	seq
...........................uagaguaauuggucgacu...............................................................	7	0	seq
...........................uagaguaauuggucgacG...............................................................	1	1	seq
............................agaguaauuggucgacu...............................................................	3	0	seq
............................agaguaauuggucgacucgucagg........................................................	1	0	seq
..............................aguaauuggucgacucU.............................................................	1	1	seq
```

Mature **Star**

acagggaaauaacgaacgauagcggguagaguaauuggucgacucgucaggccuuauuauuauuauggggccugaaaacuucggguucgaauccugcccucgccucc			
...............................uaauuggucgacucUuc...	1	1	seq
...............................uaauuggucgacucgucagg..	1	0	seq
...............................uaauuggucgacucAucaggccu...	1	1	seq
................................aauuggucgacucUucag...	1	1	seq
................................aauuggucgacucgucagg..	1	0	seq
................................aauuggucgacucUucaggc...	1	1	seq
................................aauuggucgacucgucaggcU..	1	1	seq
.................................auuggucgacucUucaggc...	2	1	seq
.................................auuggucgacucgucaggcc..	1	0	seq
.................................auuggucgacucgucaggccA...	1	1	seq
..................................uuggucgacucUucagg..	3	1	seq
..................................uuggucgacucAucagg..	1	1	seq
..................................uuggucgacucgucagg..	1	0	seq
..................................uuggucgacucUucaggc...	3	1	seq
..................................uuggucgacucCucaggc...	1	1	seq
..................................uuggucgacucgucaggc...	1	0	seq
..................................uuggucgacucAucaggc...	1	1	seq
..................................uuggucgacucgucaggccu...	2	0	seq
...................................uggucgacucAucaggc...	1	1	seq
...................................uggucgacucUucaggc...	1	1	seq
...................................uggucgacucgucaggccu...	1	0	seq
...................................uggucgacucgucaggccA...	1	1	seq
....gguucgaauccugcccCcg.....	2	1	seq
....gguucgaauccugcccCcgcc...	3	1	seq
....gguucgaauccugcccCcgccu..	4	1	seq
....gguucgaauccugcccCcgccuc.	12	1	seq
....gguucgaauccugcccCcgccucc	5	1	seq
....guucgaauccugcccCcgcc...	6	1	seq
....guucgaauccugcccCcgccu..	9	1	seq
....guucgaauccugcccCcgccuc.	3	1	seq
....guucgaauccugcccCcgccucc	3	1	seq
....uucgaauccugcccCcg.....	1	1	seq
....uucgaauccugcccCcgc....	1	1	seq
....uucgaauccugcccCcgcc...	4	1	seq
....uucgaauccugcccCcgccu..	4	1	seq
....uucgaauccugcccCcgccuc.	6	1	seq
....uucgaauccugcccCcgccucc	1	1	seq
....ucgaauccugcccCcgc....	5	1	seq
....ucgaauccugcccCcgcc...	17	1	seq
....ucgaauccugcccCcgccu..	14	1	seq
....ucgaauccugcccCcgccuc.	9	1	seq
....ucgaauccugcccCcgccucc	6	1	seq
....cgaauccugcccCcgcc...	6	1	seq
....cgaauccugcccCcgccu..	7	1	seq
....cgaauccugcccCcgccuc.	5	1	seq
....cgaauccugcccCcgccucc	6	1	seq
....gaauccugcccCcgccu..	3	1	seq
....gaauccugcccCcgccuc.	3	1	seq
....gaauccugcccCcgccucc	3	1	seq
....aauccugcccCcgccucc	27	1	seq
....auccugcccCcgccucc	57	1	seq

Provisional ID	:	MA_137623_24241
Score total	:	0
Score for star read(s)	:	-1.3
Score for read counts	:	-1.8
Score for mfe	:	1.6
Score for randfold	:	1.6
Score for cons. seed	:	
Total read count	:	8
Mature read count	:	8
Loop read count	:	0
Star read count	:	0

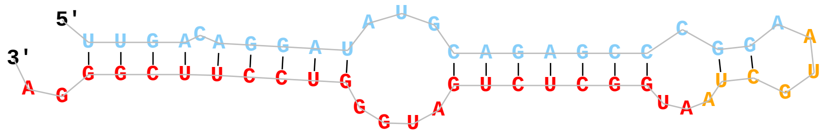

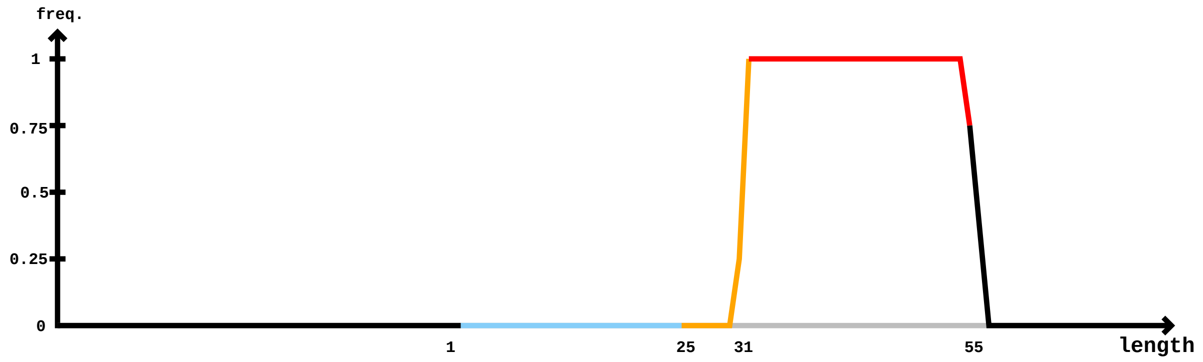


```
                                        Star                    Mature
5'- uucuauuaguagcagugaaacccagacuguucuucagggcuugacaggauaugcagagcccggaaugcuaauggcucugauggguccuucgggaucugggaaaucgacgcuucu   -3'   exp
    .......((.(((..(((..(((((((((.....)))..(((((.(((((...(((((((.((....))...)))))))....))))))))))).))))))...)))..))).))    reads   mm   sample
    .....................................................................aauggcucugauggguccuucAgg.....................    1       1    seq
    .....................................................................aauggcucugauggguccuucggg.....................    1       0    seq
    ......................................................................auCgcucugauggguccuucggga....................    1       1    seq
    ......................................................................auggcucugauggguccuuUggga....................    1       1    seq
    ......................................................................auggcucugauggguccuucggga....................    4       0    seq
```

Provisional ID	: MA_137893_24457
Score total	: 0.7
Score for star read(s)	: -1.3
Score for read counts	: 0
Score for mfe	: 0.4
Score for randfold	: 1.6
Score for cons. seed	:
Total read count	: 22
Mature read count	: 22
Loop read count	: 0
Star read count	: 0

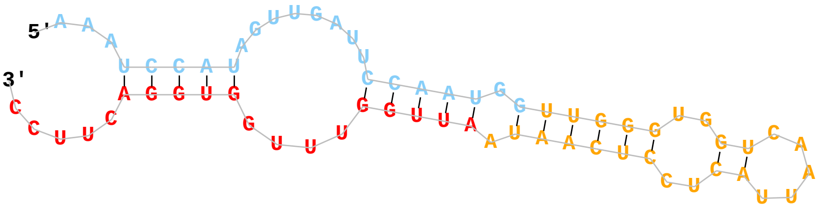

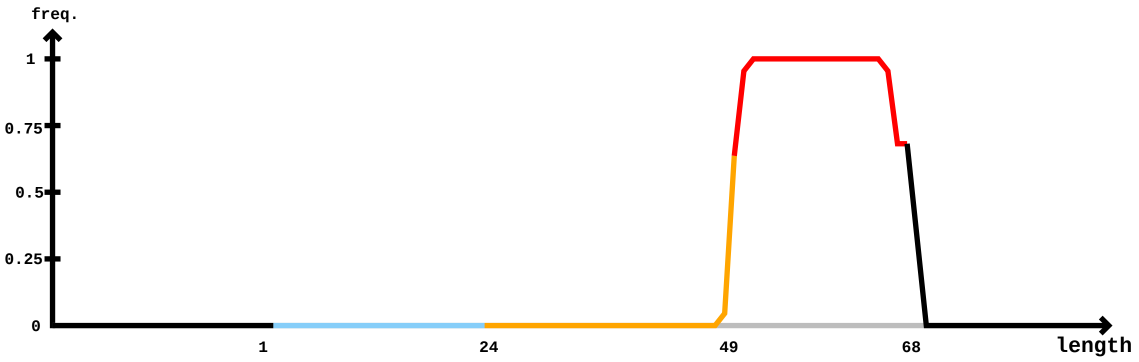

Star Mature

```
5'- uaaugguuuggauuuggggugaaauccauaguugauuccaaugguugggugguucaauuacuccucaauaauugguuugguggacuucccuucauuuacagauaagugu   -3'    exp
    .....(((((....(((((.(((.(((((........(((((.((((((..((.....))..)))))).)))))....))))).))).)))))....))))).....    reads   mm    sample
    ....................................................................aUuugguuugguggacu.......................    1       1     seq
    .....................................................................Uuugguuugguggacuu......................    5       1     seq
    .....................................................................Cuugguuugguggacuu......................    1       1     seq
    .....................................................................Uuugguuugguggacuucc....................    7       1     seq
    ......................................................................uugguuugguggacuucc....................    5       0     seq
    ......................................................................uugguuugguggacuuccA...................    2       1     seq
    .......................................................................ugguuugguggacuuccA...................    1       1     seq
```

Provisional ID	:	MA_138034_23715
Score total	:	0.4
Score for star read(s)	:	-1.3
Score for read counts	:	0
Score for mfe	:	0.1
Score for randfold	:	1.6
Score for cons. seed	:	
Total read count	:	20
Mature read count	:	20
Loop read count	:	0
Star read count	:	0

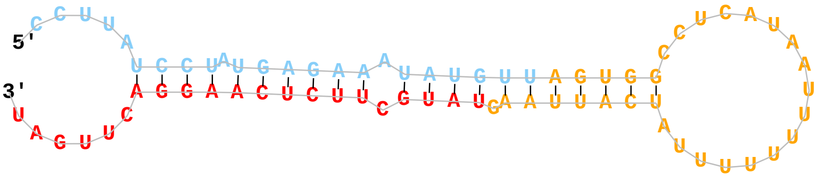

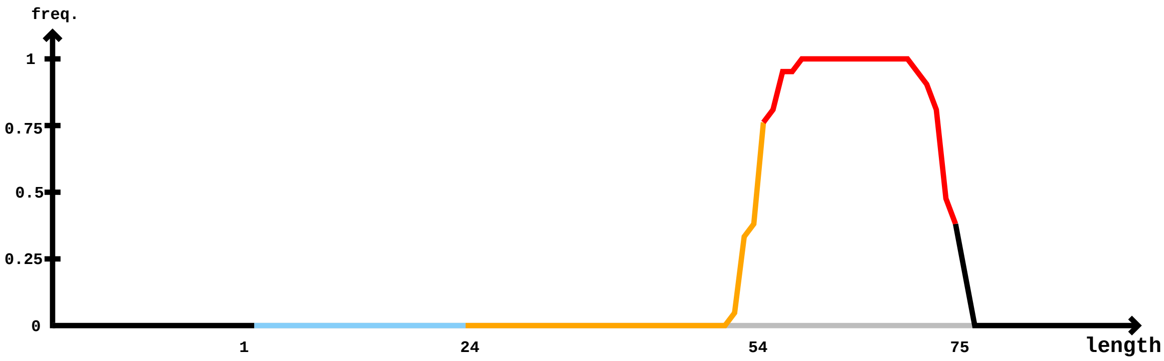

Star Mature

```
5'- cauuaccucuggaggguguccuuauccuaugagaaauauguuaguggccucauaauuuuuuuuaucauuaaguaugcuucucaaggacuugauauugucgauggggaaaau   -3'   exp
    .....(((((.((.((((((....((((.((((((.(((((((((((.................))))))).)))).))))))))))...)))))).)).).)))).....   reads   mm   sample
    .....................................................................Gaguaugcuucucaaggac.......................   1   1   seq
    ......................................................................aguaugcuucucaaggacuu.....................   1   0   seq
    ......................................................................aguaugcuucucaaggacuug....................   4   0   seq
    ......................................................................aguaugcuucuUaaggacuug....................   1   1   seq
    .......................................................................guaugcuucucaaggacuugG...................   1   1   seq
    ........................................................................uaugcuucucaaggacu......................   1   0   seq
    ........................................................................uaugcuucucaaggacuu.....................   1   0   seq
    ........................................................................uaugcuucucaaggacuugGu..................   6   1   seq
    .........................................................................augcuucucaaggacuug....................   1   0   seq
    ..........................................................................ugcuucucaaggacuug....................   1   0   seq
    ..........................................................................ugcuucucaaggacuugG...................   1   1   seq
    ..........................................................................ugcuucucaaggacuugGu..................   1   1   seq
    ............................................................................cuucucaaggacuugGu..................   1   1   seq
```

Provisional ID	:	MA_13826_5055
Score total	:	1.1
Score for star read(s)	:	-1.3
Score for read counts	:	-1.3
Score for mfe	:	2.2
Score for randfold	:	1.6
Score for cons. seed	:	
Total read count	:	9
Mature read count	:	9
Loop read count	:	0
Star read count	:	0

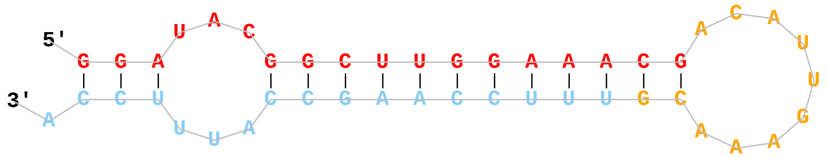

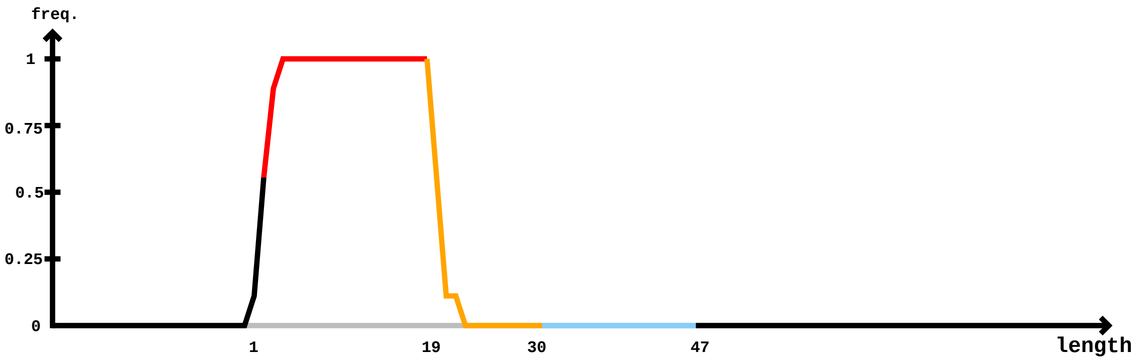

Mature Star

```
5'- uucggaaacuaaaauggaggggauacggcuuggaaacgacauugaaacguuuccaagccauuuccaaccauuucaguuucugaaauagggaauggcguauccccccuu   -3'    exp
    ((((((((((.((((((..((((...((((((((((((.........))))))))))))..))))..)))))).))))))))))...((((........)))).....    reads     mm      sample
    ...................gggauacggcuuggaaacgGca...................................................................    1         1       seq
    ....................ggauacggcuuggaaacg......................................................................    4         0       seq
    .....................gauacggcuuggaaacg......................................................................    1         0       seq
    .....................gauacgUcuuggaaacga.....................................................................    1         1       seq
    .....................gauacggcuuggaaacgG.....................................................................    1         1       seq
    ......................auacggcuuggaaacgG.....................................................................    1         1       seq
```

Provisional ID	:	MA_138737_24341
Score total	:	19384.8
Score for star read(s)	:	3.9
Score for read counts	:	19377
Score for mfe	:	2.2
Score for randfold	:	1.6
Score for cons. seed	:	
Total read count	:	38019
Mature read count	:	37980
Loop read count	:	0
Star read count	:	39

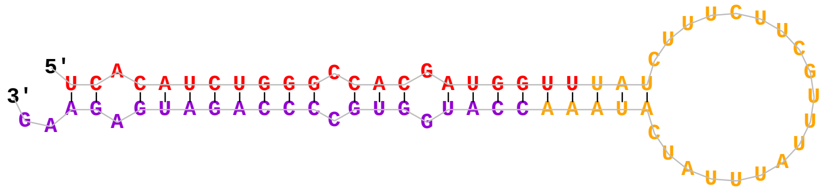

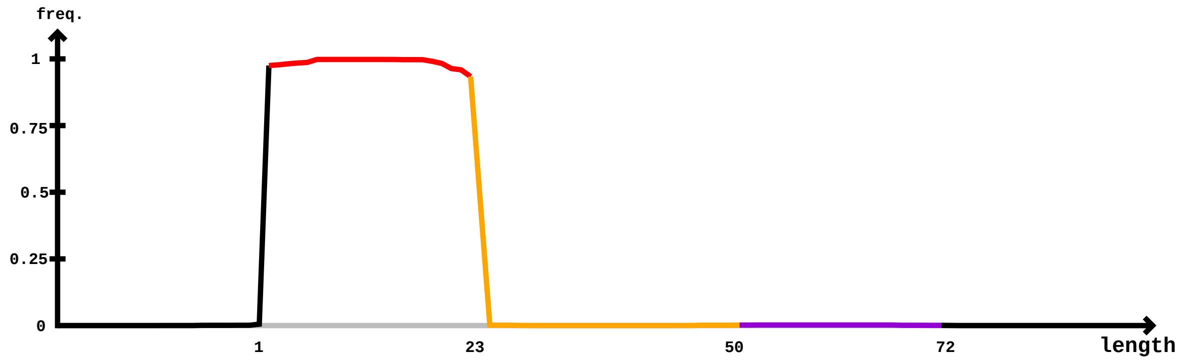

Mature **Star**

```
5'- agagguuuuggaggugaucuucacaucugggccacgaugguuuaucuuucuucguuuauuuaucauaaaccauggugccccagaugagaagagcaccuucgccuucguuuau -3'   obs
    agagguuuuggaggugaucuucacaucugggccacgaugguuuaucuuucuucguuuauuuaucauaaaccauggugccccagaugagaagagcaccuucgccuucguuuau        exp
    .(((((...(((((((.(((((.((((((((.(((.(((((((((...................))))))))).))).)))))))).))))).))))))))))))).......   reads   mm   sample
    .gagguuuGggaggugaucu............................................................................................   1       1    seq
    .........ggaggugaucuuGacaucugggcca..............................................................................   1       1    seq
    ............ggugaucuuGacaucugggccacgaugguu......................................................................   1       1    seq
    .............gugaucuuGacaucugggcc...............................................................................   5       1    seq
    .............gugaucuuAacaucugggcca..............................................................................   1       1    seq
    .............gugaucuuGacaucugggcca..............................................................................   20      1    seq
    ..............ugaucuuGacaucugggccac.............................................................................   1       1    seq
    ..............ugaucuuGacaucugggccacg............................................................................   3       1    seq
    .................ucuucacaucuggCccacg............................................................................   1       1    seq
    .................ucuuUacaucugggccacga...........................................................................   1       1    seq
    .................ucuucacaucuggCccacgaug.........................................................................   3       1    seq
    .................ucuuUacaucugggccacgaug.........................................................................   2       1    seq
    .................ucuucacGucugggccacgaugg........................................................................   1       1    seq
    ...................uucacGucugggccacg............................................................................   3       1    seq
    ...................uucacCucugggccacg............................................................................   1       1    seq
    ...................uucacGucugggccacga...........................................................................   1       1    seq
    ...................uucacaucugggccacgaug.........................................................................   3       0    seq
    ...................uucacGucugggccacgaug.........................................................................   6       1    seq
    ...................uucacaucugggccacgauA.........................................................................   1       1    seq
    ...................uucacGucugggccacgaugg........................................................................   6       1    seq
    ...................uucacGucugggccacgauggu.......................................................................   138     1    seq
    ...................uucacGucugggccacgaugguu......................................................................   1       1    seq
    ....................ucacaucugggccacgG...........................................................................   2       1    seq
    ....................ucacGucugggccacga...........................................................................   129     1    seq
    ....................Ccacaucugggccacga...........................................................................   1       1    seq
    ....................ucacaucugggccacga...........................................................................   85      0    seq
    ....................ucacaucugggccacgU...........................................................................   2       1    seq
    ....................ucacaucugAgccacga...........................................................................   1       1    seq
    ....................ucacaucuggCccacga...........................................................................   5       1    seq
    ....................ucacaucugggccUcga...........................................................................   1       1    seq
    ....................ucGcaucugggccacgau..........................................................................   1       1    seq
    ....................ucacaucugggccacgCu..........................................................................   1       1    seq
    ....................ucCcaucugggccacgau..........................................................................   1       1    seq
```

Mature Star

```
agagguuuuggaggugaucuucacaucugggccacgaugguuuaucuuucuucguuuauuuaucauaaaccauggugccccagaugagaagagcaccuucgccuucguuuau
....................ucacaucuggCccacgau..........................................................................	4	1	seq
....................ucacaucugggccacgau..........................................................................	83	0	seq
....................ucacaucugggccacgaA..........................................................................	1	1	seq
....................ucacaucAgggccacgau..........................................................................	1	1	seq
....................ucacaucugggccacgaG..........................................................................	6	1	seq
....................ucacaucugggccacgaC..........................................................................	12	1	seq
....................ucacaCcugggccacgau..........................................................................	2	1	seq
....................ucacGucugggccacgau..........................................................................	188	1	seq
....................Ccacaucugggccacgaug.........................................................................	1	1	seq
....................ucaUaucugggccacgaug.........................................................................	1	1	seq
....................ucacaucuggUccacgaug.........................................................................	1	1	seq
....................ucacaucugggccacgaCg.........................................................................	1	1	seq
....................ucacaucugggccacgauA.........................................................................	14	1	seq
....................ucacaucugggccacgCug.........................................................................	1	1	seq
....................ucacaucugggccacgaug.........................................................................	81	0	seq
....................ucacaucugggcAacgaug.........................................................................	1	1	seq
....................ucacaucuggCccacgaug.........................................................................	6	1	seq
....................ucacaucugggccacgauC.........................................................................	21	1	seq
....................ucacaucAgggccacgaug.........................................................................	1	1	seq
....................ucacGucugggccacgaug.........................................................................	536	1	seq
....................ucacaucugggccUcgaug.........................................................................	1	1	seq
....................ucacaucugggccacgauU.........................................................................	30	1	seq
....................ucacaucCgggccacgaug.........................................................................	3	1	seq
....................ucacGucugggccacgaugg........................................................................	121	1	seq
....................ucacaucugggccacgauAg........................................................................	1	1	seq
....................ucacaucugggccacgaugU........................................................................	1	1	seq
....................ucacaucugggccacgaugg........................................................................	24	0	seq
....................ucacaucugggccacgauCg........................................................................	2	1	seq
....................ucacaucuggCccacgaugg........................................................................	1	1	seq
....................ucacaucugggccacgaugC........................................................................	1	1	seq
....................ucacaucAgggccacgauggu.......................................................................	1	1	seq
....................ucacGucugggccacgauggu.......................................................................	514	1	seq
....................ucacaCcugggccacgauggu.......................................................................	1	1	seq
....................ucacaucugggccacgauggA.......................................................................	3	1	seq
....................ucacaucuggAccacgauggu.......................................................................	1	1	seq
....................ucacaucugggccacgauggC.......................................................................	32	1	seq
....................ucacaucugggccacgGuggu.......................................................................	1	1	seq
....................ucacaucuggCccacgauggu.......................................................................	7	1	seq
....................ucacauUugggccacgauggu.......................................................................	1	1	seq
....................ucacaucugggccacgauggG.......................................................................	8	1	seq
....................ucacaucugggccacgaugCu.......................................................................	1	1	seq
....................ucacaucugggccGcgauggu.......................................................................	1	1	seq
....................ucacaucugggccaUgauggu.......................................................................	1	1	seq
....................ucacaucugggccacgauggu.......................................................................	172	0	seq
....................ucacaucugggccaGgaugguu......................................................................	2	1	seq
....................ucacaucugggccacgauggCu......................................................................	9	1	seq
....................ucacaucugggcUacgaugguu......................................................................	1	1	seq
....................ucacaucugggccacgauggAu......................................................................	2	1	seq
....................Acacaucugggccacgaugguu......................................................................	4	1	seq
....................ucacGucugggccacgaugguu......................................................................	31318	1	seq
....................ucacaucCgggccacgaugguu......................................................................	3	1	seq
....................ucacaucugggccacgaugUuu......................................................................	1	1	seq
....................ucacaucugggccacgauggua......................................................................	26	1	seq
....................ucacaucugggccacgauggua......................................................................	23	1	seq
....................ucacaucugggccacgauggua......................................................................	285	1	seq
....................Ccacaucugggccacgaugguu......................................................................	4	1	seq
....................Ncacaucugggccacgaugguu......................................................................	6	1	seq
....................ucacaucugggccacgauggGu......................................................................	7	1	seq
....................ucacaucuggUccacgaugguu......................................................................	1	1	seq
....................ucacaucugggccacUaugguu......................................................................	2	1	seq
....................ucacaucugggccGcgaugguu......................................................................	6	1	seq
....................ucacaCcugggccacgaugguu......................................................................	3	1	seq
....................ucacUucugggccacgaugguu......................................................................	1	1	seq
....................ucacaucuAggccacgaugguu......................................................................	1	1	seq
....................ucacaucugCgccacgaugguu......................................................................	2	1	seq
....................ucacaucAgggccacgaugguu......................................................................	7	1	seq
....................ucacaucuggAccacgaugguu......................................................................	2	1	seq
....................Gcacaucugggccacgaugguu......................................................................	2	1	seq
....................ucacaucugggccacgGugguu......................................................................	8	1	seq
....................ucGcaucugggccacgaugguu......................................................................	7	1	seq
```

```
                    Mature                                              Star
agagguuuuggaggugaucuucacaucugggccacgaugguuuaucuuucuucguuuauuuaucauaaaccauggugccccagaugagaagagcaccuucgccuucguuuau
....................ucacaucugggccacgaCgguu.....................................................................   3      1   seq
....................ucaUaucugggccacgaugguu.....................................................................   3      1   seq
....................ucacaucugggccUcgaugguu.....................................................................   2      1   seq
....................ucacaucuggCccacgaugguu.....................................................................   1101   1   seq
....................ucacaucugggccacgaugguu.....................................................................   1675   0   seq
....................ucacaucugggccacgaugguuA....................................................................   4      1   seq
....................uUacaucugggccacgaugguuu....................................................................   3      1   seq
....................ucacaucugggccacgaugguuC....................................................................   51     1   seq
....................ucacGucugggccacgaugguuu....................................................................   240    1   seq
....................ucGcaucugggccacgaugguuu....................................................................   1      1   seq
....................ucacaucugggccacgaugguuu....................................................................   7      0   seq
....................ucacaucuggCccacgaugguuu....................................................................   1      1   seq
....................ucacaucugggccacgaugguuuU...................................................................   1      1   seq
....................ucacaucugggccacgaugguuuC...................................................................   1      1   seq
....................ucacaucugggccacgaugguuua...................................................................   2      0   seq
....................ucacaucugggccacgaugguuuGu..................................................................   1      1   seq
....................ucacaucugggccacgaugguuCau..................................................................   1      1   seq
....................ucacaucugggccacgaugguuCauc.................................................................   1      1   seq
.....................cacGucugggccacgau.........................................................................   1      1   seq
.....................cacaucugggccacgau.........................................................................   1      0   seq
.....................cacGucugggccacgaug........................................................................   5      1   seq
.....................cacaucugggccacgaug........................................................................   2      0   seq
.....................cacGucugggccacgaugg.......................................................................   6      1   seq
.....................cacGucugggccacgauggu......................................................................   18     1   seq
.....................cacaucugggccacgauggu......................................................................   3      0   seq
.....................cacaucugggccacgaugguG.....................................................................   1      1   seq
.....................cacaucugggccacgaugguu.....................................................................   7      0   seq
.....................cacGucugggccacgaugguu.....................................................................   22     1   seq
.....................cacaucugggccacgaugguC.....................................................................   1      1   seq
.....................cacaucuggCccacgaugguu.....................................................................   3      1   seq
.....................cacGucugggccacgaugguuu....................................................................   33     1   seq
.....................cacaucugggccacgaugguuC....................................................................   1      1   seq
......................acGucugggccacgaug........................................................................   12     1   seq
......................acaucugggccacgaug........................................................................   1      0   seq
......................acaucugggccacgaugg.......................................................................   3      0   seq
......................acGucugggccacgaugg.......................................................................   3      1   seq
......................acGucugggccacgauggu......................................................................   6      1   seq
......................acaucugggccacgauggu......................................................................   7      0   seq
......................acaucugggccacgaugguu.....................................................................   9      0   seq
......................acGucugggccacgaugguu.....................................................................   76     1   seq
......................acaucuggCccacgaugguu.....................................................................   2      1   seq
......................acGucugggccacgaugguuu....................................................................   22     1   seq
.......................caucugggccacgaCgg.......................................................................   1      1   seq
.......................caucugggccacgaugg.......................................................................   9      0   seq
.......................caucugggccacgauggC......................................................................   4      1   seq
.......................caucugggccacgaAggu......................................................................   1      1   seq
.......................caucugggccacgauggu......................................................................   35     0   seq
.......................caucGgggccacgauggu......................................................................   1      1   seq
.......................caucugggccacgauggA......................................................................   1      1   seq
.......................caucugggccacgauggGu.....................................................................   1      1   seq
.......................caucugggccacgaugguu.....................................................................   15     0   seq
.......................caucugggccacgaugguC.....................................................................   1      1   seq
.......................caucuggCccacgaugguu.....................................................................   1      1   seq
.......................cGucugggccacgaugguu.....................................................................   44     1   seq
.......................caucCgggccacgaugguu.....................................................................   1      1   seq
.......................cGucugggccacgaugguuu....................................................................   2      1   seq
........................aucugggccacgauggu......................................................................   4      0   seq
........................aucugggccacgauggA......................................................................   1      1   seq
........................aucugggccacgauggGu.....................................................................   1      1   seq
........................Uucugggccacgaugguu.....................................................................   1      1   seq
........................aucugggccacgaugguu.....................................................................   60     0   seq
........................aucugggccacgauggCu.....................................................................   1      1   seq
........................aucugggccacgaugguC.....................................................................   11     1   seq
........................aucuggCccacgaugguu.....................................................................   1      1   seq
........................aucGgggccacgaugguu.....................................................................   3      1   seq
........................aucugggccacgaugguuC....................................................................   1      1   seq
.........................ucugggccacgauggGu.....................................................................   2      1   seq
.........................ucugggccCcgaugguu.....................................................................   50     1   seq
.........................Ccugggccacgaugguu.....................................................................   2      1   seq
.........................ucugggccacgaugguC.....................................................................   46     1   seq
```

Mature Star

```
agagguuuuggaggugaucuucacaucugggccacgaugguuuaucuuucuucguuuauuuaucauaaaccauggugccccagaugagaagagcaccuucgccuucguuuau
.........................ucugggccacgaugguA.....................................................................    6     1    seq
.........................Acugggccacgaugguu.....................................................................    1     1    seq
.........................ucugggcUacgaugguu.....................................................................    1     1    seq
.........................ucugggccacgaugguu.....................................................................    262   0    seq
.........................ucugggAcacgaugguu.....................................................................    1     1    seq
.........................ucugUgccacgaugguu.....................................................................    1     1    seq
.........................ucugggccacgauggAu.....................................................................    1     1    seq
.........................ucugAgccacgaugguu.....................................................................    1     1    seq
.........................ucugggccacgaugguuu....................................................................    4     0    seq
.........................ucugggccCcgaugguuu....................................................................    1     1    seq
.........................ucugggccacgaugguuua...................................................................    2     0    seq
.........................ucugggccacgaugguuuau..................................................................    2     0    seq
.........................ucugggccacgaugguuAauc.................................................................    3     1    seq
.........................ucuggAccacgaugguuuauc.................................................................    7     1    seq
.........................ucCgggccacgaugguuuauc.................................................................    10    1    seq
.........................ucugggccacgaugguuuauG.................................................................    3     1    seq
.........................ucugggccacgaugguuuauUu................................................................    2     1    seq
.........................ucugggccacgaugguuAaucu................................................................    3     1    seq
.........................ucuggAccacgaugguuuaucu................................................................    3     1    seq
.........................ucCgggccacgaugguuuaucu................................................................    9     1    seq
.........................ucCgggccacgaugguuuaucuu...............................................................    1     1    seq
.........................ucCgggccacgaugguuuaucuuuc.............................................................    1     1    seq
.............................................................ucauaaaccauggugccccagauU..........................    1     1    seq
..............................................................cauaaaccauggugccccag.............................    1     0    seq
...............................................................auaaaccauggugccccagau...........................    2     0    seq
...............................................................auaaaccauggugccccagaC...........................    1     1    seq
................................................................uaaaccauggugccccagauA..........................    2     1    seq
................................................................uaaaccGuggugccccagaug..........................    1     1    seq
................................................................uaaaccauCgugccccagaug..........................    3     1    seq
................................................................uaaaccauggugccccagaug..........................    30    0    seq
................................................................uaaaccauggugccccagaugU.........................    1     1    seq
..................................................................aaccauCgugccccaga............................    1     1    seq
....................................................................ccauggugccccagaug..........................    1     0    seq
....................................................................ccauggugccccagaugaga.......................    5     0    seq
....................................................................ccauggugccccagaugagG.......................    1     1    seq
....................................................................ccauggugccccagaugagaa......................    3     0    seq
....................................................................ccauggugccccGgaugagaag.....................    1     1    seq
....................................................................ccauggugccccagaugagaag.....................    15    0    seq
.....................................................................cauggugccccagaugagaag.....................    1     0    seq
......................................................................auggugccccagaugaga.......................    3     0    seq
......................................................................auggugccccagaugagaaA.....................    1     1    seq
......................................................................auggugccccagaugagaag.....................    7     0    seq
```

Provisional ID	:	MA_139174_21250
Score total	:	5538.5
Score for star read(s)	:	3.9
Score for read counts	:	5539.4
Score for mfe	:	-2.4
Score for randfold	:	-2.2
Score for cons. seed	:	
Total read count	:	10877
Mature read count	:	10872
Loop read count	:	0
Star read count	:	5

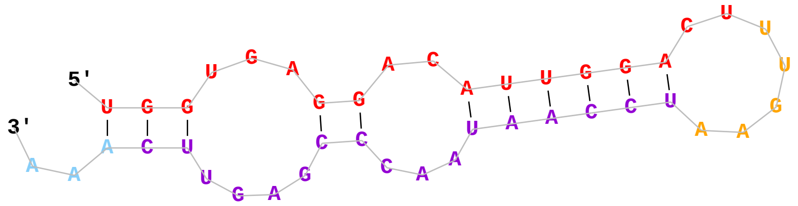

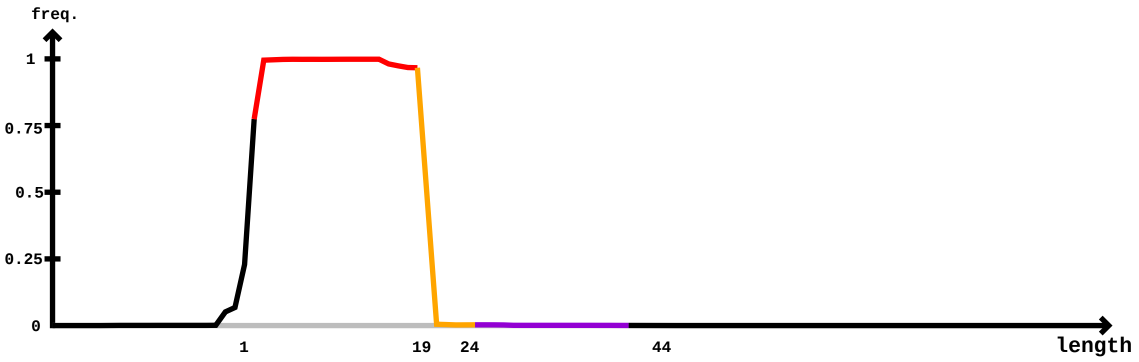

Mature Star

```
5'- augugguucuaugguauaguggugaggacauuggacuuugaauccaauaacccgaguucaaauaucgauggaaccuacauucuuuguuuucauccuuuaauuaugguu   -3'   obs
    augugguucuaugguauaguggugaggacauuggacuuugaauccaauaacccgaguucaaauaucgauggaaccuacauucuuuguuuucauccuuuaauuaugguu         exp
    ((((((((((((((((..(((...((..((((((.......))))))...))....))).))))).))))))).))))........................   reads   mm   sample
    ....gguucuaugguauagugg......................................................................................   1   0   seq
    ....gguucuaugguauagugguU....................................................................................   1   1   seq
    ....gguucuaugguGuaguggug....................................................................................   1   1   seq
    .....guucuaugguauagugg......................................................................................   1   0   seq
    .....guucuaugguGuaguggug....................................................................................   3   1   seq
    .........uGgguauagguggugag..................................................................................   1   1   seq
    ...............auagugguCaggacauug...........................................................................   1   1   seq
    ...............Nuaguggugaggacauug...........................................................................   1   1   seq
    ...............Nuaguggugaggacauugg..........................................................................   1   1   seq
    ...............Nuaguggugaggacauuggacu.......................................................................   1   1   seq
    ................uGguggugaggacauug...........................................................................   1   1   seq
    ................Naguggugaggacauug...........................................................................   1   1   seq
    ................uaguggugaggacauuA...........................................................................   7   1   seq
    ................uaguggugaggacauAg...........................................................................   1   1   seq
    ................uaguggugaggacauuC...........................................................................   1   1   seq
    ................uaguggugaggacauug...........................................................................   187   0   seq
    ................uaguggugaggGcauug...........................................................................   2   1   seq
    ................uaguggugaggacauugg..........................................................................   55   0   seq
    ................uaguggugaggacauCgg..........................................................................   1   1   seq
    ................uaguggugaggacauuAg..........................................................................   3   1   seq
    ................uaguggugaggacauugA..........................................................................   2   1   seq
    ................uaguggugaggacauugga.........................................................................   21   0   seq
    ................uaguggugGggacauuggacu.......................................................................   1   1   seq
    ................uaguggugaggacauuggacA.......................................................................   8   1   seq
    ................uaguggugaggacauuggGcu.......................................................................   3   1   seq
    ................uaguggCgaggacauuggacu.......................................................................   1   1   seq
    ................Gaguggugaggacauuggacu.......................................................................   1   1   seq
    ................uaguggugaggacauuggacG.......................................................................   4   1   seq
    ................uGguggugaggacauuggacu.......................................................................   1   1   seq
    ................uaguggugaggacauuggacu.......................................................................   219   0   seq
    ................uagAggugaggacauuggacu.......................................................................   1   1   seq
    ................uaguggugaggGcauuggacu.......................................................................   2   1   seq
    ................uaguggugaggacauuggacC.......................................................................   37   1   seq
```

Mature **Star**

augugguucuaugguauaguggugaggacauuggacuuugaauccaauaacccgaguucaaauaucgauggaaccuacauucuuuguuuucauccuuuaauuaugguu			
................uagugguAaggacauuggacuu..	2	1	seq
................uaguggugaggacauuggacuu..	1	0	seq
................uagugguCaggacauuggacuu..	1	1	seq
................uagCggugaggacauuggacuu..	11	1	seq
................uaguggugaggacauuggacuuGg..	1	1	seq
................uaguggugaggacauuggacuuGgaaucca..	2	1	seq
.................aguggugaggacauugC..	1	1	seq
.................aguggugaggacauugg..	18	0	seq
.................aguggugaggacauCgg..	1	1	seq
.................aguggugaggacauugA..	1	1	seq
.................aguggugaggacauugga...	6	0	seq
.................aguggugaggacauuggacG...	5	1	seq
.................aguggugaggCcauuggacu...	1	1	seq
.................aguggugaggacauuggacA...	5	1	seq
.................aguggugGggacauuggacu...	1	1	seq
.................aguggugaggacauuggacC...	25	1	seq
.................aguggugaggacauuggacu...	115	0	seq
.................aguggugaggacauuggacuu..	2	0	seq
.................agugguCaggacauuggacuu..	2	1	seq
.................agCggugaggacauuggacuu..	4	1	seq
.................agugguAaggacauuggacuu..	1	1	seq
.................aguggugaggacauuggacuuG...	1	1	seq
..................guggugaggacauugga...	47	0	seq
..................guggugaggacauuggac..	1	0	seq
..................guggugaggacauugAacu...	1	1	seq
..................guggugaggaGauuggacu...	1	1	seq
..................gugUugaggacauuggacu...	1	1	seq
..................guggugaggGcauuggacu...	3	1	seq
..................Nuggugaggacauuggacu...	1	1	seq
..................guggugaggacaAuggacu...	2	1	seq
..................Auggugaggacauuggacu...	2	1	seq
..................guggugaggacauuAgacu...	1	1	seq
..................guggugaggacauuggacu...	1382	0	seq
..................guggugCggacauuggacu...	1	1	seq
..................guggugaggacauuggaUu...	4	1	seq
..................guggugaggacauuggUcu...	1	1	seq
..................guggugaggacauuggacG...	49	1	seq
..................Cuggugaggacauuggacu...	2	1	seq
..................guggugagAacauuggacu...	3	1	seq
..................Uuggugaggacauuggacu...	1	1	seq
..................guggugaggacauCggacu...	1	1	seq
..................guggugaggacauAggacu...	2	1	seq
..................guggCgaggacauuggacu...	3	1	seq
..................guggugUggacauuggacu...	2	1	seq
..................gAggugaggacauuggacu...	1	1	seq
..................guggugaggUcauuggacu...	1	1	seq
..................guggugaggacGuuggacu...	2	1	seq
..................guggugaggacauuggacA...	36	1	seq
..................guggugGggacauuggacu...	8	1	seq
..................guggugaggacauuggacC...	233	1	seq
..................guggugaggacauGggacu...	1	1	seq
..................guggugaggacaCuggacu...	1	1	seq
..................guggugaggCcauuggacu...	1	1	seq
..................guggugaggacauuggCcu...	1	1	seq
..................gugAugaggacauuggacu...	1	1	seq
..................gugguCaggacauuggacuu..	7	1	seq
..................gugguUaggacauuggacuu..	1	1	seq
..................gugguAaggacauuggacuu..	4	1	seq
..................guggugaggacauuggacCu..	4	1	seq
..................guggugaggacauuggacuu..	15	0	seq
..................gCggugaggacauuggacuu..	23	1	seq
...................uggugaggacauuggac..	8	0	seq
...................uggugaggacauuggCc..	1	1	seq
...................uggugaggacauuggaU..	1	1	seq
...................uggugaggUcauuggacu...	1	1	seq
...................uggGgaggacauuggacu...	1	1	seq
...................uggCgaggacauuggacu...	16	1	seq
...................Nggugaggacauuggacu...	8	1	seq
...................uggugaggacauugAacu...	1	1	seq
...................uggugGggacauuggacu...	20	1	seq

```
                   Mature      Star
augugguucuaugguauaguggugaggacauuggacuuugaauccaauaacccgaguucaaauaucgauggaaccuacauucuuuguuuucauccuuuaauuaugguu
...................uggugaggacauuggCcu.......................................................................	3	1	seq
...................uggugaggacauuggacu.......................................................................	4851	0	seq
...................uggugaggacaCuggacu.......................................................................	9	1	seq
...................ugAugaggacauuggacu.......................................................................	1	1	seq
...................uggAgaggacauuggacu.......................................................................	3	1	seq
...................uggugaggacauAggacu.......................................................................	3	1	seq
...................uggugaggCcauuggacu.......................................................................	1	1	seq
...................uggugaggacauuggaUu.......................................................................	3	1	seq
...................uggugCggacauuggacu.......................................................................	1	1	seq
...................uggugaggacauugCacu.......................................................................	2	1	seq
...................uggugaggacauuggacC.......................................................................	848	1	seq
...................uggugaggacauugUacu.......................................................................	2	1	seq
...................uggugaggacauGggacu.......................................................................	3	1	seq
...................uCgugaggacauuggacu.......................................................................	1	1	seq
...................uggugaggacauuggUcu.......................................................................	3	1	seq
...................uggugaggacaAuggacu.......................................................................	1	1	seq
...................uggugaggacauuAgacu.......................................................................	3	1	seq
...................uggugUggacauuggacu.......................................................................	4	1	seq
...................uggugagAacauuggacu.......................................................................	3	1	seq
...................uggugaggacauCggacu.......................................................................	15	1	seq
...................uggugaAgacauuggacu.......................................................................	1	1	seq
...................uggugaggacaGuggacu.......................................................................	1	1	seq
...................uAgugaggacauuggacu.......................................................................	4	1	seq
...................uggugaggacauuggacG.......................................................................	166	1	seq
...................uggugaggacauuggacA.......................................................................	176	1	seq
...................ugguAaggacauuggacuu......................................................................	13	1	seq
...................uggugaggacauuggacuu......................................................................	33	0	seq
...................uggugaggacauuggGcuu......................................................................	1	1	seq
...................ugguUaggacauuggacuu......................................................................	1	1	seq
...................Gggugaggacauuggacuu......................................................................	1	1	seq
...................uggugaggacauuggacCu......................................................................	1	1	seq
...................uggugaggacauuggacuG......................................................................	7	1	seq
...................ugguCaggacauuggacuu......................................................................	7	1	seq
...................Aggugaggacauuggacuu......................................................................	1	1	seq
...................Cggugaggacauuggacuu......................................................................	29	1	seq
...................uggugaggacauuggacuGu.....................................................................	1	1	seq
...................uggugaggacauuggacuuu.....................................................................	2	0	seq
...................uggugaggacauuggacuuug....................................................................	1	0	seq
...................uggugaggacauuggacuuGgaaucca..............................................................	1	1	seq
...................uggugaggacauuggacuuugaaucca..............................................................	2	0	seq
...................uggugaggacauuggacuuugaauccaGuaacccgaguuca................................................	1	1	seq
....................ggugaggacauuggaUu.......................................................................	1	1	seq
....................ggugaggacaCuggacu.......................................................................	6	1	seq
....................ggugaggacauuAgacu.......................................................................	2	1	seq
....................ggugGggacauuggacu.......................................................................	5	1	seq
....................Cgugaggacauuggacu.......................................................................	2	1	seq
....................ggugaAgacauuggacu.......................................................................	1	1	seq
....................ggugaggacauGggacu.......................................................................	1	1	seq
....................ggCgaggacauuggacu.......................................................................	9	1	seq
....................ggugaggacauuggacA.......................................................................	63	1	seq
....................Agugaggacauuggacu.......................................................................	3	1	seq
....................ggugaggacauuggUcu.......................................................................	1	1	seq
....................ggugaggacauCggacu.......................................................................	3	1	seq
....................ggugaggacaGuggacu.......................................................................	1	1	seq
....................ggugagAacauuggacu.......................................................................	1	1	seq
....................ggugaggacauuggacC.......................................................................	288	1	seq
....................ggugaggaGauuggacu.......................................................................	3	1	seq
....................gAugaggacauuggacu.......................................................................	3	1	seq
....................ggAgaggacauuggacu.......................................................................	2	1	seq
....................ggugaggacauuggacG.......................................................................	75	1	seq
....................Ugugaggacauuggacu.......................................................................	2	1	seq
....................ggugaggacauuUgacu.......................................................................	1	1	seq
....................ggugaUgacauuggacu.......................................................................	1	1	seq
....................ggugaggUcauuggacu.......................................................................	1	1	seq
....................ggugagUacauuggacu.......................................................................	1	1	seq
....................ggugUggacauuggacu.......................................................................	3	1	seq
....................ggugaggacauugAacu.......................................................................	1	1	seq
....................ggugaggacauuggaGu.......................................................................	1	1	seq
....................ggugaggacauuggacu.......................................................................	1978	0	seq
....................ggugaggacauAggacu.......................................................................	1	1	seq
```

Mature Star

augugguucuaugguauaguggugaggacauuggacuuugaauccaauaacccgaguucaaauaucgauggaaccuacauucuuuguuuucauccuuuaauuaugguu

....................Ngugaggacauuggacu...	1	1	seq
....................ggugaggacauuggacCu..	1	1	seq
....................gguAaggacauuggacuu..	17	1	seq
....................ggugaggacauuggacuu..	42	0	seq
....................ggugaggacauuggGcuu..	1	1	seq
....................ggugagUacauuggacuu..	1	1	seq
....................gguUaggacauuggacuu..	5	1	seq
....................gguCaggacauuggacuu..	8	1	seq
....................gguUaggacauuggacuuu...	1	1	seq
....................ggugaggacauuggacuuug..	1	0	seq
....................gguCaggacauuggacuuugaauc..	1	1	seq
....................ggugaggacauuggacuuugaaucca..	1	0	seq
.....................gugaggacauuggacuu..	6	0	seq
.....................guCaggacauuggacuu..	1	1	seq
.....................guAaggacauuggacuu..	3	1	seq
.....................gugaggacauuggacuuu...	1	0	seq
.....................gugaggacauuggacuuG...	1	1	seq
.....................gugaggacauuggacuuGg..	1	1	seq
.....................gugaggacauuggacuuug..	1	0	seq
.....................gugaggacauuggacuuGgaaucc...	1	1	seq
.....................gugaggacauuggacuuGgaauccaa...	1	1	seq
......................uAaggacauuggacuuu...	1	1	seq
......................uCaggacauuggacuuu...	1	1	seq
......................ugaggacauuggacuuC...	2	1	seq
......................ugaggacauuggacuuG...	1	1	seq
......................uCaggacauuggacuuug..	2	1	seq
......................ugaggacauuggGcuuug..	1	1	seq
......................ugaggacauuggacuuug..	3	0	seq
......................ugaggacauuggacuuugaaucc...	2	0	seq
......................uUaggacauuggacuuugaaucca..	1	1	seq
......................ugaggacauuggacuuugaaucca..	3	0	seq
......................uAaggacauuggacuuugaaucca..	1	1	seq
.......................gaggacauuggacuuug..	2	0	seq
.......................Aaggacauuggacuuug..	3	1	seq
.......................Caggacauuggacuuug..	1	1	seq
........................aggacauuggacuuugaaucca..	2	0	seq
........................aggacauuggacuuugaauccaGuaacccgagu...	1	1	seq
.........................ggacauuggacuuugaaucca..	1	0	seq
...........................acauuggacuuugaaucca..	1	0	seq
............................cauuggacuuugaaucA...	1	1	seq
............................Gauuggacuuugaaucca..	1	1	seq
.............................auuggacuuugaaucca..	1	0	seq
...................................cuuugaauccaGuaacccga...	1	1	seq
...................................cuuugaauccaGuaacccgagu...	1	1	seq
....aauccaauaacccgaguuc...	1	0	seq
....auccaauaacccgaguu..	1	0	seq
....auccaauaacccgaguuc...	1	0	seq
....uccaUuaacccgaguuc...	1	1	seq
....uccaauaacccgaguuc...	1	0	seq

Provisional ID	:	MA_13984_4785
Score total	:	0.3
Score for star read(s)	:	-1.3
Score for read counts	:	0
Score for mfe	:	0
Score for randfold	:	1.6
Score for cons. seed	:	
Total read count	:	15
Mature read count	:	15
Loop read count	:	0
Star read count	:	0

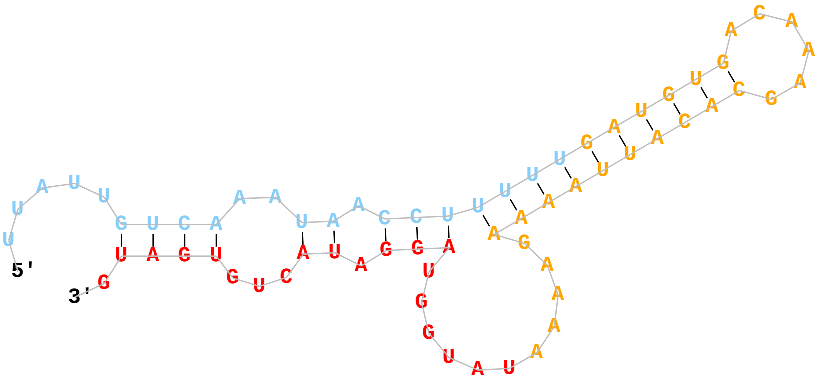

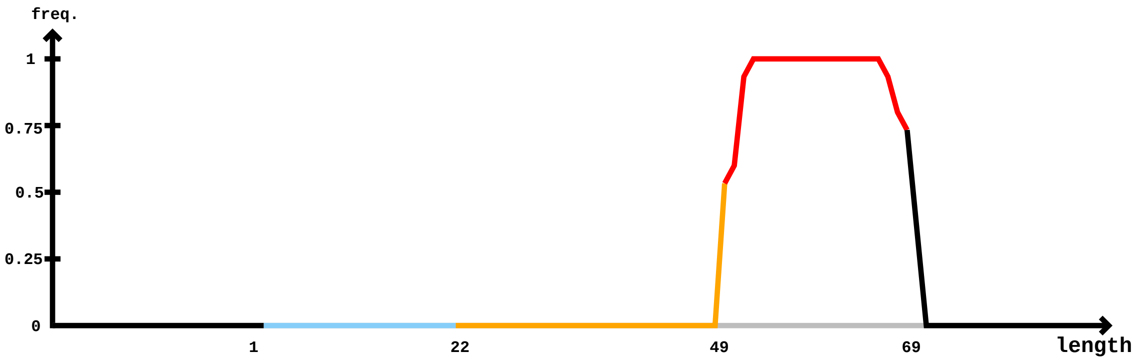

Star Mature

```
5'- uguugcauuaauggcuucuauuauugucaaauaaccuuuuugaugugacaaagcacauuaaaagaaaauaugguaggauacugugauggaggaguaugauguaucaua   -3'    exp
    ((.(((((((....((((((((((((((.....((((((((((((((......))))))))))))).......)))..))))..))))))))))....))))))).))..   reads      mm        sample
    ....................................................................Caugguaggauacugug.......................   1          1         seq
    ....................................................................Caugguaggauacuguga......................   1          1         seq
    ....................................................................Caugguaggauacugugau.....................   1          1         seq
    ....................................................................Caugguaggauacugugaug....................   5          1         seq
    .....................................................................augguaggauacuguga......................   1          0         seq
    ......................................................................ugguaggauacugugaug....................   5          0         seq
    .......................................................................gguaggauacugugaug....................   1          0         seq
```

Provisional ID	:	MA_14099_6964
Score total	:	100.1
Score for star read(s)	:	3.9
Score for read counts	:	92.4
Score for mfe	:	2.2
Score for randfold	:	1.6
Score for cons. seed	:	
Total read count	:	193
Mature read count	:	128
Loop read count	:	0
Star read count	:	65

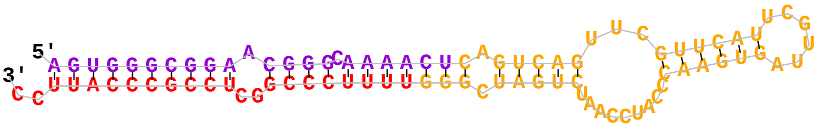

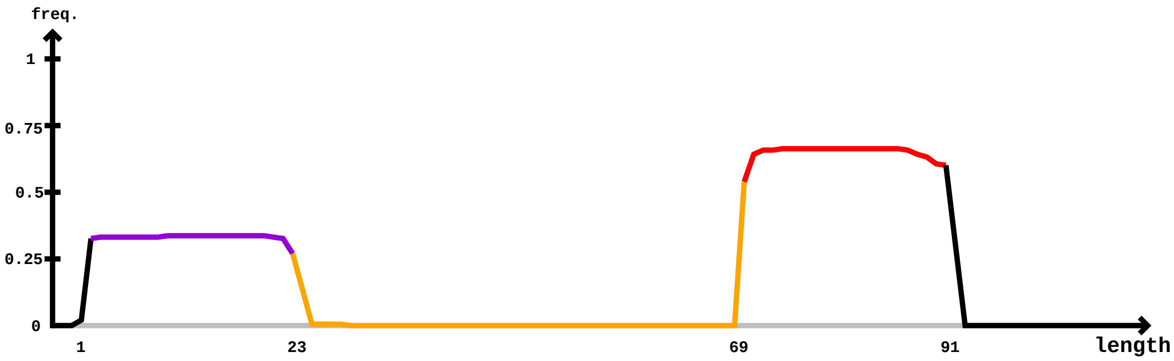

Star Mature

5'- ggagugggcggaacgggcaaaacucagucaguucguucauucguuagugaacccauccaaucugaucggguuuucccggcuccgcccauuccuacaguccucacuacgcauc -3'	obs		
ggagugggcggaacgggcaaaacucagucaguucguucauucguuagugaacccauccaaucugaucggguuuucccggcuccgcccauuccuacaguccucacuacgcauc	exp		
((((((((((((.((((.(((((((.(((((...((((((......)))))).........))))).))))))))))).)))))))))))))....................	reads	mm	sample
.gagugAgcggaacgggcaaaa..	1	1	seq
.gagugAgcggaacgggcaaaac...	3	1	seq
..agugAgcggaacgggcaaaac...	8	1	seq
..agugAgcggaacgggcaaaacu..	51	1	seq
...gugAgcggaacgggcaaa...	1	1	seq
..........gaacgggcaaaacuGaguc...	1	1	seq
....uuuucccggcuccgccc.........................	1	0	seq
....uuuucccggcuccgccca........................	2	0	seq
....uuuucccAgcuccgcccauu......................	1	1	seq
....uuuucccggcuccgcccauu......................	3	0	seq
....uuuuUccggcuccgcccauuc.....................	1	1	seq
....uuuucccggcuccgcccauucA....................	5	1	seq
....uuuucccggcuccgcUcauucc....................	1	1	seq
....uuuucccggcuccgcccauucU....................	8	1	seq
....uuuucccggcuccgcccauucc....................	78	0	seq
....uuuucccggcCccgcccauucc....................	1	1	seq
....Cuuucccggcuccgcccauucc....................	2	1	seq
....uuuucccggcuccgcccauuUc....................	1	1	seq
....uuucccggcuccgccca........................	1	0	seq
....uuucccggcuccgcccau.......................	1	0	seq
....uuucccggcuccgcccauu......................	1	0	seq
....uuucccggcuccgcccauucc....................	3	0	seq
....uuucccggcuccgcccauuccA...................	1	1	seq
....uuucccggcuccgcccauuccC...................	3	1	seq
....uuucccggcuccgcccauuccu...................	10	0	seq
....uucccggcuccgcccau.......................	1	0	seq
....uucccggcuccgcccauuccu...................	2	0	seq
....cccggcuccgcccauucA....................	1	1	seq

Provisional ID : MA_14353_11404
Score total : 1.1
Score for star read(s) : -1.3
Score for read counts : 0
Score for mfe : 0.8
Score for randfold : 1.6
Score for cons. seed :
Total read count : 38101
Mature read count : 36583
Loop read count : 1518
Star read count : 0

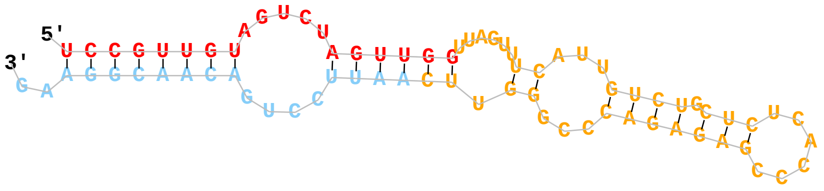

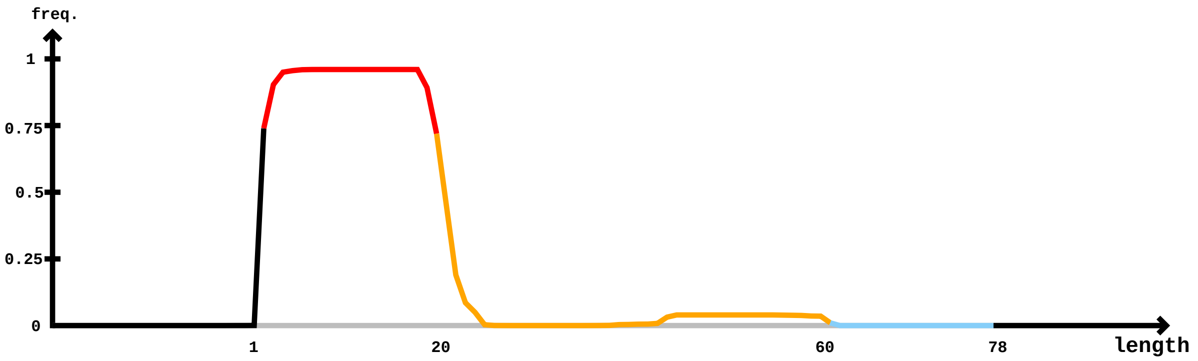


```
                                           Mature                                            Star
5'- auuuaaauauauuuggcaguuccguuguagucuaguugguuaguuucauugucugcucucacccgagagacccggguucaauuccugacaacggaaguuaaauauauuuuguag   -3'    exp
    ....(((((((((((((..(((((((((.....((((((......((...((((.(((......)))))))...)).))))))....))))))))))))))))))))))).....   reads   mm   sample
    .................aCuuccguuguagucuaguu.............................................................................   1       1    seq
    .................Cguuccguuguagucuaguug............................................................................   1       1    seq
    ..................guuccguuguCgucuagu..............................................................................   1       1    seq
    ...................uCccguuguagucuagu..............................................................................   1       1    seq
    ...................uuccguuguagucuaguu.............................................................................   1       0    seq
    ...................uuccguuguagucuaguug............................................................................   1       0    seq
    ...................uuccgCuguagucuaguug............................................................................   1       1    seq
    ...................uuccguuguagucuaguugg...........................................................................   1       0    seq
    ...................uuccguuguagucuaguuggu..........................................................................   1       0    seq
    ...................uuccguuguagucuaguugguu.........................................................................   2       0    seq
    ....................uccguuguagucuagCu.............................................................................   77      1    seq
    ....................uccUuuguagucuaguu.............................................................................   1       1    seq
    ....................uccguuguGgucuaguu.............................................................................   7       1    seq
    ....................uccgAuguagucuaguu.............................................................................   1       1    seq
    ....................uccgCuguagucuaguu.............................................................................   6       1    seq
    ....................uccguuguagucuagGu.............................................................................   6       1    seq
    ....................uccguuguagucGaguu.............................................................................   2       1    seq
    ....................uccguuguagucuCguu.............................................................................   1       1    seq
    ....................uccguuguagucuaguG.............................................................................   35      1    seq
    ....................uccguuguagucuaCuu.............................................................................   1       1    seq
    ....................uccguuguagAcuaguu.............................................................................   3       1    seq
    ....................uccguugCagucuaguu.............................................................................   3       1    seq
    ....................uccguuguUgucuaguu.............................................................................   11      1    seq
    ....................Gccguuguagucuaguu.............................................................................   3       1    seq
    ....................uccguuguagucuaguA.............................................................................   121     1    seq
    ....................Nccguuguagucuaguu.............................................................................   4       1    seq
    ....................uccguuguagucuaguu.............................................................................   2009    0    seq
    ....................uccguuAuagucuaguu.............................................................................   1       1    seq
    ....................uccguuguagucuUguu.............................................................................   1       1    seq
    ....................uccguuguagGcuaguu.............................................................................   2       1    seq
    ....................uccguAguagucuaguu.............................................................................   1       1    seq
    ....................uccguuguagucuaguC.............................................................................   288     1    seq
    ....................uccguugGagucuaguu.............................................................................   1       1    seq
    ....................uccguuguaAucuaguug............................................................................   2       1    seq
```

```
                    Mature                                                           Star
auuuaaauauauuuggcaguuccguuguagucuaguugguuaguuucauugucugcucucacccgagagacccgguucaauuccugacaacggaaguuaaauauauuuuguag
....................ucGguuguagucuaguug...........................................................................   2      1   seq
....................uccguuguGgucuaguug...........................................................................   8      1   seq
....................uccguuguagucuaguug...........................................................................   4470   0   seq
....................uccguuguagGcuaguug...........................................................................   2      1   seq
....................uccguuguagucuagGug...........................................................................   16     1   seq
....................uccguuguagucGaguug...........................................................................   2      1   seq
....................uccguuguagAcuaguug...........................................................................   3      1   seq
....................Gccguuguagucuaguug...........................................................................   4      1   seq
....................uccguAguagucuaguug...........................................................................   3      1   seq
....................uccgCuguagucuaguug...........................................................................   8      1   seq
....................uccguuguagucuCguug...........................................................................   1      1   seq
....................Nccguuguagucuaguug...........................................................................   4      1   seq
....................uccUuuguagucuaguug...........................................................................   1      1   seq
....................uccguuCuagucuaguug...........................................................................   1      1   seq
....................uccguuguagucuagCug...........................................................................   98     1   seq
....................uccguuguagucuagAug...........................................................................   3      1   seq
....................uccguugCagucuaguug...........................................................................   10     1   seq
....................uccguuguaguAuaguug...........................................................................   1      1   seq
....................uccguugAagucuaguug...........................................................................   1      1   seq
....................uccguuguaUucuaguug...........................................................................   3      1   seq
....................uccgGuguagucuaguug...........................................................................   1      1   seq
....................uccguuguagucuaguAg...........................................................................   34     1   seq
....................uccguuguagucuaguuC...........................................................................   6      1   seq
....................uccguuguaCucuaguug...........................................................................   1      1   seq
....................uccguuguaguUuaguug...........................................................................   5      1   seq
....................uccguugGagucuaguug...........................................................................   1      1   seq
....................uccguuguagucuaguGg...........................................................................   19     1   seq
....................uccguuguUgucuaguug...........................................................................   1      1   seq
....................uccguuAuagucuaguug...........................................................................   3      1   seq
....................uccguuguCgucuaguug...........................................................................   2      1   seq
....................uccguuguagucuaguuU...........................................................................   22     1   seq
....................uccguuguagucuaguCg...........................................................................   29     1   seq
....................uccguuguagucuaguuA...........................................................................   203    1   seq
....................uccguuguagucuaUuug...........................................................................   2      1   seq
....................Gccguuguagucuaguugg..........................................................................   15     1   seq
....................uccgCuguagucuaguugg..........................................................................   18     1   seq
....................uccguuguaguGuaguugg..........................................................................   1      1   seq
....................uccguugCagucuaguugg..........................................................................   18     1   seq
....................uccguuguaAucuaguugg..........................................................................   5      1   seq
....................uccguuguUgucuaguugg..........................................................................   6      1   seq
....................uNcguuguagucuaguugg..........................................................................   1      1   seq
....................Accguuguagucuaguugg..........................................................................   6      1   seq
....................uccguuguagucuagGugg..........................................................................   3      1   seq
....................uccguugAagucuaguugg..........................................................................   3      1   seq
....................uAcguuguagucuaguugg..........................................................................   1      1   seq
....................uccguuguagucuCguugg..........................................................................   1      1   seq
....................uccguuguagucGaguugg..........................................................................   1      1   seq
....................uccguuguagucuagCugg..........................................................................   71     1   seq
....................uccguuguagucuaguuCg..........................................................................   3      1   seq
....................uccguuguagucuagAugg..........................................................................   3      1   seq
....................uccguuguagucuaguugA..........................................................................   592    1   seq
....................uccUuuguagucuaguugg..........................................................................   1      1   seq
....................uccguuguagucuaguugU..........................................................................   97     1   seq
....................uccguuguagucuaguuAg..........................................................................   41     1   seq
....................uccguuguGgucuaguugg..........................................................................   25     1   seq
....................Nccguuguagucuaguugg..........................................................................   12     1   seq
....................uccguGguagucuaguugg..........................................................................   1      1   seq
....................uccgGuguagucuaguugg..........................................................................   1      1   seq
....................uccguuguaguUuaguugg..........................................................................   7      1   seq
....................uccguAguagucuaguugg..........................................................................   3      1   seq
....................uccguugGagucuaguugg..........................................................................   1      1   seq
....................uccguuguagucuUguugg..........................................................................   3      1   seq
....................ucGguuguagucuaguugg..........................................................................   1      1   seq
....................uccguuguagucuaguugC..........................................................................   71     1   seq
....................uccguuguagucuaguugg..........................................................................   7467   0   seq
....................uccgAuguagucuaguugg..........................................................................   4      1   seq
....................uccguuguagGcuaguugg..........................................................................   3      1   seq
....................uccguuguaguAuaguugg..........................................................................   1      1   seq
....................uccguuguagucAaguugg..........................................................................   1      1   seq
....................uccguuguagAcuaguugg..........................................................................   5      1   seq
```

Mature **Star**

```
auuuaaauauauuuggcaguuccguuguagucuaguugguuaguuucauugucugcucucacccgagagacccgguucaauuccugacaacggaaguuaaauauauuuuguag
....................uccguuguCgucuaguugg..........................................................................    2     1    seq
....................uccguuguagucuaguGgg..........................................................................    11    1    seq
....................uccguuguagucuaguAgg..........................................................................    44    1    seq
....................uccguuguagucuaCuugg..........................................................................    1     1    seq
....................uccguuguagucuaguCgg..........................................................................    34    1    seq
....................uccCuuguagucuaguuggu.........................................................................    1     1    seq
....................uccguuguaguUuaguuggu.........................................................................    1     1    seq
....................Gccguuguagucuaguuggu.........................................................................    5     1    seq
....................uccguuguaAucuaguuggu.........................................................................    1     1    seq
....................uccguuguaguGuaguuggu.........................................................................    1     1    seq
....................uccguuguagucuaguuggG.........................................................................    124   1    seq
....................uccguuguagucuaCuuggu.........................................................................    1     1    seq
....................uccguuguGgucuaguuggu.........................................................................    13    1    seq
....................uccguuguagucuaguuggC.........................................................................    1484  1    seq
....................uccguuguagucuaguGggu.........................................................................    3     1    seq
....................uccguuguagucuaguAggu.........................................................................    22    1    seq
....................uccgCuguagucuaguuggu.........................................................................    12    1    seq
....................uccguuAuagucuaguuggu.........................................................................    1     1    seq
....................uccguuguagucuagAuggu.........................................................................    1     1    seq
....................uccguuguagucuaguuggA.........................................................................    85    1    seq
....................uccguuguagucuaguuggu.........................................................................    3642  0    seq
....................Nccguuguagucuaguuggu.........................................................................    7     1    seq
....................Cccguuguagucuaguuggu.........................................................................    11    1    seq
....................uccguuguaguAuaguuggu.........................................................................    1     1    seq
....................uccguuguagAcuaguuggu.........................................................................    3     1    seq
....................Accguuguagucuaguuggu.........................................................................    1     1    seq
....................uccguuguagucuUguuggu.........................................................................    3     1    seq
....................uccguuguaUucuaguuggu.........................................................................    1     1    seq
....................uccguuguagucGaguuggu.........................................................................    1     1    seq
....................uccguugAagucuaguuggu.........................................................................    1     1    seq
....................uccguuguagucuaguugCu.........................................................................    1     1    seq
....................uccguuguagucuaguuCgu.........................................................................    1     1    seq
....................uccguuguagucuaUuuggu.........................................................................    1     1    seq
....................uccguuguagucuaguuAgu.........................................................................    5     1    seq
....................ucGguuguagucuaguuggu.........................................................................    2     1    seq
....................uccguuguUgucuaguuggu.........................................................................    1     1    seq
....................uccguuguagucuagGuggu.........................................................................    2     1    seq
....................uccguuguagucuaguCggu.........................................................................    5     1    seq
....................uccguuguagucAaguuggu.........................................................................    1     1    seq
....................uccguugCagucuaguuggu.........................................................................    6     1    seq
....................uccguAguagucuaguuggu.........................................................................    2     1    seq
....................uccguuguagucuagCuggu.........................................................................    15    1    seq
....................uccguuguagucuaguugAu.........................................................................    38    1    seq
....................uccgGuguagucuaguugguu........................................................................    1     1    seq
....................uccguuguagucuCguugguu........................................................................    1     1    seq
....................uccguuguagucuaguugguu........................................................................    2760  0    seq
....................uccguuguagucuaguCgguu........................................................................    3     1    seq
....................uccguugCagucuaguugguu........................................................................    5     1    seq
....................uccguuguGgucuaguugguu........................................................................    8     1    seq
....................uccguuguagucuaguuggAu........................................................................    6     1    seq
....................uccguuguagucuaguuggGu........................................................................    11    1    seq
....................uccguuguagucGaguugguu........................................................................    2     1    seq
....................Cccguuguagucuaguugguu........................................................................    11    1    seq
....................uccguuguaguUuaguugguu........................................................................    1     1    seq
....................uccguuguagucuagAugguu........................................................................    1     1    seq
....................uccguuguagucuaguGgguu........................................................................    1     1    seq
....................Gccguuguagucuaguugguu........................................................................    2     1    seq
....................uccguuguagucuagCugguu........................................................................    20    1    seq
....................uccgAuguagucuaguugguu........................................................................    2     1    seq
....................Accguuguagucuaguugguu........................................................................    4     1    seq
....................uccguuguagucuaguAgguu........................................................................    39    1    seq
....................uccguuguagucuagGugguu........................................................................    1     1    seq
....................uccgCuguagucuaguugguu........................................................................    8     1    seq
....................uccguuguaAucuaguugguu........................................................................    2     1    seq
....................uccguuguagucuaguuggCu........................................................................    99    1    seq
....................Nccguuguagucuaguugguu........................................................................    2     1    seq
....................uccguuguagAcuaguugguu........................................................................    2     1    seq
....................uccguuguagucuaguugguA........................................................................    82    1    seq
....................uccguuguagucuaguuAguu........................................................................    1     1    seq
....................uccguuAuagucuaguugguu........................................................................    2     1    seq
```

Mature   Star

```
auuuaaauauauuuggcaguuccguuguagucuaguugguuaguuucauugucugcucucacccgagagacccggguucaauuccugacaacggaaguuaaauauauuuuguag
....................uccguuguagucuaguugAuu.........................................................................   550    1    seq
....................uccguuguagucuaguugAuua........................................................................   11     1    seq
....................Cccguuguagucuaguugguua........................................................................   5      1    seq
....................uccguuguGgucuaguugguua........................................................................   3      1    seq
....................uccguuguagucuaguAgguua........................................................................   16     1    seq
....................uccUuuguagucuaguugguua........................................................................   1      1    seq
....................uccguugCagucuaguugguua........................................................................   1      1    seq
....................uccguAguagucuaguugguua........................................................................   1      1    seq
....................uccguuguaguGuaguugguua........................................................................   1      1    seq
....................uccguuguagGcuaguugguua........................................................................   2      1    seq
....................Nccguuguagucuaguugguua........................................................................   2      1    seq
....................uccguuguagucuaguugguuU........................................................................   9      1    seq
....................Gccguuguagucuaguugguua........................................................................   1      1    seq
....................ucGguuguagucuaguugguua........................................................................   1      1    seq
....................uccguuguUgucuaguugguua........................................................................   1      1    seq
....................uccguuguagAcuaguugguua........................................................................   4      1    seq
....................Accguuguagucuaguugguua........................................................................   1      1    seq
....................uccguuguagucuaguuggGua........................................................................   3      1    seq
....................uccguuguagCcuaguugguua........................................................................   4      1    seq
....................uccguuguagucuagCugguua........................................................................   6      1    seq
....................uccguuguagucuaguuAguua........................................................................   4      1    seq
....................uccguuguagucuGguugguua........................................................................   1      1    seq
....................uccguuguaAucuaguugguua........................................................................   1      1    seq
....................uccguuguagucuagAugguua........................................................................   1      1    seq
....................uccguuguagucuaguugguuG........................................................................   9      1    seq
....................uccgCuguagucuaguugguua........................................................................   3      1    seq
....................uccguuguagucuaguugguuC........................................................................   1      1    seq
....................uccguuguaguAuaguugguua........................................................................   1      1    seq
....................uccguuguagucuaguugguAa........................................................................   1      1    seq
....................uccguuguagucuaguuggCua........................................................................   12     1    seq
....................uccguuguaguUuaguugguua........................................................................   3      1    seq
....................uccguuguagucuaguugguua........................................................................   1097   0    seq
....................uccguuguagucuCguugguua........................................................................   1      1    seq
....................uccguuguagucGaguugguuag.......................................................................   1      1    seq
....................uccgAuguagucuaguugguuag.......................................................................   1      1    seq
....................uccguuguGgucuaguugguuag.......................................................................   5      1    seq
....................uccguuguagucuaguugAuuag.......................................................................   4      1    seq
....................uccguuguagucuagAugguuag.......................................................................   1      1    seq
....................uccguuguagucuUguugguuag.......................................................................   2      1    seq
....................uccguuguagucuGguugguuag.......................................................................   4      1    seq
....................uccguuguagucuaguuggCuag.......................................................................   10     1    seq
....................uccguuguagucuaguugguuGg.......................................................................   8      1    seq
....................uccgCuguagucuaguugguuag.......................................................................   4      1    seq
....................Nccguuguagucuaguugguuag.......................................................................   1      1    seq
....................uccguuguagCcuaguugguuag.......................................................................   5      1    seq
....................uccguuguagucAaguugguuag.......................................................................   2      1    seq
....................Accguuguagucuaguugguuag.......................................................................   3      1    seq
....................uccguugCagucuaguugguuag.......................................................................   1      1    seq
....................uccguuguagucuaguugguuaC.......................................................................   3      1    seq
....................uccguuguagucuaguugguuUg.......................................................................   4      1    seq
....................uccguuUuagucuaguugguuag.......................................................................   2      1    seq
....................uccguuguagucuagGugguuag.......................................................................   2      1    seq
....................uccUuuguagucuaguugguuag.......................................................................   1      1    seq
....................uccguuguagucuaguugguuaU.......................................................................   1      1    seq
....................uccguuguagucuaguuggGuag.......................................................................   1      1    seq
....................uccguuguagucuaguAgguuag.......................................................................   21     1    seq
....................uccguuguagucuaguuAguuag.......................................................................   2      1    seq
....................uccguuguaguUuaguugguuag.......................................................................   1      1    seq
....................uccguuguagAcuaguugguuag.......................................................................   1      1    seq
....................uccguuguagucuaguCgguuag.......................................................................   4      1    seq
....................Gccguuguagucuaguugguuag.......................................................................   1      1    seq
....................uccguuguagucuaguugguuag.......................................................................   1451   0    seq
....................Cccguuguagucuaguugguuag.......................................................................   5      1    seq
....................uccguuguagucuagCugguuag.......................................................................   32     1    seq
....................uccguuguagucuaguuggAuag.......................................................................   2      1    seq
....................uccguuguagucuaguugguuagC......................................................................   10     1    seq
....................uccguuguGgucuaguugguuagu......................................................................   1      1    seq
....................uccguuguagucuaguugguuagu......................................................................   7      0    seq
....................uccguuguagucuaguugguuagA......................................................................   72     1    seq
....................uccguuguagucuaguugguCagu......................................................................   5      1    seq
```

```
                    Mature                                                Star
auuuaaauauauuuggcaguuccguuguagucuaguugguuaguuucauugucugcucucacccgagagacccgguucaauuccugacaacggaaguuaaauauauuuuguag
....................uccguuguagucuagCugguuagu.....................................................................  1     1    seq
....................uccguuguagucuaguugguuagGu....................................................................  5     1    seq
....................uccguuguagucuaguugguuaguA....................................................................  1     1    seq
....................uccguuguagucuaguugguuagGuu...................................................................  2     1    seq
.....................ccguugCagucuaguug...........................................................................  3     1    seq
.....................ccguuguagucuaguCg...........................................................................  15    1    seq
.....................ccguuguaAucuaguug...........................................................................  4     1    seq
.....................Ncguuguagucuaguug...........................................................................  2     1    seq
.....................ccguuguagGcuaguug...........................................................................  1     1    seq
.....................ccgCuguagucuaguug...........................................................................  1     1    seq
.....................ccguuguagucuaguGg...........................................................................  8     1    seq
.....................ccguuguagucuaguug...........................................................................  1464  0    seq
.....................ccguuguagucuaguuC...........................................................................  1     1    seq
.....................ccguuguagucuaguAg...........................................................................  4     1    seq
.....................ccguuguagucuaguuA...........................................................................  54    1    seq
.....................ccguuguagucuagCug...........................................................................  29    1    seq
.....................ccguugAagucuaguug...........................................................................  1     1    seq
.....................ccguGguagucuaguug...........................................................................  1     1    seq
.....................ccgAuguagucuaguug...........................................................................  1     1    seq
.....................ccguuguagAcuaguug...........................................................................  1     1    seq
.....................ccguuguagucuagGug...........................................................................  2     1    seq
.....................cGguuguagucuaguug...........................................................................  1     1    seq
.....................ccguuguGgucuaguug...........................................................................  3     1    seq
.....................ccguuguagucuaguuU...........................................................................  5     1    seq
.....................ccguuguCgucuaguug...........................................................................  4     1    seq
.....................ccUuuguagucuaguugg..........................................................................  2     1    seq
.....................ccguuguagucuaguAgg..........................................................................  2     1    seq
.....................ccgCuguagucuaguugg..........................................................................  9     1    seq
.....................ccguuguUgucuaguugg..........................................................................  1     1    seq
.....................ccCuuguagucuaguugg..........................................................................  1     1    seq
.....................ccguuguagAcuaguugg..........................................................................  1     1    seq
.....................ccguuguagucuagGugg..........................................................................  1     1    seq
.....................Ncguuguagucuaguugg..........................................................................  4     1    seq
.....................ccguuguagucuagAugg..........................................................................  3     1    seq
.....................ccguuguagucuaguCgg..........................................................................  7     1    seq
.....................ccguugGagucuaguugg..........................................................................  2     1    seq
.....................ccgAuguagucuaguugg..........................................................................  1     1    seq
.....................ccguuguGgucuaguugg..........................................................................  4     1    seq
.....................ccgGuguagucuaguugg..........................................................................  2     1    seq
.....................ccguuguagucuaguGgg..........................................................................  4     1    seq
.....................ccguugCagucuaguugg..........................................................................  2     1    seq
.....................ccguuguaAucuaguugg..........................................................................  2     1    seq
.....................ccguuguagucuaguugg..........................................................................  1691  0    seq
.....................cGguuguagucuaguugg..........................................................................  1     1    seq
.....................ccguuguaguUuaguugg..........................................................................  1     1    seq
.....................ccguuguagucuaguuAg..........................................................................  6     1    seq
.....................ccguuguagucuagCugg..........................................................................  9     1    seq
.....................ccguuguaguGuaguuggu.........................................................................  1     1    seq
.....................ccguugAagucuaguuggu.........................................................................  1     1    seq
.....................ccguuguagucuaguuAgu.........................................................................  1     1    seq
.....................ccguuguagucuaguuggu.........................................................................  1885  0    seq
.....................ccUuuguagucuaguuggu.........................................................................  1     1    seq
.....................ccguuguUgucuaguuggu.........................................................................  1     1    seq
.....................ccguuguagucuaguugCu.........................................................................  1     1    seq
.....................ccguuguagucuaguAggu.........................................................................  7     1    seq
.....................ccguuguagucuaguGggu.........................................................................  2     1    seq
.....................ccgAuguagucuaguuggu.........................................................................  2     1    seq
.....................ccguuguagucuaguuggA.........................................................................  40    1    seq
.....................ccguuguCgucuaguuggu.........................................................................  1     1    seq
.....................ccguuguagucuaguCggu.........................................................................  3     1    seq
.....................Ncguuguagucuaguuggu.........................................................................  1     1    seq
.....................ccgCuguagucuaguuggu.........................................................................  8     1    seq
.....................ccguuguagucuaguuCgu.........................................................................  3     1    seq
.....................ccguuguGgucuaguuggu.........................................................................  3     1    seq
.....................ccguuguagucuaguugAu.........................................................................  1     1    seq
.....................ccguugCagucuaguuggu.........................................................................  3     1    seq
.....................ccguuguagucuagCuggu.........................................................................  9     1    seq
.....................ccguuguagucuaguuggC.........................................................................  504   1    seq
.....................ccguuAuagucuaguuggu.........................................................................  3     1    seq
.....................ccguuguagucuaguugguu........................................................................  174   0    seq
```

Mature   Star

```
auuuaaauauauuuggcaguuccguuguagucuaguugguuaguuucauugucugcucucacccgagagacccgguucaauuccugacaacggaaguuaaauauauuuuguag
.....................ccguuUuagucuaguugguu........................................................................ 1    1  seq
.....................ccguuguagucuaguAgguu........................................................................ 4    1  seq
.....................ccguuguagucuagCugguu........................................................................ 2    1  seq
.....................ccguuguagucuaguugAuu........................................................................ 3    1  seq
.....................ccguuguagucuaguugguA........................................................................ 9    1  seq
.....................ccguuguagucuaguuggCu........................................................................ 5    1  seq
.....................ccgAuguagucuaguugguua....................................................................... 1    1  seq
.....................ccguuguagucuaguugguua....................................................................... 93   0  seq
.....................ccguuguagucuaguCgguua....................................................................... 1    1  seq
.....................ccguuguagucuaguugguuU....................................................................... 2    1  seq
.....................ccguugCagucuaguugguua....................................................................... 1    1  seq
.....................ccguuguagucuaguAgguuag...................................................................... 2    1  seq
.....................ccguuguaguUuaguugguuag...................................................................... 1    1  seq
.....................ccguuguagucuaguugguuaC...................................................................... 1    1  seq
.....................ccguuguagucuGguugguuag...................................................................... 1    1  seq
.....................ccguuguagAcuaguugguuag...................................................................... 1    1  seq
.....................ccguuguagucuaguugguuag...................................................................... 90   0  seq
.....................ccguuguagucuaguCgguuag...................................................................... 1    1  seq
.....................ccguuguagucuaguugguuGg...................................................................... 1    1  seq
.....................ccguuguagucuaguugguuagA..................................................................... 4    1  seq
.....................ccguuguagucuaguugguuagu..................................................................... 2    0  seq
.....................ccguuguagucuaguugguCagu..................................................................... 1    1  seq
.....................ccguuguagucuaguugguuagGu.................................................................... 1    1  seq
......................cguuguGgucuaguugg.......................................................................... 3    1  seq
......................cgCuguagucuaguugg.......................................................................... 2    1  seq
......................cguuguagucuaguAgg.......................................................................... 5    1  seq
......................Nguuguagucuaguugg.......................................................................... 1    1  seq
......................cguuguagucuaguCgg.......................................................................... 1    1  seq
......................cguuguagucuagAugg.......................................................................... 1    1  seq
......................cguuguagucuaguugg.......................................................................... 999  0  seq
......................cguugCagucuaguugg.......................................................................... 7    1  seq
......................cguuguagucAaguugg.......................................................................... 1    1  seq
......................cgAuguagucuaguugg.......................................................................... 1    1  seq
......................cguuguagucuCguugg.......................................................................... 1    1  seq
......................cguuguagucuagCugg.......................................................................... 13   1  seq
......................cguuguagucuaguGgg.......................................................................... 1    1  seq
......................cguuguagucuaguuAg.......................................................................... 5    1  seq
......................cgGuguagucuaguugg.......................................................................... 1    1  seq
......................cguuguUgucuaguugg.......................................................................... 1    1  seq
......................cguuAuagucuaguugg.......................................................................... 1    1  seq
......................cguuguagucuaguugCu......................................................................... 1    1  seq
......................cguuguagGcuaguuggu......................................................................... 1    1  seq
......................cguuguagucGaguuggu......................................................................... 1    1  seq
......................cgAuguagucuaguuggu......................................................................... 1    1  seq
......................cguuguagucuaguuAgu......................................................................... 2    1  seq
......................cguuguagucuaguuggA......................................................................... 10   1  seq
......................cguuguagucuaguAggu......................................................................... 3    1  seq
......................cguuguagucuaguGggu......................................................................... 2    1  seq
......................cguuAuagucuaguuggu......................................................................... 1    1  seq
......................cguuguagucuagCuggu......................................................................... 5    1  seq
......................cgCuguagucuaguuggu......................................................................... 1    1  seq
......................cguuguGgucuaguuggu......................................................................... 2    1  seq
......................cguuguaUucuaguuggu......................................................................... 1    1  seq
......................cguuguagucuaguuggu......................................................................... 473  0  seq
......................cguugCagucuaguuggu......................................................................... 2    1  seq
......................cguuguagucuaguugUu......................................................................... 1    1  seq
......................cguuguagucuaguuggC......................................................................... 116  1  seq
......................cguuguagucuaguuggCu........................................................................ 1    1  seq
......................cguuguagucuaguGgguu........................................................................ 1    1  seq
......................cguuguagucuaguugguu........................................................................ 51   0  seq
......................cguuguagucuagCugguua....................................................................... 1    1  seq
......................cguuguagucuaguugguua....................................................................... 25   0  seq
......................cguuguagucuaguugguuag...................................................................... 36   0  seq
......................cguuguagucuagCugguuag...................................................................... 1    1  seq
......................cguuguagCcuaguugguuag...................................................................... 1    1  seq
......................cguuguagucuaguugguCagu..................................................................... 1    1  seq
.......................guuguagucuaguuggC......................................................................... 33   1  seq
.......................guAguagucuaguuggu......................................................................... 1    1  seq
.......................guuguagucuaguuggA......................................................................... 1    1  seq
.......................guuguagucuagCuggu......................................................................... 3    1  seq
```

Mature **Star**

```
auuuaaauauauuuggcaguuccguuguagucuaguugguuaguuucauugucugcucucacccgagagacccggguucaauuccugacaacggaaguuaaauauauuuuguag
.......................guuguagucuaguuggu..........................................................................	148	0	seq
.......................guuguagucuaguuUguu.........................................................................	1	1	seq
.......................guuguagucuaguugguu.........................................................................	21	0	seq
.......................guuguagucuaguugguA.........................................................................	1	1	seq
.......................guGguagucuaguugguua........................................................................	1	1	seq
.......................guuguagucuaguugguua........................................................................	4	0	seq
.......................guuguagucuaguugguuag.......................................................................	4	0	seq
.......................guuguagucuaguugguuagGu.....................................................................	1	1	seq
........................uuguagucuaguAgguu.........................................................................	1	1	seq
........................uuguagucuaguugguA.........................................................................	2	1	seq
........................uuguagucuaguugguu.........................................................................	70	0	seq
........................uNguagucuaguugguu.........................................................................	1	1	seq
........................Guguagucuaguugguu.........................................................................	1	1	seq
........................uuguaUucuaguugguu.........................................................................	1	1	seq
........................uuguagucuagCugguu.........................................................................	2	1	seq
........................uuguagucuagCugguua........................................................................	1	1	seq
........................uuguagucuaguugguua........................................................................	24	0	seq
........................uuguagucuaguugguuag.......................................................................	28	0	seq
........................uuguagucuaguugguuaC.......................................................................	1	1	seq
........................uuguagucuagCugguuag.......................................................................	3	1	seq
........................uuguagucuaguugguuagGu.....................................................................	1	1	seq
.........................uguagucuaguugguua........................................................................	3	0	seq
.........................uguagucuagCugguua........................................................................	2	1	seq
.........................uguagucuagCugguuag.......................................................................	1	1	seq
.........................uguagucuaguugguuag.......................................................................	19	0	seq
.........................uguagucuaguugguuagu......................................................................	1	0	seq
.........................uguagucuaguugguCagu......................................................................	1	1	seq
..........................guagucuaguugguuag.......................................................................	1	0	seq
......................................................gcucucacccgagagacccggg......................................	1	0	seq
......................................................gcucucacccgagagacccgggu.....................................	1	0	seq
.......................................................cucucacccgagagacccgg.......................................	1	0	seq
.......................................................cucucacccgagagacccggg......................................	1	0	seq
.......................................................cucucacccgagagacccAgg......................................	1	1	seq
.......................................................cucucacccgagagacccgggu.....................................	2	0	seq
.......................................................cucCcacccgagagacccgggu.....................................	1	1	seq
.......................................................cucucacccgGgagacccgggu.....................................	1	1	seq
.......................................................cucucacccgagagacccggguu....................................	1	0	seq
.......................................................cucucacccgagagacccggguC....................................	1	1	seq
.......................................................cucucacccgagagacccggguuc...................................	2	0	seq
........................................................ucucacccgagagacccg........................................	3	0	seq
........................................................ucucacccgagagacccgg.......................................	3	0	seq
........................................................ucucacccgagagacccggg......................................	2	0	seq
........................................................ucucacccgagagacccgggC.....................................	1	1	seq
........................................................ucucacccgagagacccgggu.....................................	2	0	seq
........................................................ucucaUccgagagacccgggu.....................................	1	1	seq
.........................................................cucacccgagagacccg........................................	20	0	seq
.........................................................cucacccgagagacccC........................................	1	1	seq
.........................................................cucacccgagagacccgg.......................................	13	0	seq
.........................................................cucacccgagagacccggC......................................	1	1	seq
.........................................................cucacccgagagacccggA......................................	1	1	seq
.........................................................cucacccgagagacccggg......................................	11	0	seq
.........................................................cucaUccgagagacccgggu.....................................	1	1	seq
.........................................................cucacccgagagacccgggu.....................................	52	0	seq
.........................................................cucacccgagagGcccgggu.....................................	1	1	seq
.........................................................cucacccgagagacccgggC.....................................	6	1	seq
.........................................................cucacccgagagacccgggG.....................................	1	1	seq
.........................................................cucacccgagagacccggguu....................................	3	0	seq
.........................................................cucacccgagagacccggguC....................................	1	1	seq
.........................................................cucacccgagagacccggguuc...................................	1	0	seq
.........................................................cucaUccgagagacccggguuc...................................	1	1	seq
..........................................................ucacccgagagacccgg.......................................	5	0	seq
..........................................................ucacccgagagacccggg......................................	6	0	seq
..........................................................Ccacccgagagacccgggu.....................................	1	1	seq
..........................................................ucacccgagagacccgggu.....................................	2	0	seq
..........................................................ucacccgagagacccggAuu....................................	1	1	seq
..........................................................Acacccgagagacccggguuc...................................	1	1	seq
..........................................................ucaUccgagagacccggguuc...................................	1	1	seq
..........................................................ucaUccgagagacccggguuca..................................	2	1	seq
...........................................................cacccgagagacccggC......................................	1	1	seq
...........................................................cacccgagagacccggg......................................	1	0	seq
```

Mature Star

```
auuuaaauauauuuggcaguuccguuguagucuaguugguuaguuucauugucugcucucacccgagagacccggguucaauuccugacaacggaaguuaaauauauuuuguag
...........................................................cacccgagagacccggA......................................   1    1    seq
...........................................................caUccgagagacccgggu.....................................   1    1    seq
...........................................................cacccgagagacccgggu.....................................   2    0    seq
...........................................................caUccgagagacccggguu....................................   1    1    seq
...........................................................cacccgagagacccggguuc...................................   1    0    seq
...........................................................caUccgagagacccggguuc...................................   34   1    seq
...........................................................caUccgagagacccggguuca..................................   2    1    seq
............................................................acccgagagUcccgggu.....................................   1    1    seq
............................................................aAccgagagacccgggu.....................................   1    1    seq
............................................................aUccgagagacccgggu.....................................   2    1    seq
............................................................aUccgagagacccggguuc...................................   6    1    seq
.............................................................cccgagagacccggguu....................................   9    0    seq
.............................................................cccgagagacccAgguu....................................   1    1    seq
.............................................................cccgagagacccggguC....................................   2    1    seq
.............................................................cccgagagacccggguuU...................................   3    1    seq
.............................................................cccgagagacccggguuA...................................   1    1    seq
.............................................................cccUagagacccggguuc...................................   1    1    seq
.............................................................cccgagagacccgggGuc...................................   1    1    seq
.............................................................cccgagagacUcggguuc...................................   1    1    seq
.............................................................cccgagagacccggguGc...................................   1    1    seq
.............................................................cccgagagacccUgguuc...................................   1    1    seq
.............................................................cccgagagacccAgguuc...................................   2    1    seq
.............................................................Uccgagagacccggguuc...................................   8    1    seq
.............................................................cccgagagacccggguuc...................................   58   0    seq
.............................................................cccgagagacccggguCc...................................   1    1    seq
.............................................................cccgagagacccggguuca..................................   8    0    seq
..............................................................ccgGgagacccggguuc...................................   1    1    seq
..............................................................ccgagagacccggguuU...................................   29   1    seq
..............................................................ccgagagacccggguGc...................................   4    1    seq
..............................................................ccgagagacccggguCc...................................   7    1    seq
..............................................................ccgagagacccggguuc...................................   758  0    seq
..............................................................ccgagagacAcggguuc...................................   1    1    seq
..............................................................ccgagagaGccggguuc...................................   2    1    seq
..............................................................ccgagGgacccggguuc...................................   2    1    seq
..............................................................ccgagagacccggguAc...................................   1    1    seq
..............................................................ccgagagacccAgguuc...................................   2    1    seq
..............................................................ccgagagacccgggGuc...................................   2    1    seq
..............................................................Ncgagagacccggguuc...................................   1    1    seq
..............................................................ccgagagacccgggCuc...................................   1    1    seq
..............................................................ccgagagacccgggAuc...................................   2    1    seq
..............................................................ccgagagacccUgguuc...................................   2    1    seq
..............................................................ccgagUgacccggguuc...................................   1    1    seq
..............................................................Acgagagacccggguuc...................................   1    1    seq
..............................................................ccgagagacUcggguuc...................................   1    1    seq
..............................................................ccgagagaccGggguuc...................................   3    1    seq
..............................................................ccgagagGcccggguuc...................................   1    1    seq
..............................................................ccgagagacccggguuA...................................   8    1    seq
..............................................................ccgagagacccggguuca..................................   63   0    seq
..............................................................ccgagagacccggguucU..................................   1    1    seq
..............................................................ccgagagacccgggGuca..................................   1    1    seq
..............................................................ccgagagacccUgguuca..................................   1    1    seq
...............................................................cgagagacccggguuca..................................   295  0    seq
...............................................................cgaUagacccggguuca..................................   1    1    seq
...............................................................cgagagacccggguucC..................................   2    1    seq
...............................................................cgagagacUcggguuca..................................   1    1    seq
...............................................................cgagagacccgggGuca..................................   1    1    seq
...............................................................cgagagacccggguucU..................................   2    1    seq
...............................................................cgagGgacccggguuca..................................   3    1    seq
...............................................................cgagagacccggguuGa..................................   1    1    seq
...............................................................cgagagacccggguCca..................................   2    1    seq
...............................................................Ggagagacccggguuca..................................   1    1    seq
...............................................................cgagagacccgggCuca..................................   1    1    seq
...............................................................cgagagGcccggguuca..................................   1    1    seq
...............................................................cgagagacccggguucaG.................................   1    1    seq
```

Provisional ID	:	MA_152091_13509
Score total	:	1.4
Score for star read(s)	:	-1.3
Score for read counts	:	0
Score for mfe	:	1.1
Score for randfold	:	1.6
Score for cons. seed	:	
Total read count	:	60
Mature read count	:	43
Loop read count	:	0
Star read count	:	17

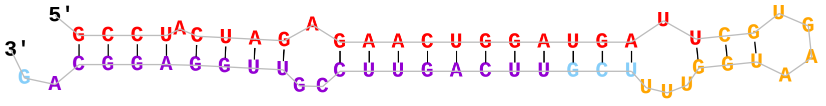

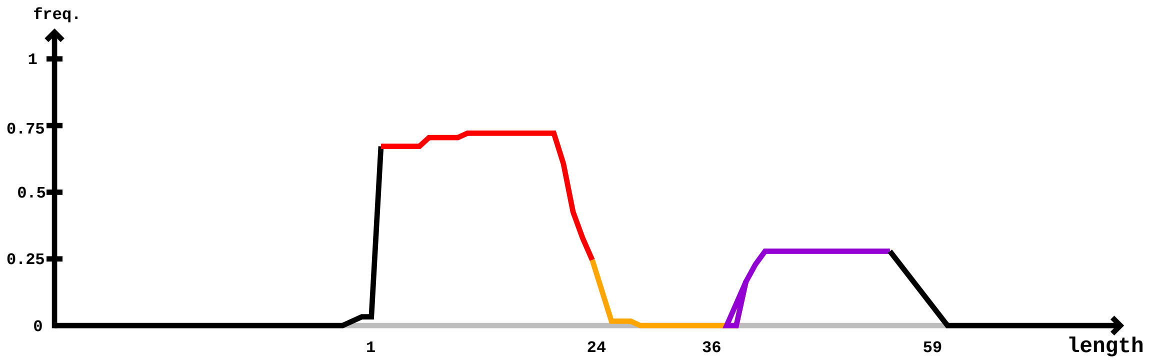

Mature Star

```
5'- gauauugaucuguuugucugguauugcauauugccuacuagagaacuggaugauucgugaaugguuuucguucaguuccguuggaggcagagaguacagaucccuguuu  -3'  obs
    gauauugaucuguuugucugguauugcauauugccuacuagagaacuggaugauucgugaaugguuuucguucaguuccguuggaggcagagaguacagaucccuguuu       exp
    ((((..(((((((...(((...........((((((.((((.(((((((((((.(((....)))...)))))))))))..)))))))))))))..)))))))..)))).  reads  mm  sample
    .............................auugccuUcuagagaacuggaugauu......................................................  1  1  seq
    ..............................uCgccuacuagagaacuggaugau.......................................................  1  1  seq
    ................................gccuUcuagagaacuggau..........................................................  7  1  seq
    ................................gccuUcuagagaacuggaug.........................................................  11  1  seq
    ................................gccuUcuagagaacuggauga........................................................  6  1  seq
    ................................gccuUcuagagaacuggaugau.......................................................  4  1  seq
    ................................gccuUcuagagaacuggaugauu......................................................  11  1  seq
    .....................................cuagagaacuggaugauu......................................................  2  0  seq
    .........................................agaacuggaugauucgCg..................................................  1  1  seq
    ......................................................................uucaguuccguuggaggU.....................  1  1  seq
    ......................................................................uucaguuccguuggaggc.....................  1  0  seq
    ......................................................................uucaguuccguuggaggca....................  8  0  seq
    .......................................................................ucaguuccguuggaggU.....................  1  1  seq
    .......................................................................ucaguuccguuggaggc.....................  1  0  seq
    .......................................................................ucaguuccguuggaggca....................  2  0  seq
    ........................................................................caguuccguuggaggca....................  3  0  seq
```

Provisional ID	:	MA_152834_3570
Score total	:	213.1
Score for star read(s)	:	3.9
Score for read counts	:	217.8
Score for mfe	:	-6.3
Score for randfold	:	-2.2
Score for cons. seed	:	
Total read count	:	439
Mature read count	:	436
Loop read count	:	0
Star read count	:	3

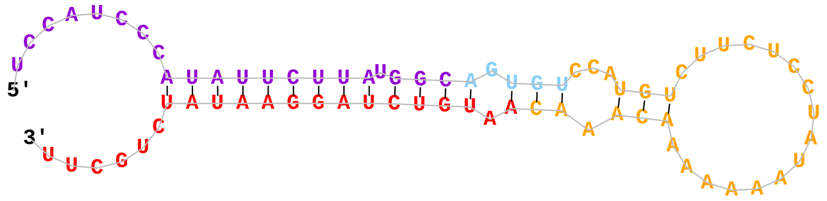

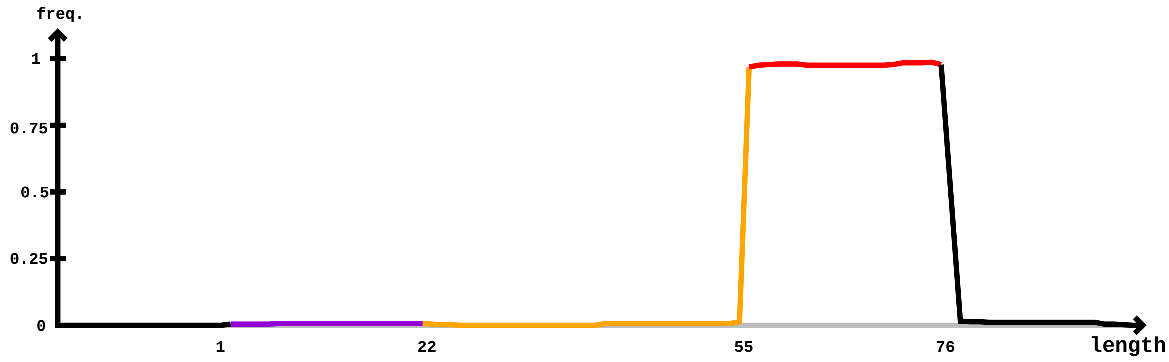


```
                    Star                                   Mature
5'- auucaccccaucuagauccaucccauauucuuauggcaguguccaugucuucuccuauaaaaaaacaaacaaugucuaggaauaucugcuucucccauggauugagacugu   -3'   obs
    auucaccccaucuagauccaucccauauucuuauggcaguguccaugucuucuccuauaaaaaaacaaacaaugucuaggaauaucugcuucucccauggauugagacugu         exp
    .((((..((((..(((..((....(((((((((.((((.(((...(((................))).))).))))))))))))).))..)))...))))..)))).....   reads   mm   sample
    ................uccaucccauauGcuuauggc..........................................................................   2       1    seq
    ....................cccauauucuuauUgcagu........................................................................   1       1    seq
    .......................................................uauaaaaaaacGaacaauguc...................................   2       1    seq
    .......................................................uauGaaaaaacaaacaauguc...................................   1       1    seq
    .....................................................................caaugucuagAaauaucugcu.....................   1       1    seq
    .....................................................................Gaaugucuaggaauaucugcuu....................   2       1    seq
    ......................................................................aaugucuaggaauaucugcu.....................   2       0    seq
    ......................................................................aaugucuaggaauaucuAcu.....................   1       1    seq
    ......................................................................aaCgucuaggaauaucugcuu....................   2       1    seq
    ......................................................................Gaugucuaggaauaucugcuu....................   6       1    seq
    ......................................................................aaugucuaUgaauaucugcuu....................   54      1    seq
    ......................................................................aaugucuaggaaCaucugcuu....................   1       1    seq
    ......................................................................aaugucuaCgaauaucugcuu....................   17      1    seq
    ......................................................................aaugucuaggaGuaucugcuu....................   1       1    seq
    ......................................................................aaugucuaggaauaucugcCu....................   3       1    seq
    ......................................................................aaugCcuaggaauaucugcuu....................   1       1    seq
    ......................................................................aaugucuaggaauaucugcuu....................   284     0    seq
    ......................................................................aaugucuaggaauaucugcuC....................   43      1    seq
    ......................................................................aaugucuaggaauaucugcuG....................   8       1    seq
    ......................................................................aGugucuaggaauaucugcuu....................   1       1    seq
    ......................................................................aaugucCaggaauaucugcuu....................   1       1    seq
    ......................................................................aaugucuaggaauaucCgcuu....................   1       1    seq
    ......................................................................aaAgucuaggaauaucugcuu....................   1       1    seq
    .......................................................................augucuaggaauaucugcuu....................   3       0    seq
    ........................................................................ugucuaggaauauUugcuucu..................   1       1    seq
    .........................................................................gucuaUgaauaucugcuu....................   1       1    seq
    ............................................................................uaggaauaucugcuucucc................   1       0    seq
    .....................................................................................Augcuucucccauggauugagacu..   1       1    seq
    ......................................................................................ugcuucucccGuggauugaga....   3       1    seq
    .........................................................................................uucucccGuggauugagacug.   1       1    seq
```

Provisional ID	:	MA_155935_13901
Score total	:	1.8
Score for star read(s)	:	-1.3
Score for read counts	:	0
Score for mfe	:	1.5
Score for randfold	:	1.6
Score for cons. seed	:	
Total read count	:	58536
Mature read count	:	58536
Loop read count	:	0
Star read count	:	0

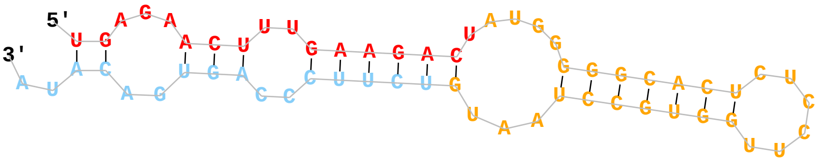

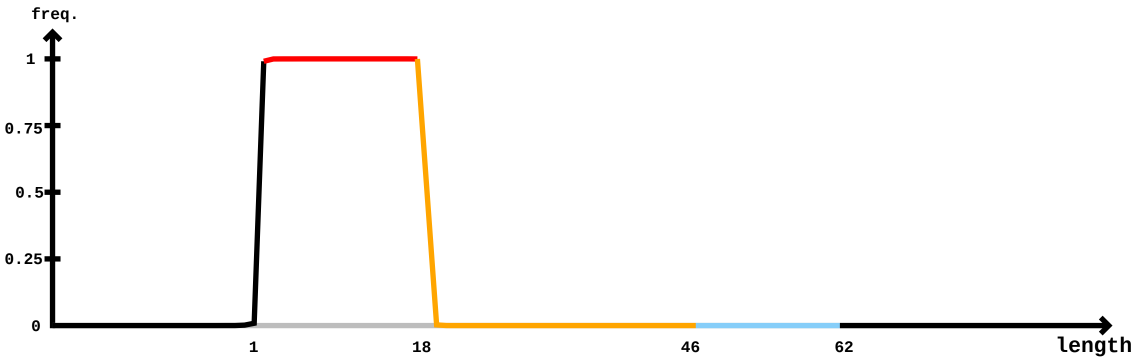

Mature **Star**

```
5'- uggaguauaucacaacagugugagaacuuugaagacuaugggggcacucuccuuggugccuaaugucuucccagugacauacugugacauagauccacauuuaucuuc -3'   exp
    ((((...(((....((((((((...(((..((((((.....(((((((......)))))))...))))))..)))..))))))))...)))..))))...........   reads   mm   sample
    ...............caUugugagaacuuugaagacua......................................................................   1       1    seq
    ................agGgugagaacuuugaaga.........................................................................   1       1    seq
    ................aUugugagaacuuugaagac........................................................................   1       1    seq
    .................gCgugagaacuuugaagacu.......................................................................   1       1    seq
    .................guCugagaacuuugaagacu.......................................................................   1       1    seq
    ..................Agugagaacuuugaagac........................................................................   4       1    seq
    ..................Agugagaacuuugaagacu.......................................................................   76      1    seq
    ..................Cgugagaacuuugaagacu.......................................................................   1       1    seq
    ..................Ngugagaacuuugaagacu.......................................................................   2       1    seq
    ..................Ggugagaacuuugaagacu.......................................................................   2       1    seq
    ..................Ngugagaacuuugaagacua......................................................................   1       1    seq
    ..................Agugagaacuuugaagacua......................................................................   2       1    seq
    ..................uAugagaacuuugaagacua......................................................................   2       1    seq
    ...................gugagaacuuugaagac........................................................................   5       0    seq
    ...................Nugagaacuuugaagac........................................................................   3       1    seq
    ...................gugGgaacuuugaagacu.......................................................................   2       1    seq
    ...................gugagaacuuugaagacG.......................................................................   4       1    seq
    ...................gugagaacuuuUaagacu.......................................................................   1       1    seq
    ...................gugagaacAuugaagacu.......................................................................   1       1    seq
    ...................gugagaacuuugaagacu.......................................................................   133     0    seq
    ...................Cugagaacuuugaagacu.......................................................................   6       1    seq
    ...................Nugagaacuuugaagacu.......................................................................   36      1    seq
    ...................gugagGacuuugaagacu.......................................................................   2       1    seq
    ...................gugagaacuuugaagacA.......................................................................   3       1    seq
    ...................gugagaacuuugUagacu.......................................................................   1       1    seq
    ...................Nugagaacuuugaagacua......................................................................   2       1    seq
    ...................gugagaacAuugaagacua......................................................................   1       1    seq
    ...................Cugagaacuuugaagacua......................................................................   2       1    seq
    ...................gugagaacuuugaagacua......................................................................   10      0    seq
    ...................gugagaacuuugaagacuG......................................................................   197     1    seq
    ...................gugagaacuuugaagacuU......................................................................   2       1    seq
    ....................ugagaUcuuugaagacu.......................................................................   1       1    seq
    ....................ugagaacuGugaagacu.......................................................................   23      1    seq
    ....................ugagaGcuuugaagacu.......................................................................   127     1    seq
```

Mature Star

```
uggaguauaucacaacagugugagaacuuugaagacuaugggggcacucuccuuggugccuaaugucuucccagugacauacugugacauagauccacauuuaucuuc
....................Ggagaacuuugaagacu.......................................................................	80	1	seq
....................ugCgaacuuugaagacu.......................................................................	8	1	seq
....................ugagaacuuugaGgacu.......................................................................	219	1	seq
....................ugagaacGuugaagacu.......................................................................	5	1	seq
....................ugagaacuuCgaagacu.......................................................................	122	1	seq
....................ugagaacuuugaagacu.......................................................................	52535	0	seq
....................ugagaacuuugCagacu.......................................................................	4	1	seq
....................ugagaacuuugaCgacu.......................................................................	10	1	seq
....................ugagaacuuuCaagacu.......................................................................	9	1	seq
....................uNagaacuuugaagacu.......................................................................	1	1	seq
....................ugagaacuuuUaagacu.......................................................................	8	1	seq
....................uAagaacuuugaagacu.......................................................................	34	1	seq
....................Ngagaacuuugaagacu.......................................................................	64	1	seq
....................uUagaacuuugaagacu.......................................................................	26	1	seq
....................uCagaacuuugaagacu.......................................................................	2	1	seq
....................ugagGacuuugaagacua......................................................................	17	1	seq
....................uAagaacuuugaagacua......................................................................	4	1	seq
....................ugagaacuuugaUgacua......................................................................	3	1	seq
....................ugagaacuuugaGgacua......................................................................	20	1	seq
....................Ngagaacuuugaagacua......................................................................	4	1	seq
....................ugagaGcuuugaagacua......................................................................	9	1	seq
....................ugagaacuuugaagacua......................................................................	3913	0	seq
....................ugagaacuuugGagacua......................................................................	23	1	seq
....................ugUgaacuuugaagacua......................................................................	2	1	seq
....................ugagCacuuugaagacua......................................................................	3	1	seq
....................ugagaacuuugaagacGa......................................................................	21	1	seq
....................uUagaacuuugaagacua......................................................................	2	1	seq
....................ugagaacuuuCaagacua......................................................................	1	1	seq
....................ugaUaacuuugaagacua......................................................................	1	1	seq
....................ugagaacuGugaagacua......................................................................	1	1	seq
....................ugagaacuuuUaagacua......................................................................	1	1	seq
....................ugagaacuuugaaAacua......................................................................	1	1	seq
....................ugaCaacuuugaagacua......................................................................	2	1	seq
....................ugagaacAuugaagacua......................................................................	1	1	seq
....................ugagaacuuCgaagacua......................................................................	7	1	seq
....................Cgagaacuuugaagacua......................................................................	7	1	seq
....................Ggagaacuuugaagacua......................................................................	7	1	seq
....................ugagaacuuugaagacCa......................................................................	53	1	seq
....................ugGgaacuuugaagacua......................................................................	16	1	seq
....................ugagaacuuugaagacuaG.....................................................................	2	1	seq
....................ugagaacuuugaagacuGu.....................................................................	106	1	seq
....................ugagaacuuugaagacuau.....................................................................	1	0	seq
....................ugagaacuuugaagacGau.....................................................................	1	1	seq
....................ugagaacuuugaagacuauA....................................................................	1	1	seq
....................ugagaacuuugaagacuGug....................................................................	1	1	seq
.....................gagaacuuugaGgacua......................................................................	3	1	seq
.....................gagGacuuugaagacua......................................................................	2	1	seq
.....................gGgaacuuugaagacua......................................................................	1	1	seq
.....................gagaacuuugaagacCa......................................................................	6	1	seq
.....................gagaacuuugGagacua......................................................................	1	1	seq
.....................gagaacuuugaagacGa......................................................................	3	1	seq
.....................gagaCcuuugaagacua......................................................................	1	1	seq
.....................gagaGcuuugaagacua......................................................................	1	1	seq
.....................gagaacuuugaagacua......................................................................	467	0	seq
.....................Nagaacuuugaagacua......................................................................	1	1	seq
.....................Cagaacuuugaagacua......................................................................	1	1	seq
.....................gagaacuuugaagacuGu.....................................................................	23	1	seq
......................agaacuuugaagacuGu.....................................................................	15	1	seq
........................aaAuuugaagacuaugg...................................................................	1	1	seq
.........................acuuugaagacuGugggg.................................................................	1	1	seq
................................................................................acugugacauagauccacauu.......	1	0	seq
```

Provisional ID	:	MA_156101_21566
Score total	:	1.5
Score for star read(s)	:	-1.3
Score for read counts	:	0
Score for mfe	:	1.2
Score for randfold	:	1.6
Score for cons. seed	:	
Total read count	:	36
Mature read count	:	36
Loop read count	:	0
Star read count	:	0

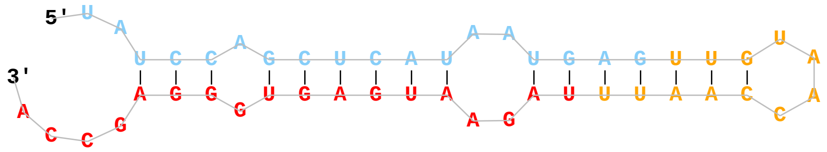

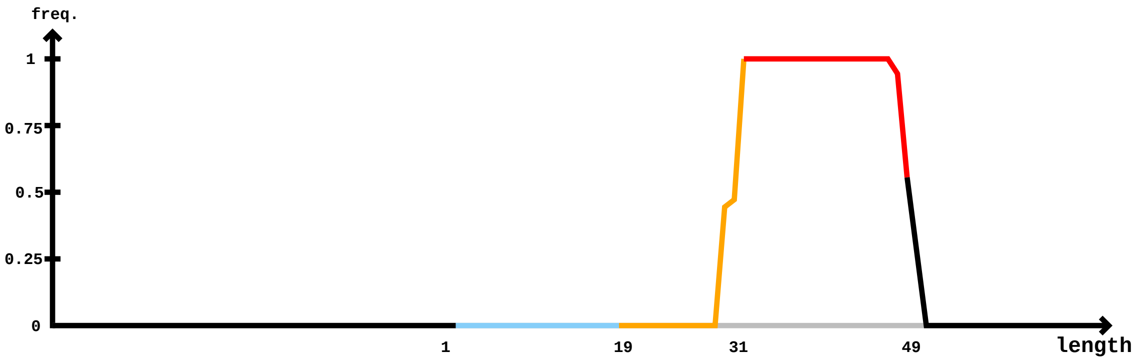

Star Mature

```
5'- gaucccucaaaauccagaaaaacuaguggagcaugaguuguauccagcucauaaugaguuguaaccaauuuagaaugagugggagccaacucacuuuaagauggaaag   -3'   exp
    ..(((.((....((((..........))))...(((((((..(((.((((((..(((((((....)))))))..)))))).)))..)))))))......)).)))...   reads   mm   sample
    ....................................................................uGuagaaugagugggagc......................   2   1   seq
    ....................................................................uGuagaaugagugggagcc.....................   5   1   seq
    ....................................................................uuuagaauAagugggagcc.....................   5   1   seq
    ....................................................................uGuagaaugagugggagcca....................   4   1   seq
    .....................................................................Guagaaugagugggagcc.....................   1   1   seq
    ......................................................................uagaaugagugggagcc.....................   2   0   seq
    ......................................................................uagaaugaAugggagcc.....................   1   1   seq
    ......................................................................uagaaugagugggagccG....................   9   1   seq
    ......................................................................uagaaugagugggagcca....................   5   0   seq
    ......................................................................uagaaugagugggagccU....................   2   1   seq
```

Provisional ID : MA_156628_7498
Score total : 0
Score for star read(s) : -1.3
Score for read counts : 0
Score for mfe : -0.2
Score for randfold : 1.6
Score for cons. seed :
Total read count : 5323
Mature read count : 5312
Loop read count : 11
Star read count : 0

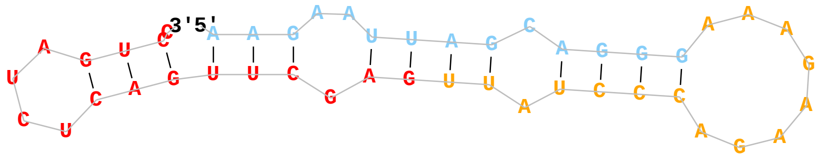

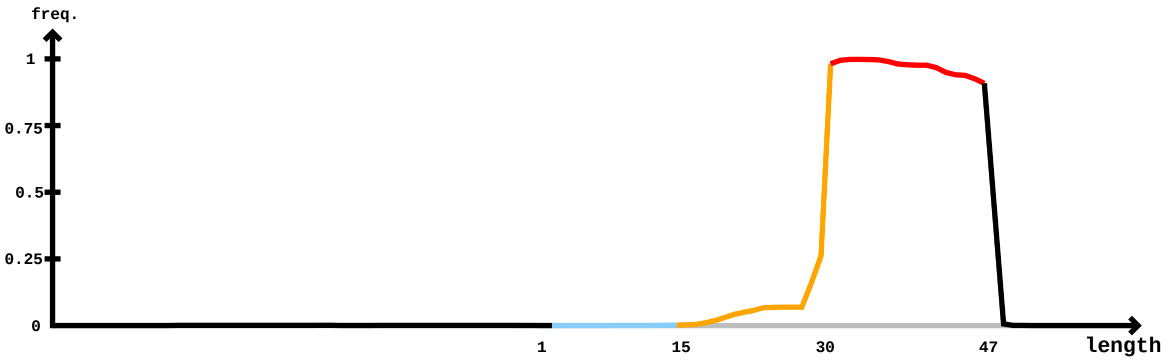

Star Mature

```
5'- cugucccuagauacuaucuagagaaacuacauccaagggaaugggcuuggaagaauuagcagggaaagaagacccuauugagcuugacucuaguccuaacccauucauagu -3'   exp
    ...((.((((((...)))))).)).............(((((((...((((((.(((((((((........))))).....)).))).))).)))...))))))).....   reads   mm   sample
    ...........uacuaucuagCgaaacu...................................................................................   4   1   seq
    ..........................cuacauccaagggaaCgggcu................................................................   1   1   seq
    ................................ccaagggaaugggcuug..............................................................   2   0   seq
    .........................................................agcagggaaagaagacc.....................................   2   0   seq
    .............................................................gggaaagaagacccuCu.................................   1   1   seq
    .............................................................gggaaagaagacccuauugagcuugacucu....................   1   0   seq
    ..............................................................ggaaagaagacccuauC................................   1   1   seq
    ..............................................................ggaaagaagacccuCuugagcuugacuc.....................   1   1   seq
    ...............................................................gaaagaagacccuauugagcuug.........................   1   0   seq
    ...............................................................gaaagaagacccuauugagcuugacucCa...................   1   1   seq
    ...............................................................gaaagaagacccuauugagcuugacucuaguc................   1   0   seq
    ................................................................aaagaagacccuauugagcuC..........................   1   1   seq
    ................................................................aaagaagacccuauugagcuug.........................   2   0   seq
    ................................................................aaagaagacccuauugagcuugacucuagu.................   1   0   seq
    .................................................................aagaagacccuCuugagcu...........................   1   1   seq
    .................................................................aagaagacccuauugagcuu..........................   3   0   seq
    .................................................................aagaagacccuauugagcuug.........................   2   0   seq
    .................................................................aagaagacccuauugagcuugacucu....................   5   0   seq
    ..................................................................agaagacccuauugagU............................   2   1   seq
    ..................................................................agaagacccuauugagc............................   1   0   seq
    ..................................................................agaagacccuauugagcu...........................   1   0   seq
    ..................................................................agaagacccuauugagcuu..........................   4   0   seq
    ..................................................................agaagacccuauugagcuC..........................   2   1   seq
    ..................................................................agaagacccuCuugagcuug.........................   1   1   seq
    ..................................................................agaagacccuauugagcuug.........................   7   0   seq
    ..................................................................agaagacccuauugagcuuA.........................   1   1   seq
    ..................................................................agaagacccuCuugagcuuga........................   1   1   seq
    ..................................................................agaagacccuauugagcuugacuU.....................   1   1   seq
    ..................................................................agaagacccuauugagcuugacucu....................   6   0   seq
    ..................................................................agaagacccuCuugagcuugacucu....................   2   1   seq
    ..................................................................agaagacccuCuugagcuugacucua...................   1   1   seq
    ..................................................................agaagacccuauugagcuugacucua...................   2   0   seq
    ..................................................................agaagacccuauugagcuugacucuag..................   1   0   seq
    ..................................................................agaagacccuauugagcuugacucuagu.................   2   0   seq
```

Star Mature

cugucccuagauacuaucuagagaaacuacauccaagggaaugggcuuggaagaauuagcagggaaagaagacccuauugagcuugacucuaguccuaacccauucauagu			
....agaagacccuauugagcuugacucuaguc................	1	0	seq
....agaagacccuauugagcuugacucuaguccGa.............	2	1	seq
....gaagacccuauugagcu...........................	3	0	seq
....gaagacccuauugagcC...........................	1	1	seq
....gaagacccuauugGgcu...........................	1	1	seq
....gaagacccuauugagcuC..........................	1	1	seq
....gaagacccuauugagcuu..........................	5	0	seq
....gaagacccuauugagcuug.........................	9	0	seq
....gaagacccuauugagcuGg.........................	1	1	seq
....gaagacccuauugagcuuga........................	1	0	seq
....gaagacccuauugagcuugac.......................	1	0	seq
....gaagacccuauugagcuugacuc.....................	3	0	seq
....gaagacccuNuugagcuugacucu....................	2	1	seq
....gaagacccuauugagcuugacucu....................	7	0	seq
....gaagacccuauugagcuugacucua...................	7	0	seq
....gaagacccuauugagcuugacucuagu.................	2	0	seq
....gaGgacccuauugagcuugacucuagu.................	1	1	seq
....gaagacccuauugagcuugacucuaguc................	1	0	seq
....gaagacccuauugagcuugacucuagucc...............	1	0	seq
....gaagacccuauugagcuugacucuaguccGa.............	1	1	seq
....aagacccuauugagcuC..........................	1	1	seq
....aagacccuauugagcuu..........................	17	0	seq
....aagacccuCuugagcuu..........................	2	1	seq
....aagacccuauugagcuug.........................	16	0	seq
....aagacccuauugagcuuga........................	3	0	seq
....aagacccuauugagcuugacuc.....................	6	0	seq
....aagacccuCuugagcuugacuc.....................	1	1	seq
....aagacccuauugagcCugacucu....................	1	1	seq
....aagacccuNuugagcuugacucu....................	1	1	seq
....aagacccuauugagcuugacucu....................	5	0	seq
....aagacccuCuugagcuugacucu....................	2	1	seq
....aagacccuauugagcuugacucA....................	1	1	seq
....aagacccuauugagcuugacucC....................	2	1	seq
....aagacccuauugagcuugacucua...................	3	0	seq
....aagacccuCuugagcuugacucua...................	1	1	seq
....aagacccuauugagcuugacucuag..................	1	0	seq
....aagacccuauugagcuugacucuagu.................	1	0	seq
....aagacccuauugagcuugacucuaguccGa.............	1	1	seq
....agacccuauugagcuug.........................	13	0	seq
....agacccuauugagcuuga........................	7	0	seq
....agacccuauugagcuugac.......................	3	0	seq
....agacccuauugagcuugacuU.....................	2	1	seq
....agacccuauugagcuugacuc.....................	7	0	seq
....agacccuauugagcuugacucC....................	3	1	seq
....agacccuCuugagcuugacucu....................	1	1	seq
....agacccuauugagcuugaAucu....................	1	1	seq
....agacccuauugagcuugacucu....................	13	0	seq
....agacccuauugagcuugacucua...................	7	0	seq
....agacccuauugagcuugacucuag..................	3	0	seq
....agacccuauugagcuugacucuagC.................	1	1	seq
....agacccuauugagcuugacucuagu.................	3	0	seq
....agacccuauugagcuugacucuaguc................	2	0	seq
....agacccuUuugagcuugacucuagucc...............	2	1	seq
....gacccuauugagcuuga........................	5	0	seq
....gacccuauugagcuugac.......................	2	0	seq
....gacccuCuugagcuugac.......................	1	1	seq
....gacccuauugagcuugacuc.....................	1	0	seq
....gacccuauugagcuugacuA.....................	1	1	seq
....gacccuCuugagcuugacucu....................	1	1	seq
....gacccuauugagcuugacucu....................	9	0	seq
....gacccuauugagcuGgacucu....................	1	1	seq
....gacccuauugagcuugacucC....................	4	1	seq
....gacccuCuugagcuugacucua...................	1	1	seq
....gacccuauugagcuugacucua...................	5	0	seq
....gacccuauugagcuCgacucua...................	1	1	seq
....gacccuauugagcuugacucuag..................	3	0	seq
....gacccuauugagcuugacucuagu.................	2	0	seq
....gacccuCuugagcuugacucuagu.................	1	1	seq
....gacccuauugagcuugacucuagC.................	1	1	seq
....gacccuauugagcuugacucuagucc...............	2	0	seq

Star Mature

cugucccuagauacuaucuagagaaacuacauccaagggaaugggcuuggaagaauuagcagggaaagaagacccuauugagcuugacucuaguccuaacccauucauagu			
....gacccuUuugagcuugacucuagucc...............	1	1	seq
....acccuauugagcuugac.......................	1	0	seq
....acccuauugagcuugacu......................	1	0	seq
....acccuauugagcuugacuc.....................	6	0	seq
....acccuCuugagcuugacuc.....................	1	1	seq
....acccuauugagcuugacucA....................	1	1	seq
....acccuauugagcuugacucu....................	14	0	seq
....acccuauugagcuugacucC....................	1	1	seq
....acccuauugagcuugacucua...................	6	0	seq
....acccuauugagcuugacucuag..................	4	0	seq
....acccuauugagcuugacucuagu.................	4	0	seq
....acccuauugagcuugacucuagucc...............	1	0	seq
....cccuauugagcuugacuc.....................	17	0	seq
....cccuCuugagcuugacuc.....................	1	1	seq
....cccuauugagcuuUacuc.....................	1	1	seq
....cccuaCugagcuugacuc.....................	1	1	seq
....cccuauugagcAugacucu....................	1	1	seq
....cccuauugagcuugacucu....................	13	0	seq
....cccuauugagUuugacucu....................	1	1	seq
....cccuauugagcuugacCcu....................	1	1	seq
....cccuCuugagcuugacucua...................	1	1	seq
....cccuauugagcuugacucua...................	11	0	seq
....cccuauugagcCugacucua...................	1	1	seq
....cccuauugagcuugacucuag..................	1	0	seq
....cccuauugagcuugacucuagu.................	5	0	seq
....cccuauugagcuugacucuaguc................	1	0	seq
....cccuauugagcuugacucuagucc...............	1	0	seq
....cccuUuugagcuugacucuagucc...............	1	1	seq
....ccuauugagcuugacuc.....................	1	0	seq
....ccuauugagcuugacucu....................	1	0	seq
....ccuauugagcuugacucua...................	1	0	seq
....ccuauugagcuugacucuagu.................	1	0	seq
....ccuauugagcuugacucuaguc................	1	0	seq
....ccuUuugagcuugacucuagucc...............	1	1	seq
....cuauugagcuugacucu....................	2	0	seq
....cuauugagcuugacucua...................	1	0	seq
....cuauugagcuugacucuag..................	1	0	seq
....cuauugagcuugacucuagu.................	1	0	seq
....cuauugagcuugacucuaguc................	1	0	seq
....cuauugagcuugacucuagucc...............	1	0	seq
....Nuugagcuugacucuagucc...............	1	1	seq
....uugagcuugacucuagG.................	2	1	seq
....uugagcuugacucuagu.................	45	0	seq
....Cugagcuugacucuaguc................	1	1	seq
....uugagcuugacucuaguc................	43	0	seq
....uugagcuugacucuagucG...............	3	1	seq
....Augagcuugacucuagucc...............	1	1	seq
....Cugagcuugacucuagucc...............	3	1	seq
....Gugagcuugacucuagucc...............	1	1	seq
....uugagcuugacucAagucc...............	2	1	seq
....uugagcAugacucuagucc...............	1	1	seq
....uugagcuugacucuagucc...............	408	0	seq
....uugagcuugacucuaguccC..............	5	1	seq
....uugagcuugacucuaguccu..............	6	0	seq
....ugagcuugacucuaguc................	41	0	seq
....uUagcuugacucuaguc................	1	1	seq
....ugagcAugacucuaguc................	1	1	seq
....uCagcuugacucuaguc................	1	1	seq
....ugagcuugacucuagucG...............	1	1	seq
....Agagcuugacucuagucc...............	2	1	seq
....ugagcuugacucuaguGc...............	1	1	seq
....uUagcuugacucuagucc...............	1	1	seq
....ugagcuCgacucuagucc...............	1	1	seq
....Ngagcuugacucuagucc...............	1	1	seq
....ugagcuugacucuagucc...............	519	0	seq
....ugagcuugacucuaguccu..............	5	0	seq
....ugagcuugacucuaguccC..............	4	1	seq
....ugagcuugacucuaguccCa.............	2	1	seq
....gagcuCgacucuagucc...............	14	1	seq
....gagcGugacucuagucc...............	3	1	seq

```
                                                  Star                         Mature
cugucccuagauacuaucuagagaaacuacauccaagggaaugggcuuggaagaauuagcagggaaagaagacccuauugagcuugacucuaguccuaacccauucauagu
...............................................................................gagcuugUcucuagucc...............  1     1    seq
...............................................................................Uagcuugacucuagucc...............  13    1    seq
...............................................................................Nagcuugacucuagucc...............  8     1    seq
...............................................................................gagcuugacuGuagucc...............  2     1    seq
...............................................................................gagcuugacucuagucc...............  3987  0    seq
...............................................................................gagcuugacucGagucc...............  1     1    seq
...............................................................................gagcuugacucuagucG...............  18    1    seq
...............................................................................gagUuugacucuagucc...............  9     1    seq
...............................................................................gagcuugacGcuagucc...............  1     1    seq
...............................................................................Cagcuugacucuagucc...............  2     1    seq
...............................................................................gUgcuugacucuagucc...............  1     1    seq
...............................................................................gCgcuugacucuagucc...............  2     1    seq
...............................................................................gagcAugacucuagucc...............  2     1    seq
...............................................................................gagcuugacucuagGcc...............  5     1    seq
...............................................................................gagcuAgacucuagucc...............  1     1    seq
...............................................................................gagcuGgacucuagucc...............  2     1    seq
...............................................................................gagcuugGcucuagucc...............  13    1    seq
...............................................................................gagcuugGcucuaguccu..............  1     1    seq
...............................................................................gagcuugacucuaguccu..............  29    0    seq
...............................................................................gagcuugacucuagucGu..............  1     1    seq
................................................................................agcuugGcucuaguccu..............  2     1    seq
................................................................................agcuugacucuCguccu..............  1     1    seq
................................................................................agcuugacucuGguccu..............  2     1    seq
................................................................................agcuugacucuaguccu..............  58    0    seq
................................................................................Ggcuugacucuaguccu..............  1     1    seq
................................................................................agcuugacucuaguccua.............  1     0    seq
................................................................................agcuugacucuaguccCa.............  9     1    seq
.................................................................................gcuugacucuaguccua.............  2     0    seq
.................................................................................gcuugacucuaguccuC.............  1     1    seq
.................................................................................gcuugacucuaguccCa.............  13    1    seq
.................................................................................gcuugacucuaguGcua.............  1     1    seq
.................................................................................gcuugacucuaguccGaac...........  2     1    seq
..................................................................................cuugacucuaguccGaac...........  1     1    seq
```

Provisional ID	:	MA_157730_8434
Score total	:	268.2
Score for star read(s)	:	3.9
Score for read counts	:	278
Score for mfe	:	-11.4
Score for randfold	:	-2.2
Score for cons. seed	:	
Total read count	:	557
Mature read count	:	554
Loop read count	:	0
Star read count	:	3

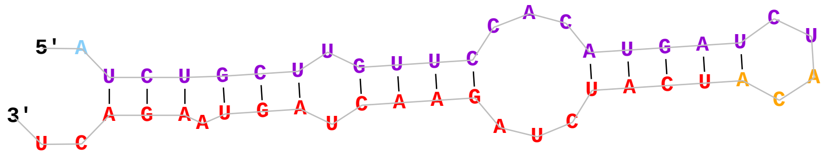

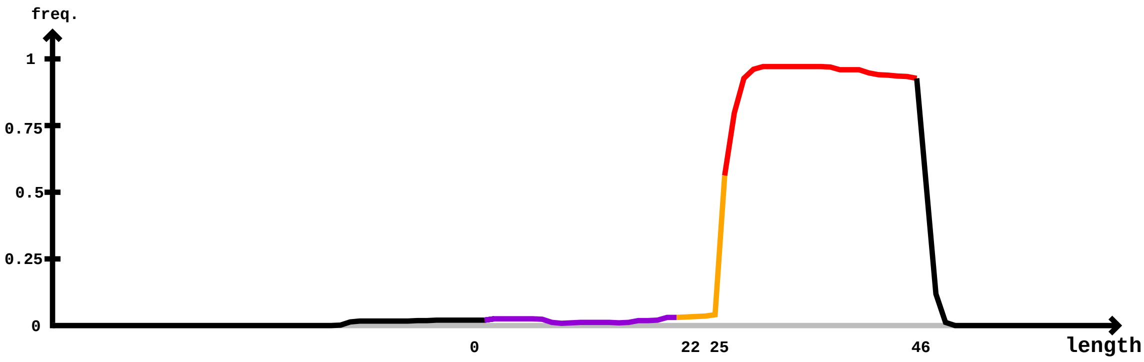

Star Mature

```
5'- uauccgggagaaauacaucuuuaacuguaacuggcaaaagugcaucugcuuguuccacaugaucuacaucaucuagaacuaguaagacuucaaaagauaagagugacaa  -3'   obs
    uauccgggagaaauacaucuuuaacuguaacuggcaaaagugcaucugcuuguuccacaugaucuacaucaucuagaacuaguaagacuucaaaagauaagagugacaa         exp
    ((((..((((...((((........))))....(((....)))...((((.((((...(((((....)))))...)))).))))...))))....))))..........   reads    mm    sample
    ............................aacGggcaaaagugcaucugc............................................................   1        1     seq
    .............................acGggcaaaagugcaucugcu...........................................................   7        1     seq
    ..............................cGggcaaaagugcaucugcuu..........................................................   2        1     seq
    ....................................aaagugcaucugcuuguucca....................................................   1        0     seq
    ......................................agugcaucugcuuguuccaca..................................................   1        0     seq
    ............................................ucugcuugCuccacaugaucu............................................   1        1     seq
    ............................................ucugcuuguuccacaugaucu............................................   2        0     seq
    ....................................................uuccacaugaucuacaucauG....................................   1        1     seq
    .....................................................uccacaugaucuacaucaucu...................................   1        0     seq
    ..........................................................augaucuacaucaucuagaac..............................   1        0     seq
    ...........................................................ugaucuacaucaucuaUaacu.............................   1        1     seq
    ...........................................................ugaucuacaucaucuagaacu.............................   4        0     seq
    .............................................................aucuacaucaucuagaacu.............................   1        0     seq
    ..............................................................ucuacaucaucuagaacuagC..........................   2        1     seq
    ..............................................................ucuacaucaucuagaacuagu..........................   4        0     seq
    ................................................................uacaucaucuagaacuaguaa........................   1        0     seq
    .................................................................acaucaucuagaacuagu..........................   1        0     seq
    .................................................................acaucaucuaUaacuagua.........................   1        1     seq
    .................................................................acaucaucuagaacuagua.........................   1        0     seq
    .................................................................acaucaucuagaacuaguaag.......................   2        0     seq
    ...................................................................aucaucuagaacuagua.........................   2        0     seq
    ...................................................................aucaucuagaacuaguaagac.....................   1        0     seq
    ....................................................................ucaucuagaacuaguaagac.....................   1        0     seq
    ....................................................................ucaucuagaacuaguaagacC....................   41       1     seq
    ....................................................................ucaucuagaacuaguaagaUu....................   1        1     seq
    ....................................................................ucaucuagaacuaguaagacG....................   2        1     seq
    ....................................................................ucaucuGgaacuaguaagacu....................   2        1     seq
    ....................................................................ucaucuagGacuaguaagacu....................   1        1     seq
    ....................................................................uUaucuagaacuaguaagacu....................   1        1     seq
    ....................................................................ucaucuagaacuaguaagacu....................   232      0     seq
    ....................................................................ucaCcuagaacuaguaagacu....................   2        1     seq
    ....................................................................ucaucuagaacuaguaagacA....................   5        1     seq
    ....................................................................ucaucuagaacuaAuaagacu....................   1        1     seq
```

Star Mature

```
uauccgggagaaauacaucuuuaacuguaacuggcaaaagugcaucugcuuguuccacaugaucuacaucaucuagaacuaguaagacuucaaaagauaagagugacaa
....................................................................ucaucCagaacuaguaagacu....................	1	1	seq
....................................................................Ccaucuagaacuaguaagacu....................	1	1	seq
....................................................................Ncaucuagaacuaguaagacu....................	2	1	seq
....................................................................ucaucuagaacuaguaGgacu....................	1	1	seq
....................................................................ucaucuagaacuaguaagGcu....................	1	1	seq
....................................................................ucaucuagaacuaguaagacuu...................	10	0	seq
....................................................................ucaucuagaacuaguaagacuC...................	1	1	seq
....................................................................ucaucuagaGcuaguaagacuu...................	1	1	seq
....................................................................ucaucuagaacuaguaagacuG...................	1	1	seq
.....................................................................caucuagaacuaguaagac.....................	2	0	seq
.....................................................................caucuagaacuaguaagacA....................	1	1	seq
.....................................................................caucuagaacuaguaagacC....................	1	1	seq
.....................................................................caucuagaacuaguaagacu....................	12	0	seq
.....................................................................caucuagaacuaguaagacG....................	1	1	seq
.....................................................................caucuagaacuaguaagacuG...................	15	1	seq
.....................................................................caucuagaacuaguaagacCu...................	1	1	seq
.....................................................................caucuagaacuaguaagacuC...................	11	1	seq
.....................................................................caucuagGacuaguaagacuu...................	1	1	seq
.....................................................................caucuagaacuaguaGgacuu...................	1	1	seq
.....................................................................caucuagaacuaguaagacuu...................	89	0	seq
.....................................................................caucuagaacuaguaagacuA...................	3	1	seq
......................................................................aucuagaacuaguaaga......................	1	0	seq
......................................................................aucuagaacuaguaagacC....................	3	1	seq
......................................................................aucuagaacuaguaagacu....................	12	0	seq
......................................................................aucuagaacuaguaagacuu...................	2	0	seq
......................................................................aucuagaacuaguaagacuuc..................	5	0	seq
......................................................................aucuagaacuaguaagacuuU..................	54	1	seq
.......................................................................ucuagaacuaguaagacA....................	1	1	seq
.......................................................................ucuagaacuaguaagacu....................	4	0	seq
.......................................................................ucuagaacuaguaagacC....................	3	1	seq
.......................................................................ucuagaacuaguaagacuG...................	2	1	seq
.......................................................................ucuagaacuaguaagacuuU..................	3	1	seq
.......................................................................ucuagaacuaguaagacuuUa.................	7	1	seq
........................................................................cuagaacuaguaagacG....................	1	1	seq
........................................................................cuagaacuaguaagacu....................	4	0	seq
........................................................................cuagaacuaguaagacuG...................	1	1	seq
.........................................................................uagaacuaguaagacuu...................	1	0	seq
..........................................................................agaacuaguaagacuuU..................	1	1	seq
```

Provisional ID	:	MA_159335_11489
Score total	:	2
Score for star read(s)	:	-1.3
Score for read counts	:	0
Score for mfe	:	1.7
Score for randfold	:	1.6
Score for cons. seed	:	
Total read count	:	178
Mature read count	:	178
Loop read count	:	0
Star read count	:	0

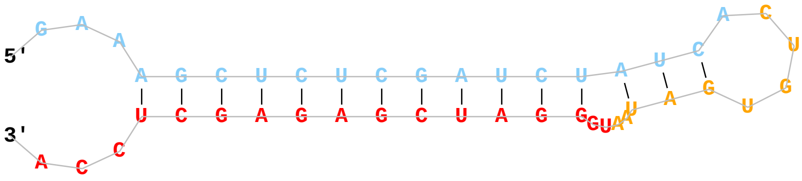

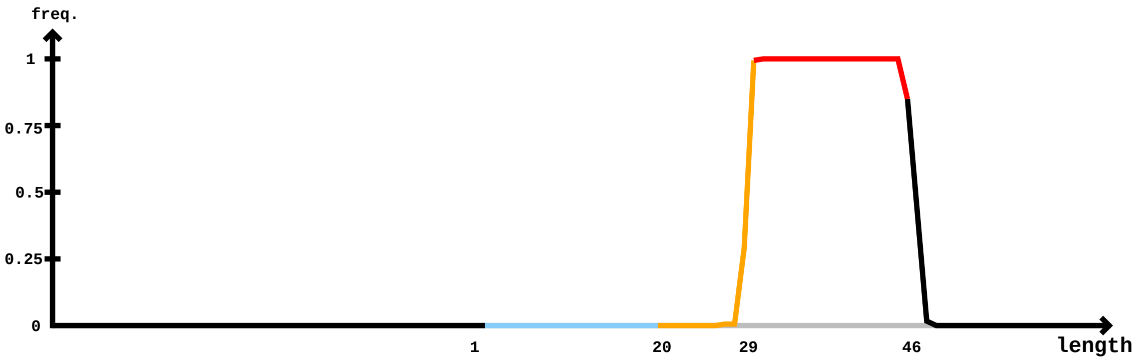

Star Mature

```
5'- guugucgaugcucucuccaagagggcccuauccgaggagacuggaaagcucucgaucuaucacugugauaaugggaucgagagcuccauuaccuccuuaaaccuuuuu   -3'    exp
    .........((((((.....)))))).......((((((..((((..((((((((((((((.....)))....)))))))))))))))....)))))).........     reads    mm    sample
    ....................................................................uCaugggaucgagagcucc.....................     1        1     seq
    ......................................................................augggaucgagagcucc.....................     23       0     seq
    ......................................................................auggUaucgagagcucc.....................     2        1     seq
    ......................................................................augggaucgagagcucU.....................     1        1     seq
    ......................................................................augggaucgagagcucca....................     16       0     seq
    ......................................................................augggaucgagagcuccC....................     1        1     seq
    ......................................................................augggaucgagagcAcca....................     1        1     seq
    ......................................................................auggUaucgagagcucca....................     2        1     seq
    ......................................................................augggaucgagagcuccG....................     2        1     seq
    ......................................................................auggAaucgagagcuccau...................     3        1     seq
    .......................................................................ugggaucgagagcuccG....................     6        1     seq
    .......................................................................ugggaucgagagcucca....................     97       0     seq
    .......................................................................uggCaucgagagcucca....................     5        1     seq
    .......................................................................Ggggaucgagagcucca....................     1        1     seq
    .......................................................................ugggaucUagagcucca....................     1        1     seq
    .......................................................................ugggaucgagagcuUca....................     1        1     seq
    .......................................................................uggAaucgagagcuccau...................     12       1     seq
    .......................................................................ugggaucgagagcuccaAu..................     1        1     seq
    .......................................................................uggAaucgagagcuccauu..................     2        1     seq
    ........................................................................ggAaucgagagcuccau...................     1        1     seq
```

Provisional ID	:	MA_16121_13692
Score total	:	0.9
Score for star read(s)	:	-1.3
Score for read counts	:	0
Score for mfe	:	0.6
Score for randfold	:	1.6
Score for cons. seed	:	
Total read count	:	466
Mature read count	:	433
Loop read count	:	0
Star read count	:	33

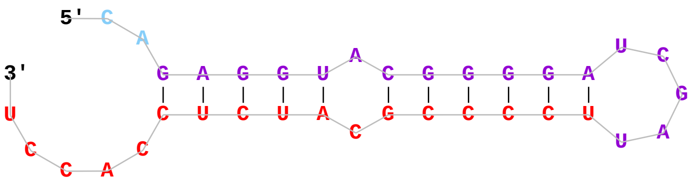

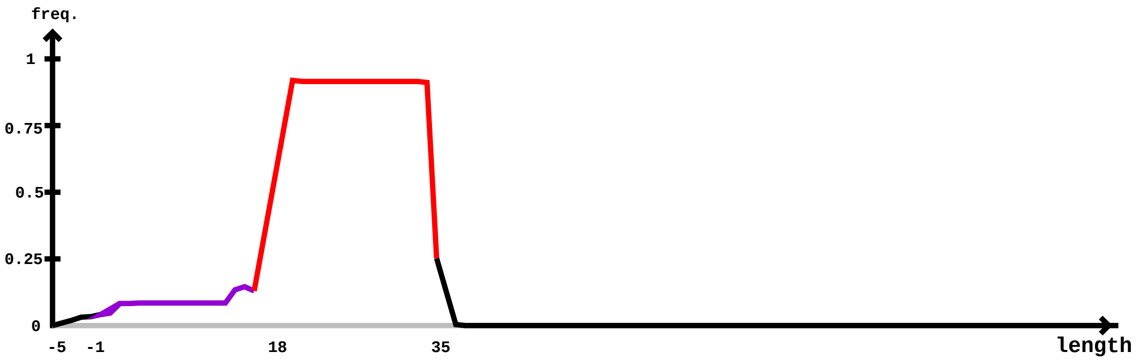

Star Mature

```
5'- ugccagagguacggggaucgauuccccgcaucuccaccuucuauguuauuaauaaauuuuaacagaaaaauugaaauguggguguuauagaguaaaauccuuggcuuu   -3'   obs
    ugccagagguacggggaucgauuccccgcaucuccaccuucuauguuauuaauaaauuuuaacagaaaaauugaaauguggguguuauagaguaaaauccuuggcuuu         exp
    .(((((((((.((((((.....)))))).)))))....(((((((.((((.....(((((((........)))))))...)))).))))))).........))))...    reads   mm   sample
    ugcUagagguacgggga...........................................................................................    1       1    seq
    ugcUagagguacggggauc.........................................................................................    5       1    seq
    ugccagagguacggggaucg........................................................................................    1       0    seq
    ugcUagagguacggggaucg........................................................................................    3       1    seq
    .gccagagguacggggauU.........................................................................................    1       1    seq
    .gAcagagguacggggauc.........................................................................................    1       1    seq
    .gcUagagguacggggauc.........................................................................................    3       1    seq
    .gccagagguacggggaucg........................................................................................    1       0    seq
    ..cUagagguacggggaucg........................................................................................    1       1    seq
    ...Uagagguacggggaucg........................................................................................    2       1    seq
    ...Nagagguacggggaucg........................................................................................    1       1    seq
    ...cagagguacggggUucg........................................................................................    1       1    seq
    ....agagguacggggaucgC.......................................................................................    1       1    seq
    ....agagguacggggaucgU.......................................................................................    1       1    seq
    ....agagguacggggaucgaG......................................................................................    1       1    seq
    .....gagguacggggaucgaG......................................................................................    2       1    seq
    .....gagguacggggaucgCu......................................................................................    2       1    seq
    .....gagguacggggaucgUu......................................................................................    3       1    seq
    .....gagguacggggaucgGu......................................................................................    2       1    seq
    .....gagguacggggaucgau......................................................................................    7       0    seq
    .....gagguacggggaucgCuu.....................................................................................    1       1    seq
    .....gagguacggggaucgauuG....................................................................................    1       1    seq
    .......gguacggggaucgauuG....................................................................................    1       1    seq
    .................ucgauuccccgcaucuccacc......................................................................    6       0    seq
    .................ucgauuccccgcaucuccacA......................................................................    1       1    seq
    .................ucgauuccccgcaucuccaccC.....................................................................    1       1    seq
    .................ucgauuccccgcaucuccaccA.....................................................................    18      1    seq
    ..................cgauuccccgcaucuccacc......................................................................    2       0    seq
    ..................cgCuuccccgcaucuccacc......................................................................    1       1    seq
    ..................cgauuccccgcaucuccaccC.....................................................................    1       1    seq
    ..................cgauuccccgcaucuccaccA.....................................................................    2       1    seq
    ...................gauuccccgcaucuccacc......................................................................    1       0    seq
    ...................gauuccccgcaucuccaccA.....................................................................    1       1    seq
```

```
     Star     Mature
ugccagagguacggggaucgauuccccgcaucuccaccuucuauguuauuaauaaauuuuaacagaaaaauugaaaugugggguguuauagaguaaaauccuuggcuuu
....................auuccccgcaucuccac........................................................................    1    0    seq
....................auGccccgcaucuccac........................................................................    1    1    seq
....................auucUccgcaucuccacc.......................................................................    1    1    seq
....................auuccccgcaucuccacc.......................................................................    8    0    seq
....................auucccUgcaucuccacc.......................................................................    3    1    seq
....................Guuccccgcaucuccacc.......................................................................    1    1    seq
....................auGccccgcaucuccacc.......................................................................    1    1    seq
....................Cuuccccgcaucuccacc.......................................................................    2    1    seq
....................auuccccgcaucuccaccA......................................................................    13   1    seq
....................auuccccgcaucuccaccu......................................................................    1    0    seq
....................Uuuccccgcaucuccaccu......................................................................    6    1    seq
.....................uGccccgcaucuccacc.......................................................................    13   1    seq
.....................uuccccgcaucuccGcc.......................................................................    2    1    seq
.....................uucUccgcaucuccacc.......................................................................    15   1    seq
.....................uuccUcgcaucuccacc.......................................................................    12   1    seq
.....................uuccccgcaucuccacc.......................................................................    249  0    seq
.....................Cuccccgcaucuccacc.......................................................................    1    1    seq
.....................uuccccgcaucuccacG.......................................................................    1    1    seq
.....................uuccccgcaucuccacA.......................................................................    9    1    seq
.....................uuUcccgcaucuccacc.......................................................................    2    1    seq
.....................uuccccUcaucuccacc.......................................................................    1    1    seq
.....................uuccccgcaucAccacc.......................................................................    1    1    seq
.....................uuccccAcaucuccacc.......................................................................    1    1    seq
.....................uuccccgUaucuccacc.......................................................................    1    1    seq
.....................uuccccgcaucuccaccG......................................................................    18   1    seq
.....................uuccccgcaucuccaccC......................................................................    5    1    seq
.....................uuccccgcaucuccaccu......................................................................    2    0    seq
......................ucUccgcaucuccaccu......................................................................    3    1    seq
......................uccccgcaucuccaccu......................................................................    8    0    seq
......................uccccgcaucuccaccC......................................................................    5    1    seq
......................Gccccgcaucuccaccu......................................................................    2    1    seq
......................uccUcgcaucuccaccu......................................................................    2    1    seq
......................uccccgcaucuccaccG......................................................................    36   1    seq
.......................ccccgcaucuccaccCu.....................................................................    1    1    seq
.......................ccccgcaucuccaccuG.....................................................................    1    1    seq
.......................ccccgcaucuccaccAuc....................................................................    2    1    seq
```

Provisional ID : MA_161279_16541
Score total : 0.3
Score for star read(s) : -1.3
Score for read counts : 0
Score for mfe : 0
Score for randfold : 1.6
Score for cons. seed :
Total read count : 228
Mature read count : 227
Loop read count : 1
Star read count : 0

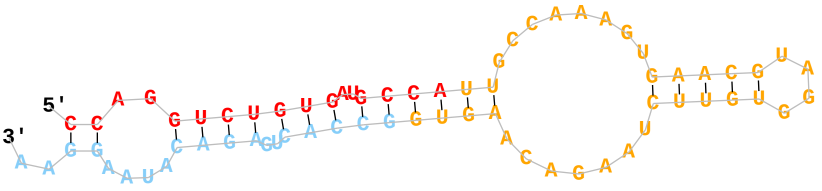

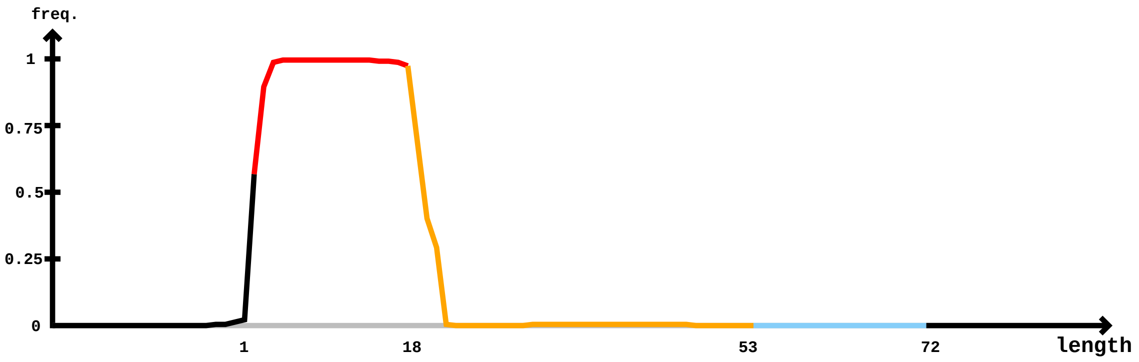

Mature Star

```
5'- aggcuuugagaagaauuuuccaggucugugaugccauugccaaagugaacguagguguucuaagacaaguggccacugagacauaaggaaaauacuuguccuuaauaa -3'   exp
    .......((.(((.(((((((..(((((((..((((((........(((((....))))).......)))))))))..))))....))))))).))).))........   reads   mm   sample
    ...............uCuuccaggucugugau............................................................................   1       1    seq
    ................uuAcaggucugugaugc...........................................................................   1       1    seq
    ................uuAcaggucugugaugcc..........................................................................   1       1    seq
    .................uAcaggucugugaugcc..........................................................................   2       1    seq
    ..................Acaggucugugaugcca.........................................................................   122     1    seq
    ...................ccaggucugugaugccC........................................................................   3       1    seq
    ....................caggucugugaugccaG.......................................................................   1       1    seq
    ....................caggucugugaugccGu.......................................................................   1       1    seq
    ....................caggucugugaugccUu.......................................................................   4       1    seq
    ....................caggucugugaugccauu......................................................................   6       0    seq
    ....................caggucugugaugccGuu......................................................................   4       1    seq
    ....................caggucugugaugccUuu......................................................................   12      1    seq
    ....................caggucugugaugccCuug.....................................................................   45      1    seq
    ....................caggucugugaugccauuA.....................................................................   2       1    seq
    .....................aggucugugaugccauu......................................................................   1       0    seq
    .....................aggucugugaugccaCu......................................................................   1       1    seq
    .....................aggucugugaugccauC......................................................................   1       1    seq
    .....................aggucugugaugccCuug.....................................................................   17      1    seq
    .....................aggucugugaugccCuugc....................................................................   1       1    seq
    ......................ggucugugaugccCuug.....................................................................   2       1    seq
    ................................................acgCagguguucuaaga...........................................   1       1    seq
```

Provisional ID	:	MA_16233_8995
Score total	:	0.4
Score for star read(s)	:	-1.3
Score for read counts	:	0
Score for mfe	:	0.1
Score for randfold	:	1.6
Score for cons. seed	:	
Total read count	:	59770
Mature read count	:	59770
Loop read count	:	0
Star read count	:	0

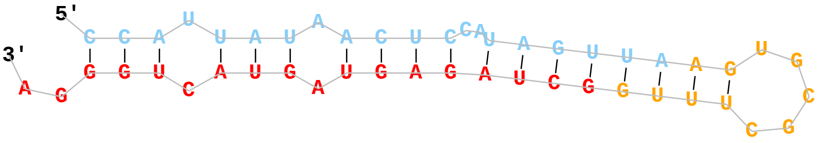

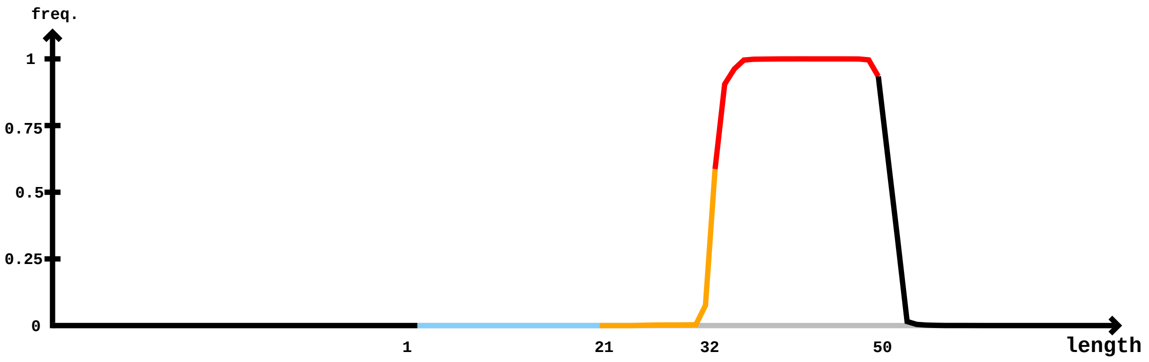

Star Mature

```
5'- cauauguauaugccuauagcguuaaugcauuggaucccauuauaacuccauaguuaagugcgcuuuggcuagaguaguacugggaugagugaccucucaggaaguccua   -3'    exp
    ((.((((((((((.....))))..)))))))).((((((.(((.((((..((((((((.....)))))))))))).))).)))))).((.(((.((....)).))))).    reads     mm      sample
    .........................................................gugcgcuuGggcuagaguaguacugggauga.....................    1         1       seq
    ...........................................................gcgcuuuggcuagagua.................................    1         0       seq
    ...........................................................gcgcuuuggcuagaguag................................    1         0       seq
    ...........................................................gcgcuuuggcuagaguaguacugg..........................    1         0       seq
    ...........................................................gcgcuuuggcuagaguaguacuggga........................    1         0       seq
    ...........................................................gcgcuuuggcuagaguaguacugggaug......................    1         0       seq
    ...........................................................gcgcuuGggcuagaguaguacugggauga.....................    16        1       seq
    ...........................................................gcgcuuGggcuagaguaguacugggaugag....................    7         1       seq
    ...........................................................gcgcuuGggcuagaguaguacugggaugagu...................    2         1       seq
    ...........................................................gcgcuuGggcuagaguaguacugggaugagug..................    4         1       seq
    ............................................................cgcuuCggcuagaguag................................    1         1       seq
    ............................................................cgcuuCggcuagaguagua..............................    1         1       seq
    ............................................................cgcuuCggcuagaguaguacuggga........................    2         1       seq
    ............................................................cgcuuGggcuagaguaguacugggauga.....................    37        1       seq
    ............................................................cgcuuGggcuagaguaguacugggaugag....................    11        1       seq
    ............................................................cgcuuGggcuagaguaguacugggaugagu...................    2         1       seq
    ............................................................cgcuuGggcuagaguaguacugggaugagug..................    3         1       seq
    .............................................................gcuuuggcuagaguagua..............................    1         0       seq
    .............................................................gcuuuggcuagaguaguacu............................    2         0       seq
    .............................................................gcuuCggcuagaguaguacugg..........................    1         1       seq
    .............................................................gcuuuggcuagaguaguacuggga........................    2         0       seq
    .............................................................gcuuuggcuagaguaguacugggU.......................    1         1       seq
    .............................................................gcuuuggcuagGguaguacugggaug......................    1         1       seq
    .............................................................gcuuuggcuagaguaguacugggaug......................    1         0       seq
    .............................................................gcuuGggcuagaguaguacugggauga.....................    20        1       seq
    .............................................................gcuuGggcuagaguaguacugggaugag....................    1         1       seq
    .............................................................gcuuGggcuagaguaguacugggaugagu...................    4         1       seq
    ..............................................................cuuuggcuagaguagua..............................    3         0       seq
    ..............................................................cuuCggcuagaguaguacug...........................    1         1       seq
    ..............................................................cuuuggcuagaguaguacug...........................    1         0       seq
    ..............................................................cuuuggcuagaguaguaUuggga........................    1         1       seq
    ..............................................................cuuuggcuagaguaguacugggG.......................    1         1       seq
    ..............................................................cuuuggcuagaguaguacugggaug......................    3         0       seq
    ..............................................................cuuCggcuagaguaguacugggaug......................    1         1       seq
```

```
                                    Star                          Mature
cauauguauaugccuauagcguuaaugcauuggaucccauuauaacuccauaguuaagugcgcuuuggcuagaguaguacugggaugagugaccucucaggaaguccua
...............................................................uuuggcuagaguaguacugg..........................   1      0    seq
................................................................uuggcuagaguaguacu............................   2      0    seq
................................................................Nuggcuagaguaguacug...........................   1      1    seq
................................................................uuggcuagaguaguacug...........................   2      0    seq
................................................................uuggcuagaguaguacugg..........................   1      0    seq
................................................................Nuggcuagaguaguacuggg.........................   1      1    seq
................................................................uuggcuagaguaguacuggg.........................   1      0    seq
................................................................uuggcuagaguaguacuggA.........................   1      1    seq
................................................................Nuggcuagaguaguacuggga........................   1      1    seq
................................................................uuggcuagaguaguacuggga........................   2      0    seq
................................................................uuggcuagaguaguacugggaC.......................   1      1    seq
................................................................Nuggcuagaguaguacugggaug.....................   2      1    seq
................................................................uuggcuagaguaguacugggaugaU...................   1      1    seq
.................................................................uggcuagaguaguacug...........................   3      0    seq
.................................................................Nggcuagaguaguacug...........................   1      1    seq
.................................................................Nggcuagaguaguacugg..........................   2      1    seq
.................................................................uggcuagaguaguacuggg.........................   2      0    seq
.................................................................uCgcuagaguaguacuggga........................   1      1    seq
.................................................................uUgcuagaguaguacuggga........................   1      1    seq
.................................................................Cggcuagaguaguacuggga........................   1      1    seq
.................................................................uggcuagaguaguacuggga........................   3      0    seq
.................................................................Nggcuagaguaguacuggga........................   1      1    seq
.................................................................uggcuagGguaguacugggau.......................   2      1    seq
.................................................................uggcuagaguaguacugggaC.......................   1      1    seq
.................................................................uggcuagaguaguacugggau.......................   3      0    seq
.................................................................Cggcuagaguaguacugggaug......................   2      1    seq
.................................................................uggcuagaguaguacugggaug......................   5      0    seq
..................................................................ggcuagaguaguacugg..........................   198    0    seq
..................................................................ggcuagaguaguacuggg.........................   307    0    seq
..................................................................ggcuagaguaguacuggga........................   1632   0    seq
..................................................................ggcuagaguCguacuggga........................   1      1    seq
..................................................................Ngcuagaguaguacuggga........................   2      1    seq
..................................................................ggcuagaguaguacugggau.......................   759    0    seq
..................................................................ggcuagaguaguaGugggau.......................   1      1    seq
..................................................................Ngcuagaguaguacugggaug......................   2      1    seq
..................................................................ggcuagaguaguacugggaug......................   1421   0    seq
..................................................................ggcuagGguaguacugggauga.....................   1      1    seq
..................................................................ggcuagaguaguacugggauga.....................   35     0    seq
..................................................................ggcuagagGaguacugggauga.....................   1      1    seq
..................................................................ggcuagaguaguacugggCugag....................   1      1    seq
..................................................................ggcuagaguaguacugggaugag....................   3      0    seq
..................................................................ggcuagaguGguacugggaugag....................   1      1    seq
...................................................................gcuagaguaguacuggg.........................   3399   0    seq
...................................................................Ncuagaguaguacuggg.........................   2      1    seq
...................................................................gcuCgaguaguacuggg.........................   1      1    seq
...................................................................gcuagaguaguaGuggg.........................   1      1    seq
...................................................................gcuCgaguaguacuggga........................   2      1    seq
...................................................................gcuagaguCguacuggga........................   3      1    seq
...................................................................gcuagaguaguaGuggga........................   3      1    seq
...................................................................gNuagaguaguacuggga........................   2      1    seq
...................................................................gcuagaguaguacuggga........................   15937  0    seq
...................................................................Ncuagaguaguacuggga........................   24     1    seq
...................................................................Ncuagaguaguacugggau.......................   7      1    seq
...................................................................gcuagaguaguacugggau.......................   3648   0    seq
...................................................................Ncuagaguaguacugggaug......................   8      1    seq
...................................................................gNuagaguaguacugggaug......................   2      1    seq
...................................................................gcuagaguaguacugggaug......................   7467   0    seq
...................................................................gcuagaguCguacugggaug......................   1      1    seq
...................................................................gcuagaguaguaGugggauga.....................   1      1    seq
...................................................................gcuagaguaguacuggAauga.....................   1      1    seq
...................................................................gcuagaguaguacugggGuga.....................   2      1    seq
...................................................................gcuagaguaguGcugggauga.....................   1      1    seq
...................................................................gcuagaguaguacugggaAga.....................   1      1    seq
...................................................................Ucuagaguaguacugggauga.....................   1      1    seq
...................................................................gcuagaguaguUcugggauga.....................   1      1    seq
...................................................................gcuagaguaguacugggaGga.....................   2      1    seq
...................................................................gcuagaguaguacugggauga.....................   110    0    seq
...................................................................gcuagGguaguacugggauga.....................   13     1    seq
...................................................................Acuagaguaguacugggauga.....................   1      1    seq
...................................................................gcuagagCaguacugggauga.....................   1      1    seq
```

```
                                    Star                         Mature
cauauguauaugccuauagcguuaaugcauuggaucccauuauaacuccauaguuaagugcgcuuuggcuagaguaguacugggaugagugaccucucaggaaguccua
...................................................................gcuagagCaguacugggaugag....................    1       1    seq
...................................................................gcuagaguaguacugggaugag....................    7       0    seq
...................................................................gcuagaguaguacugggaugaU....................    1       1    seq
...................................................................gcuagaguaguacugggaugagC...................    1       1    seq
...................................................................gcuagaguaguacugggaugCgu...................    2       1    seq
...................................................................gcuagaguaguacugggaugagug..................    1       0    seq
...................................................................gcuagaguaguacugggaugaguga.................    1       0    seq
....................................................................cuagaguCguacuggga........................    1       1    seq
....................................................................cuagaguaguacuggga........................    11182   0    seq
....................................................................Nuagaguaguacuggga........................    17      1    seq
....................................................................Nuagaguaguacugggau.......................    1       1    seq
....................................................................cuagaguaguacugggau.......................    1705    0    seq
....................................................................cuagaguaguacugggaug......................    5911    0    seq
....................................................................cuagaguCguacugggaug......................    2       1    seq
....................................................................Nuagaguaguacugggaug......................    14      1    seq
....................................................................cuCgaguaguacugggaug......................    1       1    seq
....................................................................cuagGguaguacugggauga.....................    23      1    seq
....................................................................cuagaguaguacugggGuga.....................    1       1    seq
....................................................................cuGgaguaguacugggauga.....................    1       1    seq
....................................................................cuagaguagCacugggauga.....................    1       1    seq
....................................................................cuagUguaguacugggauga.....................    1       1    seq
....................................................................cuagaguaguacugggauga.....................    173     0    seq
....................................................................cuagaguaguacugggauUa.....................    1       1    seq
....................................................................cuagGguaguacugggaugag....................    3       1    seq
....................................................................cuagaguaguacugggaugaU....................    1       1    seq
....................................................................cuagaguaguacugggaugNg....................    1       1    seq
....................................................................cuagaguaguacugggaugag....................    28      0    seq
....................................................................cuagaguaguacugggaugagu...................    7       0    seq
....................................................................Nuagaguaguacugggaugagu...................    1       1    seq
....................................................................cuagaguaguacugggGugagu...................    1       1    seq
....................................................................cuagaguaguacugggaugCgu...................    1       1    seq
....................................................................cuagaguaguacugggaugagG...................    1       1    seq
....................................................................cuagaguaguacuggAaugagu...................    1       1    seq
....................................................................cuagaguaguacugggaugagug..................    1       0    seq
.....................................................................uagaguaguacugggau.......................    1470    0    seq
.....................................................................uCgaguaguacugggau.......................    1       1    seq
.....................................................................Nagaguaguacugggau.......................    2       1    seq
.....................................................................uagaguCguacugggaug......................    1       1    seq
.....................................................................uCgaguaguacugggaug......................    1       1    seq
.....................................................................Nagaguaguacugggaug......................    1       1    seq
.....................................................................uagaguaguacugggaug......................    1889    0    seq
.....................................................................uagaUuaguacugggauga.....................    1       1    seq
.....................................................................uagaguaguacugggauga.....................    30      0    seq
.....................................................................uagGguaguacugggauga.....................    4       1    seq
.....................................................................uagaguaguacugggauUa.....................    1       1    seq
.....................................................................uagaguaguacugggaugag....................    5       0    seq
.....................................................................uagaAuaguacugggaugag....................    1       1    seq
.....................................................................uagaguaguacugggaugagu...................    2       0    seq
.....................................................................uagaguaguacuggAaugagu...................    1       1    seq
.....................................................................uagaguaguacugggaugaCu...................    1       1    seq
.....................................................................uagaguaguacugggaugagugaccucCc...........    1       1    seq
......................................................................agaguaguacugggaug......................    1946    0    seq
......................................................................Ngaguaguacugggaug......................    3       1    seq
......................................................................Cgaguaguacugggaug......................    3       1    seq
......................................................................agGguaguacugggauga.....................    3       1    seq
......................................................................agaguaguacugggauga.....................    41      0    seq
......................................................................agaguaguacugggaugag....................    8       0    seq
......................................................................agGguaguacugggaugag....................    1       1    seq
......................................................................agaguaguacugggaugagug..................    1       0    seq
......................................................................agaguaguacugggaugaguC..................    1       1    seq
.......................................................................gaguaguacCgggauga.....................    1       1    seq
.......................................................................gaguaguacugggauUa.....................    1       1    seq
.......................................................................gaguaguacugggaAga.....................    1       1    seq
.......................................................................Uaguaguacugggauga.....................    1       1    seq
.......................................................................gaguaguacugggaGga.....................    3       1    seq
.......................................................................gGguaguacugggauga.....................    5       1    seq
.......................................................................gaguGguacugggauga.....................    1       1    seq
.......................................................................gaguaguacugggauga.....................    114     0    seq
.......................................................................Naguaguacugggauga.....................    1       1    seq
.......................................................................gUguaguacugggauga.....................    1       1    seq
```

Star Mature

```
cauauguauaugccuauagcguuaaugcauuggaucccauuauaacuccauaguuaagugcgcuuuggcuagaguaguacugggaugagugaccucucaggaaguccua
.......................................................................gaguaguacugggaugag....................    39    0    seq
.......................................................................gGguaguacugggaugag....................    2     1    seq
.......................................................................gaguaguaGugggaugag....................    1     1    seq
.......................................................................gaguaguGcugggaugag....................    1     1    seq
.......................................................................gaguaguacCgggaugag....................    1     1    seq
.......................................................................gaguaguacugggaugagu...................    4     0    seq
.......................................................................gaguaguacuggAaugagu...................    3     1    seq
.......................................................................gaguaguacugggaugagug..................    6     0    seq
........................................................................aguaguacugggaugag....................    22    0    seq
........................................................................Gguaguacugggaugag....................    1     1    seq
........................................................................aguaguacugggaugagu...................    4     0    seq
........................................................................aguaguacugggaugagC...................    2     1    seq
........................................................................aguaguacugggaugagug..................    2     0    seq
........................................................................aguaguacugggaugagGg..................    1     1    seq
.........................................................................guaguacugggaugagu...................    15    0    seq
.........................................................................guaguacuggAaugagu...................    1     1    seq
.........................................................................guaguacugggaugagC...................    2     1    seq
.........................................................................guaguacugggaugagug..................    4     0    seq
.........................................................................guaguacugggaugagugacc...............    1     0    seq
..........................................................................uaguacugggaugaUug..................    1     1    seq
..........................................................................uaAuacugggaugagug..................    1     1    seq
..........................................................................uaguacugggaugagug..................    13    0    seq
..........................................................................uaguacugggaugagugacc...............    1     0    seq
..........................................................................uaguacugggaugagugGcc...............    1     1    seq
...........................................................................aguacugggaugagugacc...............    1     0    seq
...........................................................................aguacugggaugagugaccC..............    1     1    seq
............................................................................guacugggaugagugacc...............    1     0    seq
............................................................................guacugggaugagugGccu..............    1     1    seq
.............................................................................uacugggaugagugaccu..............    1     0    seq
```

Provisional ID	:	MA_163640_8871
Score total	:	1.7
Score for star read(s)	:	-1.3
Score for read counts	:	0
Score for mfe	:	1.4
Score for randfold	:	1.6
Score for cons. seed	:	
Total read count	:	7009
Mature read count	:	7009
Loop read count	:	0
Star read count	:	0

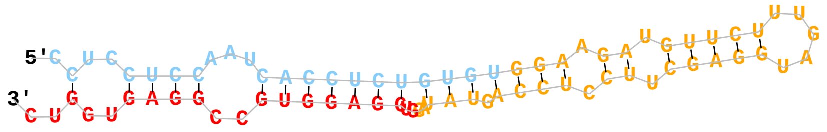

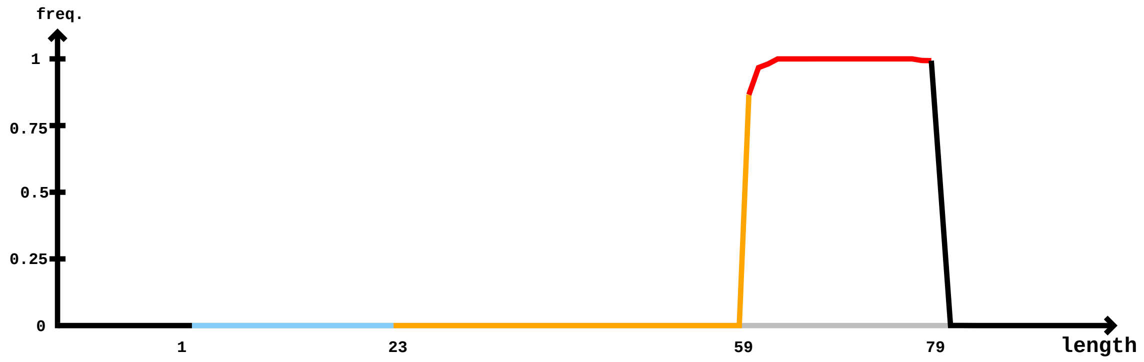


```
                  Star                                                    Mature
5'- aaucgggagauaccuccuccaaucaccucuguguggaagauguucuuugauggagcuuccuccaguauagguggaggugccggaguggucuucauaucacccugucaa   -3'   exp
    ....(((.((((((..((((...((((((((((((((.((.(((((.....))))).)).)))).)))....)))))))..)))).)))......))).)))......   reads   mm   sample
    ......................................................................guggaggugccggagugA....................   1       1    seq
    ......................................................................guggaAgugccggagugg....................   24      1    seq
    ......................................................................guggaggugccAgagugg....................   1       1    seq
    ......................................................................guggaggugccggagugg....................   10      0    seq
    ......................................................................guggaggugccggaguggu...................   2       0    seq
    ......................................................................guggaAgugccggaguggu...................   2       1    seq
    ......................................................................guggaUgugccggagugguc..................   1       1    seq
    ......................................................................guggaggugccggagugguU..................   7       1    seq
    ......................................................................guggaCgugccggagugguc..................   6015    1    seq
    ......................................................................guggaggugccggagugguc..................   2       0    seq
    ......................................................................guggaCgugccggaguggucu.................   2       1    seq
    ......................................................................guggaCgugccggaguggucuuc...............   1       1    seq
    .......................................................................uggaAgugccggagugg....................   1       1    seq
    .......................................................................uggaggugccggagugg....................   5       0    seq
    .......................................................................uggaAgugccggaguggu...................   1       1    seq
    .......................................................................uggaggugccggaguggu...................   1       0    seq
    .......................................................................uggaUgugccggagugguc..................   1       1    seq
    .......................................................................uggaCgugccggagugguc..................   702     1    seq
    ........................................................................ggaCgugccggagugguc..................   97      1    seq
    .........................................................................gaggugccggagugguU..................   2       1    seq
    .........................................................................gaCgugccggagugguc..................   130     1    seq
    ...............................................................................ugccggagugguUuuca............   1       1    seq
```

Provisional ID	:	MA_163819_17323
Score total	:	193.4
Score for star read(s)	:	3.9
Score for read counts	:	230.6
Score for mfe	:	-38.8
Score for randfold	:	-2.2
Score for cons. seed	:	
Total read count	:	464
Mature read count	:	461
Loop read count	:	0
Star read count	:	3

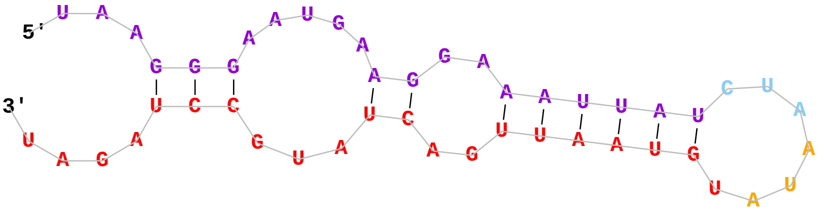

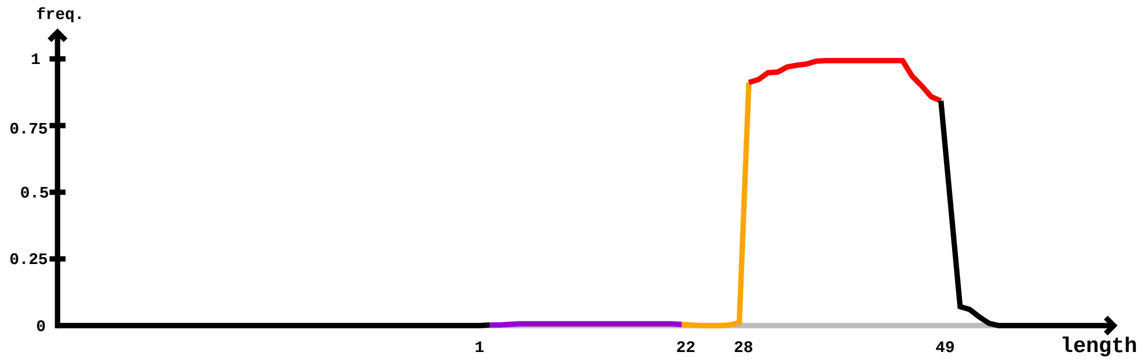


```
                                             Star                      Mature
5'- uugugcaccaggaaaaaaaaaaaucauuagguggaaucauuuguauaagggaaugaaggaaauuaucuaauauguaauugacuaugccuagaucucaaagaaaugacaau  -3'  obs
    uugugcaccaggaaaaaaaaaaaucauuagguggaaucauuuguauaagggaaugaaggaaauuaucuaauauguaauugacuaugccuagaucucaaagaaaugacaau       exp
    ((((...(((..((...........))..)))..((.((((.((..(((.....((..((((((.......))))))..))...)))..))..))))))....)))).   reads   mm   sample
    .............................................uaagggaaugaaggaaauuau............................................   1   0   seq
    ...............................................agggaaugaaggaaauua.............................................   1   0   seq
    ................................................gggaaugaaggaaauuau............................................   1   0   seq
    ......................................................................uCuguaauugacuaugccuagaucucaa............   1   1   seq
    .......................................................................Uuguaauugacuaugccuagau.................   3   1   seq
    .......................................................................Nuguaauugacuaugccuagau.................   1   1   seq
    ........................................................................uguaauugacuaugccC.....................   4   1   seq
    ........................................................................uguaauugacuaugccu.....................   21   0   seq
    ........................................................................uguaauugacuaugccG.....................   1   1   seq
    ........................................................................uguaauugacuaugccA.....................   1   1   seq
    ........................................................................uguaauugacuaugccua....................   16   0   seq
    ........................................................................uguaauugacuaugccuaA...................   1   1   seq
    ........................................................................uguaauugacuaugccuag...................   17   0   seq
    ........................................................................uguaauugacuaugccCag...................   1   1   seq
    ........................................................................uguaauugacuaugccuagC..................   1   1   seq
    ........................................................................uguaauugacuaugccuagG..................   1   1   seq
    ........................................................................uguaauugacuaugccuaga..................   5   0   seq
    ........................................................................uguaauugacuaugccuagau.................   213   0   seq
    ........................................................................uguaauugacuaugccuagaA.................   1   1   seq
    ........................................................................Cguaauugacuaugccuagau.................   1   1   seq
    ........................................................................uguaauugacuaugccuagaC.................   26   1   seq
    ........................................................................uguaauugacAaugccuagau.................   1   1   seq
    ........................................................................uguaauugacuaugccuagaG.................   2   1   seq
    ........................................................................uguaauugacuaugccCagau.................   2   1   seq
    ........................................................................Gguaauugacuaugccuagau.................   1   1   seq
    ........................................................................uguGauugacuaugccuagauc................   1   1   seq
    ........................................................................uguaauugacuaugccuagaCc................   4   1   seq
    ........................................................................uguaauugacuaugccuagauU................   6   1   seq
    ........................................................................uguaauugacuaugccuagGuc................   1   1   seq
    ........................................................................uguaauugacuaugccuagauc................   73   0   seq
    ........................................................................Cguaauugacuaugccuagauc................   2   1   seq
    ........................................................................uguaauugacuaugccuagaucu...............   4   0   seq
    ........................................................................uguaauugacuaugccuagaucuc..............   5   0   seq
```

Star **Mature**

uugugcaccaggaaaaaaaaaaucauuaguggaaucauuuguauaagggaaugaaggaaauuaucuaauauguaauugacuaugccuagaucucaaagaaaugacaau			
....uguaauugacuaugccuagaucuca.............	4	0	seq
....uguaauugacuaugccuagaucucaa............	2	0	seq
....guaauugGcuaugccua....................	1	1	seq
....guaauugacuaugccuagau.................	2	0	seq
....guaauugacuaugccuagaucuc..............	2	0	seq
....uaauugacuaugccuagaC.................	2	1	seq
....uaauugacuaugccuagau.................	3	0	seq
....uaaGugacuaugccuagauc................	1	1	seq
....uaauugacuaugccuagauc................	4	0	seq
....uaauugacuaugccuagaucuc..............	2	0	seq
....aauugacuaugccuagau.................	1	0	seq
....auugacuaugccuagau.................	3	0	seq
....auugacuaugccuagaC.................	1	1	seq
....auuCacuaugccuagauc................	1	1	seq
....auugacuaugccuagauc................	1	0	seq
....auugacuaugccuagaucu...............	1	0	seq
....auugacuaugccuagaucuc..............	1	0	seq
....auugacuaugccuagaucuca.............	1	0	seq
....uugacuaugccuagauc................	1	0	seq
....uugacuaugccuagaucucU.............	1	1	seq
....uugacuaugccuagaucucaU............	1	1	seq
....ugacuaugccuagaucuca.............	2	0	seq
....gacuaugccuagaucuc..............	3	0	seq
....gacuaugccuagaucuca.............	2	0	seq
....acuaugccuagaucuca.............	1	0	seq

Provisional ID : MA_164191_14549
Score total : 0
Score for star read(s) : -1.3
Score for read counts : 0
Score for mfe : -0.2
Score for randfold : 1.6
Score for cons. seed :
Total read count : 3749
Mature read count : 3749
Loop read count : 0
Star read count : 0

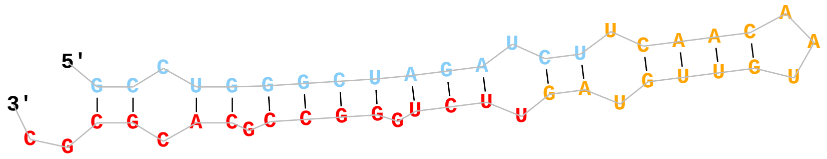

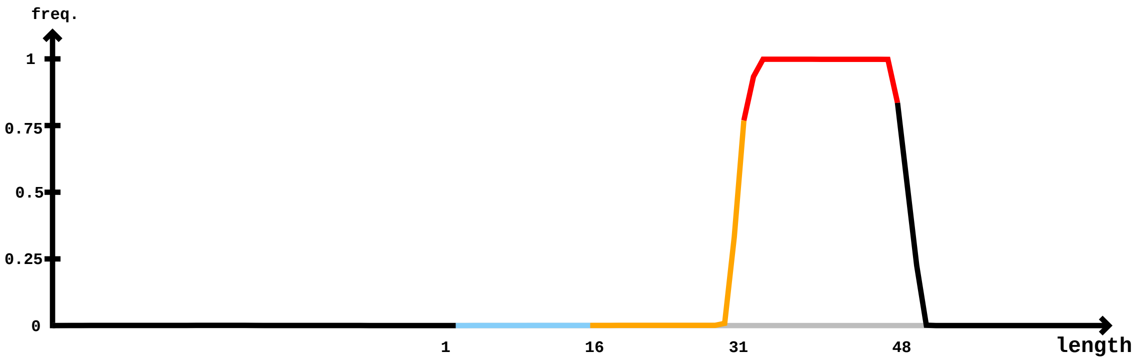

Star Mature

```
5'- acaaaacugaaggacuucaauggcugacaucguugaaggugccugggcuagaucuucaacaauguuguaguucugggccgcacgcgcaugaggauunnnnnnnnnnnn  -3'   exp
    ..............(((((((((......)))))))))((((.(((((((((.((.((((...)))).)).))).)))).)).)))).....................   reads   mm   sample
    Ucaaaacugaaggacuucaa........................................................................................   1       1    seq
    ..aaaacugaaggacuuca.........................................................................................   1       0    seq
    .............acuucaauggcugaGauc.............................................................................   1       1    seq
    ..........................................cugggcuagaucuucCacaauguu...........................................   1       1    seq
    ................................................uagaucuuCacaauguuguag.......................................   1       1    seq
    .........................................................aacaauguuguaguuUgggc...............................   1       1    seq
    ..................................................................guUguucugggccgcacgcgca....................   1       1    seq
    ....................................................................Nguucugggccgcacgc.......................   1       1    seq
    ....................................................................aguucugggccgcacgcg......................   5       0    seq
    ....................................................................Cguucugggccgcacgcg......................   3       1    seq
    ....................................................................Nguucugggccgcacgcg......................   3       1    seq
    ....................................................................Cguucugggccgcacgcgc.....................   5       1    seq
    ....................................................................aguucugggccgcacgcgc.....................   1       0    seq
    ....................................................................aguucugggccgcacgcgca....................   1       0    seq
    ....................................................................aguucugggccgcacgcgcG....................   7       1    seq
    ....................................................................Nguucugggccgcacgcgca....................   1       1    seq
    ....................................................................Uguucugggccgcacgcgcau...................   1       1    seq
    .....................................................................guucugggccgcacgcg......................   512     0    seq
    .....................................................................guucugggccgcacgcU......................   10      1    seq
    .....................................................................gCucugggccgcacgcg......................   1       1    seq
    .....................................................................guucugggccgcacgcA......................   59      1    seq
    .....................................................................guCcugggccgcacgcg......................   3       1    seq
    .....................................................................guucugggccgcGcgcg......................   2       1    seq
    .....................................................................guucCgggccgcacgcg......................   1       1    seq
    .....................................................................guucugggccgcacgGg......................   3       1    seq
    .....................................................................guucugggccgcaGgcg......................   1       1    seq
    .....................................................................Uuucugggccgcacgcg......................   1       1    seq
    .....................................................................guucAgggccgcacgcg......................   1       1    seq
    .....................................................................guucugggAcgcacgcg......................   1       1    seq
    .....................................................................guucugggccgcacgcC......................   5       1    seq
    .....................................................................Nuucugggccgcacgcg......................   1       1    seq
    .....................................................................guucCgggccgcacgcgc.....................   1       1    seq
    .....................................................................guucugggccgcacgcgU.....................   62      1    seq
    .....................................................................gCucugggccgcacgcgc.....................   1       1    seq
```

Star Mature

acaaaacugaaggacuucaauggcugacaucguugaaggugccugggcuagaucuucaacaauguuguaguucugggccgcacgcgcaugaggauunnnnnnnnnnnn			
....guucugggccgcacgcgG.....................	3	1	seq
....Cuucugggccgcacgcgc.....................	1	1	seq
....guucugggccgcacgcCc.....................	1	1	seq
....guucugggccgcacgcgc.....................	314	0	seq
....guucugggccgcacgcAc.....................	1	1	seq
....guucugggccgcGcgcgc.....................	2	1	seq
....guCcugggccgcacgcgc.....................	1	1	seq
....guucugggccUcacgcgca....................	1	1	seq
....guucugggccgcacgcgca....................	48	0	seq
....guCcugggccgcacgcgca....................	1	1	seq
....guucugggccgcacgcgcU....................	10	1	seq
....guucugAgccgcacgcgca....................	1	1	seq
....Uuucugggccgcacgcgca....................	1	1	seq
....guucugggccgcacgcgcC....................	8	1	seq
....guucugggccgcacgcgcau...................	2	0	seq
....guucugggccgcacgcgcGu...................	156	1	seq
....guucugggccgcacgcgcCu...................	1	1	seq
....uucugggccgcGcgcgc.....................	3	1	seq
....uucugggcAgcacgcgc.....................	1	1	seq
....Nucugggccgcacgcgc.....................	4	1	seq
....uucugggccgcacgcAc.....................	1	1	seq
....uucCgggccgcacgcgc.....................	2	1	seq
....uucugggccgcacgcgc.....................	586	0	seq
....Cucugggccgcacgcgc.....................	3	1	seq
....uucAgggccgcacgcgc.....................	1	1	seq
....Aucugggccgcacgcgc.....................	1	1	seq
....uCcugggccgcacgcgc.....................	1	1	seq
....uucugggccgcacgcgU.....................	108	1	seq
....uucugggccgcacgGgc.....................	1	1	seq
....uucugggccgGacgcgc.....................	1	1	seq
....uucugggccgcacgcgG.....................	7	1	seq
....uucugggccgcacgUgca....................	3	1	seq
....uucuAggccgcacgcgca....................	1	1	seq
....uucugggccgcUcgcgca....................	1	1	seq
....uucugggccgcacgcgcU....................	84	1	seq
....uucugggccgcacgcgcC....................	54	1	seq
....Cucugggccgcacgcgca....................	4	1	seq
....uucugggcAgcacgcgca....................	1	1	seq
....uucCgggccgcacgcgca....................	2	1	seq
....uucugggccgcGcgcgca....................	2	1	seq
....uucugggccgcacgcgUa....................	2	1	seq
....uucugggccgcacAcgca....................	1	1	seq
....uucugggccgcaUgcgca....................	1	1	seq
....uCcugggccgcacgcgca....................	4	1	seq
....uucugggccgcaGgcgca....................	1	1	seq
....uucugggccgcacgcAca....................	3	1	seq
....uucugggccgGacgcgca....................	1	1	seq
....uucugggccgcacgcgca....................	501	0	seq
....Gucugggccgcacgcgca....................	2	1	seq
....uucugggccgcacgGgca....................	1	1	seq
....uuUugggccgcacgcgca....................	2	1	seq
....uucugggccgcacgcgcCu...................	2	1	seq
....uucugggccgcacgcgcGu...................	246	1	seq
....uucugggccgcacgcgcau...................	2	0	seq
....ucugggccgUacgcgca....................	2	1	seq
....ucugggccgcacgGgca....................	1	1	seq
....ucugggccgcacgcgca....................	322	0	seq
....Ccugggccgcacgcgca....................	1	1	seq
....ucugggccgcacAcgca....................	2	1	seq
....ucugggccgcacgcgcC....................	35	1	seq
....ucugggccgcacgcgUa....................	1	1	seq
....ucugggccAcacgcgca....................	1	1	seq
....ucugggccgcacgcgcU....................	53	1	seq
....ucugggccgcacgcAca....................	2	1	seq
....ucuUggccgcacgcgca....................	1	1	seq
....ucugggccgcaUgcgca....................	1	1	seq
....ucugggccgcacgcgGa....................	1	1	seq
....ucugggccgcGcgcgca....................	3	1	seq
....ucugggccgcacgcgAa....................	1	1	seq
....uUugggccgcacgcgca....................	1	1	seq

Star Mature

```
acaaaacugaaggacuucaauggcugacaucguugaagguGCCUGGGCUAGAUCUucaacaauguuguaguucugggccgcacgcgcaugaggauunnnnnnnnnnnn
...................................................................ucugggccgcacgcgcau..................   1     0     seq
...................................................................ucugggccgcacgcgcGu..................   186   1     seq
...................................................................ucugggccgcacgcgcGug.................   1     1     seq
....................................................................cugggccgcacgcgcCu..................   4     1     seq
....................................................................cugggccgcacgcgcGu..................   239   1     seq
....................................................................cugggccgcacgcgcGug.................   2     1     seq
....................................................................cugggccgcacgcgcUug.................   1     1     seq
....................................................................cugggccgcacgcgcaug.................   1     0     seq
```

Provisional ID	:	MA_164785_10119
Score total	:	0.5
Score for star read(s)	:	-1.3
Score for read counts	:	0
Score for mfe	:	0.2
Score for randfold	:	1.6
Score for cons. seed	:	
Total read count	:	4774
Mature read count	:	4774
Loop read count	:	0
Star read count	:	0

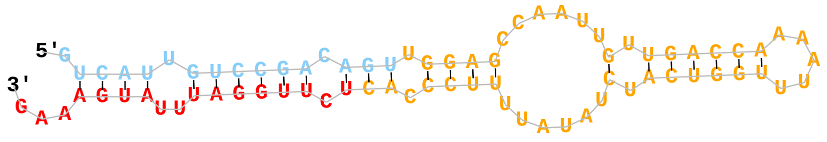

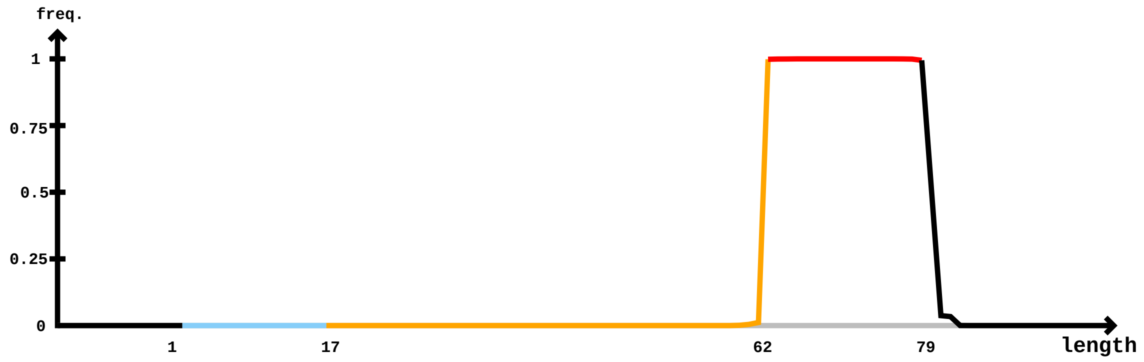


```
            Star                                                        Mature
5'- acccacaugggucauuguccgacaguuggagccaauuguugaccaaaauuuggucaucuauauuuucccacucuuggauuuaugaaagccuuguuaucaaaagaaga  -3'   exp
    .(((....))).((((.((((((.(((.((((......(.((((((.....)))))).)......)))).))).))))))..))))....(((.((.....)).))).    reads   mm   sample
    .....................................................................Aacucuuggauuuauga......................    1       1    seq
    .....................................................................Aacucuuggauuuaugaaag...................    4       1    seq
    ......................................................................acucuuggauuuaugaa.....................    2       0    seq
    ......................................................................aAucuuggauuuaugaa.....................    1       1    seq
    ......................................................................acucuuggauuuaugaaa....................    7       0    seq
    ......................................................................acucuuggauuuaugaaag...................    4       0    seq
    ......................................................................aAucuuggauuuaugaaag...................    1       1    seq
    ......................................................................acucuuggauuuaugaaagA..................    1       1    seq
    .......................................................................cucuuggauuuaugaaa....................    10      0    seq
    .......................................................................Nucuuggauuuaugaaa....................    5       1    seq
    .......................................................................cucuuggaCuuaugaaag...................    1       1    seq
    .......................................................................cucuuggauuuaugaaag...................    5       0    seq
    .......................................................................Nucuuggauuuaugaaag...................    4       1    seq
    .......................................................................Uucuuggauuuaugaaagc..................    1       1    seq
    .......................................................................cucuuggauuuaugaaagA..................    5       1    seq
    .......................................................................Uucuuggauuuaugaaagcc.................    1       1    seq
    .......................................................................cucuuggauuuaugaaagAc.................    2       1    seq
    ........................................................................ucuuggauCuaugaaag...................    3       1    seq
    ........................................................................ucuuggauuuaGgaaag...................    1       1    seq
    ........................................................................ucuGggauuuaugaaag...................    1       1    seq
    ........................................................................ucuuggauuuUugaaag...................    2       1    seq
    ........................................................................ucCuggauuuaugaaag...................    13      1    seq
    ........................................................................ucuuggauuuaugaUag...................    5       1    seq
    ........................................................................ucuuggauuuaCgaaag...................    10      1    seq
    ........................................................................ucuuggauuuaugaaag...................    4442    0    seq
    ........................................................................ucuuggauuuaugaaUg...................    4       1    seq
    ........................................................................ucuuggauuAaugaaag...................    2       1    seq
    ........................................................................ucuCggauuuaugaaag...................    5       1    seq
    ........................................................................ucuuggauuCaugaaag...................    3       1    seq
    ........................................................................Acuuggauuuaugaaag...................    3       1    seq
    ........................................................................ucuuggauuuaAgaaag...................    1       1    seq
    ........................................................................ucuuggauuuaugUaag...................    4       1    seq
    ........................................................................Ncuuggauuuaugaaag...................    10      1    seq
    ........................................................................ucuAggauuuaugaaag...................    2       1    seq
```

Star Mature

```
acccacaugggGUCAUUGUCCGACAGUuggagccaauuguugaccaaaauuuggucaucuauauuuucccacUCUUGGAUUUAUGAAAGccuuguuaucaaaagaaga
........................................................................ucuuggauuuaugaGag...................     3     1     seq
........................................................................ucuuggaAuuaugaaag...................     1     1     seq
........................................................................ucuuggauGuaugaaag...................     2     1     seq
........................................................................uUuuggauuuaugaaag...................     4     1     seq
........................................................................ucuuggGuuuaugaaag...................     16    1     seq
........................................................................ucuuggUuuuaugaaag...................     1     1     seq
........................................................................ucuuAgauuuaugaaag...................     2     1     seq
........................................................................ucuuggauuuaugaaaCc..................     1     1     seq
........................................................................ucuuggauuuaugaaagU..................     5     1     seq
........................................................................ucuuggauuuaugaaagUc.................     1     1     seq
........................................................................ucuuggauuuaugaaagGc.................     7     1     seq
........................................................................ucuuggauuuaugaaagcA.................     2     1     seq
........................................................................ucuuggauuuaugaaagcc.................     1     0     seq
........................................................................ucuuggauuuaugaaagcG.................     1     1     seq
........................................................................ucuuggauuuaugaaagGcu................     1     1     seq
........................................................................ucuuggauuuaugaaagccA................     1     1     seq
........................................................................ucuuggauuuaugaaagccu................     1     0     seq
........................................................................ucuuggauuuaugaaagccG................     4     1     seq
........................................................................ucuuggauuuaugaaagAcu................     151   1     seq
........................................................................ucuuggauuuaugaaagAcuu...............     1     1     seq
.........................................................................cuuggauuuaugaaagU..................     3     1     seq
.........................................................................cuuggauuuaugaaagcA.................     1     1     seq
..........................................................................uuggauuCaugaaagcc.................     1     1     seq
...........................................................................uggauuuaugaaagccG................     2     1     seq
```

Provisional ID	:	MA_166298_22659
Score total	:	0.2
Score for star read(s)	:	-1.3
Score for read counts	:	0
Score for mfe	:	0
Score for randfold	:	1.6
Score for cons. seed	:	
Total read count	:	2276
Mature read count	:	2276
Loop read count	:	0
Star read count	:	0

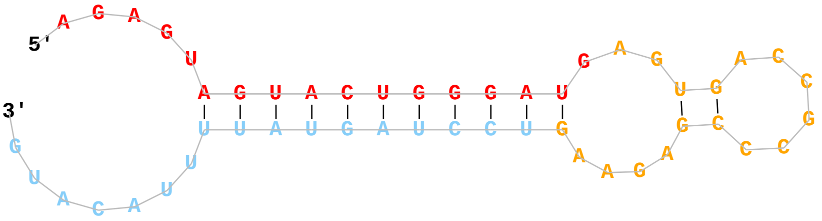

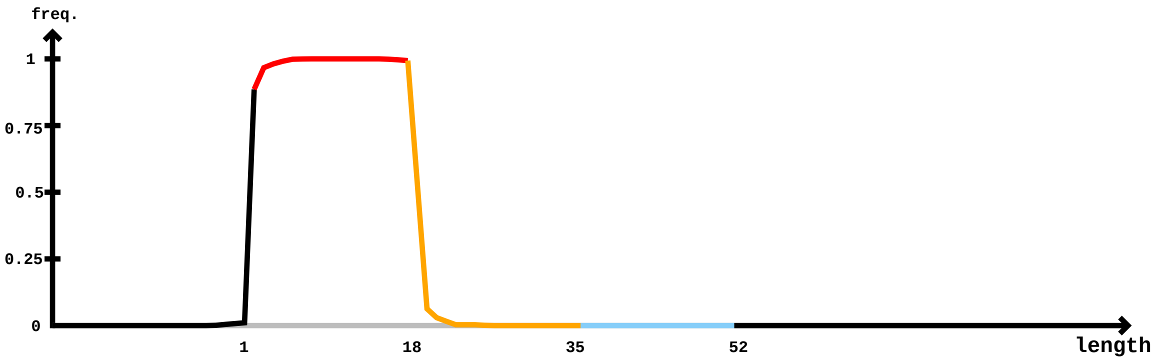

Mature **Star**

```
5'- aguuaagugcacucaggcaagaguaguacugggaugagugaccgcccgagaaguccuaguauuuuacaugccuauggcauuuuauaaacuuaguccaauuuaucccacau   -3'    exp
    ((((((((((.(..(((((.(...(((((((((((...((......))....)))))))))))...).)))))..)))))))....))))....................    reads      mm        sample
    ...............ggcaagaguaguacuggg.............................................................................    1          0         seq
    ...............ggcaagaguaguacugggaug..........................................................................    1          0         seq
    ................Acaagaguaguacuggg.............................................................................    1          1         seq
    ................gcaagaguaguacuggg.............................................................................    1          0         seq
    ................gcaagGguaguacuggga............................................................................    1          1         seq
    ................gcaagaguaguacuggga............................................................................    1          0         seq
    ................gcaagaguaguacugggau...........................................................................    2          0         seq
    ................gcaagaguaguacugggaug..........................................................................    2          0         seq
    .................caagaguaguacuggga............................................................................    3          0         seq
    .................caagaguaguacugggaug..........................................................................    4          0         seq
    ..................aagaguaguacugggau...........................................................................    2          0         seq
    ..................aagaguaguacugggaG...........................................................................    1          1         seq
    ..................Nagaguaguacugggau...........................................................................    2          1         seq
    ..................aagaguaguacugggGug..........................................................................    1          1         seq
    ..................Nagaguaguacugggaug..........................................................................    1          1         seq
    ...................Cgaguaguacugggaug..........................................................................    3          1         seq
    ...................Ngaguaguacugggaug..........................................................................    3          1         seq
    ...................agaguaguacugggaug..........................................................................    1946       0         seq
    ...................agaguaguacugggauga.........................................................................    41         0         seq
    ...................agGguaguacugggauga.........................................................................    3          1         seq
    ...................agaguaguacugggaugag........................................................................    8          0         seq
    ...................agGguaguacugggaugag........................................................................    1          1         seq
    ...................agaguaguacugggaugagug......................................................................    1          0         seq
    ...................agaguaguacugggaugaguC......................................................................    1          1         seq
    ....................Uaguaguacugggauga.........................................................................    1          1         seq
    ....................gaguGguacugggauga.........................................................................    1          1         seq
    ....................gUguaguacugggauga.........................................................................    1          1         seq
    ....................Naguaguacugggauga.........................................................................    1          1         seq
    ....................gaguaguacugggauga.........................................................................    114        0         seq
    ....................gGguaguacugggauga.........................................................................    5          1         seq
    ....................gaguaguacugggaAga.........................................................................    1          1         seq
    ....................gaguaguacugggauUa.........................................................................    1          1         seq
    ....................gaguaguacugggaGga.........................................................................    3          1         seq
    ....................gaguaguacCgggauga.........................................................................    1          1         seq
```

```
                   Mature                           Star
aguuaagugcacucaggcaagaguaguacugggaugagugaccgcccgagaaguccuaguauuuuacaugccuauggcauuuuauaaacuuaguccaauuuaucccacau
....................gaguaguaGugggaugag........................................................................	1	1	seq
....................gaguaguGcugggaugag........................................................................	1	1	seq
....................gGguaguacugggaugag........................................................................	2	1	seq
....................gaguaguacugggaugag........................................................................	39	0	seq
....................gaguaguacCgggaugag........................................................................	1	1	seq
....................gaguaguacuggAaugagu.......................................................................	3	1	seq
....................gaguaguacugggaugagu.......................................................................	4	0	seq
....................gaguaguacugggaugagug......................................................................	6	0	seq
.....................Gguaguacugggaugag........................................................................	1	1	seq
.....................aguaguacugggaugag........................................................................	22	0	seq
.....................aguaguacugggaugagC.......................................................................	2	1	seq
.....................aguaguacugggaugagu.......................................................................	4	0	seq
.....................aguaguacugggaugagGg......................................................................	1	1	seq
.....................aguaguacugggaugagug......................................................................	2	0	seq
.....................aguaguacugggaugUgugaccg..................................................................	1	1	seq
......................guaguacuggAaugagu.......................................................................	1	1	seq
......................guaguacugggaugagu.......................................................................	15	0	seq
......................guaguacugggaugagC.......................................................................	2	1	seq
......................guaguacugggaugagug......................................................................	4	0	seq
......................guaguacugggaugagugacc...................................................................	1	0	seq
.......................uaguacugggaugagug......................................................................	13	0	seq
.......................uaguacugggaugaUug......................................................................	1	1	seq
.......................uaAuacugggaugagug......................................................................	1	1	seq
.......................uaguacugggaugagugacc...................................................................	1	0	seq
.......................uaguacugggaugagugGcc...................................................................	1	1	seq
........................aguacugggaugagugacc...................................................................	1	0	seq
........................aguacugggaugagugaccC..................................................................	1	1	seq
.........................guacugggaugagugacc...................................................................	1	0	seq
```

Provisional ID	:	MA_167474_22779
Score total	:	262.3
Score for star read(s)	:	3.9
Score for read counts	:	260.6
Score for mfe	:	0
Score for randfold	:	-2.2
Score for cons. seed	:	
Total read count	:	523
Mature read count	:	522
Loop read count	:	0
Star read count	:	1

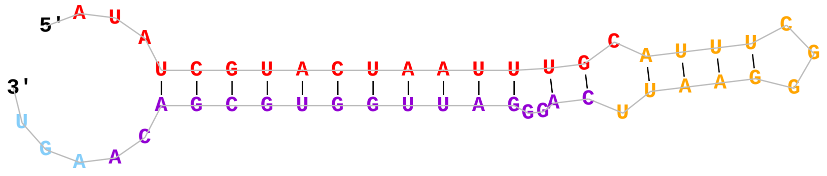

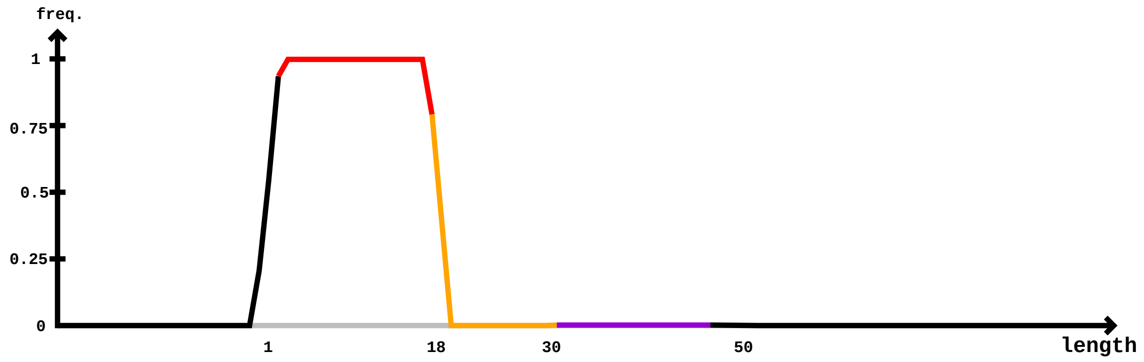

Mature **Star**

```
5'- caauucaacgagagucauuggauaucguacuaauuugcauuucgggaauucagggauuggugcgacaagugauuuuggggucgagagucgugaacucccgguucgcaa   -3'   obs
    caauucaacgagagucauuggauaucguacuaauuugcauuucgggaauucagggauuggugcgacaagugauuuuggggucgagagucgugaacucccgguucgcaa         exp
    ........(.(((((((((.....(((((((((((((.((((...)))).))..)))))))))))..)))))))))).)...........((((((.....))))))..   reads   mm   sample
    ...................Cgauaucguacuaauuug.......................................................................   85      1    seq
    ...................Cgauaucguacuaauuugc......................................................................   23      1    seq
    ....................gauaucguacuaauuug.......................................................................   23      0    seq
    ....................gauaucguacuaauuugG......................................................................   2       1    seq
    ....................gauaucguacuaauuugc......................................................................   15      0    seq
    ....................gauaucguacAaauuugc......................................................................   1       1    seq
    ....................gauaucguacuaauuugU......................................................................   101     1    seq
    ....................gauaucguacuaauuugA......................................................................   11      1    seq
    ....................gauaucguacuaauuugca.....................................................................   1       0    seq
    ....................gauaucguacuaauuugGa.....................................................................   1       1    seq
    ....................gauaucguacuaauuugcU.....................................................................   2       1    seq
    ....................gauaucguacuaauuugUa.....................................................................   17      1    seq
    ....................gauaucguacuaauuugAa.....................................................................   4       1    seq
    .....................auaucguacuaauuugU......................................................................   130     1    seq
    .....................auaucguacuaauuugG......................................................................   3       1    seq
    .....................auaucguacuaauuugc......................................................................   15      0    seq
    .....................auaucguacuaauuugA......................................................................   10      1    seq
    .....................auaucguacuaauuugAa.....................................................................   2       1    seq
    .....................auaucguacuaauuugcG.....................................................................   1       1    seq
    .....................auaucguacuaauuugUa.....................................................................   40      1    seq
    .....................auaucguacuaauuugca.....................................................................   1       0    seq
    .....................auaucguacuaauuugcU.....................................................................   1       1    seq
    ......................uaucguacuaauuugcU.....................................................................   1       1    seq
    ......................uaucguacuaauuugUa.....................................................................   24      1    seq
    ......................uaucguacuaauuugAa.....................................................................   8       1    seq
    ..................................................cagggauGggugcgaca.........................................   1       1    seq
```

Provisional ID	:	MA_170279_7179
Score total	:	0.4
Score for star read(s)	:	-1.3
Score for read counts	:	0
Score for mfe	:	0.1
Score for randfold	:	1.6
Score for cons. seed	:	
Total read count	:	6420
Mature read count	:	6420
Loop read count	:	0
Star read count	:	0

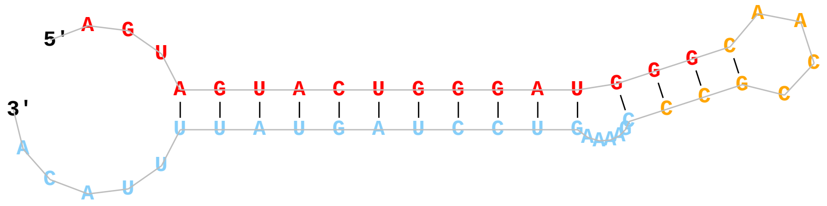

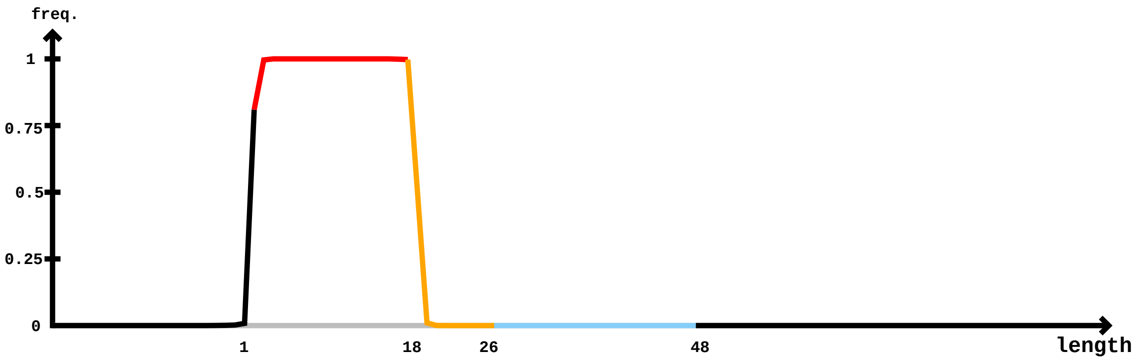


```
                    Mature          Star
5'- uuaagcaugcucaggauauaguaguacugggaugggcaaccgcccgaaaaguccuaguauuuuacaugccuauggcauuuuauaaauuuaguccaauuuaucccacau   -3'    exp
    .((((.(((((.(((.......((((((((((((((....)))).....))))))))))........))).)))))))))............................  reads    mm    sample
    ...............aGauaguaguacugggaug..........................................................................  1        1     seq
    ...............Cuauaguaguacugggaugggc.......................................................................  1        1     seq
    ................uauaguaguacugggauggA........................................................................  1        1     seq
    ................uaGaguaguacugggaugggca......................................................................  2        1     seq
    .................Cuaguaguacugggaug..........................................................................  2        1     seq
    .................auaguaguacugggaug..........................................................................  3        0     seq
    .................Nuaguaguacugggaug..........................................................................  1        1     seq
    .................Cuaguaguacugggauggg........................................................................  1        1     seq
    .................Nuaguaguacugggauggg........................................................................  1        1     seq
    ..................uaguaguacugggaugA.........................................................................  1        1     seq
    ..................uaguaguacugggaugg.........................................................................  5        0     seq
    ..................Naguaguacugggaugg.........................................................................  4        1     seq
    ..................uaguaguacugggauggg........................................................................  10       0     seq
    ..................Naguaguacugggauggg........................................................................  7        1     seq
    ..................uaguaguacugggaugggc.......................................................................  1        0     seq
    ..................uaguaguacugggaugggA.......................................................................  1        1     seq
    ..................Gaguaguacugggaugggca......................................................................  11       1     seq
    ...................aguaguacugggauggg........................................................................  4372     0     seq
    ...................Nguaguacugggauggg........................................................................  6        1     seq
    ...................aAuaguacugggaugggc.......................................................................  1        1     seq
    ...................aguaguacugggaCgggc.......................................................................  1        1     seq
    ...................aguaguacuggGugggc.......................................................................  6        1     seq
    ...................aguaguacugggauggCc.......................................................................  3        1     seq
    ...................aguaguacugggaAgggc.......................................................................  1        1     seq
    ...................aguaguacugggauggAc.......................................................................  1        1     seq
    ...................aguaguacAgggaugggc.......................................................................  1        1     seq
    ...................aguaAuacugggaugggc.......................................................................  1        1     seq
    ...................agCaguacugggaugggc.......................................................................  1        1     seq
    ...................aguaguacGgggaugggc.......................................................................  1        1     seq
    ...................aguaguacugggauCggc.......................................................................  2        1     seq
    ...................aguaguGcugggaugggc.......................................................................  1        1     seq
    ...................aguaguacugggaugggG.......................................................................  131      1     seq
    ...................Gguaguacugggaugggc.......................................................................  5        1     seq
    ...................aguaguacCgggaugggc.......................................................................  3        1     seq
```

Mature Star

```
uuaagcaugcucaggauauaguaguacugggaugggcaaccgcccgaaaaguccuaguauuuuacaugccuauggcauuuuauaaauuuaguccaauuuaucccacau
...................aguaguacugggaugAgc.......................................................................   2      1   seq
...................aguaguacugggaugggc.......................................................................   596    0   seq
...................aguaguacugggauCggca......................................................................   1      1   seq
...................aguaguacugggaugggcU......................................................................   2      1   seq
...................aguaguacugggaugggcC......................................................................   2      1   seq
...................aguaguacugggaugggGa......................................................................   1      1   seq
...................aguaguacugggaugggca......................................................................   7      0   seq
....................gCaguacugggaugggc.......................................................................   4      1   seq
....................Uuaguacugggaugggc.......................................................................   1      1   seq
....................guaguacugggaugAgc.......................................................................   2      1   seq
....................guaguacuAggaugggc.......................................................................   1      1   seq
....................guaguacuUggaugggc.......................................................................   1      1   seq
....................guaguacugUgaugggc.......................................................................   1      1   seq
....................guaguacugggaCgggc.......................................................................   2      1   seq
....................guaguacugggCugggc.......................................................................   1      1   seq
....................guaguacugggauggAc.......................................................................   3      1   seq
....................guGguacugggaugggc.......................................................................   5      1   seq
....................guaguacugggaGgggc.......................................................................   1      1   seq
....................Auaguacugggaugggc.......................................................................   1      1   seq
....................guaguacugggaugggc.......................................................................   1142   0   seq
....................guUguacugggaugggc.......................................................................   1      1   seq
....................Nuaguacugggaugggc.......................................................................   2      1   seq
....................guaguacugggaAgggc.......................................................................   2      1   seq
....................guaguacugggGugggc.......................................................................   4      1   seq
....................guaguacGgggaugggc.......................................................................   1      1   seq
....................guaguGcugggaugggc.......................................................................   8      1   seq
....................guaguacugggauggCc.......................................................................   2      1   seq
....................guaguacCgggaugggc.......................................................................   1      1   seq
....................guaguacugggaugggcU......................................................................   4      1   seq
....................guaguacugggaugggca......................................................................   5      0   seq
....................guaguacGgggaugggca......................................................................   1      1   seq
....................guaguacugggaugggcC......................................................................   3      1   seq
.....................uaguacugggaAgggca......................................................................   1      1   seq
.....................uaguacugggaugggca......................................................................   14     0   seq
.....................uaguacugggaugggcC......................................................................   3      1   seq
.....................uaguacugggaugggcU......................................................................   6      1   seq
.....................uaguacugggaugggcCa.....................................................................   1      1   seq
```

Provisional ID	:	MA_170758_16641
Score total	:	0.4
Score for star read(s)	:	-1.3
Score for read counts	:	0
Score for mfe	:	0.1
Score for randfold	:	1.6
Score for cons. seed	:	
Total read count	:	4937
Mature read count	:	4937
Loop read count	:	0
Star read count	:	0

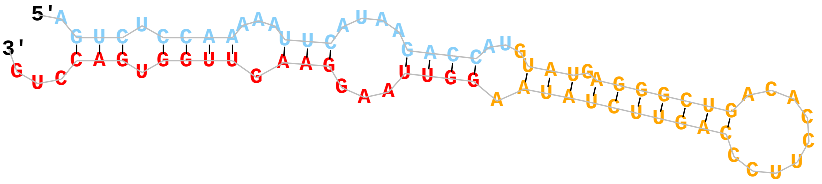

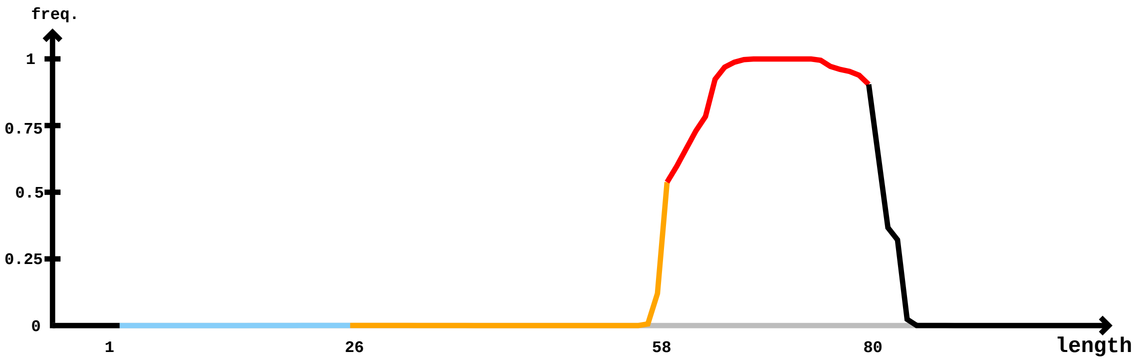

Star Mature

```
5'- acacaagucuccaaaaauucauaagaccauguaugagggcugacaccuucccaguucuauaagguuaaggaaguuggugaccugaugauggggaaaccaacgacugaa  -3'   exp
    .((((.(((.((((...(((....((((...(((.(((((((.........))))))))).))))...))).)))).))).)).))...((....))..........   reads   mm   sample
    ...................cGuaagaccauguauga........................................................................   1       1    seq
    ........................gaccauguaugagggcug..................................................................   1       0    seq
    ............................................................aagguuaaggaaguuggu..............................   2       0    seq
    ............................................................aagguuaaggaaguugguA.............................   1       1    seq
    ............................................................aagguuaaggaaguuggug.............................   4       0    seq
    ............................................................aagguuaaggaaguuggugа............................   1       0    seq
    ............................................................aagguuaaggaaguuggugac...........................   1       0    seq
    ............................................................aagguuGaggaaguuggugac...........................   1       1    seq
    ............................................................aagguuaaggaaguuggugaccuC........................   1       1    seq
    ............................................................aagguuaaggaaguuggugaccug........................   3       0    seq
    ............................................................Cagguuaaggaaguuggugaccug........................   1       1    seq
    ............................................................aagguuaaggaaguuggugaccuga.......................   2       0    seq
    ............................................................Gagguuaaggaaguuggugaccuga.......................   1       1    seq
    ............................................................aagguuaaggaaguuggugaccugaug.....................   12      0    seq
    .............................................................agguuaaggaaguuggu..............................   20      0    seq
    .............................................................agguuaUggaaguuggu..............................   1       1    seq
    .............................................................agguuaaggaaguuggC..............................   1       1    seq
    .............................................................agguuaaggaaguugguU.............................   1       1    seq
    .............................................................agguuaaggaaguuggug.............................   40      0    seq
    .............................................................agguuaaggaaguuggAg.............................   1       1    seq
    .............................................................agguuaaggaaguugguA.............................   2       1    seq
    .............................................................agguuaaggaaguuggCg.............................   1       1    seq
    .............................................................agguuaaggaaguuggGg.............................   1       1    seq
    .............................................................agguuaaggaaguuggugG............................   1       1    seq
    .............................................................agguuaaggaaguuggugа............................   21      0    seq
    .............................................................agAuuaaggaaguuggugа............................   1       1    seq
    .............................................................agguuaaggaaguuggugac...........................   15      0    seq
    .............................................................agguCaaggaaguuggugac...........................   1       1    seq
    .............................................................agguuaaggaaguuggugacc..........................   5       0    seq
    .............................................................agguuaaggaaguuggugaccu.........................   1       0    seq
    .............................................................agguuaaggaaguuggugaccA.........................   1       1    seq
    .............................................................agguuaaggaaguuggugaccCg........................   1       1    seq
    .............................................................agguuaaggaaguuggCgaccug........................   1       1    seq
    .............................................................agguuaaggaaguuggugaccug........................   96      0    seq
```

```
     Star                                                    Mature
acacaagucuccaaaaauucauaaagaccauguaugagggcugacaccuucccaguucuauaaggguuaaggaaguuggugaccugaugauggggaaaccaacgacugaa
..............................................................agguuaaggaaguuggugaUcug........................	1	1	seq
..............................................................agguuaGggaaguuggugaccug........................	1	1	seq
..............................................................agguuaaggaGguuggugaccug........................	1	1	seq
..............................................................agguuaaggaaguuggugaccuC........................	1	1	seq
..............................................................agguuaaggaaguuggugaccGg........................	1	1	seq
..............................................................aggCuaaggaaguuggugaccuga.......................	1	1	seq
..............................................................agguuaaggaaguuggugaccugG.......................	5	1	seq
..............................................................agguuaaggaaguuggugaccCga.......................	1	1	seq
..............................................................agguuaaggaGguuggugaccuga.......................	2	1	seq
..............................................................Ggguuaaggaaguuggugaccuga.......................	1	1	seq
..............................................................aggAuaaggaaguuggugaccuga.......................	1	1	seq
..............................................................agguuaaggaaguuggugUccuga.......................	1	1	seq
..............................................................agguuaaggaaguuggugaccuCa.......................	1	1	seq
..............................................................agguuaaggaaguuggugaccAga.......................	1	1	seq
..............................................................agguuaaggaaguuggugaccuga.......................	140	0	seq
..............................................................agguuaaggaagCuggugaccuga.......................	1	1	seq
..............................................................agguuaaggaaguuggugaccugaG......................	1	1	seq
..............................................................agguuaaggaaguuggugaccugau......................	16	0	seq
..............................................................agguuaaggaaguuggCgaccugau......................	1	1	seq
..............................................................aggCuaaggaaguuggugaccugau......................	1	1	seq
..............................................................agguuaaggaaguuggugaccugaC......................	2	1	seq
..............................................................Ngguuaaggaaguuggugaccugau......................	1	1	seq
..............................................................agguCaaggaaguuggugaccugaug.....................	1	1	seq
..............................................................agguuaaggaaguuggugaccugaug.....................	150	0	seq
..............................................................agguuaagUaaguuggugaccugaug.....................	1	1	seq
..............................................................agguuaaggaaguuggugGccugaug.....................	1	1	seq
..............................................................aAguuaaggaaguuggugaccugaug.....................	1	1	seq
..............................................................agguuaaggaaguugguUaccugaug.....................	1	1	seq
..............................................................agguuaaggaaguuggugaccugGug.....................	2	1	seq
..............................................................Ggguuaaggaaguuggugaccugaug.....................	1	1	seq
..............................................................Ugguuaaggaaguuggugaccugaug.....................	1	1	seq
..............................................................agguuaaggaaguuggugaccugaCg.....................	3	1	seq
..............................................................agguuaaggaaguuggugaccugauga....................	6	0	seq
..............................................................agguuaaggaaguuggugaccugaugG....................	2	1	seq
...............................................................gguuaGggaaguuggug.............................	1	1	seq
...............................................................gguuaaggaaguuggAg.............................	1	1	seq
...............................................................gguuaaggaaguugguA.............................	2	1	seq
...............................................................gguuaaggaaguuggug.............................	56	0	seq
...............................................................gguuaaggaaguugguU.............................	2	1	seq
...............................................................gguuaaggaaguuggugа............................	21	0	seq
...............................................................gguuaaggaaguuggugG............................	1	1	seq
...............................................................gguuaaggaaguuggugaU...........................	1	1	seq
...............................................................gguuaaggaaguuggugac...........................	13	0	seq
...............................................................gguuaaggaaguuggugGc...........................	1	1	seq
...............................................................gguuaaggaaguuggugacc..........................	5	0	seq
...............................................................gguuaaggaaguuggugaccG.........................	4	1	seq
...............................................................gguuaaggaaguuggCgaccu.........................	1	1	seq
...............................................................gguuaaggaaguuggugaccC.........................	7	1	seq
...............................................................gguuaaggaaguuggugUccu.........................	1	1	seq
...............................................................gguuaaggaaguuggugaccu.........................	40	0	seq
...............................................................gguuaaggaaguuggugaccCg........................	15	1	seq
...............................................................gguuaaggaaguCggugaccug........................	1	1	seq
...............................................................gguuaaggaaguuggugaccug........................	1157	0	seq
...............................................................Cguuaaggaaguuggugaccug........................	1	1	seq
...............................................................gguCaaggaaguuggugaccug........................	2	1	seq
...............................................................gguuaagUaaguuggugaccug........................	2	1	seq
...............................................................gguuaaggaGguuggugaccug........................	8	1	seq
...............................................................gguuaaggaaguuggGgaccug........................	2	1	seq
...............................................................gguuaaggaaguuggugaccAg........................	5	1	seq
...............................................................gguuaaggaaguuggugacAug........................	1	1	seq
...............................................................gguuaaggaaguuggugaccGg........................	10	1	seq
...............................................................ggGuaaggaaguuggugaccug........................	3	1	seq
...............................................................gguuaaggaaguuggugacGug........................	1	1	seq
...............................................................ggCuaaggaaguuggugaccug........................	2	1	seq
...............................................................gguuaaggGaguuggugaccug........................	3	1	seq
...............................................................gguGaaggaaguuggugaccug........................	1	1	seq
...............................................................gguuGaggaaguuggugaccug........................	4	1	seq
...............................................................gguuaaggaaguuggugaccuU........................	15	1	seq
...............................................................gguuaaggaaguuggCgaccug........................	4	1	seq
...............................................................gguuaGggaaguuggugaccug........................	5	1	seq
```

Star **Mature**

```
acacaagucuccaaaaauucauaagaccauguaugagggcugacaccuucccaguucuauaagguuaaggaaguuggugaccugaugauggggaaaccaacgacugaa
..............................................................gguuaaggaaguuggugaccuC........................    8     1    seq
..............................................................gguuUaggaaguuggugaccug........................    1     1    seq
..............................................................gguuaaggaaguugAugaccug........................    1     1    seq
..............................................................gguuaaggaaguuggugGccug........................    7     1    seq
..............................................................gguuaUggaaguuggugaccug........................    1     1    seq
..............................................................gguuaaggaaguuUgugaccug........................    1     1    seq
..............................................................Aguuaaggaaguuggugaccug........................    1     1    seq
..............................................................gguuaaggaaguuggugaccugG.......................    2     1    seq
..............................................................gguuaaggaaguuggugaccugC.......................    1     1    seq
..............................................................gguuGaggaaguuggugaccuga.......................    1     1    seq
..............................................................ggGuaaggaaguuggugaccuga.......................    1     1    seq
..............................................................Aguuaaggaaguuggugaccuga.......................    1     1    seq
..............................................................gguuaaggaaguuggugaccuga.......................    125   0    seq
..............................................................gguuaaggaaguuggugaccugaC......................    7     1    seq
..............................................................gguuaaggaaguAggugaccugau......................    1     1    seq
..............................................................gguGaaggaaguuggugaccugau......................    1     1    seq
..............................................................gguuaaggaaguuggugaccugau......................    58    0    seq
..............................................................gguuaaggaaguuggugaccugUu......................    1     1    seq
..............................................................gguuaaggaaguCggugaccugaug.....................    1     1    seq
..............................................................gguuaaggaaguuggugaccugGug.....................    1     1    seq
..............................................................gguuaaggaaguuggugaccugaug.....................    396   0    seq
..............................................................gguuaaggaaguuggCgaccugaug.....................    3     1    seq
..............................................................gguuaGggaaguuggugaccugaug.....................    2     1    seq
..............................................................gguuaaggaagCuggugaccugaug.....................    1     1    seq
..............................................................gguuaaggaCguuggugaccugaug.....................    1     1    seq
..............................................................gguuaaggaaguuggugaccGgaug.....................    1     1    seq
..............................................................gguuaaggaagAuggugaccugaug.....................    1     1    seq
..............................................................gguuaaggaaguuggugaccugaGg.....................    1     1    seq
..............................................................gguuaaggaaguuggugGccugaug.....................    3     1    seq
..............................................................gguuaaggaaAuuggugaccugaug.....................    1     1    seq
..............................................................gguuaaggaGguuggugaccugaug.....................    2     1    seq
..............................................................gguuaaggaaguuggugaccugaCg.....................    1     1    seq
..............................................................Aguuaaggaaguuggugaccugaug.....................    1     1    seq
..............................................................gguuaaggaaguuggugaccCgaug.....................    1     1    seq
..............................................................gguuaaggGaguuggugaccugaug.....................    2     1    seq
..............................................................gguuaaggaaguuggugaccugaugU....................    1     1    seq
..............................................................gguuaaggaaguuggugaccugCuga....................    1     1    seq
..............................................................gguuaaggaaguuggugaccugauga....................    23    0    seq
...............................................................guuaaggaaguuggguga............................    9     0    seq
...............................................................guuaaggaaguuggugaU...........................    1     1    seq
...............................................................guuaaggaaguuggugac...........................    1     0    seq
...............................................................guuaaggaaguuggugacc..........................    12    0    seq
...............................................................Uuuaaggaaguuggugacc..........................    1     1    seq
...............................................................guuaaggaaguuggugacA..........................    1     1    seq
...............................................................guCaaggaaguuggugaccu.........................    1     1    seq
...............................................................guuaaggGaguuggugaccu.........................    1     1    seq
...............................................................guuaaggaaguuggugaccG.........................    1     1    seq
...............................................................guuaaggaaguuggugaccC.........................    3     1    seq
...............................................................guuaaggaaguuggugaccu.........................    20    0    seq
...............................................................guuaaggGaguuggugaccug........................    1     1    seq
...............................................................guuaaggaaguuggugaccCg........................    2     1    seq
...............................................................guuaaggaGguuggugaccug........................    1     1    seq
...............................................................guuaaggaaguuggugaccuU........................    1     1    seq
...............................................................guuaUggaaguuggugaccug........................    1     1    seq
...............................................................guuaaggaaguugguUaccug........................    1     1    seq
...............................................................guuaaggaaguuggugaccug........................    88    0    seq
...............................................................Auuaaggaaguuggugaccug........................    1     1    seq
...............................................................guuaaggaaguuggugacGug........................    1     1    seq
...............................................................guuaaggaaguuggCgaccug........................    2     1    seq
...............................................................guuaaggaaguuggugaccAg........................    1     1    seq
...............................................................Uuuaaggaaguuggugaccug........................    1     1    seq
...............................................................guuaUggaaguuggugaccuga.......................    1     1    seq
...............................................................guuaaggaaguuggugaccugG.......................    2     1    seq
...............................................................guuaaggaaguuggugaccuga.......................    13    0    seq
...............................................................guuaaggaGguuggugaccugau......................    1     1    seq
...............................................................guuaaggaaguuggugaccugaC......................    4     1    seq
...............................................................guuaaggaaguuAgugaccugau......................    1     1    seq
...............................................................guuaaggaaguuggugaccugGu......................    1     1    seq
...............................................................guuaaggaaguuggugaccugau......................    30    0    seq
...............................................................guuaaggaaguuggCgaccugaug.....................    1     1    seq
```

```
     Star                                                 Mature
acacaagucuccaaaaauucauaagaccauguaugagggcugacaccuucccaguucuauaagguuaaggaaguuggugaccugaugauggggaaaccaacgacugaa
...............................................................gCuaaggaaguuggugaccugaug.....................   1    1    seq
...............................................................Auuaaggaaguuggugaccugaug.....................   1    1    seq
...............................................................guuaaggaaguuggugaccugaug.....................   80   0    seq
...............................................................guuaaggaaguuggugaccugaCg.....................   1    1    seq
...............................................................guuaaggaaguuggugaccugauga....................   4    0    seq
................................................................uuaaggaaguuggugac...........................   3    0    seq
................................................................uuaaggaaguCggugacc..........................   1    1    seq
................................................................uuaaggaaguuggugacc..........................   21   0    seq
................................................................uuaaggaaguuggugaccG.........................   1    1    seq
................................................................uuaaggaaguuggugaccC.........................   6    1    seq
................................................................uuaaggaaguuggugaccA.........................   3    1    seq
................................................................uuaaggaaguuggugaccu.........................   27   0    seq
................................................................uuaaggaaguuggugaccug........................   92   0    seq
................................................................uuaaggaaguuggugaccAg........................   1    1    seq
................................................................uuaaggGaguuggugaccug........................   1    1    seq
................................................................Nuaaggaaguuggugaccug........................   1    1    seq
................................................................uuaaggaaguuggugaccGg........................   1    1    seq
................................................................uuaaggaaguuggugacGug........................   1    1    seq
................................................................uuaaggaaguuggugaccuU........................   2    1    seq
................................................................uuaaggaaguuggugaccCg........................   2    1    seq
................................................................uuaGggaaguuggugaccug........................   1    1    seq
................................................................uuaaggaaguCggugaccug........................   1    1    seq
................................................................uuaaggaaguuggugaccuga.......................   24   0    seq
................................................................uuaaggaaguuggugaccugG.......................   1    1    seq
................................................................uuaaggaaguuggugaccugau......................   9    0    seq
................................................................uuaaggaaguuggugaccugaC......................   2    1    seq
................................................................uuaaggaaguugguUaccugaug.....................   1    1    seq
................................................................uuaaggaaguuggugaccugGug.....................   2    1    seq
................................................................uuaaggaaguuggugaccugaCg.....................   2    1    seq
................................................................uuaaggaaguuggugaccugaug.....................   112  0    seq
................................................................uuaaggaaguugUugaccugaug.....................   2    1    seq
................................................................uuaaggaaguuggCgaccugaug.....................   1    1    seq
................................................................uCaaggaaguuggugaccugaug.....................   1    1    seq
................................................................uuaaggaaguugAugaccugaug.....................   1    1    seq
................................................................uuGaggaaguuggugaccugauga....................   1    1    seq
................................................................uuaaggaaguuggugaccugauga....................   4    0    seq
.................................................................uaaggaaguuggugacc..........................   24   0    seq
.................................................................uaaggaagGuggugacc..........................   1    1    seq
.................................................................uaaggaaguuggugaccu.........................   14   0    seq
.................................................................uaaggaaguuggugaccG.........................   2    1    seq
.................................................................uaaggaaguuggugaccA.........................   1    1    seq
.................................................................uaaggaaguuggugaccC.........................   7    1    seq
.................................................................uaaggGaguuggugaccug........................   1    1    seq
.................................................................uaaggaaguuggugaccug........................   75   0    seq
.................................................................uaaggaaguuggugaccCg........................   1    1    seq
.................................................................Aaaggaaguuggugaccug........................   1    1    seq
.................................................................Caaggaaguuggugaccug........................   1    1    seq
.................................................................Caaggaaguuggugaccuga.......................   2    1    seq
.................................................................uaGggaaguuggugaccuga.......................   1    1    seq
.................................................................uaaggaaguuCgugaccuga.......................   1    1    seq
.................................................................uaaggaaguuggugaccuga.......................   25   0    seq
.................................................................uaaggaaguuggugaccugaA......................   1    1    seq
.................................................................uaaggaaguuggugaccugau......................   8    0    seq
.................................................................uaaggaaguuggugaccugaCg.....................   2    1    seq
.................................................................uaaggaaguuggCgaccugaug.....................   1    1    seq
.................................................................uaaggaaguuggugaccugGug.....................   1    1    seq
.................................................................uaaggaaguuggugaccugaug.....................   148  0    seq
.................................................................uaaggaaguuggugaccugauU....................   1    1    seq
.................................................................Gaaggaaguuggugaccugaug.....................   1    1    seq
.................................................................uaaggaaguuggugGccugaug.....................   1    1    seq
.................................................................uaaggaaguuggugaccugauga....................   7    0    seq
..................................................................aaggaaguuggugaccC.........................   3    1    seq
..................................................................aaggaaguuggugaccu.........................   20   0    seq
..................................................................aaggaaguuggugaccG.........................   2    1    seq
..................................................................aaggaaguuggugaccA.........................   2    1    seq
..................................................................aaggGaguuggugaccu.........................   1    1    seq
..................................................................aaggaaguuggugaccGg........................   1    1    seq
..................................................................aaggaGguuggugaccug........................   1    1    seq
..................................................................aaggaaguugguAaccug........................   1    1    seq
..................................................................aaggaaguuggugaccug........................   109  0    seq
```

Star Mature

acacaagucuccaaaaauucauaagaccauguaugagggcugacaccuucccaguucuauaagguuaaggaaguuggugaccugaugauggggaaaccaacgacugaa

....aaggaagCuggugaccug........................ 1 1 seq
....aaggaaguuggCgaccug........................ 1 1 seq
....Gaggaaguuggugaccug........................ 1 1 seq
....aaggGaguuggugaccug........................ 1 1 seq
....aaggaaguuggugGccug........................ 1 1 seq
....aaggaaguuggugaccuU........................ 1 1 seq
....aaggaaguuggugaccuC........................ 1 1 seq
....aaggaaguuggugaccAg........................ 2 1 seq
....aGggaaguuggugaccug........................ 1 1 seq
....aaggaaguuggugaccuga....................... 30 0 seq
....aaggGaguuggugaccuga....................... 1 1 seq
....aaggaaguuggugaccugau...................... 6 0 seq
....aaggaaguuggugaccugaug..................... 70 0 seq
....aaggaaguGggugaccugaug..................... 1 1 seq
....aaggaaguuggugaccugGug..................... 2 1 seq
....aaggaGguuggugaccugaug..................... 1 1 seq
....aaggaaguuggugaccugauga.................... 6 0 seq
....aggaaguuggugaccuU........................ 6 1 seq
....aggaagCuggugaccug........................ 1 1 seq
....aggaaguuggGgaccug........................ 1 1 seq
....aggaaguuggugaccGg........................ 2 1 seq
....aggaaguugguAaccug........................ 1 1 seq
....aggaaguuggugaccAg........................ 1 1 seq
....aggaaguuggAgaccug........................ 1 1 seq
....Nggaaguuggugaccug........................ 1 1 seq
....aggaaguuggugaccCg........................ 2 1 seq
....aggGaguuggugaccug........................ 2 1 seq
....aggaaguuggugGccug........................ 1 1 seq
....aggaaguuggugacAug........................ 1 1 seq
....aggaaguuggugaccug........................ 287 0 seq
....aggaaguuggCgaccug........................ 1 1 seq
....aggaaguCggugaccug........................ 1 1 seq
....aggaaguuggugaccuC........................ 1 1 seq
....aggaGguuggugaccug........................ 4 1 seq
....Gggaaguuggugaccug........................ 2 1 seq
....aggaaguuggugaccugG....................... 2 1 seq
....aggaGguuggugaccuga....................... 3 1 seq
....aggaaguuggugaccuga....................... 111 0 seq
....aggaaguAggugaccuga....................... 1 1 seq
....aggaaguuggugaccugaG...................... 2 1 seq
....aggaaguuggugaccugaC...................... 3 1 seq
....aggaaguuggugaccugau...................... 25 0 seq
....aggaGguuggugaccugaug..................... 1 1 seq
....Gggaaguuggugaccugaug..................... 1 1 seq
....aggaaguuggugaccugaCg..................... 4 1 seq
....aggaaguuggugaccCgaug..................... 1 1 seq
....agAaaguuggugaccugaug..................... 1 1 seq
....aggaaguuggugaccugaGg..................... 1 1 seq
....aggaaguuggugaccugaug..................... 195 0 seq
....aggaaguuggugaccugCug..................... 1 1 seq
....aggGaguuggugaccugaug..................... 1 1 seq
....aggaaguuggugGccugaug..................... 1 1 seq
....aggaaguuggugaccugaugU.................... 1 1 seq
....aggaaguuggugaccugaugG.................... 1 1 seq
....aggaaguuggugGccugauga.................... 1 1 seq
....aggaaguuggugaccugauga.................... 16 0 seq
....aggaaguuggugaccugaugauggg................ 1 0 seq
....ggaaguuggugaccuga....................... 51 0 seq
....ggaaguuggugaccuUa....................... 1 1 seq
....ggaaguuggugaccugG....................... 2 1 seq
....ggaaguuggugaccugU....................... 1 1 seq
....ggaaguuggugaccugau...................... 26 0 seq
....ggaaguuggugaccugaC...................... 1 1 seq
....ggaaguuggugaccugaCg..................... 1 1 seq
....ggGaguuggugaccugaug..................... 1 1 seq
....ggaaguuggugaccugaGg..................... 2 1 seq
....ggaaguugAugaccugaug..................... 1 1 seq
....ggaaguuggugaccugaug..................... 116 0 seq
....ggaaguuggugGccugaug..................... 1 1 seq
....ggaaguuggugaccCgaug..................... 2 1 seq

Star **Mature**

```
acacaagucuccaaaaauucauaagaccauguaugagggcugacaccuucccaguucuauaagguuaaggaaguuggugaccugaugauggggaaaccaacgacugaa
....................................................................ggaaguuggugaccGgauga....................	1	1	seq
....................................................................ggaaguuggugaccugauga....................	17	0	seq
....................................................................ggaaguuggugaccugaugU....................	1	1	seq
.....................................................................gaaguuggugaccugaC......................	3	1	seq
.....................................................................gaaguuggugaccugau......................	13	0	seq
.....................................................................gaaguuggCgaccugaug.....................	1	1	seq
.....................................................................gaagCuggugaccugaug.....................	1	1	seq
.....................................................................gUaguuggugaccugaug.....................	1	1	seq
.....................................................................gaaguuggugaccugGug.....................	1	1	seq
.....................................................................gaaguuggugaccugaCg.....................	2	1	seq
.....................................................................Naaguuggugaccugaug.....................	1	1	seq
.....................................................................gaaguuggugaccugaug.....................	63	0	seq
.....................................................................gaaguuggugaccugauga....................	5	0	seq
......................................................................aaguuggugaccugGug.....................	1	1	seq
......................................................................aaguuggugaccugaug.....................	42	0	seq
......................................................................aaguuggugaccugauga....................	5	0	seq
.......................................................................aguuggugaccugaGga....................	1	1	seq
.......................................................................aguuggugaccugaugU....................	1	1	seq
.......................................................................aguuggugaccugauga....................	10	0	seq
```

Provisional ID	:	MA_170758_16642
Score total	:	0
Score for star read(s)	:	-1.3
Score for read counts	:	0
Score for mfe	:	-0.2
Score for randfold	:	1.6
Score for cons. seed	:	
Total read count	:	4937
Mature read count	:	4937
Loop read count	:	0
Star read count	:	0

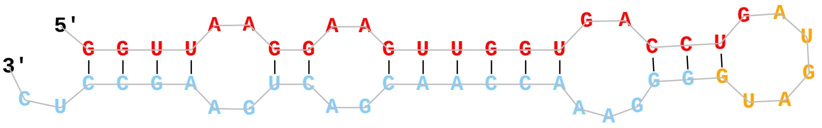

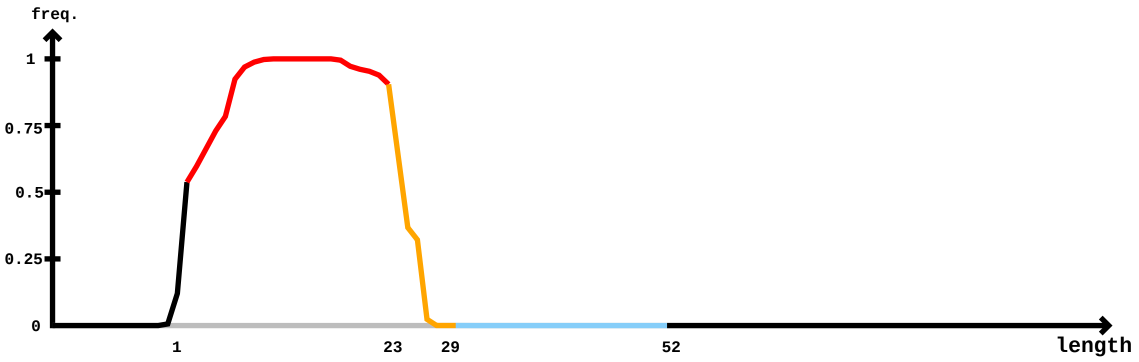

Mature **Star**

```
5'- ccaguucuauaagguuaaggaaguuggugaccugaugauggggaaaccaacgacugaagccucuaugaauuguugucacaacuauaaauuucuucaggcagaaaaauu   -3'    exp
    .((((((.((((((((..((..((((((..(((......)))...))))))..))..))))).)))))))))................(((((......)))))....    reads   mm   sample
    ..........aagguuaaggaaguuggu................................................................................    2       0    seq
    ..........aagguuaaggaaguuggug...............................................................................    4       0    seq
    ..........aagguuaaggaaguugguA...............................................................................    1       1    seq
    ..........aagguuaaggaaguuggugа..............................................................................    1       0    seq
    ..........aagguuGaggaaguuggugac.............................................................................    1       1    seq
    ..........aagguuaaggaaguuggugac.............................................................................    1       0    seq
    ..........Cagguuaaggaaguuggugaccug..........................................................................    1       1    seq
    ..........aagguuaaggaaguuggugaccuC..........................................................................    1       1    seq
    ..........aagguuaaggaaguuggugaccug..........................................................................    3       0    seq
    ..........aagguuaaggaaguuggugaccuga.........................................................................    2       0    seq
    ..........Gagguuaaggaaguuggugaccuga.........................................................................    1       1    seq
    ..........aagguuaaggaaguuggugaccugaug.......................................................................    12      0    seq
    ...........agguuaUggaaguuggu................................................................................    1       1    seq
    ...........agguuaaggaaguuggC................................................................................    1       1    seq
    ...........agguuaaggaaguuggu................................................................................    20      0    seq
    ...........agguuaaggaaguuggGg...............................................................................    1       1    seq
    ...........agguuaaggaaguugguA...............................................................................    2       1    seq
    ...........agguuaaggaaguuggAg...............................................................................    1       1    seq
    ...........agguuaaggaaguuggug...............................................................................    40      0    seq
    ...........agguuaaggaaguuggCg...............................................................................    1       1    seq
    ...........agguuaaggaaguugguU...............................................................................    1       1    seq
    ...........agguuaaggaaguuggugа..............................................................................    21      0    seq
    ...........agguuaaggaaguuggugG..............................................................................    1       1    seq
    ...........agAuuaaggaaguuggugа..............................................................................    1       1    seq
    ...........agguuaaggaaguuggugac.............................................................................    15      0    seq
    ...........agguCaaggaaguuggugac.............................................................................    1       1    seq
    ...........agguuaaggaaguuggugacc............................................................................    5       0    seq
    ...........agguuaaggaaguuggugaccA...........................................................................    1       1    seq
    ...........agguuaaggaaguuggugaccu...........................................................................    1       0    seq
    ...........agguuaaggaaguuggCgaccug..........................................................................    1       1    seq
    ...........agguuaaggaGguuggugaccug..........................................................................    1       1    seq
    ...........agguuaaggaaguuggugaccCg..........................................................................    1       1    seq
    ...........agguuaaggaaguuggugaUcug..........................................................................    1       1    seq
    ...........agguuaaggaaguuggugaccGg..........................................................................    1       1    seq
```

Mature **Star**

```
ccaguucuauaaggguuaaggaaguuggugaccugaugauggggaaaccaacgacugaagccucuaugaauuguugucacaacuauaaauuucuucaggcagaaaaauu
...........agguuaaggaaguuggugaccug..........................................................................    96     0    seq
...........agguuaaggaaguuggugaccuC..........................................................................    1      1    seq
...........agguuaGggaaguuggugaccug..........................................................................    1      1    seq
...........agguuaaggaaguuggugaccuCa.........................................................................    1      1    seq
...........agguuaaggaagCuggugaccuga.........................................................................    1      1    seq
...........aggAuaaggaaguuggugaccuga.........................................................................    1      1    seq
...........agguuaaggaaguuggugaccCga.........................................................................    1      1    seq
...........agguuaaggaaguuggugaccuga.........................................................................    140    0    seq
...........aggCuaaggaaguuggugaccuga.........................................................................    1      1    seq
...........Ggguuaaggaaguuggugaccuga.........................................................................    1      1    seq
...........agguuaaggaaguuggugaccugG.........................................................................    5      1    seq
...........agguuaaggaaguuggugUccuga.........................................................................    1      1    seq
...........agguuaaggaGguuggugaccuga.........................................................................    2      1    seq
...........agguuaaggaaguuggugaccAga.........................................................................    1      1    seq
...........agguuaaggaaguuggugaccugaG........................................................................    1      1    seq
...........agguuaaggaaguuggCgaccugau........................................................................    1      1    seq
...........aggCuaaggaaguuggugaccugau........................................................................    1      1    seq
...........Ngguuaaggaaguuggugaccugau........................................................................    1      1    seq
...........agguuaaggaaguuggugaccugau........................................................................    16     0    seq
...........agguuaaggaaguuggugaccugaC........................................................................    2      1    seq
...........agguuaagUaaguuggugaccugaug.......................................................................    1      1    seq
...........aAguuaaggaaguuggugaccugaug.......................................................................    1      1    seq
...........agguuaaggaaguuggugaccugaCg.......................................................................    3      1    seq
...........agguuaaggaaguuggugaccugGug.......................................................................    2      1    seq
...........agguCaaggaaguuggugaccugaug.......................................................................    1      1    seq
...........agguuaaggaaguuggugaccugaug.......................................................................    150    0    seq
...........Ggguuaaggaaguuggugaccugaug.......................................................................    1      1    seq
...........Ugguuaaggaaguuggugaccugaug.......................................................................    1      1    seq
...........agguuaaggaaguugguUaccugaug.......................................................................    1      1    seq
...........agguuaaggaaguuggugGccugaug.......................................................................    1      1    seq
...........agguuaaggaaguuggugaccugauga......................................................................    6      0    seq
...........agguuaaggaaguuggugaccugaugG......................................................................    2      1    seq
............gguuaaggaaguugguU...............................................................................    2      1    seq
............gguuaaggaaguuggAg...............................................................................    1      1    seq
............gguuaGggaaguuggug...............................................................................    1      1    seq
............gguuaaggaaguugguA...............................................................................    2      1    seq
............gguuaaggaaguuggug...............................................................................    56     0    seq
............gguuaaggaaguuggugа..............................................................................    21     0    seq
............gguuaaggaaguuggugG..............................................................................    1      1    seq
............gguuaaggaaguuggugGc.............................................................................    1      1    seq
............gguuaaggaaguuggugaU.............................................................................    1      1    seq
............gguuaaggaaguuggugac.............................................................................    13     0    seq
............gguuaaggaaguuggugacc............................................................................    5      0    seq
............gguuaaggaaguuggugaccu...........................................................................    40     0    seq
............gguuaaggaaguuggugUccu...........................................................................    1      1    seq
............gguuaaggaaguuggugaccG...........................................................................    4      1    seq
............gguuaaggaaguuggCgaccu...........................................................................    1      1    seq
............gguuaaggaaguuggugaccC...........................................................................    7      1    seq
............gguuaaggGaguuggugaccug..........................................................................    3      1    seq
............gguuaaggaaguuUgugaccug..........................................................................    1      1    seq
............gguuaaggaaguuggugaccGg..........................................................................    10     1    seq
............gguuaaggaaguuggGgaccug..........................................................................    2      1    seq
............gguCaaggaaguuggugaccug..........................................................................    2      1    seq
............gguuaaggaaguuggugaccuC..........................................................................    8      1    seq
............gguuaaggaaguuggugaccCg..........................................................................    15     1    seq
............gguuaaggaaguuggugaccAg..........................................................................    5      1    seq
............gguGaaggaaguuggugaccug..........................................................................    1      1    seq
............gguuaaggaGguuggugaccug..........................................................................    8      1    seq
............ggGuaaggaaguuggugaccug..........................................................................    3      1    seq
............gguuaUggaaguuggugaccug..........................................................................    1      1    seq
............gguuaaggaaguuggugGccug..........................................................................    7      1    seq
............Cguuaaggaaguuggugaccug..........................................................................    1      1    seq
............gguuaaggaaguuggugacAug..........................................................................    1      1    seq
............gguuaaggaaguuggugacGug..........................................................................    1      1    seq
............gguuaGggaaguuggugaccug..........................................................................    5      1    seq
............gguuUaggaaguuggugaccug..........................................................................    1      1    seq
............Aguuaaggaaguuggugaccug..........................................................................    1      1    seq
............gguuaaggaaguuggugaccug..........................................................................    1157   0    seq
............gguuaaggaaguuggugaccuU..........................................................................    15     1    seq
............gguuaaggaaguuggCgaccug..........................................................................    4      1    seq
```

```
                        Mature                    Star
ccaguucuauaagguuaaggaaguuggugaccugaugauggggaaaccaacgacugaagccucuaugaauuguugucacaacuauaaauuucuucaggcagaaaaauu
............ggCuaaggaaguuggugaccug..........................................................................    2     1     seq
............gguuGaggaaguuggugaccug..........................................................................    4     1     seq
............gguuaaggaaguCggugaccug..........................................................................    1     1     seq
............gguuaagUaaguuggugaccug..........................................................................    2     1     seq
............gguuaaggaaguugAugaccug..........................................................................    1     1     seq
............gguuaaggaaguuggugaccugG.........................................................................    2     1     seq
............Aguuaaggaaguuggugaccuga.........................................................................    1     1     seq
............gguuaaggaaguuggugaccuga.........................................................................    125   0     seq
............gguuGaggaaguuggugaccuga.........................................................................    1     1     seq
............ggGuaaggaaguuggugaccuga.........................................................................    1     1     seq
............gguuaaggaaguuggugaccugC.........................................................................    1     1     seq
............gguuaaggaaguAggugaccugau........................................................................    1     1     seq
............gguGaaggaaguuggugaccugau........................................................................    1     1     seq
............gguuaaggaaguuggugaccugUu........................................................................    1     1     seq
............gguuaaggaaguuggugaccugau........................................................................    58    0     seq
............gguuaaggaaguuggugaccugaC........................................................................    7     1     seq
............gguuaaggaaguuggugaccGgaug.......................................................................    1     1     seq
............gguuaaggaagAuggugaccugaug.......................................................................    1     1     seq
............gguuaaggaaguCggugaccugaug.......................................................................    1     1     seq
............gguuaaggaaAuuggugaccugaug.......................................................................    1     1     seq
............gguuaGggaaguuggugaccugaug.......................................................................    2     1     seq
............gguuaaggaagCuggugaccugaug.......................................................................    1     1     seq
............gguuaaggaaguuggugaccugaug.......................................................................    396   0     seq
............Aguuaaggaaguuggugaccugaug.......................................................................    1     1     seq
............gguuaaggaaguuggugaccCgaug.......................................................................    1     1     seq
............gguuaaggaaguuggugaccugGug.......................................................................    1     1     seq
............gguuaaggaCguuggugaccugaug.......................................................................    1     1     seq
............gguuaaggGaguuggugaccugaug.......................................................................    2     1     seq
............gguuaaggaaguuggugaccugaCg.......................................................................    1     1     seq
............gguuaaggaGguuggugaccugaug.......................................................................    2     1     seq
............gguuaaggaaguuggugGccugaug.......................................................................    3     1     seq
............gguuaaggaaguuggugaccugaGg.......................................................................    1     1     seq
............gguuaaggaaguuggCgaccugaug.......................................................................    3     1     seq
............gguuaaggaaguuggugaccugCuga......................................................................    1     1     seq
............gguuaaggaaguuggugaccugaugU......................................................................    1     1     seq
............gguuaaggaaguuggugaccugauga......................................................................    23    0     seq
.............guuaaggaaguugguga..............................................................................    9     0     seq
.............guuaaggaaguuggugaU.............................................................................    1     1     seq
.............guuaaggaaguuggugac.............................................................................    1     0     seq
.............guuaaggaaguuggugacA............................................................................    1     1     seq
.............guuaaggaaguuggugacc............................................................................    12    0     seq
.............Uuuaaggaaguuggugacc............................................................................    1     1     seq
.............guCaaggaaguuggugaccu...........................................................................    1     1     seq
.............guuaaggaaguuggugaccC...........................................................................    3     1     seq
.............guuaaggaaguuggugaccu...........................................................................    20    0     seq
.............guuaaggaaguuggugaccG...........................................................................    1     1     seq
.............guuaaggGaguuggugaccu...........................................................................    1     1     seq
.............guuaUggaaguuggugaccug..........................................................................    1     1     seq
.............guuaaggaaguugguUaccug..........................................................................    1     1     seq
.............Uuuaaggaaguuggugaccug..........................................................................    1     1     seq
.............guuaaggaaguuggugaccuU..........................................................................    1     1     seq
.............guuaaggaaguuggugaccug..........................................................................    88    0     seq
.............guuaaggaGguuggugaccug..........................................................................    1     1     seq
.............guuaaggaaguuggCgaccug..........................................................................    2     1     seq
.............Auuaaggaaguuggugaccug..........................................................................    1     1     seq
.............guuaaggaaguuggugaccAg..........................................................................    1     1     seq
.............guuaaggaaguuggugacGug..........................................................................    1     1     seq
.............guuaaggaaguuggugaccCg..........................................................................    2     1     seq
.............guuaaggGaguuggugaccug..........................................................................    1     1     seq
.............guuaaggaaguuggugaccugG.........................................................................    2     1     seq
.............guuaaggaaguuggugaccuga.........................................................................    13    0     seq
.............guuaUggaaguuggugaccuga.........................................................................    1     1     seq
.............guuaaggaaguuggugaccugaC........................................................................    4     1     seq
.............guuaaggaaguuggugaccugau........................................................................    30    0     seq
.............guuaaggaGguuggugaccugau........................................................................    1     1     seq
.............guuaaggaaguuAgugaccugau........................................................................    1     1     seq
.............guuaaggaaguuggugaccugGu........................................................................    1     1     seq
.............guuaaggaaguuggCgaccugaug.......................................................................    1     1     seq
.............guuaaggaaguuggugaccugaCg.......................................................................    1     1     seq
.............gCuaaggaaguuggugaccugaug.......................................................................    1     1     seq
```

Mature **Star**

ccaguucuauaagguuaaggaaguuggugaccugaugauggggaaaccaacgacugaagccucuaugaauuguugucacaacuauaaauuucuucaggcagaaaaauu			
.............guuaaggaaguuggugaccugaug...	80	0	seq
.............Auuaaggaaguuggugaccugaug...	1	1	seq
.............guuaaggaaguuggugaccugauga..	4	0	seq
..............uuaaggaaguuggugac...	3	0	seq
..............uuaaggaaguCggugacc..	1	1	seq
..............uuaaggaaguuggugacc..	21	0	seq
..............uuaaggaaguuggugaccC...	6	1	seq
..............uuaaggaaguuggugaccA...	3	1	seq
..............uuaaggaaguuggugaccG...	1	1	seq
..............uuaaggaaguuggugaccu...	27	0	seq
..............uuaaggaaguuggugacGug..	1	1	seq
..............uuaaggaaguuggugaccGg..	1	1	seq
..............uuaaggaaguuggugaccAg..	1	1	seq
..............Nuaaggaaguuggugaccug..	1	1	seq
..............uuaGggaaguuggugaccug..	1	1	seq
..............uuaaggaaguuggugaccug..	92	0	seq
..............uuaaggGaguuggugaccug..	1	1	seq
..............uuaaggaaguCggugaccug..	1	1	seq
..............uuaaggaaguuggugaccCg..	2	1	seq
..............uuaaggaaguuggugaccuU..	2	1	seq
..............uuaaggaaguuggugaccuga...	24	0	seq
..............uuaaggaaguuggugaccugG...	1	1	seq
..............uuaaggaaguuggugaccugau..	9	0	seq
..............uuaaggaaguuggugaccugaC..	2	1	seq
..............uuaaggaaguuggugaccugaug...	112	0	seq
..............uCaaggaaguuggugaccugaug...	1	1	seq
..............uuaaggaaguugUugaccugaug...	2	1	seq
..............uuaaggaaguugguUaccugaug...	1	1	seq
..............uuaaggaaguuggugaccugaCg...	2	1	seq
..............uuaaggaaguuggugaccugGug...	2	1	seq
..............uuaaggaaguuggCgaccugaug...	1	1	seq
..............uuaaggaaguugAugaccugaug...	1	1	seq
..............uuaaggaaguuggugaccugauga..	4	0	seq
..............uuGaggaaguuggugaccugauga..	1	1	seq
...............uaaggaagGuggugacc..	1	1	seq
...............uaaggaaguuggugacc..	24	0	seq
...............uaaggaaguuggugaccC...	7	1	seq
...............uaaggaaguuggugaccG...	2	1	seq
...............uaaggaaguuggugaccA...	1	1	seq
...............uaaggaaguuggugaccu...	14	0	seq
...............uaaggaaguuggugaccug..	75	0	seq
...............Aaaggaaguuggugaccug..	1	1	seq
...............uaaggaaguuggugaccCg..	1	1	seq
...............Caaggaaguuggugaccug..	1	1	seq
...............uaaggGaguuggugaccug..	1	1	seq
...............uaGggaaguuggugaccuga...	1	1	seq
...............uaaggaaguuggugaccuga...	25	0	seq
...............Caaggaaguuggugaccuga...	2	1	seq
...............uaaggaaguuCgugaccuga...	1	1	seq
...............uaaggaaguuggugaccugaA..	1	1	seq
...............uaaggaaguuggugaccugau..	8	0	seq
...............Gaaggaaguuggugaccugaug...	1	1	seq
...............uaaggaaguuggugaccugGug...	1	1	seq
...............uaaggaaguuggugaccugauU...	1	1	seq
...............uaaggaaguuggugaccugaCg...	2	1	seq
...............uaaggaaguuggugGccugaug...	1	1	seq
...............uaaggaaguuggCgaccugaug...	1	1	seq
...............uaaggaaguuggugaccugaug...	148	0	seq
...............uaaggaaguuggugaccugauga..	7	0	seq
................aaggaaguuggugaccG...	2	1	seq
................aaggaaguuggugaccu...	20	0	seq
................aaggGaguuggugaccu...	1	1	seq
................aaggaaguuggugaccC...	3	1	seq
................aaggaaguuggugaccA...	2	1	seq
................aaggaaguugguAaccug..	1	1	seq
................aGggaaguuggugaccug..	1	1	seq
................aaggaaguuggugaccug..	109	0	seq
................Gaggaaguuggugaccug..	1	1	seq
................aaggaaguuggugaccuC..	1	1	seq
................aaggaaguuggCgaccug..	1	1	seq

Mature **Star**

```
ccaguucuauaagguuaaggaaguuggugaccugaugauggggaaaccaacgacugaagccucuaugaauuguugucacaacuauaaauuucuucaggcagaaaaauu
................aaggaGguuggugaccug..........................................................................   1     1    seq
................aaggaaguuggugGccug..........................................................................   1     1    seq
................aaggaaguuggugaccGg..........................................................................   1     1    seq
................aaggaagCuggugaccug..........................................................................   1     1    seq
................aaggaaguuggugaccAg..........................................................................   2     1    seq
................aaggaaguuggugaccuU..........................................................................   1     1    seq
................aaggGaguuggugaccug..........................................................................   1     1    seq
................aaggGaguuggugaccuga.........................................................................   1     1    seq
................aaggaaguuggugaccuga.........................................................................   30    0    seq
................aaggaaguuggugaccugau........................................................................   6     0    seq
................aaggaGguuggugaccugaug.......................................................................   1     1    seq
................aaggaaguuggugaccugGug.......................................................................   2     1    seq
................aaggaaguGggugaccugaug.......................................................................   1     1    seq
................aaggaaguuggugaccugaug.......................................................................   70    0    seq
................aaggaaguuggugaccugauga......................................................................   6     0    seq
.................aggaaguuggugaccug..........................................................................   287   0    seq
.................aggaGguuggugaccug..........................................................................   4     1    seq
.................aggaaguuggugaccuC..........................................................................   1     1    seq
.................aggaaguuggugGccug..........................................................................   1     1    seq
.................aggaaguugguAaccug..........................................................................   1     1    seq
.................aggaaguuggugaccuU..........................................................................   6     1    seq
.................aggaaguuggugaccGg..........................................................................   2     1    seq
.................aggaaguuggugaccAg..........................................................................   1     1    seq
.................aggaaguuggAgaccug..........................................................................   1     1    seq
.................aggaaguuggGgaccug..........................................................................   1     1    seq
.................aggGaguuggugaccug..........................................................................   2     1    seq
.................aggaaguCggugaccug..........................................................................   1     1    seq
.................aggaaguuggugacAug..........................................................................   1     1    seq
.................aggaaguuggugaccCg..........................................................................   2     1    seq
.................Gggaaguuggugaccug..........................................................................   2     1    seq
.................aggaagCuggugaccug..........................................................................   1     1    seq
.................aggaaguuggCgaccug..........................................................................   1     1    seq
.................Nggaaguuggugaccug..........................................................................   1     1    seq
.................aggaaguuggugaccuga.........................................................................   111   0    seq
.................aggaaguuggugaccugG.........................................................................   2     1    seq
.................aggaaguAggugaccuga.........................................................................   1     1    seq
.................aggaGguuggugaccuga.........................................................................   3     1    seq
.................aggaaguuggugaccugaC........................................................................   3     1    seq
.................aggaaguuggugaccugaG........................................................................   2     1    seq
.................aggaaguuggugaccugau........................................................................   25    0    seq
.................aggaaguuggugaccugCug.......................................................................   1     1    seq
.................aggaaguuggugaccCgaug.......................................................................   1     1    seq
.................aggGaguuggugaccugaug.......................................................................   1     1    seq
.................Gggaaguuggugaccugaug.......................................................................   1     1    seq
.................aggaaguuggugGccugaug.......................................................................   1     1    seq
.................agAaaguuggugaccugaug.......................................................................   1     1    seq
.................aggaGguuggugaccugaug.......................................................................   1     1    seq
.................aggaaguuggugaccugaGg.......................................................................   1     1    seq
.................aggaaguuggugaccugaCg.......................................................................   4     1    seq
.................aggaaguuggugaccugaug.......................................................................   195   0    seq
.................aggaaguuggugaccugaugU......................................................................   1     1    seq
.................aggaaguuggugGccugauga......................................................................   1     1    seq
.................aggaaguuggugaccugaugG......................................................................   1     1    seq
.................aggaaguuggugaccugauga......................................................................   16    0    seq
.................aggaaguuggugaccugaugauggg..................................................................   1     0    seq
..................ggaaguuggugaccuga.........................................................................   51    0    seq
..................ggaaguuggugaccugU.........................................................................   1     1    seq
..................ggaaguuggugaccugG.........................................................................   2     1    seq
..................ggaaguuggugaccuUa.........................................................................   1     1    seq
..................ggaaguuggugaccugau........................................................................   26    0    seq
..................ggaaguuggugaccugaC........................................................................   1     1    seq
..................ggaaguuggugaccugaug.......................................................................   116   0    seq
..................ggaaguuggugaccugaGg.......................................................................   2     1    seq
..................ggaaguuggugGccugaug.......................................................................   1     1    seq
..................ggGaguuggugaccugaug.......................................................................   1     1    seq
..................ggaaguugAugaccugaug.......................................................................   1     1    seq
..................ggaaguuggugaccugaCg.......................................................................   1     1    seq
..................ggaaguuggugaccCgaug.......................................................................   2     1    seq
..................ggaaguuggugaccugauga......................................................................   17    0    seq
..................ggaaguuggugaccugaugU......................................................................   1     1    seq
```

Mature **Star**

```
ccaguucuauaagguuaaggaaguuggugaccugaugauggggaaaccaacgacugaagccucuaugaauuguugucacaacuauaaauuucuucaggcagaaaaauu
..................ggaaguuggugaccGgauga......................................................................	1	1	seq
...................gaaguuggugaccugau........................................................................	13	0	seq
...................gaaguuggugaccugaC........................................................................	3	1	seq
...................gUaguuggugaccugaug.......................................................................	1	1	seq
...................gaagCuggugaccugaug.......................................................................	1	1	seq
...................gaaguuggugaccugaug.......................................................................	63	0	seq
...................gaaguuggugaccugaCg.......................................................................	2	1	seq
...................gaaguuggCgaccugaug.......................................................................	1	1	seq
...................gaaguuggugaccugGug.......................................................................	1	1	seq
...................Naaguuggugaccugaug.......................................................................	1	1	seq
...................gaaguuggugaccugauga......................................................................	5	0	seq
....................aaguuggugaccugGug.......................................................................	1	1	seq
....................aaguuggugaccugaug.......................................................................	42	0	seq
....................aaguuggugaccugauga......................................................................	5	0	seq
.....................aguuggugaccugauga......................................................................	10	0	seq
.....................aguuggugaccugaGga......................................................................	1	1	seq
.....................aguuggugaccugaugU......................................................................	1	1	seq
```

Provisional ID	:	MA_17259_3646
Score total	:	64843.7
Score for star read(s)	:	3.9
Score for read counts	:	64836.1
Score for mfe	:	2.1
Score for randfold	:	1.6
Score for cons. seed	:	
Total read count	:	127185
Mature read count	:	102041
Loop read count	:	0
Star read count	:	25144

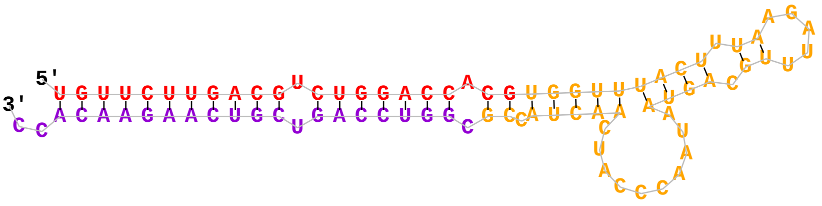

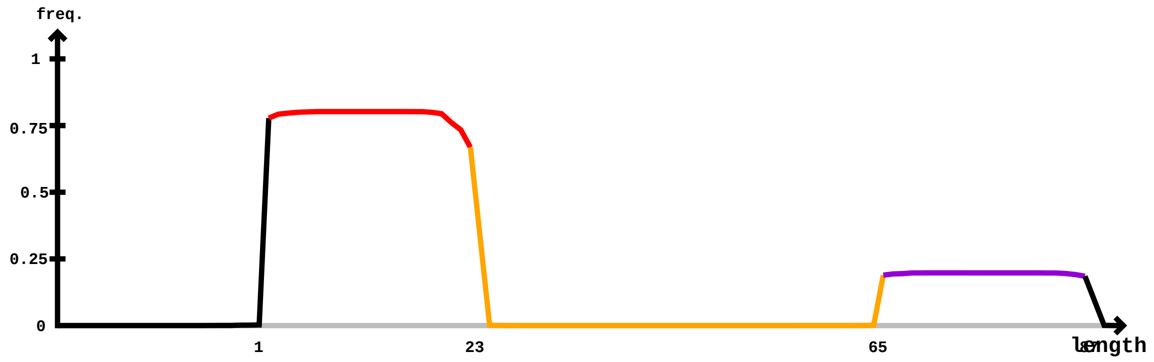


```
                          Mature                                                            Star
5'- agaggaagaggaagcgaagguguucuugacgucuggaccacgugguuuacuuuaagauuugcaguaauaacccaucaacuaccgcgguccagucgucaagaacaccuuu  -3'   obs
    agaggaagaggaagcgaagguguucuugacgucuggaccacgugguuuacuuuaagauuugcaguaauaacccaucaacuaccgcgguccagucgucaagaacaccuuu         exp
    ...............((((((((((((((.(((((((.(((((((((((.((.....)).))))..........))))).)).))))))).)))))))))))))))   reads   mm   sample
    Cgaggaagaggaagcga............................................................................................   1       1    seq
    ............agcgaagguguucuuga................................................................................   2       0    seq
    ............agcgaagguguucuCga................................................................................   1       1    seq
    ............agcgaagguguucuugaG...............................................................................   1       1    seq
    ............agcgaagguguucuugacguc............................................................................   1       0    seq
    ..............cgaagguguucuugacgucuA..........................................................................   2       1    seq
    ..............cgaGgguguucuugacgucug..........................................................................   1       1    seq
    ..............cgaagguguucuugacgucug..........................................................................   15      0    seq
    ..............cgaagguguucuugacgucuggGcc......................................................................   1       1    seq
    ...............gaagguguucuugacgucugg.........................................................................   1       0    seq
    ...............gaagguguucuugacgucugA.........................................................................   1       1    seq
    ...............gaagguguucuugacgucuggacc......................................................................   4       0    seq
    ...............gaagguguucuugacgucuggacA......................................................................   1       1    seq
    ...............gaagguguucuugacgucuggGcc......................................................................   1       1    seq
    ................aagguCuucuugacgucugga........................................................................   1       1    seq
    ................aagguguucuugacgucuggacc......................................................................   1       0    seq
    .................agguCuucuugacgucugg.........................................................................   2       1    seq
    .................agguCuucuugacgucugga........................................................................   7       1    seq
    .................agguCuucuugacgucuggac.......................................................................   1       1    seq
    .................aCguguucuugacgucuggacc......................................................................   122     1    seq
    .................agguguucuugacgucuggGcc......................................................................   13      1    seq
    ..................gguguucuugacgucuggacU......................................................................   4       1    seq
    ..................gguguucuugacAucuggacc......................................................................   1       1    seq
    ..................gguguucuugacgucuggacc......................................................................   14      0    seq
    ..................gguguucuugacgucuggGcc......................................................................   3       1    seq
    ..................Cguguucuugacgucuggacca.....................................................................   4       1    seq
    ..................gguguucuugacgucuggaccacg...................................................................   2       0    seq
    ...................Cuguucuugacgucugg.........................................................................   1       1    seq
    ...................guguucuugacgucuggGc.......................................................................   2       1    seq
    ...................guguucuugacgucuggGcc......................................................................   47      1    seq
    ...................guguucuugacgucuggacc......................................................................   5       0    seq
    ...................guguucuugacgucuggacU......................................................................   1       1    seq
    ...................Cuguucuugacgucuggacc......................................................................   1       1    seq
```

Mature / Star

agaggaagaggaagcgaagguguucuugacgucuggaccacgugguuuacuuuaagauuugcaguaauaacccaucaacuaccgcgguccagucgucaagaacaccuuu			
...................guAuucuugacgucuggacc..	1	1	seq
...................guAuucuugacgucuggacca...	1	1	seq
...................guguucuugacgucuggGcca...	10	1	seq
...................guguucuugacgucuggaccaU..	2	1	seq
...................guAuucuugacgucuggaccac..	1	1	seq
...................guguucuugacgucuggaccac..	1	0	seq
...................Cuguucuugacgucuggaccacg...	4	1	seq
...................guguucuugacgucuggGccacg...	3	1	seq
...................Auguucuugacgucuggaccacg...	1	1	seq
...................guUuucuugacgucuggaccacg...	1	1	seq
....................ugCucuugacgucugga..	1	1	seq
....................uguucuugacgucCgga..	1	1	seq
....................uguucuugacgucuAga..	1	1	seq
....................uguucuugacgucuggC..	1	1	seq
....................uguucuugacgucuggG..	28	1	seq
....................uguucuugacgCcugga..	1	1	seq
....................uguucuugacgAcugga..	1	1	seq
....................uAuucuugacgucugga..	60	1	seq
....................uCuucuugacgucugga..	1	1	seq
....................uguucuugacgucGgga..	1	1	seq
....................uguucuugGcgucugga..	2	1	seq
....................uguucuugacgucugga..	252	0	seq
....................uguucuuAacgucuggac...	2	1	seq
....................uguucuCgacgucuggac...	1	1	seq
....................uguucuugacgucuggGc...	163	1	seq
....................Cguucuugacgucuggac...	1	1	seq
....................uguucuugacgucuggac...	335	0	seq
....................uguucuugGcgucuggac...	2	1	seq
....................uguucuugacgucuggaA...	5	1	seq
....................uguucCugacgucuggac...	1	1	seq
....................uAuucuugacgucuggac...	27	1	seq
....................uguucuugacgucCggac...	1	1	seq
....................uguucuugacgucuggCc...	1	1	seq
....................uguucuugacgucuAgac...	1	1	seq
....................uguucuugacgucuggaU...	34	1	seq
....................uguucuugacgucuggacA..	123	1	seq
....................uguucuugacgucCggacc..	3	1	seq
....................uguuGuugacgucuggacc..	2	1	seq
....................uAuucuugacgucuggacc..	251	1	seq
....................uguucuGgacgucuggacc..	1	1	seq
....................uguucuugCcgucuggacc..	1	1	seq
....................uguucuugacgucuggacU..	429	1	seq
....................Gguucuugacgucuggacc..	7	1	seq
....................uguucuuAacgucuggacc..	1	1	seq
....................uguCcuugacgucuggacc..	6	1	seq
....................uguuAuugacgucuggacc..	1	1	seq
....................ugGucuugacgucuggacc..	2	1	seq
....................uguucuugaUgucuggacc..	1	1	seq
....................uguucuugacguGuggacc..	1	1	seq
....................uguucuugacgucuggCcc..	1	1	seq
....................uguucuugGcgucuggacc..	9	1	seq
....................uguucuugacgucuggGcc..	228	1	seq
....................uguAcuugacgucuggacc..	1	1	seq
....................uguucuugacgucugAacc..	1	1	seq
....................uguucCugacgucuggacc..	4	1	seq
....................uguuUuugacgucuggacc..	2	1	seq
....................uguucuugacAucuggacc..	8	1	seq
....................uguucuugacgucAggacc..	3	1	seq
....................uguucuugacgCcuggacc..	4	1	seq
....................uguucuugacgucGggacc..	2	1	seq
....................uguucuugacgucuggaAc..	2	1	seq
....................ugAucuugacgucuggacc..	1	1	seq
....................uguucuCgacgucuggacc..	8	1	seq
....................Nguucuugacgucuggacc..	2	1	seq
....................uguucuugacgucuggacG..	12	1	seq
....................uguucAugacgucuggacc..	1	1	seq
....................uguucuugacgucuggaUc..	7	1	seq
....................uguGcuugacgucuggacc..	1	1	seq
....................ugCucuugacgucuggacc..	8	1	seq
....................uguucuugacguUuggacc..	1	1	seq

```
                    Mature                                                          Star
agaggaagaggaagcgaagguguucuugacgucuggaccacgugguuuuacuuuaagauuugcaguaauaacccaucaacuaccgcgguccagucgucaagaacaccuuu
....................uguucuugacCucuggacc.......................................................................  1     1  seq
....................Aguucuugacgucuggacc.......................................................................  3     1  seq
....................Cguucuugacgucuggacc.......................................................................  7     1  seq
....................uguucuugacgucuggacc.......................................................................  2213  0  seq
....................uguucuugacgucugCacc.......................................................................  6     1  seq
....................uguucuugacgucuggGcca......................................................................  256   1  seq
....................uguucuugaUgucuggacca......................................................................  3     1  seq
....................uguuUuugacgucuggacca......................................................................  1     1  seq
....................uguucCugacgucuggacca......................................................................  8     1  seq
....................uguCcuugacgucuggacca......................................................................  5     1  seq
....................uguucuGgacgucuggacca......................................................................  1     1  seq
....................uguucuugacgucuggacca......................................................................  1648  0  seq
....................uguucuugCcgucuggacca......................................................................  1     1  seq
....................Cguucuugacgucuggacca......................................................................  3     1  seq
....................ugCucuugacgucuggacca......................................................................  4     1  seq
....................uguucuugacgucCggacca......................................................................  6     1  seq
....................uguucuCgacgucuggacca......................................................................  3     1  seq
....................uguucuugacgucugCacca......................................................................  4     1  seq
....................uguucuugacgucuggacAa......................................................................  3     1  seq
....................uguucuugacgucuggaccC......................................................................  26    1  seq
....................uguucuugUcgucuggacca......................................................................  2     1  seq
....................Aguucuugacgucuggacca......................................................................  1     1  seq
....................uguuAuugacgucuggacca......................................................................  1     1  seq
....................uguucuugacgucuAgacca......................................................................  2     1  seq
....................uguucuugacgucuggUcca......................................................................  3     1  seq
....................uguucuugaAgucuggacca......................................................................  1     1  seq
....................uguucuugacgucAggacca......................................................................  1     1  seq
....................uguucuugGcgucuggacca......................................................................  7     1  seq
....................uguucuugacgucuggacUa......................................................................  3     1  seq
....................uguucuugacgucuggaUca......................................................................  2     1  seq
....................uAuucuugacgucuggacca......................................................................  579   1  seq
....................uguucuugacgCcuggacca......................................................................  5     1  seq
....................uguucuugacUucuggacca......................................................................  1     1  seq
....................uguucuugacgucuggaccU......................................................................  43    1  seq
....................uguucuugacgGcuggacca......................................................................  1     1  seq
....................uguucuugacgucugAacca......................................................................  3     1  seq
....................uguucuugacAucuggacca......................................................................  1     1  seq
....................uguucuugacgucuggCcca......................................................................  1     1  seq
....................uguucuugacgucuggacGa......................................................................  1     1  seq
....................uguucuugacgucuggaccG......................................................................  92    1  seq
....................uguucuugacguUuggacca......................................................................  1     1  seq
....................uguucuugaUgucuggaccac.....................................................................  8     1  seq
....................uAuucuugacgucuggaccac.....................................................................  849   1  seq
....................uguucuugacgucuggaccaG.....................................................................  56    1  seq
....................Aguucuugacgucuggaccac.....................................................................  7     1  seq
....................uguucuugacgucGggaccac.....................................................................  1     1  seq
....................uguucuugacgucuggaGcac.....................................................................  1     1  seq
....................uguucuugacgucuAgaccac.....................................................................  5     1  seq
....................uguucuugacCucuggaccac.....................................................................  1     1  seq
....................ugAucuugacgucuggaccac.....................................................................  2     1  seq
....................Cguucuugacgucuggaccac.....................................................................  17    1  seq
....................uguucuugacguUuggaccac.....................................................................  6     1  seq
....................uguucuugUcgucuggaccac.....................................................................  2     1  seq
....................uguucuugacgucuggacGac.....................................................................  2     1  seq
....................uguucuugacgucugCaccac.....................................................................  3     1  seq
....................uguucuugacgucuggCccac.....................................................................  1     1  seq
....................uguucuugacAucuggaccac.....................................................................  10    1  seq
....................Nguucuugacgucuggaccac.....................................................................  5     1  seq
....................uguucuugacgucuggaccaA.....................................................................  57    1  seq
....................uguucuugacgucuggacAac.....................................................................  2     1  seq
....................uguucuugacgucuggaccCc.....................................................................  3     1  seq
....................uguCcuugacgucuggaccac.....................................................................  18    1  seq
....................uguuUuugacgucuggaccac.....................................................................  3     1  seq
....................uguucuAgacgucuggaccac.....................................................................  3     1  seq
....................uguucGugacgucuggaccac.....................................................................  1     1  seq
....................uguucCugacgucuggaccac.....................................................................  12    1  seq
....................uguucAugacgucuggaccac.....................................................................  2     1  seq
....................uguucuugacgAcuggaccac.....................................................................  1     1  seq
....................uguucuugacgucAggaccac.....................................................................  3     1  seq
....................uguucuugacgGcuggaccac.....................................................................  1     1  seq
```

```
                    Mature                                                           Star
agaggaagaggaagcgaagguguucuugacgucuggaccacgugguuuacuuuaagauuugcaguaauaacccaucaacuaccgcgguccagucgucaagaacaccuuu
....................ugGucuugacgucuggaccac....................................................................    4       1    seq
....................uguucuugGcgucuggaccac....................................................................    20      1    seq
....................uguucuCgacgucuggaccac....................................................................    18      1    seq
....................uguucuugacgucuggaUcac....................................................................    11      1    seq
....................uguucuugacguGuggaccac....................................................................    2       1    seq
....................uguucuugacgucugAaccac....................................................................    6       1    seq
....................uUuucuugacgucuggaccac....................................................................    1       1    seq
....................ugCucuugacgucuggaccac....................................................................    13      1    seq
....................uguucuugacgucuggGccac....................................................................    222     1    seq
....................uguucuuAacgucuggaccac....................................................................    3       1    seq
....................uguAcuugacgucuggaccac....................................................................    2       1    seq
....................uguucuugacguAuggaccac....................................................................    1       1    seq
....................uCuucuugacgucuggaccac....................................................................    1       1    seq
....................uguucuugacgucuggaccUc....................................................................    6       1    seq
....................uguucuugacgucuggaccGc....................................................................    35      1    seq
....................uguucuugacgucuggaccaU....................................................................    699     1    seq
....................uguucuugacgucuggacUac....................................................................    10      1    seq
....................uguuGuugacgucuggaccac....................................................................    2       1    seq
....................Gguucuugacgucuggaccac....................................................................    8       1    seq
....................uguucuugacgucuggaccac....................................................................    5927    0    seq
....................uguucuugaGgucuggaccac....................................................................    1       1    seq
....................uguucuugacgCcuggaccac....................................................................    16      1    seq
....................uguucuugacgucCggaccac....................................................................    12      1    seq
....................Gguucuugacgucuggaccacg...................................................................    83      1    seq
....................uguucuugacgucuAgaccacg...................................................................    39      1    seq
....................uguucuugacgucuggaccacA...................................................................    4701    1    seq
....................uUuucuugacgucuggaccacg...................................................................    4       1    seq
....................uguucuugaGgucuggaccacg...................................................................    6       1    seq
....................uguucuugacgucuCgaccacg...................................................................    5       1    seq
....................uguucuugaUgucuggaccacg...................................................................    45      1    seq
....................uguucuugacgucuggacUacg...................................................................    55      1    seq
....................uguucuugacgGcuggaccacg...................................................................    6       1    seq
....................uguucuugacgucugUaccacg...................................................................    11      1    seq
....................uguucuugacgucuggaUcacg...................................................................    36      1    seq
....................uguucuugacgucuggacGacg...................................................................    17      1    seq
....................ugCucuugacgucuggaccacg...................................................................    119     1    seq
....................uguucuugacgucuggaccacg...................................................................    50534   0    seq
....................uguAcuugacgucuggaccacg...................................................................    40      1    seq
....................uguucuugacgucGggaccacg...................................................................    13      1    seq
....................uguucuugCcgucuggaccacg...................................................................    7       1    seq
....................Nguucuugacgucuggaccacg...................................................................    58      1    seq
....................uguucuugaAgucuggaccacg...................................................................    3       1    seq
....................Aguucuugacgucuggaccacg...................................................................    56      1    seq
....................uguucuugacgucuggaccCcg...................................................................    17      1    seq
....................ugAucuugacgucuggaccacg...................................................................    16      1    seq
....................uguucuugacgucAggaccacg...................................................................    41      1    seq
....................uguucuugacguAuggaccacg...................................................................    7       1    seq
....................uguCcuugacgucuggaccacg...................................................................    130     1    seq
....................uguucuAgacgucuggaccacg...................................................................    11      1    seq
....................uguucuugacgucuggaccaUg...................................................................    258     1    seq
....................uguucuugacCucuggaccacg...................................................................    9       1    seq
....................ugGucuugacgucuggaccacg...................................................................    21      1    seq
....................uguuUuugacgucuggaccacg...................................................................    35      1    seq
....................uguucuugacgucuggGccacg...................................................................    16429   1    seq
....................uguucuugacUucuggaccacg...................................................................    7       1    seq
....................uguucuugacAucuggaccacg...................................................................    65      1    seq
....................uguucuugacgucCggaccacg...................................................................    123     1    seq
....................uguucGugacgucuggaccacg...................................................................    7       1    seq
....................uguucuugacgucuUgaccacg...................................................................    1       1    seq
....................uguucuGgacgucuggaccacg...................................................................    5       1    seq
....................uguucuugGcgucuggaccacg...................................................................    245     1    seq
....................uguucuugacgucuggUccacg...................................................................    14      1    seq
....................uguucuugacgAcuggaccacg...................................................................    18      1    seq
....................uguucuugacgucuggaccaNg...................................................................    2       1    seq
....................uguuAuugacgucuggaccacg...................................................................    4       1    seq
....................uguucuugacgucuggaAcacg...................................................................    7       1    seq
....................Cguucuugacgucuggaccacg...................................................................    132     1    seq
....................uguucCugacgucuggaccacg...................................................................    116     1    seq
....................uguucuugacgCcuggaccacg...................................................................    144     1    seq
....................uguucuugacgucuggacAacg...................................................................    20      1    seq
```

Mature **Star**

```
agaggaagaggaagcgaagguguucuugacgucuggaccacgugguuuacuuuaagauuugcaguaauaacccaucaacuaccgcgguccagucgucaagaacaccuuu
....................uguucuugacgucugAaccacg................................................................... 35 1 seq
....................uguucuugacgucuggCccacg................................................................... 5 1 seq
....................uguGcuugacgucuggaccacg................................................................... 15 1 seq
....................uguucuCgacgucuggaccacg................................................................... 139 1 seq
....................uguucuugUcgucuggaccacg................................................................... 16 1 seq
....................uguucuugacgucuggaccaGg................................................................... 23 1 seq
....................uguucuugacgucugCaccacg................................................................... 4 1 seq
....................uguucuugacgucuggaccGcg................................................................... 259 1 seq
....................uAuucuugacgucuggaccacg................................................................... 7158 1 seq
....................uguucuugacguGuggaccacg................................................................... 13 1 seq
....................uguucAugacgucuggaccacg................................................................... 16 1 seq
....................uguucuugacgucuggaccacU................................................................... 882 1 seq
....................uguuGuugacgucuggaccacg................................................................... 6 1 seq
....................uguucuugacgucuggaccaAg................................................................... 66 1 seq
....................uguucuugacgucuggaccacC................................................................... 425 1 seq
....................uguucuugacgucuggaccUcg................................................................... 36 1 seq
....................uguucuuUacgucuggaccacg................................................................... 2 1 seq
....................uguucuuAacgucuggaccacg................................................................... 20 1 seq
....................uNuucuugacgucuggaccacg................................................................... 1 1 seq
....................uguucuugacguUuggaccacg................................................................... 22 1 seq
....................uguucuugacgucuggaGcacg................................................................... 8 1 seq
....................uguucuugacgCcuggaccacgu.................................................................. 2 1 seq
....................uguGcuugacgucuggaccacgu.................................................................. 1 1 seq
....................uguucuugacgucuggaccacgC.................................................................. 96 1 seq
....................uguucuugacgucuggaccacCu.................................................................. 3 1 seq
....................uguucuugacgucuggacUacgu.................................................................. 1 1 seq
....................uguucCugacgucuggaccacgu.................................................................. 2 1 seq
....................uguucuugacgucuggGccacgu.................................................................. 22 1 seq
....................uguucuugacgucuggaccacUu.................................................................. 19 1 seq
....................uguCcuugacgucuggaccacgu.................................................................. 1 1 seq
....................uguucuugacgucuggaccacgu.................................................................. 398 0 seq
....................uguucuugacgucuggaccGcgu.................................................................. 3 1 seq
....................uAuucuugacgucuggaccacgu.................................................................. 94 1 seq
....................uguucuugacgucCggaccacgu.................................................................. 1 1 seq
....................uguucuugGcgucuggaccacgu.................................................................. 1 1 seq
....................Cguucuugacgucuggaccacgu.................................................................. 1 1 seq
....................uguucuugacgucuggaccacgA.................................................................. 44 1 seq
....................ugGucuugacgucuggaccacgu.................................................................. 1 1 seq
....................uguucuCgacgucuggaccacgu.................................................................. 1 1 seq
....................uguucuugacgucuggaccUcgu.................................................................. 1 1 seq
....................Gguucuugacgucuggaccacgu.................................................................. 2 1 seq
....................uguucuugacgucuggaccacgG.................................................................. 23 1 seq
....................uguucuuUacgucuggaccacgu.................................................................. 1 1 seq
....................ugCucuugacgucuggaccacgu.................................................................. 1 1 seq
....................uguucuugacgucuggaccacguU................................................................. 24 1 seq
....................uguucuugacgucuggaccacgAg................................................................. 13 1 seq
....................uguucuugacgucuggaccacgug................................................................. 16 0 seq
....................uguucuugacgucuggaccacguC................................................................. 5 1 seq
....................uguucuugacgucuggGccacgug................................................................. 4 1 seq
....................uguucuugacgucuggaccGcgug................................................................. 1 1 seq
....................uguucuugacgucuggaccacgGg................................................................. 9 1 seq
....................uguucuugacgucuggaccacgCg................................................................. 1 1 seq
....................uguucuugGcgucuggaccacgug................................................................. 1 1 seq
....................uguucuugacgucuggaccacguA................................................................. 6 1 seq
....................uAuucuugacgucuggaccacgug................................................................. 3 1 seq
....................uguucuugacgucuggaccacgGgg................................................................ 1 1 seq
....................uguucuugacgucuggaccacgCgg................................................................ 2 1 seq
....................uguucuugacgucuggaccacgugU................................................................ 1 1 seq
....................uguucuugacgucuggaccacguCg................................................................ 3 1 seq
....................uguucuugacgucuggaccacguUg................................................................ 4 1 seq
....................uguucuugacgucuggaccacgugA................................................................ 1 1 seq
....................uguucuugacgucuggaccacgAgg................................................................ 2 1 seq
....................uguucuugacgucuggaccacgCggu............................................................... 1 1 seq
....................uguucuugacgucuggaccacgugUu............................................................... 1 1 seq
....................uguucuugacgucuggaccacgGggu............................................................... 1 1 seq
....................uguucuugacgucuggaccacguggAu.............................................................. 1 1 seq
....................uguucuugacgucuggaccacgugguGu............................................................. 1 1 seq
.....................guucuugacgucuggaU....................................................................... 3 1 seq
.....................guucuugacgucuggGc....................................................................... 10 1 seq
.....................guucuCgacgucuggac....................................................................... 1 1 seq
```

Mature   Star

```
agaggaagaggaagcgaagguguucuugacgucuggaccacgugguuuacuuuaagauuugcaguaauaacccaucaacuaccgcgguccagucgucaagaacaccuuu
.....................guucuugacgucuggac.......................................................................	44	0	seq
.....................guucuugacgucuggacG......................................................................	1	1	seq
.....................guucuugacgucuggaAc......................................................................	1	1	seq
.....................Auucuugacgucuggacc......................................................................	13	1	seq
.....................gCucuugacgucuggacc......................................................................	1	1	seq
.....................guucuugacgucuggacU......................................................................	64	1	seq
.....................guGcuugacgucuggacc......................................................................	2	1	seq
.....................guucCugacgucuggacc......................................................................	1	1	seq
.....................guucuugacgucugCacc......................................................................	2	1	seq
.....................guucuugacgucugUacc......................................................................	1	1	seq
.....................guucuugacgucuggacc......................................................................	422	0	seq
.....................guucuCgacgucuggacc......................................................................	1	1	seq
.....................guucuugacgucCggacc......................................................................	1	1	seq
.....................guucuugGcgucuggacc......................................................................	1	1	seq
.....................guucuugacgucuggacA......................................................................	14	1	seq
.....................guCcuugacgucuggacc......................................................................	1	1	seq
.....................guucuugacgCcuggacc......................................................................	2	1	seq
.....................guucuugacgucuggGcc......................................................................	4	1	seq
.....................guucuugacgucAggacca.....................................................................	1	1	seq
.....................guucuugacgAcuggacca.....................................................................	1	1	seq
.....................guucuugacgucuggaccG.....................................................................	32	1	seq
.....................guucuugacgucuAgacca.....................................................................	1	1	seq
.....................guucuugacgucuggacca.....................................................................	607	0	seq
.....................guucuugacgucuggaccC.....................................................................	6	1	seq
.....................guuUuugacgucuggacca.....................................................................	1	1	seq
.....................guucuugaUgucuggacca.....................................................................	1	1	seq
.....................guucuugacAucuggacca.....................................................................	2	1	seq
.....................guucuugacgucuggaccU.....................................................................	12	1	seq
.....................gCucuugacgucuggacca.....................................................................	1	1	seq
.....................Uuucuugacgucuggacca.....................................................................	2	1	seq
.....................Auucuugacgucuggacca.....................................................................	8	1	seq
.....................guucuugacgucuggGcca.....................................................................	15	1	seq
.....................guucuugacgucugAacca.....................................................................	1	1	seq
.....................guucuugGcgucuggacca.....................................................................	1	1	seq
.....................guCcuugacgucuggacca.....................................................................	2	1	seq
.....................guucuCgacgucuggacca.....................................................................	4	1	seq
.....................guucCugacgucuggacca.....................................................................	1	1	seq
.....................guucuugacgucCggacca.....................................................................	2	1	seq
.....................guucuugacgCcuggacca.....................................................................	2	1	seq
.....................guucuugacgGcuggacca.....................................................................	1	1	seq
.....................guucuugacgucuggacUa.....................................................................	1	1	seq
.....................guucuugacgucuggaccGc....................................................................	1	1	seq
.....................guucuugacgucuggaUcac....................................................................	1	1	seq
.....................gCucuugacgucuggaccac....................................................................	1	1	seq
.....................guucuugacgucuggaccac....................................................................	101	0	seq
.....................guucuugacgucuggUccac....................................................................	1	1	seq
.....................guucuugacgucuggaccaA....................................................................	1	1	seq
.....................guucuugacgucuggaccaU....................................................................	16	1	seq
.....................guucuugacgucAggaccac....................................................................	1	1	seq
.....................guucuugacgucuggaccaG....................................................................	1	1	seq
.....................Auucuugacgucuggaccac....................................................................	14	1	seq
.....................guucuugGcgucuggaccacg...................................................................	1	1	seq
.....................guucuugacgucuAgaccacg...................................................................	1	1	seq
.....................guucuugacgCcuggaccacg...................................................................	1	1	seq
.....................Cuucuugacgucuggaccacg...................................................................	1	1	seq
.....................guucuugacgucuggaccacC...................................................................	4	1	seq
.....................guucuugaUgucuggaccacg...................................................................	1	1	seq
.....................guucCugacgucuggaccacg...................................................................	2	1	seq
.....................guucuugacgucuggaccacA...................................................................	14	1	seq
.....................guucuugacgucuggaccacg...................................................................	163	0	seq
.....................guucuugacgucuggaccGcg...................................................................	2	1	seq
.....................guCcuugacgucuggaccacg...................................................................	1	1	seq
.....................guucuugacgucuggaccacU...................................................................	2	1	seq
.....................guucuugacgucuggaccaUg...................................................................	1	1	seq
.....................Auucuugacgucuggaccacg...................................................................	190	1	seq
.....................guucuugacgucuggGccacg...................................................................	8	1	seq
.....................guucuugacgAcuggaccacgu..................................................................	1	1	seq
.....................guucuugGcgucuggaccacgu..................................................................	2	1	seq
.....................guucuugacgucuggGccacgu..................................................................	4	1	seq
.....................Auucuugacgucuggaccacgu..................................................................	4	1	seq
```

```
                      Mature                                                        Star
agaggaagaggaagcgaagguguucuugacgucuggaccacgugguuuacuuuaagauuugcaguaauaacccaucaacuaccgcgguccagucgucaagaacaccuuu
.....................guucuugacgucuggaccacgC..................................................................  7    1  seq
.....................guucuugacgucuggaccacgu..................................................................  36   0  seq
.....................guucuugacgucugAaccacgu..................................................................  1    1  seq
.....................guucuugacgCcuggaccacgu..................................................................  1    1  seq
.....................guucuugacgucuggaccacguC.................................................................  1    1  seq
......................uucuuAacgucuggacc......................................................................  1    1  seq
......................uuGuugacgucuggacc......................................................................  1    1  seq
......................uucuuUacgucuggacc......................................................................  1    1  seq
......................uucuugacgucuggGcc......................................................................  1    1  seq
......................uucuugacgucuggacU......................................................................  8    1  seq
......................uucuugacgucuggacc......................................................................  34   0  seq
......................uucuugacgucuggaccU.....................................................................  1    1  seq
......................uucuugacgucuggaccC.....................................................................  2    1  seq
......................uucuugacgucuggGcca.....................................................................  6    1  seq
......................uucuugacgucuggacca.....................................................................  43   0  seq
......................uucuugacgucuggaccG.....................................................................  1    1  seq
......................uucuCgacgucuggaccac....................................................................  1    1  seq
......................uucuugacgucuggaccac....................................................................  36   0  seq
......................uucuugacgCcuggaccac....................................................................  1    1  seq
......................uucuugacgucuggaccaA....................................................................  1    1  seq
......................uucuugacgucuggaccaU....................................................................  1    1  seq
......................uucuugacgucuggGccac....................................................................  3    1  seq
......................uucuugacgucuggaccacU...................................................................  3    1  seq
......................uucuugacgucuggaccacA...................................................................  4    1  seq
......................uucuugacgucuggaccGcg...................................................................  1    1  seq
......................uucuugGcgucuggaccacg...................................................................  1    1  seq
......................Nucuugacgucuggaccacg...................................................................  2    1  seq
......................uucuugacgucuggGccacg...................................................................  7    1  seq
......................uucuugacgucuggaccacg...................................................................  70   0  seq
......................uucuugacgucuggaccacgG..................................................................  8    1  seq
......................uucuugacgucuggaccacUu..................................................................  1    1  seq
......................uucuugacgucuggaccacgu..................................................................  7    0  seq
......................uucuugacgucuggaccacgA..................................................................  253  1  seq
......................uucuugacgucuggaccacgug.................................................................  3    0  seq
......................uucuugacgucuggaccacguA.................................................................  1    1  seq
......................uucuugacgucuggGccacgug.................................................................  1    1  seq
.......................ucuugacgucuggaccG.....................................................................  4    1  seq
.......................ucuugacgucuggacca.....................................................................  50   0  seq
.......................ucuugacgCcuggacca.....................................................................  1    1  seq
.......................ucuugacgucuggGcca.....................................................................  6    1  seq
.......................ucuugacgucuggaccaG....................................................................  1    1  seq
.......................Acuugacgucuggaccac....................................................................  1    1  seq
.......................ucuugacgucuggGccac....................................................................  3    1  seq
.......................ucuugacgucuggaccaU....................................................................  1    1  seq
.......................ucuugacgucuggaccaA....................................................................  1    1  seq
.......................ucuugacgucuggaccac....................................................................  38   0  seq
.......................ucuugacgucuggaccacg...................................................................  133  0  seq
.......................ucuugacAucuggaccacg...................................................................  1    1  seq
.......................ucuugacgucuggacUacg...................................................................  1    1  seq
.......................ucuugacgucuggaccacC...................................................................  2    1  seq
.......................ucuugacgucuggaccGcg...................................................................  1    1  seq
.......................ucuugacgucuggaccacA...................................................................  13   1  seq
.......................ucuugacgucuggaccacU...................................................................  3    1  seq
.......................ucCugacgucuggaccacg...................................................................  2    1  seq
.......................ucuugGcgucuggaccacg...................................................................  2    1  seq
.......................ucuugacgucuggGccacg...................................................................  27   1  seq
.......................ucuugacgCcuggaccacg...................................................................  2    1  seq
.......................ucuugacgGcuggaccacg...................................................................  1    1  seq
.......................ucuugacgucuggaccacgu..................................................................  2    0  seq
.......................ucuugacgucuggaccacgG..................................................................  3    1  seq
.......................ucuugacgucuggaccacgA..................................................................  63   1  seq
.......................ucuugacgucuggaccacgC..................................................................  1    1  seq
.......................ucuugacgucuggaccacgAg.................................................................  12   1  seq
.......................ucuugacgucuggaccGcgug.................................................................  1    1  seq
.......................ucuugacgucuggaccacguUg................................................................  1    1  seq
.......................ucuugacgucuggaccacgugC................................................................  5    1  seq
.......................ucuugacgucuggaccacgAgg................................................................  3    1  seq
.......................ucuugacgucuggaccacgugU................................................................  7    1  seq
........................cuugGcgucuggaccac....................................................................  1    1  seq
........................cuugacgucuggaccaU....................................................................  4    1  seq
```

```
                    Mature                                                           Star
agaggaagaggaagcgaagguguucuugacgucuggaccacgugguuuacuuuaagauuugcaguaauaacccaucaacuaccgcgguccagucgucaagaacaccuuu
........................cuugacgucuggaccac....................................................................	24	0	seq
........................cuugacgucuggaccacA...................................................................	14	1	seq
........................cuugacgucuggaccaUg...................................................................	1	1	seq
........................cuugacgCcuggaccacg...................................................................	1	1	seq
........................cuugacgucuggaccacg...................................................................	101	0	seq
........................cuugacgucuggaccacC...................................................................	1	1	seq
........................cuugacgucuAgaccacg...................................................................	1	1	seq
........................cuugacgucuggacUacg...................................................................	1	1	seq
........................cuugacgucuggGccacg...................................................................	26	1	seq
........................cuugacgucuggaccacgu..................................................................	1	0	seq
........................cuugacgucuggaccacgugUu...............................................................	6	1	seq
.........................uugCcgucuggaccacg...................................................................	1	1	seq
.........................uugacgucuggGccacg...................................................................	12	1	seq
.........................uugacgucuggaccacC...................................................................	1	1	seq
.........................uugaUgucuggaccacg...................................................................	1	1	seq
.........................uugacgucuggaccacU...................................................................	2	1	seq
.........................uCgacgucuggaccacg...................................................................	1	1	seq
.........................uugacgucuggaccacA...................................................................	12	1	seq
.........................uugacgucuggaccacg...................................................................	115	0	seq
.........................uugUcgucuggaccacg...................................................................	2	1	seq
.........................uugacgucuggaccacgA..................................................................	8	1	seq
.........................uugacgucuggaccacgC..................................................................	1	1	seq
.........................uugacgucuggaccacgu..................................................................	1	0	seq
.........................uugacgucuggaccacguU.................................................................	1	1	seq
.........................uugacgucuggaccacgugA................................................................	1	1	seq
.........................uugacgucuggaccacgugC................................................................	1	1	seq
.........................uugacgucuggaccacgugUuu..............................................................	1	1	seq
..........................ugacgucuggaccacgug.................................................................	1	0	seq
..........................ugacgucuggaccacgAg.................................................................	1	1	seq
..........................uAacgucuggaccacgugg................................................................	1	1	seq
........................................................................caucaacuaUcgcggucca..................	1	1	seq
.........................................................................aucaacuaUcgcgguccagu................	1	1	seq
..........................................................................ucaacuaUcgcgguccagu................	1	1	seq
...........................................................................caacuaUcgcgguccagu................	1	1	seq
............................................................................aacuaUcgcgguccagu................	1	1	seq
............................................................................aacuaUcgcgguccaguc...............	1	1	seq
............................................................................aacuaUcgcgguccagucg..............	1	1	seq
............................................................................aacuaUcgcgguccagucguc............	1	1	seq
.............................................................................acuaUcgcgguccagucgu.............	1	1	seq
.............................................................................acuaUcgcgguccagucguc............	2	1	seq
...............................................................................uaUcgcgguccagucguc............	2	1	seq
...............................................................................uaUcgcgguccagucgucaag.........	2	1	seq
...............................................................................uaUcgcgguccagucgucaagaac......	2	1	seq
................................................................................aUcgcgguccagucgucaaga........	5	1	seq
.................................................................................Ucgcgguccagucgucaaga........	1	1	seq
.................................................................................Ucgcgguccagucgucaagaa.......	1	1	seq
.................................................................................Ucgcgguccagucgucaagaac......	1	1	seq
..................................................................................cgcgguccagucgucUagaa.......	1	1	seq
..................................................................................cgcgguccagucgucUagaac......	14	1	seq
..................................................................................cgcgguccagucgucaagaacacU...	1	1	seq
..................................................................................cgcgguccagucgucaagaacacA...	1	1	seq
..................................................................................cgcgguccagucgucUagaacacc...	4	1	seq
..................................................................................cgcgguccagucgucaagaacaccu..	1	0	seq
...................................................................................gcgguccagucgucUaga........	1	1	seq
...................................................................................gcgguccagucgucaagaac......	1	0	seq
...................................................................................gcgguccagucgucUagaac......	6	1	seq
...................................................................................gcgguccagucgucUagaaca.....	14	1	seq
...................................................................................gcgguccagucgucaagaaca.....	5	0	seq
...................................................................................gcgguccagucgucUagaacac....	1	1	seq
...................................................................................gcgguccagucgucUagaacacc...	23	1	seq
...................................................................................gcgguccagucgucaagaacacc...	4	0	seq
...................................................................................gcgguccagucgucUagaacaccu..	1	1	seq
....................................................................................cgguccagucgucaaga........	2	0	seq
....................................................................................cgguccagucgucUaga........	20	1	seq
....................................................................................cgguccagucgucaagaa.......	1	0	seq
....................................................................................cgguccagucgucUagaa.......	10	1	seq
....................................................................................cgguccagCcgucaagaac......	1	1	seq
....................................................................................cgguccagucgucaagaGc......	1	1	seq
....................................................................................cgguccagucgucaagaac......	16	0	seq
....................................................................................cgguccagucgucUagaac......	76	1	seq
```

```
                     Mature                                                             Star
agaggaagaggaagcgaagguguucuugacgucuggaccacgugguuuuacuuuaagauuugcaguaauaacccaucaacuaccgcgguccagucgucaagaacaccuuu
.....................................................................................cgguccagucgucaagaaU......   2       1     seq
.....................................................................................cgguccagucgCcaagaac......   1       1     seq
.....................................................................................cgguccagucgucaaAaaca.....   1       1     seq
.....................................................................................cgguccagucgucUagaaca.....   97      1     seq
.....................................................................................cgguccagucgucaagaacU.....   1       1     seq
.....................................................................................cgguccagucgucaagaacG.....   2       1     seq
.....................................................................................cgguccagucgucaagaaca.....   36      0     seq
.....................................................................................cgguccagucgucaagaacaU....   9       1     seq
.....................................................................................cgguccCgucgucaagaacac....   1       1     seq
.....................................................................................Agguccagucgucaagaacac....   1       1     seq
.....................................................................................cgguccagucgucaagaacaA....   2       1     seq
.....................................................................................cggCccagucgucaagaacac....   2       1     seq
.....................................................................................cgguccagucgucaagaacac....   130     0     seq
.....................................................................................cgguccagucgucaagUacac....   1       1     seq
.....................................................................................cgguccaguUgucaagaacac....   2       1     seq
.....................................................................................cgguccagucgucaagaacCc....   1       1     seq
.....................................................................................cgguccagucgucUagaacac....   510     1     seq
.....................................................................................cgguccagucgucaagaacacA...   14      1     seq
.....................................................................................cggCccagucgucaagaacacc...   5       1     seq
.....................................................................................cgAuccagucgucaagaacacc...   1       1     seq
.....................................................................................cgguccagucgucaGgaacacc...   4       1     seq
.....................................................................................cggGccagucgucaagaacacc...   1       1     seq
.....................................................................................cgguccagucgCcaagaacacc...   2       1     seq
.....................................................................................cgguccGgucgucaagaacacc...   3       1     seq
.....................................................................................cgguccaguUgucaagaacacc...   1       1     seq
.....................................................................................cgguccagucgucGagaacacc...   5       1     seq
.....................................................................................cgguccagucgucUagaacacc...   22203   1     seq
.....................................................................................cgguccagucgucaagGacacc...   2       1     seq
.....................................................................................cgguccagucgucaagaacGcc...   1       1     seq
.....................................................................................cgguccUgucgucaagaacacc...   1       1     seq
.....................................................................................cgguccagucgucaaUaacacc...   1       1     seq
.....................................................................................cgguccagucgucaagaacacU...   67      1     seq
.....................................................................................cgguccagucgucaagaacaUc...   4       1     seq
.....................................................................................cgguccagucgucaagaGcacc...   2       1     seq
.....................................................................................cgguccagucgucaagaacUcc...   1       1     seq
.....................................................................................cgguccagucgucCagaacacc...   47      1     seq
.....................................................................................Agguccagucgucaagaacacc...   2       1     seq
.....................................................................................cgguccagucgucaagaacacc...   571     0     seq
.....................................................................................cgguccagucgucaagaacaccu..   8       0     seq
.....................................................................................cgguccagucgucaagaacaccG..   1       1     seq
.....................................................................................cgguccagucgucaagaacaccC..   1       1     seq
.....................................................................................cgguccagucgucUagaacaccu..   114     1     seq
.....................................................................................cgguccagucgucaagaacaccuuG   1       1     seq
......................................................................................gguccagucgucUagaa.......   1       1     seq
......................................................................................gguccagucgucaagaac......   4       0     seq
......................................................................................gguccagucgucUagaac......   89      1     seq
......................................................................................gguccagucgucUagaaca.....   246     1     seq
......................................................................................gguccagucgucaagaaca.....   1       0     seq
......................................................................................gguccagucgucUagaacac....   97      1     seq
......................................................................................gguccagucgucaagaacac....   2       0     seq
......................................................................................gguccagucgucCagaacacc...   1       1     seq
......................................................................................gguccagucgucaagaacacc...   2       0     seq
......................................................................................gguccagucgucUagaacacc...   109     1     seq
.......................................................................................guccagucgucUagaac......   41      1     seq
.......................................................................................guccagucgucUagaaca.....   73      1     seq
.......................................................................................guccagucgucaagaacU.....   1       1     seq
.......................................................................................guccagucgucUagaacac....   17      1     seq
.......................................................................................guccagucgucaagaacacc...   12      0     seq
.......................................................................................guccagucgucaagaacacU...   2       1     seq
.......................................................................................guccagucgucUagaacacc...   7       1     seq
.......................................................................................guccagucgucUagaacaccu..   5       1     seq
........................................................................................uccagucgucaagaacC.....   1       1     seq
........................................................................................uccagucgucUagaaca.....   7       1     seq
........................................................................................uccagucgucaagaaca.....   1       0     seq
........................................................................................Gccagucgucaagaaca.....   1       1     seq
........................................................................................uccagucgucUagaacac....   6       1     seq
........................................................................................uccagucgucaagaacac....   2       0     seq
........................................................................................uccagucgucUagaacacc...   36      1     seq
........................................................................................uccagucgucaagaacacU...   1       1     seq
........................................................................................uccagucgucaagaacacc...   4       0     seq
```

```
                    Mature                                                        Star
agaggaagaggaagcgaagguguucuugacgucuggaccacgugguuuacuuuaagauuugcaguaauaacccaucaacuaccgcgguccagucgucaagaacaccuuu
.......................................................................................uccagucgucaagaacaccu..    1     0    seq
.......................................................................................uccagucgucUagaacaccu..    178   1    seq
.......................................................................................uccagucgucaagaacaccuu.    8     0    seq
.......................................................................................uccagucgucUagaacaccuu.    77    1    seq
.......................................................................................uccagucgucUagaacaccuuu    7     1    seq
........................................................................................ccagucgucUagaacac....    4     1    seq
........................................................................................ccagucgucUagaacacc...    11    1    seq
.........................................................................................cagucgucUagaacacc...    21    1    seq
.........................................................................................cagucgucUagaacaccu..    1     1    seq
```

Provisional ID	:	MA_175789_9275
Score total	:	0.1
Score for star read(s)	:	-1.3
Score for read counts	:	0
Score for mfe	:	-0.1
Score for randfold	:	1.6
Score for cons. seed	:	
Total read count	:	21
Mature read count	:	21
Loop read count	:	0
Star read count	:	0

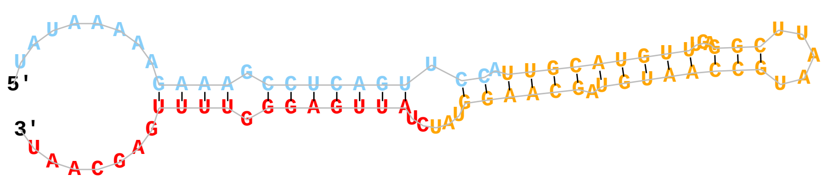

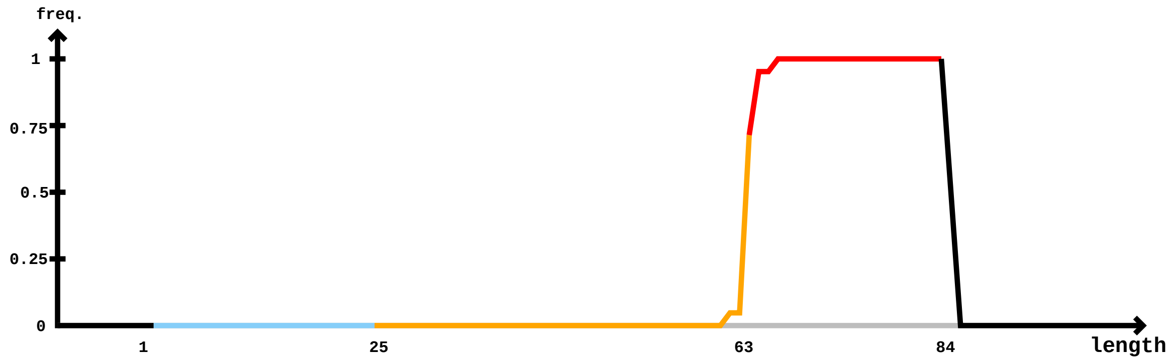

Star Mature

```
5'- acauagauuauaaaaagaaagccucaguuccauugcauguuugaggcuuaaugccaauguagcaagguaucuauugaggguuuugagcaauuuaugggaagagaggccaau   -3'    exp
    .((((((((.......((((.(((((((.((.(((((((((...(((.....)))))))).)))))).....))))))).))))....))))))))...............    reads      mm          sample
    ....................................................................aucuauugagggCuuugagcaau....................    1          1           seq
    ......................................................................cuauugaggguuuugagcaGu....................    1          1           seq
    ......................................................................cuauugaggguuuugagcaau....................    11         0           seq
    ......................................................................cuauugaggguuuugagcaaC....................    2          1           seq
    .......................................................................uauGgaggguuuugagcaauu...................    2          1           seq
    .......................................................................uauugaggguuuugagcaauu...................    2          0           seq
    .......................................................................uauugaggguuuugagcaaCu...................    1          1           seq
    .........................................................................uugaggguuuugagcaauC...................    1          1           seq
```

Provisional ID	:	MA_175803_22280
Score total	:	15287.4
Score for star read(s)	:	3.9
Score for read counts	:	15279.6
Score for mfe	:	2.4
Score for randfold	:	1.6
Score for cons. seed	:	
Total read count	:	29982
Mature read count	:	29832
Loop read count	:	0
Star read count	:	150

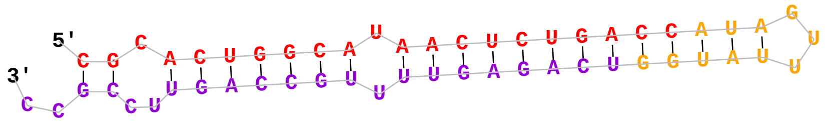

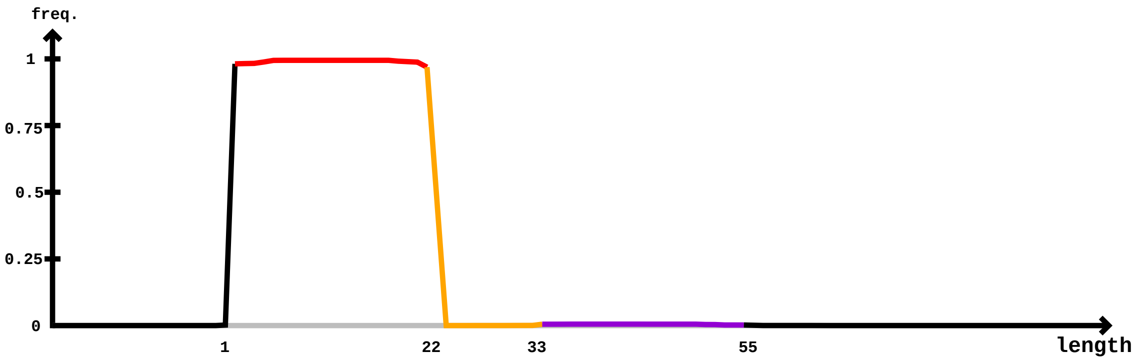

Mature **Star**

```
5'- ggcuugguaggauuaggcgcacuggcauaacucugaccauaguuuauggucagaguuuugccaguuccgcccauuccuauugugccuuuaaguaauuucagaaaugga  -3'   obs
    ggcuugguaggauuaggcgcacuggcauaacucugaccauaguuuauggucagaguuuugccaguuccgcccauuccuauugugccuuuaaguaauuucagaaaugga        exp
    (((.((((((((...((((.(((((((.((((((((((((((...)))))))))))))).)))))))..))))...)))))))).)))..........((((....))))   reads      mm      sample
    ...............ggcgGacuggcauaacucu..........................................................................   1          1       seq
    ................gcgGacuggcauaacucu..........................................................................   1          1       seq
    ................gcgGacuggcauaacucug.........................................................................   1          1       seq
    ................gcgGacuggcauaacucuga........................................................................   1          1       seq
    ................gcgGacuggcauaacucugac.......................................................................   52         1       seq
    .................cgAacuggcauaacucu..........................................................................   1          1       seq
    .................cgGacuggcauaacucu..........................................................................   92         1       seq
    .................cgGacuggcauaacucug.........................................................................   52         1       seq
    .................cgGacuggcauaacucuga........................................................................   53         1       seq
    .................cgGacuggcauaacucugac.......................................................................   508        1       seq
    .................cgAacuggcauaacucugac.......................................................................   1          1       seq
    .................cgcacuggcauaacucugacc......................................................................   3          0       seq
    .................cgGacuggcauaacucugacc......................................................................   28538      1       seq
    .................cgUacuggcauaacucugacc......................................................................   5          1       seq
    .................cgcacuggcauaacucugacU......................................................................   1          1       seq
    .................cgAacuggcauaacucugacc......................................................................   11         1       seq
    .................cgcacuggcauaacucugaccG.....................................................................   1          1       seq
    .................cgGacuggcauaacucugacca.....................................................................   116        1       seq
    ..................gGacuggcauaacucugacc......................................................................   28         1       seq
    ..................gGacuggcauaacucugacca.....................................................................   2          1       seq
    ...................Gacuggcauaacucuga........................................................................   1          1       seq
    ...................Gacuggcauaacucugacc......................................................................   16         1       seq
    ...................Gacuggcauaacucugacca.....................................................................   2          1       seq
    ....................acuggcauaacucugac.......................................................................   4          0       seq
    ....................acuggcauaacuUugacc......................................................................   1          1       seq
    ....................acuggcauaacucugacU......................................................................   12         1       seq
    ....................acuggcauGacucugacc......................................................................   2          1       seq
    ....................Ncuggcauaacucugacc......................................................................   1          1       seq
    ....................acuggcauaacucugacA......................................................................   3          1       seq
    ....................acuggcauaacucugacc......................................................................   66         0       seq
    ....................acuAgcauaacucugacc......................................................................   1          1       seq
    ....................acuggcauaacucugaccU.....................................................................   2          1       seq
    ....................acuggcauaacucugaccC.....................................................................   1          1       seq
```

```
                Mature                         Star
ggcuugguaggauuaggcgcacuggcauaacucugaccauaguuuauggucagaguuuugccaguuccgcccauuccuauugugccuuuaaguaauuucagaaaugga
....................acuggcauaacucugacca.....................................................................   7    0    seq
....................acuggcauaacucugaccG.....................................................................   57   1    seq
.....................cuggcauaacucugacA......................................................................   2    1    seq
.....................cuggcauaacucugacc......................................................................   80   0    seq
.....................cuggcauaacucugacU......................................................................   13   1    seq
.....................cuggcauaacucugaccG.....................................................................   74   1    seq
.....................cuggcauaacucugaccU.....................................................................   2    1    seq
.....................cuggcauaacucugacca.....................................................................   10   0    seq
......................uggcauaacucugaccG.....................................................................   7    1    seq
.......................................uaUuuuauggucagaguu...................................................   1    1    seq
..........................................uuuauggucagaguuuug................................................   3    0    seq
..............................................uggucagaguuuugccGguuc.........................................   1    1    seq
..............................................uggucagaguuuugccGguucc........................................   3    1    seq
...............................................ggucagaguuuugccGguucc........................................   5    1    seq
................................................gucagaguuuugccGgu...........................................   1    1    seq
................................................gucagaguuuugccGguucc........................................   1    1    seq
.................................................ucagaguuuugccGguu..........................................   38   1    seq
.................................................ucagaguuuugccaguu..........................................   1    0    seq
.................................................ucagaguuuugccGguuc.........................................   5    1    seq
.................................................ucagaguuuugccGguucc........................................   36   1    seq
.................................................ucagaguuuugccGguuccgc......................................   1    1    seq
.................................................ucagaguuuugccaguuccgU......................................   1    1    seq
.................................................ucagaguuuugccaguuccgcc.....................................   25   0    seq
.................................................ucagaguuuugccaguuccgcU.....................................   8    1    seq
.................................................ucagaguuuugccaguuccgcA.....................................   3    1    seq
.................................................ucagaguuuugccaUuuccgcc.....................................   1    1    seq
.................................................ucagaguuuugccagGuccgcc.....................................   1    1    seq
.................................................ucagaguuuugccaguuccAcc.....................................   7    1    seq
.................................................ucagaguuuugccGguuccgcc.....................................   10   1    seq
..................................................cagaguuuugccGguucc........................................   1    1    seq
...................................................agaguuuugccGguucc........................................   2    1    seq
...................................................agaguuuugccGguuccgcc.....................................   1    1    seq
....................................................gaguuuugccaguuccgc......................................   1    0    seq
....................................................gaguuuugccaguuccgccc....................................   1    0    seq
....................................................gaguuuugccaguuccgcccauuccC..............................   2    1    seq
....................................................gaguuuugccaguuccgcccauuccua.............................   2    0    seq
....................................................gaguuuugccaguuccgcccaCuccuau............................   1    1    seq
```

Provisional ID	:	MA_175993_15617
Score total	:	1.5
Score for star read(s)	:	-1.3
Score for read counts	:	0
Score for mfe	:	1.2
Score for randfold	:	1.6
Score for cons. seed	:	
Total read count	:	106
Mature read count	:	106
Loop read count	:	0
Star read count	:	0

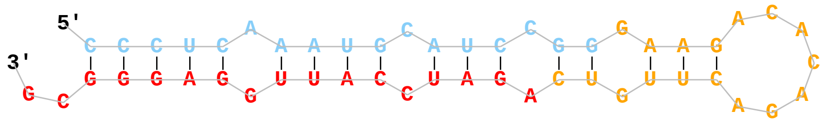

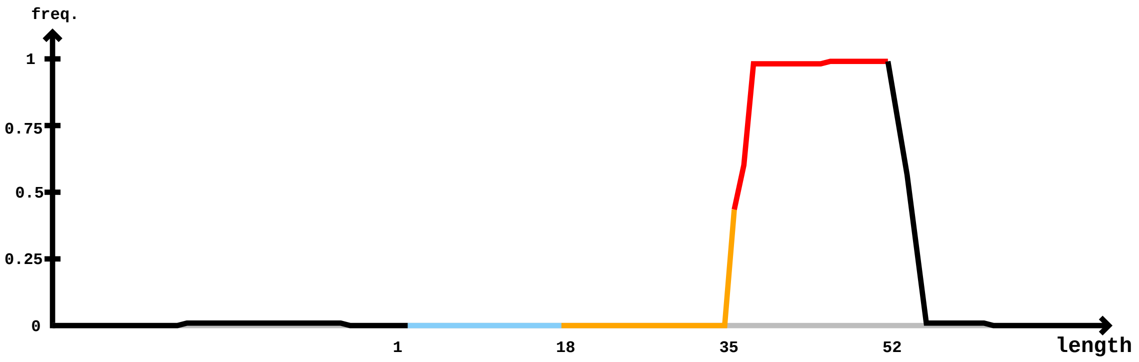

Star Mature

```
5'- aggccuaauuucacugaucacaugggaacaaucgucccucaaaugcauccgggaagacacagacuugucagauccauuggagggcgaggagggggaaguaguggcuuu   -3'    exp
    (((((..(((((.((..((...((....)).(((.(((((.((((.(((.((.(((.......))).)).))).)))).)))))))).))..)))))))...))))).   reads     mm      sample
    ...........Gcugaucacaugggaac................................................................................   1         1       seq
    .....................................................................Ggauccauuggagggcg......................   45        1       seq
    .....................................................................Ggauccauuggagggcgag....................   2         1       seq
    ......................................................................gauccauuggagggcga.....................   1         0       seq
    ......................................................................gauccauuggagggcgCg....................   1         1       seq
    ......................................................................gauccauuggagggcgag....................   8         0       seq
    ......................................................................gauccauuggagggcgUg....................   2         1       seq
    ......................................................................gauccauuggagggcgGg....................   1         1       seq
    ......................................................................gauccauuggagggcgagC...................   1         1       seq
    ......................................................................gauccauuggagggcgagA...................   1         1       seq
    ......................................................................gauccauuggagggcgagU...................   3         1       seq
    .......................................................................auccauuggagggcCag....................   1         1       seq
    .......................................................................auccauuggagggcgUg....................   1         1       seq
    .......................................................................auccauuggagggcgGg....................   2         1       seq
    .......................................................................auccauuggagggcgag....................   12        0       seq
    .......................................................................auccauuggaggAcgagg...................   1         1       seq
    .......................................................................auccauuggagggcgagC...................   2         1       seq
    .......................................................................auccauuggagggcgagU...................   21        1       seq
    .......................................................................auccauuggagggcgagA...................   1         1       seq
    ...............................................................................gagggcgaggaUgggga............   1         1       seq
```

Provisional ID	:	MA_179779_22247
Score total	:	0.8
Score for star read(s)	:	-1.3
Score for read counts	:	0
Score for mfe	:	0.5
Score for randfold	:	1.6
Score for cons. seed	:	
Total read count	:	514
Mature read count	:	510
Loop read count	:	4
Star read count	:	0

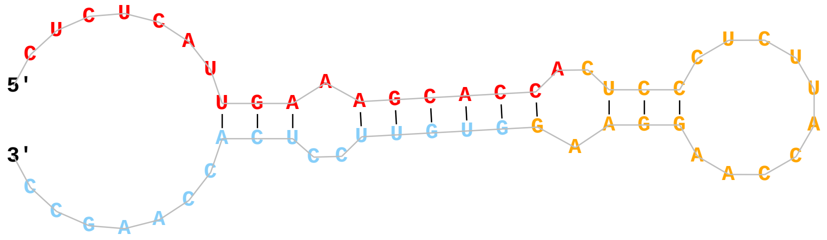

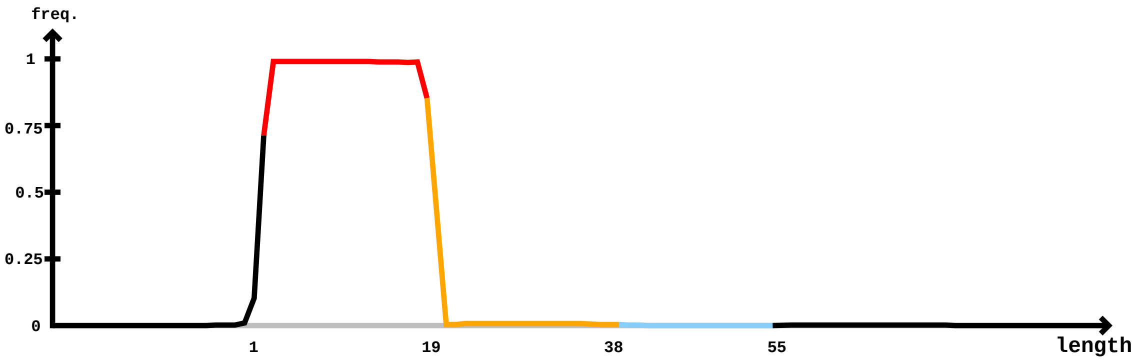

Mature **Star**

```
5'- ugggcucccguacaaaugggcucucauugaaagcaccacucccucuuaccaaggaagguguuccucaccaagcccaucuugucucugaaauagacaugcuugccccgu  -3'   exp
    .((((.........(((((((.....(((.((((((..(((...........))).))))))..)))...)))))))..(((((.......))))).....))))...   reads   mm   sample
    ...............auUggcucucauugaaa............................................................................   1       1    seq
    ..................Agcucucauugaaagca.........................................................................   1       1    seq
    ..................Agcucucauugaaagcacca......................................................................   2       1    seq
    ..................gAcucucauugaaagcacca......................................................................   1       1    seq
    ...................Ccucucauugaaagcacc.......................................................................   1       1    seq
    ...................gcucucauugaaagcacc.......................................................................   4       0    seq
    ...................Acucucauugaaagcacc.......................................................................   3       1    seq
    ...................gcucucauGgaaagcacca......................................................................   1       1    seq
    ...................Acucucauugaaagcacca......................................................................   10      1    seq
    ...................gcucucaCugaaagcacca......................................................................   1       1    seq
    ...................Ccucucauugaaagcacca......................................................................   2       1    seq
    ...................gcucucauugaaaUcacca......................................................................   1       1    seq
    ...................gAucucauugaaagcacca......................................................................   2       1    seq
    ...................Ncucucauugaaagcacca......................................................................   1       1    seq
    ...................gcucucauugaaagcacca......................................................................   20      0    seq
    ...................gcucucaGugaaagcacca......................................................................   1       1    seq
    ...................gcucucauuAaaagcacca......................................................................   1       1    seq
    ....................cucucauugaaagcacU.......................................................................   7       1    seq
    ....................cucucaGugaaagcacc.......................................................................   4       1    seq
    ....................cucucauugUaagcacc.......................................................................   1       1    seq
    ....................cucucauuCaaagcacc.......................................................................   1       1    seq
    ....................cucucauuUaaagcacc.......................................................................   1       1    seq
    ....................cucucauuAaaagcacc.......................................................................   5       1    seq
    ....................cucucauugaaagcacc.......................................................................   44      0    seq
    ....................cucucauugGaagcacca......................................................................   2       1    seq
    ....................cucucauCgaaagcacca......................................................................   1       1    seq
    ....................cucucauugaaagcaccU......................................................................   3       1    seq
    ....................cucucauuAaaagcacca......................................................................   16      1    seq
    ....................cucucaCugaaagcacca......................................................................   2       1    seq
    ....................cucucGuugaaagcacca......................................................................   1       1    seq
    ....................Uucucauugaaagcacca......................................................................   2       1    seq
    ....................cucucauugaaagcaccG.....................................................................   6       1    seq
    ....................cucucaGugaaagcacca......................................................................   5       1    seq
    ....................cucucauugaaagcaccC.....................................................................   2       1    seq
```

Mature **Star**

```
ugggcucccguacaaaugggcucucauugaaagcaccacucccucuuaccaaggaagguguuccucaccaagcccaucuugucucugaaauagacaugcuugccccgu
....................cucucauuUaaagcacca......................................................................    2      1     seq
....................cucucauugaaagcacca......................................................................    206    0     seq
....................cucucauugaaagcGcca......................................................................    3      1     seq
....................cucucauugaaagcaccac.....................................................................    1      0     seq
.....................ucucauugaaagcaccC......................................................................    1      1     seq
.....................ucCcauugaaagcacca......................................................................    1      1     seq
.....................ucucauugaaagcaccG......................................................................    6      1     seq
.....................ucucaCugaaagcacca......................................................................    1      1     seq
.....................ucucauugaaagcaccU......................................................................    2      1     seq
.....................ucucauuAaaagcacca......................................................................    8      1     seq
.....................ucucauugaaGgcacca......................................................................    1      1     seq
.....................ucucaGugaaagcacca......................................................................    3      1     seq
.....................ucucauuCaaagcacca......................................................................    1      1     seq
.....................Ccucauugaaagcacca......................................................................    2      1     seq
.....................ucucauugaaagcacca......................................................................    117    0     seq
....................................cacucccucuuaGcaagga.....................................................    1      1     seq
.....................................acucccucuuaccGagg......................................................    1      1     seq
.........................................ccucuuaccGaggaagg..................................................    1      1     seq
.........................................ccucuuaccGaggaaggug................................................    1      1     seq
...........................................................................aucuugucucugaaaua................    1      0     seq
```

Provisional ID : MA_180436_15354
Score total : 11304.5
Score for star read(s) : 3.9
Score for read counts : 11296.8
Score for mfe : 2.2
Score for randfold : 1.6
Score for cons. seed :
Total read count : 22170
Mature read count : 21840
Loop read count : 1
Star read count : 329

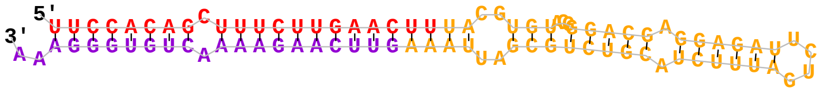

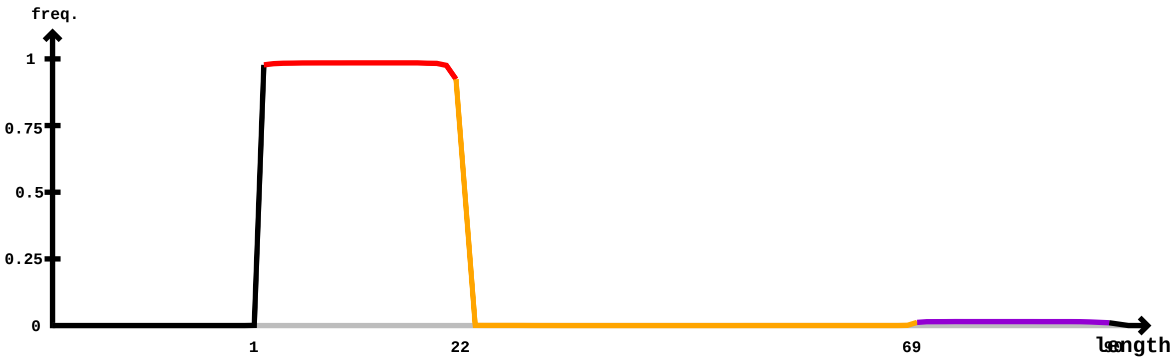


```
                    Mature                                                       Star
5'- ugacgaaucuugucauacuuuuccacagcuuucuugaacuuuacguguacgggacgaggagauucugauuucuacgucugcgauuaaaguucaagaaaacugugggaaagca  -3'   obs
    ugacgaaucuugucauacuuuuccacagcuuucuugaacuuuacguguacgggacgaggagauucugauuucuacgucugcgauuaaaguucaagaaaacugugggaaagca        exp
    ((((((...))))))..((((((((((.((((((((((((((..(((...(((((.((((((....)))))).))))))))..))))))))))))).))))))))))..    reads   mm   sample
    ...................uuuccacagcuuucuuga...........................................................................    1       0    seq
    ...................uuuccacagcuuucuugaacC........................................................................    2       1    seq
    ...................uuuccacagcuuCcuugaacu........................................................................    1       1    seq
    ...................uuuccacagcuuucuugaacu........................................................................    10      0    seq
    ...................Cuuccacagcuuucuugaacuu.......................................................................    1       1    seq
    ...................uCuccacagcuuucuugaacuuua.....................................................................    1       1    seq
    ....................uuccacagcuuucuugG...........................................................................    2       1    seq
    ....................uuccacagcuuucuuga...........................................................................    28      0    seq
    ....................uuccacagcuuucuugaa..........................................................................    12      0    seq
    ....................uuccacagcuAucuugaac.........................................................................    1       1    seq
    ....................uuccacagcuuucuugaaU.........................................................................    7       1    seq
    ....................uuccGcagcuuucuugaac.........................................................................    1       1    seq
    ....................uuccacagcuuucuCgaac.........................................................................    1       1    seq
    ....................uuccacagcuuucCugaac.........................................................................    1       1    seq
    ....................uGccacagcuuucuugaac.........................................................................    1       1    seq
    ....................uuccacagcCuucuugaac.........................................................................    1       1    seq
    ....................uuccacagcuuucuugaac.........................................................................    136     0    seq
    ....................uCccacagcuuucuugaacu........................................................................    6       1    seq
    ....................uAccacagcuuucuugaacu........................................................................    1       1    seq
    ....................uuccacagcAuucuugaacu........................................................................    1       1    seq
    ....................uuccacagcuuucuugaacG........................................................................    24      1    seq
    ....................uuccacagcuuuGuugaacu........................................................................    1       1    seq
    ....................Auccacagcuuucuugaacu........................................................................    1       1    seq
    ....................uuccacagcuuucuugaGcu........................................................................    2       1    seq
    ....................uuccacagcCuucuugaacu........................................................................    3       1    seq
    ....................uuccacagcuuucCugaacu........................................................................    2       1    seq
    ....................Cuccacagcuuucuugaacu........................................................................    8       1    seq
    ....................uuccacagcuuucuugaaUu........................................................................    3       1    seq
    ....................uuccacagcuuucuugaacA........................................................................    23      1    seq
    ....................Guccacagcuuucuugaacu........................................................................    1       1    seq
    ....................uuccaUagcuuucuugaacu........................................................................    2       1    seq
    ....................uuccacagcuuCcuugaacu........................................................................    2       1    seq
    ....................uuccacagcuuucuCgaacu........................................................................    1       1    seq
```

Mature **Star**

```
ugacgaaucuugucauacuuuuccacagcuuucuugaacuuuacguguacgggacgaggagauucugauuucuacgucugcgauuaaaguucaagaaaacugugggaaagca
....................uuccGcagcuuucuugaacu........................................................................ 3 1 seq
....................uuccacagcuuucuugaacC........................................................................ 153 1 seq
....................uuccacagcuuucuugaacu........................................................................ 848 0 seq
....................uGccacagcuuucuugaacuu....................................................................... 1 1 seq
....................uuccacagcuuucuAgaacuu....................................................................... 8 1 seq
....................uuccUcagcuuucuugaacuu....................................................................... 12 1 seq
....................uuccacagcuuucGugaacuu....................................................................... 2 1 seq
....................uuccacagcCuucuugaacuu....................................................................... 59 1 seq
....................uuccacagcuuucAugaacuu....................................................................... 10 1 seq
....................uuccacagcuCucuugaacuu....................................................................... 30 1 seq
....................uuccacagcuuuUuugaacuu....................................................................... 5 1 seq
....................uuccacagcuuucuGgaacuu....................................................................... 8 1 seq
....................uuccaGagcuuucuugaacuu....................................................................... 2 1 seq
....................uuAcacagcuuucuugaacuu....................................................................... 5 1 seq
....................uuccacagcuuucuugaacuN....................................................................... 1 1 seq
....................uucUacagcuuucuugaacuu....................................................................... 14 1 seq
....................uuccGcagcuuucuugaacuu....................................................................... 53 1 seq
....................uucGacagcuuucuugaacuu....................................................................... 1 1 seq
....................uuccacagcuuAcuugaacuu....................................................................... 1 1 seq
....................uuccacaAcuuucuugaacuu....................................................................... 8 1 seq
....................Guccacagcuuucuugaacuu....................................................................... 22 1 seq
....................uuccacaUcuuucuugaacuu....................................................................... 1 1 seq
....................uuccacagUuuucuugaacuu....................................................................... 11 1 seq
....................uAccacagcuuucuugaacuu....................................................................... 10 1 seq
....................Auccacagcuuucuugaacuu....................................................................... 12 1 seq
....................uuccacCgcuuucuugaacuu....................................................................... 3 1 seq
....................uuccacagcuuucuugaacCu....................................................................... 55 1 seq
....................uuccacUgcuuucuugaacuu....................................................................... 9 1 seq
....................uuccacagcuuuGuugaacuu....................................................................... 4 1 seq
....................uuccacagcuuucuugaacuu....................................................................... 16608 0 seq
....................uuccacagcuuucuugCacuu....................................................................... 5 1 seq
....................uuccacagcuuucuugaaGuu....................................................................... 3 1 seq
....................uuccacagcuAucuugaacuu....................................................................... 5 1 seq
....................uuccacagAuuucuugaacuu....................................................................... 1 1 seq
....................uuccacagcuuucuugaCcuu....................................................................... 1 1 seq
....................uuccaUagcuuucuugaacuu....................................................................... 13 1 seq
....................uuccacagcuuucuugaaAuu....................................................................... 2 1 seq
....................uuccacagcuuucuCgaacuu....................................................................... 51 1 seq
....................uuccacagcuuucuugaaUuu....................................................................... 12 1 seq
....................uuccacagcAuucuugaacuu....................................................................... 3 1 seq
....................uCccacagcuuucuugaacuu....................................................................... 46 1 seq
....................uuccacagcuuucuugUacuu....................................................................... 9 1 seq
....................uuccacagcuuucCugaacuu....................................................................... 41 1 seq
....................uuccacagcuuucuugaacuC....................................................................... 2434 1 seq
....................uuccacagcuuucuugaacAu....................................................................... 12 1 seq
....................uuccacagGuuucuugaacuu....................................................................... 3 1 seq
....................uuGcacagcuuucuugaacuu....................................................................... 6 1 seq
....................Nuccacagcuuucuugaacuu....................................................................... 25 1 seq
....................uuccCcagcuuucuugaacuu....................................................................... 3 1 seq
....................uuccacagcuGucuugaacuu....................................................................... 8 1 seq
....................uuccacagcuuucuugaacuG....................................................................... 271 1 seq
....................uuccacagcuuucuugGacuu....................................................................... 58 1 seq
....................uuccacagcuuCcuugaacuu....................................................................... 43 1 seq
....................uuccacagcuuucuugaacGu....................................................................... 28 1 seq
....................uuUcacagcuuucuugaacuu....................................................................... 13 1 seq
....................uucAacagcuuucuugaacuu....................................................................... 1 1 seq
....................uuccacagcuuucuuAaacuu....................................................................... 3 1 seq
....................uuccacagcuuucuugaGcuu....................................................................... 61 1 seq
....................Cuccacagcuuucuugaacuu....................................................................... 64 1 seq
....................uuccacagcGuucuugaacuu....................................................................... 2 1 seq
....................uuccacagcuuucuugaacuuC...................................................................... 31 1 seq
....................uuccacagcuuucuCgaacuuu...................................................................... 1 1 seq
....................uuccGcagcuuucuugaacuuu...................................................................... 1 1 seq
....................uuccacagcuuucuugaGcuuu...................................................................... 1 1 seq
....................uuccacagcuCucuugaacuuu...................................................................... 1 1 seq
....................uuccacagcuuucuugaacuuu...................................................................... 167 0 seq
....................Cuccacagcuuucuugaacuuu...................................................................... 1 1 seq
....................uuccacagcuuucuugaacuAu...................................................................... 2 1 seq
....................uCccacagcuuucuugaacuuu...................................................................... 1 1 seq
....................uuccacGgcuuucuugaacuuu...................................................................... 1 1 seq
```

```
                    Mature                                                                  Star
ugacgaaucuugucauacuuuuccacagcuuucuugaacuuuacguguacgggacgaggagauucugauuucuacgucugcgauuaaaguucaagaaaacugugggaaagca
....................uuccacagcuuucuugaacuuGa.....................................................................	1	1	seq
....................uuccacagcuuucuugaacuuuU.....................................................................	1	1	seq
....................uuccacagcuuucuugaacuuCac....................................................................	1	1	seq
.....................uccacagcuuucuugaac.........................................................................	7	0	seq
.....................uccacUgcuuucuugaacu........................................................................	1	1	seq
.....................uccacagcuuucuugaacu........................................................................	20	0	seq
.....................uccacagcuuucuugaacC........................................................................	7	1	seq
.....................Cccacagcuuucuugaacuu.......................................................................	1	1	seq
.....................uUcacagcuuucuugaacuu.......................................................................	2	1	seq
.....................uccacagcuuucuugUacuu.......................................................................	1	1	seq
.....................uccacagcuuucuugaacuu.......................................................................	29	0	seq
.....................uccacagcuuucuugaacuC.......................................................................	1	1	seq
.....................uccacagcuuucuugaacuuC......................................................................	4	1	seq
.....................uccacagcuuucuugaacuuu......................................................................	23	0	seq
.....................uccacagcuuucuugaacuuua.....................................................................	2	0	seq
......................ccacagcuuucuugaac.........................................................................	8	0	seq
......................ccacagcuuucuugaaU.........................................................................	2	1	seq
......................ccacagcuuucuugaaA.........................................................................	1	1	seq
......................ccacagcuuucuugaacC........................................................................	1	1	seq
......................ccacagcuuucuugaacG........................................................................	1	1	seq
......................ccacagcuuucuugaacu........................................................................	18	0	seq
......................ccacagcuuucuugaacuC.......................................................................	1	1	seq
......................ccacagcuuucuugaacuu.......................................................................	5	0	seq
.......................cacagcuuucuugaacC........................................................................	1	1	seq
.......................cacagcuuucuugaacu........................................................................	1	0	seq
.......................cacagcuuucuugaacuu.......................................................................	4	0	seq
.......................cacagcuuucuugaacuC.......................................................................	1	1	seq
.......................cacagcuuucuugaacuuuacguguac..............................................................	1	0	seq
.......................cacagcuuucuugaacuuuacguguacgg............................................................	1	0	seq
........................acagcuuucuugaacuu.......................................................................	10	0	seq
........................acagcuuucuugaacuC.......................................................................	2	1	seq
........................acagcuCucuugaacuu.......................................................................	1	1	seq
........................acagcuuucuugaacuuuacgug.................................................................	1	0	seq
.........................cagcuuucuugaacuuu......................................................................	1	0	seq
.........................cagcuuucuugaacuuuacgugu................................................................	1	0	seq
.........................cagcuuucuugaacuuuacguguacg.............................................................	1	0	seq
..........................agcuuucuugaacuuuacgugu................................................................	1	0	seq
.............................uuucuugaacuuuacgugu................................................................	2	0	seq
.............................uuucuugaacuuuacgugGa...............................................................	1	1	seq
...............................ucuugaacuuuacgugu................................................................	1	0	seq
..................................................................gauuucuacgucugUgauuaaaguucaag.................	1	1	seq
......................................................................ucuacgucugUgauuaaaguuca...................	1	1	seq
..............................................................................ugUgauuaaaguucaagaaaacu...........	1	1	seq
.......................................................................................aguucaagaaaacuguggg......	1	0	seq
.......................................................................................aguucaagaaaacuguggga.....	4	0	seq
.......................................................................................aguucaagaaaacugugggaa....	3	0	seq
.......................................................................................aguucaagaaaacugugggaaa...	8	0	seq
.......................................................................................aguucaagaaGacugugggaaa...	1	1	seq
........................................................................................guucaagaaaacugugg.......	4	0	seq
........................................................................................guucaUgaaaacugugg.......	1	1	seq
........................................................................................Nuucaagaaaacuguggg......	1	1	seq
........................................................................................guucaagaaaacuguggg......	13	0	seq
........................................................................................guucaagaaaacuguggA......	2	1	seq
........................................................................................guucaagaaaacugugggG.....	3	1	seq
........................................................................................guucaagaaaacuguggga.....	23	0	seq
........................................................................................guucaagaaaacugugggGa....	1	1	seq
........................................................................................guucaagaaaacugugggaG....	1	1	seq
........................................................................................guucaagaaaacugugggaa....	20	0	seq
........................................................................................guucaagaaaacCgugggaa....	1	1	seq
........................................................................................guucaagaaaacugugggaU....	1	1	seq
........................................................................................guuUaagaaaacugugggaaa...	1	1	seq
........................................................................................guucaagaaaacugugggaGa...	1	1	seq
........................................................................................Auucaagaaaacugugggaaa...	1	1	seq
........................................................................................guucGagaaaacugugggaaa...	1	1	seq
........................................................................................guucaagaaaGcugugggaaa...	1	1	seq
........................................................................................guucaGgaaaacugugggaaa...	3	1	seq
........................................................................................guucaagaaaacugugggaaG...	1	1	seq
........................................................................................guucaagaaaacugAgggaaa...	1	1	seq
........................................................................................guucaagaaaacugugggaaa...	162	0	seq
........................................................................................guucaagaaaacugugggaaaC..	1	1	seq
```

```
                    Mature                                                                  Star
ugacgaaucuugucauacuuuuccacagcuuucuugaacuuuacguguacgggacgaggagauucugauuucuacgucugcgauuaaaguucaagaaaacugugggaaagca
........................................................................................guucaagaaaacugugggaaaA..    1    1    seq
.........................................................................................uucaagaaaacuguggg......    5    0    seq
.........................................................................................uucaagaaaGcuguggga.....    1    1    seq
.........................................................................................uucaagaaaacuguggga.....    4    0    seq
.........................................................................................uucaagaaaacugugggaa....    5    0    seq
.........................................................................................Cucaagaaaacugugggaaa...    1    1    seq
.........................................................................................uucaagaaaacugugggaaa...    18   0    seq
.........................................................................................uucaagaaaacugugggaaG...    2    1    seq
.........................................................................................uucaagaaaacugugggaaaU..    20   1    seq
..........................................................................................ucaagaaaacugugggaaaU..    1    1    seq
..........................................................................................ucaagaaaacugugggaaaUc.    2    1    seq
...........................................................................................caagaaaacugugggaa....    1    0    seq
............................................................................................aagGaaacugugggaaa...    1    1    seq
............................................................................................aagaaaacugugggaaa...    6    0    seq
```

Provisional ID	:	MA_180988_20861
Score total	:	1.8
Score for star read(s)	:	-1.3
Score for read counts	:	0
Score for mfe	:	1.5
Score for randfold	:	1.6
Score for cons. seed	:	
Total read count	:	58
Mature read count	:	58
Loop read count	:	0
Star read count	:	0

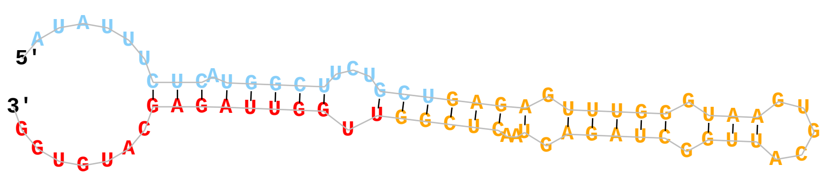

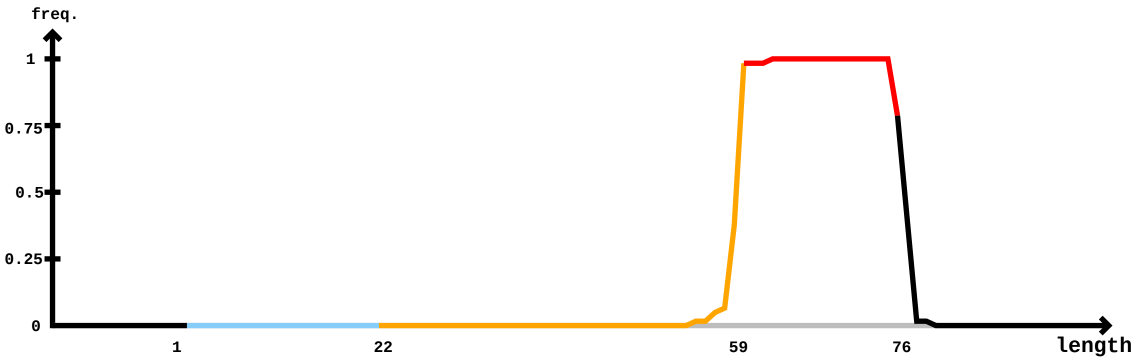

Star Mature

```
5'- ccacugaccgccauauuucucauggcuucugcugagaguuuggguaagugcauuggcuagaguaacucgguugguuagagcaugugguuguuaguguugccgguucau   -3'   exp
    .(((((((.(((((((..(((.(((((...(((((((.(((((.(((.....))).))))).)..)))))).)))))))).))))))).)))))))............   reads   mm   sample
    ..................................................................cucAguugguuagagcauguggu....................   1   1   seq
    ....................................................................cAguugguuagagcauguggu....................   2   1   seq
    .....................................................................Aguugguuagagcauguggu....................   1   1   seq
    .....................................................................guugguuagagcaugGg......................   1   1   seq
    .....................................................................guugguuagagcaugug......................   12   0   seq
    .....................................................................guugguuagagcaugugg.....................   3   0   seq
    .....................................................................guugguuagagcauguggu....................   3   0   seq
    ......................................................................uugguuagagcaugugg.....................   21   0   seq
    ......................................................................uCgguuagagcaugugg.....................   1   1   seq
    ......................................................................uugguuagagcaugugA.....................   1   1   seq
    ......................................................................uugguuagagcaugugU.....................   1   1   seq
    ......................................................................uugguuGgagcauguggu....................   1   1   seq
    ......................................................................uugguuagagcauguggA....................   1   1   seq
    ......................................................................uugguuagagcauguggu....................   9   0   seq
    ......................................................................Cugguuagagcauguggu....................   1   1   seq
    ......................................................................Gugguuagagcauguggu....................   1   1   seq
    .........................................................................guuagagcauguggAug..................   1   1   seq
```

Provisional ID	:	MA_182381_17082
Score total	:	1.5
Score for star read(s)	:	-1.3
Score for read counts	:	0
Score for mfe	:	1.2
Score for randfold	:	1.6
Score for cons. seed	:	
Total read count	:	38
Mature read count	:	38
Loop read count	:	0
Star read count	:	0

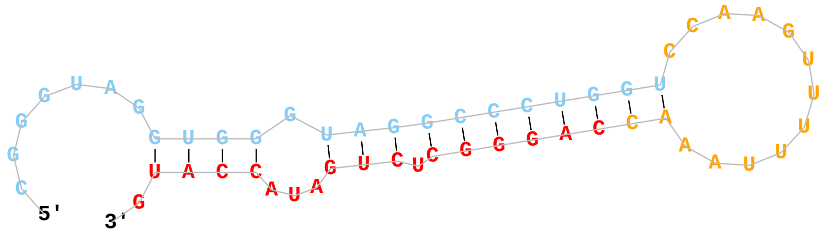

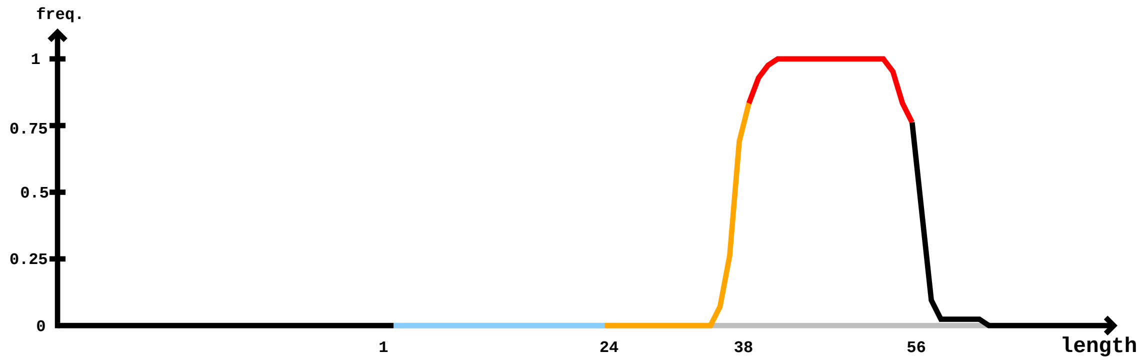


```
                                  Star                                 Mature
5'- acaauuccacuccugaauuguccuucuuacacgcggguagguggguaggcccugguccaaguuuuuaaaccagggcucugauaccauguguaaggucucuguccccac   -3'   exp
    (((((((.......)))))))...(((((((((.((....((.((..(((((((((............))))))))))).)).)).)))))))))).............   reads   mm   sample
    ...................................................................Uaccagggcucugauaccaug....................   2   1   seq
    ...................................................................Naccagggcucugauaccaugu...................   1   1   seq
    ....................................................................accagggcucugauacc.......................   2   0   seq
    ....................................................................accagggcucugauacca......................   1   0   seq
    ....................................................................aAcagggcucugauaccau.....................   1   1   seq
    ....................................................................accagggcucugauaccaug....................   1   0   seq
    ....................................................................accGgggcucugauaccaugu...................   1   1   seq
    ....................................................................acUagggcucugauaccaugu...................   1   1   seq
    ....................................................................accagggcucugauaccaugu...................   1   0   seq
    .....................................................................ccagggUucugauacca......................   1   1   seq
    .....................................................................ccagggcucugauaGca......................   1   1   seq
    .....................................................................ccagggcucugauacca......................   2   0   seq
    .....................................................................ccagggcucugauaccau.....................   2   0   seq
    .....................................................................ccagggcucugauaccauA....................   1   1   seq
    .....................................................................cUagggcucugauaccaug....................   2   1   seq
    .....................................................................ccagggcucugauaccaug....................   3   0   seq
    .....................................................................ccagggcucugauGccaug....................   1   1   seq
    .....................................................................Acagggcucugauaccaugu...................   1   1   seq
    .....................................................................ccagggcucugauaccaugu...................   3   0   seq
    .....................................................................ccagggcucugauaccauguA..................   1   1   seq
    ......................................................................cagggcucugauaccaug....................   5   0   seq
    ......................................................................caAggcucugauaccaug....................   1   1   seq
    .......................................................................aggAcucugauaccaug....................   1   1   seq
    .......................................................................agAgcucugauaccaugu...................   1   1   seq
    .......................................................................agggcucugauaccauguU..................   1   1   seq
    .......................................................................aUggcucugauaccauguguaagg.............   1   1   seq
    ........................................................................gAgcucugauaccaugu...................   1   1   seq
    ........................................................................gCgcucugauaccaugu...................   1   1   seq
    .........................................................................ggcucugauaccauguA..................   1   1   seq
```

Provisional ID	:	MA_18358_1017
Score total	:	3
Score for star read(s)	:	-1.3
Score for read counts	:	0
Score for mfe	:	2.7
Score for randfold	:	1.6
Score for cons. seed	:	
Total read count	:	37
Mature read count	:	37
Loop read count	:	0
Star read count	:	0

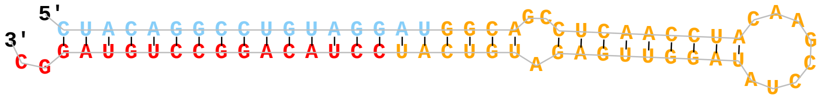

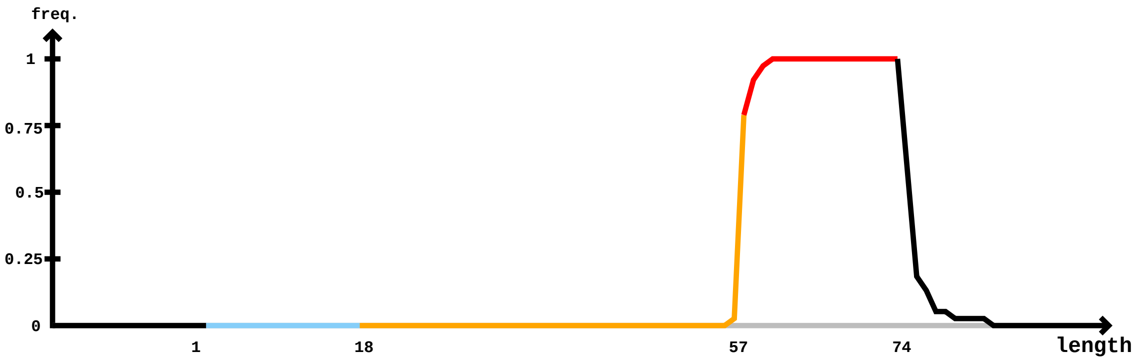

Star Mature

```
5'- gaggccaauucauccuacaggccuguaggauggcagccucaaccuacaagccuauagguugagaugucauccuacaggccuguaggcugagaaggcaaccuacaggcc  -3'   exp
    ..((((..((((.((((((((((((((((((((((..(((((((((........))))))))).))))))))))))))))))))))).))))..(....).....))))   reads   mm   sample
    .....................................................................uccuacaggccuguGggcu....................   1       1    seq
    ......................................................................ccuacaggccuguaggc.....................   8       0    seq
    ......................................................................ccuaUaggccuguaggc.....................   1       1    seq
    ......................................................................ccuacaggccuguaggcC....................   1       1    seq
    ......................................................................ccuacaggccuguaggcu....................   8       0    seq
    ......................................................................ccuacaAgccuguaggcu....................   2       1    seq
    ......................................................................ccuacaggUcuguaggcu....................   1       1    seq
    ......................................................................ccuacaggcUuguaggcu....................   2       1    seq
    ......................................................................ccuacaggccuguGggcu....................   1       1    seq
    ......................................................................ccuaUaggccuguaggcu....................   3       1    seq
    ......................................................................ccuacaggccuguaggcug...................   1       0    seq
    ......................................................................ccuacaggccuguaggcugU..................   1       1    seq
    .......................................................................cuGcaggccuguaggcu....................   1       1    seq
    .......................................................................cuacaggccuguaggcu....................   2       0    seq
    .......................................................................cuacaggccuguaggcugU..................   1       1    seq
    .......................................................................cuacaggccuguaggcugC..................   1       1    seq
    ........................................................................uacaggcAuguaggcug...................   1       1    seq
    ........................................................................uacaggccuguaggcugagaUggc............   1       1    seq
    .........................................................................acaggccuguaggcugUga................   1       1    seq
```

Provisional ID	:	MA_18480_839
Score total	:	2.6
Score for star read(s)	:	-1.3
Score for read counts	:	0
Score for mfe	:	2.3
Score for randfold	:	1.6
Score for cons. seed	:	
Total read count	:	141
Mature read count	:	141
Loop read count	:	0
Star read count	:	0

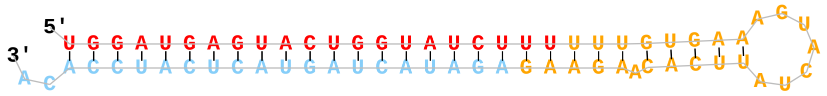

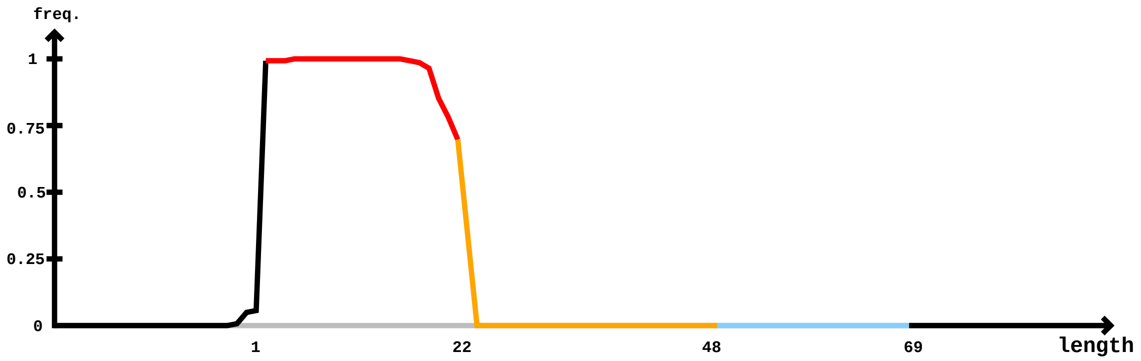

Mature Star

```
5'- ggaaauaaaggauuuaguuguggaugaguacugguaucuuuuuugugaaaguacuauucacaagaagagauacuaguacucauccacaauggaaucuucuuuaaacua   -3'    exp
    .....((((((((((.(((((((((((((((((((((((((((((((((.......))))).))))))))))))))))))))))))))))).))))).))))).....   reads    mm    sample
    .................uuguggaugaguacuggu.........................................................................   1        0     seq
    ..................uguggaugaguacugguauc......................................................................   2        0     seq
    ..................uguggaugaguacugguaucC.....................................................................   1        1     seq
    ..................uguggaugaguacugguaucu.....................................................................   3        0     seq
    ...................guggaugaguacuggua........................................................................   1        0     seq
    ....................uggaugUguacugguau.......................................................................   1        1     seq
    ....................uggaugaguacugguau.......................................................................   2        0     seq
    ....................uggaugaguacugguauc......................................................................   12       0     seq
    ....................uggaugaguGcugguauc......................................................................   2        1     seq
    ....................uggaugaguacugguaucu.....................................................................   4        0     seq
    ....................uggaugaguacugguaucC.....................................................................   2        1     seq
    ....................Cggaugaguacugguaucuu....................................................................   1        1     seq
    ....................uggaugaguacugguaucuu....................................................................   11       0     seq
    ....................uggaugaguacugguaucuuu...................................................................   2        0     seq
    ....................uggaugaguacugguaucuuC...................................................................   91       1     seq
    ....................uggaugaguacugguaucuuCu..................................................................   5        1     seq
    .......................augaguacugguaucuuCu..................................................................   1        1     seq
```

Provisional ID	:	MA_18480_840
Score total	:	1
Score for star read(s)	:	-1.3
Score for read counts	:	0
Score for mfe	:	0.7
Score for randfold	:	1.6
Score for cons. seed	:	
Total read count	:	141
Mature read count	:	141
Loop read count	:	0
Star read count	:	0

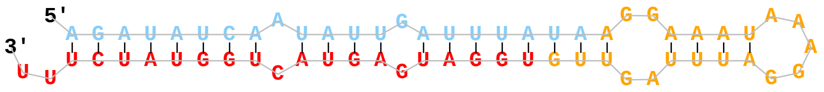

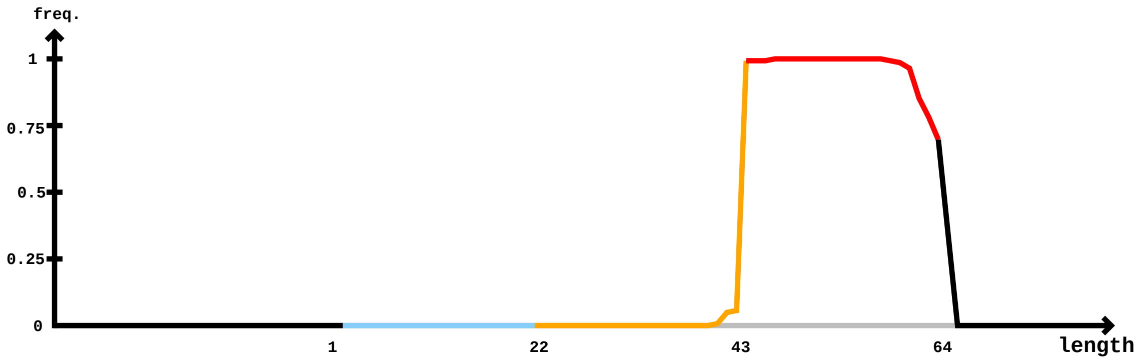

Star Mature

```
5'- uguuugugcaugaacacuuuucacaagaagauaucaauauugauuuauaaggaaauaaaggauuuaguuguggaugaguacugguaucuuuuuugugaaaguacuauu  -3'   exp
    (((((......))))).((((((((((((((((((.((((.((((((((..((((.....))))..)))))))).)))).))))))).)))))))))))).........    reads   mm   sample
    ...................................................................uuguggaugaguacuggu.......................    1       0    seq
    ....................................................................uguggaugaguacugguauc....................    2       0    seq
    ....................................................................uguggaugaguacugguaucC...................    1       1    seq
    ....................................................................uguggaugaguacugguaucu...................    3       0    seq
    .....................................................................guggaugaguacuggua......................    1       0    seq
    ......................................................................uggaugUguacugguau.....................    1       1    seq
    ......................................................................uggaugaguacugguau.....................    2       0    seq
    ......................................................................uggaugaguacugguauc....................    12      0    seq
    ......................................................................uggaugaguGcugguauc....................    2       1    seq
    ......................................................................uggaugaguacugguaucu...................    4       0    seq
    ......................................................................uggaugaguacugguaucC...................    2       1    seq
    ......................................................................Cggaugaguacugguaucuu..................    1       1    seq
    ......................................................................uggaugaguacugguaucuu..................    11      0    seq
    ......................................................................uggaugaguacugguaucuuu.................    2       0    seq
    ......................................................................uggaugaguacugguaucuuC.................    91      1    seq
    ......................................................................uggaugaguacugguaucuuCu................    5       1    seq
    .........................................................................augaguacugguaucuuCu................    1       1    seq
```

Provisional ID	:	MA_185559_5578
Score total	:	2466.3
Score for star read(s)	:	3.9
Score for read counts	:	2458.5
Score for mfe	:	2.3
Score for randfold	:	1.6
Score for cons. seed	:	
Total read count	:	4834
Mature read count	:	4798
Loop read count	:	0
Star read count	:	36

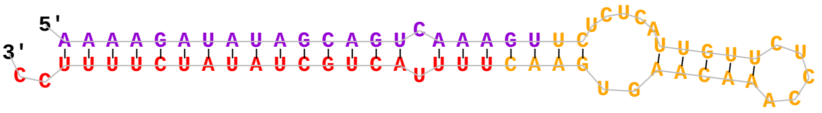

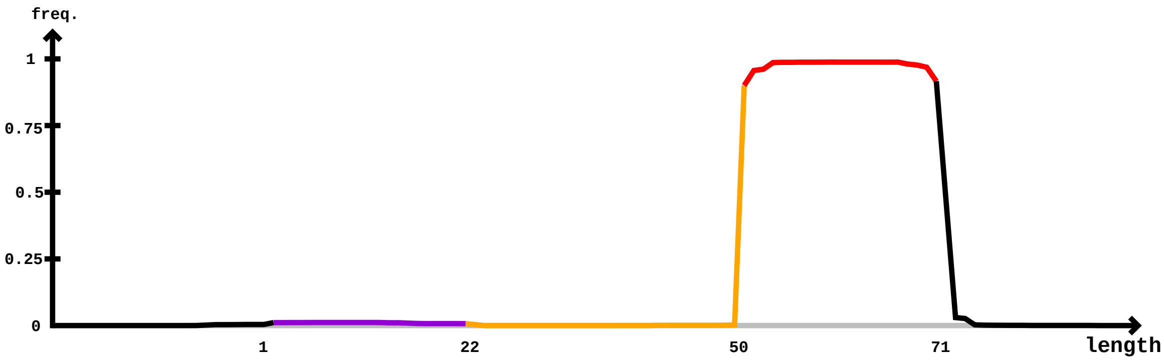


```
                                    Star                                              Mature
5'- agcaguaaaagugccagaaggaaaagauauagcagucaaaguucucucauuguucuccaaacaagugaacuuuuacugcuauaucuuuuccuucuugcaauuuuaccauug  -3'  obs
    agcaguaaaagugccagaaggaaaagauauagcagucaaaguucucucauuguucuccaaacaagugaacuuuuacugcuauaucuuuuccuucuugcaauuuuaccauug       exp
    ....((((((.(((.(((((((((((((((((((((.(((((((.....(((((.....)))))..))))))).))))))))))))))))))))).))).)))))).....   reads   mm   sample
    .............cUagaaggaaaagauauagc..............................................................................   1       1    seq
    ..............Uagaaggaaaagauauagc..............................................................................   2       1    seq
    ..............Uagaaggaaaagauauagcag............................................................................   4       1    seq
    ..............cagaaggaaaagauauagcag............................................................................   1       0    seq
    ..............Uagaaggaaaagauauagcagu...........................................................................   1       1    seq
    ...............agaaggaaaagauauagc..............................................................................   2       0    seq
    ...............agaaggaaaagauauagcagu...........................................................................   5       0    seq
    ...............agaaggaaaagauauagcagC...........................................................................   1       1    seq
    .................aaggaaaagauauagcaguc..........................................................................   1       0    seq
    ..................aggaaaagauauagcag............................................................................   1       0    seq
    ..................aggaaaagauauagcaguU..........................................................................   1       1    seq
    .....................aaaagauauagcagucaaag......................................................................   1       0    seq
    .....................aaaagauauagcagucaaagG.....................................................................   1       1    seq
    .....................aaaagauauagcaguUaaagu.....................................................................   25      1    seq
    .....................aaaagauauagcagucaaagu.....................................................................   7       0    seq
    .......................aagauauagcaguUaaagu.....................................................................   1       1    seq
    .........................gauauagcaguUaaaguu....................................................................   1       1    seq
    .........................................................caaacaCgugaacuuuuacugcuauaucuuu.......................   1       1    seq
    .............................................................caCgugaacuuuuacugcu...............................   1       1    seq
    .............................................................caCgugaacuuuuacugcuauauc..........................   1       1    seq
    .............................................................caagugaacuuuuacugcuauaucuu........................   1       0    seq
    ..............................................................aCgugaacuuuuacugcuauaucuu........................   1       1    seq
    ...............................................................Cgugaacuuuuacugcuauaucuuuuc.....................   1       1    seq
    ....................................................................acuuuuacugcuauaucuuuu......................   1       0    seq
    .....................................................................cuuuuacugcuauaucuuuuc.....................   4       0    seq
    ......................................................................uuuuacugcuauaucuu........................   27      0    seq
    ......................................................................uuuuGcugcuauaucuu........................   1       1    seq
    ......................................................................uuuuacugcuauaucuA........................   1       1    seq
    ......................................................................uuuuacugcuauaucuC........................   5       1    seq
    ......................................................................uuuuacugcuauaucuuC.......................   2       1    seq
    ......................................................................uuuuacugcuauaucuuu.......................   13      0    seq
    ......................................................................Cuuuacugcuauaucuuuu......................   1       1    seq
    ......................................................................uuuuacugcuauaucuuuu......................   31      0    seq
```

```
                    Star                                              Mature
agcaguaaaagugccagaaggaaaagauauagcagucaaaguucucucauuguucuccaaacaagugaacuuuuacugcuauaucuuuuccuucuugcaauuuuaccauug
......................................................................uuuuacugcuauaucuuuC......................    3     1    seq
......................................................................uuuuacugcuauaucuCuuc.....................    1     1    seq
......................................................................uuuuacugcuauaucuuuCc.....................    2     1    seq
......................................................................uuGuacugcuauaucuuuuc.....................    1     1    seq
......................................................................uuuuacugcuauaucuuuuc.....................    222   0    seq
......................................................................uuuuacugcuauaucuuuuA.....................    1     1    seq
......................................................................uuuuGcugcuauaucuuuuc.....................    2     1    seq
......................................................................uuuuacugcuauaucuuuuU.....................    6     1    seq
......................................................................uuuuacugcuauaucuuuAc.....................    1     1    seq
......................................................................uuuuacugcuauaCcuuuuc.....................    2     1    seq
......................................................................Auuuacugcuauaucuuuuc.....................    1     1    seq
......................................................................uuuuacugcuauaucuuuuAc....................    5     1    seq
......................................................................uuuuacugcuUuaucuuuucc....................    2     1    seq
......................................................................uuuCacugcuauaucuuuucc....................    5     1    seq
......................................................................uuuuGcugcuauaucuuuucc....................    7     1    seq
......................................................................uuuuacugcuauaucuuCucc....................    12    1    seq
......................................................................uuuuUcugcuauaucuuuucc....................    1     1    seq
......................................................................uuuuacugcuauaGcuuuucc....................    1     1    seq
......................................................................uuGuacugcuauaucuuuucc....................    2     1    seq
......................................................................uuuuacugcuCuaucuuuucc....................    1     1    seq
......................................................................uCuuacugcuauaucuuuucc....................    9     1    seq
......................................................................uuuuacugcuauaucuuuucG....................    3     1    seq
......................................................................uuuuacugcuauaucAuuucc....................    1     1    seq
......................................................................uuuuacugcuauaucuuuuUc....................    7     1    seq
......................................................................uuuuacCgcuauaucuuuucc....................    5     1    seq
......................................................................uuuuacugcuGuaucuuuucc....................    6     1    seq
......................................................................uuuuacugcuaCaucuuuucc....................    6     1    seq
......................................................................uuuuacugAuauaucuuuucc....................    1     1    seq
......................................................................uuuuacugcuauaucuuuCcc....................    4     1    seq
......................................................................uuuuacugcuauGucuuuucc....................    6     1    seq
......................................................................uuuuacugcuauaucuCuucc....................    5     1    seq
......................................................................Cuuuacugcuauaucuuuucc....................    16    1    seq
......................................................................uuuuacugUuauaucuuuucc....................    3     1    seq
......................................................................Nuuuacugcuauaucuuuucc....................    5     1    seq
......................................................................uuuuacugcuauauAuuuucc....................    2     1    seq
......................................................................Guuuacugcuauaucuuuucc....................    6     1    seq
......................................................................Auuuacugcuauaucuuuucc....................    1     1    seq
......................................................................uuuuaUugcuauaucuuuucc....................    3     1    seq
......................................................................uuuuacugcCauaucuuuucc....................    10    1    seq
......................................................................uuuuacugcuauaucuuuucc....................    3508  0    seq
......................................................................uuuuacuAcuauaucuuuucc....................    3     1    seq
......................................................................uuuuacugcuauaucuuuucU....................    244   1    seq
......................................................................uuuuacugcuauauUuuuucc....................    1     1    seq
......................................................................uuCuacugcuauaucuuuucc....................    4     1    seq
......................................................................uuuuacugcuauaucCuuucc....................    29    1    seq
......................................................................uuuuacugcuauaucuuuucA....................    84    1    seq
......................................................................uuuuacGgcuauaucuuuucc....................    4     1    seq
......................................................................uuuuacugcuauaCcuuuucc....................    8     1    seq
......................................................................uGuuacugcuauaucuuuucc....................    1     1    seq
......................................................................uAuuacugcuauaucuuuucc....................    1     1    seq
......................................................................uuuuacAgcuauaucuuuuccu...................    3     1    seq
......................................................................uuuuacugcuauaucuuuuccA...................    2     1    seq
......................................................................uuuuacugcuauaucuuuuccu...................    8     0    seq
......................................................................uuuuacugcuauaucuuuucUu...................    2     1    seq
......................................................................uuuuacugcuauaucuuuuUcu...................    1     1    seq
......................................................................uuuuacugcuauaucuuuuccC...................    3     1    seq
......................................................................uuuuacugcuauaucuuuuccuGc.................    1     1    seq
......................................................................uuuuacugcuauaucuuuuccuuU.................    2     1    seq
......................................................................uuuuacugcuauaucuuuuccuuAu................    1     1    seq
......................................................................uuuuacugcuauaucuuuuccCucu................    1     1    seq
......................................................................uuuuacugcuauaucuuuuccuuUuu...............    1     1    seq
.......................................................................uuuacugcuauaucuuC.......................    1     1    seq
.......................................................................uuuacugcuauaucuuu.......................    1     0    seq
.......................................................................uuuacugcuauaucuuuu......................    3     0    seq
.......................................................................uuuacugcuauaucuuuC......................    1     1    seq
.......................................................................uuuacugcuauaucuuuuc.....................    6     0    seq
.......................................................................uuuacCgcuauaucuuuucc....................    2     1    seq
.......................................................................uuuacugcuauaucuuuucc....................    27    0    seq
.......................................................................uuuacugcuauaucuuuucU....................    2     1    seq
.......................................................................uuuacugcuauaucuuuucAu...................    2     1    seq
```

```
                     Star                                               Mature
agcaguaaaagugccagaaggaaaagauauagcagucaaaguucucucauuguucuccaaacaagugaacuuuacugcuauaucuuuuccuucuugcaauuuuaccauug
......................................................................uuuacugUuauaucuuuuccu...................   1     1     seq
......................................................................Cuuacugcuauaucuuuuccu...................   1     1     seq
......................................................................uuuacugcuauaucuuuuccu...................   171   0     seq
......................................................................uuuacugcuauaucCuuuccu...................   2     1     seq
......................................................................uuuacAgcuauaucuuuuccu...................   1     1     seq
......................................................................uuuacugcuauaucuuuuccC...................   30    1     seq
......................................................................uuuaGugcuauaucuuuuccu...................   1     1     seq
......................................................................uuuacugcuauaucuuuuUcu...................   1     1     seq
......................................................................uuuacugcuauaucuuuuccG...................   6     1     seq
......................................................................uuuacugcuauGucuuuuccu...................   1     1     seq
......................................................................uuuacugcuauaucuuuuccA...................   11    1     seq
......................................................................uuuGcugcuauaucuuuuccu...................   1     1     seq
......................................................................uuuacugcuauaucuuuuccuu..................   1     0     seq
.......................................................................uuacugcuauaucuuuuc.....................   3     0     seq
.......................................................................uuacugcuauaucuuuucU....................   1     1     seq
.......................................................................uuacugcuauaucuuuucc....................   4     0     seq
.......................................................................uuacugcuauaucuuuuccuu..................   12    0     seq
.......................................................................uuacugcuauaucuuuuccuC..................   3     1     seq
.......................................................................uuacugcuauaucuuuuccuG..................   1     1     seq
........................................................................uacugcuauaucuuuuc.....................   3     0     seq
........................................................................uacugcuauaucuuuucc....................   4     0     seq
........................................................................uacugcuauaucuuuuccu...................   2     0     seq
........................................................................uacugcuauaucuuuuccuuc.................   101   0     seq
........................................................................uGcugcuauaucuuuuccuuc.................   1     1     seq
........................................................................uacugcuauaucuuuuccuuA.................   2     1     seq
........................................................................uacCgcuauaucuuuuccuuc.................   1     1     seq
........................................................................uacugcuauaucuuuuccuuU.................   4     1     seq
........................................................................uacugcuauaucuuuCccuuc.................   1     1     seq
........................................................................uacugcuGuaucuuuuccuuc.................   1     1     seq
........................................................................uacugcuauaCcuuuuccuuc.................   1     1     seq
.........................................................................acugcuauaucuuuuccuuc.................   1     0     seq
.........................................................................acugcuauaucuuuuccuucA................   1     1     seq
.........................................................................acugcuauaucuuuuccuucu................   1     0     seq
.........................................................................acugcuauaucuuuuccuucC................   1     1     seq
...........................................................................ugcuauaucuuuuccuucuu...............   1     0     seq
...........................................................................ugcuauaucuuuuccuucuug..............   1     0     seq
...........................................................................ugcuauaucuuuuccCucuugc.............   1     1     seq
..............................................................................uauaucuuuuccuucuugcaa...........   2     0     seq
......................................................................................uuccuucuugcaauuuuacA....   2     1     seq
```

Provisional ID : MA_185559_5580
Score total : 2468
Score for star read(s) : 3.9
Score for read counts : 2460.5
Score for mfe : 2
Score for randfold : 1.6
Score for cons. seed :
Total read count : 4838
Mature read count : 4798
Loop read count : 1
Star read count : 39

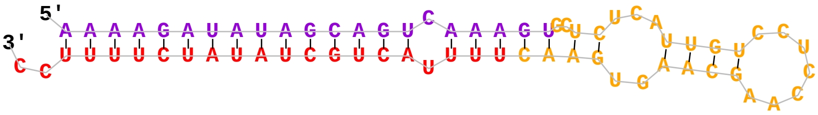

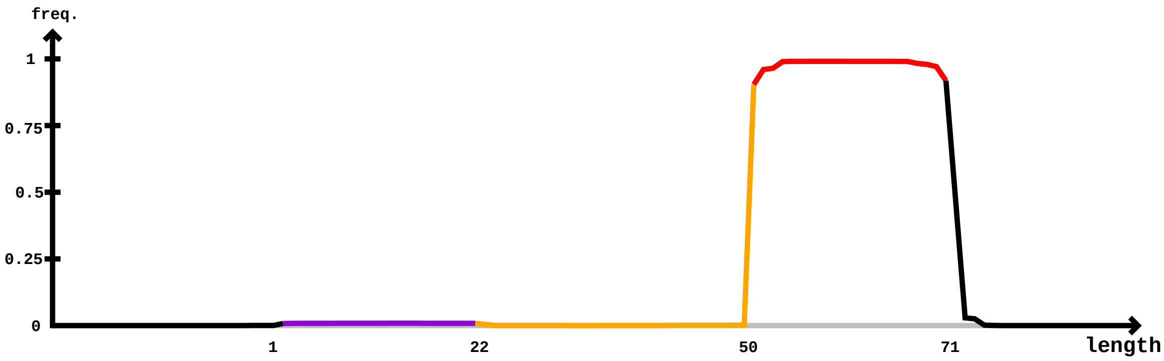

Star   Mature

```
5'- uaacaguaaaagugccagagggaaaagauauagcagucaaagugcucucauuguccuccaagcaagugaacuuuuacugcuauaucuuuuccuucuuacaauuuuaccaau   -3'    obs
    uaacaguaaaagugccagagggaaaagauauagcagucaaagugcucucauuguccuccaagcaagugaacuuuuacugcuauaucuuuuccuucuuacaauuuuaccaau          exp
    .....((((((.((..(((((((((((((((((((((.(((((..((...((((.......))))..))))))).))))))))))))))))))))..)).))))))....   reads   mm   sample
    ...................gggaaaagauauagcagC..........................................................................   1       1    seq
    ...................gggaaaagauauagcGgu..........................................................................   1       1    seq
    ......................aaaagauauagcagucaaag.....................................................................   1       0    seq
    ......................aaaagauauagcaguUaaagu....................................................................   25      1    seq
    ......................aaaagauauagcagucaaagG....................................................................   1       1    seq
    ......................aaaagauauagcagucaaagu....................................................................   7       0    seq
    ......................aaaagauauagcaguUaaagug...................................................................   1       1    seq
    ......................aaaagauauagcagucaaagugU..................................................................   1       1    seq
    .......................aaagauauagcaguUaaagug...................................................................   2       1    seq
    ........................aagauauagcaguUaaagu....................................................................   1       1    seq
    ............................uauagcagucaaGgugcucucauu...........................................................   1       1    seq
    .................................cagucaaagugcucucaCu...........................................................   1       1    seq
    .......................................................cuccaagcUagugaacu.......................................   1       1    seq
    ..............................................................caCgugaacuuuuacugcu..............................   1       1    seq
    ..............................................................caCgugaacuuuuacugcuauauc.........................   1       1    seq
    ..............................................................caagugaacuuuuacugcuauaucuu.......................   1       0    seq
    ...............................................................aCgugaacuuuuacugcuauaucuu.......................   1       1    seq
    ................................................................Cgugaacuuuuacugcuauaucuuuuc....................   1       1    seq
    .....................................................................acuuuuacugcuauaucuuuu.....................   1       0    seq
    ......................................................................cuuuuacugcuauaucuuuuc....................   4       0    seq
    .......................................................................uuuuacugcuauaucuA.......................   1       1    seq
    .......................................................................uuuuGcugcuauaucuu.......................   1       1    seq
    .......................................................................uuuuacugcuauaucuC.......................   5       1    seq
    .......................................................................uuuuacugcuauaucuu.......................   27      0    seq
    .......................................................................uuuuacugcuauaucuuC......................   2       1    seq
    .......................................................................uuuuacugcuauaucuuu......................   13      0    seq
    .......................................................................Cuuuacugcuauaucuuuu.....................   1       1    seq
    .......................................................................uuuuacugcuauaucuuuu.....................   31      0    seq
    .......................................................................uuuuacugcuauaucuuuC.....................   3       1    seq
    .......................................................................uuuuacugcuauaucuuuuU....................   6       1    seq
    .......................................................................uuuuacugcuauaucuuuuCc...................   2       1    seq
    .......................................................................uuGuacugcuauaucuuuuc....................   1       1    seq
    .......................................................................uuuuacugcuauaucuuuAc....................   1       1    seq
```

```
                     Star                                               Mature
uaacaguaaaagugccagagggaaaagauauagcagucaaagugcucucauuguccuccaagcaagugaacuuuuacugcuauaucuuuuccuucuuacaauuuuaccaau
.......................................................................uuuuacugcuauaCcuuuuc....................  2     1   seq
.......................................................................uuuuGcugcuauaucuuuuc....................  2     1   seq
.......................................................................Auuuacugcuauaucuuuuc....................  1     1   seq
.......................................................................uuuuacugcuauaucuCuuc....................  1     1   seq
.......................................................................uuuuacugcuauaucuuuuc....................  222   0   seq
.......................................................................uuuuacugcuauaucuuuuA....................  1     1   seq
.......................................................................uuCuacugcuauaucuuuucc...................  4     1   seq
.......................................................................uuuuacugcuauaCcuuuucc...................  8     1   seq
.......................................................................uuuuacCgcuauaucuuuucc...................  5     1   seq
.......................................................................uuuuacugcuauaucuuuucU...................  244   1   seq
.......................................................................uuuuaUugcuauaucuuuucc...................  3     1   seq
.......................................................................uuuuacugcuauauAuuuucc...................  2     1   seq
.......................................................................Guuuacugcuauaucuuuucc...................  6     1   seq
.......................................................................uuuuacuAcuauaucuuuucc...................  3     1   seq
.......................................................................uuuuacugcuauaucuuuucA...................  84    1   seq
.......................................................................Nuuuacugcuauaucuuuucc...................  5     1   seq
.......................................................................uuuuacugcuauaGcuuuucc...................  1     1   seq
.......................................................................Auuuacugcuauaucuuuucc...................  1     1   seq
.......................................................................uuuCacugcuauaucuuuucc...................  5     1   seq
.......................................................................uuuuacugcuauaucuuuucc...................  3508  0   seq
.......................................................................uuuuacugcuauaucuuuCcc...................  4     1   seq
.......................................................................uuuuacugcuUuaucuuuucc...................  2     1   seq
.......................................................................uuuuacugcuGuaucuuuucc...................  6     1   seq
.......................................................................uuGuacugcuauaucuuuucc...................  2     1   seq
.......................................................................uuuuacugcCauaucuuuucc...................  10    1   seq
.......................................................................uuuuGcugcuauaucuuuucc...................  7     1   seq
.......................................................................uAuuacugcuauaucuuuucc...................  1     1   seq
.......................................................................uuuuacugcuauGucuuuucc...................  6     1   seq
.......................................................................uuuuacugcuauaucAuuucc...................  1     1   seq
.......................................................................uuuuacugcuauaucCuuucc...................  29    1   seq
.......................................................................Cuuuacugcuauaucuuuucc...................  16    1   seq
.......................................................................uuuuacugAuauaucuuuucc...................  1     1   seq
.......................................................................uuuuacugcuCuaucuuuucc...................  1     1   seq
.......................................................................uuuuacugcuauaucuuuuUc...................  7     1   seq
.......................................................................uuuuUcugcuauaucuuuucc...................  1     1   seq
.......................................................................uuuuacugcuauaucuuuuAc...................  5     1   seq
.......................................................................uuuuacugcuauaucuuCucc...................  12    1   seq
.......................................................................uuuuacugUuauaucuuuucc...................  3     1   seq
.......................................................................uGuuacugcuauaucuuuucc...................  1     1   seq
.......................................................................uuuuacGgcuauaucuuuucc...................  4     1   seq
.......................................................................uuuuacugcuaCaucuuuucc...................  6     1   seq
.......................................................................uuuuacugcuauauUuuuucc...................  1     1   seq
.......................................................................uuuuacugcuauaucuCuucc...................  5     1   seq
.......................................................................uCuuacugcuauaucuuuucc...................  9     1   seq
.......................................................................uuuuacugcuauaucuuuucG...................  3     1   seq
.......................................................................uuuuacugcuauaucuuuucUu..................  2     1   seq
.......................................................................uuuuacugcuauaucuuuuccC..................  3     1   seq
.......................................................................uuuuacAgcuauaucuuuuccu..................  3     1   seq
.......................................................................uuuuacugcuauaucuuuuccu..................  8     0   seq
.......................................................................uuuuacugcuauaucuuuuUcu..................  1     1   seq
.......................................................................uuuuacugcuauaucuuuuccA..................  2     1   seq
.......................................................................uuuuacugcuauaucuuuuccuuU................  2     1   seq
.......................................................................uuuuacugcuauaucuuuuccuGc................  1     1   seq
.......................................................................uuuuacugcuauaucuuuuccuuAu...............  1     1   seq
.......................................................................uuuuacugcuauaucuuuuccCucu...............  1     1   seq
.......................................................................uuuuacugcuauaucuuuuccuuUuu..............  1     1   seq
........................................................................uuuacugcuauaucuuC......................  1     1   seq
........................................................................uuuacugcuauaucuuu......................  1     0   seq
........................................................................uuuacugcuauaucuuuC.....................  1     1   seq
........................................................................uuuacugcuauaucuuuu.....................  3     0   seq
........................................................................uuuacugcuauaucuuuuc....................  6     0   seq
........................................................................uuuacugcuauaucuuuucc...................  27    0   seq
........................................................................uuuacugcuauaucuuuucU...................  2     1   seq
........................................................................uuuacCgcuauaucuuuucc...................  2     1   seq
........................................................................uuuacugcuauGucuuuuccu..................  1     1   seq
........................................................................uuuaGugcuauaucuuuuccu..................  1     1   seq
........................................................................uuuacAgcuauaucuuuuccu..................  1     1   seq
........................................................................uuuGcugcuauaucuuuuccu..................  1     1   seq
........................................................................uuuacugcuauaucuuuuccC..................  30    1   seq
........................................................................uuuacugcuauaucuuuuccA..................  11    1   seq
```

Star Mature

```
uaacaguaaaagugccagagggaaaagauauagcagucaaagugcucucauuguccuccaagcaagugaacuuuuacugcuauaucuuuuccuucuuacaauuuuaccaau
........................................................................Cuuacugcuauaucuuuuccu..................   1     1    seq
........................................................................uuuacugcuauaucuuuuccu..................   171   0    seq
........................................................................uuuacugcuauaucuuuuccG..................   6     1    seq
........................................................................uuuacugUuauaucuuuuccu..................   1     1    seq
........................................................................uuuacugcuauaucuuuucAu..................   2     1    seq
........................................................................uuuacugcuauaucuuuuUcu..................   1     1    seq
........................................................................uuuacugcuauaucCuuuccu..................   2     1    seq
........................................................................uuuacugcuauaucuuuuccuu.................   1     0    seq
.........................................................................uuacugcuauaucuuuuc....................   3     0    seq
.........................................................................uuacugcuauaucuuuucc...................   4     0    seq
.........................................................................uuacugcuauaucuuuucU...................   1     1    seq
.........................................................................uuacugcuauaucuuuuccuC.................   3     1    seq
.........................................................................uuacugcuauaucuuuuccuu.................   12    0    seq
.........................................................................uuacugcuauaucuuuuccuG.................   1     1    seq
..........................................................................uacugcuauaucuuuuc....................   3     0    seq
..........................................................................uacugcuauaucuuuucc...................   4     0    seq
..........................................................................uacugcuauaucuuuuccu..................   2     0    seq
..........................................................................uacugcuauaucuuuCccuuc................   1     1    seq
..........................................................................uacugcuauaucuuuuccuuA................   2     1    seq
..........................................................................uacCgcuauaucuuuuccuuc................   1     1    seq
..........................................................................uacugcuauaucuuuuccuuU................   4     1    seq
..........................................................................uacugcuauaucuuuuccuuc................   101   0    seq
..........................................................................uacugcuGuaucuuuuccuuc................   1     1    seq
..........................................................................uacugcuauaCcuuuuccuuc................   1     1    seq
..........................................................................uGcugcuauaucuuuuccuuc................   1     1    seq
...........................................................................acugcuauaucuuuuccuuc................   1     0    seq
...........................................................................acugcuauaucuuuuccuucu...............   1     0    seq
...........................................................................acugcuauaucuuuuccuucA...............   1     1    seq
...........................................................................acugcuauaucuuuuccuucC...............   1     1    seq
.............................................................................ugcuauaucuuuuccuucuu..............   1     0    seq
```

Provisional ID : MA_18595_3427
Score total : 4412.6
Score for star read(s) : 3.9
Score for read counts : 4491.7
Score for mfe : -80.7
Score for randfold : -2.2
Score for cons. seed :
Total read count : 8822
Mature read count : 8821
Loop read count : 0
Star read count : 1

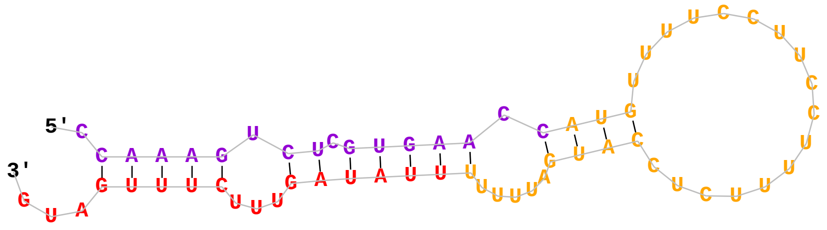

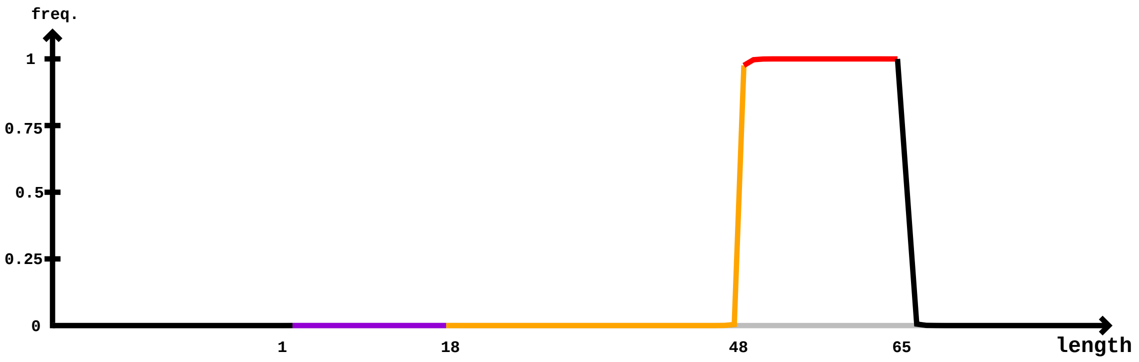

Star Mature

```
5'- auguguaauucucccuccaguuuccaaagucucgugaaccauguuuuccuuccuuuucuccaugauuuuuuuauaguuucuuugaugauguuaugaucuaguguaaaa -3'  obs
    auguguaauucucccuccaguuuccaaagucucgugaaccauguuuuccuuccuuuucuccaugauuuuuuuauaguuucuuugaugauguuaugaucuaguguaaaa       exp
    ..................(((..(((((.((.(((((.((((................)))).....)))))))...)))))..)))..................... reads  mm  sample
    .......................ccaaagucucUugaacc....................................................................  1     1   seq
    ....................................................................Nuuuauaguuucuuuga.......................  1     1   seq
    ....................................................................Nuuuauaguuucuuugaug.....................  1     1   seq
    ....................................................................uCuuauaguuucuuugaug.....................  1     1   seq
    ....................................................................Auuuauaguuucuuugauga....................  1     1   seq
    .....................................................................uuuauaguuucuuugaug.....................  10    0   seq
    .....................................................................uuGauaguuucuuugaug.....................  1     1   seq
    .....................................................................Nuuauaguuucuuugaug.....................  12    1   seq
    .....................................................................uuuauaguuucuuugaugG....................  1     1   seq
    .....................................................................uuuauaguuucuuugaugGu...................  6     1   seq
    .....................................................................Auuauaguuucuuugaugau...................  1     1   seq
    ......................................................................Nuauaguuucuuugaug.....................  10    1   seq
    ......................................................................uuauagGuucuuugaug.....................  2     1   seq
    ......................................................................uuauaguuAcuuugaug.....................  3     1   seq
    ......................................................................uuauaguuucuuugaug.....................  8104  0   seq
```
[truncated: 2,980,341 more chars]
