# Supplementary material for: Transcriptome Analysis of mRNA and miRNA in Somatic Embryos of Larix leptolepis Subjected to Hydrogen Treatment
Source: Int J Mol Sci. 2016 Nov 22;17(11):1951. doi: 10.3390/ijms17111951 (PMC5133945; doi:10.3390/ijms17111951)
Supplement: Supplementary file 1 [file ijms-17-01951-s001.zip › ijms-155406-Supplementary Materials/ijms-155406-supple.pdf]

Supplementary Materials: Transcriptome Analysis of mRNA and miRNA in Somatic Embryos of *Larix leptolepis* Subjected to Hydrogen Treatment

Yali Liu, Suying Han, Xiangming Ding, Xinmin Li, Lifeng Zhang, Wanfeng Li, Haiyan Xu, Zhexin Li and Liwang Qi

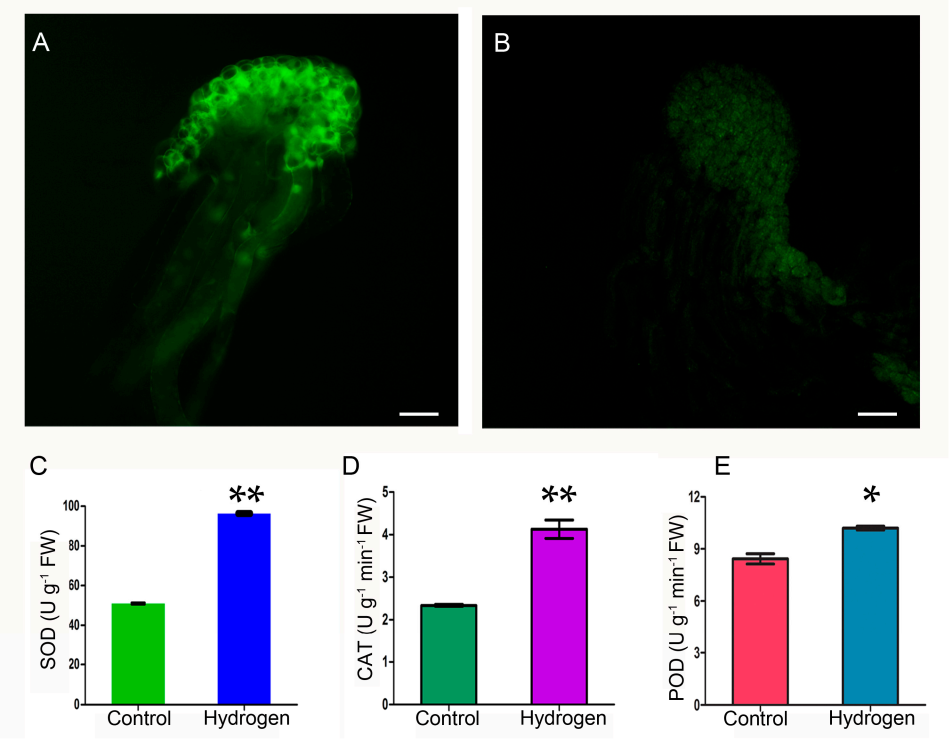

Figure S1. ROS levels and antioxidant enzyme activity in PEMs at 36 days after H_2 treatment. ROS levels in PEMs on the control medium (**A**); and hydrogen-rich medium (**B**); Effects of hydrogen treatment on superoxide dismutase (SOD) (**C**); catalase (CAT) (**D**); and peroxidase (POD) (**E**) activity in PEMs. Data represent means ± standard error from three independent experiments. Asterisks indicate significant difference at * $p < 0.05$ or ** $p < 0.01$ by Student's t test. Scale bars, 50 μm in (**A** and **B**).
